# A multitope SARS-CoV-2 vaccine provides long-lasting B cell and T cell immunity against Delta and Omicron variants

**DOI:** 10.1172/JCI157707

**Published:** 2022-05-16

**Authors:** Chang Yi Wang, Kao-Pin Hwang, Hui-Kai Kuo, Wen-Jiun Peng, Yea-Huei Shen, Be-Sheng Kuo, Juin-Hua Huang, Hope Liu, Yu-Hsin Ho, Feng Lin, Shuang Ding, Zhi Liu, Huan-Ting Wu, Ching-Tai Huang, Yuarn-Jang Lee, Ming-Che Liu, Yi-Ching Yang, Po-Liang Lu, Hung-Chin Tsai, Chen-Hsiang Lee, Zhi-Yuan Shi, Chun-Eng Liu, Chun-Hsing Liao, Feng-Yee Chang, Hsiang-Cheng Chen, Fu-Der Wang, Kuo-Liang Hou, Jennifer Cheng, Min-Sheng Wang, Ya-Ting Yang, Han-Chen Chiu, Ming-Han Jiang, Hao-Yu Shih, Hsuan-Yu Shen, Po-Yen Chang, Yu-Rou Lan, Chi-Tian Chen, Yi-Ling Lin, Jian-Jong Liang, Chun-Che Liao, Yu-Chi Chou, Mary Kate Morris, Carl V. Hanson, Farshad Guirakhoo, Michael Hellerstein, Hui-Jing Yu, Chwan-Chuen King, Tracy Kemp, D. Gray Heppner, Thomas P. Monath

**Affiliations:** 1 United Biomedical (UBI), Inc., Hauppauge, New York, USA.; 2 UBI Asia, Hsinchu, Taiwan.; 3 United BioPharma, Inc., Hsinchu, Taiwan.; 4 Division of Infectious Diseases, China Medical University Children’s Hospital and; 5 School of Medicine, College of Medicine, China Medical University, Taichung, Taiwan.; 6 StatPlus, Inc., Taipei, Taiwan.; 7 Linkou Chang Gung Memorial Hospital, Taoyuan, Taiwan.; 8 Taipei Medical University Hospital, Taipei, Taiwan.; 9 National Cheng Kung University Hospital, Tainan, Taiwan.; 10 Kaohsiung Medical University Chung-Ho Memorial Hospital, Kaohsiung, Taiwan.; 11 College of Medicine, Kaohsiung Medical University, Kaohsiung, Taiwan.; 12 Kaohsiung Veterans General Hospital, Kaoshiung, Taiwan.; 13 Chang Gung Memorial Hospital, Kaohsiung, Taiwan.; 14 Taichung Veterans General Hospital, Taichung, Taiwan.; 15 Changhua Christian Hospital, Changhua, Taiwan.; 16 Far Eastern Memorial Hospital, New Taipei, Taiwan.; 17 Tri-Service General Hospital, Taipei, Taiwan.; 18 Taipei Veterans General Hospital, Taipei, Taiwan.; 19 Institute of Biomedical Sciences and; 20 Biomedical Translation Research Center (BioTReC), Academia Sinica, Taipei, Taiwan.; 21 Viral and Rickettsial Disease Laboratory, California Department of Public Health, Richmond, California, USA.; 22 Vaxxinity Inc., Dallas, Texas, USA.; 23 Institute of Epidemiology and Preventive Medicine, College of Public Health, National Taiwan University, Taipei, Taiwan.

**Keywords:** COVID-19, Peptides

## Abstract

**Background:**

The Delta and Omicron variants of SARS-CoV-2 are currently responsible for breakthrough infections due to waning immunity. We report phase I/II trial results of UB-612, a multitope subunit vaccine containing S1-RBD-sFc protein and rationally designed promiscuous peptides representing sarbecovirus conserved helper T cell and cytotoxic T lymphocyte epitopes on the nucleocapsid (N), membrane (M), and spike (S2) proteins.

**Method:**

We conducted a phase I primary 2-dose (28 days apart) trial of 10, 30, or 100 μg UB-612 in 60 healthy young adults 20 to 55 years old, and 50 of them were boosted with 100 μg of UB-612 approximately 7 to 9 months after the second dose. A separate placebo-controlled and randomized phase II study was conducted with 2 doses of 100 μg of UB-612 (*n =* 3,875, 18–85 years old). We evaluated interim safety and immunogenicity of phase I until 14 days after the third (booster) dose and of phase II until 28 days after the second dose.

**Results:**

No vaccine-related serious adverse events were recorded. The most common solicited adverse events were injection site pain and fatigue, mostly mild and transient. In both trials, UB-612 elicited respective neutralizing antibody titers similar to a panel of human convalescent sera. The most striking findings were long-lasting virus-neutralizing antibodies and broad T cell immunity against SARS-CoV-2 variants of concern (VoCs), including Delta and Omicron, and a strong booster-recalled memory immunity with high cross-reactive neutralizing titers against the Delta and Omicron VoCs.

**Conclusion:**

UB-612 has presented a favorable safety profile, potent booster effect against VoCs, and long-lasting B and broad T cell immunity that warrants further development for both primary immunization and heterologous boosting of other COVID-19 vaccines.

**Trial Registration:**

ClinicalTrials.gov: NCT04545749, NCT04773067, and NCT04967742.

**Funding:**

UBI Asia, Vaxxinity Inc., and Taiwan Centers for Disease Control, Ministry of Health and Welfare.

## Introduction

The combined effects of SARS-CoV-2 neutralization-escape variants with high transmissibility by asymptomatic persons (1, 2) and breakthrough infections due to waning immunity of COVID-19 vaccines (3–5) continue to cost human lives and sap the world’s economy and healthcare system. While the currently authorized vaccines can prevent disease and reduce hospitalization and mortality, it is now clear that natural or vaccine immunity is short-lived and that boosters are required within a few months (6–8).

The current vaccines are manufactured with the original wild-type (WT) viral antigen. Antigenic variants Delta and Omicron have accounted for greater than 95% of all current infection cases (Supplemental Figure 1; supplemental material available online with this article; https://doi.org/10.1172/JCI157707DS1). Individuals infected with the Delta and Omicron variants can carry up to 1,000 times more virus in their nasal passages than those infected with other variants (9). People fully vaccinated with currently authorized vaccines can develop breakthrough cases, carry as much of the virus as unvaccinated people, and contribute to spread of the virus worldwide (10–12).

To maintain protection against Delta (13), the heavily mutated Omicron (B.1.1.529; ref. 14), and other ever-mutating SARS-CoV-2 strains, many regulatory agencies have approved a third dose, not only for the elderly, high-risk, and immunocompromised populations (12, 15–17), but also for healthy vaccinees who are at risk of the clinical consequences of the postvaccination drop in immunity (18–22).

Thus, beyond the durability of the 2-dose vaccine–induced immunity against breakthrough infections, the magnitude of the booster-recalled memory B and T cell immunities has become another point of consideration. The durability and magnitude issues are also applicable to natural infection, as there is reinfection with 0.7%–1.9% of cases occurring in individuals with documented prior infection (23). Moreover, the definition of “fully vaccinated” has been under discussion at the US CDC and FDA, with focus on the authorization for third and fourth booster vaccinations for certain populations, including those who are immunocompromised (24). Altogether, these underscore the importance of both the durability and memory effect of natural or vaccine-induced immunity.

While neutralizing antibody level correlates well with a vaccine’s protection efficacy (25, 26), substantial activation and expansion of antigen-specific CD4^+^ and CD8^+^ T cells are also critical for better duration of immunity and immunological memory (27, 28). Early induction of functional SARS-CoV-2–specific T cells has also been found to be critical for rapid viral clearance and amelioration of disease (29). Thus, T cell responses elicited by promiscuous helper T (Th) cell and cytotoxic T lymphocyte (CTL) peptides representing viral structural and nonstructural proteins are of increasing interest for assessment in the control of infection as the virus-derived peptides define heterologous and COVID-19–induced T cell recognition (30).

The development of immunogens that can induce CD4^+^ and CD8^+^ T cell responses to highly conserved epitopes across variants of concern (VoCs) of sarbecoviruses and can be recognized by individuals who have recovered from COVID-19 (31) could greatly augment current vaccines for SARS-CoV-2 given the emergence of variants that escape convalescent plasma and vaccine-induced antibody responses (32–36).

To the best of our knowledge, UB-612 represents the first rationally designed multitope protein/peptide subunit vaccine to activate both B and T cell immunities (37). It contains a CHO cell–produced spike protein receptor-binding domain (S1-RBD) fused with a single-chain Fc protein (S1-RBD-sFc), 5 promiscuous designer Th cell and CTL epitope peptides from the nucleocapsid (N), membrane (M), and S2 proteins of sarbecovirus, known to bind to multiple class I and class II human leukocyte antigens (HLAs) (38, 39), and an extrinsic HLA class II epitope (UBITh1a) modified from a measles virus fusion (MVF) protein that would serve as a catalyst for T cell activation (Supplemental Figure 2). The amino acid sequences for the 5 sarbecovirus peptides are highly conserved across all VoCs, including Delta and Omicron, allowing for induction of memory recall and T cell activation and effector functions in a broad population.

Here we report the results of 3 clinical trials, which include a 196-day phase I primary 2-dose series (28 days apart) of 10, 30, or 100 μg of UB-612 in healthy adults (*n =* 60) (NCT04545749), an interim 14-day phase I extension study with a 100 μg booster (*n =* 50) (NCT04967742), and an interim 56-day placebo-controlled phase II primary 2-dose study of UB-612 with a 100 μg dose (*n =* 3,875) (NCT04773067) that confirms the reproducibility of B cell and robust, broad, Th1-predominant T cell immunity. The 100 μg dose used in the phase I extension and phase II trials was selected as optimal in the initial phase I dose-ranging study.

UB-612 appeared to be safe and well tolerated. Two doses at a 28-day interval elicited long-lasting virus-neutralizing titers (*t_1/2_* of 187 days) and durable antigen-specific T cell responses. While inducing a modest level of neutralizing titer after 2 doses, a single booster dose prompted striking neutralizing antibodies against the original strain (hereafter referred to as the WT strain) isolated in Wuhan, China (geometric mean 50% virus-neutralizing titer [VNT_50_] of 3,992) associated with an unusually high cross-neutralization effect against the live Delta variant (VNT_50_ of 2,358, with a geometric mean fold reduction [GMFR] of 1.7 vs. WT) and Omicron (pseudovirus VNT_50_ [pVNT_50_] 2,325 with a GMFR of 5.2 vs. WT) strains, which rivalled titers observed with the most effective vaccines up to now and was predictive of greater than 90% efficacy (25, 26). The data suggest that UB-612 can induce immunological memory for profound B and T cell immunity when recalled by a vaccine booster or natural infection.

## Results

### Trial populations

#### Phase I primary and booster third-dose series.

**Figure 1:**
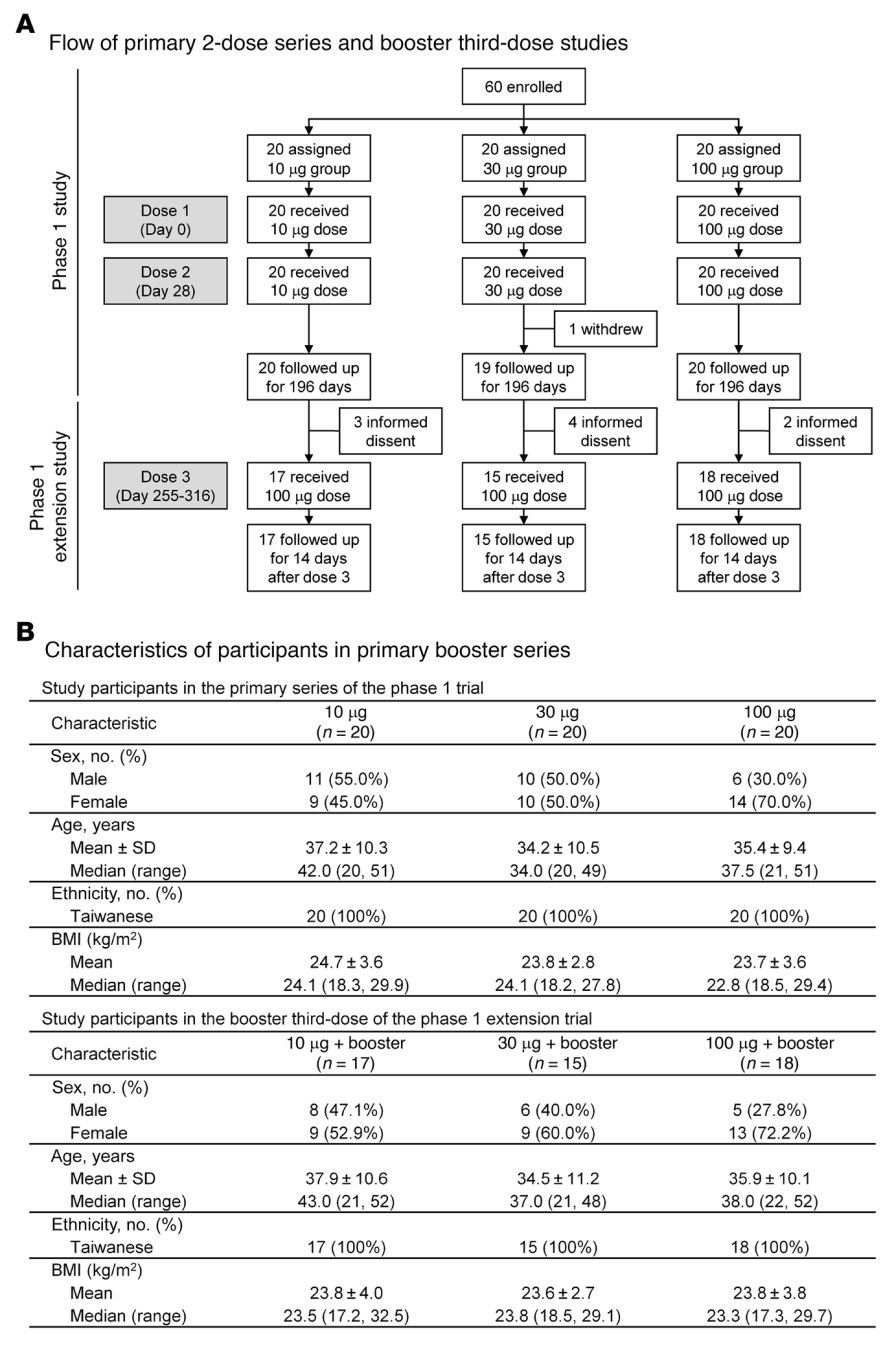
Flow of the UB-612 phase I trial primary 2-dose series with extended booster third-dose study and characteristics of study participants. (**A** and **B**) Sixty healthy young adults, male and female, 20 to 55 years old were enrolled for the primary series of the open-label, 196-day phase I study of UB-612 (NCT04545749), conducted between September 21, 2020 and May 24, 2021. They were administered intramuscularly with 2 vaccine doses at 10, 30, or 100 μg. All but one participant completed the study. The extension study (NCT04967742) that involved 50 enrollees was conducted from days 255 to 316, a time period over 6 months after the second vaccine shot. The 50 participants in the 10 μg (*n =* 17), 30 μg (*n =* 15), and 100 μg (*n =* 18) dose groups received a booster UB-612 dose of 100 μg and were followed up for 14 days for interim evaluation. They were monitored until 84 days after booster.

The characteristics of the open-label phase I trial participants (Figure 1, A and B) included the 196-day primary series study involving 60 healthy adults (20–55 years old) in 3 dose groups (*n =* 20 each) who received 2 doses (28 days apart) of UB-612 at 10, 30, or 100 μg; and the 84-day extension booster vaccination following the primary series, where 50 participants were enrolled to receive 1 additional 100 μg booster between 7.6 and 9.6 months after the second shot for the 10 μg (*n =* 17), 30 μg (*n =* 15), and 100 μg (*n =* 18) groups. The boosted participants were followed for 14 days for assessment of safety and immunogenicity in this interim report, and subsequently monitored until 84 days after booster.

#### Phase II primary 2-dose series.

**Figure 2:**
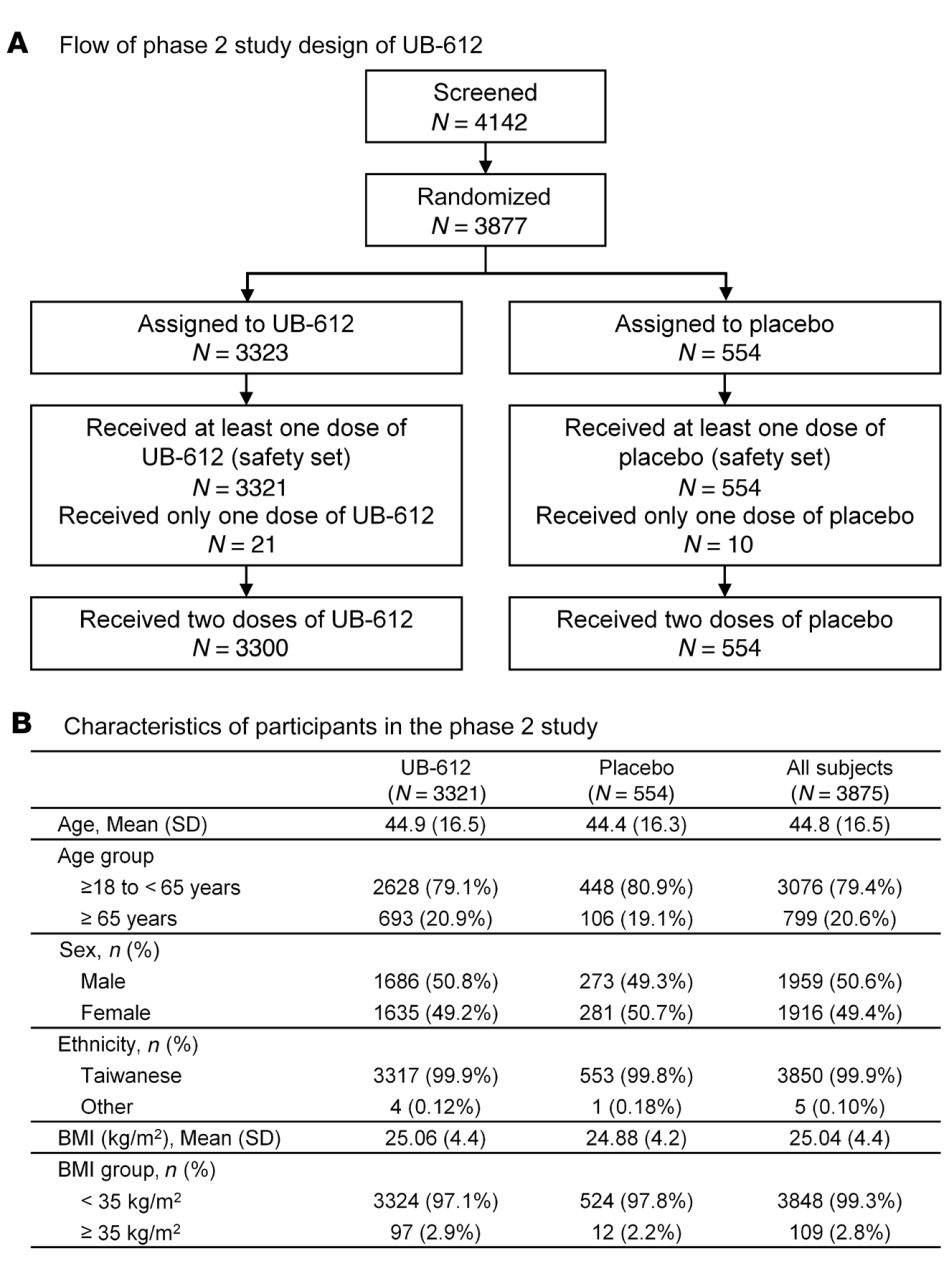
Flow of the UB-612 phase II study and characteristics of study participants. (**A** and **B**) The phase II trial (NCT04773067) was conducted between February 26, 2021 and April 16, 2021, and enrolled a total of 3,875 participants (18–85 years old) to receive 100 μg UB-612 (3,321 on 100 μg UB-612 and 554 on placebo at a 6:1 ratio).

The phase II trial was of a randomized and observer-blind design; participant characteristics are shown in Figure 2, A and B. A total of 3,875 participants who received at least 1 vaccine dose at 100 μg (3,321 received UB-612 and 554 received placebo at a 6:1 ratio) were enrolled and included in the safety population, of which 1,012 participants (vaccine 871 and placebo 141) were included in the evaluable immunogenicity population. The mean age of the participants receiving UB-612 was 44.9 years (range, 18–83 years) and that of placebo was 44.4 years (range, 19–84 years). The ratio of younger adults (18–65 years old) to elderly adults (≥65 years old) was approximately 80:20 for both UB-612 and placebo groups. All participants but 5 were Taiwanese.

### Reactogenicity and safety

#### Phase I primary 2-dose and booster third-dose series.

**Figure 3:**
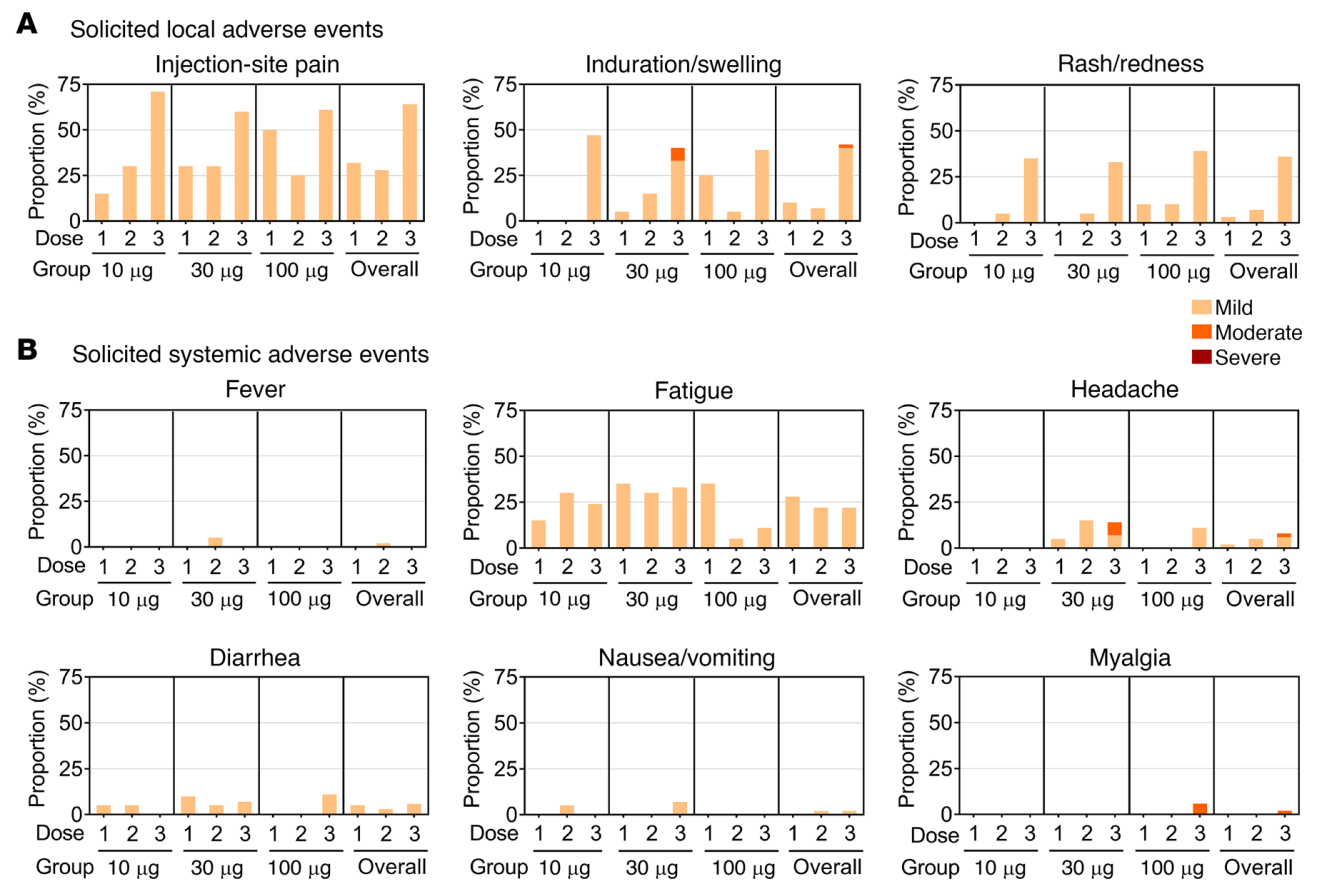
In phase I trial of primary and booster series, selected solicited local and systemic reactions within 7 days of each vaccination recorded for the 3 different doses of UB-612 vaccine. Both local and systemic reactions are shown as the percentage of participants who reported grade 1 (mild; yellow) or grade 2 (moderate, orange) for (**A**) local and (**B**) systemic adverse reactions. For dose 1 and dose 2, there were 20 participants in each dose group receiving 2 doses of UB-612 at 10, 30, or 100 μg. For the booster dose 3 at 100 μg, there were 17, 15, and 18 participants who originally were assigned to the 10, 30, and 100 μg dose groups, respectively.

In the 196-day primary series and up to 14 days after booster, neither vaccine-related severe adverse events (SAEs, including grade 3/4 AEs) nor dose-limited increase in incidence or severity was recorded. The solicited local and systemic AEs reported within 7 days in all vaccination groups (Figure 3A) were mild to moderate (grade 1/2) and transient, with lower frequencies for most systematic reactions than local reactions. The incidence of solicited local AEs was comparable after the first and second vaccination and slightly increased after the booster dose (Figure 3A), the most common post-booster solicited local AE being pain at the injection site (60%–71%). The incidence of solicited systemic AEs was similar after each vaccination (Figure 3B), with the most common post-booster solicited systemic AE being fatigue (11%–33%). The safety profile observed in the primary 2-dose vaccination series and the booster phase was similar.

#### Phase II primary 2-dose series.

There were no vaccine-related SAEs. Both local and systemic AEs were mild and transient, and were self-limited in a few days.

**Figure 4:**
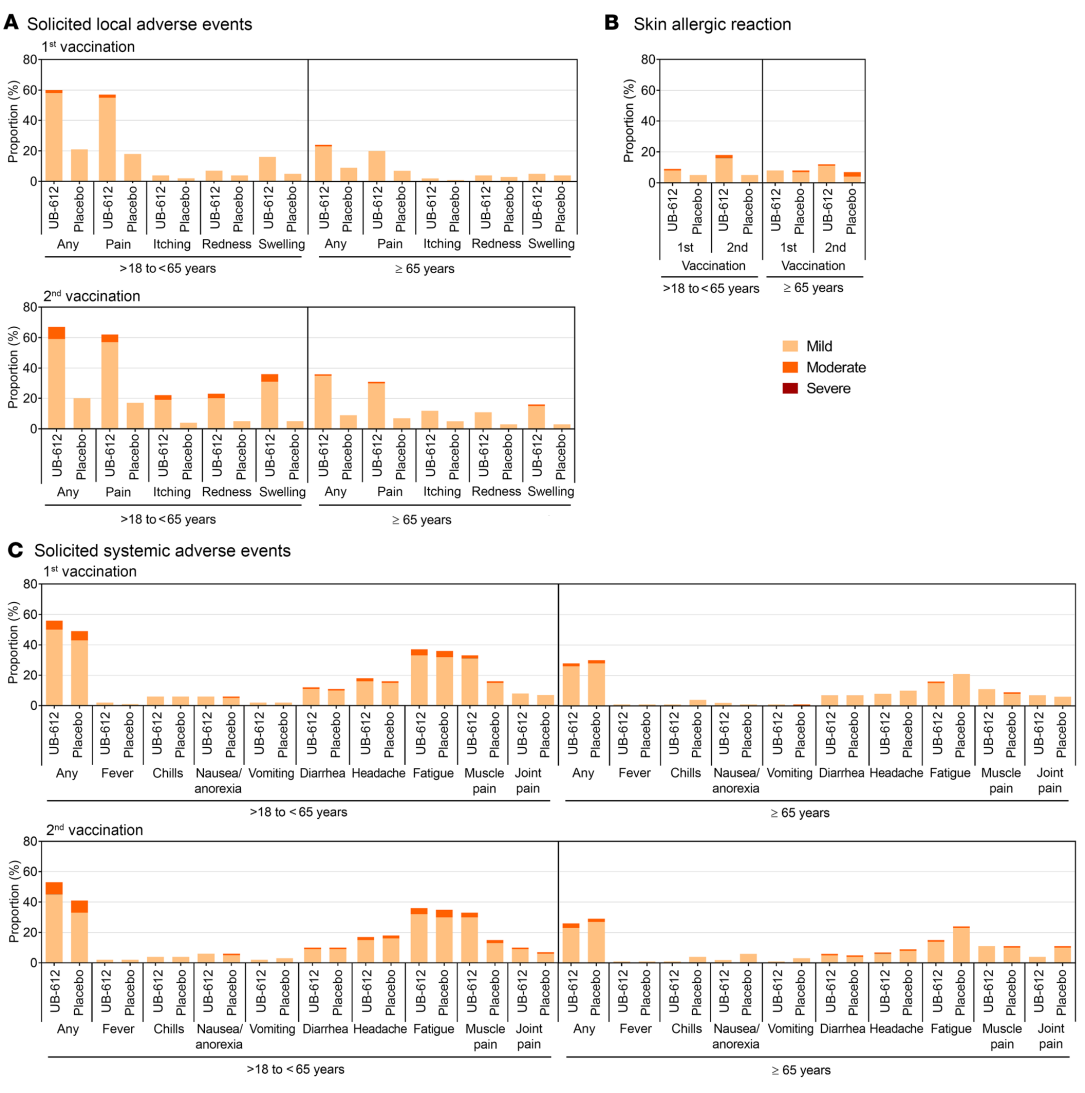
Incidence of adverse effects in the phase II interim data analysis. (**A**) Solicited local adverse reaction within 7 days after each vaccination. (**B**) Skin allergic reaction within 14 days after each vaccination. (**C**) Solicited systemic adverse reaction events 7 days after each vaccination.

Overall, 2,546 participants reported solicited local AEs, of which 2,386 (72.0%) were from UB-612 and 160 (28.9%) from the placebo group after 1 and 2 doses (Figure 4A). These local AEs were mild (grade 1) to moderate (grade 2) in severity, and the most common event was injection-site pain in 2,246 (67.8%) participants of the vaccine group, and occasional skin allergic reaction (Figure 4B).

There was no significant difference in the incidence of solicited systemic AEs between UB-612 vaccine and placebo groups across age strata (*P* > 0.05) (Figure 4C). Solicited systemic AEs were reported by 38.6% of the elderly participants (65–85 years old) among the vaccine groups, compared with 63.3% of the overall safety population. The most common solicited systemic AE was fatigue/tiredness reported in 1,488 (44.9%) of UB-612–treated participants and was generally mild.

### Neutralizing antibodies against live SARS-CoV-2 WT versus Delta, and against pseudo-SARS-CoV-2 WT versus Alpha, Beta, Gamma, and Omicron VoCs

#### Phase I primary 2-dose and booster third-dose series.

**Figure 5:**
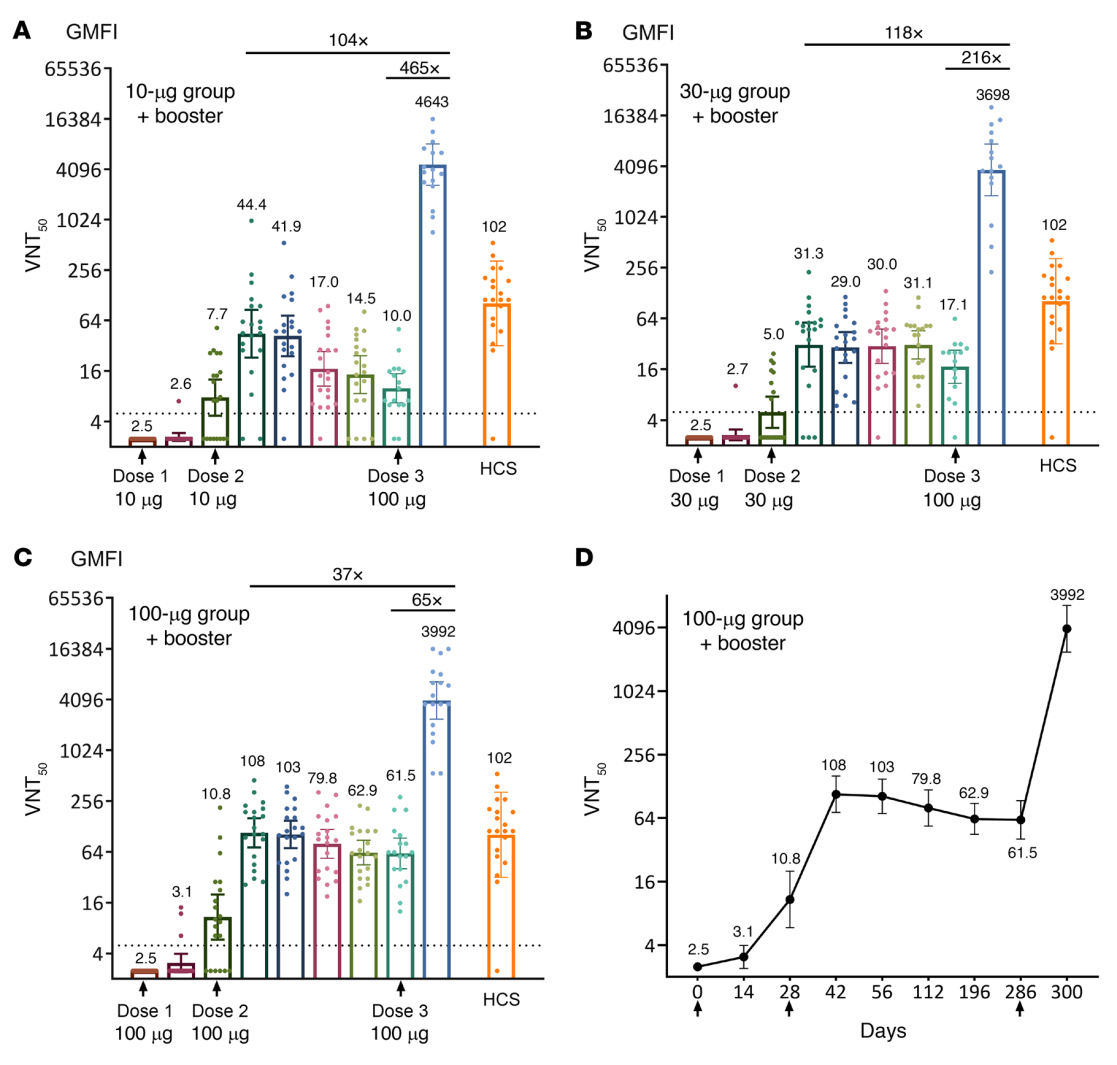
In the phase II trial, virus-neutralizing titer (VNT_50_) against live SARS-CoV-2 WT after the primary 2-dose vaccination and the booster third dose. In the primary 2-dose vaccination series of the 196-day phase I UB-612 trial, 60 participants were enrolled for the 10 μg, 30 μg, and 100 μg dose groups (*n =* 20 per group), of which 50 participants were enrolled for the extension study and received a booster third dose at 100 μg (*n =* 17 for the 10 μg; *n =* 15 for the 30 μg, and *n =* 18 for the 100 μg dose group). The virus-neutralizing antibody geometric mean titers (GMT, 95% CI) that inhibit 50% of live SARS-CoV-2 WT were measured and expressed as VNT_50_ for the (**A**) 10 μg, (**B**) 30 μg, and (**C**) 100 μg dose groups. (**D**) Illustrated with the 100 μg dose group, the VNT_50_ data were recorded on day 0 (before dose 1), day 14 (14 days after dose 1), day 28 (1 month after dose 1, before dose 2), day 42 (14 days after dose 2), day 56 (1 month after dose 2), day 112 (3 months after dose 2), day 196 (6 months after dose 2), days 255 to 316 before dose 3, the pre-booster, average day 286), and days 269 to 330 (14 days after booster, average day 300) for study participants of the 3 dose groups. The international unit (IU/mL) corresponding to 50% neutralizing GMT and 95% CI (VNT_50_) is shown in Supplemental Figure 3. The titers for individual participants are shown by the circles. The horizontal dotted lines indicate the lower limit of quantification. HCS, human convalescent serum samples in the control group (*n =* 20).

A booster dose of 100 μg given 7.6–9.6 months after the second dose induced robust neutralizing antibodies against live SARS-CoV-2 WT and Delta VoC in 100% of the participants (Figure 5). In the 10, 30, and 100 μg UB-612 dose groups, the booster elicited VNT_50_ against WT of 4,643, 3,698, and 3,992, respectively (Figure 5, A–D, and Supplemental Table 1), representing (a) 104-, 118-, and 37-fold respective increases (geometric mean fold increases, GMFIs) over the peak responses in the primary series (14 days after dose 2, i.e., day 42), and (b) GMFIs of 465, 216, and 65, respectively, over the pre-boost levels. Compared with a panel of human convalescent sera (HCS) collected approximately 1 month after onset in hospitalized COVID-19 cases, the post-booster neutralizing antibody levels were 45.5-, 36.2-, and 39.1-fold (GMFIs) higher. Neutralizing antibody titers in the same live virus test standardized with the WHO reference antiserum and expressed in international units (IU/mL) were similar (Supplemental Figure 3, A–D).

**Figure 6:**
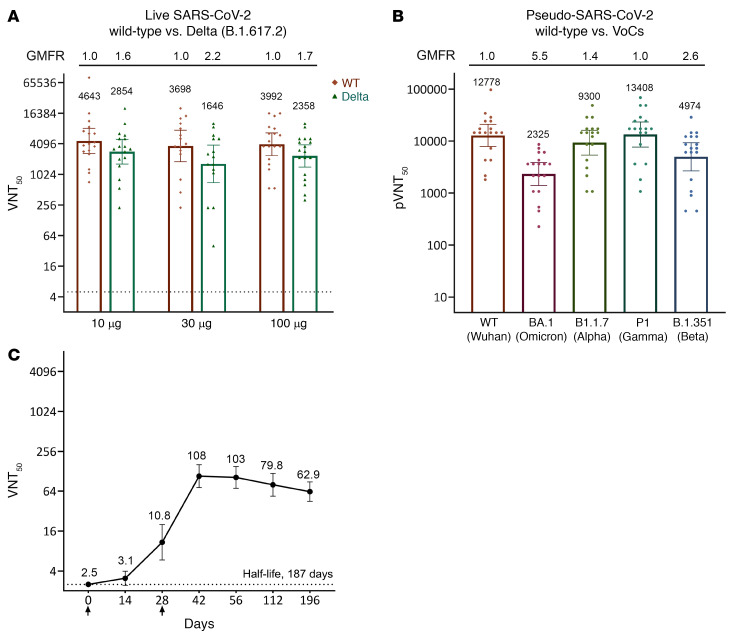
In the phase I trial, UB-612 booster third-dose produced potent neutralizing titers against SARS-CoV-2 WT, Delta, Omicron, and other VoCs, and the virus-neutralizing antibodies were long-lasting as revealed with the live WT virus. The primary 2-dose series (days 0 and 28) of the 196-day phase I trial and the extended booster third dose of 100 μg administered on mean day 286 (days 255–316). (**A**) In the participants of the 100 μg group, the VNT_50_ observed 14 days after booster reached 3,992 against live SARS-CoV-2 WT and 2,358 against live Delta. Similar high anti-WT and anti-Delta VNT_50_ levels were observed for the lower 30 and 10 μg dose groups. (**B**) In the participants of the 100 μg group, the pVNT_50_ observed 14 days after booster against pseudo-SARS-CoV-2 WT and against pseudo-SARS-CoV-2 variants, including Omicron. (**C**) Antibody persistence after 2 doses (phase I trial): The anti-WT neutralizing VNT_50_ decayed slowly, with a *t_1/2_* of 187 days, based on the first-order exponential model fitting (SigmaPlot) over days 42–196 (*r*^2^ = 0.9877; the decay rate constant *K_el_* = –0.0037; *t_1/2_* = 0.693/*K_el_*).

The booster dose induced remarkably high VNT_50_ against the live Delta VoC as well, reaching 2,854, 1,646, and 2,358 (Figure 6A), which represent modest GMFRs of 1.6, 2.4, and 1.7 (i.e., a preservation of ~63%, ~42%, and ~60% neutralizing strength, respectively) for the 10, 30, and 100 μg groups, respectively, relative to the WT strain.

The pVNT_50_ observed 14 days after booster of the 100 μg group (*n =* 18) were assessed for their cross-reactive neutralizing antibody titers against pseudo-SARS-CoV-2 and VoCs, including Omicron, as shown in Figure 6B. The pVNT_50_ against WT, Omicron, Alpha, Gamma, and Beta were 12,778, 2,325, 9,300, 13,408, and 4,974, respectively, when compared with the WT (14,171), with modest respective GMFRs of 5.5, 1.4, 1.0, and 2.6 (i.e., a preservation of 18.2%, 72.7%, 105%, and 38.9% neutralizing strength, respectively) relative to the WT strain.

The neutralizing antibodies in the primary series were long-lasting for the 100 μg group, associated with the highest increase in VNT_50_ against WT observed at 14 to 28 days after dose 2, as compared with the lower-dose 10 and 30 μg groups (Figure 5, A–C). The peak neutralizing antibody geometric mean titer (GMT; 108 on day 42, 103 on day 56) (Figure 5C) in the 100 μg group was close to the GMT of 102 for the panel of control HCS. Seroconversion rate based on the SARS-CoV-2 neutralizing antibody titers on day 57 in phase I was 100% for the 100 μg dose and remained 100% thereafter throughout the period monitored (Supplemental Table 2).

Prior to boosting (days 255–316), none of the 18 participants (0%) in the 100 μg group with VNT_50_ fell below the assay lower limit of quantification, suggesting that the induced neutralizing effect could persist for a long period of time. Antibody persistence after 2 doses for the 100 μg group from the phase I trial was calculated using first-order exponential model fitting (SigmaPlot) for the anti-WT neutralizing VNT_50_ over days 42 to 196 (*r*^2^ = 0.9877, the decay rate constant *K_el_* = –0.0037; *t_1/2_* = 0.693/*K_el_*). The neutralizing antibody VNT_50_ GMT slowly declined, with a *t_1/2_* of 187 days (Figure 6C).

We also investigated the neutralizing effects against Delta and other VoCs during the phase I primary vaccination phase with all serum samples (*n =* 20) from the primary series of phase I trial of the 100 μg UB-612 dose group (Supplemental Figure 4). The results showed preserved virus-neutralizing activities, in particular against the Delta B.1.617.2 variant, to which a 63% neutralizing activity (GMFR of 1.6) was retained relative to the WT strain. Significant neutralizing antibodies were preserved as well against the Alpha (B.1.1.7) variant, with 91% retained (GMFR of 1.1), and Gamma (P.1) variant with 56% retained (GMFR of 1.8), while that against Beta B.1.351 was weaker, with 20% retained (GMFR of 5.1).

#### Phase II primary 2-dose.

On day 57 (4 weeks after the second dose), across participants of all ages (18 to 85 years), the anti–S1-RBD titer with a GMT of 518.8 (Supplemental Figure 5A) and the virus-neutralization titer against the WT strain was age dependent, with an overall VNT_50_ of 87.2 (Supplemental Figure 5B). The younger adults (18–65 years old) had a higher VNT_50_ of 96.4, which is reproducibly close to that observed in phase I study participants 20–55 years old (VNT_50_ of 103) (Figure 5C), while the elderly adults (≥65 years old) exhibited a lower VNT_50_ of 51.6. An extension study of the phase II trial with a booster third dose is being investigated. Seroconversion rate based on the WT SARS-CoV-2 neutralizing antibody titers on day 57 (or day 56 after dose 1) across participants of all ages (18–85 years old) in phase II were from 88.6% for the elderly to 96.4% for the young adults (Supplemental Table 3).

**Figure 7:**
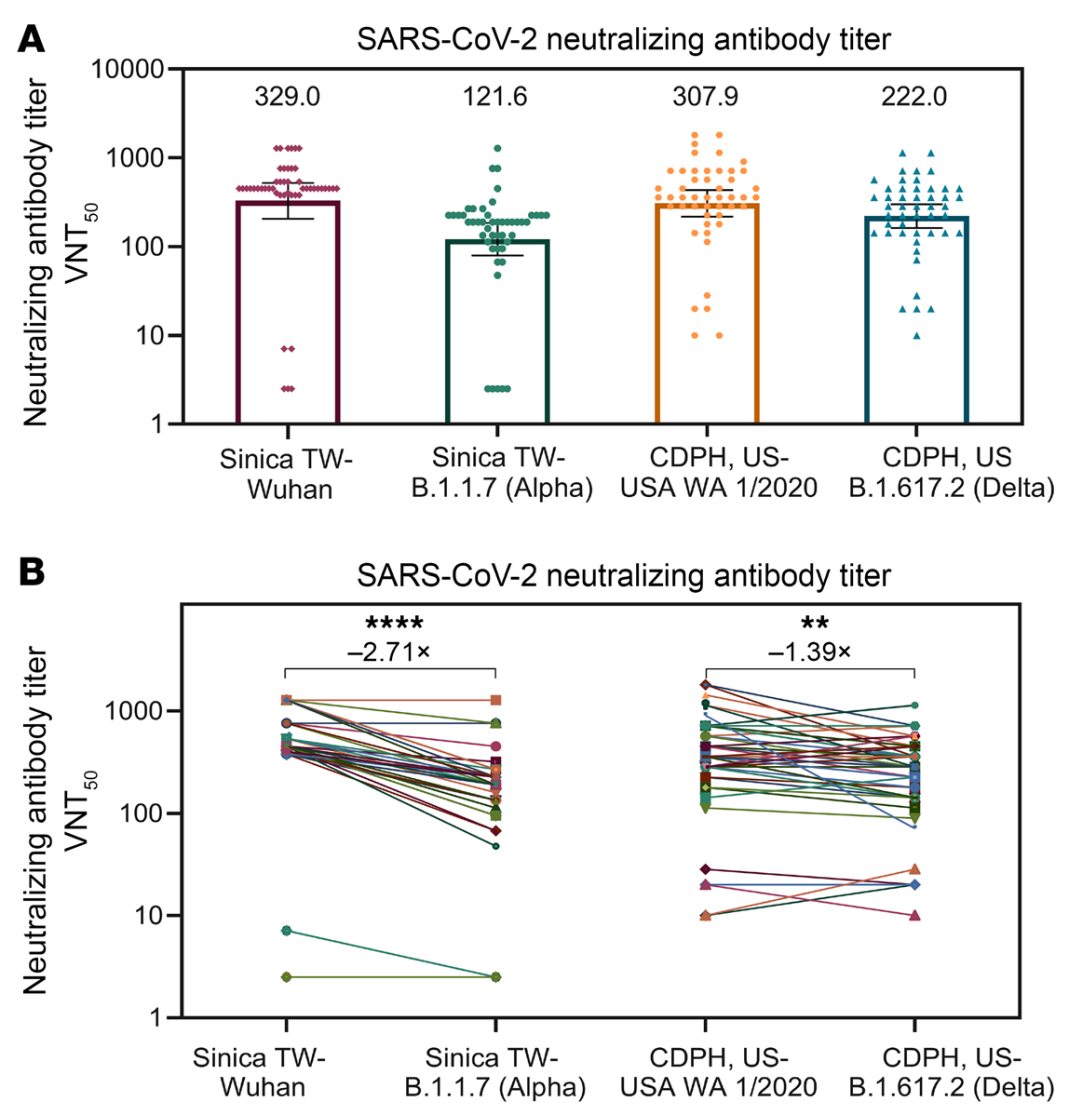
In the phase II trial primary 2-dose series, neutralizing antibody titers (VNT_50_) against SARS-CoV-2 variants. (**A**) Measurement of VNT_50_ against live SARS-CoV-2 virus variants in day 57 immune sera randomly selected from 48 vaccinees (*n =* 39 for young adults 18–65 years old; *n =* 9 for elderly adults ≥65 years old) who received 2 UB-612 vaccine doses in the phase II trial. Live WT SARS-CoV-2-TCDC#4 and USA WA1/2020, and 2 VoCs (B.1.1.7 and B.1.617.2 lineages) listed by WHO, were employed for CPE assays. The VNT_50_ values are marked on top of each column, with 95% CIs shown as horizontal bars. (**B**) The fold change (reduction) in VNT_50_ against each of the variants compared with WT strains Wuhan and USA WA1/2020 by the 2-sample *t* test. **P < 0.01; ****P < 0.0001. The 2.7- and 1.4-fold reductions also indicate 37% and 72% preservation of neutralization titers relative to the 2 WT strains isolated from 2 separate geographic locations where CPE assays were performed. Sinica, Academia Sinica, Taiwan; CDPH, California Department of Public Health.

On day 57, a substantial level of anti-Delta neutralizing antibodies was observed. A pool of 48 serum samples randomly selected from vaccinees across age groups (*n =* 39 for young adults 18–65 years old; *n =* 9 for elderly adults ≥65 years old) were subjected to an ad hoc live virus assay analysis in 2 independent laboratories (Academia Sinica and the California Department of Viral and Rickettsial Diseases). The results were concordant and revealed that immune sera could neutralize 2 key SARS-CoV-2 prototypes with a similar VNT_50_: 329 against WT obtained in Taiwan and 308 against the USA WA1/2020 strain in the United States (Figure 7). The VNT_50_ against Alpha B.1.1.7 and Delta B.1617.2 were estimated to be 122 and 222, respectively, representing a 2.7-fold and 1.4-fold reduction, relative to the USA WA1/2020 variant.

### Neutralizing antibodies against S1-RBD binding to ACE2 receptor

#### Phase I primary 2-dose and booster third-dose series.

**Figure 8:**
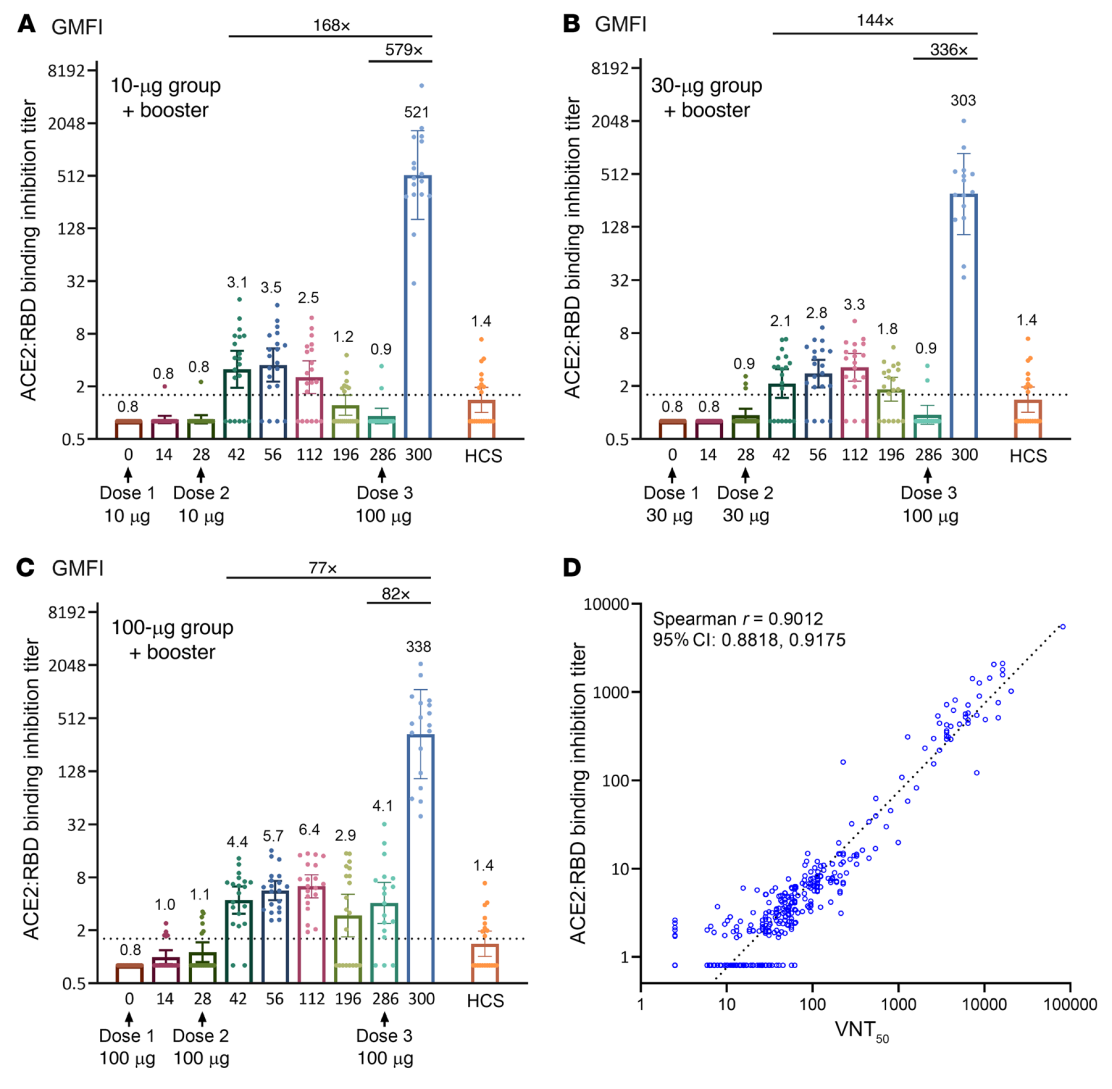
Inhibition titers against S1-RBD–ACE2 binding by ELISA in the primary 2-dose vaccination and after the booster third dose. ELISA-based neutralization (inhibition) of S1-RBD–ACE2 binding titers in the primary 2-dose vaccination series of a 196-day phase I trial (60 participants) and in the extension study with a booster third dose. Participants of (**A**) 10 μg, (**B**) 30 μg, and (**C**) 100 μg dose groups (*n =* 20 per dose group) received 2 assigned vaccine doses, 28 days apart, and a booster third dose of 100 μg at a time over 6 months administered to 50 participants (*n =* 17 for the 10 μg, *n =* 15 for the 30 μg, and *n =* 18 for the 100 μg dose groups). Serum samples were collected at the indicted time points for measuring the inhibition titers against S1-RBD binding to ACE2 by ELISA. The horizontal dotted lines indicate the lower limit of quantification. (**D**) Good correlation was found between S1-RBD–ACE2 binding inhibition and VNT_50_. Data are plotted for all prime/boost vaccinated participants (10, 30, and 100 μg dose groups). Data points for participants on day 0 were excluded from correlation analysis. Correlation analyzed by nonparametric Spearman’s correlation method.

ELISA results of the functional inhibition (neutralization) against the S1-RBD–ACE2 interaction (Figure 8) were largely consistent with the VNT_50_ data (Figure 5). The 100 μg dose group exhibited the highest neutralizing titers (Figure 8C), with an anti–S1-RBD–ACE2 quantitative neutralizing antibody (qNeuAb) level of 6.4 μg/mL on day 112, a 4.6-fold increase as compared with 1.4 μg/mL from the 20 HCS. Upon booster vaccination, the anti–S1-RBD–ACE2 qNeuAb levels reached 303 to 521 μg/mL, representing a 77- to 168-fold increase over the peaks after the primary vaccination series; similarly, profound 82- to 579-fold increases were observed as compared with the pre-boost levels (Figure 8, A–C). Thus, the UB-612 booster can elicit significant immune responses in vaccinated subjects regardless of how low their pre-boost levels are.

The neutralization of S1-RBD–ACE2 binding on ELISA correlates well with VNT_50_ findings (Spearman’s *r* = 0.9012) (Figure 8D), thus corroborating the validity of the anti-WT VNT_50_ results by the cytopathic effect (CPE) assay (Figure 5, A–C). Furthermore, the post-booster anti–S1-RBD–ACE2 qNeuAb levels of 303 to 521 μg/mL (Figure 8, A–C) were 216- to 372-fold higher than for HCS. This suggests that the majority of antibodies in HCS appear to bind more to the allosteric sites (N- or C-terminal domain of S1) than to the orthosteric (RBD) sites where viral S1-RBD interacts with the ACE2 receptor.

#### S1-RBD IgG antibody ELISA responses.

In the phase I trial, S1-RBD–binding antibodies measured by ELISA (Supplemental Figure 6) showed again that the 100 μg–vaccinated group elicited the highest immune responses over the 196-day primary series, with GMT of 2,240 on day 42, which far exceeded the GMT of 141 from the 20 HCS. Upon booster vaccination, the anti–S1-RBD GMT in the 3 dose groups peaked at 7,154 to 9,863 (3- to 28-fold increases [GMFIs] over the peaks during the primary series); similarly, profound 37- to 378-fold increases were observed as compared with the pre-boost levels. The S1-RBD ELISA results correlated well with the VNT_50_ findings (Spearman’s *r* = 0.9073). A good correlation existed also between the anti–S1-RBD antibody titers and the WHO International Reference–based Binding Antibodies Unit (BAU/mL), with similar boosting patterns (Supplemental Figure 7). In the phase II study, the anti–S1-RBD antibody level in younger adults (18–65 years old) was higher (GMT 572) than for the elderly (65–85 years old) on day 57 (GMT 312) (Supplemental Figure 5A).

### T cell responses by ELISpot

#### Phase I trial.

**Figure 9:**
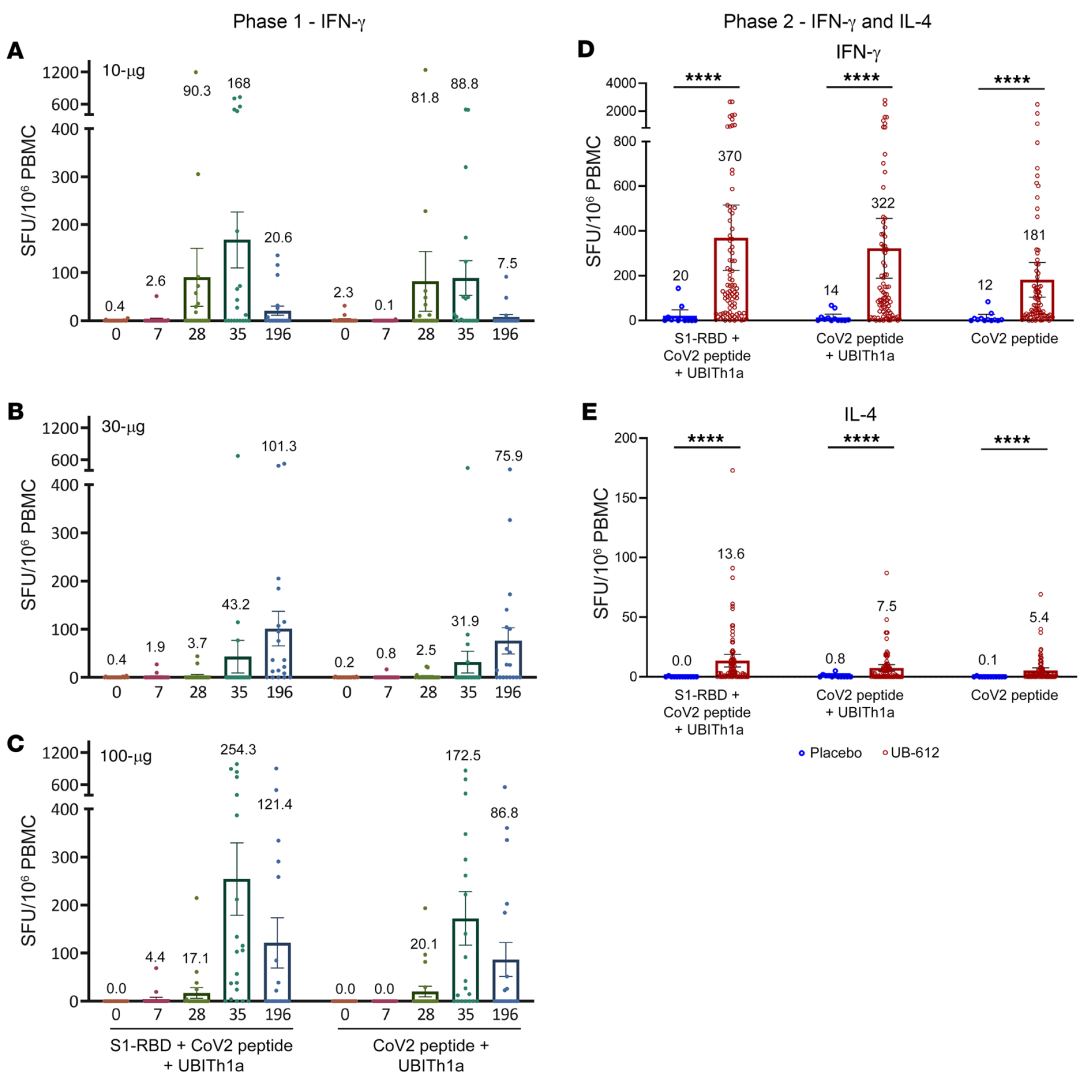
UB-612–induced long-lasting, robust Th1-predominant cell response measured by IFN-γ and IL-4 ELISpot after restimulation of PBMCs with designer peptide antigens. In the 196-day phase I trial with 2 UB-612 doses on days 0 and 28, vaccine-induced T cell responses were measured by IFN-γ ELISpot with PBMCs from young adults (20–55 years old) in (**A**) 10, (**B**) 30, or (**C**) 100 μg dose group (*n =* 20 each). In the phase II trial study, participants (younger adults, 18–65 years old) received 2 doses of UB-612 at 100 μg (*n =* 88) or saline placebo (*n =* 12), and T cell responses in PBMCs of vaccinees on day 57 restimulated with designer antigen protein/peptides were measured by (**D**) IFN-γ and (**E**) IL-4 ELISpot. Shown are spot-forming units (SFU) per 1 × 10^6^ PBMCs producing IFN-γ and IL-4 after stimulation with S1-RBD plus Th/CTL peptide pool, Th/CTL peptide pool, or SARS-CoV-2 T peptides (Th/CTL peptide pool without UBITh1a). Statistical analysis was performed with the use of the 2-sample *t* test. ****P < 0.0001.

In the primary vaccination series of the phase I trial, peripheral blood mononuclear cells (PBMCs) were collected from vaccinees, with aliquots of 250,000 PBMCs plated into each well and stimulated with 10 μg/mL (each stimulator) for evaluation by interferon-γ^+^ (IFN-γ^+^) ELISpot (Figure 9, A–C). The highest antigen-specific responses were observed in the 100 μg dose group: on day 35, 254 spot-forming units (SFU)/10^6^ PBMCs after stimulation with S1-RBD plus Th/CTL peptide pool and 173 by Th/CTL peptide pool alone (Figure 9C), demonstrating that the Th/CTL peptides in the UB-612 vaccine were principally responsible for the T cell responses.

On day 196, the IFN-γ^+^ ELISpot responses for the 100 μg dose group remained at approximately 50% of the peak responses, which decreased from 254 to 121 SFU/10^6^ cells with RBD plus Th/CTL peptide pool restimulation, or from 173 to 86.8 with Th/CTL peptide pool restimulation only. This observation suggests that the UB-612 vaccine–elicited T cell responses after 2 vaccine doses persisted for at least 6 months. This is in concert with the persistence of neutralizing antibodies noted earlier (Figure 5C).

#### Phase II trial.

In the phase II trial, the day 57 strong IFN-γ^+^ ELISpot responses were also observed: geometric mean of 370 (SFU/10^6^ cells) with S1-RBD plus Th/CTL restimulation, 322 with Th/CTL restimulation, and 181 with Th/CTL peptide pool without UBITh1a (Figure 9D), which were all far higher than the counterparts in the placebo group (*P <* 0.0001). In contrast with IFN-γ, the IL-4 responses were far lower: 13.6, 7.5, and 5.4, respectively (Figure 9E). The overall ELISpot results indicate that the inclusion of the Th/CTL peptides is essential and principally responsible for the T cell responses, while the recombinant protein S1-RBD plays only a minor role. Importantly, the orientation of the T cell response is predominantly Th1 oriented. UBITh1a plays a catalytic role as usual to trigger the Th1 responses by the virus-specific Th/CTL peptide pool.

### CD4+ and CD8+ T cell responses by intracellular cytokine staining

#### Phase II trial.

**Figure 10:**
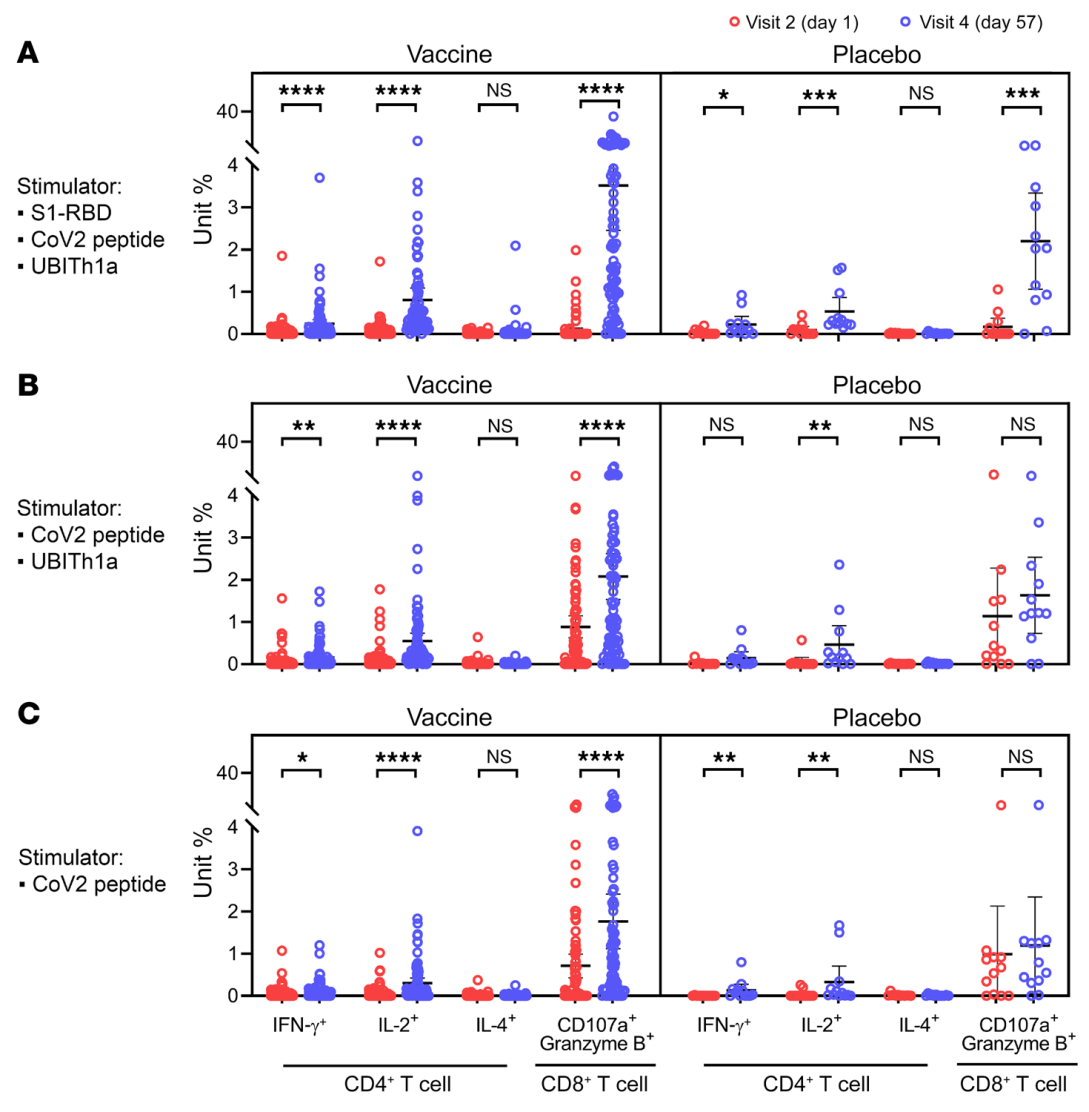
In phase II primary 2-dose vaccination series, UB-612–induced Th1-predominant T cell responses (CD4^+^ and CD8^+^) measured by IFN-γ and IL-4 ICS after restimulation of PBMCs with designer peptide antigens. In a phase II trial, study participants (younger adults 18–65 years old) receiving 2 doses (28 days apart) of UB-612 at 100 μg (*n =* 88) or saline placebo (*n =* 12). Their PBMCs harvested on days 1 and 57 (4 weeks after the second shot) were restimulated with designer antigen protein/peptides to evaluate T cell responses by intracellular cytokine staining (ICS). Frequencies of CD4^+^ and CD8^+^ T cells that produce indicated cytokines in response to the stimulation of (**A**) S1-RBD plus Th/CTL peptide pool, (**B**) Th/CTL peptide pool, and (**C**) SARS-CoV-2 T peptides (Th/CTL peptide pool without UBITh1a). Statistical analysis was performed using the Mann-Whitney *t* test. *P < 0.05; **P < 0.01; ***P < 0.001; ****P < 0.0001. NS, not significant.

T cell responses by intracellular cytokine staining (ICS) were evaluated (Figure 10). Substantial increases in IFN-γ– and IL-2–producing CD4^+^ and CD8^+^ cells were observed across the 3 peptide-restimulation groups, and, consistent with ELISpot findings (Figure 9, D and E), lower IL-4–producing CD4^+^ T cells were detected, confirming the Th1 predominance of the T cell response.

CD8^+^ T cells expressing the cytotoxic markers CD107a and granzyme B were observed, accounting for 3.5%, 2.1%, and 1.8% of circulating CD8^+^ T cells after restimulation with S1-RBD plus Th/CTL, Th/CTL, and Th/CTL pools without UBITh1a, respectively. Overall, UB-612 elicited Th1-oriented immunity with a robust CD8^+^ CTL response, which would be favorable for clearance of the viral infection, and the restimulation results indicated that Th/CTL peptides, which include non-spike N and M structural proteins, are the principal factor responsible for the T cell immunity.

## Discussion

Most of the authorized COVID-19 vaccines use the S protein as the immunogen. The UB-612 vaccine product uses the most important functional region, the RBD of the S protein and combines it with promiscuous Th and CTL epitope peptides from the N, M, and S proteins that are highly conserved across all VoCs, including Delta and Omicron, and recognized by individuals who have recovered from prior SARS-CoV-2 infection.

UB-612 is designed to not only induce neutralizing antibodies intended to block initial virus entry into human cells, but also to induce a broad T cell immunity that could eliminate virus-infected cells and a fast post-booster recall of memory immune cells upon reinfection or revaccination, should the vaccinated immunity wane overtime. Virus-specific humoral B cell and T cell responses act synergistically to protect the host from viral infection and disease severity. In the phase I primary series, UB-612 demonstrated induction of a durable neutralizing antibody response, with a long half-life of 187 days (Figure 6C) and a sustained T cell response (Figure 9C) for adults 20 to 55 years old. This is another unique feature of the vaccine design. The long-lasting nature of humoral B and T cell immune responses of UB-612 (100 μg dose group) could be an advantage when short durability of a vaccine becomes a growing concern (12, 40, 41).

There have been reports on homologous booster vaccination by other vaccine platforms (42–47). While the post-booster neutralizing antibody titers could vary due to heterogeneity in assay methodologies and in virus sources used for assay, the magnitude of the memory immune effect against prototype virus could be demonstrated by comparing the fold increases in neutralizing antibody titers of the peak responses after primary (and before boost) versus the booster vaccination series.

COVID-19 vaccines from different construct platforms were compared for the booster effect against SARS-CoV-2 WT (Table 1). The VNT_50_ measured 14 or 28 days after booster was shown to range from 122 to 6,039, with the associated differential fold increases (GMFIs) from 1.7 to 37 when compared with the respective “peak responses” in the primary vaccination series. The counterpart GMFIs of the “pre-boosting responses” were found to range from 10.3 to 92.9. The UB-612 vaccine in the 100 μg dose group produced an anti-WT VNT_50_ of 3,992, representing a 37- and a 65-fold increase over the peak primary response of 108 and over the pre-boosting response of 61.5, respectively (Figure 5C). While primary immunization with UB-612 elicited more modest neutralizing antibody responses than some other platforms, including mRNA, the levels of antibody achieved after boosting were very high and comparable.

Regarding the booster effect against the Delta variant (Table 1), the types of viral strain used for neutralization assays were sourced differently, from live clinical isolate, pseudo-type, or WT-based virus recombinantly engineered with a Delta spike. The post-booster VNT_50_ against the Delta variant has been reported to range from 54 to 2,358. Relative to WT, the fold reductions in the 50% neutralizing titer (GMFRs) ranged from 1.2 to 3.6. After boosting, UB-612 elicited an unusually high anti-Delta neutralizing VNT_50_ of 2,358 (Figure 6A), which preserves an approximately 60% neutralizing strength relative to the anti-WT VNT_50_ of 3,992, i.e., with a modest 1.7-fold reduction.

The UB-612’s post-booster preservation of substantial anti-Delta neutralizing activity (~60% relative to WT) is consistent with that observed in the primary series of the phase I trial, where UB-612 retained a remarkable 83% (1.2-fold reduction) based on the Delta versus WT VNT_50_ of 212 versus 255 (Supplemental Figure 4), and in the primary series of the phase II trial where UB-612 retained a 72% neutralizing effect (1.4-fold reduction) based on the Delta versus USA WA1/2020 VNT_50_ of 222 versus 308 (Figure 7). Overall, the differences in multitope antigenic composition could account for the observation that UB-612 vaccination preserves substantial neutralizing antibodies by 60% to 80% against the Delta strain.

A limited study of UB-612 sera from the phase I primary series showed a preservation of notable neutralizing antibodies in vaccinee sera against Alpha B.1.1.7 with 62% retained (1.6-fold reduction), Gamma P.1 with 42% retained (2.4-fold reduction), while that against Beta B.1.351 was weaker, with 23% retained (4.3-fold reduction) (Supplemental Figure 4). In the phase II primary series, UB-612 showed a 37% preservation (2.7-fold reduction) against Alpha B.1.1.7 (Figure 7).

The high anti-Delta neutralizing antibody titer (VNT_50_) observed 14 days after booster of the 100 μg group (*n* = 18) prompted us to assess cross-reactive neutralizing antibody titers (pVNT_50_) against pseudo-SARS-CoV-2 Omicron (BA.1 variant) and other VoCs, compared with WT pseudovirus (Figure 6B). The pVNT_50_ against WT, Omicron, Alpha, Gamma, and Beta were found to be 12,778, 2,325, 9,300, 13,408, and 4,974, respectively. When compared with the WT pVNT_50_ of 12,778, these variants have modest respective GMFRs of 5.5, 1.4, 1.0, and 2.6 (i.e., a preservation of 18.2%, 72.8%, 105%, and 38.9% neutralizing strength, respectively).

The profound post-booster neutralization effect against both live WT and live Delta variants illustrates one important design feature of UB-612, namely that the immune response is directed solely at the RBD that contains a concentration of potent neutralization epitopes. Boosting promptly recalls high levels of both virus-neutralizing antibodies (Figure 5) and those that inhibit RBD:ACE2 binding (Figure 8) .

Moreover, the fact that UB-612 induced much higher fold-increases in blocking the RBD:ACE2 binding than that by HCS (Figure 8C) suggests that most of the antibodies in HCS may bind allosterically to the viral spike (N- or C-terminal domain of S), rather than orthosterically to the RBD sites. This warrants further investigation that would include sera from reinfections and breakthrough infections from all vaccine platforms.

Because heterologous boosting (2-dose prime-boost) has been shown to be more efficient at stimulating high antibody responses and vaccine effectiveness than homologous boosting (48, 49), UB-612 may present itself as an effective booster for other vaccine platforms, particularly for adenovirus-vectored (AZD1222) and inactivated viral lysate (CoronaVac) vaccines that have shown modest homologous boosting effects (Table 1). In fact, United States regulatory agencies have taken further action in authorizing (Emergency Use Authorization) the use of prime vaccination and a single booster dose in both homologous (50) and heterologous (51) (i.e., “mix and match”) boosting. The availability of these authorized boosters is important for continued protection against COVID-19.

The Delta variant (B.1.617.2) contains at least 10 mutations in the S protein (52). Only 2 mutations (L452R and T478K) are located within the S1-RBD that would influence neutralization (53). L452R is located within an epitope for several neutralizing antibodies (54, 55), whereas T478K is unique to the Delta variant (56) and centrally located within the ACE2 binding site, affecting its binding affinity (57). This T478K mutation is structurally close to the E484K mutation that is known to facilitate antibody escape (58, 59). Relative to the S1-RBD design in UB-612 vaccine, all other full S protein–based vaccines are affected additionally by mutations in the N-terminal domain sequences, which contain additional neutralizing epitopes. Furthermore, structural plasticity at the RBD-ACE2 interface suggests that the RBD could tolerate and find many more mutations than found in current VoCs, and Omicron is likely not to be the end of the story for SARS-CoV-2 (60).

A study in Israel demonstrated that a booster with BNT162b2 could improve protection against infection and serious illness among people 60 years of age and older (61). Importantly, in a newly conducted phase III trial with a booster shot (July to September) involving more than 10,000 participants (62), BNT162b2 exhibited vaccine efficacy of 96.5% protection against infection and serious illness during the period when Delta was the prevalent strain, which well maintains the same high efficacy level observed from earlier post-primary 2-dose vaccination. This finding suggests that a booster vaccination could mitigate the impact of waning immunity that leads to breakthrough infections. This also suggests that the Omicron threat may be countered to some extent with a booster third shot of UB-612, as shown by a potent post-booster pVNT_50_ of 2,325 (Figure 6B).

Reportedly, induction of IFN-γ–secreting SARS-CoV-2–specific T cells is present in patients with mild disease (as opposed to severe disease) and has accelerated viral clearance (29). UB-612 vaccination in the phase II trial induced a robust CD8^+^ T cell response with a pronounced presence of cytotoxic CD8^+^ T cell markers, CD107a and granzyme B, 4 weeks after the second vaccination (Figure 10). These observations indicate that UB-612 elicits a balanced activation of memory B and T cell immunities (Supplemental Figure 2).

Overall, in the combined 3 clinical trials of the phase I primary series, an extended booster third-dose vaccination (63), and the phase II primary series, we have demonstrated that UB-612 vaccination (100 μg dose group) can induce substantial virus-neutralizing antibodies with a long half-life (Figure 6B) that go in parallel with a long-lasting cellular immunity (Figure 9C). As memory B and T cells are critical in secondary responses to infection, a successful vaccine must generate and maintain immunological memory (27, 28), and to mount a rapid recall of effective humoral and cellular responses upon natural exposure or vaccine boosting. UB-612 has indeed demonstrated such important vaccine design features through these clinical studies.

Of special note, the 5 precision-designed T cell epitope peptides represent the Th and CTL epitopes from sarbecovirus regions of the N, M, and S2 proteins (37). These epitope peptides are highly conserved across all VoCs, including Delta and Omicron, and are promiscuous epitopes that allow for induction in a broad population of memory recall and T cell activation and effector functions. Thus, the long-lasting and robust T cell immunity could be efficacious against all VoCs, including Omicron, in addition to a potent anti-Delta and anti-Omicron effect upon a booster third dose of UB-612. As structural M and N proteins fall beyond recognition by the currently authorized COVID-19 vaccines, the UB-612 vaccine has a good stance to fend off new VoCs such as Delta and Omicron, which warrants a large-scale field trial for assessment.

Safety is a major concern, especially for vaccines that will be given to billions of people that may require intermittent or even annual vaccinations. Adenoviral vectors and mRNA are innovative technologies that have only been used widely in the context of the COVID-19 pandemic. These vaccines are associated with local and systemic reactogenicity that may become more severe after repeat dosing. In addition, they have been associated with rare but serious AEs, including myocarditis, pericarditis, Guillain-Barré syndrome, and thrombosis-thrombocytopenia (64).

UB-612 has not yet been deployed widely enough to reveal rare AEs. Its composition (protein and peptides, with only aluminum adjuvant) suggests that it should have a good safety profile. So far, with approximately 4,000 people vaccinated, UB-612 has been shown to be very well tolerated and with acceptable reactogenicity upon repeated dosing. With the current sample size for safety, an upper bound of 0.08% is established for the 95% confidence interval for the incidence of an unobserved AE.

While UB-612 has demonstrated induction of a profound virus-neutralizing immunity against the Delta and Omicron variants, we understand the limitation of the small sample size of participants and the lack of booster data for the elderly and high-risk groups who have decreased immunity. An additional booster vaccination in our extended study of a phase II trial (ClinicalTrials.gov: NCT04773067) is ongoing to further demonstrate UB-612’s benefit in offering potent B and T cell immunity against multiple VoCs, including Delta and Omicron.

## Methods

### Trial design and oversight

#### Phase I trial of primary and booster third-dose series.

The safety and immunogenicity of the UB-612 vaccine were evaluated in an open-label phase I study, conducted at China Medical University Hospital, Taiwan (ClinicalTrials.gov: NCT04545749) and an 84-day extension study to evaluate a third booster dose (ClinicalTrials.gov: NCT04967742) (Figure 1). The primary-series 196-day phase I study enrolled 60 healthy adults 20–55 years old, who received 2 intramuscular injections (28 days apart) of escalating 10, 30, and 100 μg doses (*n =* 20/group). Over 6 months following completion of the primary series, 50 participants were enrolled in the extension study to receive a booster dose of 100 μg UB-612, with an interim analysis at 14 days and were also monitored until 84 days after booster. The booster dose of 100 μg was selected based on the favorable results of the phase I primary series.

#### Phase II trial of primary series.

The phase II, placebo-controlled, randomized, observer-blind, multicenter study (ClinicalTrials.gov: NCT04773067), conducted at 12 study sites in Taiwan, planned to enroll approximately 3,850 male or female adults 18+ to 85 years old (Figure 2) who were healthy or with stable and well-controlled comorbidity. Randomized to be treated with vaccine or placebo in a 6:1 ratio, study participants received 2 intramuscular doses of 100 μg UB-612 or saline placebo 28 days apart. The duration of the study was 365 days (29 days treatment period, 336 days follow-up). The dose of 100 μg for the phase II study was selected based on the favorable results of phase I primary series

The principal investigators at the study sites agreed to conduct the study according to the specifics of the study protocol and the principles of Good Clinical Practice (GCP), and all the authors assured accuracy and completeness of the data and analyses presented. The protocols were approved by the ethics committee at the site and all participants provided written informed consent. Full details of the trial design, inclusion and exclusion criteria, conduct, oversight, and statistical analyses are available in the study protocols in the supplemental material.

### Vaccine product and placebo

UB-612 used in the phase I and II trials is a multitope vaccine designed to activate both humoral and cellular responses (Supplemental Figure 2). For SARS-CoV-2 immunogens, UB-612 combines a CHO cell–expressed S1-RBD-sFc fusion protein (WT strain) and a mixture of synthetic Th and CTL epitope peptides, which were selected from immunodominant M, S2, and N regions known to bind to human major histocompatibility complexes (MHC) I and II. The preparation of the UB-612 vaccine product consists of compounding, filtration, mixing, and filling operations. Before addition of the subunit protein S1-RBD-sFc, the individual components of the vaccine were filtered through a 0.22-μm membrane filter, including the peptide solution (2 μg/mL), CpG1, a proprietary oligodeoxynucleotide solution (2 μg/mL), and 10× protein buffer containing 40 mM histidine, 500 mM arginine, 0.6% Tween 80, and 20% NaCl stock solution. After sequential addition of each component, the S1-RBD-sFc fusion protein and peptides were formulated with components described as above to form a protein-peptide complex and then adsorbed to aluminum phosphate (Adju-Phos) adjuvant (Croda Denmark). The last step was addition of water for injection containing 2-phenoxyethanol preservative solution to make the final drug product at 200 μg/mL. The UB-612 vaccine product was stored at 2°C to 8°C. Placebo used in the phase II trial was sterile 0.9% normal saline.

### Trial procedures and safety

#### Phase I trial of primary and booster third-dose series.

The phase I trial was initiated with a sentinel group of 6 participants to receive the low 10 μg dose, followed by the remaining 14 participants if without vaccine-related grade 3 or higher adverse reaction. The same procedure was extended for the 30 and 100 μg dose groups. Additional follow-up visits were scheduled for all participants on days 14, 28, 35, 42, 56, 112, and 196. Study participants were scheduled for visits 14 and 84 days after the booster. Electronic diaries were provided to the participants to be completed for the 7-day period after each injection to record solicited local reactions at the injection site (pain, induration/swelling, rash/redness, itch, and cellulitis) and solicited systemic reactions (17 varied constitutional symptoms). Severity was graded using a 5-level (0 to 4) scale from none to life-threatening. In addition, participants recorded their axillary temperature every evening starting on the day of the vaccination and for the 6 subsequent days. Safety endpoints included unsolicited AEs reported for up to 14 days after booster in this interim phase I extension report. Complete details for solicited reactions are provided in the study protocols in the supplemental material.

#### Phase II trial of primary series.

The primary safety endpoints of the phase II trial were to evaluate the safety and tolerability of all participants receiving study intervention from days 1 to 57 (28 days after the second dose). Vital signs were assessed before and after each injection. Participants were observed for 30 minutes after each injection for changes in vital signs or any acute anaphylactic reactions. After each injection, participants had to record solicited local and systemic AEs in their self-evaluation electronic diary for up to 7 days while skin allergic reactions were recorded in their electronic diary for up to 14 days. Safety endpoints included unsolicited AEs reported for days 1 to 57 in this interim phase II report. Complete details for solicited reactions are provided in the study protocols in the supplemental material.

### Data sharing

The study protocols are provided in the supplemental material. Individual participant data will be made available when the trial is complete, with data to be shared through a secure online platform.

### Statistics

As the phase I and its extension studies were not powered for formal statistical comparisons of between-dose and between-phase vaccination, we report descriptive results of safety and immunogenicity. Immunogenicity results for GMT are presented with the associated 95% confidence intervals. Statistical analyses were performed using SAS version 9.4 (SAS Institute) or Wilcoxon’s signed-rank test. Spearman’s correlation was used to evaluate the monotonic relationship between non-normally distributed data sets. The experiments were not randomized and the investigators were not blinded to allocation during experiments and outcome assessment. For the phase II study, the sample size of our trial design meets the minimum safety requirement of 3,000 study participants in the vaccine group, as recommended by the US FDA and WHO: US FDA Emergency Use Authorization for vaccines to prevent COVID-19 (guidance for industry, https://downloads.regulations.gov/FDA-2020-D-1137-0019/attachment_1.pdf) and WHO guidelines on clinical evaluation of vaccines (regulatory expectations, https://cdn.who.int/media/docs/default-source/prequal/vaccines/who-trs-1004-web-annex-9.pdf?sfvrsn=9c8f4704_2&download=true).

Safety data of solicited AEs and are presented as stacked bar charts showing the proportions of participants in each group according to the type and severity of AEs. The seroconversion rate for both the neutralization and anti–S1-RBD IgG ELISA was defined as the proportion of participants with a 4-fold or higher increase in titers from baseline. Participants from different study sites were pooled for statistical analysis. An independent data monitoring committee (IDMC) was established to monitor data safety and trial conduct. An interim analysis was triggered because the following conditions were met: all participants had completed the second dose of study intervention by 1 month (28 days), and half of participants had completed the second dose of study intervention by 2 months.

Additional methods related to immunogenicity assessment of B and T cell immunity including immunogenicity, virus-neutralizing antibody titers against SARS-CoV-2 WT and VoCs, neutralizing antibody titers against WT Wuhan-HU-1 and VoCs (Omicron, Alpha, Beta, and Gamma) by pseudovirus luciferase assay, inhibition of S1-RBD binding to ACE2 by ELISA, anti–S1-RBD binding IgG antibody by ELISA, and T cell responses by ELISpot and ICS are provided in the supplemental methods.

### Study approval

Phase I, phase II, and phase I extension studies were approved by Taiwan Food and Drug Administration (TFDA) and the Committee of Institutional Review Board (IRB) from all clinical trial sites in Taiwan with approved letters included in the supplemental appendices. The participants from the phase I and II trials received 2 doses of UB-612 vaccine and participants from the phase I extension study received a homologous booster dose at least 9.5 months after the second dose of UB-612. Phase I, phase II, and phase I extension studies were initiated in September 2020, February 2021, and August 2021, respectively. All participants enrolled in these observational studies signed their respective informed consent forms. None of the participants experienced SAEs after vaccination.

## Author contributions

CYW, FL, SD, and WJP conceptualized and designed the vaccine. SD, ZL, HKK, WJP, and HTW were responsible for vaccine manufacturing and quality control. CYW, HKK, WJP, FG, and TPM were responsible for vaccine development. YHS, CTC, JHH, KPH, HL, CYW, HKK, TK, DGH, and TPM contributed to the protocol design of the study. KPH, YHS, CTH, YJL, MCL, YCY, PLL, HCT, CH Lee, ZYS, CEL, CH Liao, FYC, HC Cheng, FDW, HTW, HL, JHH, and HC Chiu contributed to the implementation of the clinical studies, and acquired and interpreted the clinical data. CTC conducted the statistical analysis. HKK, KLH, JC, MSW, YTY, MHJ, HY Shih, HY SHen, YRL, PYC, YLL, JJL, CCL, YCC, MKM, and CVH were responsible for assay development and validation, laboratory testing and data collection, and preparation of respective reports. CYW, YHS, HKK, HL, WJP, HJY, YHH, MH, and BSK had full access to and verified all the data in the study and take responsibility for the integrity and accuracy of the data analysis. BSK and CYW drafted, edited, and prepared the manuscript. CCK provided manuscript revision and scientific comments. All authors reviewed and approved the final version of the manuscript. CYW had final responsibility for the decision to submit for publication.

## Supplementary Material

Supplemental data

Supplemental appendices

ICMJE disclosure forms

## Figures and Tables

**Table 1:**
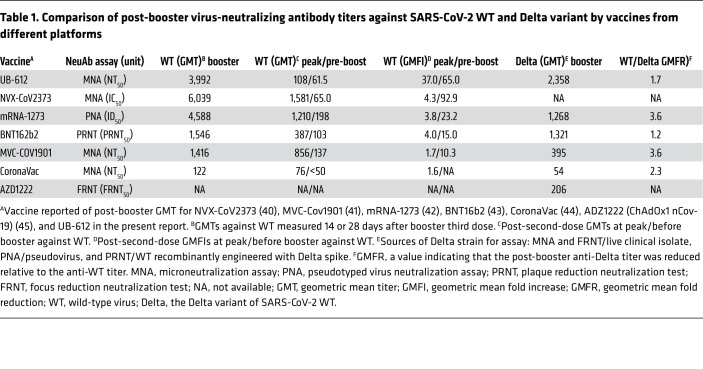
Comparison of post-booster virus-neutralizing antibody titers against SARS-CoV-2 WT and Delta variant by vaccines from different platforms

## Version 1 03/22/2022

In-Press Preview

## Version 2 05/16/2022

Electronic publication
