# Supplementary material for: A multitope SARS-CoV-2 vaccine provides long-lasting B cell and T cell immunity against Delta and Omicron variants
Source: J Clin Invest. 2022 May 16;132(10):e157707. doi: 10.1172/JCI157707 (PMC9106357; doi:10.1172/JCI157707)
Supplement: Supplemental data [file jci-132-157707-s105.pdf]

SUPPLEMENTAL MATERIAL

A Multitope SARS-COV-2 Vaccine Provides Long-Lasting B- cell and T- cell Immunity against Delta and Omicron Variants

[1,2,3,*]Chang Yi Wang, [4,5]Kao-Pin Hwang, [2]Hui-Kai Kuo, [1,2]Wen-Jiun Peng, [6]Yea-Huei Shen, [1,2,3]Be-Sheng Kuo, [2]Juin-Hua Huang, [2]Hope Liu, [2]Yu-Hsin Ho, [1]Feng Lin, [1]Shuang Ding, [1]Zhi Liu, [2]Huan-Ting Wu, [7]Ching-Tai Huang, [8] Yuan-Jang Lee, [8]Ming-Che Liu, [9]Yi-Ching Yang, [10,11]Po-Liang Lu, [12]Hung-Chin Tsai, [13]Chen-Hsiang Lee, [14]Zhi-Yuan Shi, [15]Chun-Eng Liu, [16]Chun-Hsing Liao, [17]Feng-Yee Chang, [17]Hsiang-Cheng Cheng, [18]Fu-Der Wang, [2]Kuo-Liang Hou, [2]Jennifer Cheng, [2]Min-Sheng Wang, [2]Ya-Ting Yang, [2]Han-Chen Chiu, [2]Ming-Han Jiang, [2]Hao-Yu Shih, [2]Hsuan-Yu Shen, [2]Po-Yen Chang, [2]Yu-Rou Lan, [6]Chi-Tian Chen, [19,20]Yi-Ling Lin, [20]Jian-Jong Liang, [20]Chun-Che Liao, [20]Yu-Chi Chou, [21]Mary Kate Morris, [21]Carl V. Hanson, [22]Farshad Guirakhoo, [22]Michael Hellerstein, [22]Hui-Jing Yu, [23]Chwan-Chuen King, [22]Mei Mei Hu, [22]Tracy Kemp, [22]Donald G. Heppner, [22]Thomas P. Monath

1. United Biomedical, Inc., Hauppauge, NY
2. UBI Asia, Hsinchu, Taiwan
3. United BioPharma, Inc., Hsinchu, Taiwan
4. Division of Infectious Diseases, China Medical University Children's Hospital, China Medical University, Taichung, Taiwan
5. School of Medicine, College of Medicine, China Medical University, Taichung, Taiwan
6. StatPlus, Inc., Taipei, Taiwan
7. Chang Gung Memorial Hospital, Linkou, Taiwan
8. Taipei Medical University Hospital, Taipei, Taiwan
9. National Cheng Kung University Hospital, Tainan, Taiwan
10. Department of Internal Medicine, Kaohsiung Medical University Hospital, Kaohsiung Taiwan
11. College of Medicine, Kaohsiung Medical University, Kaohsiung, Taiwan
12. Kaohsiung Veterans General Hospital, Kaoshiung, Taiwan
13. Chang Gung Memorial Hospital, Kaohsiung, Taiwan
14. Taichung Veterans General Hospital, Taichung, Taiwan
15. Changhua Christian Hospital, Changhua, Taiwan
16. Far Eastern Memorial Hospital, New Taipei, Taiwan
17. Tri-Service General Hospital, Taipei, Taiwan
18. Taipei Veterans General Hospital, Taipei, Taiwan
19. Institute of Biomedical Sciences, Academia Sinica, Taipei, Taiwan

20. Biomedical Translation Research Center (BioTReC), Academia Sinica, Taipei, Taiwan

21. Viral and Rickettsial Disease Laboratory, California Department of Public Health, Richmond, CA, USA

22. Vaxxinity Inc., Dallas, TX, USA

23. Institute of Epidemiology and Preventive Medicine, College of Public Health, National Taiwan University, Taipei, Taiwan

*To whom correspondence should be addressed: cywang@unitedbiopharma.com. +886-963085800 Chang Yi Wang, currently stationed in Taiwan with UBI Asia, Hsinchu, Taiwan

Key words: SARS-CoV-2, Sarbecovirus, Delta variant, UB-612, Multitope Vaccine, booster vaccination

TABLE OF CONTENTS

SUPPLEMENTARY METHODS

Immunogenicity..5
Viral-neutralizing antibody titers against SARS-CoV-2 wild-type and variants..................5
Neutralizing Antibody Titers against wild-type Wuhan-HU-1 and VoCs (Omicron, Alpha, Beta, and Gamma) by pesudovirus luciferase assay...6
Inhibition of S1-RBD binding to ACE2 by ELISA...8
Anti-S1-RBD binding IgG antibody by ELISA..8
T cell responses by ELISPOT...9
Intracellular Cytokine Staining (ICS)..9

SUPPLEMENTARY FIGURES

Figure S1. The globally-dominant Delta variant that constitutes ~95% of all infection cases.10
Figure S2. Components of the UB-612 multitope protein-peptide vaccine.......................11
Figure S3. Viral-neutralizing antibody titers (expressed in IU/mL) against live SARS-CoV-2 wild type after the 196-day phase-1 primary 2-dose vaccination and the booster third-dose..12
Figure S4. Viral-neutralizing VNT50 titers against different SARS-CoV-2 variants observed in the primary series of phase-1 trial of the 100-μg UB-612 dose group............13
Figure S5. Anti-S1-RBD IgG antibody and viral-neutralizing responses against SARS-CoV-2 wild type Wuhan strain..14
Figure S6. Anti-S1-RBD IgG binding titers on ELISA in the primary 2-dose vaccination and after the booster third-dose..15
Figure S7. Anti-S1-RBD antibody titers on ELISA expressed in Binding Antibodies Unit per milliliter (BAU/mL) in the primary 2-dose vaccination and after the booster third-dose..16

SUPPLEMENTARY TABLES

Table S1. In the phase-1 trial of UB-612 for the 10-, 30-, and 100-μg dose group, viral-neutralizing GMT titers (VNT50) against SARS-CoV-2 wild type (Wuhan strain) observed on Day 14 post-dose 2 in the primary 2-dose vaccination and on Day 14 after the booster third-dose of 100 μg..17
Table S2. Seroconversion Rate Based on the Wild Type SARS-CoV-2 Neutralizing Antibody Titres at Day 57 in Phase 1..17
Table S3. Seroconversion Rate Based on the Wild Type SARS-CoV-2 Neutralizing Antibody Titres at Day 57 in Phase 2..17

APPENDICES ……………………………………………………………………...20

Appendix 1
Phase 1 study V-122 protocol

Appendix 2
Phase 1 study V-122 IRB approval letter

Appendix 3
Phase 1 study V-122 Informed Consent Form (ICF)

Appendix 4
Phase 1 extension study V-123 protocol

Appendix 5
Phase 1 extension study V-123 IRB approval letter

Appendix 6
Phase 1 extension study V-123 Informed Consent Form (ICF)

Appendix 7
Phase 2 study V-205 protocol

Appendix 8
Phase 2 study V-205 IRB approval letter 1
Phase 2 study V-205 IRB approval letter 2
Phase 2 study V-205 IRB approval letter 3

Appendix 9
Phase 2 study V-205 Informed Consent Form (ICF)

SUPPLEMENTARY METHODS

Immunogenicity

The primary immunogenicity endpoints were the geometric mean titers (GMT) of neutralizing antibodies against SARS-CoV-2 wild-type (Wuhan strain), and the phase-1 post-booster effects against Delta variant were explored as well. Viral-neutralizing antibody titers that neutralize 50% (VNT_{50}) of live SARS-CoV-2 wild-type (WT) and Delta variant were measured by a cytopathic effect (CPE)-based assay using Vero-E6 (ATCC® CRL-1586) cells challenged with SARS-CoV-2-Taiwan-CDC#4 (Wuhan strain) and SARS-CoV-2-Taiwan-CDC#1144 (B.1.617.2; Delta variant). The replicating virus neutralization test conducted at Academia Sinica was fully validated using internal reference controls and results expressed as VNT_{50}. The WHO reference standard was also employed and results reported in international units (IU/mL).

The secondary immunogenicity endpoints include anti-S1-RBD IgG antibody, inhibitory titers against S1-RBD:ACE2 interaction, and T-cell responses assayed by ELISpot and Intracellular Staining. The RBD IgG ELISA was fully validated using internal reference controls and results expressed in end-point titers. The WHO reference standard was also employed and results reported in Binding Antibody Units (BAU/mL). A panel of 20 human convalescent serum samples from COVID-19 Taiwan hospitalized patients aged 20 to 55 years were also tested for comparison with those in the vaccinees. Human peripheral blood mononuclear cells (PBMCs) were used for monitoring T cell responses. All bioassay methods are detailed below.

Viral-neutralizing antibody titers against SARS-CoV-2 wild-type and variants. Neutralizing antibody titers were measured by CPE-based live virus neutralization assay using Vero-E6 cells challenged with wild type (SARS-CoV-2-Taiwan-CDC#4, Wuhan) and Delta variant (SARS-CoV-2-Taiwan-CDC#1144, B.1.617.2), which was conducted in a BSL-3 lab at Academia Sinica, Taiwan. Vero-E6 (ATCC® CRL-1586) cells were cultured in DMEM (Hyclone) supplemented with 10% fetal bovine serum (FBS, Gibco) and 1x Penicillin-Streptomycin solution (Thermo) in a humidified atmosphere with 5% CO_2 at 37°C. The 96-well microtiter plates are seeded with 1.5×10^4 cells/100 μL/well. Plates are incubated at 37° C in a CO_2 incubator overnight. The next day tested sera were heated at 56 °C for 30 min to inactivate complement, and then diluted in DMEM (supplemented with 2% FBS and 1x Penicillin/Streptomycin). Serial 2-fold dilutions of sera were carried

out for the dilutions. Fifty μL of diluted sera were mixed with an equal volume of virus (100 TCID50) and incubated at 37°C for 1 hr. After removing the overnight culture medium, 100 μL of the sera-virus mixtures were inoculated onto a confluent monolayer of Vero-E6 cells in 96-well plates in triplicate. After incubation for 4 days at 37 °C with 5% CO_2, the cells were fixed with 10% formaldehyde and stained with 0.5% crystal violet staining solution at room temperature for 20 min. Individual wells were scored for CPE as having a binary outcome of 'infection" or 'no infection.' Determination of SARS-CoV-2 virus specific neutralization titer was to measure the neutralizing antibody titer against SARS-CoV-2 virus based on the principle of VNT50 titer (≥50% reduction of virus-induced cytopathic effects). Virus neutralization titer of a serum was defined as the reciprocal of the highest serum dilution at which 50% reduction in cytopathic effects are observed and results are calculated by the method of Reed and Muench.

In a separate study with D614G, Alpha B.1.1.7, Gamma P.1, Beta B.1.351, and Delta B.1.617.2, the CPE assay was conducted at the Viral and Rickettsial Disease Laboratory, State of California Department of Public Health USA. Neutralizing antibody titers were measured by CPE-based live virus neutralization assay using cells challenged with SARS-CoV-2 variants. The study was conducted in a BSL3 laboratory at the Viral and Rickettsial Disease Laboratory (VRDL), California State Department of Public Health (CDPH), USA. SARS-CoV-2 virus specific neutralization against SARS-CoV-2 variants titers were measured by *in vitro* microneutralization assay using Vero-81cells challenged with SARS-CoV-2 variants. Vero-81 were cultured with MEM supplemented with 1x penicillin-streptomycin (Gibco) and glutamine (Gibco) and 5% Fetal calf serum (Hyclone). Determination of SARS-CoV-2 virus specific neutralization titer was to measure the neutralizing antibody titer against the viruses based on the principle of VNT50 titer (≥50% reduction of virus-induced cytopathic effects). Virus neutralization titer of a serum was defined as the reciprocal of the highest serum dilution at which 50% reduction in cytopathic effects.

Neutralizing Antibody Titers against wild-type Wuhan-HU-1 and VoCs (Omicron, Alpha, Beta, and Gamma) by pesudovirus luciferase assay. Neutralizing antibody titers were measured by neutralization assay using HEK-293T-ACE2 cells challenged with SARS-CoV-2 pseudovirus variants. The study was conducted in a BSL2 lab at RNAi core, Biomedical Translation Research Center (BioTReC), Academia Sinica. Human embryonic kidney (HEK-293T/17; ATCC® CRL-11268™) cells were obtained

from the American Type Culture Collection (ATCC). Cells were cultured in DMEM (Gibco) supplemented with 10% fetal bovine serum (Hyclone) and 100 U/mL of Penicillin-Streptomycin solution (Gibco), and then incubated in a humidified atmosphere with 5% CO2 at 37 °C. HEK-293T-ACE2 cells were generated by transduction of VSV-G pseudotyped lentivirus carrying human ACE2 gene. To produce SARS-CoV-2 pseudoviruses, a plasmid expressing C-terminal truncated wild-type Wuhan-Hu-1 strain SARS-CoV-2 spike protein (pcDNA3.1-nCoV-SΔ18) was co-transfected into HEK-293T/17 cells with packaging and reporter plasmids (pCMVΔ8.91, and pLAS2w.FLuc.Ppuro, respectively) (BioTReC, Academia Sinica), using TransIT-LT1 transfection reagent (Mirus Bio). Site-directed mutagenesis was used to generate the Omicron BA.1 and other VoCs by changing nucleotides from Wuhan-Hu-1 reference strain. For BA.1 variant, the mutations of spike protein are A67V, Δ69-70, T95I, G142D/Δ143-145, Δ211/L212I, ins214EPE, G339D, S371L, S373P, S375F, K417N, N440K, G446S, S477N, T478K, E484A, Q493R, G496S, Q498R, N501Y, Y505H, T547K, D614G, H655Y, N679K, P681H, N764K, D796Y, N856K, Q954H, N969K, L981F. Indicated plasmids were delivered into HEK-293T/17 cells by using TransITR-LT1 transfection reagent (Mirus Bio) to produce different SARS-CoV-2 pseudoviruses. At 72 hours post-transfection, cell debris were removed by centrifugation at 4,000 xg for 10 minutes, and supernatants were collected, filtered (0.45 μm, Pall Corporation) and frozen at −80 °C until use. HEK-293-hACE2 cells ($1x10^4$ cells/well) were seeded in 96-well white isoplates and incubated for overnight. Tested sera were heated at 56°C for 30 min to inactivate complement, and diluted in medium (DMEM supplemented with 1% FBS and 100 U/ml Penicillin/Streptomycin), and then 2-fold serial dilutions were carried out for a total of 8 dilutions. The 25 μL diluted sera were mixed with an equal volume of pseudovirus (1,000 TU) and incubated at 37 °C for 1 hr before adding to the plates with cells. After 1-hr incubation, the 50 μL mixture added to the plate with cells containing with 50 μL of DMEM culture medium per well at the indicated dilution factors. On the following 16 hours incubation, the culture medium was replaced with 50 μL of fresh medium (DMEM supplemented with 10% FBS and 100 U/ml Penicillin/Streptomycin). Cells were lysed at 72 hours post-infection and relative light units (RLU) was measured by using Bright-GloTM Luciferase Assay System (Promega). The luciferase activity was detected by Tecan i-control (Infinite 500). The percentage of inhibition was calculated as the ratio of RLU reduction in the presence of diluted serum to the RLU value of virus only control and the calculation formula was

shown below: (RLU ^{Control} - RLU ^{Serum}) / RLU ^{Control}. The 50% protective titer (NT50 titer) was determined by Reed and Muench method.

Inhibition of S1-RBD binding to ACE2 by ELISA. The 96-well ELISA plates were coated with 2 μg/mL ACE2-ECD-Fc antigen (100 μL/well in coating buffer, 0.1M sodium carbonate, pH 9.6) and incubated overnight (16 to 18 hr) at 4 °C. Plates were washed 6 times with Wash Buffer (25-fold solution of phosphate buffered saline, pH 7.0-7.4 with 0.05% Tween 20, 250 μL/well/wash) using an Automatic Microplate Washer. Extra binding sites were blocked by 200 μL/well of blocking solution (5 N HCl, Sucrose, Triton X-100, Casein, and Trizma Base). Five-fold dilutions of immune serum or a positive control (diluted in a buffered salt solution containing carrier proteins and preservatives) were mixed with a 1:100 dilution of S1-RBD-HRP conjugate (horseradish peroxidase-conjugated recombinant protein S1-RBD-His), incubated for 30±2 min at 25±2 °C, washed and TMB substrate (3,3',5,5'-tetramethylbenzidine diluted in citrate buffer containing hydrogen peroxide) is added. Reaction is stopped by stop solution (diluted sulfuric acid, H_2SO_4, solution, 1.0 M) and the absorbance of each well is read at 450nm within 10 min using the Microplate reader (VersaMax). Calibration standards for quantitation ranged from 0.16 to 2.5 μg/mL. Samples with titer value below 0.16 μg/mL were defined as being half of the detection limit. Samples with titer exceed 2.5 μg/mL were further diluted for reanalysis.

Anti-S1-RBD binding IgG antibody by ELISA. The 96-well ELISA plates were coated with 2 μg/mL recombinant S1-RBD-His protein antigen (100 μL/well in coating buffer, 0.1 M sodium carbonate, pH 9.6) and incubated overnight (16 to 18 hr) at room temperature. One hundred μL/well of serially diluted serum samples (diluted from 1:20, 1:1,000, 1:10,000 and 1:100,000, total 4 dilutions) in 2 replicates were added and plates are incubated at 37 °C for 1 hr. The plates were washed six times with 250 μL Wash Buffer (PBS-0.05% Tween 20, pH 7.4). Bound antibodies were detected with HRP-rProtein A/G at 37 °C for 30 min, followed by six washes. Finally, 100 μL/well of TMB (3,3',5,5'-tetramethylbenzidine) prepared in Substrate Working Solution (citrate buffer containing hydrogen peroxide) was added and incubated at 37 °C for 15 min in the dark, and the reaction stopped by adding 100 μL/well of H_2SO_4, 1.0 M. Sample color developed was measured on ELISA plate reader (Molecular Device, SpectraMax M2e). UBI® EIA Titer Calculation Program was used to calculate the relative titer. The anti-S1-RBD antibody

level is expressed as Log_{10} of an end point dilution for a test sample (SoftMax Pro 6.5, Quadratic fitting curve, Cut-off value 0.248).

T cell responses by ELISPOT. Human peripheral blood mononuclear cells (PBMCs) were used in the detection of the T cell response. For the primary-series 196-day Phase 1 study, antigen-specific interferon-gamma (IFN-γ) measurement to assess cellular (T cell) immune response were was performed by an ELISpot method using human IFN-γ ELISpotPLUS kit (ALP) (MABTECH). For the booster-series third-dose series extension study, ELISpot assays were performed using the human IFN-γ/IL-4 FluoroSpot^{PLUS} kit (MABTECH). Aliquots of 250,000 PBMCs were plated into each well and stimulated, respectively, with 10 μg/mL (each stimulator) of S1-RBD+Th/CTL, Th/CTL, or Th/CTL pool without UBITh1a (CoV2 peptides), and cultured in medium alone as negative controls for each plate for 24 hours at 37 °C with 5% CO_2. The analysis was conducted according to the manufacturer's instructions. Spot-forming units (SFU) per million cells was calculated by subtracting the negative control wells.

Intracellular Cytokine Staining (ICS). Intracellular cytokine staining and flow cytometry was used to evaluate $CD4^+$ and $CD8^+$ T cell responses. PBMCs were stimulated, respectively, with S1-RBD-His recombinant protein plus with Th/CTL peptide pool, Th/CTL peptide pool only, CoV2 peptides, PMA + Inonmycin (as positive controls), or cultured in culture medium alone as negative controls for 6 hours at 37°C with 5% CO_2. Following stimulation, cells were washed and stained with viability dye for 20 minutes at room temperature, followed by surface stain for 20 minutes at room temperature, cell fixation and permeabilization with the BD cytofix/cytoperm kit (Catalog # 554714) for 20 minutes at room temperature, and then intracellular stain for 20 minutes at room temperature. Intracellular cytokine staining of IFN-γ, IL-2 and IL-4 was used to evaluate $CD4^+$ T cell response. Intracellular cytokine staining of IFN-γ, IL-2, CD107a and Granzyme B was used to evaluate $CD8^+$ T cell responses. Upon completion of staining, cells were analyzed in a FACSCanto II flow cytometry (BD Biosciences) using BD FACSDiva software.

SUPPLEMENTAL FIGURES

Figure S1. Distribution of the globally-dominant SARS-CoV-2 Delta variant is being replaced by the Omicron variant

A

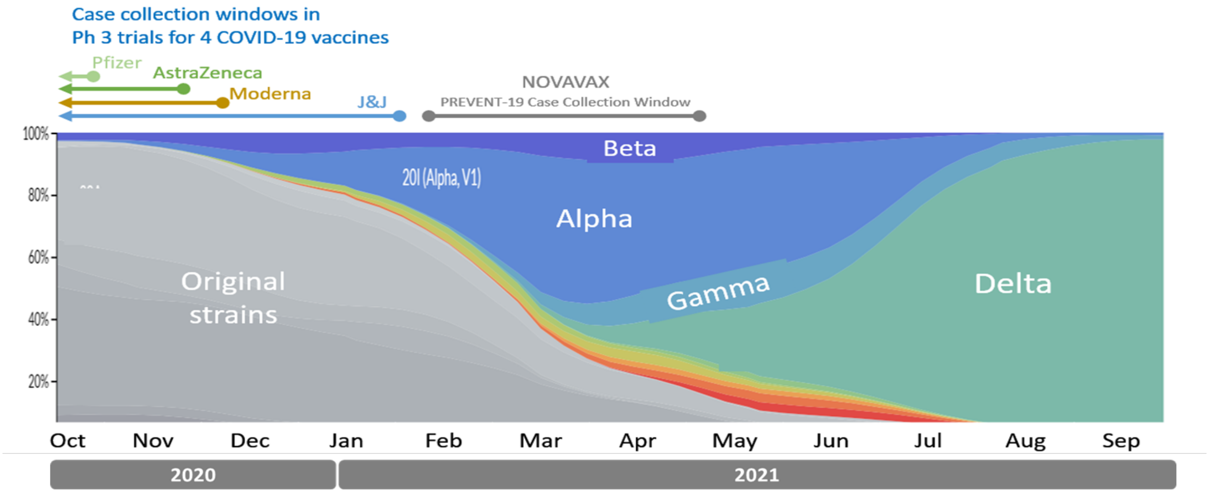

Source: Adapted from Strain distribution data from Nextstrain.org (GISAID data) as of 04-Oct-2021, with edits for vaccines EUA-authorised and (Variant of Concern).

(1) *U.S. FDA fully approved Pfizer's COVID-19 vaccine in Aug 2021 after being first made available in the U.S. under emergency use authorization in December 2020.*

(2) *Case collection cutoff dates for primary efficacy endpoint used to support EUL/conditional approvals: Moderna (25-NOV-2020; DOI: 10.1056/NEJMoa2035389), Pfizer (09-OCT-2020; DOI: 10.1056/NEJMoa2034577), AstraZeneca (04-NOV-2020; DOI: 10.1016/S0140-6736(20)32661-1), J&J (22-JAN-2021; DOI: 10.1056/NEJMoa2101544).*

(3) *Novavax case collection window for primary efficacy endpoint from 25-JAN-2021 to 30-APR-2021 (PREVENT-19 Final Data Announcement Presentation; 14-JUNE-2021)*

B

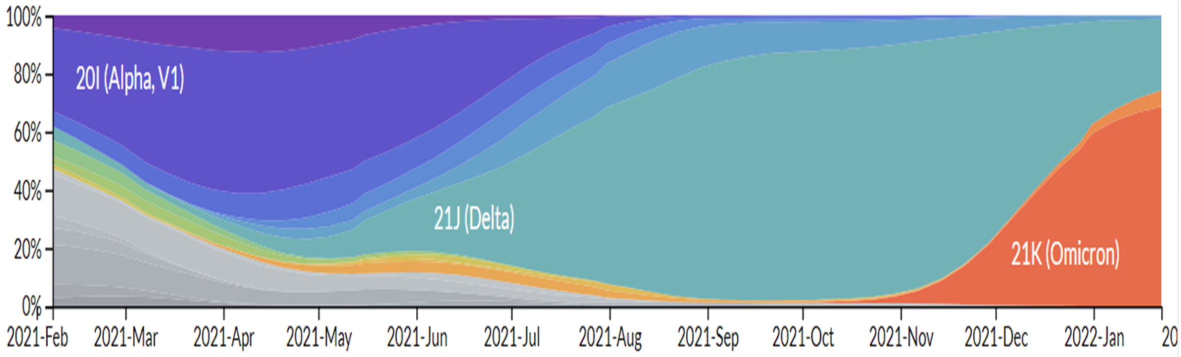

Source: Adapted from Strain Distribution data from Nextstrain.org (GISAID data) as of Jan-22-2022.

The changes overtime in %distribution of SARS-CoV-2 Variants of Concern (VoCs). **(A)** The time points when the five COVID-19 vaccines (Pfizer, Moderna, AZ, J&J and Novavax) were developed and EUA authorized; and the Delta variant that dominated with >95% of all infection cases observed in October 2021. **(B)** The once globally-dominant Delta variant in infectivity is being overtaken and replaced by the heavily-mutated Omicron strain, which constitutes ~70% of all infection cases worldwide, as of Jan. 22, 2022.

Figure S2. Components of the UB-612 multitope protein-peptide vaccine

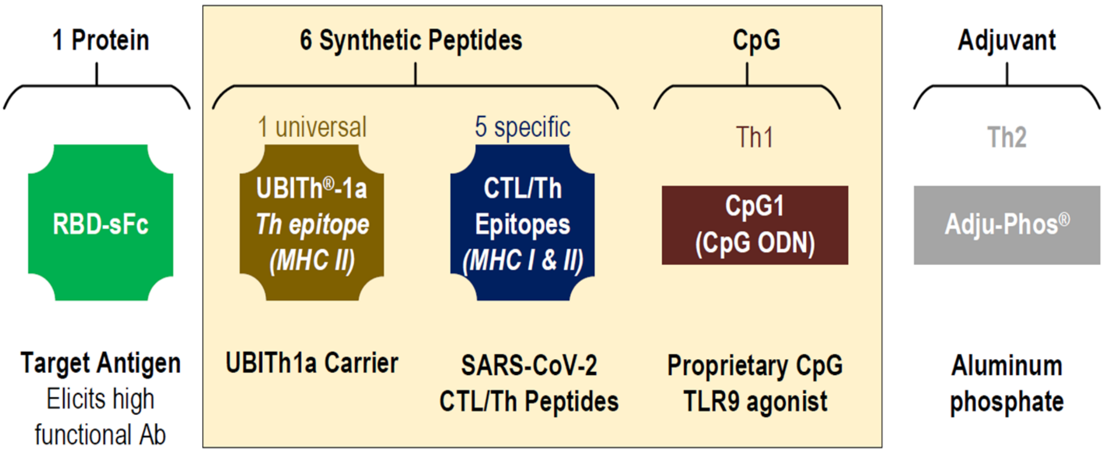

UB-612 vaccine construct contains an S1-RBD-sFc single chain fusion protein targeting only the receptor ACE2 conformational binding site for the B cell epitopes, plus five synthetic Th/CTL peptides derived from SARS-CoV-2 spike S2, membrane (M), nucleocapsid (N) proteins, and the proprietary UBITh1a peptide as a catalyst, all with promiscuous MHC class I and II binding motifs with broad HLA genetic coverage, and known to bind and trigger T cell proliferation. These positively charged designer T peptides are bound to our proprietary highly negatively charged CpG1 through charge neutralization, which is then bound to Adjuphos® as an adjuvant to constitute the UB-612 vaccine drug product.

Figure S3. Viral-neutralizing antibody titers (expressed in IU/mL) against live SARS-CoV-2 wild type after the 196-day phase-1 primary 2-dose vaccination and the booster third-dose

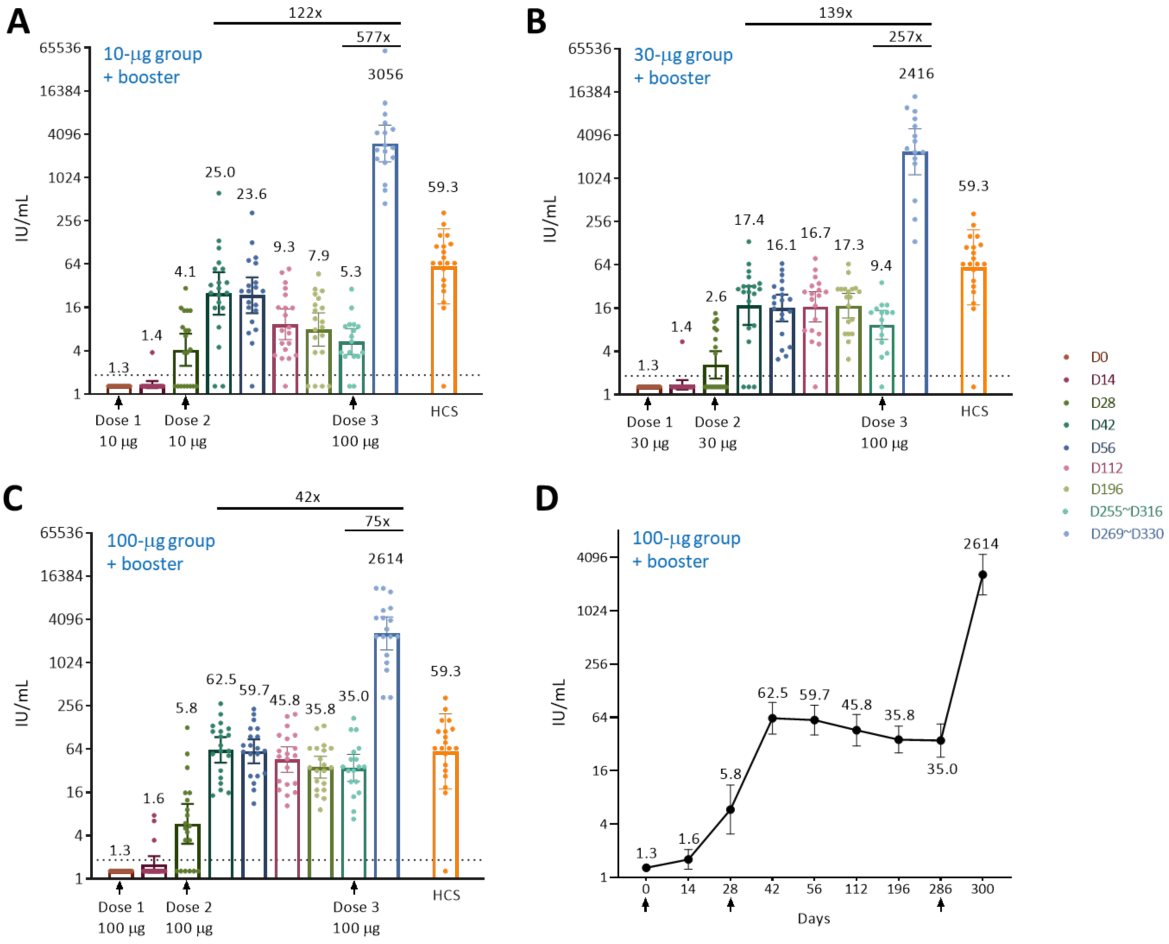

In the primary 2-dose vaccination series of the 196-day phase-1 UB-612 trial, 60 participants were enrolled for the10-µg, 30-µg, and 100-µg dose groups (n = 20 each group), of which 50 participants were enrolled for the extension study and received a booster 3rd-dose at 100 µg (n = 17 for the 10-µg; n = 15 for the 30-µg, and n = 18 for the 100-µg dose group). The viral-neutralizing antibody geometric mean titers (GMT) that inhibit 50% of live SARS-CoV-2 wild-type (WT, Wuhan strain) were measured and expressed as International Unit (IU/mL) for the **(A)** 10-µg, **(B)** 30-µg, and **(C)** 100-µg dose groups. The interchange of VNT_{50} and IU/mL is based on the lab-established relationship: International Unit (IU) = 0.4964*($VNT50^{1.0334}$). **(D)** Illustrated with the 100-µg dose group, the VNT_{50} data were recorded on Day 0 (pre-dose 1), Day 14 (14 days post-dose 1), Day 28 (1 mon. post-dose 1; pre-dose 2), Day 42 (14 days post-dose 2), Day 56 (1 mon. post-dose 2), Day 112 (3 mon. post-dose 2), Day 196 (6 mon. post-dose 2), Days 255 to 316 (pre-dose 3, the pre-booster), and Days 269 to 330 (14 days post-booster) for study participants of the three dose groups. The 50% neutralizing GMT (VNT_{50}) corresponding to International Unit (IU/mL) is shown in the Figure 5. The results are denoted on the top of the box and the 95% confidence intervals are shown by the brackets. The titers for individual participants are shown by the circles. The horizontal dotted lines indicate the lower limit of quantification (LLOQ). HCS: human convalescent serum samples in the control group (n = 20).

Figure S4. Viral-neutralizing $pVNT_{50}$ titers against different SARS-CoV-2 variants observed in the primary series of phase-1 trial of the 100-µg UB-612 dose group

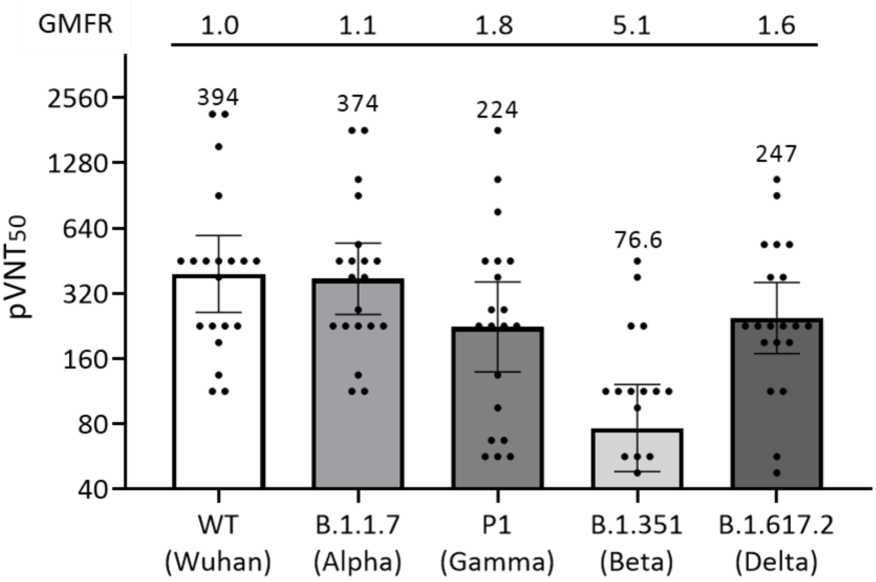

In the primary 2-dose vaccination series of phase-1 trial with vaccinees receiving two UB-612 doses at 100 µg, twenty samples (n = 20) of Day-56 immune sera (28 days after the second dose) were selected for measuring comparative neutralizing antibody activity against Variants of Concerns (VoCs). $pVNT_{50}$ titers were assessed by pseudovirus-luciferase assay (*in vitro* live virus microneutralization). The study was conducted in BSL2 lab at RNAi core facility in Sinica.

Figure S5. Anti-S1-RBD IgG antibody and viral-neutralizing responses against SARS-CoV-2 wild type Wuhan strain

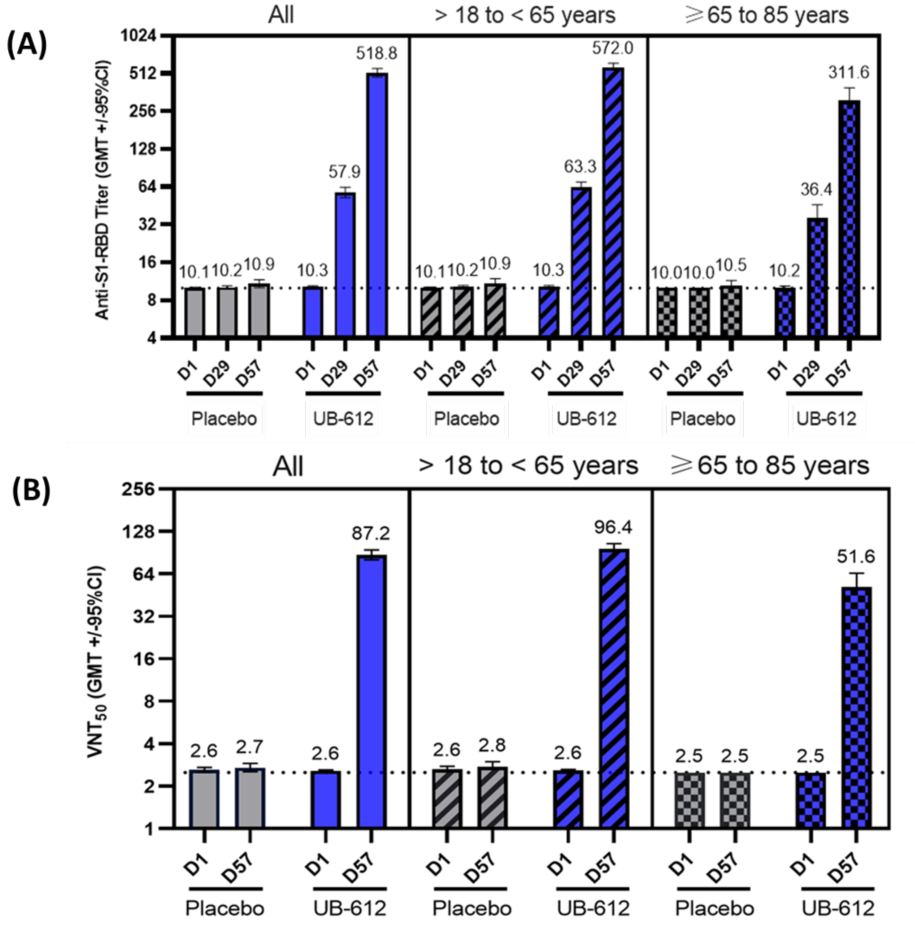

(A) In the phase-2 study of UB-612 at 100 µg, the mean ELISA-based GMTs of anti-S1-RBD IgG response at Days 1, 29 and 57 across age groups (n = 871 for All; n = 731 for 18-65 years; and n = 140 for 65-85 years). The error bars represent 95%CI, and the dashed lines denote limit of ELISA assay. **(B)** The GMTs of 50% viral-neutralizing response (VNT_{50}) against SARS-CoV-2-TCDC#4 (Wuhan wild type) virus at Days 1 and 57 across age groups were measured by the microneutralization CPE assay. The error bars represent 95%CI, and the dashed lines denote the limit of microneutralization assay. The VNT_{50} of 96.4 in younger adults aged 18-65 years was essentially reproducible as seen at Day 56 in the phase-1 trial in vaccinees (aged 20-55 years) on the 100-µg vaccine dose where the VNT_{50} was estimated to be 103 (Figure 5C).

Figure S6. Anti-S1-RBD IgG binding titers on ELISA in the primary 2-dose vaccination and after the booster third-dose

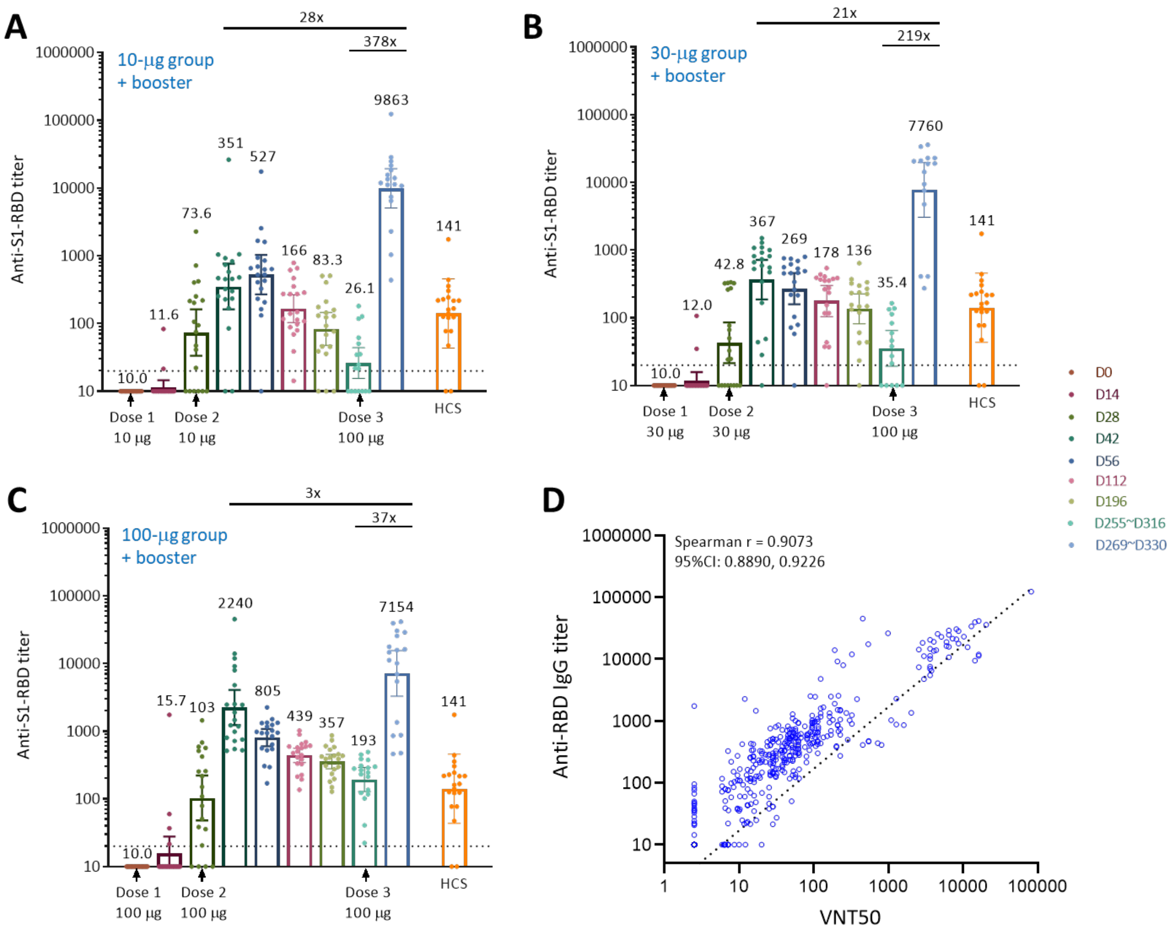

ELISA-based anti-S1-RBD antibody binding titers in the primary 2-dose vaccination series of a 196-day phase 1 trial (60 participants) and in the extension study with a booster third-dose. Participants of **(A)** 10-µg, **(B)** 30-µg, and **(C)** 100-µg dose groups (n = 20 each dose group) who had received two assigned vaccine doses, 28 days apart, and a booster third dose of 100 µg at a time over 6 months administered to 50 participants (n = 17 for the 10-µg, n = 15 for the 30-µg, and n = 18 for the 100-µg dose groups). Serum samples were collected at the indicted time points for measuring anti-S1-RBD antibody binding by ELISA, expressed as geometric mean titer GMT and 95% CI. The horizontal dotted lines indicate the lower limit of quantification (LLOQ). **(D)** Good correlation exists between anti-S1-RBD antibody binding and VNT_{50}. Data are plotted for all prime/boost vaccinated participants (10-, 30- and 100-µg dose groups). Data points for participants at day 0 are excluded from correlation analysis. Correlation analyzed by Nonparametric Spearman correlation method.

Figure S7. Anti-S1-RBD antibody titers on ELISA expressed in Binding Antibodies Unit per milliliter (BAU/mL) in the primary 2-dose vaccination and after the booster third-dose

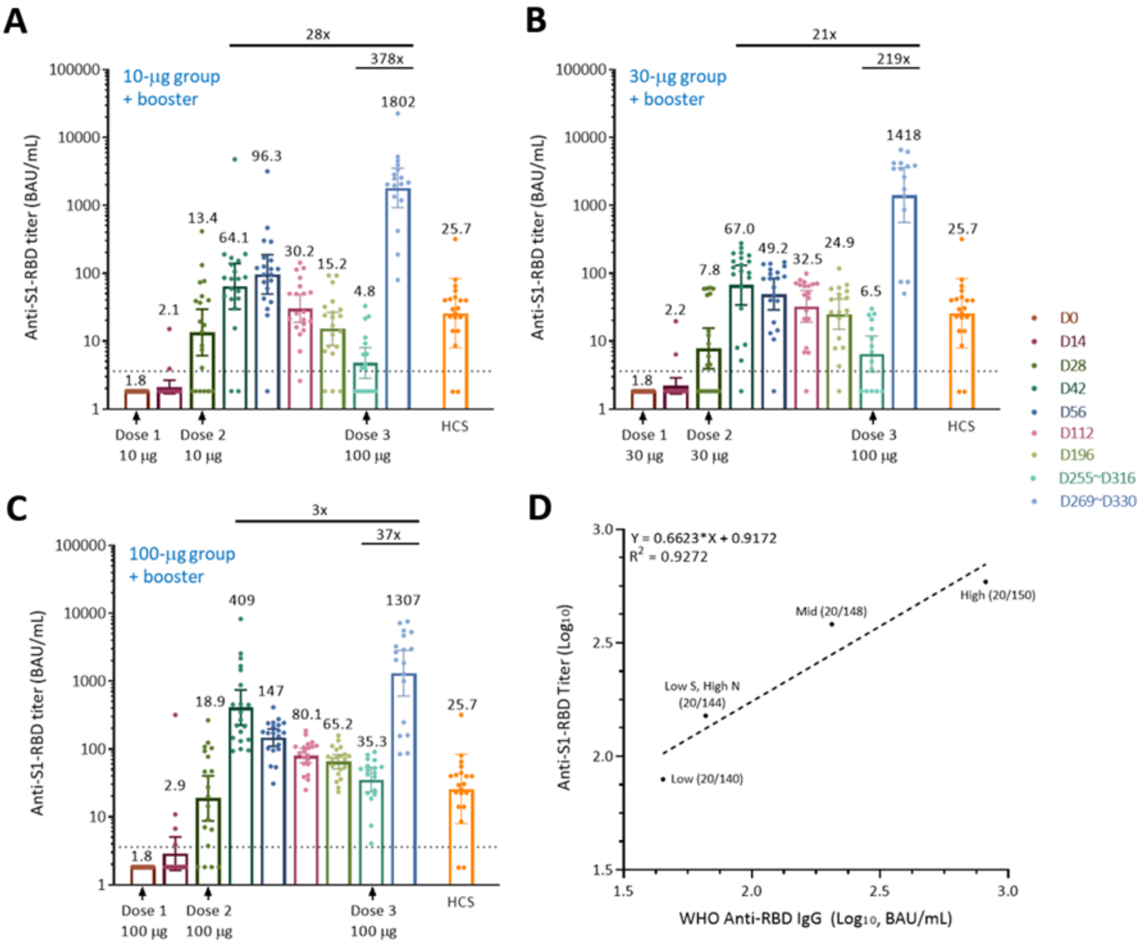

ELISA-based anti-S1-RBD antibody binding titers in the primary 2-dose vaccination series of a 196-day phase 1 trial (60 participants) and in the extension study with a booster third-dose. Participants of **(A)** 10-µg, **(B)** 30-µg, and **(C)** 100-µg dose groups (n = 20 each dose group) received two assigned vaccine doses, 28 days apart, and a booster third dose of 100 µg at a time over 6 months administered to 50 participants (n = 17 for the 10-µg, n = 15 for the 30-µg, and n = 18 for the 100-µg dose groups). Serum samples were collected at the indicted time points for measuring anti-S1-RBD antibody binding by ELISA. The horizontal dotted lines indicate the lower limit of quantification (LLOQ). **(D)** The binding titers are translated into Binding Antibodies Unit (BAU/mL) by fitting anti-S1-RBD titers and WHO International Reference panel 20/2689 with simple liner regression (Pearson r = 0.9559). Good correlation exists between anti-S1-RBD antibody binding and BAU. Data are plotted for all prime/boost vaccinated participants (10-, 30- and 100-µg dose groups). Data points for participants at day 0 are excluded from correlation analysis. Correlation is analysed by Nonparametric Spearman correlation method.

SUPPLEMENTAL TABLES

Table S1. In the phase-1 trial of UB-612 for the 10-, 30-, and 100-µg dose group, viral-neutralizing GMT titers (VNT_{50}) against SARS-CoV-2 wild type (Wuhan strain) observed on Day 14 post-dose 2 in the primary 2-dose vaccination and on Day 14 after the booster third-dose of 100 µg.

Group	Time point	n	Median [IQR]	Range	GMT (95%CI)	n paired	*P* value*
10 µg	Dose 2 + 14D	20	48.6 [28.3 - 95.1]	2.5 - 990.7	44.5 (23 - 85.8)		
	Dose 3 + 14D	17	4064 [2717 - 7984]	718.4 - 81920	4643 (2629 - 8200)	17	<0.0001
30 µg	Dose 2 + 14D	20	49.9 [15.8 - 60.7]	2.5 - 226.3	31.3 (17.2 - 57.1)		
	Dose 3 + 14D	15	4064 [2560 - 10240]	226.3 - 20480	3698 (1816 - 7530)	15	<0.0001
100 µg	Dose 2 + 14D	20	114 [48 - 203.7]	26.1 - 452.5	107.7 (72.4 - 160.1)		
	Dose 3 + 14D	18	4091 [1927 - 8277]	545.4 - 16255	3992 (2397 - 6650)	18	<0.0001

*P values shown pairwise comparison using Wilcoxon sign rank test using Dose 2+14D as reference time-point.

Table S2. Seroconversion Rate Based on the Wild Type SARS-CoV-2 Neutralizing Antibody Titres at Day 57 in Phase-1 trial

Visit	Statistics	UB-612 10 mcg	UB-612 30 mcg	UB-612 100 mcg
**Full Analysis set, N**		**20**	**20**	**20**
Day 14 since Dose1	n	20	20	20
	Seroconversion, n (%)	0 (0)	1 (5)	2 (10)
	95% CI	(0, 16.8)	(0.1, 24.9)	(1.2, 31.7)
Day 28 since Dose1	n	20	20	20
	Seroconversion, n (%)	9 (45.0)	6 (30.0)	10 (50.0)
	95% CI	(23.1, 68.5)	(11.9, 54.3)	(27.2, 72.8)
Day 42 since Dose1	n	20	20	20
	Seroconversion, n (%)	17 (85.0)	17 (85.0)	20 (100%)
	95% CI	(62.1, 96.8)	(62.1, 96.8)	(83.2, 100)
Day 56 since Dose1	n	20	20	20
	Seroconversion, n (%)	18 (90.0)	16 (80%)	20 (100%)
	95% CI	(68.3, 98.8)	(56.3, 94.3)	(83.2, 100)
Day 112 since Dose1	n	20	19	20
	Seroconversion, n (%)	12 (60.0%)	17 (89.5)	20 (100)
	95% CI	(36.1, 80.9)	(66.9, 98.7)	(83.2, 100)
Day 196 since Dose1	n	20	19	20
	Seroconversion, n (%)	14 (70.0)	18 (94.7)	20 (100)
	95% CI	(45.7, 88.1)	(74.0, 99.9)	(83.2, 100)

Abbreviations: N = number of participants in the population; n = number of participants in the specific category; %=percentage of participants with available data (n) as the denominator; CI = confidence interval. Note: Seroconversion was defined as at least 4-fold increase of post-study intervention antibody titres from the baseline titre (pre-vaccination).

Table S3 Seroconversion Rate Based on the Wild Type SARS-CoV-2 Neutralizing Antibody Titres at Day 57 in Phase-2 trial

Visit	Statistics	UB-612	Placebo
**Evaluable immunogenicity population, N**		**871**	**141**
Day 56 since Dose1	n	871	141
	Seroconversion, n (%)	829 (95.18)	2 (1.42)
	95% CI	(93.54, 96.50)	(0.17, 5.03)
**Subgroup: > 18 to < 65 years, N**		**731**	**120**
Day 56 since Dose1	n	731	120
	Seroconversion, n (%)	705 (96.44)	2 (1.67)
	95% CI	(94.83, 97.66)	(0.20, 5.89)
**Subgroup: ≥ 65 to 85 years, N**		**140**	**21**
Day 56 since Dose1	n	140	21
	Seroconversion, n (%)	124 (88.57)	0
	95% CI	(82.10, 93.32)	(0, 16.11)

Abbreviations: N = number of participants in the population; n = number of participants in the specific category; %=percentage of participants with available data (n) as the denominator; CI = confidence interval.

Note: Seroconversion was defined as at least 4-fold increase of post-study intervention antibody titres from the baseline titre (pre-vaccination).
