# Supplementary material for: A multitope SARS-CoV-2 vaccine provides long-lasting B cell and T cell immunity against Delta and Omicron variants
Source: J Clin Invest. 2022 May 16;132(10):e157707. doi: 10.1172/JCI157707 (PMC9106357; doi:10.1172/JCI157707)
Supplement: Supplemental appendices [file jci-132-157707-s106.pdf]

Appendix 1

[illegible]
10 May. 2021

CLINICAL STUDY PROTOCOL

A phase I, open-label study to evaluate the safety, tolerability, and immunogenicity of UB-612 vaccine in healthy adult volunteers

**Protocol Number:**	V-122
**EudraCT Number:**	Not Applicable
**Investigational Product:**	UB-612
**Phase:**	I
**Sponsor:**	聯亞生技開發股份有限公司<br>United Biomedical, Inc., Asia (UBI Asia),<br>Hsinchu County, Taiwan
Protocol Date:	**03 May**, 2021
Protocol Version:	**2.5**

CONFIDENTIAL
This protocol may not be reproduced or communicated to a third party without the written permission of United Biomedical, Inc., Asia

1 PROTOCOL APPROVAL SIGNATURES

Protocol Title: A phase I, open-label study to evaluate the safety, tolerability, and immunogenicity of UB-612 vaccine in healthy adult volunteers

Protocol Number: V-122

This study will be conducted in compliance with the clinical study protocol (and amendments), International Council for Harmonisation (ICH) guidelines for current Good Clinical Practice (cGCP) and applicable regulatory requirements.

Sponsor Signatory

United Biomedical, Inc., Asia (UBI Asia),
No. 45, Guangfu N. Rd., Hukou Township,
Hsinchu County 303, Taiwan (R.O.C.)

____
Signature

____
Date

2 SYNOPSIS

Protocol Number:

V-122

Title:

A phase I, open-label study to evaluate the safety, tolerability, and immunogenicity of UB-612 vaccine in healthy adult volunteers

Investigational Product:

UB-612 vaccine

Phase:

Phase I

Objectives:

Primary Objective:

To evaluate the safety and tolerability of the UB-612 vaccine in healthy adults.

Secondary Objective:

To evaluate the immunogenicity of the UB-612 vaccine in healthy adults.

Study Design:

This is a phase I, open-label, dose-escalation clinical study to evaluate the safety, tolerability and immunogenicity of 3 ascending doses of UB-612 COVID-19 vaccine in healthy adults. This study will be carried out in three groups:

(1) A group: 2 doses of UB-612 vaccine 10 μg

(2) B group: 2 doses of UB-612 vaccine 30 μg

(3) C group: 2 doses of UB-612 vaccine 100 μg

The 20 subjects in A group will be enrolled to receive two doses of 10 μg UB-612 vaccine at 28-day interval (Day 0, Day 28). If there is no safety concern regarding the UB-612 vaccinations in A group as determined by data and safety monitoring board (DSMB), the subjects in B group will be enrolled. Then subjects in C group will be recruited if no safety concerns after DSMB reviewed safety data in B group. The interim analysis will be performed when the last subject of each group completed Visit 9 (Day 56).

In each group, the first 6 subjects will be enrolled as a sentinel group to receive 1st vaccine and return at Day 3 and Day 7. If the 6 subjects in sentinel group have no ≥ grade 3 adverse reactions or serious adverse events (SAEs) related to vaccine till Day 7 as determined by DSMB, the remaining 14 subjects could be enrolled. After all subjects in A group completed Visit 4 (Day 7), DSMB will determine if B group recruitment could be initiated after review the safety data of A group. Recruitment of B and C group will follow the same schedule.

In this trail, there will be 11 clinical visits. Subjects will come to the clinics at Visit 1 (screening, Day -14 ~ -1), Visit 2 (Day 0, baseline, 1st vaccination), Visit 3 (Day 3, only for first 6 subjects in each group), Visit 4 (Day 7), Visit 5 (Day 14), Visit 6 (Day 28, 2nd vaccination), Visit 7 (Day 35), Visit 8 (Day 42), Visit 9 (Day 56), Visit 10 (Day 112, 3 months after 2nd vaccination), and Visit 11 (Day 196, 6 months after 2nd vaccination).

In all groups, an informed consent and the eligibility of subjects will be checked at screening period. The 1st study vaccine administration will be done immediately after blood draw at Visit 2 to evaluate baseline laboratory safety and humoral / cellular immune response. The first 6 subjects in each group will be closely monitored by the study staff at least 2-4 hours after vaccination, and subsequent 14 subjects in each group will be monitored at least 60 minutes after vaccination (for vital signs change and adverse events). The post-vaccination diary card for 7-day solicited adverse events after vaccination will be given to subjects with a suitable instruction. The first 6 subjects in each group will visit clinics at Visit 3 (Day 3) and Visit 4 (Day 7) for monitoring safety, and 14 subsequent subjects could be enrolled if no ≥ grade 3 AE or SAE related to vaccine is reported.

At Visit 4 (Day 7), subjects will return for laboratory safety check and have blood drawn for cellular immune response and hand in the diary card.

At Visit 5 (Day 14), the subjects will return to clinics and have blood drawn for humoral immune response.

At Visit 6 (Day 28), the 2nd study vaccine administration will be done after blood draw for humoral / cellular immune response and subjects will be closely monitored for 60 minutes after vaccination. The post-vaccination diary card will be dispensed to subjects.

At Visit 7 (Day 35), subjects will return for laboratory safety check and have blood drawn for cellular immune response and hand in the diary card.

At Visit 8 (Day 42), the subjects will return to clinics and have blood drawn for humoral immune response.

At Visit 9 (Day 56), the subjects will return to clinics and have blood drawn for humoral immune response.

At Visit 10 (Day 112) and Visit 11 (Day 196), the blood draws will be performed to observe the persistence of immunogenicity.

All adverse events and serious adverse events will be monitored from Day 0 until Day 196.

Number of Subjects:

Up to 60 subjects (20 subjects per group) will be enrolled into this study.

Treatment:

The UB-612 vaccines, 20 μg/mL, 60 μg/mL and 200 μg/mL, containing Adju-Phos® and UBI-1 Oligo (CpG1) adjuvant will be administered by an intramuscular (IM) injection, 28 days apart (on Day 0 and 28). The following dose regimens, 10 μg (0.5 mL), 30 μg (0.5 mL) and 100 μg (0.5mL), will be tested.

Subjects will be enrolled with sequential allocation to the UB-612 doses. After first 20 subjects in A group received 2 doses of UB-612 vaccine 10 μg, the next 20 subjects in B group will receive 2 doses of UB-612 vaccine 30 μg, and the final 20 subjects in C group will receive 2 doses of UB-612 vaccine 100 μg. All 3 groups vaccinations will be 28 days apart. The subjects in next ascending cohort will be enrolled only granted by DSMB after reviewing safety data of preceding cohort who completed Visit 4 (Day 7).

Duration of Treatment: 6 months

Study Population:

To be eligible for study entry, subjects must satisfy all of the following inclusion criteria:

1. Healthy male or non-pregnant female between the age of 20 and 55 years at time of enrolment.
2. Women of childbearing potential and men must agree to practice medically effective contraception from first vaccination until 3 months after the last vaccination. The acceptable effective contraception methods include:
    a. Male or female sterilization, implant, or intrauterine device;
    b. Injectable, pill, patch, ring plus one barrier method*;
    c. Two combined barrier methods*.

    *Effective barrier methods are diaphragm, male or female condoms, sponge, or spermicides (creams or gels that contain a chemical to kill sperm).
3. Able to understand the content and possible risks of informed consent and willing to sign the Informed Consent Form (ICF).
4. Able to understand and agrees to comply with all study procedures and be available for all study visits.
5. Negative serological test for Hepatitis B surface antigen (HBsAg), HCV RNA and HIV antibody screening.
6. Negative in serum antibodies (IgG) against UBI SARS-CoV-2 ELISA.

7. Negative result of RT-PCR (Reverse Transcriptase PCR) screening of nasopharyngeal or throat swabs for SARS-CoV-2.
8. Ear temperature ≤ 38.0℃.
9. The body mass index (BMI) of 18-30 kg/m^2, inclusive, at screening.
10. Indexes of blood routine, biochemistry and other laboratory tests** are within the normal ranges, or not clinically significant as judged by investigators.

    ** The items of laboratory tests will include blood routine (CBC, including Hb, Hct, RBC count, WBC count, WBC differential and platelet count), biochemistry (ALT, AST, total bilirubin, creatinine), and HbA1c.
11. Judged to be healthy by the investigator on the basis of medical history, physical examination, 12-lead ECG, vital signs (systolic/diastolic blood pressure, pulse rate, body temperature, respiratory rate), and clinical laboratory tests (blood biochemistry, routine) performed at screening.

Subjects will be excluded from the study if one or more of the following exclusion criteria is applicable:

1. History of anaphylaxis, urticarial, or other significant adverse reaction requiring medical intervention after receipt of a vaccine.
2. Female who is pregnant or positive in pregnancy test at screening or just prior to each vaccination administration.
3. Female who is breast-feeding or plans to breastfeed from the time of the first vaccination through 60 days after the last vaccination.
4. Any acute illness, as determined by the study investigator 3 days before first vaccination.
5. Evidence or history of clinically significant hematological, renal, endocrine, pulmonary, gastrointestinal, cardiovascular, hepatic, psychiatric, neurologic, or allergic disease (including drug allergies).
6. Known history of SARS or MERS.
7. Previous exposure to SARS-CoV-2 or receipt of an investigational vaccine product for the prevention of COVID-19, MERS or SARS.
8. Subjects who take part in another clinical study within 12 weeks prior to the day of informed consent.
9. With certain underlying medical conditions which are at increased risk for severe illness from COVID-19, such as chronic kidney disease, COPD (Chronic Obstruction Pulmonary Disease), serious heart conditions (e.g., heart failure, coronary heart disease, or cardiomyopathies), or with major chronic illness, such as asthma, diabetes, or thyroid disease, and other not well-controlled.
10. Congenital or acquired angioedema.
11. Immune deficiency/disorder, whether due to genetic defect, immunodeficiency disease or immunosuppressive therapy.
12. Platelet disorder or other bleeding disorder may cause injection contraindication.
13. Prior chronic administration (defined as ≥ 14 day of continuous use) of immunosuppressant or corticosteroids (equivalent to ≥ 20 mg daily of prednisone), cytotoxic treatment in last 6 months before first vaccination.
14. Prior administration of immunoglobulins and/or any blood products in last 4 months before first vaccination.
15. Prior administration of attenuated, nucleic acid (mRNA or DNA) or vectored vaccines in last 1 month before first vaccination or expectation of such vaccines in the month after the second vaccination.
16. Prior administration of subunit vaccine or inactivated vaccine in last 14 days before first vaccination or expectation of receipt of such vaccines in the 14 days after the second vaccination.
17. Current anti-tuberculosis(TB) therapy or history of TB.

18. Alcoholism or substance abuser.
19. History of malignancy within 5 years prior to screening visit, except basal cell carcinoma of the skin and cervical carcinoma in situ.
20. Any medical disease or condition that, in the opinion of the study investigator, may confound the results of the study or pose an additional risk to the subjects by their participation in the study.

Primary Endpoint(s):

- Occurrence of adverse reactions within 7 days after vaccination
- The percentage of subjects with ≥ Grade 3 adverse events within 7 days after vaccination

Secondary Endpoint(s):

- Occurrence of adverse events (AE) till Day 56
- Occurrence of serious adverse events (SAE) till Day 56
- Occurrence of serious adverse events during the whole follow-up period (6 months)
- Occurrence of adverse events of special interest during the study period
- Changes of safety laboratory measures
- Geometric mean titer (GMT) of antigen-specific antibody (Anti-S1-RBD) on Day 14, 28, 42, 56, 112, and 196.
- Seroconversion rate (SCR) of antigen-specific antibody on Day 14, 28, 42, 56, 112, and 196.
- Geometric mean fold increase of antigen-specific antibody on Day 14, 28, 42, 56, 112, and 196.

Exploratory Endpoint(s):

- GMT of neutralizing antibody against SARS-CoV-2 on Day 14, 28, 42, 56, 112 and 196
- SCR of neutralizing antibody against SARS-CoV-2 on Day 14, 28, 42, 56, 112 and 196
- Geometric mean fold increase of neutralizing antibody against SARS-CoV-2 on Day 14, 28, 42, 56, 112 and 196
- Distribution of titers
- Correlation between the immune response detected by ELISA and live virus neutralization test
- Positive rate and level of antigen-specific interferon-gamma (IFN-γ) measured by ELISpot on Day 7, 28, 35, and 196
- $CD4^+$ and $CD8^+$ T cell responses using intracellular cytokine staining and flow cytometry on Day 7, 28, 35, and 196.

Sample Size Determination

No formal statistical sample size and power computations are performed since the objectives of the study are to assess the safety and immunogenicity of the study vaccine. In this study, twenty subjects will be involved in each vaccine dose group. A total of 60 subjects will be recruited.

Data and Safety Monitoring Board (DSMB)

To enhance the safety and integrity of the study data, a DSMB will evaluate clinical and laboratory safety data of the first 6 subjects (the Sentinel Groups) in each group to evaluate the risk of remaining 14 subjects, and a total of 20 subjects in each group before advancing to the next higher dose level/cohort. The DSMB will consist of an independent medical monitor, a statistician, and 1 or more experts in vaccinology who have experience serving on DSMBs. The DSMB members will review safety data and to provide a recommendation on dosage escalation, or early termination in case there is a concern regarding safety. Both the DSMB and the Sponsor must approve further immunization in case there is no concern of safety.

Statistical Analysis

The Safety Set (SS) will consist of all subjects receiving at least one injection of the UB-612 vaccine. The Safety Set is for safety evaluation in analysis.

The Full Analysis Set (FAS) will consist of subjects who receive two vaccinations, have at least one immunogenicity assessment after vaccination, and without SARS-CoV-2 infection between Day 0 and Day 56. Subject receive prohibited medication/treatment/vaccine during pre-specified period has impact on immunogenicity may exclude from the FAS set.

The modified intention-to-treat Set (mITT) will consist of subjects who receive at least one vaccination and have at least one immunogenicity assessment after the first or second vaccination. The mITT is also analysed for immunogenicity evaluation.

The Per Protocol Set (PPS) will be a subset of FAS. PPS includes subjects who receive two vaccinations, have at least one immunogenicity assessment on Day 42 or 56 after vaccination, and without SARS-CoV-2 infection between Day 0 and Day 56. Subjects who had major protocol deviations as determined by the Study Team or who received prohibited medication/treatment/vaccine during the pre-specified period **leading to an impact on immunogenicity will be excluded from the PPS set**. The PPS is also analysed for immunogenicity evaluation as a sensitivity analysis.

All safety assessments, including AEs, PEs, VS, and clinical laboratory evaluations, where indicated, will be presented using descriptive statistics for each vaccine group of UB-612. Data will be summarized for each vaccine group and overall.

All adverse reactions within 7 days after vaccination will be summarized with numbers and percentages by study vaccine group and each vaccination. The percentage of subjects with ≥ Grade 3 adverse events within 7 days after vaccination will also be demonstrated by study vaccine group and each vaccination. AEs till Day 56 will be presented with number and percentage by system organ class, preferred term, and study vaccine groups. The number and percentage of SAEs till Day 56 will be displayed in summary table by study vaccine groups. The number and percentage of SAEs during the whole follow-up period (6 months) will be presented in summary table by study vaccine groups. Adverse events of special interest during the study period will be summarized with number and percentage from the mapping result by MedDRA.

Changes of safety laboratory measures will be summarized with descriptive statistics by study vaccine group and each time point. ANCOVA model with laboratory baseline values as covariate will analyze changes of safety laboratory measures for testing the difference among vaccine groups. Pair-wise comparisons of least square means from the ANCOVA model will be presented. Intra-group difference in safety laboratory measures will also be analyzed by paired t test.

If a subject is infected by SARS-CoV-2 between Day 0 and Day 56, immunogenicity data from he or she will not be included in analysis, but all SARS-CoV-2 infections occurred within 6 months after Day 56 should be documented and reported following the same procedure of SAE reporting, especial attention should be paid in case of the occurrence of vaccine-associated enhanced respiratory disease (VAERD).

Geometric mean titer (GMT) will be described by descriptive statistics and the 95% confidence interval for study vaccine group. Additionally, the difference among vaccine groups will be analyzed by ANCOVA model under log-transform data with baseline level as covariate, if appropriate. Pair-wise comparisons of least square means from the ANCOVA model will be presented. The reverse cumulative distribution plot will be provided to display the distribution of GMT by time points for each vaccine group.

Seroconversion rate (SCR) will be presented as count and percentage in frequency table, and the 95% exact (Clopper-Pearson) confidence interval will be provided as well. The group comparison among dose groups will be assessed by Fisher's exact test.

Geometric mean fold increase (GMI) in each study vaccine group is summarized by descriptive statistics and the 95% confidence interval for study vaccine group. The difference among vaccine groups will be analyzed by ANOVA. Pair-wise comparisons of least square means from the ANOVA model will be presented. The reverse cumulative distribution plot will be provided to display the distribution of GMI by time points for each vaccine group.

Safety Evaluations

Each subject must be carefully monitored for the development of any AEs throughout study period. Safety evaluations will be based on changes in physical examinations, vital signs and changes in laboratory parameters, and adverse event, including subject self-reporting.

All SARS-CoV-2 infections occurred within 6 months after the vaccination should be documented and reported following the same procedure of SAE reporting, and it is necessary to conduct a case investigation, Furthermore, the critically ill or dead cases need to continue to conduct special investigation, mainly to analyze where there is an ADE or VAERD phenomenon.

Schedule of Assessments

**Scheduled visit**	**1**	**2**	**3**	**4**	**5**	**6**	**7**	**8**	**9**	**10**	**11/ET**
**Test & observations**	**Screening**	**1st vaccination**				**2nd vaccination**				**Month 3 follow-up[g]**	**Month 6 follow-up[h]**
Day	**-14~-1**	**0**	**3[c] ±1day**	**7 ±1day**	**14 ±3days**	**28 ±3days**	**35 ±3days**	**42 ±3days**	**56 ±3days**	**112 ±5days**	**196 ±15days**
**Clinical assessment**											
Informed consent	X										
Inclusion/Exclusion Criteria	X	X									
Contraindication for second vaccination						X					
Demographics	X										
Medical history	X	X									
Physical Exam [a]	X	X	X	X	X	X	X	X	X	X	X
Vital sign	X	X	X	X	X	X	X	X	X	X	X
ECG	X	X [i]				X [i]			X		
**Laboratory assessment**											
HBsAg, HCV RNA, HIV antibody tests	X										
Nucleic acid test (nasopharyngeal/throat swabs)	X										
Serum antibodies (IgG) against SARS-CoV-2	X										
**Lab (Safety)**											
Blood routine	X			X		X	X				
Biochemistry	X[d]			X		X	X				
Pregnancy(HCG/urine)[b]	X	X				X					X
**Lab (Immunogenicity)**											
Humoral immune		X			X	X		X	X	X	X

**Scheduled visit**	**1**	**2**	**3**	**4**	**5**	**6**	**7**	**8**	**9**	**10**	**11/ET**
**Test & observations**	**Screening**	**1st vaccination**				**2nd vaccination**				**Month 3 follow-up[g]**	**Month 6 follow-up[h]**
Day	**-14~-1**	**0**	**3[c] ±1day**	**7 ±1day**	**14 ±3days**	**28 ±3days**	**35 ±3days**	**42 ±3days**	**56 ±3days**	**112 ±5days**	**196 ±15days**
response											
Cellular immune response		X		X		X	X				X
**Study Related Procedures**											
Vaccination		X				X					
Dispense diary card [e]		X				X					
Return diary card [e]				X			X				
AEs/SAEs		X	X	X	X	X	X	X	X	X	X
COVID-19 infection surveillance									X	X	X
Concomitant Medication	X	X	X	X	X	X	X	X	X	X[f]	X[f]

a: Body weight and height will be assessed at Visit 1 only.
b: Screening for pregnancy will be performed (serum β-HCG pregnancy test at screening and urine pregnancy test before vaccination on Day 0, 28 and 196, for WoCBP only). It is not required for postmenopausal or surgically sterilized women. A positive urine pregnancy test should be confirmed by a serum test. Serum pregnancy testing in lieu of urine pregnancy tests will not be considered a protocol deviation.
c: Only for first 6 subjects in each group.
d: HbA1c will be only assessed at Visit 1 only.
e: In case of e-diary, it is no dispensing or return activity.
f: Only record medication for SAE.
g: 3 months after 2nd vaccination
h: 6 months after 2nd vaccination
i: The ECG will be performed within 1 hour after vaccination.
ET: early termination

Blood collection at scheduled visits

**Scheduled visit**	**1**	**2**	**3**	**4**	**5**	**6**	**7**	**8**	**9**	**10**	**11/ET**
**Test & observations**	**Screening**	**1st vaccination**				**2nd vaccination**				**Month 3 follow-up**	**Month 6 follow-up**
Day	**-14~-1**	**0**	**3±1**	**7±1**	**14±3**	**28±3**	**35±3**	**42±3**	**56±3**	**112±5**	**196±15**
	**Blood volume (mL)**										
HBsAg, HCV RNA, HIV antibody tests	2										
Serum antibodies (IgG) against SARS-CoV-2	2										
**Lab (Safety)**											
Blood routine	4.5			4.5		4.5	4.5				
Biochemistry	2.5			2.5		2.5	2.5				
Pregnancy (HCG/urine)	1										
**Lab (Immunogenicity)**											
Humoral immune response		5			5	5		5	5	5	5
Cellular immune response		35		35		35	35				35
Total blood collection	12	40	0	42	5	47	42	5	5	5	40

Total amount of blood collection: 243 mL.
* If HLA genotyping will be performed, additional 2 mL of blood sample will be collected.

3 TABLE OF CONTENTS

1 Protocol Approval Signatures 2

2 Synopsis 3

3 Table of Contents 12

4 List of Abbreviations 16

5 Introduction 17

5.1 Study Rationale 17
5.2 Background 17
5.3 UB-612 COVID-19 Vaccine 18
5.4 Nonclinical Study 19
5.5 Risk/Benefit Assessment 22

6 Study Objectives & Endpoints 23

6.1 Primary Objective 23
6.2 Primary Endpoints 23
6.3 Secondary Objective 23
6.4 Secondary Endpoints 23
6.5 Exploratory Endpoints 23

7 Investigational Plan 25

7.1 Overall Study Design and Plan: Description 25
7.2 Schedule of Assessments 27
7.3 Discussion of Study Design 30
7.4 Selection of Study Population 30
7.4.1 Number of Planned Subjects 30
7.4.2 Inclusion Criteria 30
7.4.3 Exclusion Criteria 31
7.4.4 Contraindications to Second Vaccination 32
7.4.5 Removal of Subjects 33
7.4.5.1 Removal of Subjects from Immunogenicity Analysis 33
7.4.5.2 Removal of Subjects from the Study 33
7.4.5.3 Study Termination 34
7.4.5.4 Reporting and Follow-up of Pregnancies 34
7.4.5.5 SARS-CoV-2 infection 34
7.5 Investigational Products 35

7.5.1 Identity of Investigational Products 35
7.5.2 Investigational Products Administered 35
7.5.2.1 Injection Volume(s) 35
7.5.2.2 Injection Route and Rate 35
7.5.2.3 Emergency Event Management 35
7.5.3 Packaging and Labeling 36
7.5.4 Prior and Concomitant Therapy 36
7.5.4.1 Prohibited Medication/Therapy 36

8 Timing of Study Procedures 37

8.1 Visit 1 (Day -14 ~ -1) – Screening 37
8.2 Visit 2 (Day 0) –Baseline: 1st Vaccination 37
8.3 Visit 3 (Day 3 ± 1 day) 38
8.4 Visit 4 (Day 7 ± 1 day) 38
8.5 Visit 5 (Day 14 ± 3 days) 38
8.6 Visit 6 (Day 28 ± 3 days) –2nd Vaccination 38
8.7 Visit 7 (Day 35 ± 3 days) 39
8.8 Visit 8 (Day 42 ± 3 days) 39
8.9 Visit 9 (Day 56± 3 days) 39
8.10 Visit 10 (Day 112 ± 5 days) –Month 3 Follow-up 40
8.11 Visit 11 (Day 196 ± 15 days) –Month 6 Follow-up 40
8.12 Early Termination (ET) 40
8.13 Unscheduled Visit 40
8.14 Duration of Treatment 40

9 Immunogenicity and Safety Variables 41

9.1 Informed Consent Form 41
9.2 Demographics / Other Baseline Characteristics 41
9.3 Eligibility 41
9.4 Procedure for Screening 41
9.4.1 HBsAg, HCV RNA, HIV Antibody Screening 41
9.4.2 Nucleic Acid Test of SARS-CoV-2 Screening 41
9.4.3 SARS-CoV-2 Antibody Screening 41
9.4.4 Electrocardiogram(ECG) 42
9.5 Administration 42
9.6 Safety and Immunogenicity Measurements Assessed 42
9.6.1 Safety Variables 42

9.6.1.1 Physical Examination 42
9.6.1.2 Vital Sign 42
9.6.2 Clinical Laboratory Evaluation 43
9.6.3 Pregnancy Test 43
9.6.4 Self-Evaluation/Reporting (Solicited/Unsolicited Symptoms) 43
9.6.4.1 Solicited Symptoms 43
9.6.4.2 Unsolicited Adverse Event 46
9.6.5 Adverse events (AEs) 46
9.6.5.1 Definition of Adverse Events (AE) 46
9.6.5.2 Definition of Serious Adverse Events (SAE) 47
9.6.5.3 Definition of Unexpected Adverse Reaction Definition 48
9.6.5.4 Definition of Adverse Events Following Immunization (AEFI) [13] 48
9.6.5.5 Definition of Adverse Events of Special Interest (AESI) [13] 48
9.6.5.6 Assessment of Severity 49
9.6.5.7 The Relationship to Study Vaccine 50
9.6.5.8 Adverse Events Reporting 51
9.6.5.9 Human leukocyte antigen (HLA)-genotyping 52
9.6.6 Immunogenicity Assessments 52
9.6.6.1 Detection of ELISA Antibodies against S1-RBD of SARS-CoV-2 52
9.6.6.2 Detection of Antibody Titers Which Inhibit S1-RBD:ACE2 binding 52
9.6.6.3 Neutralizing Antibody Titers against SARS-CoV-2 52
9.6.6.4 Detection of T Cell Response 53
9.6.7 Overall Study Stopping Rules 53
9.6.8 Appropriateness of Measurements 53

9.7 Data and Safety Monitoring Board (DSMB) 53

9.8 Surveillance and Laboratory Diagnosis of SARS-CoV-2 Infection during Clinical Trial 54

10 Statistical Methods 55

10.1 Statistical and Analytical Plans 55

10.2 Datasets or Populations Analysed 55

10.3 Demographic and Other Baseline Characteristics 55

10.4 Safety Evaluation 56

10.4.1 Analysis of Primary Safety Endpoint 57
10.4.2 Analysis of Secondary Safety Endpoint 57

10.5 Immunogenicity Evaluation 58

10.5.1 Analysis of Immunogenicity Endpoints 58
10.5.2 Analysis of Exploratory Immunogenicity Endpoints 58

10.6 Interim Analysis 59

10.7 Handling of Missing Data 60

10.8 Determination of Sample Size 60

10.9 Protocol Deviations 60
11 Quality Assurance and Quality Control 61
11.1 Audit and Inspection 61
11.2 Monitoring 61
11.3 Data Management and Coding 61
12 Records and Supplies 62
12.1 Drug Accountability 62
12.2 Financing and Insurance 62
13 Ethics 63
13.1 Independent Ethics Committee or Institutional Review Board 63
13.2 Regulatory Authorities 63
13.3 Ethical Conduct of the Study 63
13.4 Informed Consent 63
13.5 Subject Confidentiality 64
14 Reporting and Publication, Including Archiving 65
15 References 66

4 LIST OF ABBREVIATIONS

ACE2	Angiotensin-converting enzyme 2
ADE	Antibody dependent enhancement (of viral replication)
AE	adverse event
AESI	adverse event of special interest
ALP	alkaline phosphatase
ALT	alanine aminotransferase
AST	aspartate aminotransferase
β-HCG	beta-human chorionic gonadotropin
BP	blood pressure
BUN	blood urea nitrogen
CBC	complete blood count
cGCP	current good clinical practice
CHO	Chinese hamster ovary
CRF	case report form
DSMB	Data and Safety Monitoring Board
ECG	Electrocardiogram
FDA	Food and Drug Administration
HBV	hepatitis B virus
HCT	hematocrit
HCV	hepatitis C virus
HIV	human immunodeficiency virus
HLA	human leukocyte antigen
HR	heart rate
ICF	informed consent form
ICH	International Council for Harmonisation
IEC	independent ethics committee
IM	intramuscular
IRB	institutional review board
N	number of subjects in the dataset or population
N/A	not applicable
ORF	open reading frame
PE	physical examination
RBD	receptor binding domain
RR	respiratory rate
RT-PCR	reverse transcriptase- polymerase chain reaction
SCR	seroconversion rate
SAE	serious adverse event
SUSAR	suspected unexpected serious adverse reaction
TB	tuberculosis
UB-612	United Biomedical's COVID-19 vaccine
VAERD	vaccine-associated enhanced respiratory disease
VS	vital signs
WBC	white blood cell
WoCBP	woman/women of childbearing potential
WPI	weeks post immunization

5 INTRODUCTION

5.1 Study Rationale

The global COVID-19 pandemic caused by the SARS-CoV-2 virus has made the development of an effective vaccine a top biomedical priority. Antibodies are essential elements of most vaccines and will likely be crucial component of an effective vaccine against SARS-CoV-2. Though plasma neutralizing activity is low in most convalescent individuals, the recurrent anti-SARS-CoV-2 RBD antibodies (the most immunogenic fragment within the SARS-CoV-2 Spike protein) with potent neutralizing activity can be found in individuals with unexceptional plasma neutralizing activity, suggesting that humans are intrinsically capable of generating anti-RBD antibodies that potently neutralize SARS-CoV-2. In addition, substantial activation of $CD4^+$ and $CD8^+$ T cells are required to prevent further infection and to help the clearance of virus after exposure. Thus, vaccines that efficiently induce neutralizing antibodies targeting the SARS-CoV-2 RBD and trigger SARS-CoV-2-specific cellular responses are anticipated to induce an optimal immunogenicity profile and achieve the prevention purpose.

5.2 Background

In December 2019, a cluster of patients with pneumonia surfaced in Wuhan, China. The culprit was quickly identified as a beta-coronavirus that has never been reported before, and the disease was named by WHO as Corona Virus Disease 2019 (COVID-19) and the virus that causes it by the International Committee on Taxonomy of Viruses (ICTV) as SARS-CoV-2 [1, 2]. As of July 25, 2020, a global outbreak has caused 15,811,700 confirmed cases in more than 220 countries or territories, with 641,243 deaths, making the SARS-CoV-2 pandemic a general public health event that has stirred up worldwide attention. Currently, the epidemic is still spreading and there is no effective means to prevent the infection.

SARS-CoV-2 is a positive-strand RNA virus that belongs to the group of Betacoronaviruses. The genome of SARS-CoV-2 is approximately 29,700 nucleotides long and shares 79.5% sequence identity with SARS-CoV [3]. The long ORF1ab polyprotein at 5' end of the genome encodes 15 or 16 non-structural proteins, and the 3' end encodes 4 major structural proteins, including the spike (S) protein, nucleocapsid (N) protein, membrane (M) protein, and the envelope (E) protein [4]. SARS-CoV-2 interacts with the receptor angiotensin converting enzyme 2 (ACE2) on host cells via receptor binding domain (RBD) of its S protein for viral entry and subsequent pathogenesis [5], resulting in severe respiratory illness with symptoms of fever, cough, and shortness of breath, and even death in severe cases [6].

Vaccines are the most effective and economical means to prevent and control infectious diseases [7]. The development of an effective vaccine against SARS-Co-2 infection is urgently required. Currently, more than 170 pharmaceutical companies and academic institutions worldwide have launched their programs on vaccine development against SARS-CoV-2. There are several

different types of vaccines under development; one of them is subunit vaccine. Subunit vaccines include one or more antigens with strong immunogenicity capable of efficiently stimulating the host immune system. In general, this type of vaccine is safer and easier to produce, but often requires the addition of adjuvants to elicit a strong protective immune response. So far, several institutions have initiated programs on the SARS-CoV-2 subunit vaccine, and almost all of them use the S protein as antigens. For example, the University of Queensland is developing a subunit vaccine based on the "molecular clamp" technology [8]. Clover Biopharmaceuticals Inc. revealed that they are developing a vaccine candidate against SARS-CoV-2 using the "Trimer-Tag" technology [9], and the trimeric S protein subunit vaccine candidate was produced via a mammalian cell expression system. Novavax, Inc. announced that they had produced multiple nanoparticle vaccine candidates based on S protein, and after assessing efficacy in animal models to identify an optimal vaccine candidate, began Phase I clinical testing in May, 2020. Besides, Johnson & Johnson, Pasteur Institute, Sanofi Pasteru, GSK, and Chongqing Zhifei Biological Products Co., Ltd. also started subunit vaccine development against SARS-CoV-2.

Safety is the most important issue that should be taken into consideration during drug and vaccine development, and some scientists urge that we should not rush to deploy COVID-19 vaccines and drugs without sufficient safety guarantees [10]. There have been concerns regarding vaccine-associated enhanced respiratory disease (VAERD) by certain candidate COVID-19 vaccine approaches, via antibody-dependent enhancement (ADE) or development of Th2 immunopathology [11]. Grifoni *et al.* [12] revealed predominant Th1 responses in convalescing COVID-19 cases, with little to no Th2 cytokines. Clearly more studies are required, but the data Grifoni *et al.* shown appear to predominantly represent a classic Th1 response to SARS-CoV-2.

A phase I clinical study will provide viable information on the safety, tolerability and immunogenicity of this SARS-CoV-2 vaccine (UB-612). Antibodies will be assessed to calculate the seroconversion rate in order to choose appropriate dosage of vaccine to apply in phase II study.

5.3 UB-612 COVID-19 Vaccine

United Biomedical (UBI) has developed a vaccine candidate against SARS-CoV-2 that is designed to activate both humoral and cellular responses. For SARS-CoV-2 immunogens, UB-612 includes a designer S1-RBD-sFc (SRsFc) fusion protein formulated with designer Th and CTL epitope peptides selected from immunodominant M, S2 and N regions known to bind to human MHC I and II. This mixture of designer Th/CTL peptides is designed to elicit T cell activation, memory recall and effector functions similar to that of natural COVID-19. The S1-RBD-sFc fusion protein incorporates both linear and conformation epitopes and induces high affinity antibodies to the RBD of SARS-CoV-2. The immunogen components are formulated with an oligonucleotide containing unmethylated CpG motifs and Adju-Phos® adjuvants, which

promotes the activation of antigen-presenting cells pathways to induce an optimal immunogenicity profile and achieve the prevention purpose.

In summary, UB-612 vaccine design composition (S1-RBD-sFC+Th and CTL peptides+CpG, formulated with Adju-Phos®) is expected not only to be safe and inducing high titers of neutralizing antibodies, but also to provide T cell memory for a long lasting protection against COVID-19 across all human subjects irrespective of age, sex and ethnicities.

5.4 Nonclinical Study

A series of pharmacology studies in rats and mice have demonstrated that UB-612 is a promising vaccine candidate able to elicit a robust humoral and a cell-mediated immune response. UB-612 can induce high titers of anti-S1-RBD antibodies along with potent inhibitory effect against the S1-RBD binding to ACE2 receptor and the infection in Vero-E6 cells infected by live SARS-CoV-2 virus (CPE). Moreover, the presence of Th/CTL epitope peptides in UB-612 is indispensable for activating cellular immunity against SARS-CoV-2.

Rat study. In a dose-ranging, adjuvant-selection study in rats receiving ISA51/CpG3- or Adju-Phos/CpG1-formulated UB-612 vaccine at weeks 0 and 2, immune sera collected at 0, 2, 3, and 4 weeks post initial immunization (WPI) were analysed on ELISA for S1-RBD binding titers. The results show that UB-612 formulated in two different adjuvant systems exhibits equipotent immunogenicity across all doses from 10 to 300 μg (**Figures 5-1 A and B**).

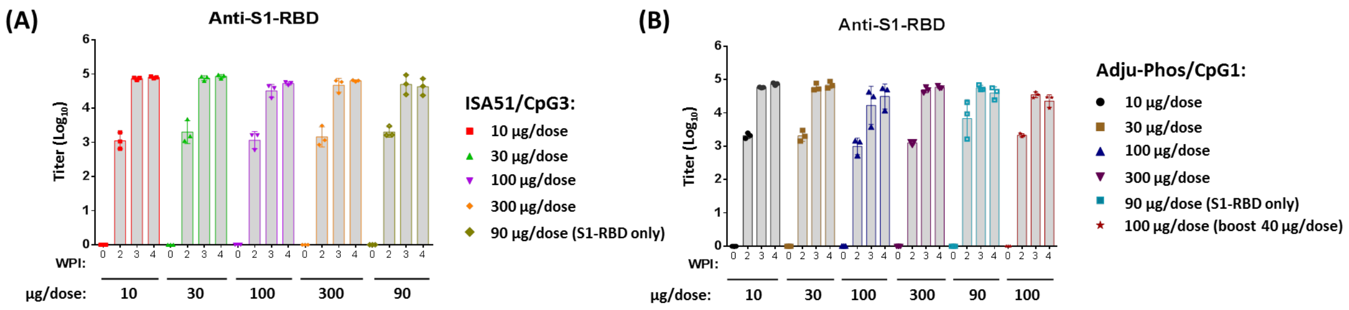

Figure 5-1. Direct binding of rat immune sera to S1-RBD protein on ELISA. Rats were immunized with different doses of UB-612 adjuvated with ISA51/CpG3 or Adju-Phos/CpG1 as indicated (n = 3 each group) at weeks 0 and 2 and immune sera were collected at 0, 2, 3 and 4 WPI. The anti-S1-RBD binding of immune sera collected from animals immunized with ISA51/CpG3- **(A)** and Adju-Phos/CpG1-adjuvated **(B)** vaccines were assayed by ELISA. Data represent the mean ± SD for each dose group of 3 rats.

UB-612 can induce high neutralizing activity to block viral infection. Immune sera collected at 4 WPI from vaccinated rats were assessed for CPE against infection in Vero-E6 cells challenged with live SARS-CoV-2 virus isolate (SARS-CoV-2-TCDC#4), UB-612 at low doses 10-30 μg (adjuvanted with ISA51/CpG3 or Adju-Phos/CpG1) could neutralize viral infection at VNT_{50} of

>10,240 dilution fold, better than by the higher doses at 100-300 µg (**Figure 5-2 A**). Similar results were obtained with S1-RBD:ACE2 binding inhibition on ELISA (**Figures 5-2 B-D**).

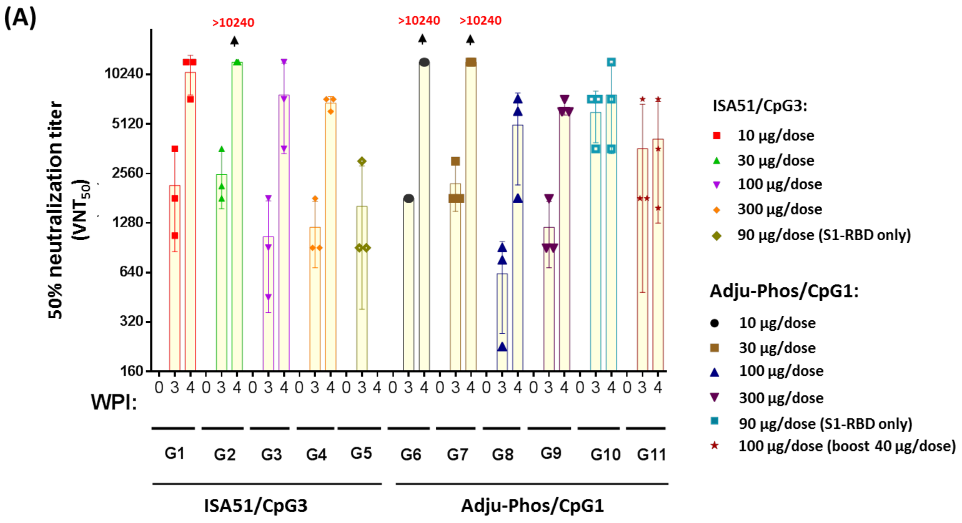

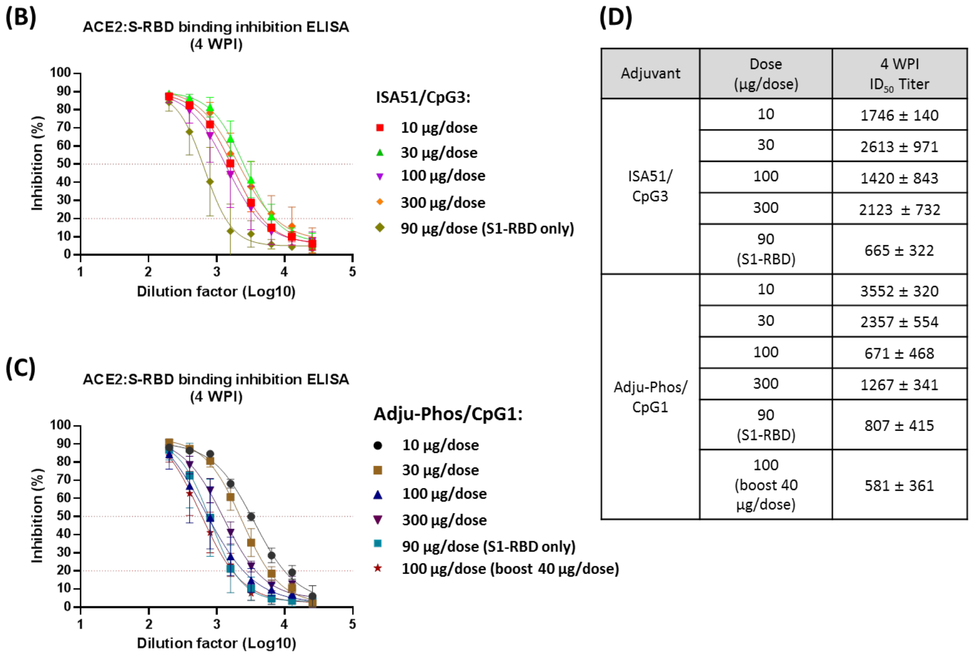

(D)

Adjuvant	Dose (µg/dose)	4 WPI ID_{50} Titer
ISA51/ CpG3	10	1746 ± 140
	30	2613 ± 971
	100	1420 ± 843
	300	2123 ± 732
	90 (S1-RBD)	665 ± 322
Adju-Phos/ CpG1	10	3552 ± 320
	30	2357 ± 554
	100	671 ± 468
	300	1267 ± 341
	90 (S1-RBD)	807 ± 415
	100 (boost 40 µg/dose)	581 ± 361

Figure 5-2. Potent neutralizing activity of immune sera from UB-612 vaccinated rats. Rats were immunized at weeks 0 and 2 with indicated vaccines. **(A)** Immune sera collected at 0, 3, and 4 WPI were analyzed on the SARS-CoV-2 infected Vero-E6 cells for cytopathic effect (CPE) assay. Neutralization VNT_{50} (50% Inhibitory Dilution Fold against viral infection) for immune sera were expressed. Each

symbol represents one individual animal. **(B)** Immune sera collected at 4 WPI were also assayed for inhibition of S1-RBD binding to ACE2 protein on ELISA **(B, C, and D)**. All data represent the mean ± SD for each dose group of 3 rats.

Mouse study. A cellular immunity assessment study in mice has been conducted to address the post-vaccination Th1/Th2 balance of UB-612. Mice (BALB/c) were immunized at weeks 0 and 2 with different UB-612 vaccine compositions adjuvated with ISA51/CpG3 or Adju-Phos/CpG1. Splenocytes collected from vaccinated mice at 4 WPI were stimulated with S1-RBD protein, Th./CTL peptide pool, or their combination, followed by ELISpot analysis for cellular secretion of IFN-γ, IL-2 and IL-4. The results show that a substantial increase in the secretion of IFN-γ and IL-2 were observed in the groups receiving vaccines containing Th/CTL epitope peptides upon re-stimulation with Th/CTL peptide pool, and there was no difference between ISA51/CpG3 and Adju-Phos/CpG1-adjuvated systems (**Figures 5-3 A-F**).

As the presence of alum is known to induce Th2-polarized immune response, a higher secretion of IL-4 was observed across all experimental settings of splenocytes collected from mice vaccinated with Adju-Phos/CpG1-adjuvanted UB-612, as compared to the counterparts of ISA51/CpG3-adjuvanted UB-612 (**Figures 5-3 G-I**). The IL-4 responses under stimulation with Th/CTL peptide pool (**Figures 5-3 G and 5I**) tended to be higher than those by S1-RBD alone (**Figure 5-3 J**).

The overall results by ELISpot suggest the presence of Th/CTL is indispensable for triggering SARS-CoV-2-specific cellular immune response, and more importantly, a balanced Th1 and Th2 cellular immune response is induced by UB-612 vaccination.

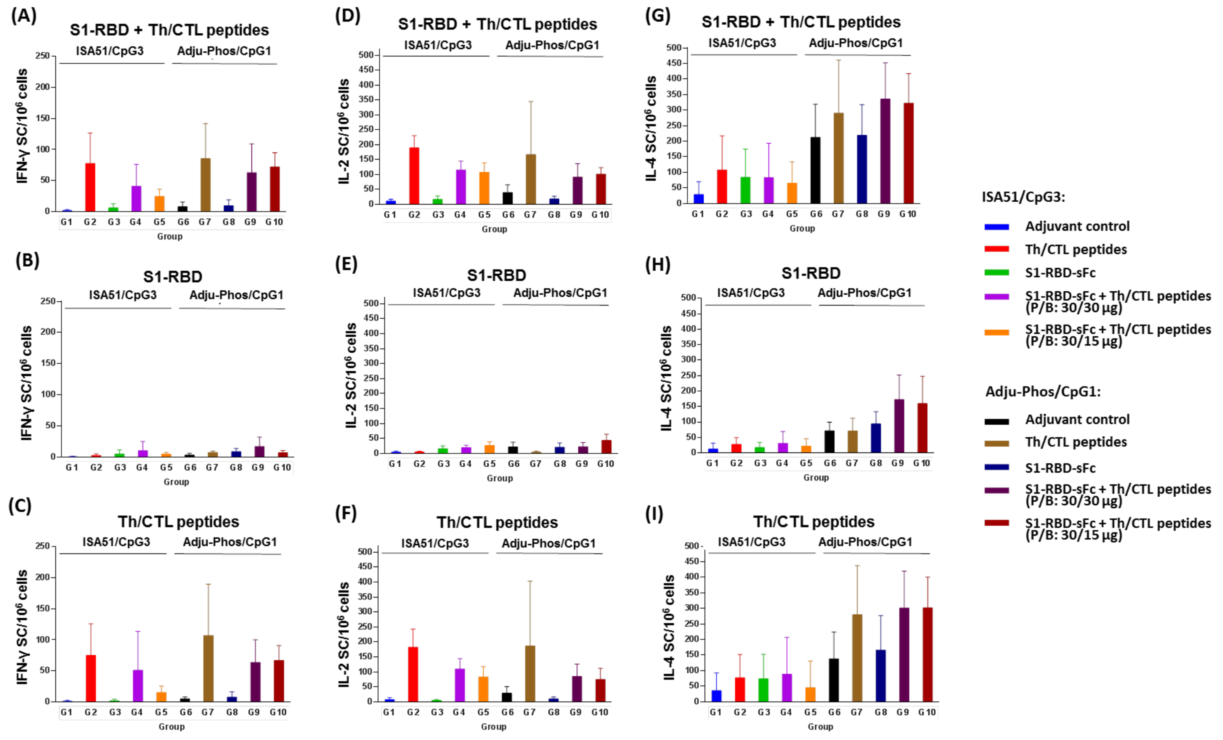

Figure 5-3. Assessment of UB-612-induced cellular response by ELISpot. Groups of mice were immunized with indicated vaccines with 2-doses spaced 2-weeks apart. Splenocytes were collected at 4 WPI and stimulated with S1-RBD protein plus Th/CTL peptide pool **(A, D, G)**, S1-RBD protein **(B, E, H)**, or Th/CTL peptide pool **(C, F, I)**. IFN-γ **- (A-C)**, IL-2**- (D-F)**, and IL-4- **(G-I)** secreting splenocytes were determined by ELISpot analysis. Bars represent the mean ± SD (n = 5).

5.5 Risk/Benefit Assessment

Nonclinical studies of UB-612 antigens have shown that IM delivery of the vaccine is (1) safe and well-tolerated in tested animals and (2) effective in inducing potent anti-S1-RBD antibody responses. Antibodies elicited by UB-612 antigens exhibit high neutralizing activity, which blocks the protein-protein interaction between ACE2 and S1-RBD and prevent SARS-CoV-2-induced *in vitro* cytopathology. Based on the promising nonclinical pharmacology data with the addition of ongoing safety evaluation in toxicology study, we aim to further evaluate the safety, tolerability and immunogenicity of UB-612 in healthy adult volunteers. The First-in-Human (FIH) study is a single-center, open-label, dose-escalating phase I clinical trial. Three different dose regimens [prime-boost (P/B) immunization with dosage of 10/10, 30/30 and 100/100 μg] of UB-612 will be tested in healthy adult volunteers between 20 and 55 years of age. Twenty subjects will be enrolled into one of the two vaccination regimen cohorts and will receive two intramuscular injections of UB-612 at a 28-day interval (Day 0 and Day 28). Safety monitoring reviews will be held to decide if the study can go on to second immunization for each cohort or to the higher dose regimens.

For safety and tolerability assessments, solicited AEs (collected by AE diary) and unsolicited AEs (from on-site/laboratory examination and subject spontaneous reporting) will be followed throughout the study period. Levels of anti-S1-RBD antibodies, neutralizing antibodies, and cellular immune responses will be examined after each vaccination for immunogenicity and pharmacodynamic evaluations.

6 STUDY OBJECTIVES & ENDPOINTS

6.1 Primary Objective

The primary objective is to evaluate the safety and tolerability of the UB-612 vaccine in healthy adults.

6.2 Primary Endpoints

- Occurrence of adverse reaction within 7 days after vaccination
  * Adverse reaction is defined as post-vaccination solicited adverse event, and unsolicited adverse event which is related to study vaccine.
- The percentage of subjects with ≥ Grade 3 adverse events within 7 days after vaccination

6.3 Secondary Objective

The secondary objective is to evaluate the immunogenicity of the UB-612 vaccine in healthy adults.

6.4 Secondary Endpoints

<u>Safety:</u>

- Occurrence of adverse events (AEs) till Day 56
- Occurrence of serious adverse events (SAEs) till Day 56
- Occurrence of SAEs during the whole follow-up period (6 months)
- Occurrence of AEs of special interest during the study period
- Changes of safety laboratory measures

<u>Immunogenicity:</u>

- Geometric mean titer (GMT) of antigen-specific antibody (Anti-S1-RBD) on Day 14, 28, 42, 56, 112, and 196.
- Seroconversion rate (SCR) of antigen-specific antibody on Day 14, 28, 42, 56, 112, and 196.
- Geometric mean fold increase of antigen-specific antibody on Day 14, 28, 42, 56, 112, and 196.

6.5 Exploratory Endpoints

- GMT of neutralizing antibody against SARS-CoV-2 on Day 14, 28, 42, 56, 112 and 196.
- SCR of neutralizing antibody against SARS-CoV-2 on Day 14, 28, 42, 56, 112 and 196.
- Geometric mean fold increase of neutralizing antibody against SARS-CoV-2 on Day 14, 28, 42, 56, 112 and 196.

- Distribution of titers
- Correlation between the immune response detected by ELISA and live virus neutralization test
- Positive rate and level of antigen-specific interferon-gamma (IFN-γ) measured by ELISpot on Day 7, 28, 35, and 196.
- $CD4^+$ and $CD8^+$ T cell responses using intracellular cytokine staining and flow cytometry on Day 7, 28, 35, and 196.

7 INVESTIGATIONAL PLAN

7.1 Overall Study Design and Plan: Description

This is a phase I, open-label, dose-escalation clinical study to evaluate the safety, tolerability and immunogenicity of 3 ascending doses of UB-612 COVID-19 vaccine in healthy adults. This study will be carried out in three groups:

(1) A group: 2 doses of UB-612 vaccine 10 μg

(2) B group: 2 doses of UB-612 vaccine 30 μg

(3) C group: 2 doses of UB-612 vaccine 100 μg

The 20 subjects in A group will be enrolled to receive two doses of UB-612 vaccine 10 μg at 28-day interval (Day 0, Day 28). If there is no safety concern regarding the UB-612 vaccinations in A group as determined by data and safety monitoring board (DSMB), the subjects in B group will be enrolled. Then subjects in C group will be recruited if no safety concerns after DSMB reviewed safety data in B group. The interim analysis will be performed when the last subject of each group completed Visit 9 (Day 56).

In each group, the first 6 subjects will be enrolled as a sentinel group to receive 1st vaccine and return at Day 3 and 7. If the 6 subjects in sentinel group have no ≥ grade 3 adverse reactions or serious adverse events related to vaccine till Day 7 as determined by DSMB, the remaining 14 subjects could be enrolled. After all subjects in A group completed Visit 4 (Day 7), DSMB will determine if B group recruitment could be initiated after review the safety data of A group. Recruitment of B and C group will follow the same schedule.

There will be consisted of 11 clinical visits. Subjects will come to the clinics at Visit 1 (screening, Day -14 ~ -1), Visit 2 (Day 0, baseline, 1st vaccination), Visit 3 (Day 3, only for the first 6 subjects in each group), Visit 4 (Day 7), Visit 5 (Day 14), Visit 6 (Day 28, 2nd vaccination), Visit 7 (Day 35), Visit 8 (Day 42), Visit 9 (Day 56), Visit 10 (Day 112, 3 months after 2nd vaccination), and Visit 11 (Day 196, 6 months after 2nd vaccination).

In all groups, an informed consent and the eligibility of subjects will be checked at screening period. The 1st study vaccine administration will be done immediately after blood draw at Visit 2 to evaluate baseline laboratory safety and humoral / cellular immune response. The first 6 subjects in each group will be closely monitored by the study staff at least 2-4 hours after vaccination, and subsequent 14 subjects in each group will be monitored at least 60 minutes after vaccination (for vital signs change and adverse events). The post-vaccination diary card for 7-day solicited adverse events after vaccination will be given to subjects with a suitable instruction. The first 6 subjects in each group will visit clinics at Day 3 (Visit 3) and Day 7 (Visit 4) for monitoring safety, and 14 subsequent subjects could be enrolled if no ≥ grade 3 AE or SAE related to vaccine is reported.

At Visit 4 (Day 7), subjects will return for laboratory safety check and have blood drawn for cellular immune response and hand in the diary card.

At Visit 5 (Day 14), the subjects will return to clinics and have blood drawn for humoral immune response.

At Visit 6 (Day 28), the 2nd study vaccine administration will be done after blood draw for

humoral / cellular immune response, and subject will be closely monitored for 60 minutes after vaccination. The post-vaccination diary card will be dispensed to subject.

At Visit 7 (Day 35), subjects will return for laboratory safety check, have blood drawn for cellular immune response, and hand in the diary card.

At Visit 8 (Day 42), the subjects will return to clinics and have blood drawn for humoral immune response.

At Visit 9 (Day 56), the subject will return to clinics and have blood drawn for humoral immune response.

At Visit 10 (Day 112) and Visit 11 (Day 196), the blood draws will be performed to observe the persistence of immunogenicity.

All adverse events and serious adverse events will be monitored from Day 0 until Day 196.

7.2 Schedule of Assessments

Below is a list of all study procedures through the study period and the signs "X" indicate when the procedures are performed.

**Scheduled visit**	**1**	**2**	**3**	**4**	**5**	**6**	**7**	**8**	**9**	**10**	**11/ET**
**Test & observations**	**Screening**	**1st vaccination**				**2nd vaccination**				**Month 3 follow-up[g]**	**Month 6 follow-up[h]**
Day	**-14~-1**	**0**	**3[c] ±1day**	**7 ±1day**	**14 ±3days**	**28 ±3days**	**35 ±3days**	**42 ±3days**	**56 ±3days**	**112 ±5days**	**196 ±15days**
**Clinical assessment**											
Informed consent	X										
Inclusion/Exclusion Criteria	X	X									
Contraindication for second vaccination						X					
Demographics	X										
Medical history	X	X									
Physical Exam [a]	X	X	X	X	X	X	X	X	X	X	X
Vital sign	X	X	X	X	X	X	X	X	X	X	X
ECG	X	X [i]				X [i]			X		
**Laboratory assessment**											
HBsAg, HCV RNA, HIV antibody tests	X										
Nucleic acid test (nasopharyngeal/throat swabs)	X										
Serum antibodies (IgG) against SARS-CoV-2	X										
**Lab (Safety)**											
Blood routine	X			X		X	X				
Biochemistry	X[d]			X		X	X				
Pregnancy(HCG/urine)[b]	X	X				X					X

**Scheduled visit**	**1**	**2**	**3**	**4**	**5**	**6**	**7**	**8**	**9**	**10**	**11/ET**
**Test & observations**	**Screening**	**1st vaccination**				**2nd vaccination**				**Month 3 follow-up[g]**	**Month 6 follow-up[h]**
Day	**-14~-1**	**0**	**3[c]<br>±1day**	**7<br>±1day**	**14<br>±3days**	**28<br>±3days**	**35<br>±3days**	**42<br>±3days**	**56<br>±3days**	**112<br>±5days**	**196<br>±15days**
**Lab (Immunogenicity)**											
Humoral immune response		X			X	X		X	X	X	X
Cellular immune response		X		X		X	X				X
**Study Related Procedures**											
Vaccination		X				X					
Dispense diary card [e]		X				X					
Return diary card [e]				X			X				
AEs/SAEs		X	X	X	X	X	X	X	X	X	X
COVID-19 infection surveillance									X	X	X
Concomitant Medication	X	X	X	X	X	X	X	X	X	X[f]	X[f]

a: Body weight and height will be assessed at Visit 1 only.
b: Screening for pregnancy will be performed (serum β-HCG pregnancy test at screening and urine pregnancy test before vaccination on Day 0, 28 and 196, for WoCBP only). It is not required for postmenopausal or surgically sterilized women. A positive urine pregnancy test should be confirmed by a serum test. Serum pregnancy testing in lieu of urine pregnancy tests will not be considered a protocol deviation.
c: Only for first 6 subjects in each group.
d: HbA1c will be only assessed at Visit 1 only.
e: In case of e-diary, it is no dispensing or return activity.
f: Only record medication for SAE.
g: 3 months after 2nd vaccination
h: 6 months after 2nd vaccination
i: The ECG will be performed within 1 hour after vaccination.
ET: early termination

Blood collection at scheduled visits

**Scheduled visit**	**1**	**2**	**3**	**4**	**5**	**6**	**7**	**8**	**9**	**10**	**11/ET**
**Test & observations**	**Screening**	**1st vaccination**				**2nd vaccination**				**Month 3 follow-up**	**Month 6 follow-up**
Day	**-14~-1**	**0**	**3±1**	**7±1**	**14±3**	**28±3**	**35±3**	**42±3**	**56±3**	**112±5**	**196±15**
**Blood volume (mL)**											
HBsAg, HCV RNA, HIV antibody tests	2										
Serum antibodies (IgG) against SARS-CoV-2	2										
**Lab (Safety)**											
Blood routine	4.5			4.5		4.5	4.5				
Biochemistry	2.5			2.5		2.5	2.5				
Pregnancy (HCG/urine)	1										
**Lab (Immunogenicity)**											
Humoral immune response		5			5	5		5	5	5	5
Cellular immune response		35		35		35	35				35
Total blood collection	12	40	0	42	5	47	42	5	5	5	40

Total amount of blood collection: 243 mL.
* If HLA genotyping will be performed, additional 2 mL of blood sample will be collected.

7.3 Discussion of Study Design

The objectives of this study are to evaluate the safety and tolerability of 3 ascending doses of UB-612. This will be done through this phase I, open-label, dose-escalation study in healthy adults. The trial will be carried out step by step from the low dose group to the high dose group, and the sentinel review approach will be employed for safety assessment. The decision of dose-escalation will be made by DSMB. Safety evaluation, including solicited post vaccination clinical events, will monitor subjects for any potential adverse reactions during and after vaccination. The expected or unexpected vaccine-related adverse events will also be assessed. The study will stop temperately to recruit subjects if any serious safety issues occur. DSMB will determine the continuation of the study.

The population of eligible subjects will be healthy adults that are suitable to explore the safety and tolerability of the vaccine. The immunogenicity will be also assessed at 7-14 days after each vaccination, and in subsequent long-term visits for observing persistence of immune responses. This dose-finding strategy will be employed to explore the suitable immunogenicity/tolerability profile for UB-612 vaccine.

In order to provide relevant safety and immunogenicity data to plan for future studies of UB-612, top-level administrative analyses for Groups A, B and C (Administrative Analysis #1: Days 0-28; Administrative Analysis #2: Days 28-42) will be performed on available safety and immunogenicity data for the two week periods after each immunization.

7.4 Selection of Study Population

7.4.1 Number of Planned Subjects

Up to 60 subjects (20 subjects per arm) will be enrolled into this study.

If a subject is withdrawn from the study, except for those subjects who will be terminated early due to AEs, the subject may be replaced as necessary with another subject assigned to the same dose. Further subjects may be enrolled at a given dose level if additional data are necessary to establish safety and tolerability prior to dose escalation suggested by DSMB.

7.4.2 Inclusion Criteria

To be eligible for study entry subjects must satisfy all of the following inclusion criteria:

1. Healthy male or non-pregnant female between the age of 20 and 55 years at time of enrolment.
2. Women of childbearing potential and men must agree to practice medically effective contraception from first vaccination until 3 months after the last vaccination. The acceptable effective contraception methods include:
    a. Male or female sterilization, implant, or intrauterine device;
    b. Injectable, pill, patch, ring plus one barrier method*;
    c. Two combined barrier methods*.

*Effective barrier methods are diaphragm, male or female condoms, sponge, or spermicides (creams or gels that contain a chemical to kill sperm).

3. Able to understand the content and possible risks of informed consent and willing to sign the Informed Consent Form (ICF).
4. Able to understand and agrees to comply with all study procedures and be available for all study visits.
5. Negative serological test for Hepatitis B surface antigen (HBsAg), HCV RNA and HIV antibody screening.
6. Negative in serum antibodies (IgG) against UBI SARS-CoV-2 ELISA.
7. Negative result of RT-PCR screening of nasopharyngeal or throat swabs for SARS-CoV-2.
8. Ear temperature ≤ 38.0°C.
9. The body mass index (BMI) of 18-30 kg/m^2, inclusive, at screening.
10. Indexes of blood routine, biochemistry and other laboratory tests** are within the normal ranges, or not clinically significant judged by investigators.

** The items of laboratory tests will include blood routine (CBC, including Hb, Hct, RBC count, WBC count, WBC differential and platelet count), biochemistry (ALT, AST, total bilirubin, creatinine), and HbA1c.

11. Judged to be healthy by the investigator on the basis of medical history, physical examination, 12-lead ECG, vital signs (systolic/diastolic blood pressure, pulse rate, body temperature, respiratory rate), and clinical laboratory tests (blood biochemistry, routine) performed at screening.

7.4.3 Exclusion Criteria

Subjects will be excluded from the study if one or more of the following exclusion criteria is applicable:

1. History of anaphylaxis, urticarial, or other significant adverse reaction requiring medical intervention after receipt of a vaccine.
2. Female who is pregnant or positive in pregnancy test at screening or just prior to each vaccination administration.
3. Female who is breast-feeding or plans to breastfeed from the time of the first vaccination through 60 days after the last vaccination.
4. Any acute illness, as determined by the study investigator 3 days before first vaccination.
5. Evidence or history of clinically significant hematological, renal, endocrine, pulmonary, gastrointestinal, cardiovascular, hepatic, psychiatric, neurologic, or allergic disease (including drug allergies).
6. Known history of SARS or MERS.

7. Previous exposure to SARS-CoV-2 or receipt of an investigational vaccine product for the prevention of COVID-19, MERS or SARS.
8. Subjects who take part in another clinical study within 12 weeks prior to the day of informed consent.
9. With certain underlying medical conditions which are at increased risk for severe illness from COVID-19, such as chronic kidney disease, COPD (Chronic Obstruction Pulmonary Disease), serious heart conditions (e.g., heart failure, coronary heart disease, or cardiomyopathies), or with major chronic illness, such as asthma, diabetes, or thyroid disease, and other not well-controlled.
10. Congenital or acquired angioedema.
11. Immune deficiency/disorder, whether due to genetic defect, immunodeficiency disease or immunosuppressive therapy.
12. Platelet disorder or other bleeding disorder may cause injection contraindication.
13. Prior chronic administration (defined as ≥14 day of continuous use) of immunosuppressant or corticosteroids (equivalent to ≥ 20 mg daily of prednisone), cytotoxic treatment in last 6 months before first vaccination.
14. Prior administration of immunoglobulins and/or any blood products in last 4 months before first vaccination.
15. Prior administration of attenuated, nucleic acid (mRNA or DNA) or vectored vaccines in last 1 month before first vaccination or expectation of such vaccines in the month after the second vaccination.
16. Prior administration of subunit vaccine or inactivated vaccine in last 14 days before first vaccination or expectation of receipt of such vaccines in the 14 days after the second vaccination.
17. Current anti-tuberculosis(TB) therapy or history of TB.
18. Alcoholism or substance abuser
19. History of malignancy within 5 years prior to screening visit, except basal cell carcinoma of the skin and cervical carcinoma in situ.
20. Any medical disease or condition that, in the opinion of the study investigator, may confound the results of the study or pose an additional risk to the subjects by their participation in the study.

7.4.4 Contraindications to Second Vaccination

The following conditions constitute a contraindication to vaccination and should be checked prior to second vaccination:

1. Had any Grade 4 adverse reaction within 7 days after first dose.

2. Subject diagnosed with SARS-CoV-2 infection or has suspected SARS-CoV-2 infection based on symptoms according to investigator's judgment (lab test conformation is not necessary).

3. Had any SAE related to first dose during the follow-up of first dose.

4. Any condition that in the opinion of the investigator would be a contraindication to a second vaccination.

5. Subject has a fever.

6. Subject becomes pregnant.

If any AEs other than listed above or fever occurred at the time scheduled for vaccination, the subject may be vaccinated at a later date no later than 7 days when symptoms or fever were resolved.

Any subjects who receive the vaccination with dosage deviation or who does not receive the second vaccination on schedule may not necessarily be withdrawn from the study as further study procedures and the follow-up visits may be performed which will be decided by sponsor.

7.4.5 Removal of Subjects

7.4.5.1 Removal of Subjects from Immunogenicity Analysis

Subjects may stop study vaccine and withdraw from the immunogenicity analysis for any of the following reasons:

- Subjects did not receive 2 doses of UB-612 vaccine (refer to Section 7.4.4).
- Administration of prohibited medication/treatment/vaccine during pre-specified period which was enough to interfere immunogenicity.

Subjects who do not comply with the protocol will be replaced. Subjects who stop study vaccine for any other reason (i.e., AE) will not be replaced.

7.4.5.2 Removal of Subjects from the Study

Subjects may withdraw from the study for any of the following reasons:

- Lost to follow-up
- Consent withdrawal
- Death
- Any pathological event, clinical adverse event, or any change in the subject's status giving indication to the doctor that further participation in the study may not be the best interests of the subject, according to investigator's discretion.

Subjects who withdraw consent will be replaced.

Subjects are free to withdraw from the study at any time without providing reason(s) for withdrawal and without prejudice to further research treatment. The reason(s) for withdrawal will be documented in the case report form (CRF).

Subjects withdrawing from the study, except subject death, will be encouraged to complete the same final evaluations (as Visit 11 procedure) within 7 days after withdrawal, as subjects completing the study according to this protocol, particularly safety evaluations. The aim is to record data in the same way as for subjects who completed the study.

Reasonable efforts will be made to contact subjects who are lost to follow-up. These efforts must be documented in the subject's file.

7.4.5.3 Study Termination

The sponsor has the right to terminate the study at any time in case of SAEs or if special circumstances concerning the study agent or the company itself occur, making further research treatment of subjects impossible. In this event, the investigator(s) will be informed of the reason for study termination.

7.4.5.4 Reporting and Follow-up of Pregnancies

A positive urine pregnancy test should be confirmed by a serum pregnancy test. A negative pregnancy result is required before the subject may receive the study treatment. Subjects who become pregnant while on study must immediately discontinue study treatment. The pregnancy must be reported and recorded on the sponsor's pregnancy form within 24 hours of the investigator's or study site staff's acknowledgement of the pregnancy. Pregnancies for female subjects, or for the female partners of male subjects occurring within 3 months after last vaccination, must be reported to the sponsor. Pregnancies should be handled and reported as AEs.

The investigator should inform the subjects of the risks of continuing with the pregnancy and the possible side effects to the fetus. The outcome of all pregnancies (spontaneous miscarriage, elective termination, normal birth, or congenital abnormality) must be followed up and documented even if the subject is discontinued from the study.

All reports of congenital abnormalities/birth defects and spontaneous miscarriages should be handled and reported as SAEs, to be reported within 24 hours of site awareness (regardless of interval since study treatment). Elective abortions should be handled and reported as AEs.

7.4.5.5 SARS-CoV-2 infection

Subjects who completed 2 doses of study vaccine, SARS-CoV-2 infection should be confirmed. For subject who received vaccination per protocol, if he/she is infected by SARS-CoV-2 between Day 28 and Day 56, immunogenicity data from this subject will not be included in analysis. Subject with suspected SARS-CoV-2 infection will be recorded as AE, while infected with SARS-Co-V-2, which fulfilling any one or more of these criteria in the definition in section 9.6.5.2, will be documented and reported following the same procedure of SAE reporting, especial attention should be paid in case of the occurrence of antibody-dependent enhancement (ADE), or vaccine-associated enhanced respiratory disease (VAERD). Subjects encountered SARS-CoV-2 infection will not withdraw from the study, unless other withdrawal reason judged by investigators.

7.5 Investigational Products

7.5.1 Identity of Investigational Products

There will be three formulations for UB-612 vaccines. Each vial of 10 mL of UB-612 vaccine will be supplied in 10 mL glass vial.

Name	UB-612
Characteristics & Physical State	Transparent liquid with micro-particle
Formulated & Supplied by	United Biomedical, Inc.
Storage Conditions	Cooled (2°C -8°C) following receipt at site until the time of use
Shipments	Cooled (2°C -8°C)
Package	A disposable multi-dose vial containing 20, 60 or 200 µg/mL UB-612 protein/peptide as the following:<br>- 20 µg/mL: 17.6 µg S1-RBD-sFc protein and 2.4 µg of six peptides/per 1 mL included<br>- 60 µg/mL: 52.8 µg S1-RBD-sFc protein and 7.2 µg of six peptides/per 1 mL included<br>- 200 µg/mL: 176 µg S1-RBD-sFc protein and 24 µg of six peptides/per 1 mL included
Batch No.	303778, 303770, 303751

The vaccine lots used in this study have been tested and released by the quality control department of the UBI Pharma Inc. Non-clinical studies of UB-612 formulated with Adju-Phos® and CpG 1 are detailed in the Investigator's Brochure (IB).

7.5.2 Investigational Products Administered

7.5.2.1 Injection Volume(s)

	1st vaccination	2nd vaccination
A group	UB-612 vaccine 10 µg, 0.5mL	UB-612 vaccine 10 µg, 0.5mL
B group	UB-612 vaccine 30 µg, 0.5mL	UB-612 vaccine 30 µg, 0.5mL
C group	UB-612 vaccine 100 µg, 0.5mL	UB-612 vaccine 100 µg, 0.5mL

7.5.2.2 Injection Route and Rate

For this trial, UB-612 vaccine must be injected by an intramuscular (IM) route, 28 days apart (on Day 0 and Day 28). Injections on Day 0 and Day 28 will be given into alternate deltoid muscles, unless injection cannot be assessed for local reactogenicity, in which case the Day 0 and Day 28 injections may be given in the same deltoid muscle.

7.5.2.3 Emergency Event Management

The first 6 subjects in each group will be closely monitored by the study staff at least 2-4 hours after vaccination, and subsequent 14 subjects in each group will be monitored at least 60 minutes

after vaccination (for the change of vital sign or adverse event). Subjects will be encouraged to quickly report any symptoms at the time during this period. The necessary rescue material, equipment, and appropriate medications will be available in the clinic to allow rapid intervention in case of anaphylaxis or other emergency.

7.5.3 Packaging and Labeling

The study packaging will be performed by UBI Pharma Inc.

All packaging and labeling operations will be performed according to Good Manufacturing Practice for Medicinal Products and the relevant regulatory requirements. The labels for the outer box/vial box/vial of the study vaccine contain the following information: the name/address/telephone number of UBI Pharma Inc. , Product name, Study code, Indication, Package size, Dosage unit, Manufacture company, Batch No., Manufacture date, Expiration date, Storage conditions, Active ingredient concentration (only for UB-612 protein/peptide), Injection method, Study site, Visit date___, Visit No.___, Subject No.___, Study PI and words of caution stating the product is for investigational and clinical trial use only.

7.5.4 Prior and Concomitant Therapy

There is no specific known evidence of contraindications between the ingredients of UB-612 vaccine and other prior and concomitant therapy. Concomitant medications and therapies will be recorded beginning 6 months prior to 1st vaccination, as well as during study period.

7.5.4.1 Prohibited Medication/Therapy

The following medications or treatments which may affect the immunogenicity and clinical efficacy assessments will be prohibited during study period:

- Immunosuppressant or corticosteroids, cytotoxic treatment
- Immunoglobulins and/or any blood products
- Investigational product (including drug, vaccine)
- Other registered vaccine could be administered after Day 56, and should be apart from nearest visit at least one month.

8 TIMING OF STUDY PROCEDURES

8.1 Visit 1 (Day -14 ~ -1) – Screening

The following assessment(s) must be collected/performed at 2 weeks prior to Day 0. Clinical and laboratory evaluations performed as part of routine standard of care do not need to be repeated if performed within the appropriate window.

(1) Record date of informed consent will be signed. The following should be documented in the subject's medical chart: that they are participating in this study that informed consent has been obtained and that a copy of the consent has been given to the subject.
(2) Assign screen number
(3) Eligibility: Assess against the inclusion and exclusion criteria
(4) Demographics
(5) Medical history and concurrent diseases.
(6) Conduct physical exam including the measurement of weight and height
(7) Measure vital signs
(8) Perform 12 lead ECG
(9) Collect blood sample for following local laboratory tests:
  - A serum pregnancy test
  - Blood routine test
  - Biochemistry tests

(10) Collect blood sample for lab screening:
  - Hepatitis B surface antigen (HBsAg), HCV RNA and HIV antibody screening.
  - Serum antibodies (IgG) against SARS-CoV-2.

(11) RT-PCR screening of nasopharyngeal /throat swabs for SARS-CoV-2.
(12) Concomitant medications and procedures: Concomitant medications and procedures will be recorded.

8.2 Visit 2 (Day 0) –Baseline: 1st Vaccination

(1) Check inclusion and exclusion criteria
(2) Assign subject number
(3) Record any pre-vaccination medical event as medical history since last visit.
(4) Conduct physical examination.
(5) Measure vital signs.
(6) Perform a urine pregnancy test
(7) Collect blood sample for immunogenicity tests (central laboratory) before vaccination
  - Humoral immune response
  - Cellular immune response

(8) Perform 1st vaccination. Observe closely during vaccination and at least 2-4 hours after vaccination for the first 6 subjects in each group and at least 60 minutes after vaccination for subsequent 14 subjects at site (for the change of vital sign or adverse event).
(9) Perform 12 lead ECG within 1 hour after vaccination.
(10) Dispense self-evaluation diary card. Instruct the subject to monitor body temperature and complete the diary correctly.

Subject will be instructed to record any solicited adverse events occurring during a 7-day post-vaccination period on the diary card. If the subject perceives any signs or symptoms are progressing or serious, contact the investigator or their delegates immediately. Additional return visits can be scheduled by the investigators when necessary.

(11) Review concomitant medications.

8.3 Visit 3 (Day 3 ± 1 day)

(1) Check stopping criteria
(2) Conduct physical examination.
(3) Measure vital signs.
(4) Review concomitant medications.
(5) Record adverse or serious adverse event if any has occurred since the previous visit.

8.4 Visit 4 (Day 7 ± 1 day)

(1) Conduct physical examination.
(2) Measure vital signs.
(3) Collect blood sample for following local laboratory tests:
  - Blood routine test
  - Biochemistry tests
(4) Collect blood sample for immunogenicity tests (central laboratory):
  - Cellular immune response
(5) Collect the previous diary cards
(6) Review concomitant medications.
(7) Record and report adverse or serious adverse event if any has occurred since the previous visit.

8.5 Visit 5 (Day 14 ± 3 days)

(1) Conduct physical examination.
(2) Measure vital signs.
(3) Collect blood sample for immunogenicity test (central laboratory):
  - Humoral immune response
(4) Review concomitant medications.
(5) Record and report adverse or serious adverse event if any has occurred since the previous visit.

8.6 Visit 6 (Day 28 ± 3 days) –2nd Vaccination

(1) Check contraindication for second vaccination.
(2) Conduct physical examination.
(3) Measure vital signs.
(4) Perform a urine pregnancy test
(5) Collect blood sample for following local laboratory tests before vaccination:
  - Blood routine tests
  - Biochemistry tests
(6) Collect blood sample for immunogenicity tests (central laboratory) before vaccination:

- ➢ Humoral immune response
- ➢ Cellular immune response

(7) Perform 2nd vaccination. Observe closely during vaccination and at least 60 minutes after vaccine administration at site.

(8) Dispense self-evaluation diary card. Instruct the subject to monitor body temperature and complete the diary correctly.
Subject will be instructed to record any solicited adverse events occurring during a 7-days post-vaccination period on the diary card. If the subject perceives any signs or symptoms are progressing or serious, contact the investigator or their delegates immediately. Additional return visits can be scheduled by the investigators when necessary.

(9) Perform 12 lead ECG within 1 hour after vaccination.

(10) Review concomitant medications.

(11) Record and report adverse or serious adverse event if any has occurred since the previous visit.

8.7 Visit 7 (Day 35 ± 3 days)

(1) Conduct physical examination.
(2) Measure vital signs.
(3) Collect blood sample for following local laboratory tests:
- ➢ Blood routine test
- ➢ Biochemistry tests

(4) Collect blood sample for immunogenicity tests (central laboratory):
- ➢ Cellular immune response

(5) Collect the previous diary cards
(6) Review concomitant medications.
(7) Record and report adverse or serious adverse event if any has occurred since the previous visit.

8.8 Visit 8 (Day 42 ± 3 days)

(1) Conduct physical examination.
(2) Measure vital signs.
(3) Collect blood sample for immunogenicity test (central laboratory):
- ➢ Humoral immune response

(4) Review concomitant medications.
(5) Record and report adverse or serious adverse event if any has occurred since the previous visit.

8.9 Visit 9 (Day 56± 3 days)

(1) Conduct physical examination.
(2) Measure vital signs.
(3) Perform 12 lead ECG.
(4) Collect blood sample for immunogenicity test (central laboratory):
- ➢ Humoral immune response

(5) Review concomitant medications.
(6) Administer flu vaccine if subjects agree

(7) Conduct COVID-19 surveillance
(8) Record and report adverse or serious adverse event if any has occurred since the previous visit.

8.10 Visit 10 (Day 112 ± 5 days) –Month 3 Follow-up

(1) Conduct physical examination.
(2) Measure vital signs.
(3) Collect blood sample for immunogenicity test (central laboratory):
   - ➢ Humoral immune response
(4) Review concomitant medications.
(5) Conduct COVID-19 surveillance
(6) Record and report adverse or serious adverse event if any has occurred since the previous visit.

8.11 Visit 11 (Day 196 ± 15 days) –Month 6 Follow-up

(1) Conduct physical examination.
(2) Measure vital signs.
(3) Perform a urine pregnancy test
(4) Collect blood sample for immunogenicity tests (central laboratory):
   - ➢ Humoral immune response
   - ➢ Cellular immune response
(5) Review concomitant medications.
(6) Conduct COVID-19 surveillance
(7) Record and report adverse or serious adverse event if any has occurred since the previous visit.

8.12 Early Termination (ET)

For subjects who discontinue this study earlier, a final follow-up may be arranged not late than 7 days and all study procedures listed for Visit 11 should be completed.

8.13 Unscheduled Visit

Subjects who suffered from severe solicited AE or unsolicited AE at any moment, which are unexpected no matter the severity or event, should return to site ASAP for further survey and treatment.

8.14 Duration of Treatment

The duration of each visit is expected to last between 4 to 5 hours during 1 day, barring any unexpected adverse reactions.

It takes about 7 months for each subject to participate in the study, from recruiting to the last visit. Some subjects may withdraw the study during the course of the study.

9 IMMUNOGENICITY AND SAFETY VARIABLES

The planned schedule of assessments is in Section 7.2.

9.1 Informed Consent Form

The investigator or designee must explain to each subject the nature of the study, its purpose, the procedures involved, the expected duration, the potential risks and benefits involved and any potential adverse events. Each subject will be informed that participation in the study is voluntary and that they can be withdrawn from participation at any time.

All subjects must provide a signed and dated informed consent at Visit 1. An informed consent form must be approved by the Institutional Review Board (IRB), Ethics Committee (EC), and/or the applicable health authorities.

9.2 Demographics / Other Baseline Characteristics

The demographic and baseline characteristic data for subjects will be collected at Visit 1. The demographics include date of birth, age, sex and ethnicity. Relevant history/conditions include all those present prior to the administration of study vaccine that are listed below:

- Relevant medical history,
- All current medical conditions,
- Allergy history,

Whenever possible, diagnoses but not symptoms should be recorded.

9.3 Eligibility

Eligibility should be checked by the investigator at Visit 1 and Visit 2 before vaccination.

9.4 Procedure for Screening

9.4.1 HBsAg, HCV RNA, HIV Antibody Screening

During the screening process, blood sample will be collected for antibody/RNA screening, those with negative results can be enrolled.

9.4.2 Nucleic Acid Test of SARS-CoV-2 Screening

Nasopharyngeal/throat swabs are collected and detected by RT-PCR methods. Those who are positive for the nucleic acid of SARS-CoV-2 will not be enrolled.

9.4.3 SARS-CoV-2 Antibody Screening

Specific IgG antibodies against SARS-CoV-2 in the serum of the subjects will be tested by ELISA assay, those who are positive for any of the antibodies will not be enrolled.

9.4.4 Electrocardiogram(ECG)

A 12-lead electrocardiogram (ECG) will be performed by the investigator or a suitable qualified designee after measurement of vital signs. The ECG measurement should be obtained after the subject has been seated and at rest for at least 5 minutes. The ECGs will be performed at screening visit, Visit 2, Visit 6, and Visit 9. At Visit 2 and Visit 6, the ECG should be performed within 1 hour after vaccination.

Qualified physicians will interpret the ECG. Any abnormal ECG reading should be noted and recorded in the CRF.

Additional 12 lead ECG could be performed by the discretion of investigators.

9.5 Administration

Take a vial of investigational vaccine, use a disposable syringe to extract vaccine and intramuscularly inject it into the middle of the lateral deltoid muscle of the subject's upper arm.

Vaccine administration will be recorded, including quantity (volume and weight). The containers from which the vaccine was administered to the subjects will be retained for dose confirmation.

9.6 Safety and Immunogenicity Measurements Assessed

9.6.1 Safety Variables

9.6.1.1 Physical Examination

Complete physical examinations should be conducted by investigator/site staff at all visits. A complete physical exam will include the examination of general appearance, HEENT (head, ears, eyes, nose, throat), neck (including thyroid), lymph nodes, skin, cardiovascular, pulmonary, abdomen, neurological system and musculoskeletal/joints.

Body weight and height will be collected at Visit 1. Body weight will be measured in indoor clothing, but without shoes and blanket to the nearest 0.1 kilogram (kg). Body height will be measured in centimeters (cm).

It must be recorded when any abnormality has been found out.

9.6.1.2 Vital Sign

Systolic/diastolic blood pressure, pulse, respiratory rate, and ear temperature will be collected at all visits.

For two vaccination visits, vital sign should be measured prior to and after vaccine administration. Post vaccination vital sign monitoring should be performed every 30 minutes (at least) at site. The first 6 subjects in each group will be closely monitored by the study staff at least 2-4 hours after 1st vaccination. Vital signs within 60 minutes after vaccination will be collected in CRF, and vital signs at 0, 30, 60, 120, 180 minutes after vaccination will be documented in electronic medical record system.

Subsequent 14 subjects in each group will be monitored at least 60 minutes after vaccination, and vital signs should be recorded in CRF.

9.6.2 Clinical Laboratory Evaluation

The clinical laboratory analyses will be performed at local laboratories. Reference ranges will be supplied by the local laboratories and used by the investigator to assess the laboratory data for clinical significance and pathological changes.

Methods and timing for assessing, recording and analyzing each laboratory variable should follow local guidelines. The following laboratory safety tests will be performed:

Blood Routine

CBC, including Hb, Hct, RBC count, WBC count, WBC differential and platelet count.

Biochemistry

ALT, AST, total bilirubin, creatinine, and HbA1c

9.6.3 Pregnancy Test

Screening for pregnancy will be performed (serum β-HCG pregnancy test at screening and urine pregnancy test before vaccination on Day 0 and Day 28, and Day 196 for WoCBP only). It is not required for postmenopausal or surgically sterilized women.

A positive urine pregnancy test should be confirmed by a serum test. Pregnancy tests may be performed more frequently per request of Institutional Review Board (IRB)/Independent Ethics Committee (IEC) or if required by local regulations.

9.6.4 Self-Evaluation/Reporting (Solicited/Unsolicited Symptoms)

9.6.4.1 Solicited Symptoms

Information of solicited symptoms/AEs and body temperature will be collected by the subjects in the provided e-diary cards during a 7-day follow-up period after each vaccination (i.e. day of vaccination and 6 subsequent days), and reported by the investigator team. The subject should complete the assessments in the e-diary every evening.

A solicited local and general symptoms or AEs are the one whose nature or intensity is consistent with the expected AEs described and listed below.

Table 9-1 Grading for adverse events at the injection site

	**Mild (Grade 1)**	**Moderate (Grade 2)**	**Severe (Grade 3)**	**Potentially Life Threatening (Grade 4)**
Pain	Do not affect physical activity	Affect physical activity	Affect daily life	Emergency room (ER) visit or hospitalization
Induration*, swelling (optional)**#	Diameter 2.5~＜5 cm and does not affect or slightly affect daily life	Diameter 5~＜10 cm or interferes with daily life	Diameter ≥ 10 cm or prevents daily activity	Necrosis
Rash*, Redness (optional)** #	Diameter 2.5~＜5 cm	Diameter 5~＜10 cm	Diameter ≥ 10 cm	Necrosis or exfoliative dermatitis
Allergic Reaction	Itching at vaccine site, no rash	Localized urticaria	Generalized urticaria; angioedema	Anaphylaxis
Cellulitis	NA	Non-injectable treatment is required (e.g., oral antibacterial, antifungal, antiviral therapy)	Intravenous treatment is required (e.g., intravenous antibacterial, antifungal, antiviral therapy)	Sepsis, or tissue necrosis, etc.

Note: *: in addition to directly measuring the diameter for grading and evaluation, the progress of the measurement results should also be recorded.
** the maximum measuring diameter or area should be used.
# the evaluation and grading of induration and swelling, rash and redness should be based on the functional level and the actual measurement results, and the indicators with higher classification should be selected.

Table 9-2 Grading for systemic adverse events

	**Mild (Grade 1)**	**Moderate (Grade 2)**	**Severe (Grade 3)**	**Potentially Life Threatening (Grade 4)**
Diarrhea	2 – 3 loose stools /24 hours	4 – 5 stools /24 hours	6 or more watery stools/24 hours or requires outpatient IV hydration	ER visit or hospitalization for hypotensive shock
Fatigue	Does not affect daily activities	Affects normal daily activities	Seriously affects daily activities and cannot work	Emergency Room visit or hospitalization
Nausea/Vomiting	No interference with activity or 1 – 2 episodes/24 hours	Some interference with activity with activity or > 2 episodes/24 hours	Prevents daily activity, requires outpatient IV hydration	Life-threatening (e.g. hypotension shock)
Anorexia	Loss of appetite, but no reduction in food intake	Loss of appetite, reduced food intake	Loss of appetite and no food intake	Need for intervention (e.g. gastric tube feeding, parenteral nutrition)
Sore throat	Transient, without treatment, without affecting daily activities	Sore throat, slightly affecting daily activities	Severe sore throat that seriously affects daily activities and requires medication	
Headache	Does not affect daily activities	Transient, slightly affects daily activities and may require treatment or intervention	Seriously affects daily activities and requires treatment or intervention	ER visit or hospitalization
Cough	Transient, without treatment	Persistent cough, effective treatment	Paroxysmal cough, uncontrollable treatment	Emergency or hospitalization
Arthralgia	Mild pain without hindering function	Moderate pain; need analgesics and / or pain that impedes function but does not affect daily activities	Severe pain; need analgesics and / or pain affecting daily activities	Disability pain
Non-injection-site muscle pain	Does not affect daily activities	Slightly affect daily activities	Severe muscle pain that seriously affects daily activities	Emergency or hospitalization
Non-injection-site itching (no skin lesions)	Slightly itchy without affecting or slightly affecting daily life	Itching affects daily life	Itching makes it impossible to carry on daily life.	NA
Abnormal skin and mucosa	Erythema / itching / color change	Diffuse rash / macular papule / dryness / desquamation	Blister / exudation / desquamation / ulcer	Exfoliative dermatitis involving mucous membrane, or erythema multiforme, or suspected Stevens-Johnsons

	**Mild (Grade 1)**	**Moderate (Grade 2)**	**Severe (Grade 3)**	**Potentially Life Threatening (Grade 4)**
				syndrome
Acute allergic reaction *	Local urticaria (blister) without treatment	Local urticaria requiring treatment or mild angioedema without treatment	Extensive urticaria or angioedema requiring treatment or mild bronchospasm	Anaphylactic shock or life- threatening bronchospasm or throat edema
Syncope	Close to syncope without losing consciousness (pre-syncope)	Loss of consciousness without treatment	Loss of consciousness and needs treatment or hospitalization	NA
Acute bronchospasm	Transient; no treatment needed	Needs treatment; bronchodilator therapy returns to normal	Bronchodilator treatment cannot return to normal	Cyanosis; or intubation required
Dyspnea	Dyspnea during exercise	Dyspnea during normal activity	Dyspnea at rest	Dyspnea, requiring oxygen therapy, hospitalization or assisted breathing

* Refers to type I hypersensitivity.

The axillary temperature will be monitored every evening on day of vaccination and 6 subsequent days. If axillary temperature has been measured for more than one time, only the highest degree level should be recorded in the e-diary card.

Table 9-3 Grading for axillary temperature

	**Mild (Grade 1)**	**Moderate (Grade 2)**	**Severe (Grade 3)**	**Potentially Life Threatening (Grade 4)**
Fever (℃)	37.3～＜38.0	38.0～＜38.5	38.5～＜39.5	≥39.5, last more than 3 days

9.6.4.2 Unsolicited Adverse Event

An unsolicited local and general AE or symptoms are the one whose nature or intensity is NOT consistent with the expected AEs described and listed above in this protocol. Information of unsolicited symptoms/AEs will be collected until 28-day follow-up period after each vaccination (i.e. day of vaccination and 27 subsequent days) and reported at each visit.

9.6.5 Adverse events (AEs)

9.6.5.1 Definition of Adverse Events (AE)

An AE is any untoward medical occurrence in a patient or clinical investigation subject temporally associated with the use of a medicinal product, whether or not considered related to the medicinal product. The occurrence does not necessarily have to have a causal relationship with this treatment. An AE can therefore be any unfavorable and unintended sign (including an

abnormal laboratory finding), symptom, or disease (new or exacerbated) temporally associated with the use of a medicinal product, whether or not considered related to the medicinal product.

Examples of an AE include:

- Exacerbation of a chronic or intermittent pre-existing condition, including either an increase in frequency and/or intensity of the condition
- A new condition detected or diagnosed after informed consent, even though it may have been present prior to this
- Signs, symptoms, or the clinical sequelae of a suspected interaction
- Signs, symptoms, or the clinical sequelae of a suspected overdose of either study drug or a concurrent medication.

Examples of an AE do NOT include:

- A medical or surgical procedure (*e.g.* endoscopy); the condition that leads to the procedure is an AE
- Situations where an untoward medical occurrence did not occur (social and/or convenience admission to hospital)
- Anticipated day-to-day fluctuations of pre-existing disease(s) or condition(s) present or detected before informed consent was obtained, that do not worsen.

A priori, immunogenicity endpoints as specified in the protocol will not be considered as AEs except if, because of the course or severity or any other features of such events, the investigator, according to his/her best medical judgment, considers these events as exceptional in this medical condition.

9.6.5.2 Definition of Serious Adverse Events (SAE)

A SAE is any untoward medical occurrence that at any dose:

- Results in death or,
- Is life-threatening or,

  Note: The term "life-threatening" in the definition of "serious" refers to an event in which the subject was at risk of death at the time of the event; it does not refer to an event, which hypothetically might have caused death if it were more severe.

- Requires inpatient hospitalization or prolongation of existing hospitalization or,

  Note: In general, hospitalization signifies that the subject has been detained (usually involving at least an overnight stay) at the hospital or emergency ward for observation and/or treatment that would not have been appropriate in the physician's office or out-subject setting. Complications that occur during hospitalization are AEs. If a complication prolongs hospitalization or fulfils any other serious criteria, the event is serious. When in doubt as to whether hospitalization occurred or was necessary, the AE should be considered serious. Hospitalization for elective treatment of a pre-existing condition that did not worsen from baseline is not considered an AE.

- Results in persistent or significant disability/incapacity or,

Note: The term disability means a substantial disruption of a person's ability to conduct normal life functions. This definition is not intended to include experiences of relatively minor medical significance such as uncomplicated headache, nausea, vomiting, diarrhoea, influenza, and accidental trauma (e.g. sprained ankle), which may interfere or prevent everyday life functions but do not constitute a substantial disruption.

- Is a congenital anomaly/birth defect or,
- Is a medically important event

Note: Medical and scientific judgment should be exercised in deciding whether expedited reporting is appropriate in other situations, such as important medical events that may not be immediately life threatening or result in death or hospitalization but may jeopardize the subject or may require intervention to prevent one of the other outcomes listed in the definition above. These should also be considered serious. Examples of such events are invasive or malignant cancers, intensive treatment in an emergency room or at home for allergic bronchospasm, blood dyscrasias or convulsions that do not result in hospitalisation, or development of drug dependency or drug abuse.

An AE fulfilling any one or more of these criteria should be reported as a SAE, irrespective of the dose of drug given, and even if it is the result of an interaction or drug abuse.

A distinction should be drawn between serious and severe AEs. The term 'severe' is often used to describe the intensity (severity) of a specific event (as in mild, moderate, or severe myocardial infarction); the event itself, however, may be of relatively minor medical significance (such as severe headache). This is not the same as 'serious,' which is based on subject's event outcome or action criteria usually associated with events that pose a threat to a subject's life or functioning. The seriousness (not severity) serves as a guide for defining regulatory reporting obligations.

9.6.5.3 Definition of Unexpected Adverse Reaction Definition

An unexpected adverse reaction is any untoward and unintended response that is related to the administration of the study agent, at any dose that is not consistent with the applicable product information (e.g., current version of the Investigator's Brochure for an unauthorized investigational medicinal product or summary of product characteristics for an authorized product).

9.6.5.4 Definition of Adverse Events Following Immunization (AEFI) [13]

An adverse event following immunization (AEFI) is defined as any untoward medical occurrence which follows immunization, and which does not necessarily have a causal relationship with the usage of the vaccine. The adverse event may be any unfavorable or unintended sign, abnormal laboratory finding, symptom or disease. In this study, adverse events, including solicited and unsolicited, will be categorized as AEFIs.

9.6.5.5 Definition of Adverse Events of Special Interest (AESI) [13]

Adverse event of special interest (AESI) is further defined in Council for International Organizations of Medical Sciences (CIOMS) VII as:

An adverse event of special interest (serious or non-serious) is one of scientific and medical concern specific to the sponsor's product or specific target disease, for which ongoing monitoring and rapid communication by the investigator to the sponsor could be appropriate. Such an event might require further investigation in order to characterize and understand it. Depending on the nature of the event, rapid communication by the trial sponsor to other parties (e.g., regulators) might also be warranted. AESI will be collected during the study period.

The AESI relevant to vaccination in general applicable to COVID-19 vaccine listing below:

Table 9-4 The AESI relevant to vaccination in general applicable to COVID-19

Body System	AESI Type
Neurologic	Generalized convulsion, Guillain-Barré syndrome (GBS), acute disseminated encephalomyelitis (ADEM)
Hematologic	Thrombocytopenia
Immunologic	Anaphylaxis, vasculitides, enhanced disease following immunization
Respiratory	Acute respiratory distress syndrome (ARDS), pneumonitis
Other	Serious local/systemic AEFI, acute cardiac injury, arrhythmia, septic shock-like syndrome, acute kidney injury

9.6.5.6 Assessment of Severity

All AEs, except AEs in in e-diary card, will be assessed according to the US NCI Common Terminology Criteria for Adverse Events (CTCAE) 5.0 (published on November 27, 2017) associated with the AE term. The following standard with 5 grades is to be used to measure the severity of adverse events in this study.

Table 9-5 Intensity scales of AE

Grades of AE	Description
Grade 1	Mild; asymptomatic or mild symptoms; clinical or diagnostic observations only; intervention not indicated.
Grade 2	Moderate; minimal, local or noninvasive intervention indicated; limiting age-appropriate instrumental ADL*
Grade 3	Severe or medically significant but not immediately life-threatening; hospitalization or prolongation of hospitalization indicated; disabling, limiting self care ADL**
Grade 4	Life-threatening consequences; urgent intervention indicated.
Grade 5	Death related to AE.

* Instrumental activities of diary living (ADL) refer to preparing meals, shopping for groceries or clothes, using the telephone, managing money, etc.

** Self-care ADL refer to bathing, dressing and undressing, feeding self, using the toilet, taking medication, and not bedridden.

9.6.5.7 The Relationship to Study Vaccine

The investigator will make an assessment of the relationship between investigational vaccine and the occurrence of each AE/SAE, except solicited reactions to vaccination. The reasonable possibility will be determined based on the investigator's clinical judgment. The causality should be considered as one of the categories described below.

Table 9-6 The relationship between AE and study vaccine:

Causality term	Assessment criteria
Certain	• Event or laboratory test abnormality, with plausible time relationship to drug intake<br>• Cannot be explained by disease or other drugs<br>• Response to withdrawal plausible (pharmacologically, pathologically)<br>• Event definitive pharmacologically or phenomenologically (i.e. an objective and specific medical disorder or a recognized pharmacological phenomenon)<br>• Rechallenge satisfactory, if necessary
Probable / Likely	• Event or laboratory test abnormality, with reasonable time relationship to drug intake<br>• Unlikely to be attributed to disease or other drugs<br>• Response to withdrawal clinically reasonable<br>• Rechallenge not required
Possible	• Event or laboratory test abnormality, with reasonable time relationship to drug intake<br>• Could also be explained by disease or other drugs<br>• Information on drug withdrawal may be lacking or unclear
Unlikely	• Event or laboratory test abnormality, with a time to drug intake that makes a relationship improbable (but not impossible)<br>• Disease or other drugs provide plausible explanations
Unrelated	• Occurred before dosing<br>• Due wholly to factors other than study treatment

The serious adverse events should be followed till Day 196 for safety assessments. Each event should be followed until resolution or the event is considered stable. Both regular return and telephone contact will be acceptable.

9.6.5.8 Adverse Events Reporting

Documentation and Reporting of Adverse Events

All investigators should follow up subjects with AEs until the event is resolved or until, in the opinion of the investigator, the event is stabilized or determined to be chronic. Details of AE resolution must be documented in the CRF.

All AEs should be reported and documented in accordance with the procedures outlined in this section. All AEs occurring during the study must be documented on the relevant CRF pages.

Reporting of Serious Adverse Events

Any SAE must be reported by the investigator if it occurs during the clinical study or within 4-7 days of receiving the study agent, whether or not the SAE is considered to be related to the investigational product. An SAE report consists of the SAE form, the AE form, and the concomitant medication form. A copy of these forms must be emailed **within 24 hours** to Contract Research Organization (CRO), StatPlus Inc., and StatPlus will inform United Biomedical, Inc., Asia on the same day.

The investigator should not wait to receive additional information to document fully the event before notification of a SAE, though additional information may be requested. Where applicable, information from relevant laboratory results, hospital case records, and autopsy reports should be obtained.

Instances of death, congenital abnormality, or an event that is of such clinical concern as to influence the overall assessment of safety, if brought to the attention of the investigator at any time after cessation of study agent administration and linked by the investigator to this study, should be reported to the study monitor.

The sponsor and/or the appointed representative(s) will promptly notify all relevant investigators and the regulatory authorities of findings that could adversely affect the safety of subjects, impact on the conduct of the study, or alter the IEC/ IRB approval/favorable opinion of the study. In addition, the sponsor and/or the appointed representative(s), will expedite the reporting to all concerned investigators, to the IEC(s)/IRB(s), where required, and to the regulatory authorities of all adverse reactions that are both serious and unexpected.

Details of the procedures to be followed if a pregnancy occurs are also provided in Section 7.4.5.

Documentation and Reporting of SUSARs

All suspected unexpected serious adverse reactions (SUSARs) will be the subject of expedited reporting. The sponsor and/or the appointed representative(s) shall ensure that all relevant information about a SUSAR that is fatal or life-threatening is reported to the relevant competent authorities and IEC/IRB within 7 days after knowledge by the sponsor of such a case and that relevant follow-up information is communicated within an additional 8 days. All other SUSARs will be reported to the relevant competent authorities and IEC/IRB within 15 days after knowledge by the sponsor of such a case. All investigators should follow up SUSARs until the

event is resolved or until, in the opinion of the investigator, the event is stabilized or determined to be chronic. Poststudy SUSARs that occur after the subject has completed the clinical study must be reported by the investigator to the sponsor.

9.6.5.9 Human leukocyte antigen (HLA)-genotyping

In order to identify the MHC alleles in subjects with AESI and/or SAEs related to vaccine, an additional 2 mL of blood sample will be collected for HLA genotyping.

9.6.6 Immunogenicity Assessments

9.6.6.1 Detection of ELISA Antibodies against S1-RBD of SARS-CoV-2

Anti-S1-RBD antibody titers will be measured by ELISA kit. A serial dilution is to begin at 1:20, followed by 1:100, 1:1000, 1:10,000 and 1:100,000 for test sera. The levels of anti-S1-RBD antibody are expressed as linear titers of an end point dilution for each tested sample. SoftMax Titer Calculation Program (Molecular Devices Co.) is used to calculate the titers. For seroconversion detected by UBI SARS-CoV2 S1-RBD ELISA, it is defined as a 4-fold increase in antibody titer from baseline. If the titer is <20, a value 2-fold lower will be imputed (1:10). If the titer is >100,000, a titer 2-fold higher (1:200,000) will be imputed for the purpose of calculating the geometric mean and geometric mean fold increase. The practice will be applied on the specimens of all subjects.

9.6.6.2 Detection of Antibody Titers Which Inhibit S1-RBD:ACE2 binding

Antibody titers for ability to inhibit S1-RBD:ACE2 binding will be measured by ELISA kit. The dilution fold is at 1:10 for test sera. Antibody levels which inhibit S1-RBD:ACE2 binding are expressed in μg/mL as titers for a test sample based on a standard curve. SoftMax Titer Calculation Program (Molecular Devices Co.) is used to calculate the titers. Specimens that do not react in the test are considered with < 1.6 μg/mL titer in this SARS-CoV-2 qNeu Ab ELISA. For samples with a titer <1.6 **μg/mL**, a value 2-fold lower (i.e. 0.8 **μg/mL**) will be imputed for the purpose of calculating the GMT and GMFI. Similarly a titer with >**25 μg/mL** will be imputed 2-fold higher for calculation of the means.

9.6.6.3 Neutralizing Antibody Titers against SARS-CoV-2

Neutralizing antibody titers will be measured by CPE-based live virus neutralization assay using Vero-E6 cells challenged with SARS-CoV-2 (SARS-CoV-2-TCDC#4). The study will be conducted in P3 lab at Academia Sinica, Taipei. Determination of SARS-CoV-2 virus specific neutralization titer is to measure the neutralizing antibody titer against SARS-CoV-2 virus based on the principle of NT_{50} titer (≥ 50% reduction of virus-induced cytopathic effects). Virus neutralization titer of a serum is defined as the reciprocal of the highest serum dilution at which 50% reduction in cytopathic effects are observed and results are calculated by the method of Reed and Muench. In seroconversion rate detected by live virus neutralization test, it is defined as a 4-fold change in antibody titer from baseline.

9.6.6.4 Detection of T Cell Response

Human peripheral blood mononuclear cells will be used in the detection of T cell response. Antigen-specific interferon-gamma (IFN-γ) measurement to assess cellular (T cell) immune response will be measured by ELISpot method. Intracellular cytokine staining and flow cytometry will be used to evaluate $CD4^+$ and $CD8^+$ T cell responses.

9.6.7 Overall Study Stopping Rules

Study administration with UB-612 vaccine will be halted and the DSMB will convene to determine if the study should be terminated for any safety concerns. See Section 9.7 for DSMB details. Overall study stopping rules is listed below.

- One or more ≥ grade 4 adverse reaction or serious adverse event possibly associated with vaccination
- Occurrence of grade 3 adverse events associated with vaccination in 3 or more of subjects in a treatment group participants or more (including injection-site reaction, systemic reaction, and change of the safety laboratory measures)
- Required by sponsor
- Required by regulatory authority
- Required by institutional review board (IRB).
- The sponsor may also end the study for administrative reasons.

Should the study be terminated prematurely, the sponsor will provide written notification to all investigators and regulatory authorities specifying the reason(s) for early termination. The investigator must inform the institutional review board (IRB)/independent ethics committees (IEC) promptly and provide the reason(s) for the termination. Previously dosed subjects will be assessed through all planned study safety visits.

9.6.8 Appropriateness of Measurements

The immunogenicity and safety assessments planned for this study are generally recognized as reliable, accurate, and relevant to the diagnostic modality and underlying disease/condition.

9.7 Data and Safety Monitoring Board (DSMB)

To enhance the safety and integrity of the study data, a DSMB will evaluate clinical and laboratory safety data of the first 6 subjects (the Sentinel Groups) in each group to evaluate the risk of remaining 14 subjects, and a total of 20 subjects in each group before advancing to the next higher dose level/cohort. The DSMB will consist of an independent medical monitor, a statistician, and 1 or more experts in vaccinology who have experience serving on DSMBs. The DSMB members will review safety data and to provide a recommendation on dosage escalation, or early termination in case there is a concern regarding safety. Both the DSMB and the Sponsor must approve further immunization in case there is no concern of safety.

9.8 Surveillance and Laboratory Diagnosis of SARS-CoV-2 Infection during Clinical Trial

During the observation period of the clinical trial, the participants with fever, cough and other respiratory symptoms should immediately go to the designated hospital. The doctor or investigator will collect the nasopharyngeal/throat swabs and to perform CT and other imaging examinations to analyse whether it is caused by SARS- CoV-2 infection. In the event of SARS-CoV-2's infection during the clinical trial, it is necessary to conduct a case investigation, and the critically ill or dead cases need to continue to conduct a special investigation, mainly to analyze whether there is an ADE or VAERD phenomenon.

In addition to SARS-CoV-2 nucleic acid detection, multiple pathogens will be detected for differential diagnosis of swabs.

10 STATISTICAL METHODS

The statistical planning and analysis of the trial will be performed by the designated contract research organization.

10.1 Statistical and Analytical Plans

A statistical analysis plan will be prepared and finalized prior to database lock of the study. The statistical analysis plan will include full details of all planned statistical analyses.

10.2 Datasets or Populations Analysed

Safety Set

The Safety Set (SS) will consist of all subjects receiving at least one injection of the UB-612 vaccine. The Safety Set is for safety evaluation in analysis.

Full Analysis Set

The Full Analysis Set (FAS) will consist of subjects who receive two vaccinations, have at least one immunogenicity assessment after vaccination, and without SARS-CoV-2 infection between Day 0 and Day 56. Subject receive prohibited medication/treatment/vaccine during pre-specified period has impact on immunogenicity may exclude from the FAS set. The FAS set is the primary analysis set for immunogenicity evaluation.

Modified Intention-to-Treat Set

The modified intention-to-treat Set (mITT) will consist of subjects who receive at least one vaccination and have at least one immunogenicity assessment after the first or second vaccination. The mITT is also analysed for immunogenicity evaluation.

Per Protocol Set

The Per Protocol Set (PPS) will be a subset of FAS. PPS includes subjects who receive two vaccinations, have at least one immunogenicity assessment on Day 42 or 56 after vaccination, and without SARS-CoV-2 infection between Day 0 and Day 56. Subjects who had major protocol deviations as determined by the Study Team or who received prohibited medication/treatment/vaccine during the pre-specified period **leading to an impact on immunogenicity will be excluded from the PPS set**. The PPS is also analysed for immunogenicity evaluation as a sensitivity analysis.

10.3 Demographic and Other Baseline Characteristics

Demographic and baseline characteristic data will be summarized for each vaccine group and overall. Descriptive statistics (N, mean, standard deviation, median, minimum, and maximum) will be presented for continuous variables. The number and percentage of subjects in each category will be presented for categorical variables. No formal testing of demographic or baseline characteristics will be performed.

10.4 Safety Evaluation

All safety assessments, including AEs, PEs, VS, ECG and clinical laboratory evaluations, where indicated, will be presented using descriptive statistics for each vaccine group of UB-612. Data will be summarized for each vaccine group and overall.

Solicited Adverse Event

Solicited AEs recorded on e-diary will be summarized by the severity grading scales by vaccine groups. Numbers and percentages of subjects experiencing each adverse event will be presented for each symptom severity by study groups. Summary tables showing the occurrence of any local or systemic adverse event overall and at each time point will also be presented.

Adverse Event

This analysis applies to all adverse events occurring during the study, recorded in AE eCRF, with a start date on or after the date of vaccination. AEs occurring during the study will be coded using the most recent version of Medical Dictionary for Regulatory Activities (MedDRA). The adverse events will then be grouped by MedDRA preferred terms into frequency tables according to system organ class.

All reported adverse events, as well as adverse events judged by the investigator as at least possibly related to study vaccine, will be summarized according to system organ class and preferred term within system organ class. When an adverse event occurs more than once for a subject, the maximal severity and strongest relationship to the vaccine group will be counted.

Separate summaries will be produced for the following categories, such as serious adverse events and AEs related to UB-612 vaccine will be presented. Data listings of all adverse events will be provided by subject.

Physical Examination

All physical examination findings will be listed and summarized by time point and study vaccine group. Shift table will be also presented, if appropriate.

Vital Signs

All vital sign findings will be listed and summarized by time point and study vaccine group.

ECG

ECG findings will be listed and summarized by time point and study vaccine group. Shift table will be also presented, if appropriate.

Laboratory Evaluations

Laboratory safety data will be analysed descriptively by time point and study vaccine group. Shift table for laboratory data will be shown by visit using categorization of laboratory according to local laboratory's normal reference range.

10.4.1 Analysis of Primary Safety Endpoint

- Occurrence of adverse reactions within 7 days after vaccination
  All adverse reactions within 7 days after vaccination will be summarized with frequencies and percentages by study vaccine group and each vaccination.
- The percentage of subjects with ≥ Grade 3 adverse events within 7 days after vaccination
  The subject who has ≥ Grade 3 solicited AE or ≥ Grade 3 unsolicited AE within 7 days after vaccination will be counted. The number and percentage of counted subjects will be demonstrated by study vaccine group and each vaccination.

10.4.2 Analysis of Secondary Safety Endpoint

- Occurrence of adverse events (AE) till Day 56
  AEs till Day 56 will be presented with number and percentage by system organ class, preferred term, and study vaccine groups.
- Occurrence of serious adverse events (SAE) till Day 56
  The number and percentage of SAEs within 56 days will be displayed in summary table by study vaccine groups.
- Occurrence of serious adverse events during the whole follow-up period (6 months)
  The number and percentage of SAEs during the whole follow-up period (6 months) will be presented in summary table by study vaccine groups.
- Occurrence of adverse events of special interest during the study period
  Adverse events of special interest during the study period will be summarized with number and percentage from the mapping result by MedDRA. Details will be defined in the statistical analysis plan.
- Changes of safety laboratory measures
  Changes of safety laboratory measures will be summarized with descriptive statistics by study vaccine group and time point. ANCOVA model with laboratory baseline values as covariate will analyse changes of safety laboratory measures for testing the difference among study vaccine groups. Pair-wise comparisons of least square means from the ANCOVA model will be presented. Intra-group difference in safety laboratory measures will also be analysed by paired t test.

10.5 Immunogenicity Evaluation

10.5.1 Analysis of Immunogenicity Endpoints

- Geometric mean titer (GMT) of antigen-specific antibody (Anti-S1-RBD) on Day 14, 28, 42, 56, 112, and 196.
  GMT of antigen-specific antibody of antigen-specific antibody (Anti-S1-RBD) on Day 14, 28, 42, 56, 112, and 196 will be described by descriptive statistics and the 95% confidence interval for study vaccine group. Additionally, the difference among vaccine groups will be analyzed by ANCOVA model under log-transform data with baseline level as covariate, if appropriate. Pair-wise comparisons of least square means from the ANCOVA model will be presented. The reverse cumulative distribution plot will be provided to display the distribution of the GMT of Anti-S1-RBD by time points for each vaccine group.
- Seroconversion rate (SCR) of antigen-specific antibody (Anti-S1-RBD) on Day 14, 28, 42, 56, 112, and 196.
  The SCR will be presented as count and percentage in frequency table, and the 95% exact (Clopper-Pearson) confidence interval will be provided as well. The group comparison among dose groups will be assessed by Fisher's exact test.
- Geometric mean fold increase of antigen-specific antibody (Anti-S1-RBD) on Day 14, 28, 42, 56, 112, and 196.
  The geometric mean fold increase (GMI) in each study vaccine group is summarized by descriptive statistics and the 95% confidence interval for study vaccine group. The difference among vaccine groups will be analysed by ANOVA. Pair-wise comparisons of least square means from the ANOVA model will be presented. The reverse cumulative distribution plot will be provided to display the distribution of the GMI of Anti-S1-RBD by time points for each vaccine group.

10.5.2 Analysis of Exploratory Immunogenicity Endpoints

- GMT of neutralizing antibody against SARS-CoV-2 on Day 14, 28, 42, 56, 112 and 196

  GMT of neutralizing antibody against SARS-CoV-2 on Day 14, 28, 42, 56, 112 and 196 will be summarized by descriptive statistics and the 95% confidence interval for study vaccine group. Additionally, the difference among vaccine groups will be analysed by ANCOVA model under log-transform data with baseline level as covariate, if appropriate. Pair-wise comparisons of least square means from the ANCOVA model will be presented. The reverse cumulative distribution plot will be provided to display the distribution of the GMT of neutralizing antibody against SARS-CoV-2 by time points for each vaccine group.

- SCR of neutralizing antibody against SARS-CoV-2 on Day 14, 28, 42, 56, 112 and 196

  SCR of neutralizing antibody against SARS-CoV-2 on Day 14, 28, 42, 56, 112 and 196 will be presented as count and percentage in frequency table, and the 95% exact (Clopper-Pearson) confidence interval will be provided as well. The group comparison among dose groups will be assessed by Fisher's exact test.
- Geometric mean fold increase of neutralizing antibody against SARS-CoV-2 on Day 14, 28, 42, 56, 112 and 196

Geometric mean fold increase of neutralizing antibody against SARS-CoV-2 on Day 14, 28, 42, 56, 112, and 196 will be summarized by descriptive statistics and the 95% confidence interval for study vaccine group. The difference among vaccine groups will be analysed by ANOVA. Pair-wise comparisons of least square means from the ANOVA model will be presented. The reverse cumulative distribution plot will be provided to display the distribution of the GMI of neutralizing antibody against SARS-CoV-2 by time points for each vaccine group.

- Distribution of titers
  For ELISA, ACE2:RBD inhibition ELISA, and Neutralizing antibody titers, reverse cumulative distribution of titers will be displayed at Day 14,28, 42, 56, 112 and 196.
- Correlation between the immune response detected by ELISA and live virus neutralization test
  Pearson correlation coefficient will be used to measure the dependence of the immune response detected by anti-S1-RBD ELISA and live virus neutralization test, and the dependence of the immune response detected by qNeuAb ELISA (for research use only) and live virus neutralization test. Correlation coefficient between ELISA (anti-S1-RBD ELISA or qNeuAb ELISA) and live virus neutralization test will be presented by each matched time points and overall. In addition, the consistency of the immune response detected by ELISA (anti-S1-RBD ELISA or qNeuAb ELISA) and live virus neutralization test will be analysed by linear regression with the result of ELISA (anti-S1-RBD ELISA or qNeuAb ELISA) as dependent variable and the value of live virus neutralization test as independent variable. The scatter plot with the linear relationship and its 95% confidence interval and 95% prediction interval will be drawn.
- Positive rate and level of antigen-specific interferon-gamma (IFN-γ) measured by ELISpot on Day 7, 28, 35, and 196.
  Positive rate and level of antigen-specific interferon-gamma (IFN-γ) in each visit and each group is summarized by descriptive statistics and the 95% confidence interval for study vaccine group. Fisher exact test will be used to compare the positive rate among vaccine groups. The difference among vaccine groups in level of antigen-specific IFN-γ will be analysed by ANOVA.

- $CD4^+$ and $CD8^+$ T cell responses using intracellular cytokine staining and flow cytometry on Day 7, 28, 35, and 196.
  The specific memory $CD4^+$ T cells and memory $CD8^+$ T cell responses of each group and each time point will be counted, and the percentage of cells in which at least one of the three indexes is positive. The level of cell response will be expressed by the percentage of positive cells, and summarized by descriptive statistics. Count the positive rate of cell reaction in each group, expressed by the number and percentage, and calculate the 95% confidence interval of the positive rate.
  ANOVA will be used to compare the reaction level among vaccine groups and Fisher exact test will be used to compare the positive rate among vaccine groups.

10.6 Interim Analysis

Prior to the interim analysis, two administrative analyses will be performed on available safety and immunogenicity data collected during the 14 days after each vaccination; Administrative

Analysis #1 will include Group A, B and C data for Days 0-28; Administrative Analysis #2 will include safety and immunogenicity data for Days 28-42.

The final interim analysis will include data from the preceding administrative analyses (#1 and #2) as well as all new data through Day 56. This analysis, regarding to safety and immunogenicity of A, B and C groups, will be implemented after subjects with two vaccinations in A group, B group and C group have completed Visit 9 (Day 56), which is 28 days after 2nd vaccination. Interim analysis population will be the subjects in groups A, B and C who had completed two vaccinations. For the evaluation of safety and immunogenicity, the following endpoints will be included in the interim report.

- Occurrence of adverse reaction within 7 days after vaccination
- Occurrence of adverse events (AEs) till Day 56
- Occurrence of serious adverse events (SAEs) till Day 56
- GMT of antigen-specific antibody (Anti-S1-RBD) on Day 14, 28, 42 and 56
- Seroconversion rate (SCR) of antigen-specific antibody on Day 14, 28, 42 and 56
- Geometric mean fold increase of antigen-specific antibody on Day 14, 28, 42 and 56
- GMT of neutralizing antibody against SARS-CoV-2 on Day 14, 28, 42 and 56
- SCR of neutralizing antibody against SARS-CoV-2 on Day 14, 28, 42 and 56
- Geometric mean fold increase of neutralizing antibody against SARS-CoV-2 on Day 14, 28, 42 and 56
- Correlation between the immune response detected by ELISA and live virus neutralization test
- Positive rate and level of antigen-specific interferon-gamma (IFN-γ) measured by ELISpot on Day 7, 28, and 35
- $CD4^+$ and $CD8^+$ T cell responses using intracellular cytokine staining and flow cytometry on Day 7, 28, and 35

10.7 Handling of Missing Data

All available data will be displayed and utilized in data analysis. No imputation will be considered for the missing observations.

10.8 Determination of Sample Size

No formal statistical sample size and power computations are performed since the objectives of the study are to assess the safety and immunogenicity of the study vaccine. The sample size of each vaccine dose group is 20. In this study, twenty subjects will be involved in each vaccine dose group. A total of 60 subjects will be recruited.

10.9 Protocol Deviations

Protocol deviations will be categorized into important and non-important items, and definitions will be illustrated in the protocol deviation handling plan (PDHD). Events that beyond the PDHD will discuss with sponsor to determine the categorization.

11 QUALITY ASSURANCE AND QUALITY CONTROL

11.1 Audit and Inspection

Study centers and study documentation may be subject to Quality Assurance audit during the course of the study by the sponsor or its nominated representative. In addition, inspections may be conducted by regulatory authorities at their discretion.

11.2 Monitoring

Data for each subject will be recorded on an eCRF. Data collection must be completed for each subject who signs an ICF and is administered study agent.

In accordance with current good clinical practice (cGCP) and International Council for Harmonisation (ICH) guidelines, the study monitor will carry out source document verification at regular intervals to ensure that the data collected in the eCRF are accurate and reliable.

The investigator must permit the monitor, the IEC/IRB, the sponsor’s internal auditors, and representatives from regulatory authorities direct access to all study-related documents and pertinent hospital or medical records for confirmation of data contained within the eCRFs.

11.3 Data Management and Coding

The sponsor and/or the appointed representative(s) will be responsible for activities associated with the data management of this study. This will include setting up a relevant database and data transfer mechanisms, along with appropriate validation of data and resolution of queries. Data generated within this clinical study will be handled according to the relevant standard operating procedures of the data management and biostatistics departments of the sponsor and/or the appointed representative(s).

Study centers will complete the eCRF. Data entered into the eCRF must be verifiable against source documents at the study center. Data to be recorded directly on the eCRF will be identified and the eCRF will be considered the source document. Any changes to the data entered into the data capture system will be compliant to FDA CFR 21 Part 11.

Medical coding will use Medical Dictionary for Regulatory Activities for AEs.

Missing or inconsistent data will be queried to the investigator for clarification. Subsequent modifications to the database will be documented.

12 RECORDS AND SUPPLIES

12.1 Drug Accountability

On receipt of the study agent (including rescue medication, if relevant), the investigator (or designee) will conduct an inventory of the supplies and verify that study agent supplies are received intact and in the correct amounts before completing a supplies receipt. The investigator will retain a copy of this receipt at the study center and return the original receipt to the study monitor. The monitor may check the study supplies at each study center at any time during the study.

It is the responsibility of the study monitor to ensure that the investigator (or designee) has correctly documented the amount of the study agent received, dispensed, and returned on the dispensing log that will be provided. A full drug accountability log will be maintained at the study center at all times. The study monitor will arrange collection of unused study agent returned by the subject. The study monitor will also perform an inventory of study agent at the close-out visit to the study center. All discrepancies must be accounted for and documented.

12.2 Financing and Insurance

Financing and insurance of this study will be outlined in a separate agreement between the contract research organization and the sponsor.

13 ETHICS

13.1 Independent Ethics Committee or Institutional Review Board

Before initiation of the study at each study center, the protocol, the ICF, other written material given to the subjects, and any other relevant study documentation will be submitted to the appropriate IEC/IRB. Written approval of the study and all relevant study information must be obtained before the study center can be initiated or the study agent is released to the investigator. Any necessary extensions or renewals of IEC/IRB approval must be obtained for changes to the study such as amendments to the protocol, the ICF or other study documentation. The written approval of the IEC/IRB together with the approved ICF must be filed in the study files.

The investigator will report promptly to the IEC/IRB any new information that may adversely affect the safety of the subjects or the conduct of the study. The investigator will submit written summaries of the study status to the IEC/IRB as required. On completion of the study, the IEC/IRB will be notified that the study has ended.

13.2 Regulatory Authorities

Relevant study documentation will be submitted to the regulatory authorities of the participating countries, according to local/national requirements, for review and approval before the beginning of the study. On completion of the study, the regulatory authorities will be notified that the study has ended.

13.3 Ethical Conduct of the Study

The investigator(s) and all parties involved in this study should conduct the study in adherence to the ethical principles based on the Declaration of Helsinki, cGCP, ICH guidelines, and the applicable national and local laws and regulatory requirements.

13.4 Informed Consent

The process of obtaining informed consent must be in accordance with applicable regulatory requirement(s) and must adhere to cGCP.

The investigator is responsible for ensuring that no subject undergoes any study related examination or activity before that subject has given written informed consent to participate in the study.

The investigator or designated personnel will inform the subject of the objectives, methods, anticipated benefits and potential risks and inconveniences of the study. The subject should be given every opportunity to ask for clarification of any points s/he does not understand and, if necessary, ask for more information. At the end of the interview, the subject will be given ample time to consider the study. Subjects will be required to sign and date the ICF. After signatures are obtained, the ICF will be kept and archived by the investigator in the investigator's study file. A signed and dated copy of the subject ICF will be provided to the subject or their authorized representative.

It should be emphasized that the subject may refuse to enter the study or to withdraw from the study at any time, without consequences for their further care or penalty or loss of benefits to which the subject is otherwise entitled. Subjects who refuse to give or who withdraw written informed consent should not be included or continue in the study.

If new information becomes available that may be relevant to the subject’s willingness to continue participation in the study, a new ICF will be approved by the IEC(s)/IRB(s) (and regulatory authorities, if required). The study subjects will be informed about this new information and reconsent will be obtained.

13.5 Subject Confidentiality

Monitors, auditors, and other authorized agents of the sponsor and/or its designee, the IEC(s)/IRB(s) approving this research, and the United States (US) FDA, as well as that of any other applicable agency(ies), will be granted direct access to the study subjects’ original medical records for verification of clinical study procedures and/or data, without violating the confidentiality of the subjects to the extent permitted by the law and regulations. In any presentations of the results of this study or in publications, the subjects’ identity will remain confidential.

All personal data collected and processed for the purposes of this study should be managed by the investigator and his/her staff with adequate precautions to ensure confidentiality of those data, applicable to national and/or local laws and regulations on personal data protection.

14 REPORTING AND PUBLICATION, INCLUDING ARCHIVING

Essential documents are those documents that individually and collectively permit evaluation of the study and quality of the data produced. After completion of the study (end of study defined as the date of the last visit of the last subject), all documents and data relating to the study will be kept in an orderly manner by the investigator in a secure study file. This file will be available for inspection by the sponsor or its representatives. Essential documents should be retained for 2 years after the final marketing approval in an ICH region or for at least 2 years since the discontinuation of clinical development of the investigational product. It is the responsibility of the sponsor to inform the study center when these documents no longer need to be retained. The investigator must contact the sponsor before destroying any study related documentation. In addition, all subject medical records and other source documentation will be kept for the maximum time permitted by the hospital, institution, or medical practice.

The sponsor must review and approve any results of the study or abstracts for professional meetings prepared by the investigator(s). Published data must not compromise the objectives of the study. Data from individual study centers in multicenter studies must not be published separately.

15 REFERENCES

1. Hui DS, I Azhar E, Madani TA, et al. The continuing 2019-nCoV epidemic threat of novel coronaviruses to global health - The latest 2019 novel coronavirus outbreak in Wuhan, China. Int J Infect Dis. 2020;91:264-266.
2. Zhu N, Zhang D, Wang W, et al. A Novel Coronavirus from Patients with Pneumonia in China, 2019. N Engl J Med. 2020;382(8):727-733.
3. Guo YR, Cao QD, Hong ZS, et al. The origin, transmission and clinical therapies on coronavirus disease 2019 (COVID-19) outbreak - an update on the status. Mil Med Res. 2020;7(1):11. Published 2020 Mar 13.
4. Phan T. Novel coronavirus: From discovery to clinical diagnostics. Infect Genet Evol. 2020;79:104211.
5. Hoffmann M, Kleine-Weber H, Schroeder S, et al. SARS-CoV-2 Cell Entry Depends on ACE2 and TMPRSS2 and Is Blocked by a Clinically Proven Protease Inhibitor. Cell. 2020;181(2):271-280.e8.
6. Huang C, Wang Y, Li X, et al. Clinical features of patients infected with 2019 novel coronavirus in Wuhan, China [published correction appears in Lancet. 2020 Jan 30;:]. Lancet. 2020;395(10223):497-506.
7. Remy V, Largeron N, Quilici S, Carroll S. The Economic Value of Vaccination: Why Prevention Is Wealth. Value Health. 2014;17(7):A450.
8. Takashima Y, Osaki M, Ishimaru Y, Yamaguchi H, Harada A. Artificial molecular clamp: a novel device for synthetic polymerases. Angew Chem Int Ed Engl. 2011;50(33):7524-7528.
9. Liu H, Su D, Zhang J, et al. Improvement of Pharmacokinetic Profile of TRAIL via Trimer-Tag Enhances its Antitumor Activity in vivo. Sci Rep. 2017;7(1):8953.
10. Jiang S. Don't rush to deploy COVID-19 vaccines and drugs without sufficient safety guarantees. Nature. 2020;579(7799):321.
11. Peeples L. News Feature: Avoiding pitfalls in the pursuit of a COVID-19 vaccine. Proc Natl Acad Sci U S A. 2020;117(15):8218-8221.
12. Grifoni A, Weiskopf D, Ramirez SI, et al. Targets of T Cell Responses to SARS-CoV-2 Coronavirus in Humans with COVID-19 Disease and Unexposed Individuals. Cell. 2020;S0092-8674(20)30610-3.
13. Barbara L., Miriam S. Priority List of Adverse Events of Special Interest: COVID-19. Safety Platform for Emergency Vaccines. V1.1, 05 March 2020

Appedix 2

中國醫藥大學附設醫院
CHINA MEDICAL UNIVERSITY HOSPITAL
台中市北區育德路2號
2 Yude Road, Taichung, 40447, Taiwan (R.O.C.)
TEL : 886-4-22052121

中國醫藥大學暨附設醫院研究倫理委員會

Tel: 886-4-22052121 ext: 1925　Fax: 886-4-2207-1478　台中市北區育德路 2 號

計畫修正案通過證明書

計畫名稱：一個評估 UB-612 於成年健康受試者的安全性、耐受性與免疫原性的開放性、第一期臨床試驗

計畫編號/本會編號：V-122 / CMUH109-REC1-125(AR-9)

計畫主持人：兒童感染科黃高彬主治醫師

試驗機構：中國醫藥大學附設醫院

原計畫通過日期：2020 年 08 月 31 日至 2021 年 08 月 30 日

修正案通過日期：2021 年 05 月 14 日至 2021 年 08 月 30 日

計畫書：Version: 2.5, Date: 03 May, 2021

上述計畫之修正案已於 2021 年 05 月 14 日經中國醫藥大學暨附設醫院研究倫理委員會第一審查委員會簡易審查通過。本委員會的運作符合優良臨床試驗準則及國內相關法令。請在持續審查必須進行前二個月向本會檢送完整之期中報告。

此計畫任何部分若經更改，必須在執行前重新提交本會審查及核准。此外，計畫主持人必須依時通報嚴重不良事件及涉及受試者或其他人風險的非預期問題。

主任委員 傅茂祖

中國醫藥大學暨附設醫院 研究倫理委員會 台中市北區育德路2號

中 華 民 國 一 一 〇 年 五 月 十 八 日

CMUH

The Committee is organized and operates in accordance with ICH6 GCP regulations and guideline.
本委員會組織與運作皆遵守 ICH6 GCP 規定

中國醫藥大學附設醫院
CHINA MEDICAL UNIVERSITY HOSPITAL
台中市北區育德路2號
2 Yude Road, Taichung, 40447, Taiwan (R.O.C.)
TEL : 886-4-22052121

Research Ethics Committee
China Medical University & Hospital, Taichung, Taiwan

Tel: 886-4-22052121 ext: 1925 Fax: 886-4-2207-1478

Clinical Trial/Human Research Approval

Amendment Review

Date：May 18, 2021

Protocol Title：A phase I, open-label study to evaluate the safety, tolerability, and immunogenicity of UB-612 vaccine in healthy adult volunteers

Protocol No. / CMUH REC No.：V-122 / CMUH109-REC1-125(AR-9)

Name of Principal Investigator：Kao-Pin Hwang (Attending Physician, Pediatric Infectious Diseases)

Name of Institution：China Medical University Hospital

Valid Date of Original Research Project：From Aug. 31, 2020 to Aug. 30, 2021

Valid Date of Amended Research Project：From May 14, 2021 to Aug. 30, 2021

Protocol：Version: 2.5, Date: 03 May, 2021

This is to certify that the above referenced amended research project has been expedited approved by the Research Ethics Committee (REC) I of the China Medical University and Hospital on May 14, 2021. The REC is organized under, and operates in accordance with, the Good Clinical Practices guidelines and the governmental laws and regulations. Please submit a completed progress report at least two months before the time at which continuing review must occur.

All the amendments to the research project should be re-submitted and approved by the REC BEFORE implementation. Also, the principal investigator is required to report all serious adverse events and unanticipated problems involving risks to the subjects or others on time.

Martin M-T Fuh MD, DMSci.
Chairman, Research Ethics Committee I
China Medical University & Hospital

CHINA MEDICAL UNIVERSITY & HOSPITAL
The Research Ethics Committee
No. 2 Yude Road, Taichung, Taiwan, R.O.C.

中國醫藥大學附設醫院
CHINA MEDICAL UNIVERSITY HOSPITAL
台中市北區育德路2號
2 Yude Road, Taichung, 40447, Taiwan (R.O.C.)
TEL：886-4-22052121

中國醫藥大學暨附設醫院研究倫理委員會
Tel: 886-4-22052121 ext: 1925　Fax: 886-4-2207-1478　台中市北區育德路 2 號

計畫修正案通過證明書

計畫名稱：一個評估 UB-612 於成年健康受試者的安全性、耐受性與免疫原性的開放性、第一期臨床試驗

計畫編號/本會編號：V-122 / CMUH109-REC1-125(AR-7)

計畫主持人：兒童感染科黃高彬主治醫師

試驗機構：中國醫藥大學附設醫院

原計畫通過日期：2020 年 08 月 31 日至 2021 年 08 月 30 日

修正案通過日期：2021 年 03 月 15 日至 2021 年 08 月 30 日

受試者同意書(A 組)：Version 2.7, Date: Mar. 12, 2021

受試者同意書(B 組)：Version 2.7, Date: Mar. 12, 2021

受試者同意書(C 組)：Version 2.7, Date: Mar. 12, 2021

上述計畫之修正案已於 2021 年 03 月 15 日經中國醫藥大學暨附設醫院研究倫理委員會第一審查委員會簡易審查通過。本委員會的運作符合優良臨床試驗準則及國內相關法令。請在持續審查必須進行前二個月向本會檢送完整之期中報告。

此計畫任何部分若經更改，必須在執行前重新提交本會審查及核准。此外，計畫主持人必須依時通報嚴重不良事件及涉及受試者或其他人風險的非預期問題。

主任委員 傅茂祖

中國醫藥大學暨附設醫院 研究倫理委員會 台中市北區育德路2號

中　華　民　國　一　一　〇　年　三　月　十　六　日

The Committee is organized and operates in accordance with ICH6 GCP regulations and guideline.
本委員會組織與運作皆遵守 ICH6 GCP 規定

中國醫藥大學附設醫院
CHINA MEDICAL UNIVERSITY HOSPITAL
台中市北區育德路2號
2 Yude Road, Taichung, 40447, Taiwan (R.O.C.)
TEL : 886-4-22052121

Research Ethics Committee

China Medical University & Hospital, Taichung, Taiwan

Tel: 886-4-22052121 ext: 1925 Fax: 886-4-2207-1478

Clinical Trial/Human Research Approval

Amendment Review

Date：Mar. 16, 2021

Protocol Title：A phase I, open-label study to evaluate the safety, tolerability, and immunogenicity of UB-612 vaccine in healthy adult volunteers

Protocol No. / CMUH REC No.：V-122 / CMUH109-REC1-125(AR-7)

Name of Principal Investigator：Kao-Pin Hwang (Attending Physician, Pediatric Infectious Diseases)

Name of Institution：China Medical University Hospital

Valid Date of Original Research Project：From Aug. 31, 2020 to Aug. 30, 2021

Valid Date of Amended Research Project：From Mar. 15, 2021 to Aug. 30, 2021

Informed Consent Form (A group)：Version 2.7, Date: Mar. 12, 2021

Informed Consent Form (B group)：Version 2.7, Date: Mar. 12, 2021

Informed Consent Form (C group)：Version 2.7, Date: Mar. 12, 2021

This is to certify that the above referenced amended research project has been expedited approved by the Research Ethics Committee (REC) I of the China Medical University and Hospital on Mar. 15, 2021. The REC is organized under, and operates in accordance with, the Good Clinical Practices guidelines and the governmental laws and regulations. Please submit a completed progress report at least two months before the time at which continuing review must occur.

All the amendments to the research project should be re-submitted and approved by the REC BEFORE implementation. Also, the principal investigator is required to report all serious adverse events and unanticipated problems involving risks to the subjects or others on time.

Martin M-T Fuh MD, DMSci.
Chairman, Research Ethics Committee I
China Medical University & Hospital

CHINA MEDICAL UNIVERSITY & HOSPITAL
The Research Ethics Committee
No. 2 Yude Road, Taichung, Taiwan, R.O.C.

中國醫藥大學暨附設醫院研究倫理委員會
審查結果通知書

本會編號	CMUH109-REC1-125(AR-7)	送審文件類型	修正案
計畫主持人	感染控制小組黃高彬主治醫師	計畫經費來源	廠商合作計畫
計畫名稱	一個評估 UB-612 於成年健康受試者的安全性、耐受性與免疫原性的開放性、第一期臨床試驗		
審查流程	簡易審查		
審查結果	通過		
初審審查意見	一、請重新簽署受試者同意書。		

中國醫藥大學暨附設醫院
研究倫理委員會
台中市北區育德路2號

Appendix 3

中國醫藥大學暨附設醫院
受試者同意書（A組）

黃高彬
2021.3.12

您被邀請參與此研究。此同意書主要是提供您本研究之相關資訊，以便您決定是否參加本研究。計畫主持人或其指定之研究人員會為您說明研究內容並回答您的疑問。您可以提出任何和此研究有關的問題，在您的問題尚未獲得滿意的答覆之前，請不要簽署此同意書。如果您願意參與本研究，此文件將視為您的同意紀錄。即使在您同意後，您可以隨時退出本研究不需任何理由。

計畫名稱<br>中文：一個評估 UB-612 於成年健康受試者的安全性、耐受性與免疫原性的開放性、第一期臨床試驗<br>英文：A phase I, open-label study to evaluate the safety, tolerability, and immunogenicity of UB-612 vaccine in healthy adult volunteers	
執行單位：中國醫藥大學附設醫院感染科、家庭醫學科	委託單位/藥廠：聯亞生技開發股份有限公司<br>研究經費來源：聯亞生技開發股份有限公司<br>受託研究機構：晉加股份有限公司
計畫主持人：黃高彬	職稱：主治醫師
協同主持人：林文元	職稱：主治醫師
協同主持人：林伯昌	職稱：主治醫師
緊急聯絡人：黃高彬	電話：0975-681-950
受試者姓名：<br>性別：<br>身分證字號：<br>通訊地址：	病歷號碼：<br>出生日期：<br>聯絡電話：
法定代理人或有同意權人之姓名：<br>性別：<br>身分證字號：<br>通訊地址：	與受試者關係：<br>出生日期：<br>聯絡電話：

(一)試驗簡介：

1. 本品/技術資料：

新型冠狀病毒(SARS-CoV-2)於2019年12月起造成中國湖北省武漢市發現多起病毒性肺炎群聚，隨後於2020年1月底台灣出現第一起境外移入確診個案。此疾病在全球擴散，世界衛生組織宣布將此疫情為「國際關注公共衛生緊急事件」。截至2020年6月

為止，此疫情已經造成全球824萬人感染，44.6萬人死亡，但是目前在全世界尚未研究出有效的疫苗。

UB-612疫苗為聯亞生技開發股份有限公司所開發新型冠狀病毒預防性疫苗，疫苗含病毒棘狀融合蛋白和胜肽片段，可產生高親和力抗體與新型冠狀病毒結合，並誘發細胞免疫反應，進而達到預防新型冠狀病毒的感染。

2. 本品上市狀況：

本品首次應用於人體試驗，尚未在我國上市。

(二)試驗目的：

- 本試驗之主要試驗目的為評估UB-612疫苗於成年健康受試者之安全性與耐受性。
- 本試驗之次要試驗目的為評估UB-612疫苗於成年健康受試者之免疫原性。

(三) 試驗之主要納入與排除條件：

執行本研究計畫的醫師或相關研究人員將會與您討論有關參加本研究的必要條件。請您配合必須誠實告知我們您過去的健康情形，若您有不符合參加本研究的情況，將不能參加本研究計畫。

1. 納入條件(參加本試驗/研究的條件):

(1) 您為篩選訪視時20-55歲之間健康男性或未懷孕的女性受試者。

(2) 您為具生育能力的女性與男性應於首次接種疫苗至最後一次疫苗後3個月同意進行有效的避孕方式。可接受的有效避孕方式包括:

a. 男性或女性以手術方法絕育、植入式避孕、或子宮避孕器。

b. 注射避孕、避孕藥、避孕貼片、避孕環加上一種屏障避孕法*。

c. 兩種合併使用的屏障避孕法*。

*有效的屏障避孕法為避孕隔膜、男性或女性保險套、避孕海綿或殺精劑(含可殺精化學物質的藥膏或凝膠)。

(3) 您能理解受試者同意書內容的說明與可能的風險，提供簽名的受試者同意書。

(4) 您能夠理解與遵從本試驗程序與能參與每次訪視。

(5) 您的B型肝炎表面抗原檢測、C型肝炎RNA檢測，與人類免疫缺乏病毒抗體檢測呈現陰性。

(6) 以聯亞(UBI)酵素結合免疫吸附分析法進行新型冠狀病毒血清抗體(免疫球蛋白G(IgG))檢測，您的採檢結果呈現陰性。

(7) 以新型冠狀病毒反轉錄聚合酶連鎖反應檢驗鼻咽或喉嚨拭子檢體，您的採檢結果為陰性。

(8) 您的耳溫≤ 38.0°C。

(9) 您的身體質量指數為18-30 kg/m^2。

(10) 經試驗主持人判斷，您的常規血液檢查、血液生化學檢查或其他實驗室數值為正常，或非臨床顯著。

(11) 經試驗主持人判斷，您在篩選訪視時為健康的受試者，健康情況依醫療病史、身體檢查、心電圖、生命徵象(血壓、心跳、體溫、呼吸速率)、臨床實驗檢測(血液常規與生化學檢測)判斷。

2. 排除條件(若您有下列任一情況，您將無法參加本試驗/研究):

(1) 您有接種疫苗後需要醫療介入的過敏性休克、蕁麻疹或其他顯著不良反應的病史。

(2) 您為已懷孕女性或懷孕檢測為陽性的女性。

(3) 您為正在哺乳的女性，或計畫從接種第一劑疫苗至最後一劑疫苗後 60 天哺乳的女性。

(4) 您在接種第一劑疫苗前3天內，經試驗主持人判斷，患有任何急性疾病。

(5) 您有血液學疾病、腎臟疾病、內分泌疾病、肺臟疾病、腸胃道疾病、心血管疾病、肝臟疾病、精神學疾病、神經疾病、或過敏疾病(包含藥物過敏的) 的臨床顯著證據或病史。

(6) 已知您有嚴重急性呼吸道症候群(SARS)或中東呼吸症候群(MERS)冠狀病毒感染史。

(7) 已知您曾暴露於新型冠狀病毒，或曾接受預防新型冠狀病毒、中東呼吸症候群冠狀病毒、嚴重急性呼吸道症候群的試驗產品。

(8) 您在簽署受試者同意書前12周內參與其他的臨床試驗。

(9) 您的醫療狀況可能增加罹患新型冠狀病毒感染後，成為重症的風險，例如慢性腎臟病、慢性阻塞性肺病、嚴重的心臟疾病(例如心臟衰竭、冠狀動脈心臟病或心肌病變)，或有重大未能控制的慢性疾病，例如氣喘、糖尿病、胸腔疾病。

(10) 您有先天性或後天性血管性水腫。

(11) 您有免疫缺乏/失調疾病，無論是否由基因缺陷、免疫缺乏症或免疫抑制療法所造成。

(12) 您患有血小板異常或其他凝血異常可能造成注射之禁忌症。

(13) 您在接種第一劑疫苗前6個月長期接受(≥14天連續使用)免疫抑制劑、皮質類固醇(相當於一天使用≥ 20 mg強的松(prednisone))或細胞毒性治療。

(14) 您在接種第一劑疫苗前4個月接受免疫球蛋白和/或任何血液製劑的治療。

(15) 您在接種第一劑疫苗前1個月接種過減毒性疫苗、核酸 (mRNA或DNA)或載體疫苗，或預計在接種第二劑疫苗前1個月接種此類疫苗。

(16) 您在接種第一劑疫苗前14天接種過次單元疫苗或去活化疫苗，或預計在接種第二劑疫苗前14天接種此類疫苗。

(17) 您正在接受抗肺結核治療，或有肺結核病史。

(18) 您為酒精成癮及物質濫用者。

(19) 除了皮膚基底細胞癌和子宮頸原位癌，您在篩選訪視前5年有惡性腫瘤病史。

(20) 經試驗主持人判斷，您有任何醫療疾病或狀況，可能會影響試驗結果或參與試驗可能會對受試者引發額外風險。

(四) 試驗方法及相關檢驗：

這是一個第一期、開放性、劑量遞增試驗，評估3種遞增劑量UB-612新型冠狀病毒疫苗接種於健康成年受試者的安全性、耐受性與免疫原性。試驗共納入三組:

本試驗所使用的UB-612疫苗*劑量與預計納入受試者人數如下:

A組	施打兩劑UB-612疫苗(含10微克融合蛋白與胜肽，0.5 毫升)	20位受試者
B組	施打兩劑UB-612疫苗(含30微克融合蛋白與胜肽，0.5 毫升)	20位受試者
C組	施打兩劑UB-612疫苗(含100微克融合蛋白與胜肽，0.5 毫升)	20位受試者

*: UB-612疫苗，含Adju-Phos® 與CpG寡核苷酸佐劑

總計本試驗將納入60名受試者，整個試驗的納入方式為在A組的前20位受試者將納入試驗，施打兩劑UB-612疫苗(含10微克融合蛋白與胜肽)，施打間隔為28天(第0天和第28天)。若經過資料及安全性監測委員會決定A組接種劑量沒有任何的安全疑慮，可以納入B組受試者。若經過資料及安全性監測委員會決定B組接種劑量沒有任何的安全疑慮，可以納入C組受試者。各組將在最後一位受試者完成第9次訪視(第56天)時進行期中分析。

您目前的組別為A組，前6位受試者為前哨組，接受第一劑疫苗後，於第3天和第7天回診。若經過資料及安全性監測委員會決定前6位受試者在7天內沒有發生與試驗疫苗相關第三級以上的不良反應或嚴重不良事件，將可納入隨後的14位受試者。當A組所有受試者完成第4次訪視(第7天)，資料及安全性監測委員會在審查過A組的安全性資料後，將決定B組是否可以開始納入受試者。

這個試驗將有11次訪視，包括第1次訪視(篩選期，第-14天至第-1天)，第2次訪視(第0天，基礎值，第一次接種疫苗)，第3次訪視(第3天，只有前6位受試者需要回診)，第4次訪視(第7天)，第5次訪視(第14天)，第6次訪視(第28天，第二次接種疫苗)，第7次訪視(第35天)，第8次訪視(第42天)，第9次訪視(第56天)，第10次訪視(第112天，第二次接種疫苗後3個月)，第11次訪視(第196天，第二次接種疫苗後6個月)。

注意事項

1. 如果您同意參加本試驗，研究人員會請您簽署本份受試者同意書，並確認您符合參加本試驗的條件。

2. 從您參與試驗的當天開始，每次訪視都將有合格的試驗人員執行試驗流程與聯繫。
3. 於試驗期間，您不論任何理由提前退出試驗，試驗研究人員都將安排您完成最後一次的訪視之所有試驗項目。您有權利拒絕此項安排，您的決定不會引起任何影響日後醫師對您的醫療照護。

試驗步驟

篩選訪視(第一次訪視，第-14 天~第-1 天)

在試驗醫師或試驗研究人員為您提供足夠的試驗資訊，並確保您有充分的時間考慮以及詢問任何問題後，您願意讓您參與本試驗，並由您簽署本受試者同意書。在確認您已完成受試者同意書簽署並且您也保有一份副本後，試驗醫師或試驗研究人員將會進行以下試驗程序：

(1) 記錄您簽署受試者同意書的日期
(2) 為您指定一組受試者篩選編號
(3) 確認您是否符合本試驗的納入排除條件
(4) 收集您的個人基本資料（例如生日、年齡及性別）
(5) 記錄您的醫療/用藥病史
(6) 進行身體檢查，包括身高體重
(7) 確認生命徵象
(8) 進行心電圖檢查
(9) 收集血液檢體(共 12 毫升)，進行下列檢測:
- 血清懷孕檢測（具有生育能力女性）
- 常規血液檢測
- 血液生化學檢測
- B 型肝炎表面抗原, C 型肝炎 RNA, 人類免疫缺乏病毒抗體檢測
- 新型冠狀病毒血清抗體(IgG)檢測

(10) 收集鼻咽/喉嚨拭子檢體，進行新型冠狀病毒核酸檢測
(11) 收集併用藥物/治療

進行人類免疫缺乏病毒(HIV)檢測，若檢測結果呈現陽性，依法將通報主管機關。

☐同意　簽名：__________日期：__________________

第二次訪視(第 0 天)-基礎值，第一次接種疫苗

將會進行以下試驗程序：

(1) 確認您是否符合本試驗的納入排除條件
(2) 為您指定一組受試者編號
(3) 若有任何接種疫苗前的醫療狀況，應紀錄為醫療病史
(4) 進行身體檢查
(5) 確認生命徵象
(6) 進行尿液懷孕檢測（具生育能力的女性）
(7) 接種疫苗前，收集血液檢體(共 40 毫升)，進行下列檢測:
- 體液免疫原性
- 細胞免疫原性

(8) 進行第一次疫苗接種。接種疫苗後，每組前 6 位受試者應留在試驗地點至少 2-4 小時，隨後的 14 位受試者應留在試驗地點至少 60 分鐘，每 30 分鐘監測一次生命徵象和不良事件。
(9) 接種疫苗後一小時內測量心電圖
(10) 將發給您日誌卡，並詳細地指導您如何填寫日誌卡
(11) 收集併用藥物/治療

第三次訪視(第 3±1 天)-僅前 6 個受試者需要回診

將會進行以下試驗程序：

(1) 確認您接種疫苗後的安全性
(2) 進行身體檢查
(3) 確認生命徵象
(4) 收集併用藥物/治療
(5) 記錄上一次訪視至此次訪視之間的不良事件或嚴重不良事件

第四次訪視(第 7±1 天)

將會進行以下試驗程序：

(1) 進行身體檢查
(2) 確認生命徵象
(3) 收集血液檢體(共 42 毫升) ，進行下列檢測:
- 常規血液檢測
- 血液生化學檢測
- 細胞免疫原性

(4) 收回日誌卡

(5) 收集併用藥物/治療

(6) 記錄上一次訪視至此次訪視之間的不良事件或嚴重不良事件

第五次訪視(第 14±3 天)

將會進行以下試驗程序：

(1) 進行身體檢查

(2) 確認生命徵象

(3) 收集血液檢體(共 5 毫升) ，進行下列檢測:
- 體液免疫原性

(4) 收集併用藥物/治療

(5) 記錄上一次訪視至此次訪視之間的不良事件或嚴重不良事件

第六次訪視(第 28±3 天) -第二次接種疫苗

將會進行以下試驗程序：

(1) 進行第二次接種評估

(2) 進行身體檢查

(3) 確認生命徵象

(4) 進行尿液懷孕檢測（具生育能力的女性）

(5) 接種疫苗前，收集血液檢體(共 47 毫升)，進行下列檢測:
- 常規血液檢測
- 血液生化學檢測
- 體液免疫原性
- 細胞免疫原性

(6) 進行第二次疫苗接種。接種疫苗後，應留在試驗地點 60 分鐘，每 30 分鐘監測一次生命徵象和不良事件。

(7) 接種疫苗後一小時內測量心電圖

(8) 將發給您日誌卡，並詳細地指導您如何填寫日誌卡。

(9) 收集併用藥物/治療

(10) 記錄上一次訪視至此次訪視之間的不良事件或嚴重不良事件

第七次訪視(第 35±3 天)

將會進行以下試驗程序：

(1) 進行身體檢查

(2) 確認生命徵象

(3) 收集血液檢體(共 42 毫升) ，進行下列檢測:

- 常規血液檢測
- 血液生化學檢測
- 細胞免疫原性

(4) 收回日誌卡。

(5) 收集併用藥物/治療

(6) 記錄上一次訪視至此次訪視之間的不良事件或嚴重不良事件

第八次訪視(第 42±3 天)

將會進行以下試驗程序：

(1) 進行身體檢查

(2) 確認生命徵象

(3) 收集血液檢體(共 5 毫升) ，進行下列檢測:

- 體液免疫原性

(4) 收集併用藥物/治療

(5) 記錄上一次訪視至此次訪視之間的不良事件或嚴重不良事件

第九次訪視(第 56±3 天)

將會進行以下試驗程序：

(1) 進行身體檢查

(2) 確認生命徵象

(3) 進行心電圖檢查

(4) 收集血液檢體(共 5 毫升) ，進行下列檢測:

- 體液免疫原性

(5) 收集併用藥物/治療

(6)若您同意，將施打一劑流感疫苗

□同意　簽名：__________日期：______________

(7) 進行新型冠狀病毒感染監測

*新型冠狀病毒感染監測: 在試驗第 56 天後至試驗結束，若您有發燒、咳嗽、或其他呼吸道症狀應立即至試驗地點回診。試驗團隊將會以鼻咽/喉嚨拭子收集檢體，並進行電腦斷層掃描或其他影像學檢查，以確認您是否有新型冠狀病毒感染。若您為新型冠狀病毒感染的患者，也會觀察您是否有疾病增強的反應(請見(五)可能產生之副作用、發生率及處理方法，關於疾病增強的說明)

(8) 記錄上一次訪視至此次訪視之間的不良事件或嚴重不良事件

第十次訪視(第 112±5 天) –第 3 個月追蹤

將會進行以下試驗程序：

(1) 進行身體檢查

(2) 確認生命徵象

(3) 收集血液檢體(共 5 毫升) ，進行下列檢測:

- 體液免疫原性

(4) 收集併用藥物/治療

(5) 進行新型冠狀病毒感染監測

(6) 記錄上一次訪視至此次訪視之間的不良事件或嚴重不良事件

第十一次訪視(第 196±15 天)-第 6 個月追蹤

(1) 進行身體檢查

(2) 確認生命徵象

(3) 尿液懷孕檢測（具生育能力的女性）

(4) 收集血液檢體(共 40 毫升) ，進行下列檢測:

- 體液免疫原性
- 細胞免疫原性

(5) 收集併用藥物/治療

(6) 進行新型冠狀病毒感染監測

(7) 記錄上一次訪視至此次訪視之間的不良事件或嚴重不良事件

受試者之檢體(含其衍生物)之保存、使用與再利用：

1. 檢體及剩餘檢體之保存與使用

(1) 檢體(含其衍生物)之保存與使用

為研究所需，我們所蒐集您的檢體，將依本研究計畫使用，檢體將保存於聯亞生技開發(股)公司及聯合生物製藥(股)公司，直至 20 年保存期限屆滿，我們將依法銷毀。為了保護您的個人隱私，我們將以一個試驗編號來代替您的名字及相關個人資料，以確認您的檢體及與相關資料受到完整保密。如果您對檢體的使用有疑慮，或您有任何想要銷毀檢體的需求，請立即與我們聯絡(聯絡人：黃高彬醫師電話：0975-681-950），我們即會將您的檢體銷毀。您也可以聯繫中國醫藥大學暨附設醫院研究倫理委員會(電話：04-22052121 轉 1925、1926)，以協助您解決檢體在研究使用上的任何爭議。

(2) 剩餘檢體(含其衍生物)之再利用

中國醫藥大學暨附設醫院
受試者同意書（A組）

您的生物檢體將會以專屬號碼進行編碼並在聯亞生技開發股份有限公司(試驗委託者)的控管下儲存最長20年。

所有新的研究計畫都要再經由中國醫藥大學暨附設醫院研究倫理委員會審議通過，倫理審查委員會若認定新的研究超出您同意的範圍，將要求我們重新得到您的同意。

是否同意剩餘檢體保留提供未來新型冠狀病毒感染研究之用，並授權中國醫藥大學暨附設醫院研究倫理委員會審議是否需要再取得您的同意(擇一)

☐ 不同意保存我的剩餘檢體，試驗結束後請銷毀

☐ 同意以非去連結之方式保存我的剩餘檢體，逾越原同意使用範圍時，需再次得到我的同意才可使用我的檢體進行新的研究

您的剩餘檢體將運送至以下實驗室進行新型冠狀病毒變異株的檢驗:

實驗室名稱	機構地址
UTMB	University of Texas Medical Branch 301 University Boulevard Keiller Building, Room 2.150 Galveston, Texas, USA(美國)
Virology (Tropical Medicine Institute, University of São Paulo)	University of São Paulo, Brazil Rua Dr Enéas de Carvalho Aguiar 470 , CEP 05403-000(巴西)
Viral and Rickettsial Disease Laboratory (VRDL)	850 Marina Bay Parkway Richmond, CA 94804, USA(美國)

請問您是否同意?

☐ 同意 ☐ 不同意　簽名：__________日期：______________

2. 檢體及剩餘檢體之部分類型(檢體類型可依計畫書內容自行增減)

(1) 一般生化、血液檢驗檢體、鼻咽/喉嚨拭子檢體

在試驗期間，會將您的檢體送往聯亞生技開發股份有限公司(試驗委託者)委託的中央實驗室中國醫藥大學暨附設醫院分析，此機構地址為台中市北區育德路2號，中央實驗室會在分析後立即將分析結果提供給試驗中心，若有剩餘的檢體，將會儲存直到檢驗結果複驗完畢即銷毀，不會長期儲存。

(2) 抗體/細胞免疫試驗

在試驗期間，會將您的檢體送往聯亞生技開發(股)公司(試驗委託者)分析實驗室及聯

合生物製藥(股)公司生物分析實驗室進行處置、處理與進一步分析。聯亞生技開發(股)公司機構地址為新竹縣竹北市生醫路二段6-1號5樓，聯合生物製藥(股)公司機構地址為新竹縣竹北市生醫路二段12號1樓。完成試驗後，若有剩餘檢體，將儲存於聯亞生技開發(股)公司及聯合生物製藥(股)公司，直到至少完成臨床試驗報告為止，最長將保存20年。

(3) 中和試驗(neutralization test, NT)

在試驗期間，會將您的檢體送往聯亞生技開發股份有限公司(試驗委託者)委託的中央實驗室中央研究院進行處置、處理與進一步分析。此機構地址為台北市南港區研究院路二段128號。完成試驗後，若有剩餘檢體，將儲存直到至少完成臨床試驗報告為止，最長將保存20年。

(4) 遺傳學檢體

在試驗期間，若發生嚴重不良反應或特定不良反應，您的DNA檢體將用於HLA分型檢驗，會將您的檢體送往聯亞生技開發股份有限公司(試驗委託者)委託的中央實驗室有勁基因分析，此機構地址為新北市樹林區復興路376-5號，中央實驗室會在分析後立即將分析結果提供給試驗中心，若有剩餘的檢體，將會儲存直到檢驗結果複驗完畢即銷毀，不會長期儲存。

(五)可能產生之副作用、發生率及處理方法：

1. 與試驗藥物相關的風險（本試驗疫苗的副作用）：

冠狀病毒疫苗的開發

過去針對與SARS-CoV-2病毒相同屬於人類冠狀病毒的SARS-CoV(嚴重急性呼吸綜合症冠狀病毒(SARS冠狀病毒))的疫苗研究發現，接種過SARS-CoV疫苗的小鼠在暴露到SARS-CoV後會發生過度免疫反應而產生病變，因此不得不停止這種疫苗的開發。所以，成功的人類冠狀病毒疫苗不只要產生可以抑制病毒的免疫反應，更要避免過度免疫產生的副作用。

疫苗相關的風險

本試驗疫苗首次使用於人體，因此尚未有人體安全性資料。

接種疫苗可能會出現注射部位的不良反應(例如疼痛、硬化腫脹、皮疹發紅、過敏反應、蜂窩性組織炎)，或全身性不良反應(例如發燒、腹瀉、疲倦、噁心/嘔吐、厭食、咽喉痛、頭痛、咳嗽、關節痛、非注射部位疼痛、非注射部位搔癢、皮膚和黏膜異常、急性過敏反應、昏厥、急性支氣管痙攣、呼吸困難)。

疾病增強(disease enhancement) 的風險

SARS-CoV-2候選疫苗也可能會有引發疾病增強(disease enhancement) 的風險，包括抗體依賴性增強(antibody-dependent enhancement)或疫苗相關聯的增強的呼吸道疾病(vaccine-associated enhanced respiratory disease)。在先前研發SARS疫苗時，在數個SARS-CoV動物攻毒試驗(包括鼠類、雪貂、猴類)當中，有發現疾病增強的現象。疾病增強反應的免疫病理現象包括TH2偏向及嗜酸性白血球的肺部浸潤。但是目前已發表的新型冠狀病毒肺炎疫苗研究，仍尚未發現類似的疾病增強現象。

本試驗疫苗於數個藥理試驗呈現不一致的TH1/TH2偏向，雖未一致偏向TH2，仍已規劃並正在執行動物攻毒模型試驗，以進一步排除疾病增強風險。依據文獻指出，組成複雜或容易引起非中和抗體之抗原，如不活化病毒或整片段之蛋白(包含S蛋白與N蛋白)，與易引起偏向Th2免疫反應之佐劑成分，如鋁製佐劑，皆較有可能引起疾病增強。本試驗疫苗的主要抗原為S蛋白上之RBD 區域，已有多篇文獻指出，針對S-RBD設計之SARS與MERS疫苗從未於試驗動物模型上引發疾病增強現象。本試驗疫苗雖使用易引起偏向Th2免疫反應之佐劑，但由動物實驗證實，也同時引起偏向Th1之反應，因此發生疾病增強應屬低風險。且已於多種動物模型中證實，能誘發高效價之中和抗體，於細胞培養中亦能有效抑制新冠病毒感染。

建議您在有效疫苗上市前或本試驗疫苗的產品資訊有進一步更新前，盡量避免暴露於可能感染病毒的環境。研究團隊將會在試驗中執行相關安全性監測。若有任何關於本試驗疫苗與疾病增強風險相關之任何最新資訊，將即時更新並提供給您。

疫苗佐劑相關的風險

本試驗疫苗所使用的佐劑含Adju-Phos®，是屬於一種磷酸鋁類的佐劑。磷酸鋁類佐劑已經使用超過半個世紀，具有相當的安全性。由於此類佐劑可誘導免疫反應，因此可能會造成局部發炎反應，例如在注射部位產生輕微而短暫的疼痛、發紅以及腫脹。

2. 與試驗/研究過程相關的風險：

抽血

本試驗需要抽血檢驗。抽血可能引起一些不適和瘀血。整個試驗期間6個月，共需抽血 243毫升。若需要進行HLA分型檢驗，將額外抽血2毫升。

鼻咽/喉嚨拭子採檢

將以鼻咽/喉嚨拭子採檢您的該部位的分泌物，可能會引起噁吐感或咽喉不適。研究人員將做好相關防護措施。

心電圖(ECG)

心電圖檢查可建立心臟電流活動的影像。在進行本程序時，您將需要靜躺幾分鐘，讓電極貼附在您的胸前、手臂及腿上。在您皮膚上貼上與取下電極時，可能會引起一些不適。若您為有任何胸毛的男性，可能需要剃除放置電極部位的毛髮。

在接種疫苗過程中，可能會出現一些尚未在已完成試驗中發現的副作用。一般而言，接種某一新疫苗總是會有一定的風險，但是計畫主持人會採取一切措施預防風險的發生。**計畫主持人鼓勵您報告您遇到的任何不適。**

(六) 其他替代療法及說明：

您不是非參加不可，若不參加研究，由於目前尚未有疫苗可用來預防新型冠狀病毒感染，因此預防措施與其他呼吸道感染相同，包括：勤洗手、減少觸摸眼口鼻、注意咳嗽禮節、妥善處理口鼻分泌物等，避免出入公共場所，並不要接觸野生動物。

如果您對於本試驗疫苗有任何的疑問，您可以提出來向您的試驗醫師討論。

(七) 試驗預期效益：

依據臨床前試驗結果，預期本試驗疫苗對您可能可以產生抗體，預防新型冠狀病毒感染，但因每個人體質不同也有可能不會產生療效，故參加本試驗可能不會有直接的好處。

但是您參加本試驗，可協助我們獲得更多資訊，以瞭解UB-612疫苗的安全性與免疫力。

(八)試驗進行中受試者之禁忌、限制與應配合之事項：

<u>禁止使用的藥物</u>

以下藥物請勿在試驗期間使用：

- 禁止使用免疫抑制劑、皮質類固醇或細胞毒性治療
- 禁止使用免疫球蛋白和/或任何血液製劑
- 整個試驗期間使用試驗產品(包括藥物或疫苗)
- 第56天後可接種其他已經上市的疫苗，但應與最近一次訪視間隔至少一個月

<u>允許使用的藥物</u>

若您的藥物或治療必須常規使用，經試驗醫師判斷不會影響本試驗疫苗的免疫原性、臨床療效與安全性，則可以正常使用。您有任何關於在試驗期間可允許使用何種藥物或治療的問題，請詢問您的試驗醫師。

<u>懷孕或母乳哺乳的風險</u>

目前未知本試驗疫苗對於未出生胎兒的影響，因此：

- 您為具生育能力的女性受試者 (除非手術絕育或停經)，或您為男性受試者應於接種疫苗至最後一次疫苗後 3 個月同意進行有效的避孕方式，同意進行有效的避孕方式(例如子宮內節育器、荷爾蒙療法或避孕套)。
- 若您為具生育能力的女性，將請您進行懷孕檢測，結果必須為陰性，方可參與試驗。
- 若您為懷孕的女性，將被告知不可參與本試驗。
- 若您在試驗期間懷孕，請盡速通知試驗人員，並且停止施打本疫苗。
- 基於安全性考量，若您為女性受試者而在試驗期間懷孕，或您為男性受試者而您的性伴侶在試驗期間懷孕(將請您的懷孕性伴侶需簽署另外一份同意書)，您與您的胎兒將會被追蹤監測至分娩，除非另有醫學指示。

您應向您的配偶或性伴侶告知您有參與此試驗與相關風險:

簽名：___________日期：____________________

(九)機密性：

中國醫藥大學附設醫院將依法把任何可辨識您的身分之紀錄與您的個人隱私資料視為機密來處理，不會公開。研究人員將以一個研究代碼代表您的身分，此代碼不會顯示您的姓名、國民身分證統一編號、住址等可識別資料。如果發表試驗/研究結果，您的身分仍將保密。您亦瞭解若簽署同意書即同意您的原始醫療紀錄可直接受監測者、稽核者、研究倫理委員會及主管機關檢閱，以確保臨床試驗/研究過程與數據符合相關法律及法規要求，上述人員並承諾絕不違反您的身分之機密性。除了上述機構依法有權檢視外，我們會小心維護您的隱私。由於試驗藥物可能同時申請美國臨床試驗，依美國藥品管理規定，試驗結果將公佈於公開的臨床試驗資訊網站：Clinicaltrials.gov (美國)，但您的個人資料仍將保密，該網站只會有試驗之結果摘要，您可以在任何時候搜尋該網站。

在試驗/研究期間，依據計畫類型與您所授權的內容，我們將會蒐集與您有關的病歷資料、醫療紀錄、量表、問卷等資料與資訊，並以一個編號來代替您的名字及相關個人資料。前述資料若為紙本型式，將會與本同意書分開存放於研究機構之上鎖櫃中；若為電子方式儲存或建檔以供統計與分析之用，將會存放於設有密碼與適當防毒軟體之專屬電腦內。這些研究資料與資訊將會保存至藥品於我國上市後至少兩年，若試驗疫苗終止研發則保存至試驗正式停止後至少二年，至多將保存至疫苗上市後或試驗正式停止後二年。

上述資料與資訊若傳輸至國外分析與統計，您仍會獲得與本國法規相符之保障，計畫主持人與相關團隊將盡力確保您的個人資料獲得妥善保護。

<需進行 HIV 檢測適用>

因本試驗需排除感染人類免疫缺乏病毒(HIV)者，您將接受人類免疫缺乏病毒(HIV)檢測，若檢驗結果為陰性始得參與本試驗，若檢驗結果為陽性(包含偽陽性)，本試驗將提供後續就醫轉介或諮詢，且經確認後需依法(人類免疫缺乏病毒傳染防治及感染者權益保障條例)通報主管機關。

(十)損害補償與保險：

1. 如依本研究所訂臨床試驗計畫，因發生不良反應造成損害，由聯亞生技開發股份有限公司負補償責任。但本受試者同意書上所記載之可預期不良反應，不予補償。
2. 如依本研究所訂臨床試驗計畫，因而發生不良反應或損害，贊助廠商將依法負責損害賠償責任。本醫院願意提供專業醫療照顧及醫療諮詢。您不必負擔治療不良反應或損害之必要醫療費用。
3. 除前二項補償及醫療照顧外，本研究不提供其他形式之補償。若您不願意接受這樣的風險，請勿參加試驗。
4. 您不會因為簽署本同意書，而喪失在法律上的任何權利。
5. 本研究有投保責任保險。

（十一）受試者權利：

1. 試驗過程中，與您的健康或是疾病有關，可能影響您繼續接受臨床試驗意願的任何重大發現，都將即時提供給您。
2. 如果您在試驗過程中對試驗工作性質產生疑問，對身為患者之權利有意見或懷疑因參與研究而受害時，可與本院之研究倫理委員會聯絡請求諮詢，其電話號碼為：04-22052121轉1925、1926。
3. 為進行試驗工作，您必須接受黃高彬醫師的照顧。如果您現在或於試驗期間有任何問題或狀況，請不必客氣，可與在中國醫藥大學附設醫院兒童感染科的黃高彬醫師聯絡（24小時聯繫電話：0975-681-950）。
4. 參加試驗研究計畫之補助：本計畫將在每次訪視提供交通費3000元給您，整個試驗預計給予您30000~33000元。
5. 本同意書一式2份，醫師已將同意書副本交給您，並已完整說明本研究之性質與目的。醫師已回答您有關藥品與研究的問題。

（十二）試驗之退出與中止：

您可自由決定是否參加本試驗；試驗過程中也可隨時撤銷同意，退出試驗，不需

任何理由，且不會引起任何不愉快或影響其日後醫師對您的醫療照顧。

計畫主持人或贊助廠商亦可能於必要時中止該試驗之進行。

（十三）簽名：

1. 計畫主持人、或協同主持人已詳細解釋有關本研究計畫中上述研究方法的性質與目的，及可能產生的危險與利益。

   計畫主持人/協同主持人簽名：____________日期：________年____月____日

2. 受試者已詳細瞭解上述研究方法及其所可能產生的危險與利益，有關本試驗計畫的疑問，業經試驗主持人詳細予以解釋。本人同意接受為臨床試驗計畫的自願受試者。

   受試者簽名：____________________________日期：________年____月____日

   法定代理人簽名：_______________________日期：________年____月____日

   * 受試者為無行為能力(未滿七歲之未成年人者或禁治產人)，由法定代理人為之；禁治產人，由監護人擔任其法定代理人。

   * 受試者為限制行為人者(滿七歲以上之未成年人)，應得法定代理人之同意。

   有同意權人簽名：_______________________日期：________年____月____日

   * 受試者雖非無行為能力或限制行為能力者，但因意識混亂或有精神與智能障礙，而無法進行有效溝通和判斷時，由有同意權之人為之。前項有同意權人為配偶及直系親屬。

3. 見證人

   見證人簽名：_____________________________日期：________年____月____日

   身分證字號：　　　　　　　　　　　聯絡電話：

   通訊地址：

   * 受試者、法定代理人或有同意權之人皆無法閱讀時，應由見證人在場參與所有有關受試者同意之討論。並確定受試者、法定代理人或有同意權之人之同意完全出於其自由意願後，應於受試者同意書簽名並載明日期。試驗相關人員不得為見證人。

流程表:

訪視	1	2	3	4	5	6	7	8	9	10	11/ET
**檢測項目**	**篩選**	**第一次接種**			**第二次接種**					**第 3 個月追蹤 [g]**	**第 6 個月追蹤 [h]**
**天數**	**-14~-1**	**0**	**3[c] ±1 天**	**7 ±1 天**	**14 ±3 天**	**28 ±3 天**	**35 ±3 天**	**42 ±3 天**	**56 ±3 天**	**112 ±5 天**	**196 ±15 天**
臨床檢測											
獲得受試者同意書	X										
納入/排除條件	X	X									
第二次接種評估						X					
基本資料	X										
醫療病史	X	X									
身體檢查 [a]	X	X	X	X	X	X	X	X	X	X	X
生命徵象	X	X	X	X	X	X	X	X	X	X	X
心電圖	X	X [i]				X [i]			X		
**實驗室數值檢測**											
B 型肝炎表面抗原, C 型肝炎 RNA, 人類免疫缺乏病毒抗體檢測	X										
核酸檢測(鼻咽/喉嚨拭子)	X										
新型冠狀病毒血清抗體(IgG)檢測	X										
**實驗室檢測 (安全性)**											
血液常規檢測	X			X		X	X				
血液生化學檢測	X[d]			X		X	X				
懷孕(HCG/尿液)檢測 [b]	X	X				X					X

訪視	1	2	3	4	5	6	7	8	9	10	11/ET
檢測項目	篩選	第一次接種				第二次接種				第 3 個月追蹤 [g]	第 6 個月追蹤 [h]
天數	-14~-1	0	3[c] ±1 天	7 ±1 天	14 ±3 天	28 ±3 天	35 ±3 天	42 ±3 天	56 ±3 天	112 ±5 天	196 ±15 天
**實驗室檢測(免疫原性)**											
體液免疫原性		X			X	X		X	X	X	X
細胞免疫原性		X		X		X	X				X
**試驗步驟**											
疫苗接種		X				X					
發給日誌卡 [e]		X				X					
回收日誌卡 [e]			X	X			X				
不良事件/嚴重不良事件		X	X	X	X	X	X	X	X	X	X
新型冠種病毒感染監測									X	X	X
併用藥物	X	X	X	X	X	X	X	X	X	X[f]	X[f]

a: 身高與體重僅在第一次訪視測量。
b: 將執行懷孕檢測(對於有懷孕能力的女性於第 0, 28, 196 天使用血清 β-HCG 懷孕檢測和尿液懷孕檢測)，但是已絕經或以手術絕育的女性將不作此檢測。若尿液檢測為陽性，應以血清懷孕檢測再次確認。以血清懷孕檢測替代尿液檢測將不視為試驗偏差。
c: 僅針對每組的前 6 名受試者
d: 僅在第一次訪視執行 HbA1c 檢測。
e: 若使用電子化日誌卡，將沒有發給和收回日誌卡程序。
f: 只記錄嚴重不良事件的使用藥物。
g: 第二次接種疫苗後 3 個月
h: 第二次接種疫苗後 6 個月
i: 應在接種疫苗後一小時內測量心電圖。
ET: 提早退出試驗

黃高彬
2021.3.12

中國醫藥大學暨附設醫院
受試者同意書（B組）

您被邀請參與此研究。此同意書主要是提供您本研究之相關資訊，以便您決定是否參加本研究。計畫主持人或其指定之研究人員會為您說明研究內容並回答您的疑問。您可以提出任何和此研究有關的問題，在您的問題尚未獲得滿意的答覆之前，請不要簽署此同意書。如果您願意參與本研究，此文件將視為您的同意紀錄。即使在您同意後，您可以隨時退出本研究不需任何理由。

計畫名稱

中文：一個評估 UB-612 於成年健康受試者的安全性、耐受性與免疫原性的開放性、第一期臨床試驗

英文：A phase I, open-label study to evaluate the safety, tolerability, and immunogenicity of UB-612 vaccine in healthy adult volunteers

執行單位：中國醫藥大學附設醫院感染科、家庭醫學科	委託單位/藥廠：聯亞生技開發股份有限公司<br>研究經費來源：聯亞生技開發股份有限公司<br>受託研究機構：晉加股份有限公司
計畫主持人：黃高彬	職稱：主治醫師
協同主持人：林文元	職稱：主治醫師
協同主持人：林伯昌	職稱：主治醫師
緊急聯絡人：黃高彬	電話：0975-681-950
受試者姓名：<br>性別：<br>身分證字號：<br>通訊地址：	病歷號碼：<br>出生日期：<br>聯絡電話：
法定代理人或有同意權人之姓名：<br>性別：<br>身分證字號：<br>通訊地址：	與受試者關係：<br>出生日期：<br>聯絡電話：

(一)試驗簡介：

1. 本品/技術資料：

新型冠狀病毒(SARS-CoV-2)於2019年12月起造成中國湖北省武漢市發現多起病毒性肺炎群聚，隨後於2020年1月底台灣出現第一起境外移入確診個案。此疾病在全球擴散，世界衛生組織宣布將此疫情為「國際關注公共衛生緊急事件」。截至2020年6月

為止，此疫情已經造成全球824萬人感染，44.6萬人死亡，但是目前在全世界尚未研究出有效的疫苗。

UB-612疫苗為聯亞生技開發股份有限公司所開發新型冠狀病毒預防性疫苗，疫苗含病毒棘狀融合蛋白和胜肽片段，可產生高親和力抗體與新型冠狀病毒結合，並誘發細胞免疫反應，進而達到預防新型冠狀病毒的感染。

2. 本品上市狀況：

本品首次應用於人體試驗，尚未在我國上市。

（二）試驗目的：

- 本試驗之主要試驗目的為評估UB-612疫苗於成年健康受試者之安全性與耐受性。
- 本試驗之次要試驗目的為評估UB-612疫苗於成年健康受試者之免疫原性。

(三) 試驗之主要納入與排除條件：

執行本研究計畫的醫師或相關研究人員將會與您討論有關參加本研究的必要條件。請您配合必須誠實告知我們您過去的健康情形，若您有不符合參加本研究的情況，將不能參加本研究計畫。

1. 納入條件(參加本試驗/研究的條件):

(1) 您為篩選訪視時20-55歲之間健康男性或未懷孕的女性受試者。

(2) 您為具生育能力的女性與男性應於首次接種疫苗至最後一次疫苗後3個月同意進行有效的避孕方式。可接受的有效避孕方式包括:

a. 男性或女性以手術方法絕育、植入式避孕、或子宮避孕器。

b. 注射避孕、避孕藥、避孕貼片、避孕環加上一種屏障避孕法*。

c. 兩種合併使用的屏障避孕法*。

*有效的屏障避孕法為避孕隔膜、男性或女性保險套、避孕海綿或殺精劑(含可殺精化學物質的藥膏或凝膠)。

(3) 您能理解受試者同意書內容的說明與可能的風險，提供簽名的受試者同意書。

(4) 您能夠理解與遵從本試驗程序與能參與每次訪視。

(5) 您的B型肝炎表面抗原檢測、C型肝炎RNA檢測，與人類免疫缺乏病毒抗體檢測呈現陰性。

(6) 以聯亞(UBI)酵素結合免疫吸附分析法進行新型冠狀病毒血清抗體(免疫球蛋白G(IgG))檢測，您的採檢結果呈現陰性。。

(7) 以新型冠狀病毒反轉錄聚合酶連鎖反應檢驗鼻咽或喉嚨拭子檢體，您的採檢結果為陰性。

(8) 您的耳溫≤ 38.0°C。

(9) 您的身體質量指數為18-30 kg/m^2。
(10) 經試驗主持人判斷，您的常規血液檢查、血液生化學檢查或其他實驗室數值為正常，或非臨床顯著。
(11) 經試驗主持人判斷，您在篩選訪視時為健康的受試者，健康情況依醫療病史、身體檢查、心電圖、生命徵象(血壓、心跳、體溫、呼吸速率)、臨床實驗檢測(血液常規與生化學檢測)判斷。

2. 排除條件(若您有下列任一情況，您將無法參加本試驗/研究):
(1) 您有接種疫苗後需要醫療介入的過敏性休克、蕁麻疹或其他顯著不良反應的病史。
(2) 您為已懷孕女性或懷孕檢測為陽性的女性。
(3) 您為正在哺乳的女性，或計畫從接種第一劑疫苗至最後一劑疫苗後 60 天哺乳的女性。
(4) 您在接種第一劑疫苗前3天內，經試驗主持人判斷，患有任何急性疾病。
(5) 您有血液學疾病、腎臟疾病、內分泌疾病、肺臟疾病、腸胃道疾病、心血管疾病、肝臟疾病、精神學疾病、神經疾病、或過敏疾病(包含藥物過敏的) 的臨床顯著證據或病史。
(6) 已知您有嚴重急性呼吸道症候群(SARS)或中東呼吸症候群(MERS)冠狀病毒感染史。
(7) 已知您曾暴露於新型冠狀病毒，或曾接受預防新型冠狀病毒、中東呼吸症候群冠狀病毒、嚴重急性呼吸道症候群的試驗產品。
(8) 您在簽署受試者同意書前12周內參與其他的臨床試驗。
(9) 您的醫療狀況可能增加罹患新型冠狀病毒感染後，成為重症的風險，例如慢性腎臟病、慢性阻塞性肺病、嚴重的心臟疾病(例如心臟衰竭、冠狀動脈心臟病或心肌病變)，或有重大未能控制的慢性疾病，例如氣喘、糖尿病、胸腔疾病。
(10) 您有先天性或後天性血管性水腫。
(11) 您有免疫缺乏/失調疾病，無論是否由基因缺陷、免疫缺乏症或免疫抑制療法所造成。
(12) 您患有血小板異常或其他凝血異常可能造成注射之禁忌症。
(13) 您在接種第一劑疫苗前6個月長期接受(≥14天連續使用)免疫抑制劑、皮質類固醇(相當於一天使用≥ 20 mg強的松(prednisone))或細胞毒性治療。
(14) 您在接種第一劑疫苗前4個月接受免疫球蛋白和/或任何血液製劑的治療。
(15) 您在接種第一劑疫苗前1個月接種過減毒性疫苗、核酸 (mRNA或DNA)或載體疫苗，或預計在接種第二劑疫苗前1個月接種此類疫苗。
(16) 您在接種第一劑疫苗前14天接種過次單元疫苗或去活化疫苗，或預計在接種第二劑疫苗前14天接種此類疫苗。
(17) 您正在接受抗肺結核治療，或有肺結核病史。

(18) 您為酒精成癮及物質濫用者。

(19) 除了皮膚基底細胞癌和子宮頸原位癌，您在篩選訪視前5年有惡性腫瘤病史。

(20) 經試驗主持人判斷，您有任何醫療疾病或狀況，可能會影響試驗結果或參與試驗可能會對受試者引發額外風險。

(四) 試驗方法及相關檢驗：

這是一個第一期、開放性、劑量遞增試驗，評估3種遞增劑量UB-612新型冠狀病毒疫苗接種於健康成年受試者的安全性、耐受性與免疫原性。試驗共納入三組:

本試驗所使用的UB-612疫苗*劑量與預計納入受試者人數如下:

A組	施打兩劑UB-612疫苗(含10微克融合蛋白與胜肽，0.5 毫升)	20位受試者
B組	施打兩劑UB-612疫苗(含30微克融合蛋白與胜肽，0.5 毫升)	20位受試者
C組	施打兩劑UB-612疫苗(含100微克融合蛋白與胜肽,0.5 毫升)	20位受試者

*: UB-612疫苗，含Adju-Phos® 與CpG寡核苷酸佐劑

總計本試驗將納入60名受試者，整個試驗的納入方式為在A組的前20位受試者將納入試驗，施打兩劑UB-612疫苗(含10微克融合蛋白與胜肽)，施打間隔為28天(第0天和第28天)。若經過資料及安全性監測委員會決定A組接種劑量沒有任何的安全疑慮，可以納入B組受試者。若經過資料及安全性監測委員會決定B組接種劑量沒有任何的安全疑慮，可以納入C組受試者。各組將在最後一位受試者完成第9次訪視(第56天)時進行期中分析。

您目前的組別為B組，前6位受試者為前哨組，接受第一劑疫苗後，於第3天和第7天回診。若經過資料及安全性監測委員會決定前6位受試者在7天內沒有發生與試驗疫苗相關第三級以上的不良反應或嚴重不良事件，將可納入隨後的14位受試者。當B組所有受試者完成第4次訪視(第7天)，資料及安全性監測委員會在審查過B組的安全性資料後，將決定C組是否可以開始納入受試者。

這個試驗將有11次訪視，包括第1次訪視(篩選期，第-14天至第-1天)，第2次訪視(第0天，基礎值，第一次接種疫苗)，第3次訪視(第3天，只有前6位受試者需要回診)，第4次訪視(第7天)，第5次訪視(第14天)，第6次訪視(第28天，第二次接種疫苗)，第7次訪視(第35天)，第8次訪視(第42天)，第9次訪視(第56天)，第10次訪視(第112天，第二次接種疫苗後3個月)，第11次訪視(第196天，第二次接種疫苗後6個月)。

<u>注意事項</u>

1. 如果您同意參加本試驗，研究人員會請您簽署本份受試者同意書，並確認您符合參加本試驗的條件。

2. 從您參與試驗的當天開始，每次訪視都將有合格的試驗人員執行試驗流程與聯繫。
3. 於試驗期間，您不論任何理由提前退出試驗，試驗研究人員都將安排您完成最後一次的訪視之所有試驗項目。您有權利拒絕此項安排，您的決定不會引起任何影響日後醫師對您的醫療照護。

試驗步驟

篩選訪視(第一次訪視，第-14 天~第-1 天)

在試驗醫師或試驗研究人員為您提供足夠的試驗資訊，並確保您有充分的時間考慮以及詢問任何問題後，您願意讓您參與本試驗，並由您簽署本受試者同意書。在確認您已完成受試者同意書簽署並且您也保有一份副本後，試驗醫師或試驗研究人員將會進行以下試驗程序：

(1) 記錄您簽署受試者同意書的日期
(2) 為您指定一組受試者篩選編號
(3) 確認您是否符合本試驗的納入排除條件
(4) 收集您的個人基本資料（例如生日、年齡及性別）
(5) 記錄您的醫療/用藥病史
(6) 進行身體檢查，包括身高體重
(7) 確認生命徵象
(8) 進行心電圖檢查
(9) 收集血液檢體(共 12 毫升)，進行下列檢測:
- 血清懷孕檢測（具有生育能力女性）
- 常規血液檢測
- 血液生化學檢測
- B 型肝炎表面抗原, C 型肝炎 RNA, 人類免疫缺乏病毒抗體檢測
- 新型冠狀病毒血清抗體(IgG)檢測

(10) 收集鼻咽/喉嚨拭子檢體，進行新型冠狀病毒核酸檢測
(11) 收集併用藥物/治療

進行人類免疫缺乏病毒(HIV)檢測，若檢測結果呈現陽性，依法將通報主管機關。

☐同意　簽名：__________日期：__________________

第二次訪視(第 0 天)-基礎值，第一次接種疫苗

將會進行以下試驗程序：

(1) 確認您是否符合本試驗的納入排除條件

(2) 為您指定一組受試者編號

(3) 若有任何接種疫苗前的醫療狀況，應紀錄為醫療病史

(4) 進行身體檢查

(5) 確認生命徵象

(6) 進行尿液懷孕檢測（具生育能力的女性）

(7) 接種疫苗前，收集血液檢體(共 40 毫升)，進行下列檢測:

- 體液免疫原性
- 細胞免疫原性

(8) 進行第一次疫苗接種。接種疫苗後，每組前 6 位受試者應留在試驗地點至少 2-4 小時，隨後的 14 位受試者應留在試驗地點至少 60 分鐘，每 30 分鐘監測一次生命徵象和不良事件。

(9) 接種疫苗後一小時內測量心電圖

(10) 將發給您日誌卡，並詳細地指導您如何填寫日誌卡。

(11) 收集併用藥物/治療

第三次訪視(第 3±1 天)-僅前 6 個受試者需要回診

將會進行以下試驗程序：

(1) 確認您接種疫苗後的安全性

(2) 進行身體檢查

(3) 確認生命徵象

(4) 收集併用藥物/治療

(5) 記錄上一次訪視至此次訪視之間的不良事件或嚴重不良事件

第四次訪視(第 7±1 天)

將會進行以下試驗程序：

(1) 進行身體檢查

(2) 確認生命徵象

(3) 收集血液檢體(共 42 毫升) ，進行下列檢測:

- 常規血液檢測
- 血液生化學檢測
- 細胞免疫原性

(4) 收回日誌卡
(5) 收集併用藥物/治療
(6) 記錄上一次訪視至此次訪視之間的不良事件或嚴重不良事件

第五次訪視(第 14±3 天)

將會進行以下試驗程序：

(1) 進行身體檢查
(2) 確認生命徵象
(3) 收集血液檢體(共 5 毫升) ，進行下列檢測:
- 體液免疫原性

(4) 收集併用藥物/治療
(5) 記錄上一次訪視至此次訪視之間的不良事件或嚴重不良事件

第六次訪視(第 28±3 天) -第二次接種疫苗

將會進行以下試驗程序：

(1) 進行第二次接種評估
(2) 進行身體檢查
(3) 確認生命徵象
(4) 進行尿液懷孕檢測（具生育能力的女性）
(5) 接種疫苗前，收集血液檢體(共 47 毫升)，進行下列檢測:
- 常規血液檢測
- 血液生化學檢測
- 體液免疫原性
- 細胞免疫原性

(6) 進行第二次疫苗接種。接種疫苗後，應留在試驗地點 60 分鐘，每 30 分鐘監測一次生命徵象和不良事件。
(7) 接種疫苗後一小時內測量心電圖
(8) 將發給您日誌卡，並詳細地指導您如何填寫日誌卡。
(9) 收集併用藥物/治療
(10) 記錄上一次訪視至此次訪視之間的不良事件或嚴重不良事件

第七次訪視(第 35±3 天)

將會進行以下試驗程序：

(1) 進行身體檢查
(2) 確認生命徵象

(3) 收集血液檢體(共 42 毫升) ，進行下列檢測:

- 常規血液檢測
- 血液生化學檢測
- 細胞免疫原性

(4) 收回日誌卡。

(5) 收集併用藥物/治療

(6) 記錄上一次訪視至此次訪視之間的不良事件或嚴重不良事件

第八次訪視(第 42±3 天)

將會進行以下試驗程序：

(1) 進行身體檢查

(2) 確認生命徵象

(3) 收集血液檢體(共 5 毫升) ，進行下列檢測:

- 體液免疫原性

(4) 收集併用藥物/治療

(5) 記錄上一次訪視至此次訪視之間的不良事件或嚴重不良事件

第九次訪視(第 56±3 天)

將會進行以下試驗程序：

(1) 進行身體檢查

(2) 確認生命徵象

(3) 進行心電圖檢查

(4) 收集血液檢體(共 5 毫升) ，進行下列檢測:

- 體液免疫原性

(5) 收集併用藥物/治療

(6)若您同意，將施打一劑流感疫苗

□同意　簽名：＿＿＿＿＿＿日期：＿＿＿＿＿＿＿

(7) 進行新型冠狀病毒感染監測

*新型冠狀病毒感染監測: 在試驗第 56 天後至試驗結束，若您有發燒、咳嗽、或其他呼吸道症狀應立即至試驗地點回診。試驗團隊將會以鼻咽/喉嚨拭子收集檢體，並進行電腦斷層掃描或其他影像學檢查，以確認您是否有新型冠狀病毒感染。若您為新型冠狀病毒感染的患者，也會觀察您是否有疾病增強的反應(請見(五)可能產生之副作用、發生率及處理方法，關於疾病增強的說明)

(8) 記錄上一次訪視至此次訪視之間的不良事件或嚴重不良事件

第十次訪視(第 112±5 天) –第 3 個月追蹤

將會進行以下試驗程序：

(1) 進行身體檢查
(2) 確認生命徵象
(3) 收集血液檢體(共 **5** 毫升) ，進行下列檢測:
- 體液免疫原性

(4) 收集併用藥物/治療
(5) 進行新型冠狀病毒感染監測
(6) 記錄上一次訪視至此次訪視之間的不良事件或嚴重不良事件

第十一次訪視(第 196±15 天)-第 6 個月追蹤

(1) 進行身體檢查
(2) 確認生命徵象
(3) 尿液懷孕檢測（具生育能力的女性）
(4) 收集血液檢體(共 40 毫升) ，進行下列檢測:
- 體液免疫原性
- 細胞免疫原性

(5) 收集併用藥物/治療
(6) 進行新型冠狀病毒感染監測
(7) 記錄上一次訪視至此次訪視之間的不良事件或嚴重不良事件

受試者之檢體(含其衍生物)之保存、使用與再利用：

1. 檢體及剩餘檢體之保存與使用

(1) 檢體(含其衍生物)之保存與使用

為研究所需，我們所蒐集您的檢體，將依本研究計畫使用，檢體將保存於聯亞生技開發(股)公司及聯合生物製藥(股)公司，直至 20 年保存期限屆滿，我們將依法銷毀。為了保護您的個人隱私，我們將以一個試驗編號來代替您的名字及相關個人資料，以確認您的檢體及與相關資料受到完整保密。如果您對檢體的使用有疑慮，或您有任何想要銷毀檢體的需求，請立即與我們聯絡(聯絡人：黃高彬醫師電話：0975-681-950)，我們即會將您的檢體銷毀。您也可以聯繫中國醫藥大學暨附設醫院研究倫理委員會(電話：04-22052121 轉 1925、1926)，以協助您解決檢體在研究使用上的任何爭議。

(2) 剩餘檢體(含其衍生物)之再利用

中國醫藥大學暨附設醫院
受試者同意書（B組）

您的生物檢體將會以專屬號碼進行編碼並在聯亞生技開發股份有限公司(試驗委託者)的控管下儲存最長20年。

所有新的研究計畫都要再經由中國醫藥大學暨附設醫院研究倫理委員會審議通過，倫理審查委員會若認定新的研究超出您同意的範圍，將要求我們重新得到您的同意。

是否同意剩餘檢體保留提供未來新型冠狀病毒感染研究之用，並授權中國醫藥大學暨附設醫院研究倫理委員會審議是否需要再取得您的同意(擇一)

☐ 不同意保存我的剩餘檢體，試驗結束後請銷毀

☐ 同意以非去連結之方式保存我的剩餘檢體，逾越原同意使用範圍時，需再次得到我的同意才可使用我的檢體進行新的研究

您的剩餘檢體將運送至以下實驗室進行新型冠狀病毒變異株的檢驗:

實驗室名稱	機構地址
**UTMB**	**University of Texas Medical Branch 301 University Boulevard Keiller Building, Room 2.150 Galveston, Texas, USA(美國)**
**Virology (Tropical Medicine Institute, University of São Paulo)**	**University of São Paulo, Brazil Rua Dr Enéas de Carvalho Aguiar 470 , CEP 05403-000(巴西)**
**Viral and Rickettsial Disease Laboratory (VRDL)**	**850 Marina Bay Parkway Richmond, CA 94804, USA(美國)**

請問您是否同意?

☐ 同意 ☐ 不同意　簽名：__________日期：_____________

2. 檢體及剩餘檢體之部分類型(檢體類型可依計畫書內容自行增減)

(1) 一般生化、血液檢驗檢體、鼻咽/喉嚨拭子檢體

在試驗期間，會將您的檢體送往聯亞生技開發股份有限公司(試驗委託者)委託的中央實驗室中國醫藥大學暨附設醫院分析，此機構地址為台中市北區育德路2號，中央實驗室會在分析後立即將分析結果提供給試驗中心，若有剩餘的檢體，將會**儲存直到**檢驗結果複驗完畢即銷毀，不會長期儲存。

(2) 抗體/細胞免疫試驗

試驗期間，會將您的檢體送往聯亞生技開發(股)公司(試驗委託者)分析實驗室及聯合

生物製藥(股)公司生物分析實驗室進行處置、處理與進一步分析。聯亞生技開發(股)公司機構地址為新竹縣竹北市生醫路二段6-1號5樓，聯合生物製藥(股)公司機構地址為新竹縣竹北市生醫路二段12號1樓。完成試驗後，若有剩餘檢體，將儲存於聯亞生技開發(股)公司及聯合生物製藥(股)公司，直到至少完成臨床試驗報告為止，最長將保存20年。

(3) 中和試驗(neutralization test, NT)

在試驗期間，會將您的檢體送往聯亞生技開發股份有限公司(試驗委託者)委託的中央實驗室中央研究院進行處置、處理與進一步分析。此機構地址為台北市南港區研究院路二段128號。完成試驗後，若有剩餘檢體，將儲存直到至少完成臨床試驗報告為止，最長將保存20年。

(4) 遺傳學檢體

在試驗期間，若發生嚴重不良反應或特定不良反應，您的DNA檢體將用於HLA分型檢驗，會將您的檢體送往聯亞生技開發股份有限公司(試驗委託者)委託的中央實驗室有勁基因分析，此機構地址為新北市樹林區復興路376-5號，中央實驗室會在分析後立即將分析結果提供給試驗中心，若有剩餘的檢體，將會儲存直到檢驗結果複驗完畢即銷毀，不會長期儲存。

(五)可能產生之副作用、發生率及處理方法：

1. 與試驗藥物相關的風險（本試驗疫苗的副作用）：

冠狀病毒疫苗的開發

過去針對與SARS-CoV-2病毒相同屬於人類冠狀病毒的SARS-CoV(嚴重急性呼吸綜合症冠狀病毒(SARS冠狀病毒))的疫苗研究發現，接種過SARS-CoV疫苗的小鼠在暴露到SARS-CoV後會發生過度免疫反應而產生病變，因此不得不停止這種疫苗的開發。所以，成功的人類冠狀病毒疫苗不只要產生可以抑制病毒的免疫反應，更要避免過度免疫產生的副作用。

疫苗相關的風險

本試驗疫苗首次使用於人體，因此尚未有人體安全性資料。

接種疫苗可能會出現注射部位的不良反應(例如疼痛、硬化腫脹、皮疹發紅、過敏反應、蜂窩性組織炎)，或全身性不良反應(例如發燒、腹瀉、疲倦、噁心/嘔吐、厭食、咽喉痛、頭痛、咳嗽、關節痛、非注射部位疼痛、非注射部位搔癢、皮膚和黏膜異常、急性過敏反應、昏厥、急性支氣管痙攣、呼吸困難)。

<u>疾病增強(disease enhancement) 的風險</u>

SARS-CoV-2候選疫苗也可能會有引發疾病增強(disease enhancement) 的風險，包括抗體依賴性增強(antibody-dependent enhancement)或疫苗相關聯的增強的呼吸道疾病(vaccine-associated enhanced respiratory disease)。在先前研發SARS疫苗時，在數個SARS-CoV動物攻毒試驗(包括鼠類、雪貂、猴類)當中，有發現疾病增強的現象。疾病增強反應的免疫病理現象包括TH2偏向及嗜酸性白血球的肺部浸潤。但是目前已發表的新型冠狀病毒肺炎疫苗研究，仍尚未發現類似的疾病增強現象。

本試驗疫苗於數個藥理試驗呈現不一致的TH1/TH2偏向，雖未一致偏向TH2，仍已規劃並正在執行動物攻毒模型試驗，以進一步排除疾病增強風險。依據文獻指出，組成複雜或容易引起非中和抗體之抗原，如不活化病毒或整片段之蛋白(包含S蛋白與N蛋白)，與易引起偏向Th2免疫反應之佐劑成分，如鋁製佐劑，皆較有可能引起疾病增強。本試驗疫苗的主要抗原為S蛋白上之RBD 區域，已有多篇文獻指出，針對S-RBD設計之SARS與MERS疫苗從未於試驗動物模型上引發疾病增強現象。本試驗疫苗雖使用易引起偏向Th2免疫反應之佐劑，但由動物實驗證實，也同時引起偏向Th1之反應，因此發生疾病增強應屬低風險。且已於多種動物模型中證實，能誘發高效價之中和抗體，於細胞培養中亦能有效抑制新冠病毒感染。

建議您在有效疫苗上市前或本試驗疫苗的產品資訊有進一步更新前，盡量避免暴露於可能感染病毒的環境。研究團隊將會在試驗中執行相關安全性監測。若有任何關於本試驗疫苗與疾病增強風險相關之任何最新資訊，將即時更新並提供給您。

<u>疫苗佐劑相關的風險</u>

本試驗疫苗所使用的佐劑含Adju-Phos®，是屬於一種磷酸鋁類的佐劑。磷酸鋁類佐劑已經使用超過半個世紀，具有相當的安全性。由於此類佐劑可誘導免疫反應，因此可能會造成局部發炎反應，例如在注射部位產生輕微而短暫的疼痛、發紅以及腫脹。

2. 與試驗/研究過程相關的風險：

<u>**抽血**</u>

本試驗需要抽血檢驗。抽血可能引起一些不適和瘀血。整個試驗期間6個月，共需抽血 243毫升。若需要進行HLA分型檢驗，將額外抽血2毫升。

<u>**鼻咽/喉嚨拭子採檢**</u>

將以鼻咽/喉嚨拭子採檢您的該部位的分泌物，可能會引起噁吐感或咽喉不適。研究人員將做好相關防護措施。

<u>**心電圖(ECG)**</u>

心電圖檢查可建立心臟電流活動的影像。在進行本程序時，您將需要靜躺幾分鐘，讓電極貼附在您的胸前、手臂及腿上。在您皮膚上貼上與取下電極時，可能會引起一些不適。若您為有任何胸毛的男性，可能需要剃除放置電極部位的毛髮。

在接種疫苗過程中，可能會出現一些尚未在已完成試驗中發現的副作用。一般而言，接種某一新疫苗總是會有一定的風險，但是計畫主持人會採取一切措施預防風險的發生。**計畫主持人鼓勵您報告您遇到的任何不適。**

(六) 其他替代療法及說明：

您不是非參加不可，若不參加研究，由於目前尚未有疫苗可用來預防新型冠狀病毒感染，因此預防措施與其他呼吸道感染相同，包括：勤洗手、減少觸摸眼口鼻、注意咳嗽禮節、妥善處理口鼻分泌物等，避免出入公共場所，並不要接觸野生動物。

如果您對於本試驗疫苗有任何的疑問，您可以提出來向您的試驗醫師討論。

(七) 試驗預期效益：

依據臨床前試驗結果，預期本試驗疫苗對您可能可以產生抗體，預防新型冠狀病毒感染，但因每個人體質不同也有可能不會產生療效，故參加本試驗可能不會有直接的好處。

但是您參加本試驗，可協助我們獲得更多資訊，以瞭解UB-612疫苗的安全性與免疫力。

(八)試驗進行中受試者之禁忌、限制與應配合之事項：

禁止使用的藥物

以下藥物請勿在試驗期間使用：

- 禁止使用免疫抑制劑、皮質類固醇或細胞毒性治療
- 禁止使用免疫球蛋白和/或任何血液製劑
- 整個試驗期間使用試驗產品(包括藥物或疫苗)
- 第56天後可接種其他已經上市的疫苗，但應與最近一次訪視間隔至少一個月

允許使用的藥物

若您的藥物或治療必須常規使用，經試驗醫師判斷不會影響本試驗疫苗的免疫原性、臨床療效與安全性，則可以正常使用。您有任何關於在試驗期間可允許使用何種藥物或治療的問題，請詢問您的試驗醫師。

懷孕或母乳哺乳的風險

目前未知本試驗疫苗對於未出生胎兒的影響，因此：

- 您為具生育能力的女性受試者 (除非手術絕育或停經)，或您為男性受試者應於接種疫苗至最後一次疫苗後 3 個月同意進行有效的避孕方式，同意進行有效的避孕方式(例如子宮內節育器、荷爾蒙療法或避孕套)。
- 若您為具生育能力的女性，將請您進行懷孕檢測，結果必須為陰性，方可參與試驗。
- 若您為懷孕的女性，將被告知不可參與本試驗。
- 若您在試驗期間懷孕，請盡速通知試驗人員，並且停止施打本疫苗。
- 基於安全性考量，若您為女性受試者而在試驗期間懷孕，或您為男性受試者而您的性伴侶在試驗期間懷孕(將請您的懷孕性伴侶需簽署另外一份同意書)，您與您的胎兒將會被追蹤監測至分娩，除非另有醫學指示。

您應向您的配偶或性伴侶告知您有參與此試驗與相關風險:

簽名：__________日期：__________________

(九)機密性：

中國醫藥大學附設醫院將依法把任何可辨識您的身分之紀錄與您的個人隱私資料視為機密來處理，不會公開。研究人員將以一個研究代碼代表您的身分，此代碼不會顯示您的姓名、國民身分證統一編號、住址等可識別資料。如果發表試驗/研究結果，您的身分仍將保密。您亦瞭解若簽署同意書即同意您的原始醫療紀錄可直接受監測者、稽核者、研究倫理委員會及主管機關檢閱，以確保臨床試驗/研究過程與數據符合相關法律及法規要求，上述人員並承諾絕不違反您的身分之機密性。除了上述機構依法有權檢視外，我們會小心維護您的隱私。由於試驗藥物可能同時申請美國臨床試驗，依美國藥品管理規定，試驗結果將公佈於公開的臨床試驗資訊網站：Clinicaltrials.gov (美國)，但您的個人資料仍將保密，該網站只會有試驗之結果摘要，您可以在任何時候搜尋該網站。

在試驗/研究期間，依據計畫類型與您所授權的內容，我們將會蒐集與您有關的病歷資料、醫療紀錄、量表、問卷等資料與資訊，並以一個編號來代替您的名字及相關個人資料。前述資料若為紙本型式，將會與本同意書分開存放於研究機構之上鎖櫃中；若為電子方式儲存或建檔以供統計與分析之用，將會存放於設有密碼與適當防毒軟體之專屬電腦內。這些研究資料與資訊將會保存至藥品於我國上市後至少兩年，若試驗疫苗終止研發則保存至試驗正式停止後至少二年，至多將保存至疫苗上市後或試驗正式停止後二年。

上述資料與資訊若傳輸至國外分析與統計，您仍會獲得與本國法規相符之保障，計畫主持人與相關團隊將盡力確保您的個人資料獲得妥善保護。

<需進行 HIV 檢測適用>

因本試驗需排除感染人類免疫缺乏病毒(HIV)者，您將接受人類免疫缺乏病毒(HIV)檢測，若檢驗結果為陰性始得參與本試驗，若檢驗結果為陽性(包含偽陽性)，本試驗將提供後續就醫轉介或諮詢，且經確認後需依法(人類免疫缺乏病毒傳染防治及感染者權益保障條例)通報主管機關。

（十)損害補償與保險：

1. 如依本研究所訂臨床試驗計畫，因發生不良反應造成損害，由聯亞生技開發股份有限公司負補償責任。但本受試者同意書上所記載之可預期不良反應，不予補償。
2. 如依本研究所訂臨床試驗計畫，因而發生不良反應或損害，贊助廠商將依法負責損害賠償責任。本醫院願意提供專業醫療照顧及醫療諮詢。您不必負擔治療不良反應或損害之必要醫療費用。
3. 除前二項補償及醫療照顧外，本研究不提供其他形式之補償。若您不願意接受這樣的風險，請勿參加試驗。
4. 您不會因為簽署本同意書，而喪失在法律上的任何權利。
5. 本研究有投保責任保險。

（十一）受試者權利：

1. 試驗過程中，與您的健康或是疾病有關，可能影響您繼續接受臨床試驗意願的任何重大發現，都將即時提供給您。
2. 如果您在試驗過程中對試驗工作性質產生疑問，對身為患者之權利有意見或懷疑因參與研究而受害時，可與本院之研究倫理委員會聯絡請求諮詢，其電話號碼為：04-22052121轉1925、1926。
3. 為進行試驗工作，您必須接受黃高彬醫師的照顧。如果您現在或於試驗期間有任何問題或狀況，請不必客氣，可與在中國醫藥大學附設醫院兒童感染科的黃高彬醫師聯絡（24小時聯繫電話：0975-681-950）。
4. 參加試驗研究計畫之補助：本計畫將在每次訪視提供交通費3000元給您，整個試驗預計給予您30000~33000元。
5. 本同意書一式2份，醫師已將同意書副本交給您，並已完整說明本研究之性質與目的。醫師已回答您有關藥品與研究的問題。

（十二）試驗之退出與中止：

中國醫藥大學暨附設醫院
受試者同意書（B組）

您可自由決定是否參加本試驗；試驗過程中也可隨時撤銷同意，退出試驗，不需任何理由，且不會引起任何不愉快或影響其日後醫師對您的醫療照顧。<br>計畫主持人或贊助廠商亦可能於必要時中止該試驗之進行。

（十三）簽名：

1. 計畫主持人、或協同主持人已詳細解釋有關本研究計畫中上述研究方法的性質與目的，及可能產生的危險與利益。

   計畫主持人/協同主持人簽名：____________日期：________年____月____日

2. 受試者已詳細瞭解上述研究方法及其所可能產生的危險與利益，有關本試驗計畫的疑問，業經試驗主持人詳細予以解釋。本人同意接受為臨床試驗計畫的自願受試者。

   受試者簽名：____________________________日期：________年____月____日

   法定代理人簽名：________________________日期：________年____月____日

   * 受試者為無行為能力(未滿七歲之未成年人者或禁治產人)，由法定代理人為之；禁治產人，由監護人擔任其法定代理人。

   * 受試者為限制行為人者(滿七歲以上之未成年人)，應得法定代理人之同意。

   有同意權人簽名：________________________日期：________年____月____日

   * 受試者雖非無行為能力或限制行為能力者，但因意識混亂或有精神與智能障礙，而無法進行有效溝通和判斷時，由有同意權之人為之。前項有同意權人為配偶及直系親屬。

3. 見證人

   見證人簽名：_____________________________日期：________年____月____日

   身分證字號：　　　　　　　　　　　　聯絡電話：

   通訊地址：

   * 受試者、法定代理人或有同意權之人皆無法閱讀時，應由見證人在場參與所有有關受試者同意之討論。並確定受試者、法定代理人或有同意權之人之同意完全出於其自由意願後，應於受試者同意書簽名並載明日期。試驗相關人員不得為見證人。

流程表:

訪視	1	2	3	4	5	6	7	8	9	10	11/ET
**檢測項目**	**篩選**	**第一次接種**	**第二次接種**							**第 3 個月追蹤[g]**	**第 6 個月追蹤[h]**
**天數**	**-14~-1**	**0**	**3[c] ±1 天**	**7 ±1 天**	**14 ±3 天**	**28 ±3 天**	**35 ±3 天**	**42 ±3 天**	**56 ±3 天**	**112 ±5 天**	**196 ±15 天**
臨床檢測											
獲得受試者同意書	X										
納入/排除條件	X	X									
第二次接種評估						X					
基本資料	X										
醫療病史	X	X									
身體檢查 [a]	X	X	X	X	X	X	X	X	X	X	X
生命徵象	X	X	X	X	X	X	X	X	X	X	X
心電圖	X	X [i]				X [i]			X		
**實驗室數值檢測**											
B 型肝炎表面抗原, C 型肝炎 RNA, 人類免疫缺乏病毒抗體檢測	X										
核酸檢測(鼻咽/喉嚨拭子)	X										
新型冠狀病毒血清抗體(IgG)檢測	X										
**實驗室檢測 (安全性)**											
血液常規檢測	X			X		X	X				
血液生化學檢測	X[d]			X		X	X				
懷孕(HCG/尿液)檢測 [b]	X	X				X					X

訪視	1	2	3	4	5	6	7	8	9	10	11/ET
**檢測項目**	**篩選**	**第一次接種**	**第二次接種**							**第 3 個月追蹤** [g]	**第 6 個月追蹤** [h]
**天數**	**-14~-1**	**0**	**3**[c] **±1 天**	**7 ±1 天**	**14 ±3 天**	**28 ±3 天**	**35 ±3 天**	**42 ±3 天**	**56 ±3 天**	**112 ±5 天**	**196 ±15 天**
**實驗室檢測(免疫原性)**											
體液免疫原性		X			X	X		X	X	X	X
細胞免疫原性		X		X		X	X				X
**試驗步驟**											
疫苗接種		X				X					
發給日誌卡 [e]		X				X					
回收日誌卡 [e]			X	X			X				
不良事件/嚴重不良事件		X	X	X	X	X	X	X	X	X	X
新型冠種病毒感染監測									X	X	X
併用藥物	X	X	X	X	X	X	X	X	X	X[f]	X[f]

a: 身高與體重僅在第一次訪視測量。
b: 將執行懷孕檢測(對於有懷孕能力的女性於第 0, 28, 196 天使用血清 β-HCG 懷孕檢測和尿液懷孕檢測)，但是已絕經或以手術絕育的女性將不作此檢測。若尿液檢測為陽性，應以血清懷孕檢測再次確認。以血清懷孕檢測替代尿液檢測將不視為試驗偏差。
c: 僅針對每組的前 6 名受試者
d: 僅在第一次訪視執行 HbA1c 檢測。
e: 若使用電子化日誌卡，將沒有發給和收回日誌卡程序。
f: 只記錄嚴重不良事件的使用藥物。
g: 第二次接種疫苗後 3 個月
h: 第二次接種疫苗後 6 個月
i: 應在接種疫苗後一小時內測量心電圖。
ET: 提早退出試驗

中國醫藥大學暨附設醫院
受試者同意書（C組）

黃高彬
2021.3.12

您被邀請參與此研究。此同意書主要是提供您本研究之相關資訊，以便您決定是否參加本研究。計畫主持人或其指定之研究人員會為您說明研究內容並回答您的疑問。您可以提出任何和此研究有關的問題，在您的問題尚未獲得滿意的答覆之前，請不要簽署此同意書。如果您願意參與本研究，此文件將視為您的同意紀錄。即使在您同意後，您可以隨時退出本研究不需任何理由。

<table>
<tr><td colspan="2">計畫名稱
中文：一個評估 UB-612 於成年健康受試者的安全性、耐受性與免疫原性的開放性、第一期臨床試驗
英文：A phase I, open-label study to evaluate the safety, tolerability, and immunogenicity of UB-612 vaccine in healthy adult volunteers</td></tr>
<tr><td>執行單位：中國醫藥大學附設醫院感染科、家庭醫學科</td><td>委託單位/藥廠：聯亞生技開發股份有限公司
研究經費來源：聯亞生技開發股份有限公司
受託研究機構：晉加股份有限公司</td></tr>
<tr><td>計畫主持人：黃高彬</td><td>職稱：主治醫師</td></tr>
<tr><td>協同主持人：林文元</td><td>職稱：主治醫師</td></tr>
<tr><td>協同主持人：林伯昌</td><td>職稱：主治醫師</td></tr>
<tr><td>緊急聯絡人：黃高彬</td><td>電話：0975-681-950</td></tr>
<tr><td>受試者姓名：
性別：
身分證字號：
通訊地址：</td><td>病歷號碼：
出生日期：
聯絡電話：</td></tr>
<tr><td>法定代理人或有同意權人之姓名：
性別：
身分證字號：
通訊地址：</td><td>與受試者關係：
出生日期：
聯絡電話：</td></tr>
</table>

(一)試驗簡介：

1. 本品/技術資料：

新型冠狀病毒(SARS-CoV-2)於2019年12月起造成中國湖北省武漢市發現多起病毒性肺炎群聚，隨後於2020年1月底台灣出現第一起境外移入確診個案。此疾病在全球擴散，世界衛生組織宣布將此疫情為「國際關注公共衛生緊急事件」。截至2020年6月

為止，此疫情已經造成全球824萬人感染，44.6萬人死亡，但是目前在全世界尚未研究出有效的疫苗。

UB-612疫苗為聯亞生技開發股份有限公司所開發新型冠狀病毒預防性疫苗，疫苗含病毒棘狀融合蛋白和胜肽片段，可產生高親和力抗體與新型冠狀病毒結合，並誘發細胞免疫反應，進而達到預防新型冠狀病毒的感染。

2. 本品上市狀況：

本品首次應用於人體試驗，尚未在我國上市。

（二）試驗目的：

- 本試驗之主要試驗目的為評估UB-612疫苗於成年健康受試者之安全性與耐受性。
- 本試驗之次要試驗目的為評估UB-612疫苗於成年健康受試者之免疫原性。

(三) 試驗之主要納入與排除條件：

執行本研究計畫的醫師或相關研究人員將會與您討論有關參加本研究的必要條件。請您配合必須誠實告知我們您過去的健康情形，若您有不符合參加本研究的情況，將不能參加本研究計畫。

1. 納入條件(參加本試驗/研究的條件):

(1) 您為篩選訪視時20-55歲之間健康男性或未懷孕的女性受試者。

(2) 您為具生育能力的女性與男性應於首次接種疫苗至最後一次疫苗後3個月同意進行有效的避孕方式。可接受的有效避孕方式包括:

a. 男性或女性以手術方法絕育、植入式避孕、或子宮避孕器。

b. 注射避孕、避孕藥、避孕貼片、避孕環加上一種屏障避孕法*。

c. 兩種合併使用的屏障避孕法*。

*有效的屏障避孕法為避孕隔膜、男性或女性保險套、避孕海綿或殺精劑(含可殺精化學物質的藥膏或凝膠)。

(3) 您能理解受試者同意書內容的說明與可能的風險，提供簽名的受試者同意書。

(4) 您能夠理解與遵從本試驗程序與能參與每次訪視。

(5) 您的B型肝炎表面抗原檢測、C型肝炎RNA檢測，與人類免疫缺乏病毒抗體檢測呈現陰性。

(6) 以聯亞(UBI)酵素結合免疫吸附分析法進行新型冠狀病毒血清抗體(免疫球蛋白G(IgG))檢測，您的採檢結果呈現陰性。

(7) 以新型冠狀病毒反轉錄聚合酶連鎖反應檢驗鼻咽或喉嚨拭子檢體，您的採檢結果為陰性。

(8) 您的耳溫≤ 38.0°C。

(9) 您的身體質量指數為18-30 kg/m^2。

(10) 經試驗主持人判斷，您的常規血液檢查、血液生化學檢查或其他實驗室數值為正常，或非臨床顯著。

(11) 經試驗主持人判斷，您在篩選訪視時為健康的受試者，健康情況依醫療病史、身體檢查、心電圖、生命徵象(血壓、心跳、體溫、呼吸速率)、臨床實驗檢測(血液常規與生化學檢測)判斷。

2. 排除條件(若您有下列任一情況，您將無法參加本試驗/研究):

(1) 您有接種疫苗後需要醫療介入的過敏性休克、蕁麻疹或其他顯著不良反應的病史。

(2) 您為已懷孕女性或懷孕檢測為陽性的女性。

(3) 您為正在哺乳的女性，或計畫從接種第一劑疫苗至最後一劑疫苗後 60 天哺乳的女性。

(4) 您在接種第一劑疫苗前3天內，經試驗主持人判斷，患有任何急性疾病。

(5) 您有血液學疾病、腎臟疾病、內分泌疾病、肺臟疾病、腸胃道疾病、心血管疾病、肝臟疾病、精神學疾病、神經疾病、或過敏疾病(包含藥物過敏的) 的臨床顯著證據或病史。

(6) 已知您有嚴重急性呼吸道症候群(SARS)或中東呼吸症候群(MERS)冠狀病毒感染史。

(7) 已知您曾暴露於新型冠狀病毒，或曾接受預防新型冠狀病毒、中東呼吸症候群冠狀病毒、嚴重急性呼吸道症候群的試驗產品。

(8) 您在簽署受試者同意書前12周內參與其他的臨床試驗。

(9) 您的醫療狀況可能增加罹患新型冠狀病毒感染後，成為重症的風險，例如慢性腎臟病、慢性阻塞性肺病、嚴重的心臟疾病(例如心臟衰竭、冠狀動脈心臟病或心肌病變) ，或有重大未能控制的慢性疾病，例如氣喘、糖尿病、胸腔疾病。

(10) 您有先天性或後天性血管性水腫。

(11) 您有免疫缺乏/失調疾病，無論是否由基因缺陷、免疫缺乏症或免疫抑制療法所造成。

(12) 您患有血小板異常或其他凝血異常可能造成注射之禁忌症。

(13) 您在接種第一劑疫苗前6個月長期接受(≥14天連續使用)免疫抑制劑、皮質類固醇(相當於一天使用≥ 20 mg強的松(prednisone))或細胞毒性治療。

(14) 您在接種第一劑疫苗前4個月接受免疫球蛋白和/或任何血液製劑的治療。

(15) 您在接種第一劑疫苗前1個月接種過減毒性疫苗、核酸 (mRNA或DNA)或載體疫苗，或預計在接種第二劑疫苗前1個月接種此類疫苗。

(16) 您在接種第一劑疫苗前14天接種過次單元疫苗或去活化疫苗，或預計在接種第二劑疫苗前14天接種此類疫苗。

(17) 您正在接受抗肺結核治療，或有肺結核病史。

(18) 您為酒精成癮及物質濫用者。

(19) 除了皮膚基底細胞癌和子宮頸原位癌，您在篩選訪視前5年有惡性腫瘤病史。

(20) 經試驗主持人判斷，您有任何醫療疾病或狀況，可能會影響試驗結果或參與試驗可能會對受試者引發額外風險。

(四) 試驗方法及相關檢驗：

這是一個第一期、開放性、劑量遞增試驗，評估3種遞增劑量UB-612新型冠狀病毒疫苗接種於健康成年受試者的安全性、耐受性與免疫原性。試驗共納入三組:

本試驗所使用的UB-612疫苗*劑量與預計納入受試者人數如下:

A組	施打兩劑UB-612疫苗(含10微克融合蛋白與胜肽，0.5 毫升)	20位受試者
B組	施打兩劑UB-612疫苗(含30微克融合蛋白與胜肽，0.5 毫升)	20位受試者
C組	施打兩劑UB-612疫苗(含100微克融合蛋白與胜肽，0.5 毫升)	20位受試者

*: UB-612疫苗，含Adju-Phos® 與CpG寡核苷酸佐劑

總計本試驗將納入60名受試者，整個試驗的納入方式為在A組的前20位受試者將納入試驗，施打兩劑UB-612疫苗(含10微克融合蛋白與胜肽)，施打間隔為28天(第0天和第28天)。若經過資料及安全性監測委員會決定A組接種劑量沒有任何的安全疑慮，可以納入B組受試者。若經過資料及安全性監測委員會決定B組接種劑量沒有任何的安全疑慮，可以納入C組受試者。各組將在最後一位受試者完成第9次訪視(第56天)時進行期中分析。

您目前的組別為C組，前6位受試者為前哨組，接受第一劑疫苗後，於第3天和第7天回診。若經過資料及安全性監測委員會決定前6位受試者在7天內沒有發生與試驗疫苗相關第三級以上的不良反應或嚴重不良事件，將可納入隨後的14位受試者。

這個試驗將有11次訪視，包括第1次訪視(篩選期，第-14天至第-1天)，第2次訪視(第0天，基礎值，第一次接種疫苗)，第3次訪視(第3天，只有前6位受試者需要回診)，第4次訪視(第7天)，第5次訪視(第14天)，第6次訪視(第28天，第二次接種疫苗)，第7次訪視(第35天)，第8次訪視(第42天)，第9次訪視(第56天)，第10次訪視(第112天，第二次接種疫苗後3個月)，第11次訪視(第196天，第二次接種疫苗後6個月)。

注意事項

1. 如果您同意參加本試驗，研究人員會請您簽署本份受試者同意書，並確認您符合參加本試驗的條件。
2. 從您參與試驗的當天開始，每次訪視都將有合格的試驗人員執行試驗流程與聯繫。

3. 於試驗期間，您不論任何理由提前退出試驗，試驗研究人員都將安排您完成最後一次的訪視之所有試驗項目。您有權利拒絕此項安排，您的決定不會引起任何影響日後醫師對您的醫療照護。

試驗步驟

筛選訪視(第一次訪視，第-14天~第-1天)

在試驗醫師或試驗研究人員為您提供足夠的試驗資訊，並確保您有充分的時間考慮以及詢問任何問題後，您願意讓您參與本試驗，並由您簽署本受試者同意書。在確認您已完成受試者同意書簽署並且您也保有一份副本後，試驗醫師或試驗研究人員將會進行以下試驗程序：

(1) 記錄您簽署受試者同意書的日期
(2) 為您指定一組受試者篩選編號
(3) 確認您是否符合本試驗的納入排除條件
(4) 收集您的個人基本資料（例如生日、年齡及性別）
(5) 記錄您的醫療/用藥病史
(6) 進行身體檢查，包括身高體重
(7) 確認生命徵象
(8) 進行心電圖檢查
(9) 收集血液檢體(共12毫升)，進行下列檢測:
- 血清懷孕檢測（具有生育能力女性）
- 常規血液檢測
- 血液生化學檢測
- B型肝炎表面抗原, C型肝炎 RNA, 人類免疫缺乏病毒抗體檢測
- 新型冠狀病毒血清抗體(IgG)檢測

(10) 收集鼻咽/喉嚨拭子檢體，進行新型冠狀病毒核酸檢測
(11) 收集併用藥物/治療

進行人類免疫缺乏病毒(HIV)檢測，若檢測結果呈現陽性，依法將通報主管機關。

☐同意　簽名：__________日期：__________________

第二次訪視(第0天)-基礎值，第一次接種疫苗

將會進行以下試驗程序：

(1) 確認您是否符合本試驗的納入排除條件

(2) 為您指定一組受試者編號
(3) 若有任何接種疫苗前的醫療狀況，應紀錄為醫療病史
(4) 進行身體檢查
(5) 確認生命徵象
(6) 進行尿液懷孕檢測（具生育能力的女性）
(7) 接種疫苗前，收集血液檢體(共 40 毫升)，進行下列檢測:
- 體液免疫原性
- 細胞免疫原性

(8) 進行第一次疫苗接種。接種疫苗後，每組前 6 位受試者應留在試驗地點至少 2-4 小時，隨後的 14 位受試者應留在試驗地點至少 60 分鐘，每 30 分鐘監測一次生命徵象和不良事件。
(9) 接種疫苗後一小時內測量心電圖
(10) 將發給您日誌卡，並詳細地指導您如何填寫日誌卡。
(11) 收集併用藥物/治療

<u>第三次訪視(第 3±1 天)-僅前 6 個受試者需要回診</u>

將會進行以下試驗程序：

(1) 確認您接種疫苗後的安全性
(2) 進行身體檢查
(3) 確認生命徵象
(4) 收集併用藥物/治療
(5) 記錄上一次訪視至此次訪視之間的不良事件或嚴重不良事件

<u>第四次訪視(第 7±1 天)</u>

將會進行以下試驗程序：

(1) 進行身體檢查
(2) 確認生命徵象
(3) 收集血液檢體(共 42 毫升) ，進行下列檢測:
- 常規血液檢測
- 血液生化學檢測
- 細胞免疫原性

(4) 收回日誌卡。
(5) 收集併用藥物/治療
(6) 記錄上一次訪視至此次訪視之間的不良事件或嚴重不良事件

第五次訪視(第 14±3 天)

將會進行以下試驗程序：

(1) 進行身體檢查

(2) 確認生命徵象

(3) 收集血液檢體(共 5 毫升) ，進行下列檢測:

- 體液免疫原性

(4) 收集併用藥物/治療

(5) 記錄上一次訪視至此次訪視之間的不良事件或嚴重不良事件

第六次訪視(第 28±3 天) -第二次接種疫苗

將會進行以下試驗程序：

(1) 進行第二次接種評估

(2) 進行身體檢查

(3) 確認生命徵象

(4) 進行尿液懷孕檢測（具生育能力的女性）

(5) 接種疫苗前，收集血液檢體(共 47 毫升)，進行下列檢測:

- 常規血液檢測
- 血液生化學檢測
- 體液免疫原性
- 細胞免疫原性

(6) 進行第二次疫苗接種。接種疫苗後，應留在試驗地點 60 分鐘，每 30 分鐘監測一次生命徵象和不良事件。

(7) 接種疫苗後一小時內測量心電圖

(8) 將發給您日誌卡，並詳細地指導您如何填寫日誌卡。

(9) 收集併用藥物/治療

(10) 記錄上一次訪視至此次訪視之間的不良事件或嚴重不良事件

第七次訪視(第 35±3 天)

將會進行以下試驗程序：

(1) 進行身體檢查

(2) 確認生命徵象

(3) 收集血液檢體(共 42 毫升) ，進行下列檢測:

- 常規血液檢測
- 血液生化學檢測

- 細胞免疫原性

(4) 收回日誌卡。

(5) 收集併用藥物/治療

(6) 記錄上一次訪視至此次訪視之間的不良事件或嚴重不良事件

第八次訪視(第 42±3 天)

將會進行以下試驗程序：

(1) 進行身體檢查

(2) 確認生命徵象

(3) 收集血液檢體(共 5 毫升) ，進行下列檢測:
- 體液免疫原性

(4) 收集併用藥物/治療

記錄上一次訪視至此次訪視之間的不良事件或嚴重不良事件

第九次訪視(第 56±3 天)

將會進行以下試驗程序：

(1) 進行身體檢查

(2) 確認生命徵象

(3) 進行心電圖檢查

(4) 收集血液檢體(共 5 毫升) ，進行下列檢測:
- 體液免疫原性

(5) 收集併用藥物/治療

(6)若您同意，將施打一劑流感疫苗

☐同意　簽名：__________日期：_____________

(7) 進行新型冠狀病毒感染監測

*新型冠狀病毒感染監測: 在試驗第 56 天後至試驗結束，若您有發燒、咳嗽、或其他呼吸道症狀應立即至試驗地點回診。試驗團隊將會以鼻咽/喉嚨拭子收集檢體，並進行電腦斷層掃描或其他影像學檢查，以確認您是否有新型冠狀病毒感染。若您為新型冠狀病毒感染的患者，也會觀察您是否有疾病增強的反應(請見(五)可能產生之副作用、發生率及處理方法，關於疾病增強的說明)

(8) 記錄上一次訪視至此次訪視之間的不良事件或嚴重不良事件

第十次訪視(第 112±5 天) –第 3 個月追蹤

將會進行以下試驗程序：

(1) 進行身體檢查

(2) 確認生命徵象

(3) 收集血液檢體(共 5 毫升) ，進行下列檢測:

- 體液免疫原性

(4) 收集併用藥物/治療

(5) 進行新型冠狀病毒感染監測

(6) 記錄上一次訪視至此次訪視之間的不良事件或嚴重不良事件

第十一次訪視(第 196±15 天)-第 6 個月追蹤

(1) 進行身體檢查

(2) 確認生命徵象

(3) 尿液懷孕檢測（具生育能力的女性）

(4) 收集血液檢體(共 40 毫升) ，進行下列檢測:

- 體液免疫原性
- 細胞免疫原性

(5) 收集併用藥物/治療

(6) 進行新型冠狀病毒感染監測

(7) 記錄上一次訪視至此次訪視之間的不良事件或嚴重不良事件

受試者之檢體(含其衍生物)之保存、使用與再利用：

1. 檢體及剩餘檢體之保存與使用

(1) 檢體(含其衍生物)之保存與使用

為研究所需，我們所蒐集您的檢體，將依本研究計畫使用，檢體將保存於聯亞生技開發(股)公司及聯合生物製藥(股)公司，直至 20 年保存期限居滿，我們將依法銷毀。為了保護您的個人隱私，我們將以一個試驗編號來代替您的名字及相關個人資料，以確認您的檢體及與相關資料受到完整保密。如果您對檢體的使用有疑慮，或您有任何想要銷毀檢體的需求，請立即與我們聯絡(聯絡人：黃高彬醫師電話：0975-681-950)，我們即會將您的檢體銷毀。您也可以聯繫中國醫藥大學暨附設醫院研究倫理委員會(電話：04-22052121 轉 1925、1926)，以協助您解決檢體在研究使用上的任何爭議。

(2) 剩餘檢體(含其衍生物)之再利用

您的生物檢體將會以專屬號碼進行編碼並在聯亞生技開發股份有限公司(試驗委託者)的控管下儲存最長20年。

中國醫藥大學暨附設醫院
受試者同意書（C組）

所有新的研究計畫都要再經由中國醫藥大學暨附設醫院研究倫理委員會審議通過，倫理審查委員會若認定新的研究超出您同意的範圍，將要求我們重新得到您的同意。

是否同意剩餘檢體保留提供未來新型冠狀病毒感染研究之用，並授權中國醫藥大學暨附設醫院研究倫理委員會審議是否需要再取得您的同意(擇一)

☐ 不同意保存我的剩餘檢體，試驗結束後請銷毀

☐ 同意以非去連結之方式保存我的剩餘檢體，逾越原同意使用範圍時，需再次得到我的同意才可使用我的檢體進行新的研究

您的剩餘檢體將運送至以下實驗室進行新型冠狀病毒變異株的檢驗:

**實驗室名稱**	**機構地址**
**UTMB**	**University of Texas Medical Branch 301 University Boulevard Keiller Building, Room 2.150 Galveston, Texas, USA(美國)**
**Virology (Tropical Medicine Institute, University of São Paulo)**	**University of São Paulo, Brazil Rua Dr Enéas de Carvalho Aguiar 470 , CEP 05403-000(巴西)**
**Viral and Rickettsial Disease Laboratory (VRDL)**	**850 Marina Bay Parkway Richmond, CA 94804, USA(美國)**

請問您是否同意?

☐ 同意 ☐ 不同意　簽名：＿＿＿＿＿＿日期：＿＿＿＿＿＿＿＿

2. 檢體及剩餘檢體之部分類型(檢體類型可依計畫書內容自行增減)

(1) 一般生化、血液檢驗檢體、鼻咽/喉嚨拭子檢體

在試驗期間，會將您的檢體送往聯亞生技開發股份有限公司(試驗委託者) 委託的中央實驗室中國醫藥大學暨附設醫院分析，此機構地址為台中市北區育德路2號，中央實驗室會在分析後立即將分析結果提供給試驗中心，若有剩餘的檢體，將會儲存直到檢驗結果複驗完畢即銷毀，不會長期儲存。

(2) 抗體/細胞免疫試驗

在試驗期間，會將您的檢體送往聯亞生技開發(股)公司(試驗委託者)分析實驗室及聯合生物製藥(股)公司生物分析實驗室進行處置、處理與進一步分析。聯亞生技開發(股)公司機構地址為新竹縣竹北市生醫路二段 6-1 號 5 樓，聯合生物製藥(股)公司機構地址為新竹縣竹北市生醫路二段 12 號 1 樓。完成試驗後，若有剩餘檢體，將儲存於聯

亞生技開發(股)公司及聯合生物製藥(股)公司，直到至少完成臨床試驗報告為止，最長將保存 20 年。

(3) 中和試驗(neutralization test, NT)

在試驗期間，會將您的檢體送往聯亞生技開發股份有限公司(試驗委託者)委託的中央實驗室中央研究院進行處置、處理與進一步分析。此機構地址為台北市南港區研究院路二段128號。完成試驗後，若有剩餘檢體，將儲存直到至少完成臨床試驗報告為止，最長將保存20年。

(4) 遺傳學檢體

在試驗期間，若發生嚴重不良反應或特定不良反應，您的DNA檢體將用於HLA分型檢驗，會將您的檢體送往聯亞生技開發股份有限公司(試驗委託者)委託的中央實驗室有勁基因分析，此機構地址為新北市樹林區復興路376-5號，中央實驗室會在分析後立即將分析結果提供給試驗中心，若有剩餘的檢體，將會儲存直到檢驗結果複驗完畢即銷毀，不會長期儲存。

(五)可能產生之副作用、發生率及處理方法：

1. 與試驗藥物相關的風險（本試驗疫苗的副作用）：

冠狀病毒疫苗的開發

過去針對與SARS-CoV-2病毒相同屬於人類冠狀病毒的SARS-CoV(嚴重急性呼吸綜合症冠狀病毒(SARS冠狀病毒))的疫苗研究發現，接種過SARS-CoV疫苗的小鼠在暴露到SARS-CoV後會發生過度免疫反應而產生病變，因此不得不停止這種疫苗的開發。所以，成功的人類冠狀病毒疫苗不只要產生可以抑制病毒的免疫反應，更要避免過度免疫產生的副作用。

疫苗相關的風險

本試驗疫苗首次使用於人體，因此尚未有人體安全性資料。

接種疫苗可能會出現注射部位的不良反應(例如疼痛、硬化腫脹、皮疹發紅、過敏反應、蜂窩性組織炎)，或全身性不良反應(例如發燒、腹瀉、疲倦、噁心/嘔吐、厭食、咽喉痛、頭痛、咳嗽、關節痛、非注射部位疼痛、非注射部位搔癢、皮膚和黏膜異常、急性過敏反應、昏厥、急性支氣管痙攣、呼吸困難)。

疾病增強(disease enhancement) 的風險

SARS-CoV-2候選疫苗也可能會有引發疾病增強(disease enhancement) 的風險，包括抗體

依賴性增強(antibody-dependent enhancement)或疫苗相關聯的增強的呼吸道疾病(vaccine-associated enhanced respiratory disease)。在先前研發SARS疫苗時，在數個SARS-CoV動物攻毒試驗(包括鼠類、雪貂、猴類)當中，有發現疾病增強的現象。疾病增強反應的免疫病理現象包括TH2偏向及嗜酸性白血球的肺部浸潤。但是目前已發表的新型冠狀病毒肺炎疫苗研究，仍尚未發現類似的疾病增強現象。

本試驗疫苗於數個藥理試驗呈現不一致的TH1/TH2偏向，雖未一致偏向TH2，仍已規劃並正在執行動物攻毒模型試驗，以進一步排除疾病增強風險。依據文獻指出，組成複雜或容易引起非中和抗體之抗原，如不活化病毒或整片段之蛋白(包含S蛋白與N蛋白)，與易引起偏向Th2免疫反應之佐劑成分，如鋁製佐劑，皆較有可能引起疾病增強。本試驗疫苗的主要抗原為S蛋白上之RBD 區域，已有多篇文獻指出，針對S-RBD設計之SARS與MERS疫苗從未於試驗動物模型上引發疾病增強現象。本試驗疫苗雖使用易引起偏向Th2免疫反應之佐劑，但由動物實驗證實，也同時引起偏向Th1之反應，因此發生疾病增強應屬低風險。且已於多種動物模型中證實，能誘發高效價之中和抗體，於細胞培養中亦能有效抑制新冠病毒感染。

建議您在有效疫苗上市前或本試驗疫苗的產品資訊有進一步更新前，盡量避免暴露於可能感染病毒的環境。研究團隊將會在試驗中執行相關安全性監測。若有任何關於本試驗疫苗與疾病增強風險相關之任何最新資訊，將即時更新並提供給您。

疫苗佐劑相關的風險

本試驗疫苗所使用的佐劑含Adju-Phos®，是屬於一種磷酸鋁類的佐劑。磷酸鋁類佐劑已經使用超過半個世紀，具有相當的安全性。由於此類佐劑可誘導免疫反應，因此可能會造成局部發炎反應，例如在注射部位產生輕微而短暫的疼痛、發紅以及腫脹。

2. 與試驗/研究過程相關的風險：

抽血

本試驗需要抽血檢驗。抽血可能引起一些不適和瘀血。整個試驗期間6個月，共需抽血243毫升。若需要進行HLA分型檢驗，將額外抽血2毫升。

鼻咽/喉嚨拭子採檢

將以鼻咽/喉嚨拭子採檢您的該部位的分泌物，可能會引起噁吐感或咽喉不適。研究人員將做好相關防護措施。

心電圖(ECG)

心電圖檢查可建立心臟電流活動的影像。在進行本程序時，您將需要靜躺幾分鐘，讓電極貼附在您的胸前、手臂及腿上。在您皮膚上貼上與取下電極時，可能會引起一些不適。

若您為有任何胸毛的男性，可能需要剃除放置電極部位的毛髮。

在接種疫苗過程中，可能會出現一些尚未在已完成試驗中發現的副作用。一般而言，接種某一新疫苗總是會有一定的風險，但是計畫主持人會採取一切措施預防風險的發生。**計畫主持人鼓勵您報告您遇到的任何不適。**

(六) 其他替代療法及說明：

您不是非參加不可，若不參加研究，由於目前尚未有疫苗可用來預防新型冠狀病毒感染，因此預防措施與其他呼吸道感染相同，包括：勤洗手、減少觸摸眼口鼻、注意咳嗽禮節、妥善處理口鼻分泌物等，避免出入公共場所，並不要接觸野生動物。

如果您對於本試驗疫苗有任何的疑問，您可以提出來向您的試驗醫師討論。

(七) 試驗預期效益：

依據臨床前試驗結果，預期本試驗疫苗對您可能可以產生抗體，預防新型冠狀病毒感染，但因每個人體質不同也有可能不會產生療效，故參加本試驗可能不會有直接的好處。

但是您參加本試驗，可協助我們獲得更多資訊，以瞭解UB-612疫苗的安全性與免疫力。

(八)試驗進行中受試者之禁忌、限制與應配合之事項：

禁止使用的藥物

以下藥物請勿在試驗期間使用：

- 禁止使用免疫抑制劑、皮質類固醇或細胞毒性治療
- 禁止使用免疫球蛋白和/或任何血液製劑
- 整個試驗期間使用試驗產品(包括藥物或疫苗)
- 第56天後可接種其他已經上市的疫苗，但應與最近一次訪視間隔至少一個月

允許使用的藥物

若您的藥物或治療必須常規使用，經試驗醫師判斷不會影響本試驗疫苗的免疫原性、臨床療效與安全性，則可以正常使用。您有任何關於在試驗期間可允許使用何種藥物或治療的問題，請詢問您的試驗醫師。

懷孕或母乳哺乳的風險

目前未知本試驗疫苗對於未出生胎兒的影響，因此：

- 您為具生育能力的女性受試者 (除非手術絕育或停經)，或您為男性受試者應於接種疫苗至最後一次疫苗後 3 個月同意進行有效的避孕方式，同意進行有效的避孕方式(例如子宮內節育器、荷爾蒙療法或避孕套)。
- 若您為具生育能力的女性，將請您進行懷孕檢測，結果必須為陰性，方可參與試驗。
- 若您為懷孕的女性，將被告知不可參與本試驗。
- 若您在試驗期間懷孕，請盡速通知試驗人員，並且停止施打本疫苗。
- 基於安全性考量，若您為女性受試者而在試驗期間懷孕，或您為男性受試者而您的性伴侶在試驗期間懷孕(將請您的懷孕性伴侶需簽署另外一份同意書)，您與您的胎兒將會被追蹤監測至分娩，除非另有醫學指示。

您應向您的配偶或性伴侶告知您有參與此試驗與相關風險:

簽名：__________日期：__________________

（九)機密性：

中國醫藥大學附設醫院將依法把任何可辨識您的身分之紀錄與您的個人隱私資料視為機密來處理，不會公開。研究人員將以一個研究代碼代表您的身分，此代碼不會顯示您的姓名、國民身分證統一編號、住址等可識別資料。如果發表試驗/研究結果，您的身分仍將保密。您亦瞭解若簽署同意書即同意您的原始醫療紀錄可直接受監測者、稽核者、研究倫理委員會及主管機關檢閱，以確保臨床試驗/研究過程與數據符合相關法律及法規要求，上述人員並承諾絕不違反您的身分之機密性。除了上述機構依法有權檢視外，我們會小心維護您的隱私。由於試驗藥物可能同時申請美國臨床試驗，依美國藥品管理規定，試驗結果將公佈於公開的臨床試驗資訊網站：Clinicaltrials.gov (美國)，但您的個人資料仍將保密，該網站只會有試驗之結果摘要，您可以在任何時候搜尋該網站。

在試驗/研究期間，依據計畫類型與您所授權的內容，我們將會蒐集與您有關的病歷資料、醫療紀錄、量表、問卷等資料與資訊，並以一個編號來代替您的名字及相關個人資料。前述資料若為紙本型式，將會與本同意書分開存放於研究機構之上鎖櫃中；若為電子方式儲存或建檔以供統計與分析之用，將會存放於設有密碼與適當防毒軟體之專屬電腦內。這些研究資料與資訊將會保存至藥品於我國上市後至少兩年，若試驗疫苗終止研發則保存至試驗正式停止後至少二年，至多將保存至疫苗上市後或試驗正式停止後二年。

上述資料與資訊若傳輸至國外分析與統計，您仍會獲得與本國法規相符之保障，計畫主持人與相關團隊將盡力確保您的個人資料獲得妥善保護。

<需進行 HIV 檢測適用>

因本試驗需排除感染人類免疫缺乏病毒(HIV)者，您將接受人類免疫缺乏病毒(HIV)檢測，若檢驗結果為陰性始得參與本試驗，若檢驗結果為陽性(包含偽陽性)，本試驗將提供後續就醫轉介或諮詢，且經確認後需依法(人類免疫缺乏病毒傳染防治及感染者權益保障條例)通報主管機關。

（十)損害補償與保險：

1. 如依本研究所訂臨床試驗計畫，因發生不良反應造成損害，由聯亞生技開發股份有限公司負補償責任。但本受試者同意書上所記載之可預期不良反應，不予補償。
2. 如依本研究所訂臨床試驗計畫，因而發生不良反應或損害，贊助廠商將依法負責損害賠償責任。本醫院願意提供專業醫療照顧及醫療諮詢。您不必負擔治療不良反應或損害之必要醫療費用。
3. 除前二項補償及醫療照顧外，本研究不提供其他形式之補償。若您不願意接受這樣的風險，請勿參加試驗。
4. 您不會因為簽署本同意書，而喪失在法律上的任何權利。
5. 本研究有投保責任保險。

（十一）受試者權利：

1. 試驗過程中，與您的健康或是疾病有關，可能影響您繼續接受臨床試驗意願的任何重大發現，都將即時提供給您。
2. 如果您在試驗過程中對試驗工作性質產生疑問，對身為患者之權利有意見或懷疑因參與研究而受害時，可與本院之研究倫理委員會聯絡請求諮詢，其電話號碼為：04-22052121轉1925、1926。
3. 為進行試驗工作，您必須接受黃高彬醫師的照顧。如果您現在或於試驗期間有任何問題或狀況，請不必客氣，可與在中國醫藥大學附設醫院兒童感染科的黃高彬醫師聯絡（24小時聯繫電話：0975-681-950）。
4. 參加試驗研究計畫之補助：本計畫將在每次訪視提供交通費3000元給您，整個試驗預計給予您30000~33000元。
5. 本同意書一式2份，醫師已將同意書副本交給您，並已完整說明本研究之性質與目的。醫師已回答您有關藥品與研究的問題。

（十二）試驗之退出與中止：

您可自由決定是否參加本試驗；試驗過程中也可隨時撤銷同意，退出試驗，不需

任何理由，且不會引起任何不愉快或影響其日後醫師對您的醫療照顧。

計畫主持人或贊助廠商亦可能於必要時中止該試驗之進行。

（十三）簽名：

1. 計畫主持人、或協同主持人已詳細解釋有關本研究計畫中上述研究方法的性質與目的，及可能產生的危險與利益。

   計畫主持人/協同主持人簽名：____________日期：________年____月____日

2. 受試者已詳細瞭解上述研究方法及其所可能產生的危險與利益，有關本試驗計畫的疑問，業經試驗主持人詳細予以解釋。本人同意接受為臨床試驗計畫的自願受試者。

   受試者簽名：____________________________日期：________年____月____日

   法定代理人簽名：________________________日期：________年____月____日

   * 受試者為無行為能力(未滿七歲之未成年人者或禁治產人)，由法定代理人為之；禁治產人，由監護人擔任其法定代理人。

   * 受試者為限制行為人者(滿七歲以上之未成年人)，應得法定代理人之同意。

   有同意權人簽名：________________________日期：________年____月____日

   * 受試者雖非無行為能力或限制行為能力者，但因意識混亂或有精神與智能障礙，而無法進行有效溝通和判斷時，由有同意權之人為之。前項有同意權人為配偶及直系親屬。

3. 見證人

   見證人簽名：______________________________日期：________年____月____日

   身分證字號：　　　　　　　　　　　聯絡電話：

   通訊地址：

   * 受試者、法定代理人或有同意權之人皆無法閱讀時，應由見證人在場參與所有有關受試者同意之討論。並確定受試者、法定代理人或有同意權之人之同意完全出於其自由意願後，應於受試者同意書簽名並載明日期。試驗相關人員不得為見證人。

中國醫藥大學暨附設醫院
受試者同意書（C組）

流程表:

訪視	1	2	3	4	5	6	7	8	9	10	11/ET
**檢測項目**	**篩選**	**第一次接種**				**第二次接種**				**第 3 個月追蹤** [g]	**第 6 個月追蹤** [h]
**天數**	**-14~-1**	**0**	**3**[c] **±1 天**	**7 ±1 天**	**14 ±3 天**	**28 ±3 天**	**35 ±3 天**	**42 ±3 天**	**56 ±3 天**	**112 ±5 天**	**196 ±15 天**
**臨床檢測**											
獲得受試者同意書	X										
納入/排除條件	X	X									
第二次接種評估						X					
基本資料	X										
醫療病史	X	X									
身體檢查 [a]	X	X	X	X	X	X	X	X	X	X	X
生命徵象	X	X	X	X	X	X	X	X	X	X	X
心電圖	X	X[i]				X[i]			X		
**實驗室數值檢測**											
B 型肝炎表面抗原, C 型肝炎 RNA, 人類免疫缺乏病毒抗體檢測	X										
核酸檢測(鼻咽/喉嚨拭子)	X										
新型冠狀病毒血清抗體(IgG)檢測	X										
**實驗室檢測 (安全性)**											
血液常規檢測	X			X		X	X				
血液生化學檢測	X[d]			X		X	X				
懷孕(HCG/尿液)檢測 [b]	X	X				X					X

中國醫藥大學暨附設醫院
受試者同意書（C組）

訪視	1	2	3	4	5	6	7	8	9	10	11/ET
檢測項目	篩選	第一次接種			第二次接種					第 3 個月追蹤[g]	第 6 個月追蹤[h]
天數	-14~-1	0	3[c] ±1 天	7 ±1 天	14 ±3 天	28 ±3 天	35 ±3 天	42 ±3 天	56 ±3 天	112 ±5 天	196 ±15 天
**實驗室檢測(免疫原性)**											
體液免疫原性		X			X	X		X	X	X	X
細胞免疫原性		X		X		X	X				X
**試驗步驟**											
疫苗接種		X				X					
發給日誌卡[e]		X				X					
回收日誌卡[e]			X	X			X				
不良事件/嚴重不良事件		X	X	X	X	X	X	X	X	X	X
新型冠種病毒感染監測									X	X	X
併用藥物	X	X	X	X	X	X	X	X	X	X[f]	X[f]

a: 身高與體重僅在第一次訪視測量。
b: 將執行懷孕檢測(對於有懷孕能力的女性於第 0, 28, 196 天使用血清 β-HCG 懷孕檢測和尿液懷孕檢測)，但是已絕經或以手術絕育的女性將不作此檢測。若尿液檢測為陽性，應以血清懷孕檢測再次確認。以血清懷孕檢測替代尿液檢測將不視為試驗偏差。
c: 僅針對每組的前 6 名受試者
d: 僅在第一次訪視執行 HbA1c 檢測。
e: 若使用電子化日誌卡，將沒有發給和收回日誌卡程序。
f: 只記錄嚴重不良事件的使用藥物。
g: 第二次接種疫苗後 3 個月
h: 第二次接種疫苗後 6 個月
i: 應在接種疫苗後一小時內測量心電圖。
ET: 提早退出試驗

Appendix 4

[illegible]
11 Aug. 2021

CLINICAL STUDY PROTOCOL

A Phase I, Open-Label Extension Study to Evaluate the Safety, Tolerability, and Immunogenicity of UB-612 Vaccine in healthy adult volunteers

**Protocol Number:**	V-123
**EudraCT Number:**	Not Applicable
**Investigational Product:**	UB-612
**Phase:**	I extension
**Sponsor:**	聯亞生技開發股份有限公司<br>United Biomedical, Inc., Asia (UBI Asia),<br>Hsinchu County, Taiwan
Protocol Date:	**09 August, 2021**
Protocol Version:	1.2

CONFIDENTIAL
This protocol may not be reproduced or communicated to a third party without the written permission of United Biomedical, Inc., Asia

1 PROTOCOL APPROVAL SIGNATURES

Protocol Title: A Phase I, Open-Label Extension Study to Evaluate the Safety, Tolerability, and Immunogenicity of UB-612 Vaccine in healthy adult volunteers

Protocol Number: V-123

This study will be conducted in compliance with the clinical study protocol (and amendments), International Council for Harmonisation (ICH) guidelines for current Good Clinical Practice (cGCP) and applicable regulatory requirements.

Sponsor Signatory

United Biomedical, Inc., Asia (UBI Asia),
No. 45, Guangfu N. Rd., Hukou Township,
Hsinchu County 303, Taiwan (R.O.C.)

Signature

Date

Reviewed and Approved by:

Sponsor's Representative

Thomas P. Monath, MD, FACP, FASTMH
Vaxxinity Inc.
1717 Main Street, Suite 3388
Dallas, TX 75201
Mobile: (978) 549-0708
Email: tom@vaxxinity.com

DocuSigned by:
Thomas P Monath
BE1FC5087856494...
Signature
8/12/2021
Date

Sponsor's Medical Expert

D. Gray Heppner, MD, FACP, FASTMH
Vaxxinity Inc.
1717 Main Street, Suite 3388
Dallas, TX 75201
Mobile: (703) 598-1940
Email: gray@vaxxinity.com

DocuSigned by:
Donald Gray Heppner
6D94BC78249D4A1...
Signature
8/12/2021
Date

2 SYNOPSIS

Protocol Number:

V-123

Title:

A Phase I, Open-Label Extension Study to Evaluate the Safety, Tolerability, and Immunogenicity of UB-612 Vaccine in healthy adult volunteers

Investigational Product:

UB-612 vaccine

Phase:

Phase I extension study

Objectives:

Primary Objective:

- To evaluate the safety and tolerability of a booster dose of UB-612 vaccine in Phase I subjects.
- To evaluate the SARS-CoV-2 neutralizing antibody titers induced by a booster dose of UB-612.

Secondary Objective:

- To evaluate the humoral immune response to SARS-CoV-2 during this study.

Exploratory Objective:

- To evaluate the cellular immune response induced by **a booster dose of** UB-612.
- To evaluate the neutralizing antibody titer against SARS-CoV-2 variants induced by **a booster dose of** UB-612.
- **To compare primary series and booster vaccination of UB-612 on humoral immune responses**
- **To evaluate the kinetics of humoral immune responses of primary series and booster vaccination**
- **To evaluate the effect of prime-boost interval on immune responses**

Study Design:

This is an extension study to evaluate the safety, tolerability and immunogenicity of one booster dose of UB-612 COVID-19 vaccine in adults who completed two vaccinations of UB-612 vaccine 10, 30, and 100 μg in V-122 study (Phase I).

The subjects who completed two vaccinations in Phase I study will be recruited in this extension study. After the inform**ed** consent is obtained from the subject, eligible subjects will receive one booster dose of UB-612 vaccines 100 μg with the same dose which was offered in Phase II study, at least 6 months after **completing the 2-dose primary vaccination series..** This study will be carried out in three groups:

(1) A group: 1 booster dose of UB-612 vaccine 100 μg, for subjects at UB-612 vaccine 10 μg group previously

(2) B group: 1 booster dose of UB-612 vaccine 100 μg, for subjects at UB-612 vaccine 30 μg group previously

(3) C group: 1 booster dose of UB-612 vaccine 100 μg, for subjects at UB-612 vaccine 100 μg group previously

In this study, there will be 3 clinical visits. Subjects will come to the clinics at Visit 1 (Day 1, baseline, booster vaccination, at least 6 months after **completing the 2-dose primary vaccination series** in V-122 study), Visit 2 (Day 15), and Visit 3 (Day 85, 3 months after booster vaccination).

In all groups, an informed consent and the eligibility of subjects will be checked at Visit 1(Day 1). The booster study vaccine administration will be done immediately after blood draw which will evaluate baseline laboratory safety and humoral / cellular immune response/ SARS-CoV-2 test. All subjects in each group will be monitored at least 30 minutes after vaccination (for vital signs change and adverse events). The post-vaccination e-diary card for 7-day

solicited adverse events after vaccination will be given to subjects with a suitable instruction.

At Visit 2 (Day 15), subjects will return to clinics for laboratory safety check and have a blood drawn for humoral / cellular immune response.

At Visit 3 (Day 85, 3-month follow-up visit after booster vaccination), the subjects will return to clinics and have blood drawn for humoral immune / cellular immune response/ SARS-CoV-2 test.

All adverse events and serious adverse events will be monitored from Visit 1 (Day 1) until Visit 3 (Day 85).

Number of Subjects:

All subjects, up to 60 subjects, who completed two vaccinations in Phase I study will be enrolled into this extension study.

Treatment:

One 0.5 mL dose of UB-612 vaccines, 200 µg/mL, containing Adju-Phos® and CpG1, will be administered by an intramuscular (IM) injection **in the deltoid muscle** (on Day 1).

Duration of Treatment: 3 months

Study Population:

To be eligible for study entry, subjects must satisfy all of the following inclusion criteria:

1. Male or non-pregnant female who previously participated in and completed two vaccinations in the V-122 Study.
2. Women of childbearing potential and men must agree to practice medically effective contraception during study period. The acceptable effective contraception methods include:
    a. Male or female sterilization, implant, or intrauterine device;
    b. Injectable, pill, patch, ring plus one barrier method*;
    c. Two combined barrier methods*.

    *Effective barrier methods are diaphragm, male or female condoms, sponge, or spermicides (creams or gels that contain a chemical to kill sperm).
3. Able to understand the content and possible risks of informed consent and willing to sign the Informed Consent Form (ICF).
4. Able to understand and agrees to comply with all study procedures and be available for all study visits.
5. Ear temperature ≤ 38.0°C.
6. At screening visit, at least 6 months after **completing the 2-dose primary vaccination series** in the V-122 study.

Subjects will be excluded from the study if one or more of the following exclusion criteria is applicable:

1. Female who is pregnant or positive in pregnancy test at screening visit.
2. Female who is breast-feeding or plans to breastfeed within 90 days after booster vaccination.
3. Any acute illness, as determined by the study investigator 3 days before booster vaccination.
4. Prior administration of attenuated, nucleic acid (mRNA or DNA) or vectored vaccines in last 1 month before booster vaccination or expectation of such vaccines in the month after the booster vaccination.
5. Prior administration of subunit vaccine or inactivated vaccine in last 14 days before booster vaccination or expectation of receipt of such vaccines in the 14 days after the booster vaccination.
6. Any medical disease or condition that, in the opinion of the study investigator, may confound the results of the study or pose an additional risk to the subjects by their participation in the study.

7. Previous exposure to SARS-CoV-2 or receipt of an investigational or EUA vaccine product for the prevention of COVID-19, MERS or SARS, except UB-612.
8. Subjects who take part in another clinical study, other than V-122 study, within 12 weeks prior to the day of informed consent.
9. Prior administration of immunoglobulins and/or any blood products in last 4 months before booster vaccination.
10. Prior chronic administration (defined as ≥ 14 day of continuous use) of immunosuppressant or corticosteroids (equivalent to ≥ 20 mg daily of prednisone), cytotoxic treatment in last 6 months before booster vaccination.

Primary Endpoint(s):

Immunogenicity Endpoint(s)

- Geometric mean titer (GMT) of neutralizing antibody against SARS-CoV-2 **(Wuhan strain)** on Day 1, 15 and 85
- Geometric mean fold increase **(GMFI)** of neutralizing antibody against SARS-CoV-2 **(Wuhan strain)** on Day 15 and 85

Safety Endpoint(s)

- Occurrence of adverse reactions within 7 days after vaccination
- The percentage of subjects with ≥ Grade 3 adverse events within 7 days after vaccination

Secondary Endpoint(s):

Immunogenicity Endpoint(s)

- GMT of antigen-specific antibody (Anti-S1-RBD) on Day 1, 15 and 85
- Geometric mean fold increase of antigen-specific antibody (Anti-S1-RBD) on Day 15 and 85
- Distribution of titers
- Correlation between the immune response detected by ELISA and live virus neutralization test

Safety Endpoint(s)

- Occurrence of serious adverse events during the whole follow-up period (3 months)
- Occurrence of adverse events of special interests (AESIs), medically attend adverse events (MAAEs) and serious adverse events (SAEs) during the study period
- Changes of safety laboratory measures

Exploratory Endpoint(s):

- T cell responses to UB-612 measured by ELISpot and flow cytometric assays on Day 1, 15 and 85
- GMT of neutralizing antibody against SARS-CoV-2 variants on Day 1, 15, 85 using pseudovirus neutralizing antibody assay
- **The comparison between primary series and booster vaccination on the humoral immune response by comparing the Day 15 GMT and GMFI of neutralizing antibody (Wuhan strain) and antigen-specific antibody (Anti-S1-RBD) after booster dose to the GMT and GMFI 2 weeks after the second dose of the primary vaccination series (i.e. Day 42 of V-122 Phase 1).**
- **The comparison between primary series and booster vaccination on the humoral immune response by comparing the Day 85 GMT and GMFI of neutralizing antibody (Wuhan strain) and antigen-specific antibody (Anti-S1-RBD) after booster dose to the GMT and GMFI 3 months after the second dose of the primary vaccination series (i.e. Day 112 of V-122 Phase 1).**
- **Figures (dot-plot) to show the individual responses and GMT of neutralizing antibody (Wuhan strain) and antigen-specific antibody (Anti-S1-RBD) at each time point of V-122 Phase 1 and V-123 Phase 1**

extension studies in order to provide a comprehensive view of the kinetics of the primary and booster immune responses.

- **Figures (dot-plot) of individual prime-boost interval with immune responses to show 1) individual fold increase of Day 15 value of neutralizing antibody (Wuhan strain) compared with baseline of V-122 Phase 1 study, 2) individual fold increase of Day 15 value of neutralizing antibody (Wuhan strain) compared with baseline of this V-123 study, 3) individual fold increase of Day 15 value of antigen-specific antibody (Anti-S1-RBD) compared with baseline of V-122 Phase 1 study, 4) individual fold increase of Day 15 value of antigen-specific antibody (Anti-S1-RBD) compared with baseline of this V-123 study, 5) individual ELISpot IFN-gamma on Day 15.**

Sample Size Determination

A total of 60 subjects who had enrolled in V-122 phase I Study will be recruited.

Statistical Analysis

The Safety Set (SS) will consist of all subjects who completed two vaccinations in Phase I study enrolled into this extension study and received one booster dose of UB-612 vaccines with the same dose as Phase II study. The Safety Set is for safety evaluation in analysis.

The Full Analysis Set (FAS) will consist of subjects who completed two vaccinations in Phase I study enrolled into this extension study, received one booster dose of UB-612 vaccines with the same dose as Phase II study, and have at least one immunogenicity assessment on Day 15 or 85 after vaccination. Subjects receive prohibited medication/treatment/vaccine during pre-specified period has impact on immunogenicity may exclude from the FAS set.

The Per Protocol Set (PPS) will be a subset of FAS. PPS includes subjects who completed two vaccinations in Phase I study enrolled into this extension study, received one booster dose of UB-612 vaccines with the same dose as Phase II study, and were compliant to the protocol. Subjects who had major protocol deviations as determined by the Study Team or who received prohibited medication/treatment/vaccine during the pre-specified period leading to an impact on immunogenicity will be excluded from the PPS set. The PPS is also analysed for immunogenicity evaluation.

All safety assessments, including AEs, PEs, VS, and clinical laboratory evaluations, where indicated, will be presented using descriptive statistics for each vaccine group of UB-612. Data will be summarized for each vaccine group.

All adverse reactions within 7 days after vaccination will be summarized with numbers and percentages by study vaccine groups. The percentage of subjects with ≥ Grade 3 adverse events within 7 days after vaccination will also be demonstrated by study vaccine groups. AEs till Day 85 will be presented with number and percentage by system organ class, preferred term, and study vaccine groups. The number and percentage of SAEs during the whole follow-up period (3 months) will be presented in summary table by study vaccine groups. Adverse events of special interest during the study period will be summarized with number and percentage from the mapping result by MedDRA.

Changes of safety laboratory measures will be summarized with descriptive statistics by study vaccine group and each time point. ANCOVA model with laboratory baseline values as covariate will analyze changes of safety laboratory measures for testing the difference among vaccine groups. Pair-wise comparisons of least square means from the ANCOVA model will be presented. Intra-group difference in safety laboratory measures will also be analyzed by paired t test.

Geometric mean titer (GMT) will be described by descriptive statistics and the 95% confidence interval for study vaccine group. The difference on Day 1 among vaccine groups will be analyzed by ANOVA model under log-transform data. Additionally, the difference on Day 15 and 85 among vaccine groups will be analyzed by ANCOVA model under log-transform data with baseline level as covariate, if appropriate. Pair-wise comparisons of least square means from the ANOVA or ANCOVA model will be presented. The reverse cumulative distribution plot will be provided to display the distribution of titer by time points for each vaccine group.

Geometric mean fold increase (GMFI) in each study vaccine group is summarized by descriptive statistics and the 95% confidence interval for study vaccine group. The difference among vaccine groups will be analyzed by ANOVA. Pair-wise comparisons of least square means from the ANOVA model will be presented. The reverse cumulative distribution plot will be provided to display the distribution of fold increase by time points for each vaccine group.

For anti-S1-RBD ELISA, ACE2:RBD inhibition ELISA, and Neutralizing antibody titers, reverse cumulative distribution of titers and fold increases will be displayed at Day1, 15 and 85.

Neutralizing antibody GMT and GMFI will be determined against at least the following SARS-CoV-2 variants: Alpha (B.1.1.7), Beta (B.1.351), Gamma (P.1), and Delta (B.1.617.2). The fold decrease between titers of the original (Wuhan) strain and each variant will be determined, and compared using descriptive statistics.

In addition to the evaluations of the post-boost response, an analysis will be performed comparing the Day 15 GMT and GMFI after booster dose to the GMT and GMFI 2 weeks after the second dose of the primary vaccination series (i.e. Day 42 of V-122 Phase 1). Similarly, a second analysis will be performed comparing the Day 85 GMT and GMFI after booster dose to the GMT and GMFI 3 months after the second dose of the primary vaccination series (i.e. Day 112 of V-122 Phase 1).

For each vaccine group, figures (dot-plot) will be generated showing the individual responses and GMT at each time point of V-122 Phase 1 and V-123 Phase 1 extension studies in order to provide a comprehensive view of the kinetics of the primary and booster immune responses. Separate figures will show the three primary vaccination dose groups with the booster response.

The prime-booster interval is defined as the interval between of the second dose of primary vaccination series and the booster dose.

For each vaccine group, figures (dot-plot) of individual prime-boost interval with immune responses will be generated to show 1) individual fold increase of Day 15 value of neutralizing antibody (Wuhan strain) compared with baseline of V-122 Phase 1 study, 2) individual fold increase of Day 15 value of neutralizing antibody (Wuhan strain) compared with baseline of this V-123 study, 3) individual fold increase of Day 15 value of antigen-specific antibody (Anti-S1-RBD) compared with baseline of V-122 Phase 1 study, 4) individual fold increase of Day 15 value of antigen-specific antibody (Anti-S1-RBD) compared with baseline of this V-123 study, 5) individual ELISpot IFN-gamma on Day 15.

Safety Evaluations

Each subject must be carefully monitored for the development of any AEs throughout study period. Safety evaluations will be based on changes in physical examinations, vital signs and changes in laboratory parameters, and adverse event, including subject self-reporting.

All SARS-CoV-2 infections occurred within 3 months after the vaccination should be documented and reported following the same procedure of SAE reporting, and it is necessary to conduct a case investigation. Furthermore, the critically ill or dead cases need to continue to conduct special investigation, mainly to analyze where there is an ADE or VAERD phenomenon.

Schedule of Assessments

**Scheduled visit**	**1**	**2**	**3/ET**
**Test & observations**	**Booster vaccination** [d]		**Follow-up** [e]
Day	1	15<br>±3 days	85<br>±5 days
Informed consent	X		
Inclusion/Exclusion Criteria	X		
Demographics	X		
Medical history	X		
Physical Exam [a]	X	X	X
Vital sign	X	X	X
**Lab (Safety)**			
Blood routine	X	X	
Biochemistry	X	X	
Pregnancy [b]	X		X
**Lab (Immunogenicity)**			
Humoral immune response	X	X	X
Cellular immune response	X	X	X
Lab (SARS-CoV-2 test)			
Serum antibodies (IgG) against SARS-CoV-2[f]	X		X
Vaccination	X		
e-diary card instruction	X		
AEs/SAEs	X	X	X
COVID-19 infection surveillance		X	X
Concomitant Medication	X	X	X [c]

a: Body weight and height will be assessed at Visit 1 only.

b: Screening for pregnancy will be performed before vaccination. It is not required for postmenopausal or surgically sterilized women. When positive urine pregnancy test is presented, it should be confirmed by a serum β-HCG test. Serum testing in lieu of urine tests will not be considered a protocol deviation.

c: Only record medication for SAE and AESI.

d: The day is at least 6 months after **completing the primary vaccination series** in V-122 study.

e: 3 months after booster vaccination.

f: Serum antibodies (IgG) against SARS-CoV-2 will be assessed by UBI SARS-CoV-2 ELISA kit.

ET: early termination

Blood collection at scheduled visits

**Scheduled visit**	**1**	**2**	**3**
**Test & observations**	**Booster vaccination**		**Follow-up**
**Day**	**1**	**15±3**	**85±5**
	**Blood volume (mL)**		
**Lab (Safety)**			
Blood routine	4.5	4.5	
Biochemistry	2.5	2.5	
**Lab (Immunogenicity)**			
Humoral immune response	10	10	10
Cellular immune response	56	56	56
**Lab (SARS-CoV-2 test)**			
Serum antibodies (IgG) against SARS-CoV-2	2		2
Total blood collection	75	73	68

Total amount of blood collection: 216 mL.
* If HLA genotyping will be performed, additional 3 mL of blood sample will be collected.

3 TABLE OF CONTENTS

1 Protocol Approval Signatures ... 2

2 Synopsis ... 3

3 Table of Contents ... 10

4 List of Abbreviations ... 14

5 Introduction ... 15

5.1 Study Rationale ... 15

5.2 Background ... 15

5.3 UB-612 COVID-19 Vaccine ... 16

5.4 Nonclinical Study for Booster Dose ... 16

5.5 The Summary of Phase I Study ... 20

5.6 Risk/Benefit Assessment ... 23

6 Study Objectives & Endpoints ... 24

6.1 Primary Objective ... 24

6.2 Primary Endpoints ... 24

6.3 Secondary Objective ... 24

6.4 Secondary Endpoints ... 24

6.5 Exploratory Objective ... 24

6.6 Exploratory Endpoints ... 25

7 Investigational Plan ... 25

7.1 Overall Study Design and Plan: Description ... 25

7.2 Schedule of Assessments ... 27

7.3 Discussion of Study Design ... 29

7.4 Selection of Study Population ... 29

7.4.1 Number of Planned Subjects ... 29
7.4.2 Inclusion Criteria ... 29
7.4.3 Exclusion Criteria ... 30
7.4.4 Removal of Subjects ... 30
7.4.4.1 Removal of Subjects from Immunogenicity Analysis ... 30
7.4.4.2 Removal of Subjects from the Study ... 30
7.4.4.3 Study Termination ... 31
7.4.4.4 Reporting and Follow-up of Pregnancies ... 31

7.4.4.5 SARS-CoV-2 infection 32

7.5 Investigational Products 32

7.5.1 Identity of Investigational Products 32
7.5.2 Investigational Products Administered 32
7.5.2.1 Injection Volume(s) 32
7.5.2.2 Injection Route and Rate 33
7.5.2.3 Emergency Event Management 33
7.5.3 Packaging and Labeling 33
7.5.4 Prior and Concomitant Therapy 33
7.5.4.1 Prohibited Medication/Therapy 33

8 Timing of Study Procedures 34

8.1 Visit 1 (Day 1) – Screening and booster vaccination 34

8.2 Visit 2 (Day 15 ± 3 day) 34

8.3 Visit 3 (Day 85 ± 5 days) –Month 3 Follow-up /End of Study 35

8.4 Early Termination (ET) 35

8.5 Unscheduled Visit 35

8.6 Duration of Treatment 35

9 Immunogenicity and Safety Variables 36

9.1 Informed Consent Form 36

9.2 Demographics / Other Baseline Characteristics 36

9.3 Eligibility 36

9.4 Administration 36

9.5 Safety and Immunogenicity Measurements Assessed 37

9.5.1 Safety Variables 37
9.5.1.1 Physical Examination 37
9.5.1.2 Vital Sign 37
9.5.2 Clinical Laboratory Evaluation 37
9.5.3 Pregnancy Test 37
9.5.4 Self-Evaluation/Reporting (Solicited Symptoms) 38
9.5.4.1 Solicited Symptoms 38
9.5.4.2 Unsolicited Adverse Event 40
9.5.5 Adverse events (AEs) 40
9.5.5.1 Definition of Adverse Events (AE) 40
9.5.5.2 Definition of Serious Adverse Events (SAE) 41
9.5.5.3 Definition of Unexpected Adverse Reaction Definition 42
9.5.5.4 Definition of Medically Attended Adverse Event 42
9.5.5.5 Definition of Adverse Events Following Immunization (AEFI) [12] 43
9.5.5.6 Definition of Adverse Events of Special Interest (AESI) [12] 43

9.5.5.7 Assessment of Severity 44
9.5.5.8 The Relationship to Study Vaccine 45
9.5.5.9 Adverse Events Reporting 46
9.5.5.10 Human leukocyte antigen (HLA)-genotyping 47
9.5.6 Immunogenicity Assessments 47
9.5.6.1 Detection of ELISA Antibodies against S1-RBD of SARS-CoV-2 47
9.5.6.2 Detection of Antibody Titers Which Inhibit S1-RBD:ACE2 binding 47
9.5.6.3 Neutralizing Antibody Titers against SARS-CoV-2 47
9.5.6.4 Neutralizing Antibody Titers against SARS-CoV-2 variants using pseudovirus neutralizing antibody assay 47
9.5.6.5 Detection of T Cell Response 48
9.5.7 SARS-CoV-2 test 48
9.5.8 Overall Study Stopping Rules 48
9.5.9 Appropriateness of Measurements 49

9.6 Surveillance and Laboratory Diagnosis of SARS-CoV-2 Infection during Clinical Trial 49

10 Statistical Methods 50

10.1 Statistical and Analytical Plans 50

10.2 Datasets or Populations Analysed 50

10.3 Demographic and Other Baseline Characteristics 50

10.4 Safety Evaluation 51

10.4.1 Analysis of Primary Safety Endpoint 51
10.4.2 Analysis of Secondary Safety Endpoint 52

10.5 Immunogenicity Evaluation 52

10.5.1 Analysis of Primary Immunogenicity Endpoints 52
10.5.2 Analysis of Secondary Immunogenicity Endpoints 53
10.5.3 Analysis of Exploratory Immunogenicity Endpoints 54

10.6 Interim Analysis 55

10.7 Handling of Missing Data 56

10.8 Determination of Sample Size 56

10.9 Protocol Deviations 57

11 Quality Assurance and Quality Control 58

11.1 Audit and Inspection 58

11.2 Monitoring 58

11.3 Data Management and Coding 58

12 Records and Supplies 59

12.1 Drug Accountability 59
12.2 Financing and Insurance 59
13 Ethics 60
13.1 Independent Ethics Committee or Institutional Review Board 60
13.2 Regulatory Authorities 60
13.3 Ethical Conduct of the Study 60
13.4 Informed Consent 60
13.5 Subject Confidentiality 61
14 Reporting and Publication, Including Archiving 62
15 References 63

4 LIST OF ABBREVIATIONS

ACE2	Angiotensin-converting enzyme 2
ADE	Antibody dependent enhancement (of viral replication)
AE	adverse event
AESI	adverse event of special interest
ALT	alanine aminotransferase
AST	aspartate aminotransferase
β-HCG	beta-human chorionic gonadotropin
BP	blood pressure
BUN	blood urea nitrogen
CBC	complete blood count
cGCP	current good clinical practice
CHO	Chinese hamster ovary
CRF	case report form
FDA	Food and Drug Administration
**GMFI**	**geometric mean fold increase**
**GM**T	**geometric mean titer**
HCT	hematocrit
HLA	human leukocyte antigen
HR	heart rate
ICF	informed consent form
ICH	International Council for Harmonisation
IEC	independent ethics committee
IM	intramuscular
IRB	institutional review board
N	number of subjects in the dataset or population
N/A	not applicable
ORF	open reading frame
PE	physical examination
RBD	receptor binding domain
RR	respiratory rate
SCR	seroconversion rate
SAE	serious adverse event
SUSAR	suspected unexpected serious adverse reaction
UB-612	United Biomedical's COVID-19 vaccine
VAERD	vaccine-associated enhanced respiratory disease
**VOC**	**variant of concern**
VS	vital signs
WBC	white blood cell
WoCBP	woman/women of childbearing potential

5 INTRODUCTION

5.1 Study Rationale

US healthcare regulator the Food and Drug Administration (FDA) has updated its guidance to help vaccine and therapeutics companies to address the emergence of new variants of SARS-CoV-2, the virus that causes Covid-19 [1]. Over the past few months, multiple SARS-CoV-2 variants have been emerging across the world. This has created concerns that drugs and vaccines in development and authorised for Covid-19 will no longer be effective against the viral disease. For vaccines, the FDA has updated its October 2020 guidance to support companies in amending their emergency use authorisations. The guidance informs companies of the additional data needed for modified vaccines to tackle the viral variants. It also notes that a modified vaccine must be “clearly distinguished” from the original prototype vaccine. The guidance also allows for booster studies to be carried out. This is where the modified vaccine is administered to people who have received the original prototype vaccine. Another version is where a booster of the original prototype is trialed to see if it protects against the new variants, which is the rationale for this present booster study.

The Advisory Committee on Immunization Practice (ACIP) updated their “Interim Clinical Considerations for Use of COVID-19 Vaccines Currently Authorized in the United States” according to the emerging variant virus. Regarding to booster doses, they only stated that the need for and timing for COVID-19 booster doses have not been established. No additional doses are recommended at this time. Therefore, all possibilities that render higher or keep high neutralization antibody titer regimen and/or schedule should be designed and tested based on the needs.

A rise in breakthrough COVID-19 infections — those happening in people who are vaccinated, but who still get infected when exposed to the virus — would be a warning sign. Thus, to see those who were full vaccinated and will travel to high risk area, if the booster shot, 2 weeks before departure, can render the immunity to against the variant of concern is great of value.

5.2 Background

In December 2019, a cluster of patients with pneumonia surfaced in Wuhan, China. The culprit was quickly identified as a beta-coronavirus that has never been reported before, and the disease was named by WHO as Corona Virus Disease 2019 (COVID-19) and the virus that causes it by the International Committee on Taxonomy of Viruses (ICTV) as SARS-CoV-2 [2, 3]. As of May 2021, a global outbreak has caused over 150 million confirmed cases in more than 220 countries or territories, with over 3 million deaths, making the SARS-CoV-2 pandemic a general public health event that has stirred up worldwide attention. Development of effective vaccines that prevent disease, viral shedding and transmission is urgently needed. At least 14 vaccines are now approved in one or more countries, but vaccine coverage is still in its infancy.

SARS-CoV-2 is a positive-strand RNA virus that belongs to the group of Betacoronaviruses. The genome of SARS-CoV-2 is approximately 29,700 nucleotides long and shares 79.5% sequence identity with SARS-CoV [4]. The long ORF1ab polyprotein at 5’ end of the genome encodes 15 or 16 non-structural proteins, and the 3’ end encodes 4 major structural proteins, including the spike (S) protein, nucleocapsid (N) protein, membrane (M) protein, and the envelope (E) protein [5]. SARS-CoV-2 interacts with the receptor angiotensin converting enzyme 2 (ACE2) on host cells via receptor binding domain (RBD) of its S protein for viral entry and subsequent

pathogenesis [6], resulting in severe respiratory illness with symptoms of fever, cough, and shortness of breath, and even death in severe cases [7].

Vaccines are the most effective and economical means to prevent and control infectious diseases [8]. The development and distribution of effective vaccines against SARS-Co-2 infection are urgently required. Currently, multiple vaccines have demonstrated efficacy in randomized controlled trials, and global vaccinations campaigns are underway under Emergency Use Authorizations. However, Johns Hopkins University estimates on ~17% of the world population has received primary immunization against SARS-CoV-2. This shortfall in actual vaccinations underscores the need for additional vaccines [8].

Safety is the most important issue that should be taken into consideration during drug and vaccine development, and some scientists urge that we should not rush to deploy COVID-19 vaccines and drugs without sufficient safety guarantees [9]. There have been concerns regarding vaccine-associated enhanced respiratory disease (VAERD) by certain candidate COVID-19 vaccine approaches, via antibody-dependent enhancement (ADE) or development of Th2 immunopathology [10]. Grifoni *et al.* [11] revealed predominant Th1 responses in convalescing COVID-19 cases, with little to no Th2 cytokines. Clearly more studies are required, but the data Grifoni *et al.* shown appear to predominantly represent a classic Th1 response to SARS-CoV-2.

The emergence of SARS-CoV-2 variants with mutations in the spike protein has raised concern due to their increased rates of transmission and their potential of resistance to immunity elicited by natural infection or vaccination. Participants in Phase 1 study (V-122) who previously vaccinated with 2 doses of UB-612 will be enrolled in an extension study. This open-labeled Phase 1 extension study is to evaluate the potential of a booster dose of UB-612 to elicit high titers of neutralizing antibodies that may counteract the decrease in neutralizing activity against variants of concern (VOC).

5.3 UB-612 COVID-19 Vaccine

United Biomedical (UBI) has developed a vaccine candidate against SARS-CoV-2 that is designed to activate both humoral and cellular responses. For SARS-CoV-2 immunogens, UB-612 includes a designer S1-RBD-sFc (SRsFc) fusion protein formulated with designer Th and CTL epitope peptides selected from immunodominant M, S2 and N regions known to bind to human MHC I and II. This mixture of designer Th/CTL peptides is designed to elicit T cell activation, memory recall and effector functions similar to that of natural COVID-19. The S1-RBD-sFc fusion protein incorporates both linear and conformation epitopes and induces high affinity antibodies to the RBD of SARS-CoV-2. The immunogen components are formulated with an oligonucleotide containing unmethylated CpG motifs and Adju-Phos® adjuvants, which promotes the activation of antigen-presenting cells pathways to induce an optimal immunogenicity profile and achieve the prevention purpose.

In summary, UB-612 vaccine design composition (S1-RBD-sFC+Th and CTL peptides+CpG, formulated with Adju-Phos®) is expected not only to be safe and inducing high titers of neutralizing antibodies, but also to provide T cell memory for a long lasting protection against COVID-19 across all human subjects irrespective of age, sex and ethnicities.

5.4 Nonclinical Study for Booster Dose

A booster dose induces high levels of neutralizing antibodies in rhesus macaques (RM)

Four groups of young adult animals (n = 2 males and 2 females /group) were assigned to receive 10, 30 or 100 µg of UB-612 or saline IM in 0.5mL in on day 0 and Day 28. A booster dose was given on Day 70 (**Figure 5-1(a)**).

Neutralizing antibody measurements were performed using replicating SARS-CoV-2 virus (CPE assay). Neutralizing antibodies against live SARS-CoV-2 increased to high levels after the 2nd dose of study vaccine in all dose groups (**Figure 5-1(b)**). Notably, the virus neutralizing titers (VNT_{100}) were significantly increased 4 - 8-fold after the Day 70 booster dose, which suggests that a booster dose could be effective to enhance the neutralizing activity of antibodies against SARS-CoV-2.

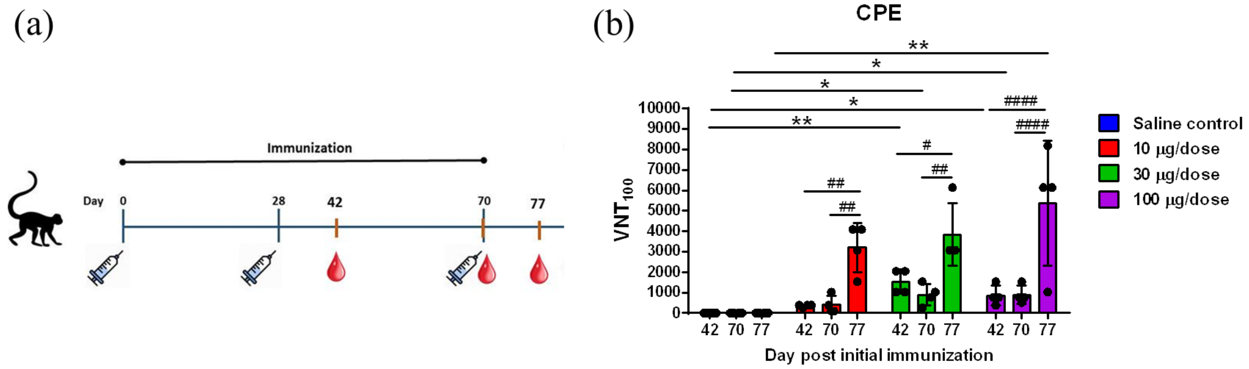

Figure 5-1. Potent neutralization against live SARS-CoV-2 by immune sera from UB-612-immunized rhesus macaques. (a) Study design of immunogenicity assessment in rhesus macaques receiving UB-612. Rhesus macaques were immunized with 10, 30 or 100 µg of UB-612 or saline (n = 4, 2 females and 2 males each group) at Days 0, 28, and 70 via intramuscular route. Humoral and cellular immune responses induced by UB-612 were evaluated in different time points as indicated. **(b)** Immune sera collected at Days 42, 70, and 77 from vaccinated rhesus macaques were analyzed on the SARS-CoV-2 infected Vero-E6 cells for cytopathic effect (CPE) assay. Virus neutralization titers VNT_{100} (100% Inhibitory Dilution Fold against viral infection) for immune sera were expressed as mean ± SD (n = 4). Each symbol represents one individual animal. * p ≦ 0.05, ** p ≦ 0.01 for comparison between groups at the same time point (Kruskal-Wallis ANOVA with Dunn's multiple comparisons test). # p ≦ 0.05, ## p ≦ 0.01, #### p ≦ 0.0001 for comparison within group at different time points (2-way ANOVA with Tukey's multiple comparisons test).

Neutralization response against SARS-CoV-2 variants in RM

To assess the ability of UB-612 to elicit neutralizing antibodies against the new SARS-CoV-2 variants, sera collected from vaccinated monkeys at Day 42, 70 and 77 were subjected to measure neutralization titer by SARS-CoV-2 variants pseudovirus assay (**Figure 5-2**). With this pseudovirus assay, there was a 1.7-1.8-fold decrease in GMT against the B.1.1.7 (UK), 9.5-9.6-fold decrease in GMT against the B.1.351 (SA), 1.8-2.1-fold decrease in GMT against the P.1 (BR), 1.1-1.8-fold decrease in GMT against the B.1.429 (CA), and 3.8-5.0-fold decrease in GMT against the B.1.526 (NY) for monkeys immunized with 30 µg or 100 µg UB-612 at Day 42. Notably, a booster dose of UB-612 increased the neutralizing titer response against SARS-CoV-2 that counteracted the decrease in neutralizing activity against variants (**Figure 5-3**).

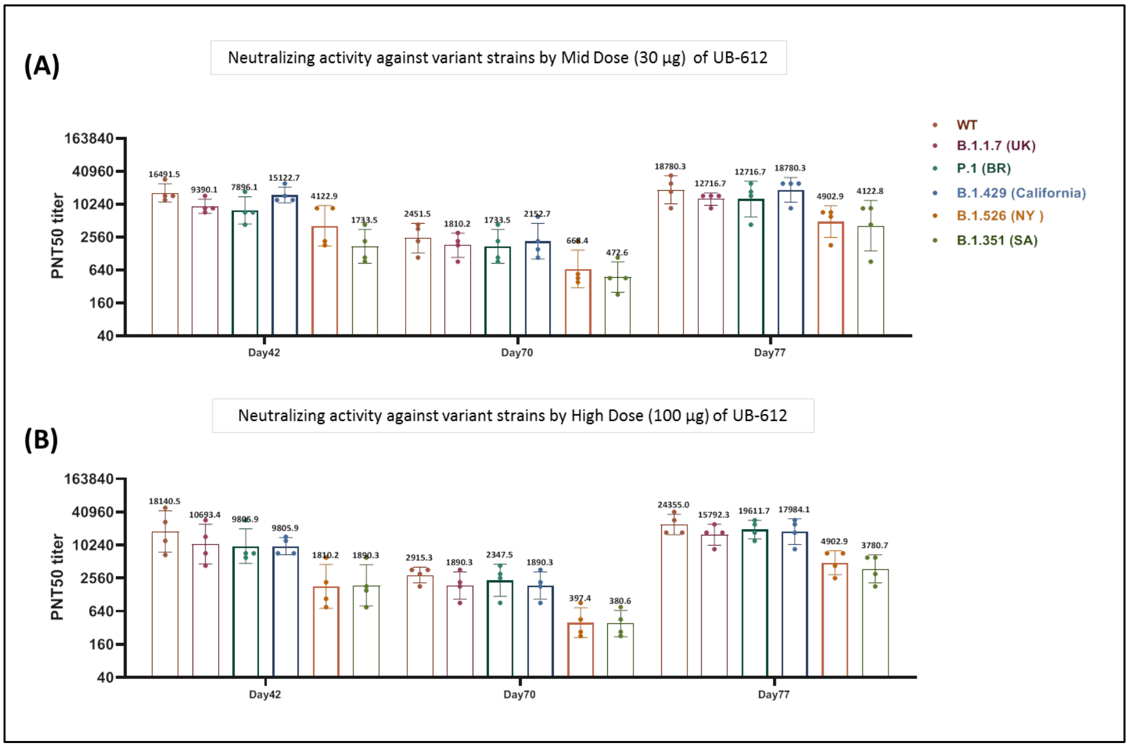

Figure 5-2. Ability of UB-612 immune sera from RM to neutralize SARS-CoV-2 pseudoviruses bearing wild-type or variant spike proteins. Rhesus macaques were immunized with 30 or 100 µg of UB-612 on Days 0, 28, and 70, and immune sera were collected at Days 42, 70, and 77. Neutralization was measured by a recombinant VSV-based SARS-CoV-2 pseudovirus neutralization assay incorporating full-length spike protein from TW_CDC#4 (Wuhan), B.1.1.7 (UK, United Kingdom), B.1.351 (SA, South Africa), P.1 (BZ, Brazil), B.1.429 (CA, California), or B.1.526 (NY, New York) variants. Virus neutralization titers PNT_{50} for immune sera were expressed as geometric mean titer ± geometric SD (n = 4). Each symbol represents one individual animal.

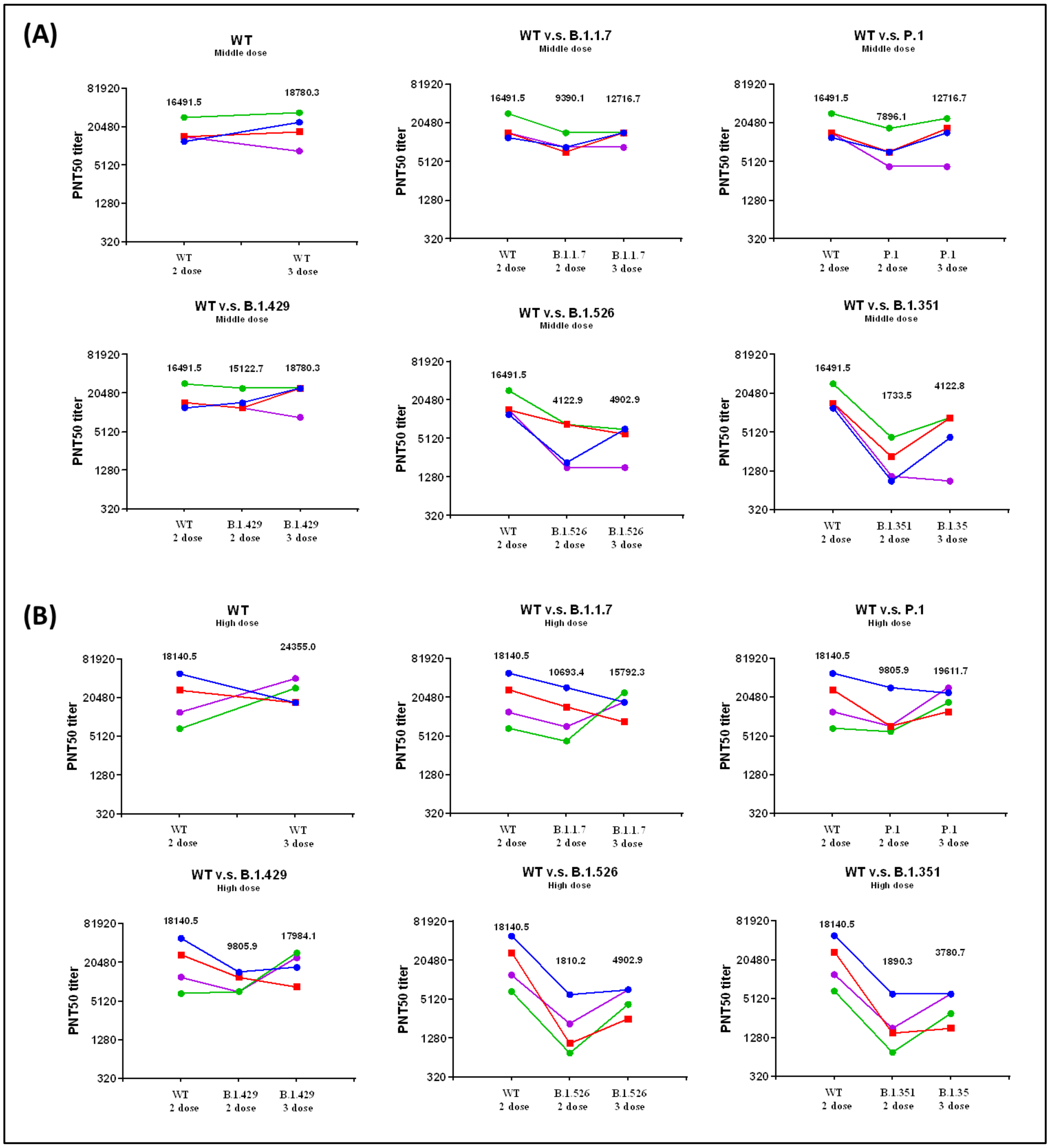

Figure 5-3. Neutralization of RM sera against SARS-CoV-2 VOC in the VSV-based pseudovirus neutralization assay. Rhesus macaques were immunized with 30 µg (A) or 100 µg (B) of UB-612 on Days 0, 28, and 70, and sera were collected on Days 42 and 77. Neutralization against indicated VOC was measured in pseudovirus neutralization assay. Results from individual animal are represented as dots on each figure, with lines connecting the WT and variant neutralization titers. The values represent the geometric mean titers (n = 4). Each symbol represents one individual animal.

5.5 The Summary of Phase I Study

In the single-centre, open-label, dose-escalating phase I clinical trial, three different dose regimens [prime-boost (P/B) immunization with dosage of 10/10, 30/30 and 100/100 μg] of UB-612 will be tested in healthy adult volunteers between 20 and 55 years of age. Twenty subjects were enrolled into one of the two vaccination regimen cohorts and received two intramuscular injections of UB-612 at a 28-day interval (Day 0 and Day 28). Safety monitoring reviews were held to decide if the study could go on to second immunization for each cohort or to the higher dose regimens.

For safety and tolerability assessments, solicited AEs (collected by AE e-diary) and unsolicited AEs (from on-site/laboratory examination and subject spontaneous reporting) were followed throughout the study period. Levels of anti-S1-RBD antibodies, neutralizing antibodies, and cellular immune responses were examined after each vaccination for immunogenicity and pharmacodynamic evaluations.

In the safety profile, the date of data-cut on 2021-05-13, the results indicated that administration of UB-612 vaccine was safe and tolerable across the dose levels. Almost all subjects, except one subject in B group, complete the study visit in Day 112, the 3 months after second vaccination, and most of subjects (59 of 60) completed study procedures in Day 196.

1. A total of 65.0% subjects receiving UB-612 vaccine 10 μg, 70.0% subjects receiving UB-612 vaccine 30 μg, and 80.0% subjects receiving UB-612 vaccine 100 μg had any adverse reactions within 7 days after first vaccination. Any adverse reactions within 7 days after second vaccination were reported in 55.0% subjects receiving UB-612 vaccine 10 μg, 60.0% subjects receiving UB-612 vaccine 30 μg and 35.0% subjects receiving UB-612 vaccine 100 μg.

2. There were 2 subjects (10.0%) in UB-612 vaccine 10 μg group, and 1 subject (5.0%) in UB-612 vaccine 30 μg group reported ≥ Grade 3 adverse events within 7 days after first vaccination, while no any ≥ grade 3 adverse events within 7 days after second vaccination among three dose cohorts occurred. All 3 were reports of increased diastolic blood pressure and occurred in subjects with pre-existing Grade 2 hypertension (Grade 2 was based on CTCAE version 5.0 at baseline); the events were assessed as unrelated to study vaccine.

3. Unsolicited adverse events were reported by 85.0% subjects in UB-612 vaccine 10 μg group, 60.0% subjects in UB-612 vaccine 30 μg group, and 35.0% subjects in UB-612 vaccine 100 μg group. Most of unsolicited adverse events was judged as Grade 1 and 2. Blood pressure diastolic increased in 2 subjects and blood pressure ambulatory increased in 1 subject in UB-612 vaccine 10 μg group, and blood pressure diastolic increased in 1 subject in UB-612 vaccine 30 μg group, were reported as Grade 3. No ≥ Grade 3 unsolicited adverse events was found in UB-612 vaccine 100 μg group.

4. Due to procedural issues, some local reactions at the vaccination sites were recorded in the AE eCRF as unsolicited events and were not reported in the subject e-diary as solicited events. The related-vaccine unsolicited AEs were found in 50.0%, 15.0%, and 5.0% subjects in UB-612 vaccine 10, 30 and 100 μg group, respectively. The frequently related unsolicited adverse events related to vaccine were injection site reaction (6 subjects, 30.0%) and malaise (5 subjects, 25.0%) in UB-612 vaccine 10 μg group. The injection site rash, alanine

aminotransferase increased, haemoglobin decreased, which were reported as the related unsolicited adverse events in each 1 subjects in UB-612 vaccine 30 μg group. Constipation in 1 subjects was judged as related unsolicited adverse events in UB-612 vaccine 100 μg group.

5. No deaths, and no serious adverse events reported was found in this study till now. No clinically significant change in the physical examination or ECG measurement in this trial was noted.

The immune responses in antigen-specific antibody and neutralizing antibody could be observed at Day 28 and could achieve 4-fold increase in the majority of 10, 30, and 100 μg vaccine group at Day 42 and 56.

1. All subjects in UB-612 vaccine 10, 30 and 100 μg group revealed no detection in antigen-specific antibody before vaccination. In UB-612 vaccine 10 μg group, GMT increased to 350.79 at Day 42 (14 days after second vaccination) and 526.98 at Day 56 (28 days after second vaccination). In UB-612 vaccine 30 μg group, GMT increased to 366.80 at Day 42 and 269.06 at Day 56. In UB-612 vaccine 100 μg group, GMT increased to 2240.15 at Day 42 and 805.11 at Day 56.

2. SCR of antigen-specific antibody was observed in 90.0% participants receiving UB-612 vaccine 10 μg, 90.0% participants receiving UB-612 vaccine 30 μg, and 100% receiving UB-612 vaccine 100 μg at Day 42. SCR of antigen-specific antibody was observed in 95.0% participants receiving UB-612 vaccine 10 μg, 95.0% participants receiving UB-612 vaccine 30 μg, and 100% receiving UB-612 vaccine 100 μg at Day 56.

3. All subjects revealed no detection in neutralizing antibody before vaccination. In subjects receiving UB-612 vaccine 10 μg, the GMT of neutralizing antibody responses increased to 44.45 at Day 42 and 41.92 at Day 56. In subjects receiving UB-612 vaccine 30 μg, the GMT of neutralizing antibody responses increased to 31.29 at Day 42 and 28.99 at Day 56. In subjects receiving UB-612 vaccine 100 μg, the GMT of neutralizing antibody responses increased to 107.66 at Day 42 and 102.97 at Day 56.

4. SCR of neutralizing antibody was observed in 85.0% participants receiving UB-612 vaccine 10 μg, 85.0% participants receiving UB-612 vaccine 30 μg, and 100% receiving UB-612 vaccine 100 μg at Day 42. SCR of neutralizing antibody was observed in 90.0% participants receiving UB-612 vaccine 10 μg, 80.0% participants receiving UB-612 vaccine 30 μg, and 100% receiving UB-612 vaccine 100 μg at Day 56.

Table 5-1 Geometric Mean Titer of Antigen-Specific Antibody and Neutralizing Antibody

Visit		UB-612 vaccine 10 µg (N = 20)	UB-612 vaccine 30 µg (N = 20)	UB-612 vaccine 100 µg (N = 20)
**Antigen-Specific Antibody by S1-RBD ELISA**				
Day 0	n	20	20	20
	GMT	10.00	10.00	10.00
	95% CI	--	--	--
Day 42	n	20	20	20
	GMT	350.79	366.80	2240.15
	95% CI	(161.614, 761.410)	(187.085, 719.139)	(1233.797, 4067.353)
Day 56	n	20	20	20
	GMT	526.98	269.06	805.11
	95% CI	(269.663, 1029.839)	(157.931, 458.381)	(598.880, 1082.350)
**Neutralizing Antibody versus Parenteral Strain**				
Day 0	n	20	20	20
	GMT	2.50	2.50	2.50
	95% CI	--	--	--
Day 42	n	20	20	20
	GMT	44.45	31.29	107.66
	95% CI	(23.017, 85.821)	(17.159, 57.060)	(72.402, 160.087)
Day 56	n	20	20	20
	GMT	41.92	28.99	102.97
	95% CI	(24.020, 73.146)	(19.101, 43.991)	(70.764, 149.836)

Table 5-2 Seroconversion Rate (SCR) of Antigen-Specific Antibody and Neutralizing Antibody

Visit		UB-612 vaccine 10 µg (N = 20)	UB-612 vaccine 30 µg (N = 20)	UB-612 vaccine 100 µg (N = 20)
**Antigen-Specific Antibody by S1-RBD ELISA**				
Day 42	n	20	20	20
	SCR	18 (90.0%)	18 (90.0%)	20 (100%)
	95% Exact CI	(68.30%, 98.77%)	(68.30%, 98.77%)	(83.16%, 100%)
Day 56	n	20	20	20
	SCR	19 (95.0%)	19 (95.0%)	20 (100%)
	95% Exact CI	(75.13%, 99.87%)	(75.13%, 99.87%)	(83.16%, 100%)
**Neutralizing Antibody versus Parenteral Strain**				
Day 42	n	20	20	20
	SCR	17 (85.0%)	17 (85.0%)	20 (100%)
	95% Exact CI	(62.11%, 96.79%)	(62.11%, 96.79%)	(83.16%, 100%)
Day 56	n	20	20	20
	SCR	18 (90.0%)	16 (80.0%)	20 (100%)
	95% Exact CI	(68.30%, 98.77%)	(56.34%, 94.27%)	(83.16%, 100%)

In conclusion, the 100 µg vaccine group showed a highest improvement on antigen-specific antibody and neutralizing antibody among three vaccine groups with comparable safety profile. In a previous Phase II study, participants vaccinated with UB-612 have been offered 100 µg dose. The Phase II trial is still ongoing.

5.6 Risk/Benefit Assessment

Based on the promising data in the phase I study, we observed no significant safety signals after administration of 2 doses of vaccine. Given the substantial increase in neutralizing antibodies against variants of concern conferred by a single booster dose. We aimed to further evaluate the safety, tolerability and immunogenicity of booster vaccination of UB-612 vaccine in healthy adult volunteers in phase I extension study. In this study, the subjects who completed two vaccinations in Phase I study will receive one booster dose of UB-612 vaccines 100 μg, the same dose which was offered in Phase II study, at least 6 months after **completing the 2-dose primary vaccination series.**

6 STUDY OBJECTIVES & ENDPOINTS

6.1 Primary Objective

- To evaluate the safety and tolerability of a booster dose of UB-612 vaccine in Phase I subjects.
- To evaluate the SARS-CoV-2 neutralizing antibody titers induced by a booster dose of UB-612.

6.2 Primary Endpoints

Immunogenicity Endpoint(s)

- Geometric mean titer (GMT) of neutralizing antibody against SARS-CoV-2 **(Wuhan strain)** on Day 1, 15 and 85
- Geometric mean fold increase **(GMFI)** of neutralizing antibody against SARS-CoV-2 **(Wuhan strain)** on Day 15 and 85

Safety Endpoint(s)

- Occurrence of adverse reactions within 7 days after vaccination
- The percentage of subjects with ≥ Grade 3 adverse events within 7 days after vaccination

6.3 Secondary Objective

To evaluate the humoral immune response to SARS-CoV-2 during this study.

6.4 Secondary Endpoints

Immunogenicity Endpoint(s)

- GMT of antigen-specific antibody (Anti-S1-RBD) on Day 1, 15 and 85
- Geometric mean fold increase of antigen-specific antibody (Anti-S1-RBD) on Day 15 and 85
- Distribution of titers
- Correlation between the immune response detected by ELISA and live virus neutralization test

Safety Endpoint(s)

- Occurrence of serious adverse events during the whole follow-up period (3 months)
- Occurrence of adverse events of special interests (AESIs), medically attend adverse events (MAAEs) and serious adverse events (SAEs) during the study period
- Changes of safety laboratory measures

6.5 Exploratory Objective

- To evaluate the cellular immune response induced by **a booster dose of** UB-612.

- To evaluate the neutralizing antibody titer against SARS-CoV-2 variants induced by **a booster dose of** UB-612.
- **To compare primary series and booster vaccination of UB-612 on humoral immune responses.**
- **To evaluate the kinetics of humoral immune responses of primary series and booster vaccination**
- **To evaluate the effect of prime-boost interval on immune responses**

6.6 Exploratory Endpoints

- T cell responses to UB-612 measured by ELISpot and flow cytometric assays on Day 1, 15 and 85
- GMT of neutralizing antibody against SARS-CoV-2 variants on Day 1, 15, 85 using pseudovirus neutralizing antibody assay
- **The comparison between primary series and booster vaccination on the humoral immune response by comparing the Day 15 GMT and GMFI of neutralizing antibody (Wuhan strain) and antigen-specific antibody (Anti-S1-RBD) after booster dose to the GMT and GMFI 2 weeks after the second dose of the primary vaccination series (i.e. Day 42 of V-122 Phase 1).**
- **The comparison between primary series and booster vaccination on the humoral immune response by comparing the Day 85 GMT and GMFI of neutralizing antibody (Wuhan strain) and antigen-specific antibody (Anti-S1-RBD) after booster dose to the GMT and GMFI 3 months after the second dose of the primary vaccination series (i.e. Day 112 of V-122 Phase 1).**
- **Figures (dot-plot) to show the individual responses and GMT of neutralizing antibody (Wuhan strain) and antigen-specific antibody (Anti-S1-RBD) at each time point of V-122 Phase 1 and V-123 Phase 1 extension studies in order to provide a comprehensive view of the kinetics of the primary and booster immune responses.**
- **Figures (dot-plot) of individual prime-boost interval with immune responses to show 1) individual fold increase of Day 15 value of neutralizing antibody (Wuhan strain) compared with baseline of V-122 Phase 1 study, 2) individual fold increase of Day 15 value of neutralizing antibody (Wuhan strain) compared with baseline of this V-123 study, 3) individual fold increase of Day 15 value of antigen-specific antibody (Anti-S1-RBD) compared with baseline of V-122 Phase 1 study, 4) individual fold increase of Day 15 value of antigen-specific antibody (Anti-S1-RBD) compared with baseline of this V-123 study, 5) individual ELISpot IFN-gamma on Day 15.**

7 INVESTIGATIONAL PLAN

7.1 Overall Study Design and Plan: Description

This is an extension study to evaluate the safety, tolerability and immunogenicity of one booster dose of UB-612 COVID-19 vaccine in adults who completed two vaccinations of UB-612 vaccine 10, 30, and 100 μg in V-122 study (Phase I).

The subjects who completed two vaccinations in Phase I study will be recruited in this extension study. After the informed consent is obtained from the subject, eligible subjects will receive one booster dose of UB-612 vaccines 100 µg with the same dose which was offered in Phase II study, at least 6 months after **completing the 2-dose primary vaccination series**. This study will be carried out in three groups:

(1) A group: 1 booster dose of UB-612 vaccine 100 µg, for subjects at UB-612 vaccine 10 µg group previously

(2) B group: 1 booster dose of UB-612 vaccine 100 µg, for subjects at UB-612 vaccine 30 µg group previously

(3) C group: 1 booster dose of UB-612 vaccine 100 µg, for subjects at UB-612 vaccine 100 µg group previously

In this study, there will be 3 clinical visits. Subjects will come to the clinics at Visit 1 (Day 1, baseline, booster vaccination, at least 6 months after **completing the primary vaccination series** in V-122 study), Visit 2 (Day 15), and Visit 3 (Day 85, 3 months after booster vaccination).

In all groups, an informed consent and the eligibility of subjects will be checked at Visit 1(Day 1). The booster study vaccine administration will be done immediately after blood draw which will evaluate baseline laboratory safety and humoral / cellular immune response / SARS-CoV-2 test. All subjects in each group will be monitored at least 30 minutes after vaccination (for vital signs change and adverse events). The post-vaccination e-diary card for 7-day solicited adverse events after vaccination will be given to subjects with a suitable instruction.

At Visit 2 (Day 15), subjects will return to clinics and for laboratory safety check and have a blood drawn for humoral / cellular immune response.

At Visit 3 (Day 85, 3-month follow-up visit after booster vaccination), the subjects will return to clinics and have blood drawn for humoral immune / cellular immune response/ SARS-CoV-2 test

All adverse events and serious adverse events will be monitored from Visit 1 (Day 1) until Visit 3 (Day 85).

7.2 Schedule of Assessments

Below is a list of all study procedures through the study period and the signs "X" indicate when the procedures are performed.

**Scheduled visit**	**1**	**2**	**3/ET**
**Test & observations**	**Booster vaccination**[d]		**Follow-up** [e]
Day	1	15 ±3 days	85 ±5 days
Informed consent	X		
Inclusion/Exclusion Criteria	X		
Demographics	X		
Medical history	X		
Physical Exam [a]	X	X	X
Vital sign	X	X	X
**Lab (Safety)**			
Blood routine	X	X	
Biochemistry	X	X	
Pregnancy[b]	X		X
**Lab (Immunogenicity)**			
Humoral immune response	X	X	X
Cellular immune response	X	X	X
**Lab (SARS-CoV-2 test)**			
Serum antibodies (IgG) against SARS-CoV-2[f]	X		X
Vaccination	X		
e-diary instruction	X		
AEs/SAEs	X	X	X
COVID-19 infection surveillance		X	X
Concomitant Medication	X	X	X [c]

a: Body weight and height will be assessed at Visit 1 only.
b: Screening for pregnancy will be performed before vaccination. It is not required for postmenopausal or surgically sterilized women. When positive urine pregnancy test is presented, it should be confirmed by a serum β-HCG test. Serum testing in lieu of urine tests will not be considered a protocol deviation.
c: Only record medication for SAE and AESI.
d: The day is at least 6 months after **completing the primary vaccination series** in V-122 study.
e: 3 months after booster vaccination.
f: Serum antibodies (IgG) against SARS-CoV-2 will be assessed by UBI SARS-CoV-2 ELISA kit.
ET: early termination

Blood collection at scheduled visits

**Scheduled visit**	**1**	**2**	**3**
**Test & observations**	**Booster vaccination**		**Follow-up**
**Day**	**1**	**15±3**	**85±5**
	**Blood volume (mL)**		
**Lab (Safety)**			
Blood routine	4.5	4.5	
Biochemistry	2.5	2.5	
**Lab (Immunogenicity)**			
Humoral immune response	10	10	10
Cellular immune response	56	56	56
**Lab (SARS-CoV-2 test)**			
Serum antibodies (IgG) Against SARS-CoV-2	2		2
Total blood collection	75	73	68

Total amount of blood collection: 216 mL.
* If HLA genotyping will be performed, additional 3 mL of blood sample will be collected.

7.3 Discussion of Study Design

The objectives of this study are to evaluate the safety, tolerability, and immunogenicity of booster dose of UB-612 vaccine in Phase I subjects. The trial will be carried out with 3 dosing groups as the design of Phase I study. Eligible subjects will receive one booster dose of UB-612 vaccine 100 μg with the same dose which was offered in Phase II study, at least 6 months after **completing the 2-dose primary vaccination series**. All expected or unexpected vaccine-related adverse events will also be assessed through study period. The post-vaccination solicited AEs will be evaluated via e-diary, and other AE issues will be reported at each visit. The immunogenicity will be also assessed at 14 days after booster vaccination, and in the subsequent 3-month long-term visit for observing the persistence of immune responses. In this study, full immunogenicity/tolerability profile of one booster UB-612 vaccination will have expected to be established.

7.4 Selection of Study Population

7.4.1 Number of Planned Subjects

All subjects, up to 60 subjects, who completed two vaccinations in Phase I study will be enrolled into this extension study.

7.4.2 Inclusion Criteria

To be eligible for study entry subjects must satisfy all of the following inclusion criteria:

1. Male or non-pregnant female who previously participated in and completed two vaccinations in the V-122 Study.
2. Women of childbearing potential and men must agree to practice medically effective contraception during study period. The acceptable effective contraception methods include:
    a. Male or female sterilization, implant, or intrauterine device;
    b. Injectable, pill, patch, ring plus one barrier method*;
    c. Two combined barrier methods*.

    *Effective barrier methods are diaphragm, male or female condoms, sponge, or spermicides (creams or gels that contain a chemical to kill sperm).
3. Able to understand the content and possible risks of informed consent and willing to sign the Informed Consent Form (ICF).
4. Able to understand and agrees to comply with all study procedures and be available for all study visits.
5. Ear temperature ≤ 38.0°C.
6. At screening visit, at least 6 months after **completing the 2-dose primary vaccination series** in the V-122 study.

7.4.3 Exclusion Criteria

Subjects will be excluded from the study if one or more of the following exclusion criteria is applicable:

1. Female who is pregnant or positive in pregnancy test at screening visit.
2. Female who is breast-feeding or plans to breastfeed within 90 days after booster vaccination.
3. Any acute illness, as determined by the study investigator 3 days before booster vaccination.
4. Prior administration of attenuated, nucleic acid (mRNA or DNA) or vectored vaccines in last 1 month before booster vaccination or expectation of such vaccines in the month after booster vaccination.
5. Prior administration of subunit vaccine or inactivated vaccine in last 14 days before booster vaccination or expectation of receipt of such vaccines in the 14 days after booster vaccination.
6. Any medical disease or condition that, in the opinion of the study investigator, may confound the results of the study or pose an additional risk to the subjects by their participation in the study.
7. Previous exposure to SARS-CoV-2 or receipt of an investigational or EUA vaccine product for the prevention of COVID-19, MERS or SARS except UB-612.
8. Subjects who take part in another clinical study, other than V-122 study, within 12 weeks prior to the day of informed consent.
9. Prior administration of immunoglobulins and/or any blood products in last 4 months before booster vaccination.
10. Prior chronic administration (defined as ≥ 14 day of continuous use) of immunosuppressant or corticosteroids (equivalent to ≥ 20 mg daily of prednisone), cytotoxic treatment in last 6 months before first vaccination.

7.4.4 Removal of Subjects

7.4.4.1 Removal of Subjects from Immunogenicity Analysis

Subjects may withdraw from the immunogenicity analysis for any of the following reasons:

- Administration of prohibited vaccine/medication during pre-specified period which was enough to interfere immunogenicity.

Subjects who do not comply with the protocol will not be replaced. Subjects who stop study vaccine for any other reason (i.e., AE) will not be replaced.

7.4.4.2 Removal of Subjects from the Study

Subjects may withdraw from the study for any of the following reasons:

- Subjects would not receive any booster dose of UB-612 vaccine after enrolment.
- Lost to follow-up
- Consent withdrawal
- Death
- Any pathological event, clinical adverse event, or any change in the subject's status giving indication to the doctor that further participation in the study may not be the best interests of the subject, according to investigator's discretion.

Subjects who withdraw consent will not be replaced.

Subjects are free to withdraw from the study at any time without providing reason(s) for withdrawal and without prejudice to further research treatment. The reason(s) for withdrawal will be documented in the case report form (CRF).

Subjects withdrawing from the study, except subject death, will be encouraged to complete the same final evaluations (as Visit 3 procedure) within 7 days after withdrawal, as subjects completing the study according to this protocol, particularly safety evaluations. The aim is to record data in the same way as for subjects who completed the study.

Reasonable efforts will be made to contact subjects who are lost to follow-up. These efforts must be documented in the subject's file.

7.4.4.3 Study Termination

The sponsor has the right to terminate the study at any time in case of SAEs or if special circumstances concerning the study agent or the company itself occur, making further research treatment of subjects impossible. In this event, the investigator(s) will be informed of the reason for study termination.

7.4.4.4 Reporting and Follow-up of Pregnancies

A positive urine pregnancy test should be confirmed by a serum pregnancy test. A negative serum pregnancy result is required before the subject may receive the study treatment. Subjects who become pregnant while on study must immediately discontinue study treatment. The pregnancy must be reported and recorded on the sponsor's pregnancy form within 24 hours of the investigator's or study site staff's acknowledgement of the pregnancy. Pregnancies for female subjects, or for the female partners of male subjects occurring during study period after booster vaccination, must be reported to the sponsor. Pregnancies should be handled and reported as AEs.

The investigator should inform the subjects of the risks of continuing with the pregnancy and the possible side effects to the fetus. The outcome of all pregnancies (spontaneous miscarriage, elective termination, normal birth, or congenital abnormality) must be followed up and documented even if the subject is discontinued from the study.

All reports of congenital abnormalities/birth defects and spontaneous miscarriages should be handled and reported as SAEs, to be reported within 24 hours of site awareness (regardless of interval since study treatment). Elective abortions should be handled and reported as AEs.

7.4.4.5 SARS-CoV-2 infection

SARS-CoV-2 infection should be confirmed during the whole study period. For subject who received vaccination per protocol, if he/she is infected by SARS-CoV-2, immunogenicity data from this subject will not be included in analysis. Subject with suspected SARS-CoV-2 infection will be recorded as AE, while infected with SARS-Co-V-2, which fulfilling any one or more of these criteria in the definition in section 9.5.5.2, will be documented and reported following the same procedure of SAE reporting, especial attention should be paid in case of the occurrence of antibody-dependent enhancement (ADE), or vaccine-associated enhanced respiratory disease (VAERD). Subjects encountered SARS-CoV-2 infection will not withdraw from the study, unless other withdrawal reason judged to be necessary by investigators.

7.5 Investigational Products

7.5.1 Identity of Investigational Products

There will be one formulation for UB-612 vaccines. Each vial of 6.5 mL of UB-612 vaccine will be supplied in 10 mL glass vial.

Name	UB-612
Characteristics & Physical State	White to off-white **suspension without foreign objects**
Formulated & Supplied by	United Biomedical, Inc.
Storage Conditions	Cooled (2°C -8°C) following receipt at site until the time of use
Shipments	Cooled (2°C -8°C)
Package	A disposable multi-dose vial containing 200 µg/mL UB-612 protein/peptide as the following:<br>- 200 µg/mL: 176 µg S1-RBD-sFc protein and 24 µg of six peptides/per 1 mL included
Batch No.	303981

The vaccine lots used in this study have been tested and released by the quality control department of the UBI Pharma Inc. Non-clinical studies of UB-612 formulated with Adju-Phos® and CpG 1 are detailed in the Investigator's Brochure (IB).

7.5.2 Investigational Products Administered

7.5.2.1 Injection Volume(s)

	Booster vaccination
A group	UB-612 vaccine 100 µg, 0.5mL
B group	UB-612 vaccine 100 µg, 0.5mL
C group	UB-612 vaccine 100 µg, 0.5mL

7.5.2.2 Injection Route and Rate

For this trial, one booster dose of UB-612 vaccine must be injected by an intramuscular (IM) route. Injections on Day 1 will be given into deltoid muscles preferably of the nondominant arm.

7.5.2.3 Emergency Event Management

All subjects in each group will be monitored at least 30 minutes after vaccination (for the change of vital sign or adverse event). Subjects will be encouraged to quickly report any symptoms at the time during this period. The necessary rescue material, equipment, and appropriate medications will be available in the clinic to allow rapid intervention in case of anaphylaxis or other emergency.

7.5.3 Packaging and Labeling

The study packaging will be performed by UBI Pharma Inc.

All packaging and labeling operations will be performed according to Good Manufacturing Practice for Medicinal Products and the relevant regulatory requirements. The labels for the outer box/vial box/vial of the study vaccine contain the following information: the name/address/telephone number of UBI Pharma Inc. , Product name, Study code, Indication, Package size, Dosage unit, Manufacture company, Batch No., Manufacture date, Expiration date, Storage conditions, Active ingredient concentration (only for UB-612 protein/peptide), Injection method, Study site, Visit date___, Visit No.___, Subject No.___, Study PI and words of caution stating the product is for investigational and clinical trial use only.

7.5.4 Prior and Concomitant Therapy

There is no specific known evidence of contraindications between the ingredients of UB-612 vaccine and other prior and concomitant therapy. Concomitant medications and therapies will be recorded beginning 1 months prior to the booster vaccination, as well as during study period.

7.5.4.1 Prohibited Medication/Therapy

The following medications or treatments which may affect the immunogenicity and clinical efficacy assessments will be prohibited during study period:

- Investigational product (including drug, vaccine)
- Immunoglobulins and/or any blood products
- Immunosuppressant or corticosteroids, cytotoxic treatment
- COVID-19 vaccine other than study vaccine
- Other registered vaccine could be administered after Day 29 (subunit vaccine or inactivated vaccine could be after Day 15), and should be apart from Day 85 at least one month.

8 TIMING OF STUDY PROCEDURES

8.1 Visit 1 (Day 1) – Screening and booster vaccination

The following assessment(s) must be collected/performed at Day 1.

(1) Record date of informed consent will be signed. The following should be documented in the subject's medical chart: that they are participating in this study that informed consent has been obtained and that a copy of the consent has been given to the subject.
(2) Assign screen number
(3) Eligibility: Assess against the inclusion and exclusion criteria
(4) Demographics
(5) Medical history and concurrent diseases.
(6) Conduct physical exam including the measurement of weight and height
(7) Measure vital signs
(8) Perform a urine pregnancy test
(9) Collect blood sample for following local laboratory tests before vaccination
   - ➢ Blood routine test
   - ➢ Biochemistry tests

(10) Collect blood sample for immunogenicity tests (central laboratory) before vaccination
   - ➢ Humoral immune response
   - ➢ Cellular immune response

(11) Collect blood sample for SARS-CoV-2 test before vaccination
   - ➢ Serum antibodies (IgG) against SARS-CoV-2.

(12) Perform booster vaccination. All subjects in each group and at least 30 minutes after vaccination at site (for the change of vital sign or adverse event).
(13) Instruct the subject to monitor body temperature and complete self-evaluation e-diary correctly.
Subject will be instructed to record any solicited adverse events occurring during a 7-day post-vaccination period on the e-diary. If the subject perceives any signs or symptoms are progressing or serious, contact the investigator or their delegates immediately. Additional return visits can be scheduled by the investigators when necessary.
(14) Conduct COVID-19 surveillance
(15) Review concomitant medications.

8.2 Visit 2 (Day 15 ± 3 day)

The following assessment(s) must be collected/performed:

(1) Conduct physical examination.
(2) Measure vital signs.
(3) Collect blood sample for following local laboratory tests:
   - ➢ Blood routine test
   - ➢ Biochemistry tests

(4) Collect blood sample for immunogenicity tests (central laboratory)
   - ➢ Humoral immune response
   - ➢ Cellular immune response

(5) Review concomitant medications.
(6) Conduct COVID-19 surveillance
(7) Record and report adverse or serious adverse event if any has occurred since the previous visit.

8.3 Visit 3 (Day 85 ± 5 days) –Month 3 Follow-up /End of Study

The following assessment(s) must be collected/performed:

(1) Conduct physical examination.
(2) Measure vital signs.
(3) Perform a urine pregnancy test
(4) Collect blood sample for immunogenicity tests (central laboratory):
   - ➢ Humoral immune response
   - ➢ Cellular immune response

(5) Collect blood sample for SARS-CoV-2 test
   - ➢ Serum antibodies (IgG) against SARS-CoV-2.

(6) Review concomitant medications.
(7) Conduct COVID-19 surveillance
(8) Record and report adverse or serious adverse event if any has occurred since the previous visit.

8.4 Early Termination (ET)

For subjects who discontinue this study earlier, a final follow-up may be arranged not late than 7 days and all study procedures listed for Visit 3 should be completed.

8.5 Unscheduled Visit

Subjects who suffered from severe solicited AE or unsolicited AE at any moment, which are unexpected no matter the severity or event, should return to site ASAP for further survey and treatment.

8.6 Duration of Treatment

The duration of each visit is expected to last between 4 to 5 hours during 1 day, barring any unexpected adverse reactions.

It takes about 3 months for each subject to participate in the study, from recruiting to the last visit. Some subjects may withdraw from the study during the course of the study.

9 IMMUNOGENICITY AND SAFETY VARIABLES

The planned schedule of assessments is in Section 7.2.

9.1 Informed Consent Form

The investigator or designee must explain to each subject the nature of the study, its purpose, the procedures involved, the expected duration, the potential risks and benefits involved and any potential adverse events. Each subject will be informed that participation in the study is voluntary and that they can be withdrawn from participation at any time.

All subjects must provide a signed and dated informed consent at Visit 1. An informed consent form must be approved by the Institutional Review Board (IRB), Ethics Committee (EC), and/or the applicable health authorities.

9.2 Demographics / Other Baseline Characteristics

The demographic and baseline characteristic data for subjects will be collected at Visit 1. The demographics include date of birth, age, sex and ethnicity. Any pre-vaccination medical events will be recorded as medical history. Relevant history/conditions include all those present prior to the administration of study vaccine that are listed below:

- Relevant medical history,
- All current medical conditions,
- Allergy history,

Whenever possible, diagnoses but not symptoms should be recorded.

9.3 Eligibility

Eligibility should be checked by the investigator at Visit 1 before vaccination.

9.4 Administration

Take a vial of investigational vaccine, use a disposable needle and syringe to extract vaccine and intramuscularly inject it into the middle of the lateral deltoid muscle of the subject's upper arm.

Vaccine administration will be recorded, including quantity (volume and dose). The containers from which the vaccine was administered to the subjects will be retained for dose confirmation.

9.5 Safety and Immunogenicity Measurements Assessed

9.5.1 Safety Variables

9.5.1.1 Physical Examination

Complete physical examinations should be conducted by investigator/site staff at all visits. A complete physical exam will include the examination of general appearance, HEENT (head, ears, eyes, nose, throat), neck (including thyroid), lymph nodes, skin, cardiovascular, pulmonary, abdomen, neurological system and musculoskeletal/joints.

Body weight and height will be collected at Visit 1. Body weight will be measured in indoor clothing, but without shoes and blanket to the nearest 0.1 kilogram (kg). Body height will be measured in centimeters (cm).

It must be recorded when any abnormality has been found out.

9.5.1.2 Vital Sign

Systolic/diastolic blood pressure, pulse, respiratory rate, and ear temperature will be collected at all visits.

For booster vaccination visit, vital sign should be measured prior to and after vaccine administration. All subjects in each group will be monitored at least 30 minutes after vaccination, and vital signs should be recorded in CRF.

9.5.2 Clinical Laboratory Evaluation

The clinical laboratory analyses will be performed at local laboratories. Reference ranges will be supplied by the local laboratories and used by the investigator to assess the laboratory data for clinical significance and pathological changes.

Methods and timing for assessing, recording and analyzing each laboratory variable should follow local guidelines. The following laboratory safety tests will be performed:

Blood Routine

CBC, including Hb, Hct, RBC count, WBC count, WBC differential and platelet count.

Biochemistry

ALT, AST, total bilirubin, creatinine, and HbA1c

9.5.3 Pregnancy Test

Screening for urine pregnancy, using pregnancy strip, will be performed on Day 1 before vaccination and on Day 85 for WoCBP only. It is not required for postmenopausal or surgically sterilized women. A positive urine pregnancy test should be confirmed by a serum test. Pregnancy

tests may be performed more frequently per request of Institutional Review Board (IRB)/Independent Ethics Committee (IEC) or if required by local regulations.

9.5.4 Self-Evaluation/Reporting (Solicited Symptoms)

9.5.4.1 Solicited Symptoms

Information of solicited symptoms/AEs and body temperature will be collected by the subjects in the provided e-diary cards during a 7-day follow-up period after the vaccination (i.e. day of vaccination and 6 subsequent days), and reported by the investigator team. The subject should complete the assessments in the e-diary every evening.

A solicited local and general symptoms or AEs are the one whose nature or intensity is consistent with the expected AEs described and listed below.

Table 9-1 Grading for solicited adverse events at the injection site

	**Mild (Grade 1)**	**Moderate (Grade 2)**	**Severe (Grade 3)**	**Potentially Life Threatening (Grade 4)**
Pain	Do not affect physical activity	Affect physical activity	Affect daily life	Emergency room (ER) visit or hospitalization
Induration*, swelling (optional)**#	Diameter 2.5~＜5 cm and does not affect or slightly affect daily life	Diameter 5~＜10 cm or interferes with daily life	Diameter ≥ 10 cm or prevents daily activity	Necrosis
Rash*, Redness (optional)** #	Diameter 2.5~＜5 cm	Diameter 5~＜10 cm	Diameter ≥ 10 cm	Necrosis or exfoliative dermatitis
Allergic Reaction	Itching at vaccine site, no rash	Localized urticaria	Generalized urticaria; angioedema	Anaphylaxis
Cellulitis	NA	Non-injectable treatment is required (e.g., oral antibacterial, antifungal, antiviral therapy)	Intravenous treatment is required (e.g., intravenous antibacterial, antifungal, antiviral therapy)	Sepsis, or tissue necrosis, etc.

Note: *: in addition to directly measuring the diameter for grading and evaluation, the progress of the measurement results should also be recorded.
** the maximum measuring diameter or area should be used.
# the evaluation and grading of induration and swelling, rash and redness should be based on the functional level and the actual measurement results, and the indicators with higher classification should be selected.

Table 9-2 Grading for solicited systemic adverse events

	**Mild (Grade 1)**	**Moderate (Grade 2)**	**Severe (Grade 3)**	**Potentially Life Threatening (Grade 4)**
Diarrhea	2 – 3 loose stools /24 hours	4 – 5 stools /24 hours	6 or more watery stools/24 hours or requires outpatient IV hydration	ER visit or hospitalization for hypotensive shock
Fatigue	Does not affect daily activities	Affects normal daily activities	Seriously affects daily activities and cannot work	Emergency Room visit or hospitalization
Nausea/Vomiting	No interference with activity or 1 – 2 episodes/24 hours	Some interference with activity with activity or > 2 episodes/24 hours	Prevents daily activity, requires outpatient IV hydration	Life-threatening (e.g. hypotension shock)
Anorexia	Loss of appetite, but no reduction in food intake	Loss of appetite, reduced food intake	Loss of appetite and no food intake	Need for intervention (e.g. gastric tube feeding, parenteral nutrition)
Sore throat	Transient, without treatment, without affecting daily activities	Sore throat, slightly affecting daily activities	Severe sore throat that seriously affects daily activities and requires medication	
Headache	Does not affect daily activities	Transient, slightly affects daily activities and may require treatment or intervention	Seriously affects daily activities and requires treatment or intervention	ER visit or hospitalization
Cough	Transient, without treatment	Persistent cough, effective treatment	Paroxysmal cough, uncontrollable treatment	Emergency or hospitalization
Arthralgia	Mild pain without hindering function	Moderate pain; need analgesics and / or pain that impedes function but does not affect daily activities	Severe pain; need analgesics and / or pain affecting daily activities	Disability pain
Non-injection-site muscle pain	Does not affect daily activities	Slightly affect daily activities	Severe muscle pain that seriously affects daily activities	Emergency or hospitalization
Non-injection-site itching (no skin lesions)	Slightly itchy without affecting or slightly affecting daily life	Itching affects daily life	Itching makes it impossible to carry on daily life.	NA
Abnormal skin and mucosa	Erythema / itching / color change	Diffuse rash / macular papule / dryness / desquamation	Blister / exudation / desquamation / ulcer	Exfoliative dermatitis involving mucous membrane, or erythema multiforme, or suspected Stevens-

	**Mild (Grade 1)**	**Moderate (Grade 2)**	**Severe (Grade 3)**	**Potentially Life Threatening (Grade 4)**
				Johnsons syndrome
Acute allergic reaction *	Local urticaria (blister) without treatment	Local urticaria requiring treatment or mild angioedema without treatment	Extensive urticaria or angioedema requiring treatment or mild bronchospasm	Anaphylactic shock or life- threatening bronchospasm or throat edema
Syncope	Close to syncope without losing consciousness (pre-syncope)	Loss of consciousness without treatment	Loss of consciousness and needs treatment or hospitalization	NA
Acute bronchospasm	Transient; no treatment needed	Needs treatment; bronchodilator therapy returns to normal	Bronchodilator treatment cannot return to normal	Cyanosis; or intubation required
Dyspnea	Dyspnea during exercise	Dyspnea during normal activity	Dyspnea at rest	Dyspnea, requiring oxygen therapy, hospitalization or assisted breathing

* Refers to type I hypersensitivity.

The axillary temperature will be monitored every evening on day of vaccination and 6 subsequent days. If daily axillary temperature has been measured for more than one time, only the highest degree level on any day should be recorded in the e-diary card.

Table 9-3 Grading for axillary temperature

	**Mild (Grade 1)**	**Moderate (Grade 2)**	**Severe (Grade 3)**	**Potentially Life Threatening (Grade 4)**
Fever (°C)	37.3～＜38.0	38.0～＜38.5	38.5～＜39.5	≥39.5, last more than 3 days

9.5.4.2 Unsolicited Adverse Event

An unsolicited local and general AE or symptoms are the one whose nature or intensity is NOT consistent with the expected AEs described and listed above in this protocol. Information of unsolicited symptoms/AEs will be collected until 28-day follow-up period after each vaccination (i.e. day of vaccination and 27 subsequent days) and reported at each visit.

9.5.5 Adverse events (AEs)

In this study, all AEs/SAEs will be recorded after vaccination.

9.5.5.1 Definition of Adverse Events (AE)

An AE is any untoward medical occurrence in a patient or clinical investigation subject temporally associated with the use of a medicinal product, whether or not considered related to the medicinal product. The occurrence does not necessarily have to have a causal relationship with this treatment.

An AE can therefore be any unfavorable and unintended sign (including an abnormal laboratory finding), symptom, or disease (new or exacerbated) temporally associated with the use of a medicinal product, whether or not considered related to the medicinal product.

Examples of an AE include:

- Exacerbation of a chronic or intermittent pre-existing condition, including either an increase in frequency and/or intensity of the condition
- A new condition detected or diagnosed after informed consent, even though it may have been present prior to this
- Signs, symptoms, or the clinical sequelae of a suspected interaction
- Signs, symptoms, or the clinical sequelae of a suspected overdose of either study drug or a concurrent medication.
- Abnormal laboratory finding: Results that lie outside the normal ranges of laboratory parameters of biochemistry test or blood routine test are considered to be abnormal. Any of the following situations in one laboratory parameter of biochemistry test or blood routine test will be recorded as AEs during this trial. (1) change from “normal” at a former visit to “abnormal, clinical significance (CS)” at a latter visit (2) change from “abnormal, non-clinical significance (NCS)” at a former visit to “abnormal, clinical significance (CS)” at a latter visit.

Examples of an AE do NOT include:

- A medical or surgical procedure (*e.g.* endoscopy); the condition that leads to the procedure is an AE
- Situations where an untoward medical occurrence did not occur (social and/or convenience admission to hospital)
- Anticipated day-to-day fluctuations of pre-existing disease(s) or condition(s) present or detected before informed consent was obtained, that do not worsen.

A priori, immunogenicity endpoints as specified in the protocol will not be considered as AEs except if, because of the course or severity or any other features of such events, the investigator, according to his/her best medical judgment, considers these events as exceptional in this medical condition.

9.5.5.2 Definition of Serious Adverse Events (SAE)

A SAE is any untoward medical occurrence that at any dose:

- Results in death or,
- Is life-threatening or,

  Note: The term "life-threatening" in the definition of "serious" refers to an event in which the subject was at risk of death at the time of the event; it does not refer to an event, which hypothetically might have caused death if it were more severe.

- Requires inpatient hospitalization or prolongation of existing hospitalization or,

  Note: In general, hospitalization signifies that the subject has been detained (usually involving at least an overnight stay) at the hospital or emergency ward for observation and/or treatment that would not have been appropriate in the physician's office or out-subject setting. Complications that occur during hospitalization are AEs. If a complication prolongs hospitalization or fulfils any other serious criteria, the event is serious. When in doubt as to

whether hospitalization occurred or was necessary, the AE should be considered serious. Hospitalization for elective treatment of a pre-existing condition that did not worsen from baseline is not considered an AE.

- Results in persistent or significant disability/incapacity or,

  Note: The term disability means a substantial disruption of a person's ability to conduct normal life functions. This definition is not intended to include experiences of relatively minor medical significance such as uncomplicated headache, nausea, vomiting, diarrhoea, influenza, and accidental trauma (e.g. sprained ankle), which may interfere or prevent everyday life functions but do not constitute a substantial disruption.

- Is a congenital anomaly/birth defect or,
- Is a medically important event

  Note: Medical and scientific judgment should be exercised in deciding whether expedited reporting is appropriate in other situations, such as important medical events that may not be immediately life threatening or result in death or hospitalization but may jeopardize the subject or may require intervention to prevent one of the other outcomes listed in the definition above. These should also be considered serious. Examples of such events are invasive or malignant cancers, intensive treatment in an emergency room or at home for allergic bronchospasm, blood dyscrasias or convulsions that do not result in hospitalisation, or development of drug dependency or drug abuse.

An AE fulfilling any one or more of these criteria should be reported as a SAE, irrespective of the dose of drug given, and even if it is the result of an interaction or drug abuse.

A distinction should be drawn between serious and severe AEs. The term 'severe' is often used to describe the intensity (severity) of a specific event (as in mild, moderate, or severe myocardial infarction); the event itself, however, may be of relatively minor medical significance (such as severe headache). This is not the same as 'serious,' which is based on subject's event outcome or action criteria usually associated with events that pose a threat to a subject's life or functioning. The seriousness (not severity) serves as a guide for defining regulatory reporting obligations.

9.5.5.3 Definition of Unexpected Adverse Reaction Definition

An unexpected adverse reaction is any untoward and unintended response that is related to the administration of the study agent, at any dose that is not consistent with the applicable product information (e.g., current version of the Investigator's Brochure for an unauthorized investigational medicinal product or summary of product characteristics for an authorized product).

9.5.5.4 Definition of Medically Attended Adverse Event

A Medically Attended Adverse Event (MAAE) is an AE with a medically-attended visit that is not a routine visit for physical examination or vaccination, such as visits for hospitalization, an emergency room visit, or an otherwise unscheduled visit to or from medical personnel (medical doctor) for any reason.

9.5.5.5 Definition of Adverse Events Following Immunization (AEFI) [12]

An adverse event following immunization (AEFI) is defined as any untoward medical occurrence which follows immunization, and which does not necessarily have a causal relationship with the usage of the vaccine. The adverse event may be any unfavorable or unintended sign, abnormal laboratory finding, symptom or disease. In this study, adverse events, including solicited adverse events, will be categorized as AEFIs.

9.5.5.6 Definition of Adverse Events of Special Interest (AESI) [12]

Adverse event of special interest (AESI) is further defined in Council for International Organizations of Medical Sciences (CIOMS) VII as:

An adverse event of special interest (serious or non-serious) is one of scientific and medical concern specific to the sponsor’s product or specific target disease, for which ongoing monitoring and rapid communication by the investigator to the sponsor could be appropriate. Such an event might require further investigation in order to characterize and understand it. Depending on the nature of the event, rapid communication by the trial sponsor to other parties (e.g., regulators) might also be warranted. AESI will be collected during the study period.

The AESI relevant to vaccination in general applicable to COVID-19 vaccine listing below:

Table 9-4 The AESI relevant to vaccination in general applicable to COVID-19

Body System	AESI Type
Neurologic	Generalized convulsion, Guillain-Barré syndrome (GBS), acute disseminated encephalomyelitis (ADEM)
Hematologic	Thrombocytopenia
Immunologic	Anaphylaxis, vasculitides, enhanced disease following immunization
Respiratory	Acute respiratory distress syndrome (ARDS), pneumonitis
Other	Serious local/systemic AEFI, acute cardiac injury, arrhythmia, septic shock-like syndrome, acute kidney injury

9.5.5.7 Assessment of Severity

All AEs, except AEs in in e-diary card, will be assessed according to the US NCI Common Terminology Criteria for Adverse Events (CTCAE) 5.0 (published on November 27, 2017) associated with the AE term. The following standard with 5 grades is to be used to measure the severity of adverse events in this study.

Table 9-5 Intensity scales of AE

Grades of AE	Description
Grade 1	Mild; asymptomatic or mild symptoms; clinical or diagnostic observations only; intervention not indicated.
Grade 2	Moderate; minimal, local or noninvasive intervention indicated; limiting age-appropriate instrumental ADL*
Grade 3	Severe or medically significant but not immediately life-threatening; hospitalization or prolongation of hospitalization indicated; disabling, limiting self care ADL**
Grade 4	Life-threatening consequences; urgent intervention indicated.
Grade 5	Death related to AE.

* Instrumental activities of diary living (ADL) refer to preparing meals, shopping for groceries or clothes, using the telephone, managing money, etc.

** Self-care ADL refer to bathing, dressing and undressing, feeding self, using the toilet, taking medication, and not bedridden.

9.5.5.8 The Relationship to Study Vaccine

The investigator will make an assessment of the relationship between investigational vaccine and the occurrence of each AE/SAE, except solicited reactions to vaccination. The reasonable possibility will be determined based on the investigator's clinical judgment. The causality should be considered as one of the categories described below.

Table 9-6 The relationship between AE and study vaccine:

Causality term	Assessment criteria
Certain	● Event or laboratory test abnormality, with plausible time relationship to drug intake<br>● Cannot be explained by disease or other drugs<br>● Response to withdrawal plausible (pharmacologically, pathologically)<br>● Event definitive pharmacologically or phenomenologically (i.e. an objective and specific medical disorder or a recognized pharmacological phenomenon)<br>● Rechallenge satisfactory, if necessary
Probable / Likely	● Event or laboratory test abnormality, with reasonable time relationship to drug intake<br>● Unlikely to be attributed to disease or other drugs<br>● Response to withdrawal clinically reasonable<br>● Rechallenge not required
Possible	● Event or laboratory test abnormality, with reasonable time relationship to drug intake<br>● Could also be explained by disease or other drugs<br>● Information on drug withdrawal may be lacking or unclear
Unlikely	● Event or laboratory test abnormality, with a time to drug intake that makes a relationship improbable (but not impossible)<br>● Disease or other drugs provide plausible explanations
Unrelated	● Occurred before dosing<br>● Due wholly to factors other than study treatment

Each event should be followed until resolution or the event is considered stable. Both regular return and telephone contact will be acceptable.

9.5.5.9 Adverse Events Reporting

Documentation and Reporting of Adverse Events

All investigators should follow up subjects with AEs until the event is resolved or until, in the opinion of the investigator, the event is stabilized or determined to be chronic. Details of AE resolution must be documented in the CRF.

All AEs should be reported and documented in accordance with the procedures outlined in this section. All AEs occurring during the study must be documented on the relevant CRF pages.

Reporting of Serious Adverse Events

Any SAE must be reported by the investigator if it occurs during the clinical study or within 4-7 days of receiving the study agent, whether or not the SAE is considered to be related to the investigational product. An SAE report consists of the SAE form, the AE form, and the concomitant medication form. A copy of these forms must be emailed **within 24 hours** to Contract Research Organization (CRO), StatPlus Inc., and StatPlus will inform United Biomedical, Inc., Asia on the same day.

The investigator should not wait to receive additional information to document fully the event before notification of a SAE, though additional information may be requested. Where applicable, information from relevant laboratory results, hospital case records, and autopsy reports should be obtained.

Instances of death, congenital abnormality, or an event that is of such clinical concern as to influence the overall assessment of safety, if brought to the attention of the investigator at any time after cessation of study agent administration and linked by the investigator to this study, should be reported to the study monitor.

The sponsor and/or the appointed representative(s) will promptly notify all relevant investigators and the regulatory authorities of findings that could adversely affect the safety of subjects, impact on the conduct of the study, or alter the IEC/ IRB approval/favorable opinion of the study. In addition, the sponsor and/or the appointed representative(s), will expedite the reporting to all concerned investigators, to the IEC(s)/IRB(s), where required, and to the regulatory authorities of all adverse reactions that are both serious and unexpected.

Details of the procedures to be followed if a pregnancy occurs are also provided in Section 7.4.4.

Documentation and Reporting of SUSARs

All suspected unexpected serious adverse reactions (SUSARs) will be the subject of expedited reporting. The sponsor and/or the appointed representative(s) shall ensure that all relevant information about a SUSAR that is fatal or life-threatening is reported to the relevant competent authorities and IEC/IRB within 7 days after knowledge by the sponsor of such a case and that relevant follow-up information is communicated within an additional 8 days. All other SUSARs will be reported to the relevant competent authorities and IEC/IRB within 15 days after knowledge by the sponsor of such a case. All investigators should follow up SUSARs until the event is

resolved or until, in the opinion of the investigator, the event is stabilized or determined to be chronic. Poststudy SUSARs that occur after the subject has completed the clinical study must be reported by the investigator to the sponsor.

9.5.5.10 Human leukocyte antigen (HLA)-genotyping

In order to identify the MHC alleles in subjects with **any allergic reactions with severity of grade 3 or above** and/or SAEs related to vaccine, an additional 3 mL of blood sample will be collected for HLA genotyping.

9.5.6 Immunogenicity Assessments

9.5.6.1 Detection of ELISA Antibodies against S1-RBD of SARS-CoV-2

Anti-S1-RBD antibody titers will be measured by ready-to-use ELISA kit, which is manufactured by UBIA. A dilution microplate is used for preparing serum dilutions and then transferred to reactive microplate for measurement of absorbance at 450 nm. The Anti-S1-RBD antibody titer is determined by SoftMax Titer Calculation Program (Molecular Devices Co.). The calculated titers need to be transformed into linear titers for analysis, if needed. The minimum dilution factor in this assay is 20-fold.

9.5.6.2 Detection of Antibody Titers Which Inhibit S1-RBD:ACE2 binding

Antibody titers to inhibit S1-RBD:ACE2 binding will be measured by SARS-CoV-2 qNeu Ab ELISA kit, which is manufactured by UBIA. A dilution microplate is used for preparing serum dilutions and then transferred to reactive microplate for measurement of absorbance at 450/650 nm. Antibody levels which inhibit S1-RBD:ACE2 binding are expressed in μg/mL for each test specimen, using SoftMax Titer Calculation Program (Molecular Devices Co.). Specimens that do not have ability to inhibit the binding of S1-RBD:ACE2 are considered with < 1.6 μg/mL.

9.5.6.3 Neutralizing Antibody Titers against SARS-CoV-2

Neutralizing antibody titers will be measured by CPE-based live virus neutralization assay using Vero-E6 cells challenged with SARS-CoV-2 (SARS-CoV-2-TCDC#4). The study will be conducted in P3 lab at Academia Sinica, Taipei. Determination of SARS-CoV-2 virus specific neutralization titer is to measure the neutralizing antibody titer against SARS-CoV-2 virus based on the principle of NT_{50} titer (50% reduction of virus-induced cytopathic effects). Virus neutralization titer of a serum is defined as the reciprocal of serum dilution at which 50% reduction in cytopathic effects, which are calculated by Reed and Muench method.

9.5.6.4 Neutralizing Antibody Titers against SARS-CoV-2 variants using pseudovirus neutralizing antibody assay

Neutralizing antibody titers will be measured by pseudovirus neutralization assay using HEK-293T-ACE2 cells. To produce SARS-CoV-2 pseudoviruses, a plasmid expressing full-length wild-type Wuhan-Hu-1 strain SARS-CoV-2 spike protein was constructed. Pseudoviruses were produced by co-transfection of plasmids encoding a luciferase reporter, lentivirus backbone, and S genes into HEK-293T cells. Site-directed mutagenesis was used to generate variants by changing

nucleotide from Wuhan-Hu-1 reference strain. In brief, heat-inactivated serum will be mixed with pseudoviruses, incubated, and then added to HEK-293T-ACE2 cells. After post-infection, cells will be lysed and relative luciferase units (RLU) measured. The percentage of inhibition is calculated as the ratio of RLU reduction in the presence of diluted serum to the RLU value of only virus control. The pNT_{50} values are calculated by Reed and Muench method.

9.5.6.5 Detection of T Cell Response

Human peripheral blood mononuclear cells will be used in the detection of T cell response. Measurement of antigen-specific interferon-gamma (IFN-γ) and IL-4 production to assess cellular (T cell) immune response will be evaluated by ELISpot method. Intracellular cytokine staining of IFN-γ, IL-2 and IL-4 via flow cytometry will be used to evaluate $CD4^+$ T cell responses. Intracellular cytokine staining of IFN-γ, IL-2, CD107a and Granzyme B via flow cytometry will be used to evaluate $CD8^+$ T cell responses.

9.5.7 SARS-CoV-2 test

Specific IgG antibodies against SARS-CoV-2 in the serum of the subjects will be tested by UBI SARS-CoV-2 ELISA kit. Those who have been infected by SARS-CoV-2 will show positive for the antibody test.

9.5.8 Overall Study Stopping Rules

Overall study stopping rules is listed below.

- One or more ≥ grade 4 adverse reaction or serious adverse event possibly associated with vaccination

- Occurrence of grade 3 adverse events associated with vaccination in 3 or more of subjects in a treatment group participants or more (including injection-site reaction, systemic reaction, and change of the safety laboratory measures)

- Required by sponsor

- Required by regulatory authority

- Required by institutional review board (IRB).

- The sponsor may also end the study for administrative reasons.

Should the study be terminated prematurely, the sponsor will provide written notification to all investigators and regulatory authorities specifying the reason(s) for early termination. The investigator must inform the institutional review board (IRB)/independent ethics committees (IEC) promptly and provide the reason(s) for the termination. Previously dosed subjects will be assessed through all planned study safety visits.

9.5.9 Appropriateness of Measurements

The immunogenicity and safety assessments planned for this study are generally recognized as reliable, accurate, and relevant to the diagnostic modality and underlying disease/condition.

9.6 Surveillance and Laboratory Diagnosis of SARS-CoV-2 Infection during Clinical Trial

During the observation period of the clinical trial, the participants with fever, cough and other respiratory symptoms should immediately go to the designated hospital. The doctor or investigator will collect the nasopharyngeal/throat swabs and to perform CT and other imaging examinations to analyse whether it is caused by SARS- CoV-2 infection. In the event of SARS-CoV-2's infection during the clinical trial, it is necessary to conduct a case investigation, and the critically ill or dead cases need to continue to conduct a special investigation, mainly to analyze whether there is an ADE or VAERD phenomenon.

In addition to SARS-CoV-2 nucleic acid detection, multiple pathogens will be detected for differential diagnosis with swabs.

10 STATISTICAL METHODS

The statistical planning and analysis of the trial will be performed by the designated contract research organization.

10.1 Statistical and Analytical Plans

A statistical analysis plan will be prepared and finalized prior to database lock of the study. The statistical analysis plan will include full details of all planned statistical analyses. In order to inform any potential modification of current or planned trials of UB-612, an interim analysis will be performed to present Day 1 thru Day 15 safety and immunogenicity data.

10.2 Datasets or Populations Analysed

Safety Set

The Safety Set (SS) will consist of all subjects receiving at least one injection of the UB-612 vaccine. The Safety Set is for safety evaluation in analysis.

Full Analysis Set

The Full Analysis Set (FAS) will consist of subjects who completed two vaccinations in Phase I study enrolled into this extension study, received one booster dose of UB-612 vaccines with the same dose as Phase II study, and have at least one immunogenicity assessment on Day 15 or 85 after vaccination. Subjects receive prohibited medication/treatment/vaccine during pre-specified period has impact on immunogenicity may exclude from the FAS set. The FAS set is the primary analysis set for immunogenicity evaluation.

Per Protocol Set

The Per Protocol Set (PPS) will be a subset of FAS. PPS includes subjects who completed two vaccinations in Phase I study enrolled into this extension study, received one booster dose of UB-612 vaccines with the same dose as Phase II study, and were compliant to the protocol. Subjects who had major protocol deviations as determined by the Study Team or who received prohibited medication/treatment/vaccine during the pre-specified period leading to an impact on immunogenicity will be excluded from the PPS set. The PPS is also analysed for immunogenicity evaluation.

10.3 Demographic and Other Baseline Characteristics

Demographic and baseline characteristic data will be summarized for each vaccine group and overall. Descriptive statistics (N, mean, standard deviation, median, minimum, and maximum) will be presented for continuous variables. The number and percentage of subjects in each category will be presented for categorical variables. No formal testing of demographic or baseline characteristics will be performed. **The prime-boost interval, which is the interval between the second dose of the primary vaccination series and the booster dose, will be calculated and presented.**

10.4 Safety Evaluation

All safety assessments, including AEs, PEs, VS and clinical laboratory evaluations, where indicated, will be presented using descriptive statistics for each vaccine group of UB-612. Data will be summarized for each vaccine group and overall.

Solicited Adverse Event

Solicited AEs recorded on e-diary will be summarized by the severity grading scales by vaccine groups. Numbers and percentages of subjects experiencing each adverse event will be presented for each symptom severity by study groups. Summary tables showing the occurrence of any local or systemic adverse event overall and at each time point will also be presented.

Adverse Event

This analysis applies to all adverse events occurring during the study, recorded in AE eCRF, with a start date on or after the date of vaccination. AEs occurring during the study will be coded using MedDRA version 23.0 which is consistent with the V-122 phase I main study. The adverse events will then be grouped by MedDRA preferred terms into frequency tables according to system organ class.

All reported adverse events, as well as adverse events judged by the investigator as at least possibly related to study vaccine, will be summarized according to system organ class and preferred term within system organ class. When an adverse event occurs more than once for a subject, the maximal severity and strongest relationship to the vaccine group will be counted.

Separate summaries will be produced for the following categories, such as serious adverse events and AEs related to UB-612 vaccine will be presented. Data listings of all adverse events will be provided by subject.

Physical Examination

All physical examination findings will be listed and summarized by time point and study vaccine group. Shift table will be also presented, if appropriate.

Vital Signs

All vital sign findings will be listed and summarized by time point and study vaccine group.

Laboratory Evaluations

Laboratory safety data will be analysed descriptively by time point and study vaccine group. Shift table for laboratory data will be shown by visit using categorization of laboratory according to local laboratory’s normal reference range.

10.4.1 Analysis of Primary Safety Endpoint

- Occurrence of adverse reactions within 7 days after vaccination

All adverse reactions within 7 days after vaccination will be summarized with frequencies and percentages by study vaccine group.

- The percentage of subjects with ≥ Grade 3 adverse events within 7 days after vaccination

  The subject who has ≥ Grade 3 solicited AE or ≥ Grade 3 unsolicited AE within 7 days after vaccination will be counted. The number and percentage of counted subjects will be demonstrated by study vaccine group.

10.4.2 Analysis of Secondary Safety Endpoint

- Occurrence of serious adverse events during the whole follow-up period (3 months)

  The number and percentage of SAEs during the whole follow-up period (3 months) will be presented in summary table by study vaccine groups.

- Occurrence of adverse events of special interests (AESIs), medically attend adverse events (MAAEs) and serious adverse events (SAEs) during the study period

  Adverse events of special interests (AESIs), medically attend adverse events (MAAEs) and serious adverse events (SAEs) during the study period will be summarized with number and percentage from the mapping result by MedDRA. Details will be defined in the statistical analysis plan.

- Changes of safety laboratory measures

  Changes of safety laboratory measures will be summarized with descriptive statistics by study vaccine group and time point. ANCOVA model with laboratory baseline values as covariate will analyse changes of safety laboratory measures for testing the difference among study vaccine groups. Pair-wise comparisons of least square means from the ANCOVA model will be presented. Intra-group difference in safety laboratory measures will also be analysed by paired t test.

10.5 Immunogenicity Evaluation

10.5.1 Analysis of Primary Immunogenicity Endpoints

- GMT of neutralizing antibody against SARS-CoV-2 **(Wuhan strain)** on Day 1, 15 and 85

  GMT of neutralizing antibody against SARS-CoV-2 **(Wuhan strain)** on Day 1, 15 and 85 will be summarized by descriptive statistics and the 95% confidence interval for study vaccine group. The difference on Day 1 among vaccine groups will be analyzed by ANOVA model under log-transform data. Additionally, the difference on Day 15 and 85 among vaccine groups will be analysed by ANCOVA model under log-transform data with baseline level as covariate, if appropriate. Pair-wise comparisons of least square means from the ANCOVA model will be presented. The reverse cumulative distribution plot will be provided to display the distribution of the GMT of neutralizing antibody against SARS-CoV-2 **(Wuhan strain)** by time points for each vaccine group.

- Geometric mean fold increase of neutralizing antibody against SARS-CoV-2 **(Wuhan strain)** on Day 15 and 85

  Geometric mean fold increase of neutralizing antibody against SARS-CoV-2 **(Wuhan strain)** on Day 15 and 85 will be summarized by descriptive statistics and the 95%

confidence interval for study vaccine group. The difference among vaccine groups will be analysed by ANOVA. Pair-wise comparisons of least square means from the ANOVA model will be presented. The reverse cumulative distribution plot will be provided to display the distribution of the fold increase of neutralizing antibody against SARS-CoV-2 **(Wuhan strain)** by time points for each vaccine group.

10.5.2 Analysis of Secondary Immunogenicity Endpoints

- Geometric mean titer (GMT) of antigen-specific antibody (Anti-S1-RBD) on Day 1, 15 and 85

  GMT of antigen-specific antibody of antigen-specific antibody (Anti-S1-RBD) on Day 1, 15 and 85 will be described by descriptive statistics and the 95% confidence interval for study vaccine group. The difference on Day 1 among vaccine groups will be analyzed by ANOVA model under log-transform data. Additionally, the difference on Day 15 and 85 among vaccine groups will be analyzed by ANCOVA model under log-transform data with baseline level as covariate, if appropriate. Pair-wise comparisons of least square means from the ANOVA or ANCOVA model will be presented. The reverse cumulative distribution plot will be provided to display the distribution of the titer of Anti-S1-RBD by time points for each vaccine group.

- Geometric mean fold increase of antigen-specific antibody (Anti-S1-RBD) on Day 15 and 85

  The geometric mean fold increase (GMFI) in each study vaccine group is summarized by descriptive statistics and the 95% confidence interval for study vaccine group. The difference among vaccine groups will be analysed by ANOVA. Pair-wise comparisons of least square means from the ANOVA model will be presented. The reverse cumulative distribution plot will be provided to display the distribution of the fold increase of Anti-S1-RBD by time points for each vaccine group.

- Distribution of titers

  For **anti-S1-RBD** ELISA, ACE2:RBD inhibition ELISA, and Neutralizing antibody titers, reverse cumulative distribution of titers **and fold increases** will be displayed at Day1, 15 and 85.

- Correlation between the immune response detected by ELISA and live virus neutralization test

  Pearson correlation coefficient will be used to measure the dependence of the immune response detected by anti-S1-RBD ELISA and live virus neutralization test, and the dependence of the immune response detected by qNeuAb ELISA (for research use only) and live virus neutralization test. Correlation coefficients will be presented by time point in each dose group, overall time points in each dose group and overall time points in overall UB-612 group. In addition, the consistency of the immune response detected by anti-S1-RBD ELISA and live virus neutralization test will be analysed by linear regression with the result of anti-S1-RBD ELISA as dependent variable and the value of live virus

neutralization test as independent variable. The scatter plot with the linear relationship and its 95% confidence interval will be drawn.

10.5.3 Analysis of Exploratory Immunogenicity Endpoints

- T cell responses to UB-612 measured by ELISpot and flow cytometric assays on Day 1,15 and 85

  T cell responses to UB-612 measured by ELISpot and flow cytometric assays on Day 1, 15 and 85 will be summarized by descriptive statistics.

- GMT of neutralizing antibody against SARS-CoV-2 variants on Day 1, 15 and 85 using pseudovirus neutralizing antibody assay

  GMT of neutralizing antibody against SARS-CoV-2 variants on Day 1, 15 and 85 using pseudovirus neutralizing antibody assay will be summarized by descriptive statistics and the 95% confidence interval for study vaccine group. The difference on Day 1 among vaccine groups will be analyzed by ANOVA model under log-transform data. Additionally, the difference among vaccine groups will be analysed by ANCOVA model under log-transform data with baseline level as covariate, if appropriate. Pair-wise comparisons of least square means from the ANCOVA model will be presented. The reverse cumulative distribution plot will be provided to display the distribution of the GMT of neutralizing antibody against SARS-CoV-2 by time points for each vaccine group.

  Neutralizing antibody GMT and GMFI will be determined against at least the following SARS-CoV-2 variants: Alpha (B.1.1.7), Beta (B.1.351), Gamma (P.1), and Delta (B.1.617.2). The fold decrease between titers of the original (Wuhan) strain and each variant will be determined, and compared using descriptive statistics.

- **The comparison between primary series and booster vaccination on the humoral immune response by comparing the Day 15 GMT and GMFI of neutralizing antibody (Wuhan strain) and antigen-specific antibody (Anti-S1-RBD) after booster dose to the GMT and GMFI 2 weeks after the second dose of the primary vaccination series (i.e. Day 42 of V-122 Phase 1).**

  An analysis will be performed on neutralizing antibody (Wuhan strain) and antigen-specific antibody (Anti-S1-RBD) comparing the Day 15 GMT and GMFI after booster dose to the GMT and GMFI 2 weeks after the second dose of the primary vaccination series (i.e. Day 42 of V-122 Phase 1). The comparison is tested via a paired-t test.

- **The comparison between primary series and booster vaccination on the humoral immune response by comparing the Day 85 GMT and GMFI of neutralizing antibody (Wuhan strain) and antigen-specific antibody (Anti-S1-RBD) after booster dose to the GMT and GMFI 3 months after the second dose of the primary vaccination series (i.e. Day 112 of V-122 Phase 1).**

  An analysis will be performed on neutralizing antibody (Wuhan strain) and antigen-specific antibody (Anti-S1-RBD) comparing the Day 85 GMT and GMFI after booster dose to the GMT and GMFI 3 months after the second dose of the primary

vaccination series (i.e. Day 112 of V-122 Phase 1). The comparison is tested via a paired-t test.

- **Figures (dot-plot) to show the individual responses and GMT at each time point of V-122 Phase 1 and V-123 Phase 1 extension studies in order to provide a comprehensive view of the kinetics of the primary and booster immune responses.**

  In order to study the kinetics of humoral immune responses of primary series and booster vaccination, for each vaccine group, figures (dot-plot) of neutralizing antibody (Wuhan strain) and antigen-specific antibody (Anti-S1-RBD) will be generated showing the individual responses and GMT at each available time point of V-122 Phase 1 and V-123 Phase 1 extension studies in order to provide a comprehensive view of the kinetics of the primary and booster immune responses. Separate figures will show the three primary vaccination dose groups with the booster response.

- **Figures (dot-plot) of individual prime-boost interval with immune responses to show 1) individual fold increase of Day 15 value of neutralizing antibody (Wuhan strain) compared with baseline of V-122 Phase 1 study, 2) individual fold increase of Day 15 value of neutralizing antibody (Wuhan strain) compared with baseline of this V-123 study, 3) individual fold increase of Day 15 value of antigen-specific antibody (Anti-S1-RBD) compared with baseline of V-122 Phase 1 study, 4) individual fold increase of Day 15 value of antigen-specific antibody (Anti-S1-RBD) compared with baseline of this V-123 study, 5) individual ELISpot IFN-gamma on Day 15.**

  In order to study the effect of prime-boost interval, for each vaccine group, figures (dot-plot) of individual prime-boost interval (X axis) with various immune responses (Y axis) described as follows will be generated to show 1) individual fold increase of Day 15 value of neutralizing antibody (Wuhan strain) compared with baseline of V-122 Phase 1 study (Y axis), 2) individual fold increase of Day 15 value of neutralizing antibody (Wuhan strain) compared with baseline of this V-123 study (Y axis), 3) individual fold increase of Day 15 value of antigen-specific antibody (Anti-S1-RBD) compared with baseline of V-122 Phase 1 study (Y axis), 4) individual fold increase of Day 15 value of antigen-specific antibody (Anti-S1-RBD) compared with baseline of this V-123 study (Y axis), 5) individual ELISpot IFN-gamma on Day 15.

10.6 Interim Analysis

The interim analysis, regarding to safety and immunogenicity of A, B and C groups, will be implemented after subjects with booster vaccination in A group, B group and C group have completed Visit 2 (Day 15), which is 14 days after booster vaccination. Interim analysis population will be the subjects in groups A, B and C who had completed booster vaccination. For the evaluation of safety and immunogenicity, the following endpoints will be included in the interim report.

- Occurrence of adverse reaction within 7 days after vaccination
- The percentage of subjects with ≥ Grade 3 adverse events within 7 days after vaccination

- Occurrence of adverse events of special interests (AESIs), medically attend adverse events (MAAEs) and serious adverse events (SAEs)
- GMT of antigen-specific antibody (Anti-S1-RBD) on Day 1 and 15
- Changes of safety laboratory measures
- Distribution of titers
- Geometric mean fold increase of antigen-specific antibody (Anti-S1-RBD) on Day 15
- GMT of neutralizing antibody against SARS-CoV-2 **(Wuhan strain)** on Day 1 and 15
- Geometric mean fold increase of neutralizing antibody against SARS-CoV-2 **(Wuhan strain)** on Day 15.
- Correlation between the immune response detected by ELISA and live virus neutralization test
- T cell responses to UB-612 measured by ELISpot and flow cytometric assays on Day 1 and 15
- GMT of neutralizing antibody against SARS-CoV-2 variants on Day 1 and 15 using pseudovirus neutralizing antibody assay
- **The comparison between primary series and booster vaccination on the humoral immune response by comparing the Day 15 GMT and GMFI of neutralizing antibody (Wuhan strain) and antigen-specific antibody (Anti-S1-RBD) after booster dose to the GMT and GMFI 2 weeks after the second dose of the primary vaccination series (i.e. Day 42 of V-122 Phase 1).**
- **Figures (dot-plot) to show the individual responses and GMT of neutralizing antibody (Wuhan strain) and antigen-specific antibody (Anti-S1-RBD) at each time point of V-122 Phase 1 and Day 1 and Day 15 of V-123 Phase 1 extension studies in order to provide a comprehensive view of the kinetics of the primary and booster immune responses.**
- **Figures (dot-plot) of individual prime-boost interval with immune responses to show 1) individual fold increase of Day 15 value of neutralizing antibody (Wuhan strain) compared with baseline of V-122 Phase 1 study, 2) individual fold increase of Day 15 value of neutralizing antibody (Wuhan strain) compared with baseline of this V-123 study, 3) individual fold increase of Day 15 value of antigen-specific antibody (Anti-S1-RBD) compared with baseline of V-122 Phase 1 study, 4) individual fold increase of Day 15 value of antigen-specific antibody (Anti-S1-RBD) compared with baseline of this V-123 study, 5) individual ELISpot IFN-gamma on Day 15.**

10.7 Handling of Missing Data

All available data will be displayed and utilized in data analysis. No imputation will be considered for the missing observations.

10.8 Determination of Sample Size

No formal statistical sample size and power computations are performed since the objectives of the study are to assess the safety and immunogenicity of the study vaccine. The sample size of each vaccine dose group is 20. In this study, twenty subjects will be involved in each vaccine dose group. A total of 60 subjects will be recruited.

10.9 Protocol Deviations

Protocol deviations will be categorized into important and non-important items, and definitions will be illustrated in the protocol deviation handling plan (PDHD). Events that beyond the PDHD will discuss with sponsor to determine the categorization.

11 QUALITY ASSURANCE AND QUALITY CONTROL

11.1 Audit and Inspection

Study centers and study documentation may be subject to Quality Assurance audit during the course of the study by the sponsor or its nominated representative. In addition, inspections may be conducted by regulatory authorities at their discretion.

11.2 Monitoring

Data for each subject will be recorded on an eCRF. Data collection must be completed for each subject who signs an ICF and is administered study agent.

In accordance with current good clinical practice (cGCP) and International Council for Harmonisation (ICH) guidelines, the study monitor will carry out source document verification at regular intervals to ensure that the data collected in the eCRF are accurate and reliable.

The investigator must permit the monitor, the IEC/IRB, the sponsor's internal auditors, and representatives from regulatory authorities direct access to all study-related documents and pertinent hospital or medical records for confirmation of data contained within the eCRFs.

11.3 Data Management and Coding

The sponsor and/or the appointed representative(s) will be responsible for activities associated with the data management of this study. This will include setting up a relevant database and data transfer mechanisms, along with appropriate validation of data and resolution of queries. Data generated within this clinical study will be handled according to the relevant standard operating procedures of the data management and biostatistics departments of the sponsor and/or the appointed representative(s).

Study centers will complete the eCRF. Data entered into the eCRF must be verifiable against source documents at the study center. Data to be recorded directly on the eCRF will be identified and the eCRF will be considered the source document. Any changes to the data entered into the data capture system will be compliant to FDA CFR 21 Part 11.

Medical coding will use Medical Dictionary for Regulatory Activities for AEs.

Missing or inconsistent data will be queried to the investigator for clarification. Subsequent modifications to the database will be documented.

12 RECORDS AND SUPPLIES

12.1 Drug Accountability

On receipt of the study agent (including rescue medication, if relevant), the investigator (or designee) will conduct an inventory of the supplies and verify that study agent supplies are received intact and in the correct amounts before completing a supplies receipt. The investigator will retain a copy of this receipt at the study center and return the original receipt to the study monitor. The monitor may check the study supplies at each study center at any time during the study.

It is the responsibility of the study monitor to ensure that the investigator (or designee) has correctly documented the amount of the study agent received, dispensed, and returned on the dispensing log that will be provided. A full drug accountability log will be maintained at the study center at all times. The study monitor will arrange collection of unused study agent returned by the subject. The study monitor will also perform an inventory of study agent at the close-out visit to the study center. All discrepancies must be accounted for and documented.

12.2 Financing and Insurance

Financing and insurance of this study will be outlined in a separate agreement between the contract research organization and the sponsor.

13 ETHICS

13.1 Independent Ethics Committee or Institutional Review Board

Before initiation of the study at each study center, the protocol, the ICF, other written material given to the subjects, and any other relevant study documentation will be submitted to the appropriate IEC/IRB. Written approval of the study and all relevant study information must be obtained before the study center can be initiated or the study agent is released to the investigator. Any necessary extensions or renewals of IEC/IRB approval must be obtained for changes to the study such as amendments to the protocol, the ICF or other study documentation. The written approval of the IEC/IRB together with the approved ICF must be filed in the study files.

The investigator will report promptly to the IEC/IRB any new information that may adversely affect the safety of the subjects or the conduct of the study. The investigator will submit written summaries of the study status to the IEC/IRB as required. On completion of the study, the IEC/IRB will be notified that the study has ended.

13.2 Regulatory Authorities

Relevant study documentation will be submitted to the regulatory authorities of the participating countries, according to local/national requirements, for review and approval before the beginning of the study. On completion of the study, the regulatory authorities will be notified that the study has ended.

13.3 Ethical Conduct of the Study

The investigator(s) and all parties involved in this study should conduct the study in adherence to the ethical principles based on the Declaration of Helsinki, cGCP, ICH guidelines, and the applicable national and local laws and regulatory requirements.

13.4 Informed Consent

The process of obtaining informed consent must be in accordance with applicable regulatory requirement(s) and must adhere to cGCP.

The investigator is responsible for ensuring that no subject undergoes any study related examination or activity before that subject has given written informed consent to participate in the study.

The investigator or designated personnel will inform the subject of the objectives, methods, anticipated benefits and potential risks and inconveniences of the study. The subject should be given every opportunity to ask for clarification of any points s/he does not understand and, if necessary, ask for more information. At the end of the interview, the subject will be given ample time to consider the study. Subjects will be required to sign and date the ICF. After signatures are obtained, the ICF will be kept and archived by the investigator in the investigator's study file. A signed and dated copy of the subject ICF will be provided to the subject or their authorized representative.

It should be emphasized that the subject may refuse to enter the study or to withdraw from the study at any time, without consequences for their further care or penalty or loss of benefits to which the subject is otherwise entitled. Subjects who refuse to give or who withdraw written informed consent should not be included or continue in the study.

If new information becomes available that may be relevant to the subject's willingness to continue participation in the study, a new ICF will be approved by the IEC(s)/IRB(s) (and regulatory authorities, if required). The study subjects will be informed about this new information and reconsent will be obtained.

13.5 Subject Confidentiality

Monitors, auditors, and other authorized agents of the sponsor and/or its designee, the IEC(s)/IRB(s) approving this research, and the United States (US) FDA, as well as that of any other applicable agency(ies), will be granted direct access to the study subjects' original medical records for verification of clinical study procedures and/or data, without violating the confidentiality of the subjects to the extent permitted by the law and regulations. In any presentations of the results of this study or in publications, the subjects' identity will remain confidential.

All personal data collected and processed for the purposes of this study should be managed by the investigator and his/her staff with adequate precautions to ensure confidentiality of those data, applicable to national and/or local laws and regulations on personal data protection.

14 REPORTING AND PUBLICATION, INCLUDING ARCHIVING

Essential documents are those documents that individually and collectively permit evaluation of the study and quality of the data produced. After completion of the study (end of study defined as the date of the last visit of the last subject), all documents and data relating to the study will be kept in an orderly manner by the investigator in a secure study file. This file will be available for inspection by the sponsor or its representatives. Essential documents should be retained for 2 years after the final marketing approval in an ICH region or for at least 2 years since the discontinuation of clinical development of the investigational product. It is the responsibility of the sponsor to inform the study center when these documents no longer need to be retained. The investigator must contact the sponsor before destroying any study related documentation. In addition, all subject medical records and other source documentation will be kept for the maximum time permitted by the hospital, institution, or medical practice.

The sponsor must review and approve any results of the study or abstracts for professional meetings prepared by the investigator(s). Published data must not compromise the objectives of the study. Data from individual study centers in multicenter studies must not be published separately.

15 REFERENCES

1. FDA. Emergency Use Authorization for Vaccine to Prevent COVID-19. Guidance for Industry 2021, May 25. https://www.fda.gov/media/142749/download
2. Hui DS, I Azhar E, Madani TA, et al. The continuing 2019-nCoV epidemic threat of novel coronaviruses to global health - The latest 2019 novel coronavirus outbreak in Wuhan, China. Int J Infect Dis. 2020;91:264-266.
3. Zhu N, Zhang D, Wang W, et al. A Novel Coronavirus from Patients with Pneumonia in China, 2019. N Engl J Med. 2020;382(8):727-733.
4. Guo YR, Cao QD, Hong ZS, et al. The origin, transmission and clinical therapies on coronavirus disease 2019 (COVID-19) outbreak - an update on the status. Mil Med Res. 2020;7(1):11. Published 2020 Mar 13.
5. Phan T. Novel coronavirus: From discovery to clinical diagnostics. Infect Genet Evol. 2020;79:104211.
6. Hoffmann M, Kleine-Weber H, Schroeder S, et al. SARS-CoV-2 Cell Entry Depends on ACE2 and TMPRSS2 and Is Blocked by a Clinically Proven Protease Inhibitor. Cell. 2020;181(2):271-280.e8.
7. Huang C, Wang Y, Li X, et al. Clinical features of patients infected with 2019 novel coronavirus in Wuhan, China [published correction appears in Lancet. 2020 Jan 30;:]. Lancet. 2020;395(10223):497-506.
8. Bloomberg News. Vaccine Trar. More than 2.71 billion shots given: Covid-19 Tracker Updated 22 June 2021: https://www.bloomberg.com/graphics/covid-vaccine-tracker-global-distribution/ (accessed 23 June 2021)
9. Jiang S. Don't rush to deploy COVID-19 vaccines and drugs without sufficient safety guarantees. Nature. 2020;579(7799):321.
10. Peeples L. News Feature: Avoiding pitfalls in the pursuit of a COVID-19 vaccine. Proc Natl Acad Sci U S A. 2020;117(15):8218-8221.
11. Grifoni A, Weiskopf D, Ramirez SI, et al. Targets of T Cell Responses to SARS-CoV-2 Coronavirus in Humans with COVID-19 Disease and Unexposed Individuals. Cell. 2020;S0092-8674(20)30610-3.
12. Barbara L., Miriam S. Priority List of Adverse Events of Special Interest: COVID-19. Safety Platform for Emergency Vaccines. V1.1, 05 March 2020

Appendix 5

中國醫藥大學附設醫院
CHINA MEDICAL UNIVERSITY HOSPITAL
台中市北區育德路2號
2 Yude Road, Taichung, 40447, Taiwan (R.O.C.)
TEL : 886-4-22052121

中國醫藥大學暨附設醫院研究倫理委員會

Tel: 886-4-22052121 ext: 1925　Fax: 886-4-2207-1478　台中市北區育德路 2 號

計畫修正案通過證明書

計畫名稱：一個評估 UB-612 疫苗於成年健康受試者的安全性、耐受性與免疫原性的開放性、第一期延伸試驗

計畫編號/本會編號：V-123 / CMUH110-REC1-111(AR-1)

計畫主持人：感染管制中心黃高彬主治醫師

試驗機構：中國醫藥大學附設醫院

原計畫通過日期：2021 年 07 月 22 日至 2022 年 07 月 21 日

修正案通過日期：2021 年 09 月 15 日至 2022 年 07 月 21 日

計畫書：Version 1.2, Date: 09 August, 2021

中文摘要：Version 1.2, Date: 2021-08-09

英文摘要：Version 1.2, Date: 09 Aug, 2021

受試者同意書：Version 1.4, Date: Aug. 09, 2021

個案報告表：Version 1.2, Date: 10 Aug, 2021

上述計畫已於 2021 年 09 月 15 日經中國醫藥大學暨附設醫院研究倫理委員會第一審查委員會 2021 年第 10 次審查會議審查。本委員會的運作符合優良臨床試驗準則及國內相關法令。請在持續審查必須進行前二個月向本會檢送完整之期中報告。

此計畫任何部分若經更改，必須在執行前重新提交本會審查及核准。此外，計畫主持人必須依時通報嚴重不良事件及涉及受試者或其他人風險的非預期問題。

中國醫藥大學暨附設醫院 研究倫理委員會 台中市北區育德路2號

主任委員 [signature]

中　華　民　國　一　一　〇　年　九　月　二　十　三　日

中國醫藥大學附設醫院
CHINA MEDICAL UNIVERSITY HOSPITAL
台中市北區育德路2號
2 Yude Road, Taichung, 40447, Taiwan (R.O.C.)
TEL : 886-4-22052121

Research Ethics Committee

China Medical University & Hospital, Taichung, Taiwan

Tel: 886-4-22052121 ext: 1925 Fax: 886-4-2207-1478

Clinical Trial/Human Research Approval

Amendment Review

Date：Sep. 23, 2021

Protocol Title：A Phase I, Open-Label Extension Study to Evaluate the Safety, Tolerability, and Immunogenicity of UB-612 Vaccine in healthy adult volunteers

Protocol No. / CMUH REC No.：V-123 / CMUH110-REC1-111(AR-1)

Name of Principal Investigator：Kao-Pin Hwang (Attending Physician, Center for Infection control)

Name of Institution：China Medical University Hospital

Valid Date of Original Research Project：From Jul. 22, 2021 to Jul. 21, 2022

Valid Date of Amended Research Project：From Sep. 15, 2021 to Jul. 21, 2022

Protocol：Version 1.2, Date: 09 August, 2021

Chinese Synopsis：Version 1.2, Date: 2021-08-09

English Synopsis：Version 1.2, Date: 09 Aug, 2021

Informed Consent Form：Version 1.4, Date: Aug. 09, 2021

Case Report Form：Version 1.2, Date: 10 Aug, 2021

This is to certify that the above referenced amended research project has been reviewed by the 2021 10th meeting of the Research Ethics Committee (REC) I of the China Medical University and Hospital on Sep. 15, 2021. The REC is organized under, and operates in accordance with, the Good Clinical Practices guidelines and the governmental laws and regulations. Please submit a completed progress report at least two months before the time at which continuing review must occur.

All the amendments to the research project should be re-submitted and approved by the REC BEFORE implementation. Also, the principal investigator is required to report all serious adverse events and unanticipated problems involving risks to the subjects or others on time.

Martin M-T Fuh MD, DMSci.
Chairman, Research Ethics Committee I
China Medical University & Hospital

CHINA MEDICAL UNIVERSITY & HOSPITAL
The Research Ethics Committee
No. 2 Yude Road, Taichung, Taiwan, R.O.C.

中國醫藥大學暨附設醫院研究倫理委員會
審查結果通知書

本 會 編 號	CMUH110-REC1-111(AR-1)	送審文件類型	修正案
計畫主持人	感染控制小組黃高彬主治醫師	計畫經費來源	廠商合作計畫
計 畫 名 稱	一個評估 UB-612 疫苗於成年健康受試者的安全性、耐受性與免疫原性的開放性、第一期延伸試驗		
審 查 流 程	一般審查，審查會議日期：110 年 09 月 15 日		
審 查 結 果	通過		
**初審**審查意見	一、請重新簽署受試者同意書。		

中國醫藥大學暨附設醫院
研究倫理委員會
台中市北區育德路2號

中國醫藥大學附設醫院
CHINA MEDICAL UNIVERSITY HOSPITAL
台中市北區育德路2號
2 Yude Road, Taichung, 40447, Taiwan (R.O.C.)
TEL : 886-4-22052121

中國醫藥大學暨附設醫院研究倫理委員會
Tel: 886-4-22052121 ext: 1925 Fax: 886-4-2207-1478 台中市北區育德路2號

計畫修正案通過證明書

計畫名稱：一個評估 UB-612 疫苗於成年健康受試者的安全性、耐受性與免疫原性的開放性、第一期延伸試驗

計畫編號/本會編號：V-123 / CMUH110-REC1-111(AR-2)

計畫主持人：感染管制中心黃高彬主治醫師

試驗機構：中國醫藥大學附設醫院

原計畫通過日期：2021 年 07 月 22 日至 2022 年 07 月 21 日

修正案通過日期：2021 年 10 月 13 日至 2022 年 07 月 21 日

受試者同意書：Version 1.5, Date: Sep. 23, 2021

上述計畫之修正案已於2021年10月13日經中國醫藥大學暨附設醫院研究倫理委員會第一審查委員會簡易審查通過。本委員會的運作符合優良臨床試驗準則及國內相關法令。請在持續審查必須進行前二個月向本會檢送完整之期中報告。

此計畫任何部分若經更改，必須在執行前重新提交本會審查及核准。此外，計畫主持人必須依時通報嚴重不良事件及涉及受試者或其他人風險的非預期問題。

中國醫藥大學暨附設醫院 研究倫理委員會 台中市北區育德路2號

主任委員 傅茂祖

中 華 民 國 一 一 〇 年 十 月 二 十 五 日

The Committee is organized and operates in accordance with ICH6 GCP regulations and guideline.
本委員會組織與運作皆遵守 ICH6 GCP 規定

中國醫藥大學附設醫院
CHINA MEDICAL UNIVERSITY HOSPITAL
台中市北區育德路2號
2 Yude Road, Taichung, 40447, Taiwan (R.O.C.)
TEL : 886-4-22052121

Research Ethics Committee

China Medical University & Hospital, Taichung, Taiwan

Tel: 886-4-22052121 ext: 1925 Fax: 886-4-2207-1478

Clinical Trial/Human Research Approval

Amendment Review

Date：Oct. 25, 2021

Protocol Title：A Phase I, Open-Label Extension Study to Evaluate the Safety, Tolerability, and Immunogenicity of UB-612 Vaccine in healthy adult volunteers

Protocol No. / CMUH REC No.：V-123 / CMUH110-REC1-111(AR-2)

Name of Principal Investigator：Kao-Pin Hwang (Attending Physician, Center for Infection control)

Name of Institution：China Medical University Hospital

Valid Date of Original Research Project：From Jul. 22, 2021 to Jul. 21, 2022

Valid Date of Amended Research Project：From Oct. 13, 2021 to Jul. 21, 2022

Informed Consent Form：Version 1.5, Date: Sep. 23, 2021

This is to certify that the above referenced amended research project has been expedited approved by the Research Ethics Committee (REC) I of the China Medical University and Hospital on Oct. 13, 2021. The REC is organized under, and operates in accordance with, the Good Clinical Practices guidelines and the governmental laws and regulations. Please submit a completed progress report at least two months before the time at which continuing review must occur.

All the amendments to the research project should be re-submitted and approved by the REC BEFORE implementation. Also, the principal investigator is required to report all serious adverse events and unanticipated problems involving risks to the subjects or others on time.

Martin M-T Fuh MD, DMSci.
Chairman, Research Ethics Committee I
China Medical University & Hospital

CHINA MEDICAL UNIVERSITY & HOSPITAL
The Research Ethics Committee
No. 2 Yude Road, Taichung, Taiwan, R.O.C.

中國醫藥大學暨附設醫院研究倫理委員會
審查結果通知書

本會編號	CMUH110-REC1-111(AR-2)	送審文件類型	修正案
計畫主持人	感染控制小組黃高彬主治醫師	計畫經費來源	廠商合作計畫
計畫名稱	一個評估 UB-612 疫苗於成年健康受試者的安全性、耐受性與免疫原性的開放性、第一期延伸試驗		
審查流程	簡易審查		
審查結果	通過		
初審審查意見	一、請重新簽署受試者同意書。		

中國醫藥大學暨附設醫院
研究倫理委員會
台中市北區育德路2號

Appendix 6

黃高彬
2021.9.30.

中國醫藥大學暨附設醫院
受試者同意書

您被邀請參與此研究。此同意書主要是提供您本研究之相關資訊，以便您決定是否參加本研究。計畫主持人或其指定之研究人員會為您說明研究內容並回答您的疑問。您可以提出任何和此研究有關的問題，在您的問題尚未獲得滿意的答覆之前，請不要簽署此同意書。如果您願意參與本研究，此文件將視為您的同意紀錄。即使在您同意後，您可以隨時退出本研究不需任何理由。

計畫名稱

中文：一個評估 UB-612 疫苗於成年健康受試者的安全性、耐受性與免疫原性的開放性、第一期延伸試驗

英 文：A Phase I, Open-Label Extension Study to Evaluate the Safety, Tolerability, and Immunogenicity of UB-612 Vaccine in healthy adult volunteers

執行單位：中國醫藥大學附設醫院感染科、家庭醫學科	委託單位/藥廠：聯亞生技開發股份有限公司<br>研究經費來源：聯亞生技開發股份有限公司<br>受託研究機構：晉加股份有限公司
計畫主持人：黃高彬	職稱：主治醫師
協同主持人：林文元	職稱：主治醫師
協同主持人：林伯昌	職稱：主治醫師
緊急聯絡人：黃高彬	電話：0975-681-950

受試者姓名：	病歷號碼：
性別：	出生日期：
身分證字號：	聯絡電話：
通訊地址：	

法定代理人或有同意權人之姓名：	與受試者關係：
性別：	出生日期：
身分證字號：	聯絡電話：
通訊地址：	

(一)試驗簡介：

1. 本品/技術資料：

新型冠狀病毒(SARS-CoV-2)於2019年12月起造成中國湖北省武漢市發現多起病毒性肺炎群聚，隨後於2020年1月底台灣出現第一起境外移入確診個案。此疾病在全球擴散，世界衛生組織宣布將此疫情為「國際關注公共衛生緊急事件」。

UB-612疫苗為聯亞生技開發股份有限公司所開發新型冠狀病毒預防性疫苗，疫苗含病毒棘狀融合蛋白和胜肽片段，可產生高親和力抗體與新型冠狀病毒結合，並誘發細胞免疫反應，進而達到預防新型冠狀病毒的感染。

UB-612疫苗已經進行第一期與第二期臨床試驗。本試驗為第一期試驗之延伸試驗，將用於探索施打追加接種疫苗(第三劑疫苗)之耐受性和免疫原性，以使用於短期提升體內抗體需求之應用(如短期出國暴露於高風險地區等)。

2. 本品上市狀況：

本品仍應用於人體試驗，尚未在我國上市。

(二)試驗目的：

<u>主要試驗目的</u>

- 評估追加接種UB-612疫苗於第一期臨床試驗之受試者之安全性與耐受性。
- 評估追加接種UB-612疫苗所誘發的新型冠狀病毒(SARS-CoV-2)中和抗體效價。

<u>次要試驗目的</u>

- 評估整個試驗期間的對於新型冠狀病毒的體液免疫反應。

<u>探索式試驗目的</u>

- 評估追加接種UB-612疫苗所誘發的細胞免疫反應。
- 評估對於新型冠狀病毒變異株，追加接種UB-612疫苗所引發的中和抗體反應。
- 比較初級系列疫苗接種(primary series vaccination)和追加接種(booster vaccination)誘發的體液免疫反應。
- 評估初級系列疫苗接種(primary series vaccination)和追加接種(booster vaccination)誘發的體液免疫反應之動力學。
- 評估初級系列疫苗接種和追加接種的間隔時間對免疫反應的影響。

(三)試驗之主要納入與排除條件：

執行本研究計畫的醫師或相關研究人員將會與您討論有關參加本研究的必要條件。請您配合必須誠實告知我們您過去的健康情形，若您有不符合參加本研究的情況，將不能參加本研究計畫。

1. 納入條件(參加本試驗/研究的條件):

(1) 您為參與V-122試驗，並完成兩劑疫苗接種之男性或未懷孕的女性受試者。

(2) 您為具生育能力的女性與男性應於整個試驗期間同意進行有效的避孕方式。可接受的有效避孕方式包括:

a. 男性或女性以手術方法絕育、植入式避孕、或子宮避孕器。

b. 注射避孕、避孕藥、避孕貼片、避孕環加上一種屏障避孕法*。

c. 兩種合併使用的屏障避孕法*。

*有效的屏障避孕法為避孕隔膜、男性或女性保險套、避孕海綿或殺精劑(含可殺精化學物質的藥膏或凝膠)。

(3) 您能理解受試者同意書內容的說明與可能的風險，提供簽名的受試者同意書。

(4) 您能夠理解與遵從本試驗程序與能參與每次訪視。

(5) 您的耳溫≤ 38.0°C。

(6) 您於篩選訪視時，在V-122試驗第二劑接種至少6個月後。

2. 排除條件(若您有下列任一情況，您將無法參加本試驗/研究):

(1) 您於篩選訪視時，為已懷孕女性或懷孕檢測為陽性的女性。

(2) 您為正在哺乳的女性，或計畫在追加接種一劑試驗疫苗後 90 天內哺乳的女性。

(3) 您在追加接種一劑試驗疫苗前 3 天內，經試驗主持人判斷，患有任何急性疾病。

(4) 您在追加接種一劑試驗疫苗前1個月接種過減毒性疫苗、核酸 (mRNA或DNA)或載體疫苗，或預計在追加接種一劑試驗疫苗後1個月內接種此類疫苗。

(5) 您在追加接種一劑試驗疫苗前14天內接種過次單元疫苗或去活化疫苗，或預計在追加接種一劑試驗疫苗後14天內接種此類疫苗。

(6) 經試驗主持人判斷，您有任何醫療疾病或狀況，可能會影響試驗結果或參與試驗可能會對受試者引發額外風險。

(7) 已知您曾暴露於新型冠狀病毒**，或除了UB-612，您曾接受預防新型冠狀病毒、中東呼吸症候群冠狀病毒、嚴重急性呼吸道症候群的試驗或已通過我國緊急授權使用之產品。

****: 即新型冠狀病毒的確定病例，或確定病例的接觸者，包括居家隔離者。**

(8) 您在簽署受試者同意書前12周內參與除了V-122試驗以外的其他臨床試驗。

(9) 您在追加接種一劑試驗疫苗前4個月接受免疫球蛋白和/或任何血液製劑的治療。

(10) 您在追加接種一劑試驗疫苗前6個月長期接受(≥14天連續使用)免疫抑制劑、皮質類固醇(相當於一天使用≥ 20 mg強的松(prednisone))或細胞毒性治療。

（四）試驗方法及相關檢驗：

這是一個第一期延伸試驗，評估在V-122臨床試驗完成施打兩劑UB-612疫苗(含10微克，30微克，和100微克融合蛋白與胜肽)之受試者在追加疫苗接種後的安全性、耐受性與免疫原性。

在第一期臨床試驗中完成兩劑疫苗接種的受試者，可加入本試驗。在簽署受試者同意書後，合格的受試者在V-122試驗第二劑接種至少6個月後，追加接種一劑與V-205第二期臨床試驗選擇之同劑量之疫苗。試驗共納入三組:

本試驗所使用的UB-612疫苗*劑量與預計納入受試者人數如下:

A組	追加接種一劑之UB-612疫苗(含100微克融合蛋白與胜肽，0.5毫升) ，施打於原UB-612疫苗10微克組之受試者	20位受試者
B組	追加接種一劑之UB-612疫苗(含100微克融合蛋白與胜肽，0.5毫升) ，施打於原UB-612疫苗30微克組之受試者	20位受試者
C組	追加接種一劑之UB-612疫苗(含100微克融合蛋白與胜肽，0.5毫升) ，施打於原UB-612疫苗100微克組之受試者	20位受試者

*: UB-612疫苗，含Adju-Phos® 與CpG 1寡核苷酸佐劑

總計本試驗將納入60名受試者。這個試驗將有3次訪視，包括第1次訪視(第1天，基礎值，追加疫苗接種，V-122試驗第二劑接種至少6個月後)，第2次訪視(第15天)，第3次訪視(第85天，追加疫苗接種後3個月)。

<u>注意事項</u>

1. 如果您同意參加本試驗，研究人員會請您簽署本份受試者同意書，並確認您符合參加本試驗的條件。
2. 從您參與試驗的當天開始，每次訪視都將有合格的試驗人員執行試驗流程與聯繫。
3. 於試驗期間，您不論任何理由提前退出試驗，試驗研究人員都將安排您完成最後一次的訪視之所有試驗項目。您有權利拒絕此項安排，您的決定不會引起任何影響日後醫師對您的醫療照護。

試驗步驟

第一次訪視(篩選和追加疫苗接種，第 1 天)

在試驗醫師或試驗研究人員為您提供足夠的試驗資訊，並確保您有充分的時間考慮以及詢問任何問題後，您願意讓您參與本試驗，並由您簽署本受試者同意書。在確認您已完成受試者同意書簽署並且您也保有一份副本後，試驗醫師或試驗研究人員將會進行以下試驗程序：

(1) 記錄您簽署受試者同意書的日期

(2) 為您指定一組受試者篩選編號

(3) 確認您是否符合本試驗的納入排除條件

(4) 收集您的個人基本資料（例如生日、年齡及性別）

(5) 記錄您的醫療/用藥病史

(6) 進行身體檢查，包括身高與體重

(7) 確認生命徵象

(8) 進行尿液懷孕檢測（具有生育能力女性）

(9) 接種疫苗前，收集血液檢體(共 7 毫升)，進行下列檢測:

- 常規血液檢測
- 血液生化學檢測

(10) 接種疫苗前，收集血液檢體(共 66 毫升)，進行下列檢測:

- 體液免疫原性
- 細胞免疫原性

(11) 接種疫苗前，收集血液檢體(共 2 毫升)，進行下列檢測:

- 新型冠狀病毒檢測

(12) 進行疫苗接種。接種疫苗後，您應留在試驗地點至少 30 分鐘，監測生命徵象和不良事件。

(13) 詳細地指導您如何填寫電子日誌卡

(14) 進行新型冠狀病毒感染監測

(15) 收集併用藥物/治療

第二次訪視(第 15±3 天)

將會進行以下試驗程序：

(1) 進行身體檢查

(2) 確認生命徵象

(3) 收集血液檢體(共 7 毫升)，進行下列檢測:

- 常規血液檢測
- 血液生化學檢測

(4) 收集血液檢體(共 66 毫升)，進行下列檢測:

- 體液免疫原性
- 細胞免疫原性

(5) 收集併用藥物/治療

(6) 進行新型冠狀病毒感染監測

(7) 記錄上一次訪視至此次訪視之間的不良事件或嚴重不良事件

第三次訪視(第 85±5 天)

將會進行以下試驗程序：

(1) 進行身體檢查

(2) 確認生命徵象

(3) 進行尿液懷孕檢測（具有生育能力女性）

(4) 收集血液檢體(共 66 毫升) ，進行下列檢測:

- 體液免疫原性
- 細胞免疫原性

(5) 收集血液檢體(共 2 毫升)，進行下列檢測:

- 新型冠狀病毒檢測

(6) 收集併用藥物/治療(只記錄嚴重不良事件和特殊不良事件的使用藥物)

(7) 進行新型冠狀病毒感染監測

(8) 記錄上一次訪視至此次訪視之間的不良事件或嚴重不良事件

受試者之檢體(含其衍生物)之保存、使用與再利用：

1. 檢體及剩餘檢體之保存與使用

(1) 檢體(含其衍生物)之保存與使用

為研究所需，我們所蒐集您的檢體，將依本研究計畫使用，檢體將保存於聯亞生技開發(股)公司及聯合生物製藥(股)公司，直至 20 年保存期限屆滿，我們將依法銷毀。為了保護您的個人隱私，我們將以一個試驗編號來代替您的名字及相關個人資料，以確認您的檢體及與相關資料受到完整保密。如果您對檢體的使用有疑慮，或您有任何想要銷毀檢體的需求，請立即與我們聯絡(聯絡人：黃高彬醫師電話：0975-681-950)，我們即會將您的檢體銷毀。您也可以聯繫中國醫藥大學暨附設醫院研究倫理委員會(電話：04-22052121 轉 1925、1926)，以協助您解決檢體在研究使用上的任何爭議。

(2) 剩餘檢體(含其衍生物)之再利用

您的生物檢體將會以專屬號碼進行編碼並在聯亞生技開發股份有限公司(試驗委託者)的控管下儲存最長20年，以研究UB-612疫苗反應者的生物標記，及改善治療方式。

☐ 不同意保存我的剩餘檢體，試驗結束後請銷毀

☐ 同意**保存我的剩餘檢體，逾越原同意使用範圍時，需再次得到我的同意才可使用我的檢體進行新的研究。您的剩餘檢體被送往試驗委託者的合作實驗室(包含但不限於下表中的實驗室)進行新型冠狀病毒變異株或免疫反應相關的檢驗或研究前，將以編碼處理您的剩餘檢體，合作實驗室將無法取得可辨識您身分之資料，以保護您的個資。<註：可辨識身分之資料意指可直接或間接識別身分之個資(identifiers)，包含美國 HIPPA規定的 18 種 identifiers，例如姓名、身分證字號、健保卡號、電話號碼、地址等個資。>**

實驗室名稱	機構地址
UTMB	University of Texas Medical Branch 301 University Boulevard Keiller Building, Room 2.150 Galveston, Texas, USA(美國)
Virology (Tropical Medicine	University of São Paulo, Brazil Rua Dr Enéas de

Institute, University of São Paulo)	Carvalho Aguiar 470 , CEP 05403-000(巴西)
Viral and Rickettsial Disease Laboratory (VRDL)	850 Marina Bay Parkway Richmond, CA 94804, USA(美國)
VisMederi	VisMederi Srl, Strada del Petriccio e Belriguardo, 35, 53100 Siena, Italy(義大利)
Vaccinology and Immunology Infection, Immunity & Inflammation Dept UCL GOS Institute of Child Health	UCL Great Ormond Street Institute of Child Health 30 Guilford Street London WC1N 1EH, England (英國)
中央研究院生物醫學科學研究所	台北市南港區研究院路二段128號
中央研究院生醫轉譯研究中心	台北市南港區研究院路一段130巷99號
**Viroclinics**	**Rotterdam Science Tower, Marconistraat 16, 3029 AK Rotterdam, The Netherlands(荷蘭)**
**DASA**	**Jonas Cruz de Araujo, Diagnostics da America S/A, Surubiju Avenue, 1890, Barueri, SP, Brazil(巴西), 06455-040**
**PHE Porton Down**	**Salisbury Wiltshire SP4 0JG, England(英國)**
**NEXELIS**	**525 Boul. Cartier Ouest Laval, Qulbec, Canada, H7V 3S8(加拿大)**

2. 檢體及剩餘檢體之部分類型

(1) 一般生化、血液檢驗檢體

在試驗期間，會將您的檢體送往聯亞生技開發股份有限公司(試驗委託者)委託的中央實驗室中國醫藥大學暨附設醫院分析，此機構地址為台中市北區育德路2號，中央實驗室會在分析後立即將分析結果提供給試驗中心，若有剩餘的檢體，將會儲存直到檢驗結果複驗完畢即銷毀，不會長期儲存。

(2) 抗體/細胞免疫試驗

在試驗期間，會將您的檢體送往聯亞生技開發(股)公司(試驗委託者)分析實驗室及聯合生物製藥(股)公司生物分析實驗室進行處置、處理與進一步分析。聯亞生技開發(股)公司機構地址為新竹縣竹北市生醫路二段6-1號5樓，聯合生物製藥(股)公司機構地址為新竹

縣竹北市生醫路二段12號1樓。完成試驗後，若有剩餘檢體，將儲存於聯亞生技開發(股)公司及聯合生物製藥(股)公司，直到至少完成臨床試驗報告為止，最長將保存20年。

(3) 中和試驗(neutralization test, NT)

在試驗期間，會將您的檢體送往聯亞生技開發股份有限公司(試驗委託者)委託的中央實驗室中央研究院生物醫學科學研究所進行處置、處理與進一步分析。此機構地址為台北市南港區研究院路二段128號。完成試驗後，若有剩餘檢體，將儲存直到至少完成臨床試驗報告為止，最長將保存20年。

(4) 偽病毒中和抗體檢測

在試驗期間，會將您的檢體送往聯亞生技開發股份有限公司(試驗委託者)委託的中央實驗室中央研究院生醫轉譯研究中心進行處置、處理與進一步分析。此機構地址為台北市南港區研究院路一段130巷99號。完成試驗後，若有剩餘檢體，將儲存直到至少完成臨床試驗報告為止，最長將保存20年

(5) 遺傳學檢體

在試驗期間，若發生與UB-612疫苗相關的嚴重不良反應或嚴重度等於或大於三級的過敏反應，您的DNA檢體將用於HLA分型檢驗**(人類白血球組織抗原分型檢驗，將檢驗您的人類白血球組織抗原分型)，主要檢測第一型(HLA-A,B,C)及第二型(HLA-DR,DP,DQ)，**會將您的檢體送往聯亞生技開發股份有限公司(試驗委託者)委託的中央實驗室有勁基因股份有限公司分析，此機構地址為新北市樹林區復興路376-5號，2021年8月會搬遷至新北市汐止區新台五路一段93號26樓之6，中央實驗室會在分析後立即將分析結果提供給試驗中心，若有剩餘的檢體，將會儲存直到檢驗結果複驗完畢即銷毀，不會長期儲存。

(五)可能產生之副作用、發生率及處理方法：

1. 與試驗藥物相關的風險（本試驗疫苗的副作用）：

冠狀病毒疫苗的開發

過去針對與SARS-CoV-2病毒相同屬於人類冠狀病毒的SARS-CoV(嚴重急性呼吸綜合症冠狀病毒(SARS冠狀病毒))的疫苗研究發現，接種過SARS-CoV疫苗的小鼠在暴露到SARS-CoV後會發生過度免疫反應而產生病變，因此不得不停止這種疫苗的開發。所以，成功的人類冠狀病毒疫苗不只要產生可以抑制病毒的免疫反應，更要避免過度免疫產生的副作用。

疫苗相關的風險

接種疫苗可能會出現注射部位的不良反應(例如疼痛、硬化腫脹、皮疹發紅、過敏反應、蜂窩性組織炎)，或全身性不良反應(例如發燒、腹瀉、疲倦、噁心/嘔吐、厭食、咽喉痛、

頭痛、咳嗽、關節痛、非注射部位疼痛、非注射部位搔癢、皮膚和黏膜異常、急性過敏反應、昏厥、急性支氣管痙攣、呼吸困難)。

第一期臨床試驗已經有60位受試者接種兩劑疫苗(含10微克、30微克、100微克融合蛋白)，第一次接種疫苗後第一次7天內不良反應在低劑量疫苗的發生率為65%，中劑量疫苗為70%，高劑量疫苗為80%。第二次接種疫苗後第一次7天內不良反應在低劑量疫苗的發生率為55%，中劑量疫苗為60%，高劑量疫苗為35%。常見的預期性不良事件為疼痛、疲倦、腹瀉，但大部分的預期性不良事件都是輕微的，大約於2天之內症狀都會緩解。安全實驗室數值並沒有顯示有任何的臨床顯著不正常數值，也沒有任何的嚴重不良事件或特殊不良事件被通報。

疾病增強(disease enhancement) 的風險

SARS-CoV-2候選疫苗也可能會有引發疾病增強(disease enhancement) 的風險，包括抗體依賴性增強(antibody-dependent enhancement)或疫苗相關聯的增強的呼吸道疾病(vaccine-associated enhanced respiratory disease)。在先前研發SARS疫苗時，在數個SARS-CoV動物攻毒試驗(包括鼠類、雪貂、猴類)當中，有發現疾病增強的現象。疾病增強反應的免疫病理現象包括TH2偏向及嗜酸性白血球的肺部浸潤。但是目前已發表的新型冠狀病毒肺炎疫苗研究，仍尚未發現類似的疾病增強現象。

本試驗疫苗於數個藥理試驗呈現不一致的TH1/TH2 (輔助型T細胞1/輔助型T細胞2)免疫反應偏向，雖未一致偏向TH2，仍已規劃並正在執行動物攻毒模型試驗，以進一步排除疾病增強風險。依據文獻指出，組成複雜或容易引起非中和抗體之抗原，如不活化病毒或整片段之蛋白(包含S蛋白與N蛋白)，與易引起偏向Th2免疫反應之佐劑成分，如鋁製佐劑，皆較有可能引起疾病增強。本試驗疫苗的主要抗原為S蛋白上之RBD 區域，已有多篇文獻指出，針對S-RBD設計之SARS與MERS(中東呼吸症候群冠狀病毒)疫苗從未於試驗動物模型上引發疾病增強現象。本試驗疫苗雖使用易引起偏向Th2免疫反應之佐劑，但由動物實驗證實，也同時引起偏向Th1之反應，因此發生疾病增強應屬低風險。且已於多種動物模型中證實，能誘發高效價之中和抗體，於細胞培養中亦能有效抑制新冠病毒感染。

建議您在有效疫苗上市前或本試驗疫苗的產品資訊有進一步更新前，盡量避免暴露於可能感染病毒的環境。研究團隊將會在試驗中執行相關安全性監測。若有任何關於本試驗疫苗與疾病增強風險相關之任何最新資訊，將即時更新並提供給您。

疫苗佐劑相關的風險

本試驗疫苗所使用的佐劑含Adju-Phos®，是屬於一種磷酸鋁類的佐劑。磷酸鋁類佐劑已經使用超過半個世紀，具有相當的安全性。由於此類佐劑可誘導免疫反應，因此可能會造成局部發炎反應，例如在注射部位產生輕微而短暫的疼痛、發紅以及腫脹。

2. 與試驗/研究過程相關的風險：

抽血

本試驗需要抽血檢驗。抽血可能引起一些不適和瘀血。整個試驗期間3個月，共需抽血 216毫升。若需要進行HLA分型檢驗(人類白血球組織抗原分型檢驗)，將額外抽血3毫升。

在接種疫苗過程中，可能會出現一些尚未在已完成試驗中發現的副作用。一般而言，接種某一新疫苗總是會有一定的風險，但是計畫主持人會採取一切措施預防風險的發生。**計畫主持人鼓勵您報告您遇到的任何不適。**

(六)其他替代療法及說明：

您不是非參加不可，若不參加研究，預防措施與其他呼吸道感染相同，包括：勤洗手、減少觸摸眼口鼻、注意咳嗽禮節、妥善處理口鼻分泌物等，避免出入公共場所，並不要接觸野生動物。

如果您對於本試驗疫苗有任何的疑問，您可以提出來向您的試驗醫師討論。

(七)試驗預期效益：

依據臨床前試驗結果，預期本試驗疫苗對您可能可以產生抗體，預防新型冠狀病毒感染，但因每個人體質不同也有可能不會產生療效，故參加本試驗可能不會有直接的好處。

但是您參加本試驗，可協助我們獲得更多資訊，以瞭解UB-612疫苗的安全性與免疫力。

(八)試驗進行中受試者之禁忌、限制與應配合之事項：

禁止使用的藥物

以下藥物請勿在試驗期間使用：

- 整個試驗期間使用試驗產品(包括藥物或疫苗)
- 禁止使用免疫球蛋白和/或任何血液製劑
- 禁止使用免疫抑制劑、皮質類固醇或細胞毒性治療
- 本試驗疫苗以外的新型冠狀病毒疫苗
- 第29天後可接種其他已經上市的疫苗(若為次單元疫苗或去活化疫苗可於第15天後接種)，但應與第85天間隔至少一個月

允許使用的藥物

若您的藥物或治療必須常規使用，經試驗醫師判斷不會影響本試驗疫苗的免疫原性、臨床療效與安全性，則可以正常使用。您有任何關於在試驗期間可允許使用何種藥物或治療的問題，請詢問您的試驗醫師。

懷孕或母乳哺乳的風險

目前未知本試驗疫苗對於未出生胎兒的影響，因此：

- 您為具生育能力的女性受試者 (除非手術絕育或停經)，或您為男性受試者應於整個試驗期間同意進行有效的避孕方式，同意進行有效的避孕方式(例如子宮內節育器、荷爾蒙療法或避孕套)。
- 若您為具生育能力的女性，將請您進行懷孕檢測，結果必須為陰性，方可參與試驗。
- 若您為懷孕的女性，將被告知不可參與本試驗。
- 若您在試驗期間懷孕，請盡速通知試驗人員，並且停止施打本疫苗。
- 基於安全性考量，若您為女性受試者而在試驗期間懷孕，或您為男性受試者而您的性伴侶在試驗期間懷孕(將請您的懷孕性伴侶需簽署另外一份同意書)，您與您的胎兒將會被追蹤監測至分娩，除非另有醫學指示。

您應向您的配偶或性伴侶告知您有參與此試驗與相關風險:

簽名：__________日期：__________________

(九)機密性：

中國醫藥大學附設醫院將依法把任何可辨識您的身分之紀錄與您的個人隱私資料視為機密來處理，不會公開。研究人員將以一個研究代碼代表您的身分，此代碼不會顯示您的姓名、國民身分證統一編號、住址等可識別資料。如果發表試驗/研究結果，您的身分仍將保密。您亦瞭解若簽署同意書即同意您的原始醫療紀錄可直接受監測者、稽核者、研究倫理委員會及主管機關檢閱，以確保臨床試驗/研究過程與數據符合相關法律及法規要求，上述人員並承諾絕不違反您的身分之機密性。除了上述機構依法有權檢視外，我們會小心維護您的隱私。由於試驗藥物可能同時申請美國臨床試驗，依美國藥品管理規定，試驗結果將公佈於公開的臨床試驗資訊網站：Clinicaltrials.gov (美國)，但您的個人資料仍將保密，該網站只會有試驗之結果摘要，您可以在任何時候搜尋該網站。

在試驗/研究期間，依據計畫類型與您所授權的內容，我們將會蒐集與您有關的病歷資料、醫療紀錄、量表、問卷等資料與資訊，並以一個編號來代替您的名字及相關個人資料。前述資料若為紙本型式，將會與本同意書分開存放於研究機構之上鎖櫃中；若為電子方式儲存或建檔以供統計與分析之用，將會存放於設有密碼與適當防毒軟體之專屬電腦內。

中國醫藥大學暨附設醫院
受試者同意書

這些研究資料與資訊將會保存至藥品於我國上市後至少兩年，若試驗疫苗終止研發則保存至試驗正式停止後至少二年，至多將保存至疫苗上市後或試驗正式停止後二年。
上述資料與資訊若傳輸至國外分析與統計，您仍會獲得與本國法規相符之保障，計畫主持人與相關團隊將盡力確保您的個人資料獲得妥善保護。

(十)損害補償與保險：

1. 如依本研究所訂臨床試驗計畫，因發生不良反應造成損害，由聯亞生技開發股份有限公司負補償責任。但本受試者同意書上所記載之可預期不良反應，不予補償。
2. 如依本研究所訂臨床試驗計畫，因而發生不良反應或損害，贊助廠商將依法負責損害賠償責任。本醫院願意提供專業醫療照顧及醫療諮詢。您不必負擔治療不良反應或損害之必要醫療費用。
3. 除前二項補償及醫療照顧外，本研究不提供其他形式之補償。若您不願意接受這樣的風險，請勿參加試驗。
4. 您不會因為簽署本同意書，而喪失在法律上的任何權利。
5. 本研究有投保責任保險。

(十一）受試者權利：

1. 試驗過程中，與您的健康或是疾病有關，可能影響您繼續接受臨床試驗意願的任何重大發現，都將即時提供給您。
2. 如果您在試驗過程中對試驗工作性質產生疑問，對身為患者之權利有意見或懷疑因參與研究而受害時，可與本院之研究倫理委員會聯絡請求諮詢，其電話號碼為：04-22052121轉1925、1926。
3. 為進行試驗工作，您必須接受黃高彬醫師的照顧。如果您現在或於試驗期間有任何問題或狀況，請不必客氣，可與在中國醫藥大學附設醫院兒童感染科的黃高彬醫師聯絡（24小時聯繫電話：0975-681-950）。
4. 參加試驗研究計畫之補助：本計畫將在每次訪視提供交通費3000元給您，整個試驗預計給予您9000元。
5. **如果您同意參與本研究，請簽署同意書。您將收到一份受試者同意書的副本，以供記錄。感謝您的時間。**。

(十二）試驗之退出與中止：

您可自由決定是否參加本試驗；試驗過程中也可隨時撤銷同意，退出試驗，不需任何理由，且不會引起任何不愉快或影響其日後醫師對您的醫療照顧。

計畫主持人或贊助廠商亦可能於必要時中止該試驗之進行。

（十三）簽名：

1. 計畫主持人、或協同主持人已詳細解釋有關本研究計畫中上述研究方法的性質與目的，及可能產生的危險與利益。

   計畫主持人/協同主持人簽名：____________日期：________年____月____日

2. 受試者已詳細瞭解上述研究方法及其所可能產生的危險與利益，有關本試驗計畫的疑問，業經試驗主持人詳細予以解釋。本人同意接受為臨床試驗計畫的自願受試者。

   受試者簽名：___________________________日期：________年____月____日

   法定代理人簽名：_______________________日期：________年____月____日

   * 受試者為無行為能力(未滿七歲之未成年人者或禁治產人)，由法定代理人為之；禁治產人，由監護人擔任其法定代理人。

   * 受試者為限制行為人者(滿七歲以上之未成年人)，應得法定代理人之同意。

   有同意權人簽名：_______________________日期：________年____月____日

   * 受試者雖非無行為能力或限制行為能力者，但因意識混亂或有精神與智能障礙，而無法進行有效溝通和判斷時，由有同意權之人為之。前項有同意權人為配偶及直系親屬。

3. 見證人

   見證人簽名：____________________________日期：________年____月____日

   身分證字號：　　　　　　　　　　　　聯絡電話：

   通訊地址：

   * 受試者、法定代理人或有同意權之人皆無法閱讀時，應由見證人在場參與所有有關受試者同意之討論。並確定受試者、法定代理人或有同意權之人之同意完全出於其自由意願後，應於受試者同意書簽名並載明日期。試驗相關人員不得為見證人。

流程表:

訪視	1	2	3/ET
檢測項目	追加疫苗接種[d]		追蹤訪視[e]
Day	1	15<br>±3 天	85<br>±5 天
獲得受試者同意書	X		
納入/排除條件	X		
基本資料	X		
醫療病史	X		
身體檢查[a]	X	X	X
生命徵象	X	X	X
**實驗室檢測 (安全性)**			
血液常規檢測	X	X	
血液生化學檢測	X	X	
懷孕檢測[b]	X		X
**實驗室檢測 (免疫原性)**			
體液免疫原性	X	X	X
細胞免疫原性	X	X	X
**實驗室檢測 (新型冠狀病毒檢測)**			
新型冠狀病毒血清抗體(IgG)檢測[f]	X		**X**
疫苗接種	X		
指導使用電子日誌卡[c]	X		
不良事件/嚴重不良事件	X	X	X
新型冠狀病毒感染監測		X	X
併用藥物/治療	X	X	X[c]

a: 身高與體重僅在第一次訪視測量。
b: 篩選訪視將在施打疫苗前執行懷孕檢測。但是已絕經或以手術絕育的女性將不作此檢測。若尿液檢測為陽性，應以血清懷孕檢測再次確認。以血清懷孕檢測替代尿液檢測將不視為試驗偏差。
c: 只記錄嚴重不良事件和特殊不良事件的使用藥物。
d: 為 V-122 臨床試驗第二劑接種至少 6 個月後。
e: 追加接種疫苗後 3 個月
f: 以聯亞(UBI)新冠肺炎病毒(SARS-CoV2)結構蛋白抗體 ELISA 診斷試劑盒進行新型冠狀病毒血清抗體(免疫球蛋白 G(IgG))檢測
ET: 提早退出試驗

Appendix 7

[illegible]
06 Sep. 2021.

CLINICAL STUDY PROTOCOL

A Phase II, Placebo-controlled, Randomized, Observer-blind Study to Evaluate the Immunogenicity, Safety, and Tolerability of UB-612 Vaccine against COVID-19 in Adolescent, Younger and Elderly Adult Volunteers

**Protocol Number:**	V-205
**EudraCT Number:**	Not Applicable
**Investigational Product:**	UB-612
**Phase:**	II
**Sponsor:**	聯亞生技開發股份有限公司<br>United Biomedical, Inc., Asia (UBI Asia),<br>Hsinchu County, Taiwan
**Protocol Date:**	06 September, 2021
**Protocol Version:**	3.0

CONFIDENTIAL
This protocol may not be reproduced or communicated to a third party without the written permission of United Biomedical, Inc., Asia

1 PROTOCOL APPROVAL SIGNATURES

Protocol Title: A Phase II, Placebo-controlled, Randomized, Observer-blind Study to Evaluate the Immunogenicity, Safety, and Tolerability of UB-612 Vaccine against COVID-19 in Adolescent, Younger and Elderly Adult Volunteers

Protocol Number: V-205

This study will be conducted in compliance with the clinical study protocol (and amendments), International Council for Harmonisation (ICH) guidelines for current Good Clinical Practice (cGCP) and applicable regulatory requirements.

Sponsor Signatory

United Biomedical, Inc., Asia (UBI Asia),
No. 45, Guangfu N. Rd., Hukou Township,
Hsinchu County 303, Taiwan (R.O.C.)

____
Signature

____
Date

Reviewed and Approved by:

Sponsor's Representative

Thomas P. Monath, MD, FACP, FASTMH
Vaxxinity Inc.
1717 Main Street, Suite 3388
Dallas, TX 75201
Mobile: (978) 549-0708
Email: tom@vaxxinity.com

聯亞生技開發股份有限公司

06. Sep. 2021

____

____

2 SYNOPSIS

Protocol Number:

V-205

Title:

A Phase II, Placebo-controlled, Randomized, Observer-blind Study to Evaluate the Immunogenicity, Safety, and Tolerability of UB-612 Vaccine against COVID-19 in Adolescent, Younger and Elderly Adult Volunteers

Investigational Product:

UB-612 vaccine

Phase:

Phase II

Objectives:

Primary Objectives

- To evaluate the SARS-CoV-2 neutralizing antibody titer induced by UB-612 vaccine
- To evaluate the safety and tolerability of the UB-612 vaccine after vaccination

Secondary Objectives

- To evaluate the immune response to SARS-CoV-2 during the study
- To evaluate the lot consistency of immune responses induced by 3 independent batches of vaccine

Exploratory Objectives

- To evaluate the T cell function induced by UB-612
- To evaluate the safety and immunogenicity of the UB-612 vaccine in adolescents
- To evaluate the efficacy of UB-612 vaccine
- To describe the serological responses to the UB-612 vaccine in confirmed and/or severe COVID-19 cases
- **To evaluate antibody against SARS-CoV-2 antigens**

Study Design:

This is a phase II, observer-blind, multiple-centre, randomized, placebo-controlled study to evaluate the immunogenicity, safety, tolerability and lot consistency of 2 doses of UB-612 vaccine in adolescent, younger and elderly adults. Around 3850 adult subjects will be randomized to be composed of the core group for EUA application, while around 385 adolescents will be randomized to be the supplementary group for broader indication. All subjects will be randomly allocated to receive 2 doses of 100 µg vaccine or placebo in a 6:1 ratio, including 462 aged from >18 to < 65 years old, evaluable subjects in the lot-to-lot consistency group. As for immunogenicity, at least 350 evaluable young adults (aged >18 to < 65 years old) and 154 evaluable elderly (aged ≥ 65 years old) will be enrolled for descriptive analysis. Subjects in immunogenicity group should be enrolled first. All subjects will be included in the safety group, and it is intended that a minimum of 770 subjects will be randomized to be in the ≥ 65-year stratum. Adolescents will start to enrol after recruitment of the core group has been completed. 385 adolescents will be randomized to be allocated in 6:1 ratio, in which 154 evaluable adolescents will have immunogenicity data to compare with adults.

It will be consisted of 7 clinical visits and one long-term follow-up visit. All subjects will have blood test for safety before and after full vaccination. Subjects in lot-to-lot consistency and immunogenicity group will also have blood drawn for immune response, in which tests for T cell function will be optional. Subjects will come to the clinics at Visit 1 for screening, Visit 2 (Day 1, baseline) for randomization and 1st vaccination, Visit 3 (Day 29) for 2nd vaccination, Visit 4 (Day 57) for safety check and immunogenicity assessment, and Visit 5 (Day 197) for safety check and assessment of the persistence of immune response. **Subjects will also be unblinded at Visit 5, subjects in placebo group will withdraw from the study and subjects in vaccine group will be encouraged to have 3rd dose**

of vaccination (Day 197~Day 242) at Visit 6. Those who received 3rd dose will have Visit 7 (14 days after Visit 6) to check the booster effect. After Day 197, subjects will enter the long-term follow-up with a safety call bi-monthly. Subjects in lot-to-lot and immunogenicity group will be encouraged to visit site at Day 365 to check immune persistence. Subjects will be expected to participate for up to a maximum of approximately 13 months. Unblinding will be carried out after **subjects** in core group or supplementary group have completed Visit 5. For females of child-bearing potential, a urine pregnancy test will be performed before **each** vaccination and will be found negative. Female subjects or the female partners of male subjects who are pregnant during the study period will be followed the pregnancy outcomes.

Before any study-related procedures are performed, voluntary, written, study-specific informed consent will be obtained from the subjects. Subject will be screened at Visit 1. All subjects will have blood sampling for hematology, and biochemistry. After checking the eligibility of the subject at Visit 2, the randomization number and adjoining study intervention allocation will be assigned. **Subjects in lot-to-lot consistency and immunogenicity group will have blood drawn for immunogenicity before vaccination.** Unblinded site staff member(s) will dispense/administer 0.5 mL UB-612/placebo into the deltoid muscle of the preferably nondominant arm. Blinded site staff must observe the subject for at least 30 minutes after study vaccine administration for changes in vital signs or any acute anaphylactic reactions. The investigator will provide instructions for reactogenicity e-diary completion and ask the subject to complete the reactogenicity e-diary from Day 1 to Day 7, skin allergy reaction from Day 1 to Day 14, with Day 1 being the day of vaccination. Participants will be instructed to contact the site staff or investigator immediately if he or she experiences any of the Grade 3/4 AEs from Day 1 to Day 7 to determine if an unscheduled reactogenicity visit is required. Apart from reactogenicity, allergic skin rash should be monitored for 14 days post vaccination. **Once subject encountered the Grade 3/4 allergic skin rash, , site staff should call for detail to evaluate the necessarily of unscheduled visit. Investigators could ask for HLA typing survey if he/she concerned about the possibility of hypersensitivity. Of course, IDMC could also request HLA typing once they reviewed the safety data.**

At Visit 3 (Day 29), the 2nd study vaccine administration will be done and subjects will be closely monitored for 30 minutes after vaccination for changes in vital signs or acute anaphylactic reaction. The **7/14**-day post-vaccination e-diary will be given to subjects with a suitable instruction. For females of child-bearing potential, a urine pregnancy test will be performed before vaccination and found negative. At Visit 4 (Day 57, 1-month follow-up visit after 2nd vaccination), collect sample for safety and immunogenicity, review the participants' reactogenicity e-diary data and record any unsolicited AE.

All subjects will come back on Day 197, Visit 5, for immunogenicity persistence check in immunogenicity group and safety check for safety group. **Once subject completes Visit 5, he or she will be unblinded. Subjects in placebo group will withdraw from the study. Subjects who received UB-612 vaccine will be invited to join the extension study to determine the durability of the immune response and long-term safety after unblinding. Meanwhile, subjects in vaccine group will be encouraged to have a third dose of UB-612. All subjects who received UB-612 vaccine will be followed up for 12 months.**

All subjects will receive safety calls on Day 8, Day 15, Day 22, Day 36, Day 43, Day 64, Day 71, Day 78, Day 85, Day 253, and Day 309 that will serve both to monitor for unsolicited AEs or/and to monitor for symptoms of COVID-19. **After Day 57, each week (i.e., every 7 days) the subjects will receive a prompt on their smartphone device to regularly surveillance for signs and symptoms of COVID-19 and SAE. Subjects who received 3rd dose of UB-612 will receive an extra safety call on 7 days post 3rd vaccination.**

If a Grade 3 local reaction, systemic event, or fever are reported in the reactogenicity e-diary, a telephone contact should occur to ascertain further details and determine whether a site visit is clinically indicated. If suspected Grade 4 local reaction systemic event and /or fever is reported in the reactogenicity e-diary, a site visit should occur to confirm whether the event meets the criteria for Grade 4. All subjects were encouraged to contact site staff once they encountered Grade 3/4 AE. Allergic skin reaction should be monitored for 14 days post vaccination and should be contacted regardless of severity.

If a subject experiences a febrile illness associated with respiratory symptoms, he or she is instructed to contact the site immediately. Subjects may utilize a COVID-19 illness e-diary to prompt him/her to report any symptoms.

Number of Subjects:

At least 3850 adult subjects will be enrolled into core group, and 385 adolescents will be recruited for supplementary group. All subjects will randomly receive the 100 µg of UB-612 vaccine or placebo with allocation rate 6:1.

Treatment:

The UB-612 vaccines (200 μg/mL), 6.5 mL/vial, containing Adju-Phos® and CpG1 adjuvant, and the placebo, normal saline 0.9%, will be administered 0.5 ml by an intramuscular (IM) injection, 28 days apart (on Day 1 and 29)**, and a 3rd dose of UB-612 will be given after unblinding if subjects qualified.**

Duration of Study: 13 months

Study Population:

To be eligible for study entry, subjects must satisfy all of the following inclusion criteria:

1. Healthy male or non-pregnant female between the age of 12 to 85 years at time of enrolment.
2. Women of childbearing potential and men must agree to practice medically effective contraception from first vaccination until 3 months after the last vaccination. The acceptable effective contraception methods include:
   a. Male or female sterilization, implant, or intrauterine device;
   b. Injectable, pill, patch, ring plus one barrier method*;
   c. Two combined barrier methods*.

   *Effective barrier methods are diaphragm, male or female condoms, sponge, or spermicides (creams or gels that contain a chemical to kill sperm).
3. Able to understand the content and possible risks of informed consent and willing to sign the Informed Consent Form (ICF).
4. Able to understand and agrees to comply with all study procedures and be available for all study visits.
5. Ear temperature ≤ 38.0°C.
6. Healthy participants** who are determined by medical history, physical examination, and clinical judgment of the investigator to be eligible for inclusion in the study. In the investigator's clinical judgement, participant may have a stable and well-controlled comorbidity associated with an increased risk of progression to severe COVID-19.

   ** Healthy participants with preexisting stable disease, defined as disease not requiring significant change in therapy or hospitalization for worsening disease during the 12 weeks before enrolment and unlikely to require a significant change in therapy or hospitalization in the six months following enrollment, can be included.

Subjects will be excluded from the study if one or more of the following exclusion criteria is applicable:

1. History of anaphylaxis, urticarial, or other significant adverse reaction requiring medical intervention after receipt of a vaccine.
2. Female who is pregnant or positive in pregnancy test at screening or just prior to each vaccination administration.
3. Female who is breast-feeding or plans to breastfeed from the time of the first vaccination through 60 days after the last vaccination.
4. Any acute illness, as determined by the study investigator 3 days before first vaccination (these subjects can be re-scheduled).
5. Any major surgery one month before first vaccination (these subjects can be -rescheduled).
6. Known HIV antibody positive
7. Known active hepatitis B and hepatitis C disease. Active hepatitis means liver aminotransferase (AST and/or ALT) greater than 3xULN, and/or total bilirubin greater then 3xULN at screening.
8. Previous exposure to SARS-CoV-2 or receipt of an investigational or licensed product for the prevention of COVID-19, MERS or SARS.
9. Have history of Guillain-Barre syndrome.

10. Subjects who take part in another clinical study within 12 weeks prior to the day of informed consent.
11. Immune deficiency/disorder, whether due to genetic defect, immunodeficiency disease or immunosuppressive therapy
12. Subjects who plan to or are undergoing anti-cancer therapy
13. Platelet disorder or other bleeding disorder may cause injection contraindication.
14. Prior chronic administration (defined as ≥ 14 day of continuous use) of immunosuppressant or corticosteroids (equivalent to ≥ 20 mg daily of prednisone), cytotoxic treatment in last 6 months before first vaccination.
15. Prior administration of immunoglobulins and/or any blood products in last 4 months before first vaccination.
16. Receipt of any seasonal or pandemic influenza vaccine within 14 days, or any other nonstudy vaccine within 28 days, before study intervention administration.
17. Anticipated receipt of any seasonal or pandemic influenza vaccine within 14 days, or any other nonstudy vaccine within 28 days, after study intervention administration.
18. Receipt of short-term (<14 days) systemic corticosteroids. Study intervention administration should be delayed until systemic corticosteroid use has been discontinued for at least 28 days. Inhaled/nebulized, intra-articular, intrabursal, or topical (skin or eyes) corticosteroids are permitted.
19. Loss or donation of blood over 500 mL within 3 months prior to Screening Visit or intention to donate blood or blood products for transfusion during the study.
20. Any medical disease or condition that, in the opinion of the study investigator, may confound the results of the study or pose an additional risk to the subjects by their participation in the study.
21. Employees at the investigator's site, of the Sponsor or the contract research organization (CRO) **who** directly involved in the conduct of the study.

Primary Endpoint(s):

Immunogenicity Endpoint(s)

- Geometric mean titer (GMT) of SARS-CoV-2 neutralizing antibody on Day 57
- Seroconversion rate (SCR) of SARS-CoV-2 neutralizing antibody on Day 57

Safety Endpoint(s)

- Local reactions for up to 7 days following each dose
- Systemic events for up to 7 days following each dose
- Unsolicited AEs from Day 1 to Day 57
- MAAEs and SAEs from Day 1 to Day **365**
- AESIs and ADEs from Day 1 to Day **365**

Secondary Endpoint(s):

Immunogenicity Endpoint(s)

- Seroconversion rate (SCR) of antigen-specific antibody (Anti-S1-RBD) on Day 57
- Geometric mean titer (GMT) of SARS-CoV-2 neutralizing antibody on Day 197 and 365
- Geometric mean titer (GMT) of antigen-specific antibody (Anti-S1-RBD) on Day 57, 197 and 365
- Geometric mean fold increase in **SARS-CoV-2 neutralizing antibody and** antigen-specific antibody (Anti-S1-RBD) on Day 57, 197 and 365
- Lot consistency as assessed by the comparisons of the GMT of SARS-CoV-2 neutralizing antibody on Day 57 induced by 3 independent UB-612 vaccine clinical materials. The 95% confidence intervals between groups will be within the margin of 0.5 to 2.

Safety Endpoint(s)

- Changes of safety laboratory measures

Exploratory endpoint(s):

- T cell responses to UB-612 measured by ELISpot and flow cytometric assays on Day 57
- **T cell responses to UB-612 measured by ELISpot and flow cytometric assays on 14 days post 3rd dose of UB-612**
- **Geometric mean titer (GMT) of SARS-CoV-2 neutralizing antibody on 14 days post 3rd dose of UB-612**
- **Geometric mean titer (GMT) of antigen-specific antibody (Anti-S1-RBD) on 14 days post 3rd dose of UB-612**
- **Geometric mean fold increase in SARS-CoV-2 neutralizing antibody and antigen-specific antibody (Anti-S1-RBD) on 14 days post 3rd dose against pre-3rd dose baseline**
- **Geometric mean titer (GMT) of SARS-CoV-2 neutralizing antibody and antigen-specific antibody (Anti-S1-RBD) on Day 57, Day 197 and Day 365 in adolescents**
- **Seroconversion rate (SCR) of SARS-CoV-2 neutralizing antibody and antigen-specific antibody (Anti-S1-RBD) on Day 57 in adolescents**
- **Geometric mean fold increase in SARS-CoV-2 neutralizing antibody and antigen-specific antibody (Anti-S1-RBD) on Day 57, Day 197, and Day 365 in adolescents**
- Local reactions for up to 7 days following each dose in adolescents
- Systemic events for up to 7 days following each dose in adolescents
- Unsolicited AEs from Day 1 to Day 57 in adolescents
- MAAEs and SAEs from Day 1 to Day 365 in adolescents
- AESIs and ADEs from Day 1 to Day 365 in adolescents
- Changes of safety laboratory measures in adolescents
- COVID-19 incidence per 1000 person-years of follow-up based on PCR test
- To describe the anti-S1-RBD IgG levels and SARS-CoV-2 neutralizing titers to UB-612 in confirmed and/or severe COVID-19 cases
- **To detect antibody against SARS-CoV-2 antigens derived from S2, N, and M protein**

Sample Size Determination:

Consider a 10% drop-out rate, around 3850 adult subjects in core group will be recruited and randomized with 6:1 allocation rate (100 µg and placebo) and around 385 adolescents will be recruited for supplementary group and randomized with same allocation ratio.

Sample Size for Safety Evaluation

For safety outcomes, the following table shows the probability of observing at least 1 SAE for a given true event rate of a particular SAE. For example, if the true SAE rate is 0.01%, with 2400 young adult subjects, 600 elderly adult subjects, and 300 adolescent subjects received UB-612 vaccine, there are 21.3%, 5.8%, and 3.0% probabilities of observing at least 1 SAE. Overall, probability of observing at least 1 SAE is 25.9% with true SAE rate of 0.01% for all 3000 adult subjects received study vaccine in core group.

True SAE Rate	N=300	N=600	N=2400	N=3000
0.001%	0.003	0.006	0.024	0.030
0.010%	0.030	0.058	0.213	0.259

0.100%	0.259	0.451	0.909	0.950

Sample Size for Lot-to-Lot Consistency

Younger adults are selected for this analysis since they should have immune responses less affected by immune senescence, which tends to result in more variable responses. The sample size is driven by the objective to demonstrate the consistency of GMT ratio (GMTR) with the 3 consecutive manufacturing lots of the UB-612 vaccine. The clinical lot-to-lot consistency will be tested for the three pair-wise comparisons by computing the two-sided 95% CI on the GMTR. If all confidence intervals are within the pre-defined clinical limits of [0.5, 2.0], one can conclude that the lots are consistent.

Assume the expected GMTR of 1 and a value of 0.6 for the standard deviation (SD) of the decimal logarithmic transformation (log base 10) of antibody titers (log10(GSD) is about 0.2 to 0.6; refer to following table of the V-122 interim result). In order to have at least 90% power to achieve the lot-to-lot consistency, the estimated sample size should ensure beta less than 3.3% (Bonferroni adjustment of beta for 3 comparisons between 3 lots). With the parameters above it estimates a minimum evaluable sample size per lot of 115 subjects. Consider a 10% drop-out rate, a total of 396 subjects (132 subjects per lot) will be needed for ensuring overall lot-to-lot consistency with at least 90% overall power.

**Antibody Titers**	**UB-612 10 µg**		**UB-612 30 µg**		**UB-612 100 µg**	
	GSD	log10(GSD)	GSD	log10(GSD)	GSD	log10(GSD)
Neutralizing	3.286	0.52	2.438	0.39	1.670	0.223
Anti-S1-RBD	4.185	0.62	3.112	0.49	1.436	0.16

Statistical Analysis:

Enrolled Population: Enrolled Population includes subjects who have a signed ICF.

Randomized Population: Subjects who are assigned a random number be regarded as Randomized Population.

Evaluable Immunogenicity Population: Evaluable Immunogenicity Population will consist of all eligible randomized subjects who are assigned to **lot-to-lot and** immunogenicity group, receive two vaccinations within the predefined window, have a valid immunogenicity result at visit 4 (Day 57), have no major protocol deviations or protocol deviations having impact on immunogenicity data. Evaluable Immunogenicity Population will be regarded as primary population for immunogenicity evaluation on primary and secondary immunogenicity endpoints except lot-to-lot consistency.

Evaluable Lot-to-Lot Population: A subset of Evaluable Immunogenicity but only includes subjects who are assigned to lot-to-lot consistency group and have immunogenicity determination at Day 57. Evaluable Lot-to-Lot Population will be used for evaluating lot-to-lot consistency only.

Evaluable Efficacy Population: **All eligible randomized subjects who receive two vaccinations within the predefined window and have no major protocol deviations or protocol deviations having impact on immunogenicity data will be the Evaluable Efficacy Population.** Evaluable Efficacy Population will be used to evaluate vaccine efficacy in exploratory analysis.

Available Immunogenicity Population: Available Immunogenicity Population will consist of all eligible randomized subjects who receive at least one vaccination, and have at least one post immunogenicity data determination. Available Immunogenicity Population will also be used to the immunogenicity evaluation except lot-to-lot consistency.

Evaluable Booster Population: All eligible randomized subjects who receive a third dose of UB-612 within the predefined window and have no major protocol deviations or protocol deviations having impact on immunogenicity data will be the Evaluable Booster Population. Evaluable Booster Population will be used to explore the immunogenicity evaluation after the booster vaccination.

Safety Population: The Safety Population (SAF) will consist of all subjects who received at least one vaccination. The Safety Set is for safety evaluation in analysis.

All safety assessments, including AEs, physical examinations (PEs), vital signs (VS), and clinical laboratory evaluations, where indicated, will be presented using descriptive statistics for each study group.

Local reactions for up to 7 days following each dose and systemic events for up to 7 days following each dose will be summarized with counts and percentages by study groups. Unsolicited AEs will be presented in counts and percentage with system organ class and preferred term by study groups. The number and percentage of MAAEs, SAEs, AESIs and ADEs will be displayed in summary table by study groups. Changes of safety laboratory measures will be summarized with descriptive statistics by study group and each time point.

The immunogenicity will be evaluated descriptively by seroconversion rate (SCR), GMT, GMFI and the associated 95% confidence intervals (CIs). **Immunogenicity evaluation for subjects received two vaccinations and three vaccinations will be summarized separately. Analysis of immunogenicity for subjects received a third dose of UB-612 will be performed with the Evaluable Booster Population only.**

For lot consistency, all pairs of lots, the two sided 95% CIs for the GMT ratios of SARS-COV-2 neutralizing titers will be calculated. If the two-sided 95% CIs for the GMTR of SARS-CoV-2 neutralizing titers are within the [0.5, 2.0] clinical limit interval, lot consistency will be concluded.

Interim Analysis:

First interim analysis and report will be performed when all adult immunogenicity data (350 evaluable young adults and 154 evaluable elderly subjects) for Day 57 are available

Second interim analysis and report, as EUA application dossier, will be performed when at least half of core group subjects (at least 3500 evaluable subjects) will be completed Day 85 safety follow up.

Third interim analysis and report, as EUA application dossier, will be performed when all immunogenicity data for lot-to lot consistency of Day 57 are available

Fourth interim analysis and report, as supplementary dossier for EUA, will be performed when 350 evaluable adolescents completed Day 57 safety follow up.

Fifth interim analysis will be performed for immunogenicity and safety data for young and elderly adult subjects receiving 3rd vaccination completed Visit 7, which is 14 days after 3rd vaccination.

Sixth interim analysis will be performed for immunogenicity and safety data for adolescent subjects receiving 3rd vaccination completed Visit 7, which is 14 days after 3rd vaccination.

Planned IDMC Meeting:

1st time: when all adult immunogenicity data (350 evaluable young adults and 154 evaluable elderly subjects) for Day 57 are available

2nd time: when at least half of core group subjects (at least 3500 evaluable subjects) will be completed Day 85 safety follow up. Adolescence could be enrolled after 2nd IDMC meeting.

3rd time: when all immunogenicity data for lot-to lot consistency of Day 57 are available.

4th time: when 350 evaluable adolescents will be completed Day 57 safety follow up.

The meeting schedule might be adjusted based on the progress of recruitment. The independent data monitoring committee (IDMC), consisting of at least two physicians and one statistician, will review the interim analysis results or reports.

Disease-Related Events and/or Disease-Related Outcomes Not Qualifying as AEs or SAEs:

Potential COVID-19 illnesses and their sequelae that are consistent with the disease definition should not be recorded as AEs. These data will be captured as efficacy assessment data only on the relevant pages of the CRF, as these will be expected exploratory endpoints.

Schedule of Assessments

Lot-to-lot consistency & Immunogenicity group

Scheduled visit	***1[m]***	***2[m]***		***3***		***4***		***5/ET***	***6[t]***		***7***		***Long-term follow-up***
Test & observations	***Screening***	***1st vaccination***		***2nd vaccination***		***Follow-up***		***Unblinding***	***3rd vaccination[h]***		***Follow-up[h]***		***Month 12 follow-up[i]***
Day	***-28~-1***	*1*	*8, 15, 22*	*29 ±3 days*	*36, 43*	*57 ±3 days*	*64, 71, 78, 85*	*197 ±15 days*	***197~242***	***7 days after Visit 6***	***14 days after Visit 6 ±3 days***	*253, 309*	*365 ±45 days*
Informed consent	X								X[h]				
Inclusion/Exclusion Criteria	X	X											
Randomization		X											
**Contraindication to vaccination**				X					X[h]				
Demographics	X												
Medical history	X	X											
Physical Exam[a]	X	X		X		X		X	X[h]		X[h]		
Vital sign	X	X		X		X		X	X[h]		X[h]		X
ECG	X												
**Lab (Safety)**													
Blood routine[j]	X					X			X[h]		X[h]		
Biochemistry[j]	X					X			X[h]		X[h]		
Immunology[j]	X					X			X[h]		X[h]		
Pregnancy[b]		X		X					X[h]				
Urinalysis[b]	X	X[q]		X[q]		X[q]		X[q]	X[q, h]		X[q, h]		
**Lab (Immunogenicity)**													
Immune response assessment[c]		X		X[n]		X		X					X

Scheduled visit	***1[m]***	***2[m]***		***3***		***4***		***5/ET***	***6[t]***		***7***		***Long-term follow-up***
Test & observations	***Screening***	***1st vaccination***		***2nd vaccination***		***Follow-up***		***Unblinding***	***3rd vaccination[h]***		***Follow-up[h]***		***Month 12 follow-up[i]***
Day	***-28~-1***	*1*	*8, 15, 22*	*29 ±3 days*	*36, 43*	*57 ±3 days*	*64, 71, 78, 85*	*197 ±15 days*	***197~242***	***7 days after Visit 6***	***14 days after Visit 6 ±3 days***	*253, 309*	*365 ±45 days*
T cell function (optional [o])		X				X							
**Lab (Exploratory)**													
**T cell function (optional [u])**									**X [h]**		**X [h]**		
**Spared sample [r]**	**X**					**X**							
**Immune response[s]**									**X [h]**		**X[h]**		
Vaccination		X		X					**X [h]**				
e-Diary instruction		X		X					**X [h]**				
Safety calls [d]			X		X		X			**X[s,h]**		X	
AEs/AESIs [p] /MAAEs/SAEs		X [k]	X [k]	X [k]	X [k]	X [k]	X [k]	X [l]	**X [l, h]**	**X [l, h]**	**X [l, h]**	X [l]	X [l]
COVID-19 infection surveillance		X	X	X	X	X [e]	X [e]	X [e]	**X [e, h]**	**X [e, h]**	**X [e, h]**	X [e]	X [e]
Concomitant Medication		X		X		X		X [f]	**X [f, h]**		**X [f, h]**		

a: Body weight and height will be assessed at Visit 1 only.
b: Screening for pregnancy will be performed by urine sample test on Day 1, Day 29. When positive urine pregnancy test is presented, it should be confirmed by a serum test. Serum testing in lieu of urine tests will not be considered a protocol deviation. Proteinuria will be checked via urine routine as baseline.
c: For anti-S1-RBD IgG levels, SARS-CoV-2 neutralizing titers, and antibody titers which inhibit S1-RBD: ACE2 binding.
d: All subjects will receive safety calls that will serve both to monitor for unsolicited AEs, including AESIs, and to monitor for symptoms of COVID-19.
e: Each week (i.e., every 7 days) the subject will receive a prompt on their smartphone device to regularly surveillance for signs and symptoms of COVID-19. Record medication for potential COVID-19 on COVID-19 page.
f: Only record medications for MAAE and SAE.
g: 28 days after 2nd vaccination
h: Only for subjects in vaccine group and have agreed to receive 3rd dose of UB-612.
i: 12 months (336 days) after 2nd vaccination

j: Safety laboratory includes complete blood count (Hgb, Hct, RBC), WBC with differential, platelet count, creatinine, ALT, AST, total and direct bilirubin, hs-CRP, and ANA.
k: Active collection period
l: Passive surveillance period
m: Visit 1 and 2 could be emerged as one visit.
n: Anti-S1-RBD IgG level only
o: At least 100 aged >18-< 65 subjects in selected sites will be invited
p: To include PIMMC (listed in **Table 9-6**), or any newly identified potential AESI followed through 12 months after participants' final vaccination. Complications of COVID-19, also termed as ADE, (listed in **Table 9-5**) should be considered and reported as AESIs.
q: If subject encountered ≥ Grade 3 hypertension, the existence or deterioration of proteinuria will be checked.
r: An aliquot of spared serum is to be frozen and stored for UBI SARS-CoV-2 ELISA, Confirmatory SARS-CoV-2 ELISA, and future immunologic tests.
s: Subjects who qualified to receive 3rd dose will enter 2 weeks e-diary for follow-up and a safety call post 7 days post vaccination.
t: Visit 5 and Visit 6 could be the same day.
u: Approximately thirty aged >18-< 65 subjects and approximately thirty aged ≥ 65 years subjects in selected sites will be invited. When applicable, subjects who were assessed for T cell functions on Day 57 are preferred to be invited.
ADE (Antibody Dependent Enhancement), AESIs (Adverse Event of Special Interests), ANA (Anti-Nuclear Antibody), ET (Early Termination), MAAEs (Medically Attend Adverse Events)

Safety check group

Scheduled visit	***1*** [l]	***2*** [l]		***3***		***4***		***5/ET***	***6*** [q]		***7***		***Long-term follow-up***
Test & observations	***Screening***	***1st vaccination***		***2nd vaccination***		***Follow-up*** [f]		***Unblinding***	***3rd vaccination*** [g]		***Follow-up*** [g]		***Month 12 follow-up*** [h]
Day	***-28~-1***	*1*	*8, 15, 22*	*29 ±3 days*	*36, 43*	*57 ±3 days*	*64, 71, 78, 85*	*197 ±15 days*	***197~242***	***7 days after Visit 6***	***14 days after Visit 6 ±3 days***	*253, 309*	*365 ±45 days*
Informed consent	X								**X** [g]				
Inclusion/Exclusion Criteria	X	X											
Randomization		X											
**Contraindication to vaccination**				X					**X** [g]				
Demographics	X												
Medical history	X	X											
Physical Exam [a]	X	X		X		X		X	**X** [g]		**X** [g]		
Vital sign	X	X		X		X		X	**X** [g]		**X** [g]		X
ECG	X												
**Lab (Safety)**													
Blood routine [i]	X					X			**X** [g]		**X** [g]		
Biochemistry [i]	X					X			**X** [g]		**X** [g]		
Immunology [i]	X					X			**X** [g]		**X** [g]		
Pregnancy [b]		X		X					**X** [g]				
Urinalysis [b]	X	X [n]		X [n]		X [n]		X [n]	**X** [g, n]		**X** [g, n]		
**Lab (Exploratory)**													

Scheduled visit	***1*** [l]	***2*** [l]		***3***		***4***		***5/ET***	***6*** [q]		***7***		***Long-term follow-up***
Test & observations	***Screening***	***1st vaccination***		***2nd vaccination***		***Follow-up*** [f]		***Unblinding***	***3rd vaccination*** [g]		***Follow-up*** [g]		***Month 12 follow-up*** [h]
Day	***-28~-1***	*1*	*8, 15, 22*	*29 ±3 days*	*36, 43*	*57 ±3 days*	*64, 71, 78, 85*	*197 ±15 days*	***197~242***	***7 days after Visit 6***	***14 days after Visit 6 ±3 days***	*253, 309*	*365 ±45 days*
**Spared sample** [o]	**X**					**X**							
**Immune response** [g]									**X** [g]		**X** [g]		**X** [g]
Vaccination		X		X					**X** [g]				
e-Diary instruction		X		X					**X** [g]				
Safety calls [c]			X		X		X			**X** [p, g]		X	
AEs/AESIs[m]/MAAEs/SAEs		X [j]	X [j]	X [j]	X [j]	X [j]	X [j]	X [k]	**X** [k, g]	**X** [k, g]	**X** [k, g]	**X** [k]	X [k]
COVID-19 infection surveillance		X	X	X	X	X [d]	X [d]	X [d]	**X** [d, g]	**X** [d, g]	**X** [d, g]	**X** [d]	X[d]
Concomitant Medication		X		X		X		X [e]	**X** [e, g]		**X** [e, g]		

a: Body weight and height will be assessed at Visit 1 only.
b: Screening for pregnancy and proteinuria will be performed by urine sample on Day 1, Day 29 . When positive urine pregnancy test is presented, it should be confirmed by a serum test. Serum testing in lieu of urine tests will not be considered a protocol deviation. Proteinuria will be checked via urine routine as baseline.
c: All subjects will receive safety calls that will serve both to monitor for unsolicited AEs, including AESIs, and to monitor for symptoms of COVID-19.
d: Each week (i.e., every 7 days) the subject will receive a prompt on their smartphone device to regularly surveillance for signs and symptoms of COVID-19. Record medication for potential COVID-19 on COVID-19 page.
e: Only record medications for MAAE and SAE.
f: 28 days after 2nd vaccination
g: Only for subjects in vaccine group and have agreed to receive 3rd dose of UB-612.
h: 12 months (336 days) after 2nd vaccination
i: Safety laboratory includes complete blood count (Hgb, Hct, RBC), WBC with differential, platelet count, creatinine, ALT, AST, total and direct bilirubin, hs-CRP and ANA.
j: Active collection period
k: Passive surveillance period
l: Visit 1 and 2 could be emerged as one visit.

m: To include PIMMC (listed in **Table 9-6**), or any newly identified potential AESI followed through 12 months after participants' final vaccination. Complications of COVID-19, also termed as ADE, (listed in **Table 9-5**) should be considered and reported as AESIs.

n: If subject encountered ≥ Grade 3 hypertension, the existence or deterioration of proteinuria will be checked .

o: An aliquot of spared serum is to be frozen and stored for UBI SARS-CoV-2 ELISA, Confirmatory SARS-CoV-2 ELISA, anti-S1-RBD IgG ELISA, S1-RBD:ACE2 binding inhibition ELISA, and future immunologic tests.

p: Subjects who qualified to receive 3rd dose will enter 2 weeks e dairy for follow-up and a safety call post 7 days post vaccination.

q: Visit 5 and Visit 6 could be the same day.

ADE (Antibody Dependent Enhancement), AESIs (Adverse Event of Special Interests), ANA (Anti-Nuclear Antibody), ET (Early Termination), MAAEs (Medically Attend Adverse Events)

Blood collection at scheduled visits
Lot-to-lot consistency & Immunogenicity group

Scheduled visit	***1***	***2***		***3***		***4***		***5/ET***	***6***		***7***		***Long-term follow-up***
Test & observations	***Screening***	***1st vaccination***		***2nd vaccination***		***Follow-up***		***Unblinding***	***3rd vaccination a***		***Follow-up a***		***Month 12 follow-up***
Day	***-28~-1***	*1*	*8, 15, 22*	*29 ±3 days*	*36, 43*	*57 ±3 days*	*64, 71, 78, 85*	*197 ±15 days*	***197~242***	***7 days after Visit 6***	***14 days after Visit 6 ±3 days***	*253, 309*	*365 ±45 days*
**Lab (Safety)**													
Blood routine	4.5					4.5			**4.5**		**4.5**		
Biochemistry	**3**					**3**			**3**		**3**		
Immunology	3					3			**3**		**3**		
**Lab (Immunogenicity)**													
Immune response assessment		20		5		20		20					20
**Lab (Exploratory)**													
**Spared sample**	**5**					**5**							
**Immune response***									**10[b]**		**10**		
Total blood collection	**15.5**	20		5		**35.5**		20	**20.5**		**20.5**		20

Total amount of blood collection: **157 mL.**

* An additional 56 mL of blood sample will be collected for the optional T cell assessments at **Visit 2, Visit 4, Visit 6 and Visit 7.**

a: Only for subjects in vaccine group and have agreed to receive 3rd dose of UB-612. An additional 10 mL of blood sample will be collected at Visit 6 and 14 days after Visit 6.

b: If Visit 5 and Visit 6 are the same visit, the 10mL blood sample for exploratory immune response will be not collected.

<u>Safety check group</u>

Scheduled visit	***1***	***2***		***3***		***4***		***5/ET***	***6***		***7***		***Long-term follow-up***
Test & observations	***Screening***	***1st vaccination***		***2nd vaccination***		***Follow-up***		***Unblinding***	***3rd vaccination[a]***		***Follow-up[a]***		***Month 12 follow-up[a]***
Day	***-28~-1***	*1*	*8, 15, 22*	*29 ±3 days*	*36, 43*	*57 ±3 days*	*64, 71, 78, 85*	*197 ±15 days*	***197~242***	***7 days after Visit 6***	***14 days after Visit 6 ±3 days***	*253, 309*	*365 ±45 days*
**Lab (Safety)**													
Blood routine	4.5					4.5			**4.5**		**4.5**		
Biochemistry	**3**					**3**			**3**		**3**		
Immunology	3					3			**3**		**3**		
**Lab (Exploratory)**													
**Spared sample**	**5**					**5**							
**Immune response**									**10**		**10**		**10**
Total blood collection	**15.5**					**15.5**			**20.5**		**20.5**		**10**

Total amount of blood collection: **82 mL.**

a: Only for subjects in vaccine group and have agreed to receive 3rd dose of UB-612. An additional 10 mL of blood sample will be collected at Visit 6, 14 days after Visit 6, and Day 365.

3 TABLE OF CONTENTS

1 Protocol Approval Signatures 2

2 Synopsis 3

3 Table of Contents 18

4 List of Abbreviations 22

5 Introduction 24

5.1 Study Rationale 24
5.2 Background 24
5.3 UB-612 COVID-19 Vaccine 25
5.4 Phase I study summary (V-122 and V-123) 25
5.5 Risk/Benefit Assessment 27

6 Study Objectives & Endpoints 29

6.1 Primary Objective(s) 29
6.2 Primary Endpoint(s) 29
6.3 Secondary Objective(s) 29
6.4 Secondary Endpoint(s) 29
6.5 Exploratory Objective(s) 29
6.6 Exploratory endpoint(s) 30

7 Investigational Plan 31

7.1 Overall Study Design and Plan: Description 31
7.1.1 End of Study Definition 32
7.2 Schedule of Assessments 33
7.3 Discussion of Study Design 42
7.4 Selection of Study Population 42
7.4.1 Number of Planned Subjects 42
7.4.2 Inclusion Criteria 43
7.4.3 Exclusion Criteria 43
7.4.4 Contraindications to Vaccination 45
7.4.4.1 Criteria for Delay of Study Treatment 45
7.4.4.2 Criteria for Discontinuation of Study Treatment 45
7.4.5 Screen Failures 45
7.4.6 Method of Assigning Subjects to Treatment Groups 46

7.4.7 Blinding 46
7.4.8 Removal of Subjects 47
7.4.8.1 Removal of Subjects from Immunogenicity Analysis 47
7.4.8.2 Removal of Subjects from the Study 47
7.4.8.3 Study Termination 47
7.4.8.4 Reporting and Follow-up of Pregnancies 48
7.4.8.5 SARS-CoV-2 infection 48

7.5 Investigational Products 48

7.5.1 Identity of Investigational Products 48
7.5.2 Investigational Products Administered 49
7.5.2.1 Injection Volume(s) 49
7.5.2.2 Administration 49
7.5.2.3 Emergency Event Management 49
7.5.3 Packaging and Labeling 49
7.5.4 Prior and Concomitant Therapy 49
7.5.4.1 Prohibited Medication/Therapy 50

8 Timing of Study Procedures 51

8.1 Study Procedures 51

8.1.1 Visit 1 (Day -28 ± 3 days) –Screening 51
8.1.2 Visit 2 (Day 1) –Baseline: 1st Vaccination 51
8.1.3 Day 8, 15, and 22 –Safety calls 52
8.1.4 Visit 3 (Day 29 ± 3 day) –2nd Vaccination 52
8.1.5 Day 36 and 43 –Safety calls 53
8.1.6 Visit 4 (Day 57 ± 3 day) –Follow-up 53
8.1.7 Day 64, Day 71, Day 78, and Day 85 –Safety calls 53
8.1.8 Visit 5 (Day 197 ± 15 days) –Unblinding / Early Termination 53
8.1.9 Visit 6 (Day 197~242) –3rd Vaccination 54
8.1.10 Safety call on 7 days after Visit 6 (Only for subjects who have 3rd vaccination) 55
8.1.11 Follow up on 14 days after Visit 6 (± 3 day) (Only for subjects who have 3rd vaccination) 55
8.1.12 Day 253 and Day 309- Bi-monthly Safety calls 55
8.1.13 Long-term follow-up (Day 365 ± 45 days) 55

8.2 Early Termination (ET) 56

8.3 Unscheduled Visit 56

8.4 COVID-19 Surveillance (All Subjects) 56

8.4.1 Taiwan CDC Criteria for Case Definition of COVID-19 56
8.4.2 Intercurrent COVID-19 57
8.4.3 Potential COVID-19 Infection 59
8.4.4 COVID-19 Convalescent Visit 59

8.5 Duration of Treatment 59

9 Immunogenicity and Safety Variables ... 60

9.1 Informed Consent Form ... 60

9.2 Demographics / Other Baseline Characteristics ... 60

9.3 Eligibility ... 60

9.4 Administration ... 60

9.5 Safety and Immunogenicity Measurements Assessed ... 60

9.5.1 Safety Variables ... 60
9.5.1.1 Physical Examination ... 60
9.5.1.2 Vital Sign ... 61
9.5.2 Electrocardiogram(ECG) ... 61
9.5.3 Clinical Laboratory Evaluation ... 61
9.5.4 Pregnancy Test ... 62
9.5.5 Self-Evaluation/Reporting (Solicited/Unsolicited Symptoms) ... 62
9.5.5.1 Solicited Symptoms ... 62
9.5.5.2 Unsolicited Adverse Event ... 64
9.5.5.3 Time Period and Frequency for Collecting AE and SAE Information ... 64
9.5.6 Adverse events (AEs) ... 65
9.5.6.1 Definition of Adverse Events (AE) ... 65
9.5.6.2 Definition of Serious Adverse Events (SAE) ... 65
9.5.6.3 Definition of Unexpected Adverse Reaction Definition ... 66
9.5.6.4 Definition of Medically Attended Adverse Event ... 66
9.5.6.5 Definition of Adverse Events of Special Interest (AESI) ... 67
9.5.6.6 Assessment of Severity ... 69
9.5.6.7 The Relationship to Study Vaccine ... 69
9.5.6.8 Adverse Events Reporting ... 71
9.5.7 Immunogenicity Assessments ... 72
9.5.7.1 Surveillance and Laboratory Diagnosis of SARS-CoV-2 Infection during Clinical Trial ... 72
9.5.7.2 Detection of ELISA Antibodies against S1-RBD of SARS-CoV-2 ... 73
9.5.7.3 Detection of Antibody Titers Which Inhibit S1-RBD:ACE2 binding ... 73
9.5.7.4 Neutralizing Antibody Titers against SARS-CoV-2 ... 73
9.5.7.5 Detection of antibody against SARS-CoV-2 antigens ... 73
9.5.7.6 Detection of T Cell Response ... 73
9.5.8 Communication and Use of Technology ... 74
9.5.9 Appropriateness of Measurements ... 74

9.6 Independent Data Monitoring Committee (IDMC) ... 74

9.6.1 Overall Study Stopping Rules ... 75

10 Statistical Methods ... 77

10.1 Statistical and Analytical Plans ... 77

10.2 Sample Size Determination ... 77

10.2.1 Sample Size for Safety Evaluation 77
10.2.2 Sample Size for Lot-to-Lot Consistency 77

10.3 Analysis Populations 78

10.4 Demographic and Other Baseline Characteristics 79

10.5 Safety Evaluation 79

10.5.1 Analysis of Primary Safety Endpoint(s) 80
10.5.2 Analysis of Secondary Safety Endpoint 80

10.6 Immunogenicity Evaluation 81

10.6.1 Analysis of Primary Immunogenicity Endpoint(s) 81
10.6.2 Analysis of Secondary Immunogenicity Endpoint(s) 81

10.7 Analysis of Exploratory Endpoint(s) 82

10.8 Subgroup Analysis 85

10.9 Interim Analysis 85

10.10 Handling of Missing Data 86

10.11 Protocol Deviations 86

11 Quality Assurance and Quality Control 87

11.1 Audit and Inspection 87

11.2 Monitoring 87

11.3 Data Management and Coding 87

12 Records and Supplies 88

12.1 Drug Accountability 88

12.2 Financing and Insurance 88

13 Ethics 89

13.1 Independent Ethics Committee or Institutional Review Board 89

13.2 Regulatory Authorities 89

13.3 Ethical Conduct of the Study 89

13.4 Informed Consent 89

13.5 Subject Confidentiality 90

14 Reporting and Publication, Including Archiving 91

15 References 92

4 LIST OF ABBREVIATIONS

ACE2	Angiotensin-converting enzyme 2
ADE	Antibody dependent enhancement (of viral replication)
AE	adverse event
AESI	adverse event of special interest
AEFI	adverse events following immunization
ALP	alkaline phosphatase
ALT	alanine aminotransferase
ANA	Anti-nuclear antibody
ANCA	anti-neutrophil cytoplasmic antibody
AST	aspartate aminotransferase
β-HCG	beta-human chorionic gonadotropin
BP	blood pressure
BUN	blood urea nitrogen
CBC	complete blood count
cGCP	current good clinical practice
CHO	Chinese hamster ovary
CRF	case report form
CT	computed tomography
ECG	Electrocardiogram
EUA	Emergency Use Authorization
FDA	Food and Drug Administration
**GMFI**	**geometric mean fold increase**
**GMT**	**geometric mean titer**
HBV	hepatitis B virus
HCT	hematocrit
HCV	hepatitis C virus
HIV	human immunodeficiency virus
HR	heart rate
hs-CRP	high sensitivity (hs) CRP
ICF	informed consent form
ICH	International Council for Harmonisation
ICTV	International Committee on Taxonomy of Viruses
IEC	independent ethics committee
IDMC	Independent Data Monitoring Committee
IgA	immunoglobulin A
IgG	immunoglobulin G
IM	intramuscular
IRB	institutional review board
N	number of subjects in the dataset or population
N/A	not applicable
ORF	open reading frame
PE	physical examination
**PIMMC**	**potential immune-mediated medical conditions**
RBD	receptor binding domain
RR	respiratory rate
RT-PCR	reverse transcriptase- polymerase chain reaction
SCR	seroconversion rate

SAE	serious adverse event
SUSAR	suspected unexpected serious adverse reaction
TB	tuberculosis
UB-612	United Biomedical, Inc.'s COVID-19 vaccine
VAERD	vaccine-associated enhanced respiratory disease
VS	vital signs
WBC	white blood cell
WoCBP	woman/women of childbearing potential

5 INTRODUCTION

5.1 Study Rationale

The global COVID-19 pandemic caused by the SARS-CoV-2 virus has made the development of an effective vaccine a top biomedical priority. Antibodies are essential elements of most vaccines and will likely be crucial component of an effective vaccine against SARS-CoV-2. Though plasma neutralizing activity is low in most convalescent individuals, the recurrent anti-SARS-CoV-2 RBD antibodies (the most immunogenic fragment within the SARS-CoV-2 Spike protein) with potent neutralizing activity can be found in individuals with unexceptional plasma neutralizing activity, suggesting that humans are intrinsically capable of generating anti-RBD antibodies that potently neutralize SARS-CoV-2. In addition, substantial activation of $CD4^+$ and $CD8^+$ T cells are required to prevent further infection and to help the clearance of virus after exposure. Thus, vaccines that efficiently induce neutralizing antibodies targeting the SARS-CoV-2 RBD and trigger SARS-CoV-2-specific cellular responses are anticipated to induce an optimal immunogenicity profile and achieve the prevention purpose.

5.2 Background

In December 2019, a cluster of patients with pneumonia surfaced in Wuhan, China. The culprit was quickly identified as a beta-coronavirus that has never been reported before, and the disease was named by WHO as <u>C</u>orona <u>Vi</u>rus <u>D</u>isease 20<u>19</u> (COVID-19) and the virus that causes it by the International Committee on Taxonomy of Viruses (ICTV) as SARS-CoV-2 [1, 2]. As of January 7, 2020, a global outbreak has caused 87,200,000 confirmed cases in more than 220 countries or territories, with 1,880,000 deaths, making the SARS-CoV-2 pandemic a general public health event that has stirred up worldwide attention. Currently, the epidemic is still spreading and there is no effective means to prevent the infection.

SARS-CoV-2 is a positive-strand RNA virus that belongs to the group of *Betacoronaviruses*. The genome of SARS-CoV-2 is approximately 29,700 nucleotides long and shares 79.5% sequence identity with SARS-CoV [3]. The long ORF1ab polyprotein at 5' end of the genome encodes 15 or 16 non-structural proteins, and the 3' end encodes 4 major structural proteins, including the spike (S) protein, nucleocapsid (N) protein, membrane (M) protein, and the envelope (E) protein [4]. SARS-CoV-2 interacts with the receptor angiotensin converting enzyme 2 (ACE2) on host cells via receptor binding domain (RBD) of its S protein for viral entry and subsequent pathogenesis [5], resulting in severe respiratory illness with symptoms of fever, cough, and shortness of breath, and even death in severe cases [6].

Vaccines are the most effective and economical means to prevent and control infectious diseases [7]. The development of an effective vaccine against SARS-Co-2 infection is urgently required. Currently, more than 200 pharmaceutical companies and academic institutions worldwide have launched their programs on vaccine development against SARS-CoV-2. There are several different types of vaccines under development; one of them is subunit vaccine. Subunit vaccines include one or more antigens with strong immunogenicity capable of efficiently stimulating the host immune system. In general, this type of vaccine is safer and easier to produce, but often requires the addition of adjuvants to elicit a strong protective immune response. So far, several institutions have initiated programs on the SARS-CoV-2 subunit vaccine, and almost all of them use the S protein as antigens. For example, the University of Queensland is developing a subunit vaccine based on the "molecular clamp" technology [8]. Clover Biopharmaceuticals Inc. revealed that they

are developing a vaccine candidate against SARS-CoV-2 using the “Trimer-Tag” technology [9], and the trimeric S protein subunit vaccine candidate was produced via a mammalian cell expression system. Novavax, Inc. announced that they had produced multiple nanoparticle vaccine candidates based on S protein, and after assessing efficacy in animal models to identify an optimal vaccine candidate, began Phase I clinical testing in May, 2020. Besides, Johnson & Johnson, Pasteur Institute, Sanofi Pasteru, GSK, and Chongqing Zhifei Biological Products Co., Ltd. also started subunit vaccine development against SARS-CoV-2.

Safety is the most important issue that should be taken into consideration during drug and vaccine development, and some scientists urge that we should not rush to deploy COVID-19 vaccines and drugs without sufficient safety guarantees [10]. There have been concerns regarding vaccine-associated enhanced respiratory disease (VAERD) by certain candidate COVID-19 vaccine approaches, via antibody-dependent enhancement (ADE) or development of Th2 immunopathology [11]. Grifoni *et al.* [12] revealed predominant Th1 responses in convalescing COVID-19 cases, with little to no Th2 cytokines. Clearly more studies are required, but the data Grifoni *et al.* shown appear to predominantly represent a classic Th1 response to SARS-CoV-2.

5.3 UB-612 COVID-19 Vaccine

United Biomedical, Inc. (UBI) has developed a vaccine candidate against SARS-CoV-2 that is designed to activate both humoral and cellular responses. For SARS-CoV-2 immunogens, UB-612 includes a designer S1-RBD-sFc (SRsFc) fusion protein formulated with designer Th and CTL epitope peptides selected from immunodominant M, S2 and N regions known to bind to human MHC I and II. This mixture of designer Th/CTL peptides is designed to elicit T cell activation, memory recall and effector functions similar to that of natural COVID-19. The S1-RBD-sFc fusion protein incorporates both linear and conformation epitopes and induces high affinity antibodies to the RBD of SARS-CoV-2. The immunogen components are formulated with an oligonucleotide containing unmethylated CpG motifs and Adju-Phos® adjuvants, which promotes the activation of antigen-presenting cells pathways to induce an optimal immunogenicity profile and achieve the prevention purpose.

In summary, UB-612 vaccine design composition (S1-RBD-sFc+Th and CTL peptides + CpG, formulated with Adju-Phos®) is expected not only to be safe and induce high titers of neutralizing antibodies, but also to provide T cell memory for a long lasting protection against COVID-19 across all human subjects irrespective of age, sex and ethnicities.

5.4 Phase I study summary (V-122 and V-123)

The First-in-Human (FIH) phase I study began dosing on 25 September 2020 (clinicaltrials.gov**: NCT04545749, protocol number: V-122)** and is ongoing. This is an open-label, dose-escalation clinical study to evaluate the safety, tolerability and immunogenicity of 3 ascending doses of UB-612 COVID-19 vaccine in healthy adults between 20 and 55 years of age. This study **was** carried out in three groups, including A group (receiving 2 doses of UB-612 vaccine 10 µg), B group (receiving 2 doses of UB-612 vaccine 30 µg), and C group (receiving 2 doses of UB-612 vaccine 100 µg). A total of 60 subjects **were** enrolled into the study (20 subjects per groups). Safety monitoring reviews **were** held to decide if the study can go on to second immunization for each cohort or to the higher dose regimens. In the co-primary endpoints, occurrence of adverse reaction within 7 days after vaccination, and the percentage of subjects with ≥ Grade 3 adverse events within 7 days after vaccination **were** calculated. In the secondary endpoints, the immunogenicity

of the UB-612 vaccine **was** assessed, including GMT, SCR, and geometric mean fold increase of antigen-specific antibody (Anti-S1-RBD). In the exploratory endpoints, GMT, SCR, and geometric mean fold increase of neutralizing antibody, the correlation between immune response detected by ELISA and live virus neutralization test, and T cell responses **were** evaluated.

A total number of 63 subjects were screened, in which 60 subjects were enrolled equally disturbed in three dose groups, that is 20 subjects receiving UB-612 vaccine 10 μg (A group), 20 subjects receiving UB-612 vaccine 30 μg (B Group), and 20 subjects receiving UB-612 vaccine 100 μg (C Group). This study is still ongoing, and no one withdrew from the study.

In Group A, 13 subjects (65.0%) in the subjects receiving UB-612 vaccine 10 μg had any adverse reactions within 7 days after first vaccination. No any ≥ Grade 3 adverse reaction in UB-612 vaccine 10 μg group within 7 days after first vaccination was reported. For any adverse reactions within 7 days after second vaccination were reported in 11 subjects (55.0%) in UB-612 vaccine 10 μg group. There was no any ≥ grade 3 adverse reactions within 7 days after second vaccination in UB-612 vaccine 10 μg group.

In Group A, all subjects in UB-612 vaccine 10 μg group revealed no detection in antigen-specific antibody before vaccination. The GMT of antigen-specific antibody increased to 73.56(95% CI: 33.458~161.720) at Visit 6 (Day 28), 350.83 (95% CI: 161.630~761.501) at Visit 8 (Day 42) and continued to increase to 526.98 (95% CI: 269.663~1029.839) at Visit 9 (Day 56). At Day 56 after second vaccination, nineteen participants showed a 4-fold change in antigen-specific antibody titer from baseline. The GMT of neutralizing antibody increased to 7.73 (95% CI: 4.704~12.694) at Visit 6 (Day 28), 44.45 (95% CI: 23.017~85.821) at Visit 8 (Day 42), and 41.92 (95% CI: 24.020~73.146) at Visit 9 (Day 56). SCR of neutralizing antibody was observed in 18 (90.0%; 95% CI: 68.30%~98.77%) participants at Visit 9 (Day 56).

In Group B receiving UB-612 vaccine 30 μg, 14 (70.0%) had any adverse reactions within 7 days after first vaccination. No any ≥ Grade 3 adverse reaction within 7 days after first vaccination was found. For any adverse reactions within 7 days after second vaccination were reported in 12 (60.0%). There was no any ≥ grade 3 adverse reactions within 7 days after second vaccination.

All subjects in UB-612 vaccine 30 μg group reported no detection in antigen-specific antibody before vaccination. The GMT of antigen-specific antibody were to 42.75 (95% CI: 21.412 ~ 85.358) at Visit 6 (Day 28), increased to 366.80 (95% CI: 187.085 ~ 719.139) at Visit 8 (Day 42) but decrease to 269.06 (95% CI: 157.931 ~ 458.381) at Visit 9 (Day 56). At Day 56 after second vaccination, 19 (95.0%) participants showed a 4-fold change in antigen-specific antibody titer from baseline. The GMT of neutralizing antibody increased to 4.97 (95% CI: 3.259~7.594) at Visit 6 (Day 28), 31.29 (95% CI: 17.159, 57.060) at Visit 8 (Day 42), and 28.99 (95% CI: 19.101, 43.991) at Visit 9 (Day 56). SCR of neutralizing antibody was observed in 16 (80.0%; 95% CI: 56.34 %~ 94.27 %) participants at Visit 9 (Day 56).

In Group C receiving UB-612 vaccine 100 μg, 16 (80.0%) had any adverse reactions within 7 days after first vaccination. No any ≥ Grade 3 adverse reaction within 7 days after first vaccination was found. For any adverse reactions within 7 days after second vaccination were reported in 7 (35.0%). There was no any ≥ grade 3 adverse reactions within 7 days after second vaccination. All subjects reported no detection in antigen-specific antibody before vaccination. The GMT of antigen-specific antibody were to 103.20 (95% CI: 48.132 ~ 221.265) at Visit 6 (Day 28), increased to 2240.15 (95% CI: 1233.797 ~ 4067.353) at Visit 8 (Day 42). In additional, The GMT of antigen-specific antibody at Visit 9 (Day 56) in first 6 subjects was 897.44 (95% CI: 613.808 ~ 1312.121).

At Day 42 after second vaccination, 20 (100%) participants showed a 4-fold change in antigen-specific antibody titer from baseline. At Day 56 after second vaccination, first 6 participants all showed a 4-fold change in antigen-specific antibody titer from baseline. The GMT of neutralizing antibody increased to 10.84 (95% CI: 5.859~20.048) at Visit 6 (Day 28) and 107.66 (95% CI:72.402 ~160.087) at Visit 8 (Day 42). At Day 56, GMT of neutralizing antibody first 6 participants was 88.79 (95% CI: 51.828, 152.108). SCR of neutralizing antibody were 100% (95% CI: 83.16% ~ 100.00%) observed at Visit 8 (Day 42). At Day 56, SCR of neutralizing antibody was 100% (95% CI: 54.07% ~ 100%) in first 6 participants at Visit 9 (Day 56).

V-123 is an extension study (clinicaltrials.gov: NCT04967742) to evaluate the safety, tolerability and immunogenicity of one booster dose of UB-612 COVID-19 vaccine in adults who completed two vaccinations of UB-612 vaccine at 10, 30, and 100 µg in V-122 study.

The subjects who completed two vaccinations in Phase I study will be recruited in the extension study. After the inform consent is obtained from the subject, eligible subjects will receive one booster dose of UB-612 vaccines 100 µg with the same dosing which was offered in Phase II study, at least 6 months after the second vaccination.

Preliminary data showed extremely high immune response (Figure 1) after 14 days post 3rd vaccination. No SAE had been reported since then. The incidence of local reactions increased, but no grade 3/4 AE recorded.

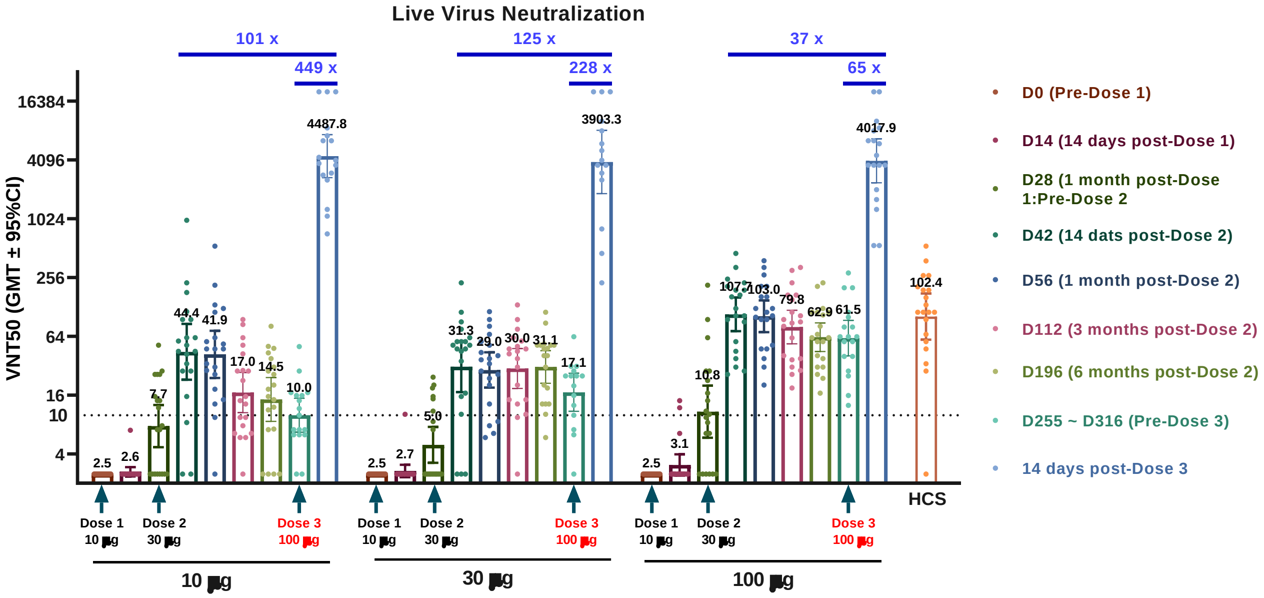

Figure 1. Neutralizing antibody titers after 3rd vaccination measured by wild-type live virus assay

5.5 Risk/Benefit Assessment

Nonclinical studies of UB-612 antigens have shown that IM delivery of the vaccine is (1) safe and well-tolerated in tested animals and (2) effective in inducing potent anti-S1-RBD antibody responses. Antibodies elicited by UB-612 antigens exhibit high neutralizing activity, which blocks

the protein-protein interaction between ACE2 and S1-RBD and prevent SARS-CoV-2-induced *in vitro* cytopathology. Regarding to the ongoing phase 1 study, based on DSMB judgement, there was no safety concern in UB-612 at 10 µg, 30 µg and 100 µg.

Based on the current report, administration 100 µg of UB-612 vaccine was safe and tolerable. The first 6 subjects' immune responses in neutralizing antibody titer at Day 42 could achieve 107, close to the human convalescent serum. We aim to further evaluate the immunogenicity, tolerability and safety of 100 µg UB-612 in adults and adolescents in phase II study.

Since no Grade 3/4 solicited AE and SAE reported from V-123, administration of 3rd dose of 100 µg of UB-612 vaccine is expected to be safe and tolerable. And based on immune response from V-123, the booster effect of 3rd vaccination on Visit 6 (Day 197-242) could also be expected.

6 STUDY OBJECTIVES & ENDPOINTS

6.1 Primary Objective(s)

- To evaluate the SARS-CoV-2 neutralizing antibody titer induced by UB-612 vaccine
- To evaluate the safety and tolerability of the UB-612 vaccine after vaccination

6.2 Primary Endpoint(s)

Immunogenicity Endpoint(s)

- Geometric mean titer (GMT) of SARS-CoV-2 neutralizing antibody on Day 57
- Seroconversion rate (SCR) of SARS-CoV-2 neutralizing antibody on Day 57

Safety Endpoint(s)

- Local reactions for up to 7 days following each dose
- Systemic events for up to 7 days following each dose
- Unsolicited AEs from Day 1 to Day 57
- MAAEs and SAEs from Day 1 to Day **365**
- AESIs and ADEs from Day 1 to Day **365**

6.3 Secondary Objective(s)

- To evaluate the immune response to SARS-CoV-2 during the study
- To evaluate the lot consistency of immune responses induced by 3 independent batches of vaccine

6.4 Secondary Endpoint(s)

Immunogenicity Endpoint(s)

- Seroconversion rate (SCR) of antigen-specific antibody (Anti-S1-RBD) on Day 57
- Geometric mean titer (GMT) of SARS-CoV-2 neutralizing antibody on Day 197 and 365
- Geometric mean titer (GMT) of antigen-specific antibody (Anti-S1-RBD) on Day 57, 197 and 365
- Geometric mean fold increase in **SARS-CoV-2 neutralizing antibody and** antigen-specific antibody (Anti-S1-RBD) on Day57, 197 and 365
- Lot consistency as assessed by the comparisons of the GMT of SARS-CoV-2 neutralizing antibody on Day 57 induced by 3 independent UB-612 vaccine clinical materials. The 95% confidence intervals between groups will be within the margin of 0.5 to 2.

Safety Endpoint(s)

- Changes of safety laboratory measures

6.5 Exploratory Objective(s)

- To evaluate the T cell function induced by **UB-612** vaccine
- To evaluate the safety and immunogenicity of the UB-612 vaccine in adolescents

- To evaluate the efficacy of UB-612 vaccine
- To describe the serological responses to the UB-612 vaccine in confirmed and/or severe COVID-19 cases
- **To evaluate antibody against SARS-CoV-2 antigens**
-

6.6 Exploratory endpoint(s)

- T cell responses to UB-612 measured by ELISpot and flow cytometric assays on Day 57
- **T cell responses to UB-612 measured by ELISpot and flow cytometric assays on 14 days post 3rd dose of UB-612**
- **Geometric mean titer (GMT) of SARS-CoV-2 neutralizing antibody on 14 days post 3rd dose of UB-612**
- **Geometric mean titer (GMT) of antigen-specific antibody (Anti-S1-RBD) 14 days post 3rd dose of UB-612**
- **Geometric mean fold increase in SARS-CoV-2 neutralizing antibody and antigen-specific antibody (Anti-S1-RBD) on14 days post 3rd dose against pre-3rd dose baseline**
- **Geometric mean titer (GMT) of SARS-CoV-2 neutralizing antibody and antigen-specific antibody (Anti-S1-RBD) on Day 57, Day 197 and Day 365 in adolescents**
- **Seroconversion rate (SCR) of SARS-CoV-2 neutralizing antibody and antigen-specific antibody (Anti-S1-RBD) on Day 57 in adolescents**
- **Geometric mean fold increase in SARS-CoV-2 neutralizing antibody and antigen-specific antibody (Anti-S1-RBD) on Day 57, Day 197, and Day 365 in adolescents**
- Local reactions for up to 7 days following each dose in adolescents
- Systemic events for up to 7 days following each dose in adolescents
- Unsolicited AEs from Day 1 to Day 57 in adolescents
- MAAEs and SAEs from Day 1 to Day **365** in adolescents
- AESIs and ADEs from Day 1 to Day **365** in adolescents
- Changes of safety laboratory measures in adolescents
- COVID-19 incidence per 1000 person-years of follow-up based on PCR test
- To describe the anti-S1-RBD IgG levels and SARS-CoV-2 neutralizing titers to UB-612 in confirmed and/or severe COVID-19 cases
- **To detect antibody against SARS-CoV-2 antigens derived from S2, N, and M protein**

7 INVESTIGATIONAL PLAN

7.1 Overall Study Design and Plan: Description

This is a phase II, observer-blind, multiple-centre, randomized, placebo-controlled study to evaluate the immunogenicity, safety, tolerability and lot consistency of 2 doses of UB-612 vaccine in adolescent, younger and elderly adults. Around 3850 adult subjects will be randomized to be composed of the core group for EUA application, while around 385 adolescents will be randomized to be the supplementary group for broader indication. All subjects will be randomly allocated to receive 2 doses of 100 µg vaccine or placebo in a 6:1 ratio, including 462 aged from >18 to < 65 years old, evaluable subjects in the lot-to-lot consistency group. As for immunogenicity, at least 350 evaluable young **adults** (aged >18 to < 65 years old) and 154 evaluable elderly (aged ≥ 65 years old) will be enrolled for descriptive analysis. Subjects in immunogenicity group should be enrolled first. All subjects will be included in the safety group, and it is intended that a minimum of 770 subjects will be randomized to be in the ≥ 65-year stratum. Adolescents will start to enrol after recruitment of the core group has been completed. Around 385 adolescents will be randomized to be allocated in 6:1 ratio, in which 154 evaluable adolescents will have immunogenicity data to compare with adults.

It will be consisted of 7 clinical visits and one long-term follow-up visit. All subjects will have blood test for safety before and after full vaccination. Subjects in lot-to-lot consistency and immunogenicity group will also have blood drawn for immune response, in which tests for T cell function will be optional. Subjects will come to the clinics at Visit 1 for screening, Visit 2 (Day 1, baseline) for randomization and 1st vaccination, Visit 3 (Day 29) for 2nd vaccination, Visit 4 (Day 57) for safety check and immunogenicity assessment, and Visit 5 (Day 197) for safety check and assessment of the persistence of immune response. **Subjects will also be unblinded at Visit 5. Subjects in placebo group will withdraw from the study and subjects in vaccine group will be encouraged to have 3rd dose of vaccination at Visit 6 (Day 197~Day 242). Those who received 3rd dose will have Visit 7 (14 days after Visit 6) to check the booster effect. After Day 197, subjects will enter the long-term follow-up with a safety call bi-monthly.** Subjects in lot-to-lot and immunogenicity group will be encouraged to visit site at Day 365 to check the immune persistence. Thus, subjects will be expected to participate for up to a maximum of approximately 13 months. Unblinding will be carried out when **subjects** in core group or supplementary group have completed Visit 5. For females of child-bearing potential, a urine pregnancy test will be performed before **each** vaccination and will be found negative. Female subjects or the female partners of male subjects who are pregnant during the study period will be followed the pregnancy outcomes.

Before any study-related procedures are performed, voluntary, written, study-specific informed consent will be obtained from the subjects. **Subjects will be screened at Visit 1.** All subjects will have blood sampling for hematology and biochemistry. After checking the eligibility of the subject at Visit 2, the randomization number and adjoining study intervention allocation will be assigned. **Subjects in lot-to-lot consistency and immunogenicity group will have blood drawn for immunogenicity before vaccination.** Unblinded site staff member(s) will dispense/administer 0.5 mL UB-612/placebo into the deltoid muscle of the preferably nondominant arm. Blinded site staff must observe the subject for at least 30 minutes after study vaccine administration for changes in vital signs or any acute anaphylactic reactions. The investigator will provide instructions for reactogenicity e-diary completion and ask the subject to complete the reactogenicity e-diary from

Day 1 to Day 7, skin allergic reaction e-diary from Day 1 to Day 14, with Day 1 being the day of vaccination. Participants will be instructed to contact the site staff or investigator immediately if he or she experiences any of the Grade 3/4 AEs from Day 1 to Day 7 to determine if an unscheduled reactogenicity visit is required. Apart from reactogenicity, allergic skin rash should be monitored for 14 days post vaccination. **Once subject encountered the Grade 3/4 allergic skin rash, site staff should call for detail to evaluate the necessarily of unscheduled visit. Investigators could ask for HLA typing survey if he/she concerned about the possibility of hypersensitivity. Of course, IDMC could also request HLA typing once they reviewed the safety data.**

At Visit 3 (Day 29), the 2nd study vaccine administration will be done and subjects will be closely monitored for 30 minutes after vaccination for changes in vital signs or acute anaphylactic reaction. The 7/14-day post-vaccination e-diary will be given to subjects with a suitable instruction. For females of child-bearing potential, a urine pregnancy test will be performed before vaccination and found negative. At Visit 4 (Day 57, 1-month follow-up visit after 2nd vaccination), collect sample for safety and immunogenicity, review the participants' reactogenicity e-diary data and record any unsolicited AE.

All subjects will come back on Day 197, Visit 5, for immunogenicity persistence check in immunogenicity group and safety check for safety group. **Once subject completes Visit 5, he or she will be unblinded. Subjects received placebo will withdraw from the study and subjects who received UB-612 vaccine will be invited to join the extension study to determine the durability of the immune response and long-term safety after unblinding. Meanwhile, subjects in vaccine group will be encouraged to have a third dose of UB-612.All subjects who received UB-612 vaccine will be followed up for 12 months.**

All subjects will receive safety calls on Day 8, Day 15, Day 22, Day 36, Day 43, Day 64, Day 71, Day 78, Day 85, Day 253, and Day 309 that will serve both to monitor for unsolicited AEs or/and to monitor for symptoms of COVID-19. After Day 57, each week (i.e., every 7 days) the subject will receive a prompt on their smartphone device to regularly surveillance for signs and symptoms of COVID-19 and SAE. **Subjects who received 3rd dose of UB-612 will receive an extra safety call on 7 days post 3rd vaccination.**

If a Grade 3 local reaction, systemic event, or fever are reported in the reactogenicity e-diary, a telephone contact should occur to ascertain further details and determine whether a site visit is clinically indicated. If suspected Grade 4 local reaction systemic event and /or fever is reported in the reactogenicity e-diary, a site visit should occur to confirm whether the event meets the criteria for Grade 4. All subjects were encouraged to contact site staff once they encountered Grade 3/4 AE. Allergic skin reaction should be monitored for 14 days post vaccination and should be contacted regardless of severity.

If a subject experiences a febrile illness associated with respiratory symptoms, he or she is instructed to contact the site immediately. Subjects may utilize a COVID-19 illness e-diary to prompt him/her to report any symptoms.

7.1.1 End of Study Definition

The end of study is defined as the date of last visit of the last participant in the study. A subject is supposed to have completed the study if he/she has completed all data required for this study.

7.2 Schedule of Assessments

Below is a list of all study procedures through the study period and the signs “X” indicate when the procedures are performed.

Lot-to-lot consistency & Immunogenicity group

Scheduled visit	***1[m]***	***2[m]***		***3***		***4***		***5/ET***	***6[t]***		***7***		***Long-term follow-up***
Test & observations	***Screening***	***1st vaccination***		***2nd vaccination***		***Follow-up***		***Unblinding***	***3rd vaccination[h]***		***Follow-up[h]***		***Month 12 follow-up[i]***
Day	*-28~-1*	*1*	*8, 15, 22*	*29 ±3 days*	*36, 43*	*57 ±3 days*	*64, 71, 78, 85*	*197 ±15 days*	*197~242*	*7 days after Visit 6*	*14 days after Visit 6 ±3 days*	*253, 309*	*365 ±45 days*
Informed consent	X								X[h]				
Inclusion/Exclusion Criteria	X	X											
Randomization		X											
**Contraindication to vaccination**				X					X[h]				
Demographics	X												
Medical history	X	X											
Physical Exam[a]	X	X		X		X		X	X[h]		X[h]		
Vital sign	X	X		X		X		X	X[h]		X[h]		X
ECG	X												
**Lab (Safety)**													
Blood routine[j]	X					X			X[h]		X[h]		
Biochemistry[j]	X					X			X[h]		X[h]		
Immunology[j]	X					X			X[h]		X[h]		
Pregnancy[b]		X		X					X[h]				
Urinalysis[b]	X	X[q]		X[q]		X[q]		X[q]	X[h, q]		X[h, q]		
**Lab (Immunogenicity)**													

Scheduled visit	***1[m]***	***2[m]***		***3***		***4***		***5/ET***	***6[t]***		***7***		***Long-term follow-up***
Test & observations	***Screening***	***1st vaccination***		***2nd vaccination***		***Follow-up***		***Unblinding***	***3rd vaccination[h]***		***Follow-up[h]***		***Month 12 follow-up[i]***
Day	***-28~-1***	*1*	*8, 15, 22*	*29 ±3 days*	*36, 43*	*57 ±3 days*	*64, 71, 78, 85*	*197 ±15 days*	***197~242***	***7 days after Visit 6***	***14 days after Visit 6 ±3 days***	*253, 309*	*365 ±45 days*
Immune response assessment[c]		X		X[n]		X		X					X
T cell function (optional[o])		X				X							
**Lab (Exploratory)**													
**T cell function (optional[u])**									**X[h]**		**X[h]**		
**Spared sample[r]**	**X**					**X**							
**Immune response[s]**									**X[h]**		**X[h]**		
Vaccination		X		X					**X[h]**				
e-Diary instruction		X		X					**X[h]**				
Safety calls[d]			X		X		X			**X[s,h]**		X	
AEs/AESIs[p] /MAAEs/SAEs		X[k]	X[k]	X[k]	X[k]	X[k]	X[k]	X[l]	**X[l, h]**	**X[l, h]**	**X[l, h]**	X[l]	X[l]
COVID-19 infection surveillance		X	X	X	X	X[e]	X[e]	X[e]	**X[e, h]**	**X[e, h]**	**X[e, h]**	X[e]	X[e]
Concomitant Medication		X		X		X		X[f]	**X[f, h]**		**X[f, h]**		

a: Body weight and height will be assessed at Visit 1 only.
b: Screening for pregnancy and proteinuria will be performed by urine sample on Day 1, Day 29. When positive urine pregnancy test is presented, it should be confirmed by a serum test. Serum testing in lieu of urine tests will not be considered a protocol deviation. Proteinuria is checked via urine routine as baseline.
c: For anti-S1-RBD IgG levels, SARS-CoV-2 neutralizing titers, and antibody titers which inhibit S1-RBD: ACE2 binding.
d: All subjects will receive safety calls that will serve both to monitor for unsolicited AEs, including AESIs, and to monitor for symptoms of COVID-19.
e: Each week (i.e., every 7 days) the subject will receive a prompt on their smartphone device to regularly surveillance for signs and symptoms of COVID-19. Record medication for potential COVID-19 on COVID-19 page.
f: Only record medications for MAAE and SAE.

g: 28 days after 2nd vaccination
h: **Only for subjects in vaccine group and have agreed to receive 3rd dose of UB-612.**
i: 12 months (336 days) after 2nd vaccination
j: Safety laboratory includes complete blood count (Hgb, Hct, RBC), WBC with differential, platelet count, creatinine, ALT, AST, total and direct bilirubin, hs-CRP, and ANA.
k: Active collection period
l: Passive surveillance period.
m: Visit 1 and 2 could be emerged as one visit.
n: Anti-S1-RBD IgG level only
o: Up to 100 aged >18-< 65 subjects in selected sites will be invited
p: To include PIMMC (listed in **Table 9-6**), or any newly identified potential AESI followed through 12 months after participants' final vaccination. Complications of COVID-19, also termed as ADE, (listed in **Table 9-5**) should be considered and reported as AESIs.
q: If subject encountered ≥ Grade 3 hypertension, the existence or deterioration of proteinuria will be checked .
r: An aliquot of spared serum is to be frozen and stored for UBI SARS-CoV-2 ELISA, Confirmatory SARS-CoV-2 ELISA, and future immunologic tests.
s: Subjects who qualified to receive 3rd dose will enter 2 weeks e-diary for follow-up and a safety call post 7 days post vaccination.
t: Visit 5 and Visit 6 could be the same day.
u: Approximately thirty aged >18-< 65 subjects and approximately thirty aged ≥ 65 years subjects in selected sites will be invited. When applicable, subjects who were assessed for T cell functions on Day 57 are preferred to be invited.
ADE (Antibody Dependent Enhancement), AESIs (Adverse Event of Special Interests), ANA (Anti-Nuclear Antibody), ET (Early Termination), MAAEs (Medically Attend Adverse Events)

Safety check group

Scheduled visit	***1*** [l]	***2*** [l]		***3***		***4***		***5/ET***	***6*** [q]		***7***		***Long-term follow-up***
Test & observations	***Screening***	***1st vaccination***		***2nd vaccination***		***Follow-up*** [f]		***Unblinding***	***3rd vaccination*** [g]		***Follow-up*** [g]		***Month 12 follow-up*** [h]
Day	***-28~-1***	*1*	*8, 15, 22*	*29 ±3 days*	*36, 43*	*57 ±3 days*	*64, 71, 78, 85*	*197 ±15 days*	***197~242***	***7 days after Visit 6***	***14 days after Visit 6 ±3 days***	*253, 309*	*365 ±45 days*
Informed consent	X								**X** [g]				
Inclusion/Exclusion Criteria	X	X											
Randomization		X											
**Contraindication to vaccination**				X					**X** [g]				
Demographics	X												
Medical history	X	X											
Physical Exam [a]	X	X		X		X		X	**X** [g]		**X** [g]		
Vital sign	X	X		X		X		X	**X** [g]		**X** [g]		X
ECG	X												
**Lab (Safety)**													
Blood routine [i]	X					X			**X** [g]		**X** [g]		
Biochemistry [i]	X					X			**X** [g]		**X** [g]		
Immunology [i]	X					X			**X** [g]		**X** [g]		
Pregnancy [b]		X		X					**X** [g]				
Urinalysis [b]	X	X [n]		X [n]		X [n]		X [n]	**X** [g, n]		**X** [g, n]		
**Lab (Exploratory)**													
**Spared sample** [o]	**X**					**X**							

Scheduled visit	***1*** [l]	***2*** [l]		***3***		***4***		***5/ET***	***6*** [q]		***7***		***Long-term follow-up***
Test & observations	***Screening***	***1st vaccination***		***2nd vaccination***		***Follow-up*** [f]		***Unblinding***	***3rd vaccination*** [g]		***Follow-up*** [g]		***Month 12 follow-up*** [h]
Day	***-28~-1***	*1*	*8, 15, 22*	*29 ±3 days*	*36, 43*	*57 ±3 days*	*64, 71, 78, 85*	*197 ±15 days*	***197~242***	***7 days after Visit 6***	***14 days after Visit 6 ±3 days***	*253, 309*	*365 ±45 days*
**Immune response** [g]									**X** [g]		**X** [g]		**X** [g]
Vaccination		X		X					**X** [g]				
e-Diary instruction		X		X					**X** [g]				
Safety calls [c]			X		X		X			**X** [p, g]		X	
AEs/AESIs[m]/MAAEs/SAEs		X [j]	X [j]	X [j]	X [j]	X [j]	X [j]	X [k]	**X** [k, g]	**X** [k, g]	**X** [k ,g]	**X** [k]	X [k]
COVID-19 infection surveillance		X	X	X	X	X [d]	X [d]	X [d]	**X** [d, g]	**X** [d, g]	**X** [d, g]	**X** [d]	X[d]
Concomitant Medication		X		X		X		X [e]	**X** [e, g]		**X** [e, g]		

a: Body weight and height will be assessed at Visit 1 only.
b: Screening for pregnancy and proteinuria will be performed by urine sample on Day 1 and Day 29. When positive urine pregnancy test is presented, it should be confirmed by a serum test. Serum testing in lieu of urine tests will not be considered a protocol deviation. Proteinuria will be checked via urine routine as baseline.
c: All subjects will receive safety calls that will serve both to monitor for unsolicited AEs, including AESIs, and to monitor for symptoms of COVID-19.
d: Each week (i.e., every 7 days) the subject will receive a prompt on their smartphone device to regularly surveillance for signs and symptoms of COVID-19. Record medication for potential COVID-19 on COVID-19 page.
e: Only record medications for MAAE and SAE.
f: 28 days after 2nd vaccination
g: Only for subjects in vaccine group and have agreed to receive 3rd dose of UB-612.
h: 12 months (336 days) after 2nd vaccination
i: Safety laboratory includes complete blood count (Hgb, Hct, RBC), WBC with differential, platelet count, creatinine, ALT, AST, total and direct bilirubin, hs-CRP, and ANA.
j: Active collection period
k: Passive surveillance period,
l: Visit 1 and 2 could be emerged as one visit.
m: To include PIMMC (listed in **Table 9-6**), or any newly identified potential AESI followed through 12 months after participants' final vaccination. Complications of COVID-19, also termed as ADE, (listed in **Table 9-5**) should be considered and reported as AESIs.
n: If subject encountered ≥ Grade 3 hypertension, the existence or deterioration of proteinuria will be checked.

o: An aliquot of spared serum is to be frozen and stored for UBI SARS-CoV-2 ELISA, Confirmatory SARS-CoV-2 ELISA, anti-S1-RBD IgG ELISA, S1-RBD:ACE2 binding inhibition ELISA, and future immunologic tests.
p: Subjects who qualified to receive 3rd dose will enter 2 weeks e dairy for follow-up and a safety call post 7 days post vaccination.
q: Visit 5 and Visit 6 could be the same day.
ADE (Antibody Dependent Enhancement), AESIs (Adverse Event of Special Interests), ET (Early Termination), MAAEs (Medically Attend Adverse Events)

Blood collection at scheduled visits
<u>Lot-to-lot consistency & Immunogenicity group</u>

Scheduled visit	***1***	***2***		***3***		***4***		***5/ET***	***6***		***7***		***Long-term follow-up***
Test & observations	***Screening***	***1st vaccination***		***2nd vaccination***		***Follow-up***		***Unblinding***	***3rd vaccination [a]***		***Follow-up [a]***		***Month 12 follow-up***
Day	***-28~-1***	*1*	*8, 15, 22*	*29 ±3 days*	*36, 43*	*57 ±3 days*	*64, 71, 78, 85*	*197 ±15 days*	***197~242***	***7 days after Visit 6***	***14 days after Visit 6 ±3 days***	*253, 309*	*365 ±45 days*
**Lab (Safety)**													
Blood routine	4.5					4.5			**4.5**		**4.5**		
Biochemistry	**3**					**3**			**3**		**3**		
Immunology	3					3			**3**		**3**		
**Lab (Immunogenicity)**													
Immune response assessment		20		5		20		20					20
**Lab (Exploratory)**													
**Spared sample**	**5**					**5**							
**Immune response***									**10[b]**		**10**		
Total blood collection	**15.5**	20		5		**35.5**		20	**20.5**		**20.5**		20

Total amount of blood collection: **157 mL.**
* An additional 56 mL of blood sample will be collected for the optional T cell assessments at **Visit 2, Visit 4, Visit 6 and Visit 7.**
a: Only for subjects in vaccine group and have agreed to receive 3rd dose of UB-612. An additional 10 mL of blood sample will be collected at Visit 6 and 14 days after Visit 6.
b: If Visit 5 and Visit 6 are the same visit, the 10mL blood sample for exploratory immune response will be not collected.

Safety check group

Scheduled visit	***1***	***2***		***3***		***4***		***5/ET***	***6***		***7***		***Long-term follow-up***
Test & observations	***Screening***	***1st vaccination***		***2nd vaccination***		***Follow-up***		***Unblinding***	***3rd vaccination[a]***		***Follow-up[a]***		***Month 12 follow-up***
Day	***-28~-1***	*1*	*8, 15, 22*	*29 ±3 days*	*36, 43*	*57 ±3 days*	*64, 71, 78, 85*	*197 ±15 days*	***197~242***	***7 days after Visit 6***	***14 days after Visit 6 ±3 days***	*253, 309*	*365 ±45 days*
**Lab (Safety)**													
Blood routine	4.5					4.5			**4.5**		**4.5**		
Biochemistry	**3**					**3**			**3**		**3**		
Immunology	3					3			**3**		**3**		
**Lab (Exploratory)**													
**Spared sample**	**5**					**5**							
**Immune response**									**10**		**10**		**10**
Total blood collection	**15.5**					**15.5**			**20.5**		**20.5**		**10**

Total amount of blood collection: **82** mL.

a: Only for subjects in vaccine group and have agreed to receive 3rd dose of UB-612. An additional 10 mL of blood sample will be collected at Visit 6, 14 days after Visit 6, and Day 365.

Diagram of the study design (N represented evaluable numbers)

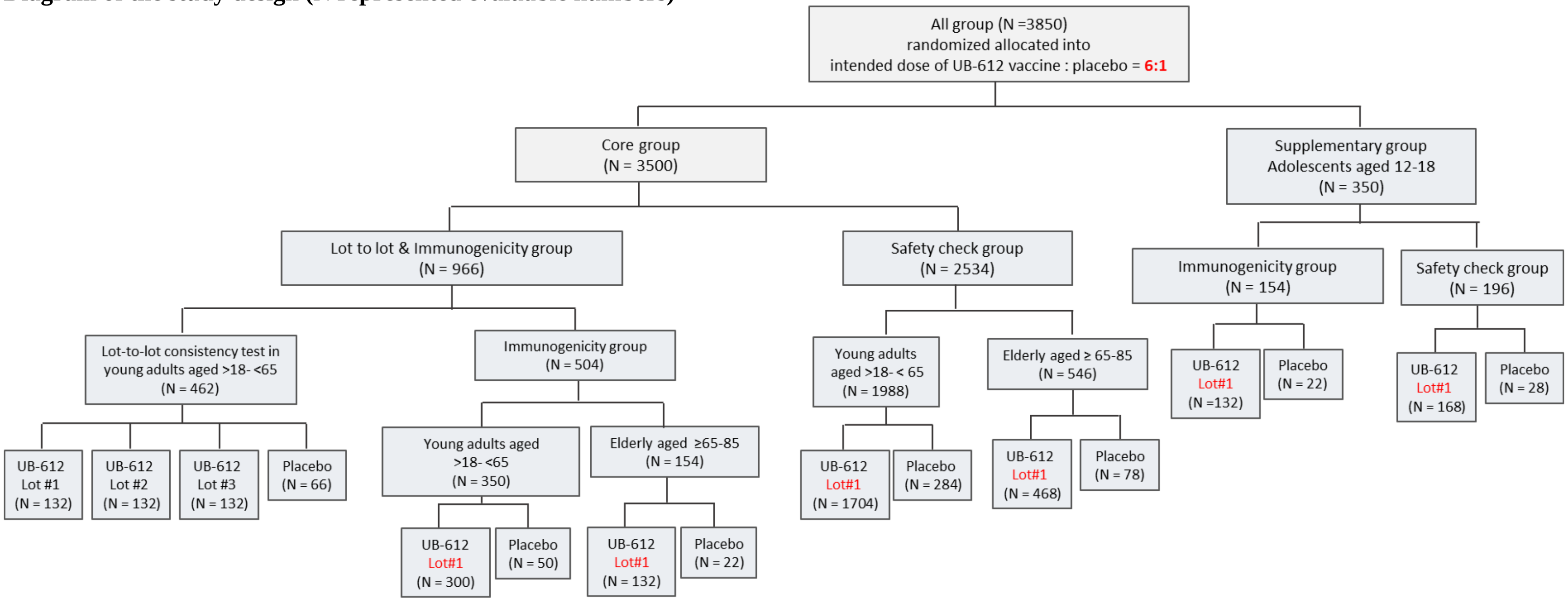

7.3 Discussion of Study Design

The primary objectives of this study are to evaluate the immune response after full immunization. Secondary objectives are to evaluate persistence of immune response, and safety and tolerability of UB-612 vaccine to prevent SARS-CoV-2 infection up to 12 months. COVID-19 can affect anyone, and the disease can cause symptoms ranging from mild to very severe. For some other illnesses caused by respiratory viruses (such as influenza), some people may be more likely to have severe illness than others because they have characteristics or medical conditions that increase their risk. These are commonly called "risk factors." Examples include older age or having certain underlying medical conditions. Older adults are at greatest risk of severe disease and death due to coronavirus disease 2019 (COVID-19). Globally, persons older than 65 years comprise 9% of the population, yet account for 30% to 40% of cases and more than 80% of deaths [13]. COVID-19 is a new disease. Currently there are limited data and information about the impact of many underlying medical conditions on the risk for severe illness from COVID-19. In this study design, adults of any age might be at an increased risk for severe illness from the virus that causes COVID-19 as defined who had at least one of the Charlson comorbidity index [14] category or obesity only (BMI $\geq$30 kg/m^2).

Per EUA requirement issued by TFDA, the sponsor should submit the lot-to-lot consistency report to demonstrate the quality of vaccine product. Core group is designed for EUA application. Among 3850 subjects, 462 evaluable subjects aged from >18 to < 65 will be enrolled for lot-to-lot consistency. Additional 350 evaluable adult subjects will be assigned into immunogenicity study. In order to demonstrate vaccine efficacy in elderly, authority requests EUA package should include at least 600 elder subjects who received the vaccine. Thus we plan to enrol around 770 randomized subjects aged from ≥ 65 to 85 years old, in which at least 154 evaluable subjects will have immunogenicity data to compare with young adults. After completion recruitment of the core group, 385 adolescents will be randomly recruited as supplementary group to extend vaccine indication. At least 154 evaluable adolescents (allocation rate 6:1) will have immune data to compare with adults.

There are two interim reports for EUA application. The first is for safety follow up in core group, and the second is for lot-to-lot consistency. Also, an additional interim report of immunogenicity and safety in adolescents, will be submitted for indication extension.

The surveillance for COVID-19 will be conducted as part of the study. The serological evidences in case of confirmed and/or severe COVID-19 will be described for the change of antibody level in vaccinated subjects. All subjects would be included in the exploratory objectives of protective efficacy against COVID-19 if case detected.

Unblinding will be carried out when the subject in core group or supplementary group has completed Visit 5. Subjects who receive the placebo vaccine will allow to withdraw from the study.

7.4 Selection of Study Population

7.4.1 Number of Planned Subjects

In core group, around 3850 adult subjects will be randomized to receive 100 µg UB-612 vaccine or placebo with 6:1 allocation rate. In supplementary group, around 385 adolescent subjects aged

from 12 to 18 years old will randomly receive UB-612 vaccine 100 µg or placebo with allocation rate 6:1, too.

7.4.2 Inclusion Criteria

To be eligible for study entry subjects must satisfy all of the following inclusion criteria:

1. Healthy male or non-pregnant female between the age of 12 to 85 years at time of enrolment.
2. Women of childbearing potential and men must agree to practice medically effective contraception from first vaccination until 3 months after the last vaccination. The acceptable effective contraception methods include:
    a. Male or female sterilization, implant, or intrauterine device;
    b. Injectable, pill, patch, ring plus one barrier method*;
    c. Two combined barrier methods*.

    *Effective barrier methods are diaphragm, male or female condoms, sponge, or spermicides (creams or gels that contain a chemical to kill sperm).
3. Able to understand the content and possible risks of informed consent and willing to sign the Informed Consent Form (ICF).
4. Able to understand and agrees to comply with all study procedures and be available for all study visits.
5. Ear temperature ≤ 38.0°C.
6. Healthy participants** who are determined by medical history, physical examination, and clinical judgment of the investigator to be eligible for inclusion in the study. In the investigator's clinical judgement, participant may have a stable and well-controlled comorbidity associated with an increased risk of progression to severe COVID-19.

    ** Healthy participants with preexisting stable disease, defined as disease not requiring significant change in therapy or hospitalization for worsening disease during the 12 weeks before enrolment and unlikely to require a significant change in therapy or hospitalization in the six months following enrollment, can be included.

7.4.3 Exclusion Criteria

Subjects will be excluded from the study if one or more of the following exclusion criteria is applicable:

1. History of anaphylaxis, urticarial, or other significant adverse reaction requiring medical intervention after receipt of a vaccine.
2. Female who is pregnant or positive in pregnancy test at screening or just prior to each vaccination administration.
3. Female who is breast-feeding or plans to breastfeed from the time of the first vaccination through 60 days after the last vaccination.
4. Any acute illness, as determined by the study investigator 3 days before first vaccination (these subjects can be re-scheduled).

5. Any major surgery one month before first vaccination (these subjects can be -rescheduled)
6. Known HIV antibody positive
7. Known active hepatitis B and hepatitis C disease. Active hepatitis means liver aminotransferase (AST and/or ALT) greater than 3xULN, and/or total bilirubin greater then 3xULN at screening.
8. Previous exposure to SARS-CoV-2 or receipt of an investigational or licensed product for the prevention of COVID-19, MERS or SARS.
9. Have history of Guillain-Barre syndrome.
10. Subjects who take part in another clinical study within 12 weeks prior to the day of informed consent.
11. Immune deficiency/disorder, whether due to genetic defect, immunodeficiency disease or immunosuppressive therapy
12. Subjects who plan to or are undergoing anti-cancer therapy
13. Platelet disorder or other bleeding disorder may cause injection contraindication.
14. Prior chronic administration (defined as ≥ 14 day of continuous use) of immunosuppressant or corticosteroids (equivalent to ≥ 20 mg daily of prednisone), cytotoxic treatment in last 6 months before first vaccination.
15. Prior administration of immunoglobulins and/or any blood products in last 4 months before first vaccination.
16. Receipt of any seasonal or pandemic influenza vaccine within 14 days, or any other nonstudy vaccine within 28 days, before study intervention administration.
17. Anticipated receipt of any seasonal or pandemic influenza vaccine within 14 days, or any other nonstudy vaccine within 28 days, after study intervention administration.
18. Receipt of short-term (<14 days) systemic corticosteroids. Study intervention administration should be delayed until systemic corticosteroid use has been discontinued for at least 28 days. Inhaled/nebulized, intra-articular, intrabursal, or topical (skin or eyes) corticosteroids are permitted.
19. Loss or donation of blood over 500 mL within 3 months prior to Screening Visit or intention to donate blood or blood products for transfusion during the study.
20. Any medical disease or condition that, in the opinion of the study investigator, may confound the results of the study or pose an additional risk to the subjects by their participation in the study.
21. Employees at the investigator’s site, of the Sponsor or the contract research organization (CRO) **who** directly involved in the conduct of the study.

7.4.4 Contraindications to Vaccination

7.4.4.1 Criteria for Delay of Study Treatment

The following conditions constitute a contraindication to vaccination and should be checked prior to second **and third** vaccinations:

1. Subject has a fever or other acute illness within 24 hours before vaccination.
2. Clinically significant acute illness at the time of vaccination, including concurrent symptoms which may be considered as a potential COVID-19 illness. This does not include minor illnesses, such as diarrhea or mild upper respiratory tract infection.
3. An illness which in the judgement of the investigator may interfere with reactogenicity and 7-day safety assessments after vaccination.

If any AEs other than listed above or fever occurred at the time scheduled for vaccination, the subject may be vaccinated at a later date no later than 7 days when symptoms or fever are resolved.

7.4.4.2 Criteria for Discontinuation of Study Treatment

The following conditions constitute a contraindication to vaccination and should be checked prior to second **and third vaccinations**:

1. Has any Grade 4 adverse reaction related to IP within 7 days after **previous vaccination**.
2. Has any SAE related to **previous** dose related to IP during the follow-up of **previous vaccination**.
3. Subject has or develops the symptom or condition listing in 1 or more exclusion criteria.
4. Subject diagnoses with SARS-CoV-2 infection or has suspected SARS-CoV-2 infection based on symptoms according to investigator’s judgment (lab test conformation is not necessary).
5. Subject becomes pregnant.
6. Any condition that in the opinion of the investigator would be a contraindication to **vaccinations.**

Any subjects who receive the vaccination with dosage deviation or who does not receive the second **and third** vaccination on schedule may not necessarily be withdrawn from the study as further study procedures and the follow-up visits may be performed which will be decided by sponsor.

7.4.5 Screen Failures

Screen failures are defined as participants who consent to participate in the clinical study but are not eligible randomly assigned to study intervention. **A minimal set of screen failure information is required demography, screen failure details, and eligibility criteria.**

Participants who do not meet the selection criteria in this study may be allowed to rescreened under different screen number.

7.4.6 Method of Assigning Subjects to Treatment Groups

The study vaccine and placebo will be randomly assigned to subjects. The randomized sequence of study treatments will be generated by an independent qualified biostatistician. The randomization schedule will be kept and maintained by the designated personnel before the study begins and until the database lock. The independent biostatistician prepares the randomization schedules via the SAS software (9.4 or higher version).

For core group, around 3850 participants will be randomly assigned to UB-612 vaccine 100 µg or placebo with a 6:1 randomization ratio (vaccine: placebo) by block randomization. In additional, a total of 966 evaluable subjects including 812 evaluable young adult and 154 evaluable elderly subjects from core group will be apportioned to immunogenicity and **lot-to-lot group** with a randomization ratio of 6:1 to UB-612 vaccine (696 evaluable young adult subjects and 132 evaluable elderly subjects) and placebo (116 evaluable young adult subjects and 22 evaluable elderly subjects). Furthermore, the 396 evaluable young adult subjects in immunogenicity group assigned to UB-612 vaccine will be allocated to 3 specified vaccine lots with a 1:1:1 ratio for evaluating the lot-to-lot consistency. Therefore, a total of 462 evaluable young adult subjects of immunogenicity group will be randomized to 3 vaccine lots and placebo with a ratio of 2:2:2:1 (vaccine lot 1: vaccine lot 2: vaccine lot 3: placebo).

In supplementary group, around 385 adolescent subjects aged from 12 to 18 years old will randomly receive UB-612 vaccine 100 µg or placebo with allocation rate 6:1, too.

Eligible subjects will receive a random number at randomization in form of a sealed envelope. The authorized unblinded dispenser(s)/administrator(s) who involve in the treatment of vaccine/ placebo will be required to open the envelope and administer the corresponding treatment assigned by the envelope. For any reason that a subject withdraws from the study prematurely after the randomization, his/her randomization number will not be reused. The next eligible subject will receive the lowest available randomization number.

7.4.7 Blinding

This is an observer-blind study, as the physical appearance of the investigational vaccine candidates and the placebo may differ. Only the dispenser(s)/administrator(s) will be unblinded. These study staffs are responsible for receiving, dispensing, preparing and administrating the study intervention. The investigational products should be administered in a manner that prevents the subjects to identifying their treatment based on vaccine physical appearance. The dispenser(s)/ administrator(s) should not participate in any of the study clinical evaluations or assay, and keep minimum contacts with other study personnel.

Neither the subjects nor the investigators, sponsor staffs, clinical monitors, study coordinators other than dispenser(s)/administrator(s), or other site staff who involve in clinical evaluation of the subjects will be aware of the treatment received either at the time of randomization or later, throughout the conduct of the trial. **These study personnel will be blinded until Visit 5. Unblinding will be carried out via disclosed Randomization Envelop during Visit 5.**

The treatment codes will be not prematurely broken unless an emergency situation, when the appropriate management of the subject necessitated knowledge of the treatment allocation, occurred. In the event of a medical emergency, if possible, the clinical monitor should be contacted before the treatment code blind is broken to discuss the need for unblinding. For unblinding a

subject, the treatment code blind can be obtained by the investigator, by opening the emergency envelope.

The sponsor must be notified immediately if the treatment code blind is broken. The date, time, and reason that the blind is broken must be recorded in the source document and the same information (except the time) must be recorded on the eCRF.

7.4.8 Removal of Subjects

7.4.8.1 Removal of Subjects from Immunogenicity Analysis

Subjects may stop study vaccine and withdraw from the immunogenicity analysis for any of the following reasons:

- Subjects do not receive **the first** 2 doses of UB-612 vaccine (refer to Section 7.4.4).
- Administration of prohibited medication/treatment/vaccine during pre-specified period which is enough to interfere with immunogenicity.

Subjects who do not comply with the protocol will be replaced. Subjects who stop study vaccine for any other reason (i.e., AE) will not be replaced.

7.4.8.2 Removal of Subjects from the Study

Subjects may withdraw from the study for any of the following reasons:

- Lost to follow-up
- Consent withdrawal
- Death
- Any pathological event, clinical adverse event, or any change in the subject's status giving indication to the doctor that further participation in the study may not be the best interests of the subject, according to investigator's discretion.
- Study terminated by sponsor

Subjects who withdraw consent will be replaced.

Subjects are free to withdraw from the study at any time without providing reason(s) for withdrawal and without prejudice to further research treatment. The reason(s) for withdrawal will be documented in the case report form (CRF).

Subjects withdrawing from the study, except subject death, will be encouraged to complete the same final evaluations (as Visit 5 procedure) within 7 days after withdrawal, as subjects completing the study according to this protocol, particularly safety evaluations. The aim is to record data in the same way as for subjects who completed the study.

Reasonable efforts will be made to contact subjects who are lost to follow-up (where possible, at least 3 telephone calls). These efforts must be documented in the subject's file.

7.4.8.3 Study Termination

The sponsor has the right to terminate the study at any time in case of SAEs or if special

circumstances concerning the study agent or the company itself occur, making further research treatment of subjects impossible. In this event, the investigator(s) will be informed of the reason for study termination.

7.4.8.4 Reporting and Follow-up of Pregnancies

A positive urine pregnancy test should be confirmed by a serum pregnancy test. A negative pregnancy result is required before the subject may receive the study treatment. Subjects who become pregnant while on study must immediately discontinue study treatment. The pregnancy must be reported to sponsor within 24 hours of the investigator's or study site staff's acknowledgement of the pregnancy. Pregnancies for female subjects, or for the female partners of male subjects occurring during the study period, must be reported to the sponsor. Pregnancies should be handled and reported as AEs.

The investigator should inform the subjects of the risks of continuing with the pregnancy and the possible side effects to the fetus. The outcome of all pregnancies (spontaneous miscarriage, elective termination, normal birth, or congenital abnormality) must be followed up and documented even if the subject is discontinued from the study. If subject is pregnant **from first vaccination until 3 months after the last vaccination**, she will be encouraged to contact site and will be follow-up till delivery.

All reports of congenital abnormalities/birth defects and spontaneous miscarriages should be handled and reported as SAEs, to be reported within 24 hours of site awareness (regardless of interval since study treatment). Elective abortions should be handled and reported as AEs.

7.4.8.5 SARS-CoV-2 infection

Subjects encountered SARS-CoV-2 infection will not withdraw from the study, unless other withdrawal reason judged by investigators.

7.5 Investigational Products

7.5.1 Identity of Investigational Products

Name	UB-612	Saline Placebo
Characteristics & Physical State	**White to off-white suspension without foreign objects**	Transparent liquid
Formulated & Supplied by	UBI Pharma Inc.	Taiwan Biotech Co., LTD
Storage Conditions	Cooled (2℃ - 8℃) following receipt at site until the time of use	< 25℃
Shipments	Cooled (2℃ - 8℃)	< 25℃
Package	A disposable multi-dose vial containing 200 µg/mL UB-612 protein/peptide as the following:<br>- 200 µg/mL: 176 µg S1-RBD-sFc protein and 24 µg of six peptides/per 1 mL included	A plastic vial with 0.9% sodium chloride solution for injection as open-label supply will be provided.
Batch No.	**Lot #1: 303981**	**1MN2B022**

	**Lot #2: 304007**<br>**Lot #3: 304008**	

Each vial of 6.5 mL of UB-612 vaccine will be supplied in 10 mL glass vial. The vaccine lots used in this study have been tested and released by the quality control department of the UBI Pharma Inc.

7.5.2 Investigational Products Administered

7.5.2.1 Injection Volume(s)

	1st vaccination	2nd vaccination	**3rd vaccination**
Active vaccine group	UB-612 vaccine 100 µg, 0.5 mL	UB-612 vaccine 100 µg, 0.5 mL	**UB-612 vaccine 100 µg, 0.5 mL**
Placebo group	Normal saline 0.9%, 0.5 mL	Normal saline 0.9%, 0.5 mL	**N/A**

7.5.2.2 Administration

For this trial, the **first two doses** of UB-612 vaccine or saline placebo must be injected by an intramuscular (IM) route spaced 28 days apart. Injections on Day 1 and Day 29 will be given into non-dominant deltoid muscles, as detailed in Pharmacy Manual. **The 3rd dose of UB-612 will be given after unblinding once subjects qualified.**

7.5.2.3 Emergency Event Management

The subjects in each group will be closely monitored by the study staff at least 30 minutes after vaccination (for the change of vital signs or acute anaphylactic event). Subjects will be encouraged to quickly report any symptoms at the time during this period. The necessary rescue material, equipment, and appropriate medications will be available in the clinic to allow rapid intervention in case of anaphylaxis or other emergency.

7.5.3 Packaging and Labeling

The study packaging of UB-612 vaccine will be performed by UBI Pharma Inc.

All packaging and labeling operations will be performed according to Good Manufacturing Practice for Medicinal Products and the relevant regulatory requirements. The labels for the outer box/vial of the study vaccine contain the following information: the name/address/telephone number of UBI Pharma Inc., Product name, Study code, Indication, Package size, Dosage unit, Manufacture company, Batch No., Manufacture date, Expiration date, Storage conditions, Active ingredient concentration (only for UB-612 protein/peptide), Injection method, and words of caution stating the product is for investigational and clinical trial use only.

7.5.4 Prior and Concomitant Therapy

There is no specific known evidence of contraindications between the ingredients of UB-612 vaccine and other prior and concomitant therapy. Concomitant medications and therapies will be recorded beginning 1 month prior to 1st vaccination, as well as during study period.

7.5.4.1 Prohibited Medication/Therapy

The following medications or treatments which may affect the immunogenicity and clinical efficacy assessments will be prohibited in subjects who assess immunogenicity:

- Immunosuppressant, cytotoxic treatment until Day 197
- Immunoglobulins and/or any blood products until Day 197
- Systemic corticosteroids ($\geq$ 20 mg/day of prednisone or equivalent) until Day 197
- Investigational product (including drug, vaccine) during study period
- Any seasonal or pandemic influenza vaccine within 14 days, or any other nonstudy vaccine within 28 days, after study intervention administration. Licensed COVID-19 vaccine is prohibited through the whole study period.

The following medications or treatments which may affect the immunogenicity and clinical efficacy assessments will be prohibited in subjects who assess safety only:

- Immunosuppressant, cytotoxic treatment until Day 57
- Immunoglobulins and/or any blood products until Day 57
- Systemic corticosteroids ($\geq$ 20 mg/day of prednisone or equivalent) until Day 57
- Investigational product (including drug, vaccine) during study period
- Any seasonal or pandemic influenza vaccine within 14 days, or any other nonstudy vaccine within 28 days, after study intervention administration. Licensed COVID-19 vaccine is prohibited through the whole study period.

8 TIMING OF STUDY PROCEDURES

8.1 Study Procedures

8.1.1 Visit 1 (Day -28~-1 ± 3 days) –Screening

(1) Record date of informed consent will be signed. The following should be documented in the subject's medical chart: that they are participating in this study that informed consent has been obtained and that a copy of the consent has been given to the subject.

(2) Assign screen number

(3) Eligibility: Assess against the inclusion and exclusion criteria

(4) Demographics

(5) Medical history and concurrent diseases.

(6) Conduct physical exam including the measurement of weight and height

(7) Measure vital signs

(8) Perform 12 lead ECG

(9) Collect urine sample for urinalysis

(10) Collect blood sample for following tests:

- ➢ Blood routine tests
- ➢ Biochemistry tests
- ➢ Immunology test
- ➢ **Spared sample (tests for detecting serum antibody against SARS-CoV-2 or further researches)**

8.1.2 Visit 2 (Day 1) –Baseline: 1st Vaccination

(1) Recheck eligibility: Assess against the inclusion and exclusion criteria

(2) Randomization; assign randomization number

(3) Medical history and concurrent diseases. Any pre-vaccination medical event will be recorded as medical history since last visit.

(4) Conduct physical exam

(5) Measure vital signs

(6) Perform a urine pregnancy test.

(7) If subject encountered ≥ Grade 3 hypertension, subject should be checked the existence or deterioration of proteinuria or other renal damage sign.

(8) Collect blood sample before vaccination for following tests only for subjects in lot-to-lot consistency /immunogenicity group:

- ➢ Immune response assessments

- ➢ T cell function (optional)

(9) Perform 1st vaccination by unblinded administrator.

(10) Observe closely during vaccination and at least 30 minutes after vaccination in each group (for the change of vital signs or acute anaphylactic event) by blinded site staff.

(11) Instruct the subject to monitor body temperature and complete self-evaluation e-diary correctly.

Subject will be instructed to record any solicited adverse events occurring during a 7-day post-vaccination period on the e-diary system. Meanwhile, skin allergic reaction should be monitored for 14 days via e-diary. If the subject perceives any signs or symptoms are progressing or serious, contact the investigator or their delegates immediately. Additional return visits can be scheduled by the investigators when necessary.

(12) Review concomitant medications.

8.1.3 Day 8, 15, and 22 –Safety calls

Safety calls on Day 8, Day 15 and Day 22 to monitor subjects' unsolicited AEs and symptoms of COVID-19. Calls on Day 8 and Day 15 should be inquire the phenomenon of allergic skin reaction, especially itching and redness, or other unsolicited allergy reaction. No matter injection site or extending to other body site, site staff should arrange unscheduled visit to verify the allergic reaction and check HLA typing if indicated.

8.1.4 Visit 3 (Day 29 ± 3 day) –2nd Vaccination

(1) Check contraindication for second vaccination and the possibility of delay vaccination

(2) Conduct physical examination.

(3) Measure vital signs.

(4) Perform a urine pregnancy test.

(5) If subject encountered ≥ Grade 3 hypertension, subject should be checked the existence or deterioration of proteinuria or other renal damage sign.

(6) Collect blood sample before vaccination for following tests only for subjects in lot-to-lot consistency/immunogenicity only:

- ➢ Anti-RBD-S1 antibody

(7) Perform 2nd vaccination by unblinded administrator.

(8) Observe closely during vaccination and at least 30 minutes after vaccine administration for the change of vital sign or acute anaphylactic event at site by blinded site staff.

(9) Instruct the subject to monitor body temperature and complete self-evaluation e-diary correctly.

Subject will be instructed to record any solicited adverse events occurring during a 7-day post-vaccination period on the e-diary system. Meanwhile, skin allergic reaction should be monitored for 14 days via e-diary. If the subject perceives any signs or

symptoms are progressing or serious, contact the investigator or their delegates immediately. Additional return visits can be scheduled by the investigators when necessary.

(10) Review concomitant medications.

(11) Record and report adverse event, serious adverse event, or any symptoms of COVID-19 if any has occurred since the previous visit.

8.1.5 Day 36 and 43 –Safety calls

Safety calls on Day 36 and 43 to monitor subjects' unsolicited AEs and symptoms of COVID-19.

8.1.6 Visit 4 (Day 57 ± 3 day) –Follow-up

(1) Conduct physical examination.

(2) Measure vital signs.

(3) If subject encountered ≥ Grade 3 hypertension, subject should be checked the existence or deterioration of proteinuria or other renal damage sign.

(4) Collect blood sample for following tests for subjects in lot-to-lot consistency /immunogenicity group:

- Immune response assessments
- T cell function (optional)

(5) Collect blood sample for following tests for all subjects:

- Blood routine tests
- Biochemistry test
- Immunology test
- **Spared sample (tests for detecting serum antibody against SARS-CoV-2 or further researches)**

(6) Review concomitant medications.

(7) Record and report adverse event, serious adverse event, or any symptoms of COVID-19 if any has occurred since the previous visit.

After Day 57, each week (i.e., every 7 days) the subject will receive a prompt on their smartphone device to regularly surveillance for signs and symptoms of COVID-19.

8.1.7 Day 64, Day 71, Day 78, and Day 85 –Safety calls

Safety calls on Day 64, Day 71, Day 78, and Day 85 to monitor subjects' unsolicited AEs and symptoms of COVID-19.

8.1.8 Visit 5 (Day 197 ± 15 days) –Unblinding / Early Termination

(1) Conduct physical examination.

(2) Measure vital signs.

(3) If subject encountered ≥ Grade 3 hypertension, subject should be checked the existence or deterioration of proteinuria or other renal damage sign.

(4) **Unblinding**

(5) **Withdraw subjects who received placebo**

(6) Collect blood sample for following tests only for subjects in lot-to-lot consistency /immunogenicity group:

- Immune response assessments

(7) Review concomitant medications.

(8) Record and report adverse event, serious adverse event, or any symptoms of COVID-19 if any has occurred since the previous visit.

The subject will receive a prompt on their smartphone device to regularly surveillance for signs and symptoms of COVID-19 each week.

8.1.9 Visit 6 (Day 197~242) –3rd Vaccination

The following procedures will only be performed on subjects who agreed to have 3rd vaccination. The Visit 5 and Visit 6 could be the same day.

(1) Collect inform consent form for vaccine group who agreed to have 3rd vaccination.

(2) Check contraindication for the third vaccination and the possibility of delay vaccination

(3) Conduct physical examination.

(4) Measure vital signs.

(5) Perform a urine pregnancy test.

(6) If subject encountered ≥ Grade 3 hypertension, subject should be checked the existence or deterioration of proteinuria or other renal damage sign.

(7) Collect blood sample for following tests:

- **Immune response assessments**
- **Blood routine tests**
- **Biochemistry test**
- **Immunology test**
- **T cell function (optional)**

(8) Perform 3rd vaccination

(9) Observe closely during vaccination and at least 30 minutes after vaccine administration for the change of vital sign or acute anaphylactic event at site by site staff.

(10) Instruct the subject to monitor body temperature and complete self-evaluation e-diary correctly.

(11) Review concomitant medications.

(12) Conduct COVID-19 surveillance

(13) Record and report adverse event, serious adverse event, or any symptoms of COVID-19 if any has occurred since the previous visit.

8.1.10 Safety call on 7 days after Visit 6 (Only for subjects who have 3rd vaccination)

Safety calls on 7 days after Visit 6 to monitor subjects' unsolicited AEs and symptoms of COVID-19. Call should be inquired the phenomenon of allergic skin reaction, especially itching and redness, or other unsolicited allergy reaction. No matter injection site or extending to other body site, site staff should arrange unscheduled visit to verify the allergic reaction and check HLA typing if indicated.

8.1.11 Follow up on 14 days after Visit 6 (± 3 day) (Only for subjects who have 3rd vaccination)

(1) Conduct physical examination.

(2) Measure vital signs.

(3) If subject encountered ≥ Grade 3 hypertension, subject should be checked the existence or deterioration of proteinuria or other renal damage sign.

(4) Collect blood sample for following tests:

- **Immune response assessments**
- **Blood routine tests**
- **Biochemistry test**
- **Immunology test**
- **T cell function (optional)**

(5) Review concomitant medications.

(6) Conduct COVID-19 surveillance

(7) Record and report adverse or serious adverse event.

8.1.12 Day 253 and Day 309- Bi-monthly Safety calls

After Day 197, subjects will enter the **long-term follow-up** with a safety call bi-monthly on Day 253 and Day 309 to monitor subjects' unsolicited AEs and symptoms of COVID-19.

8.1.13 Long-term follow-up (Day 365 ± 45 days)

(1) Measure vital signs.

(2) If subject encountered ≥ Grade 3 hypertension, subject should be checked the existence or deterioration of proteinuria or other renal damage sign.

(3) Collect blood sample for following tests only for subjects **have 2 vaccinations** in lot-to-lot consistency /immunogenicity group:

- Immune response assessments

(4) **Collect blood sample for following tests only for subjects have 3rd vaccination:**

- **Immune response assessments**

(5) Record adverse events, including AESI, MAAE, SAE, or any symptoms of COVID-19 if any has occurred since the previous visit.

8.2 Early Termination (ET)

For subjects in lot-to-lot consistency/immunogenicity group who discontinue this study earlier, a final follow-up may be arranged not **later** than 7 days and all study procedures listed for Visit 5 should be completed.

8.3 Unscheduled Visit

Subjects who suffer from Grade 3/4 solicited AE should return to site ASAP for further survey and treatment. All AEs recognised during the unscheduled visits are medically attended adverse events (MAAE), except for confirmed COVID-19 disease.

- If a Grade 3 local reaction, systemic event, or fever is reported in the reactogenicity e-diary, a telephone contact should occur to ascertain further details and determine whether a site visit is clinically indicated.
- If suspected Grade 4 local reaction systemic event or fever is reported in the reactogenicity e-diary, a site visit should occur to confirm whether the event meets the criteria for Grade 4.

8.4 COVID-19 Surveillance (All Subjects)

If a subject experiences any of the following (irrespective of perceived etiology or clinical significance), he or she is instructed to contact the site immediately. During the 7 days following each vaccination, potential COVID-19 symptoms that overlap with solicited systemic events (i.e. fever, chills, new or increased muscle pain, diarrhea, vomiting) should not trigger a potential COVID-19 illness visit unless, in the investigator's opinion, the event is more indicative of a possible COVID-19 illness than vaccine reactogenicity.

8.4.1 Taiwan CDC Criteria for Case Definition of COVID-19

Criteria	Definition
**Clinical Presentation Criteria**	One or more of the following:<br>(1) Fever (≥ 38 °C) or symptoms of acute respiratory tract infection.<br>(2) Abnormal sense of smell (anosmia), abnormal sense of taste (dysgeusia), or diarrhea of unknown etiology.<br>(3) .Community-acquired pneumonia (CAP) highly suspected to be COVID-19 by doctors
**Laboratory Diagnosis Criteria**	One or more of the following:<br>(1) Pathogen (SARS-CoV-2) isolated and identified from a clinical specimen (nasopharyngeal swab, throat swab,

	expectorated sputum, or lower respiratory tract aspirates).<br>(2) Positive molecular biological testing for viral (SARS-CoV-2) RNA from a clinical specimen (nasopharyngeal swab, throat swab, expectorated sputum, or lower respiratory tract aspirates).
**Epidemiological Criteria**	One or more of the following within 14 days prior to symptom onset:<br>(1) History of traveling or living abroad, or contact with symptomatic (fever or other respiratory tract infection symptoms) individuals returning from abroad.<br>(2) History of close contact with symptomatic suspected or confirmed case(s), including caring for or interacting with these individuals, or direct contact with body fluid or respiratory secretions without adequate personal protective equipment (PPE).<br>(3) History of cluster related to confirmed cases.
**Reporting Requirements for COVID—19**	Any cases with one or more of the following conditions should be reported to the Taiwan Centers for Disease Control (Taiwan CDC):<br>(1) Meet clinical presentation criteria (1) **AND** one or more epidemiological criteria.<br>(2) Meet clinical presentation criteria (2) **AND** any of epidemiological criteria (1)) or (2).<br>(3) Meet clinical presentation criteria (3).<br>(4) Meet laboratory diagnosis criteria.
**Case Definition**	(1) Suspected case: meet clinical presentation criteria but not laboratory proven, plus history of close contact with symptomatic confirmed case(s) within 14 days prior to symptom onset.<br>(2) Confirmed case: meet laboratory diagnosis criteria, regardless. of clinical signs and symptoms.

8.4.2 Intercurrent COVID-19

If, at any time, a subject develops acute respiratory illness, for the purposes of the study she will be considered to potentially have COVID-19 illness. **In this circumstance, the subject should contact the site, an in-person or telemedicine contact should occur, and assessments should be conducted, as appropriate.** The assessments will include a clinical specimen defined above, which will be tested at a laboratory using an TFDA-approved polymerase chain reaction (PCR) test to detect SARS-CoV-2.

Taiwan CDC Criteria for case definition of COVID-19 is described in Section 8.4.1, and subjects who fulfill the reporting criteria should report to CDC. The diagram for COVID-19 surveillance is as below:

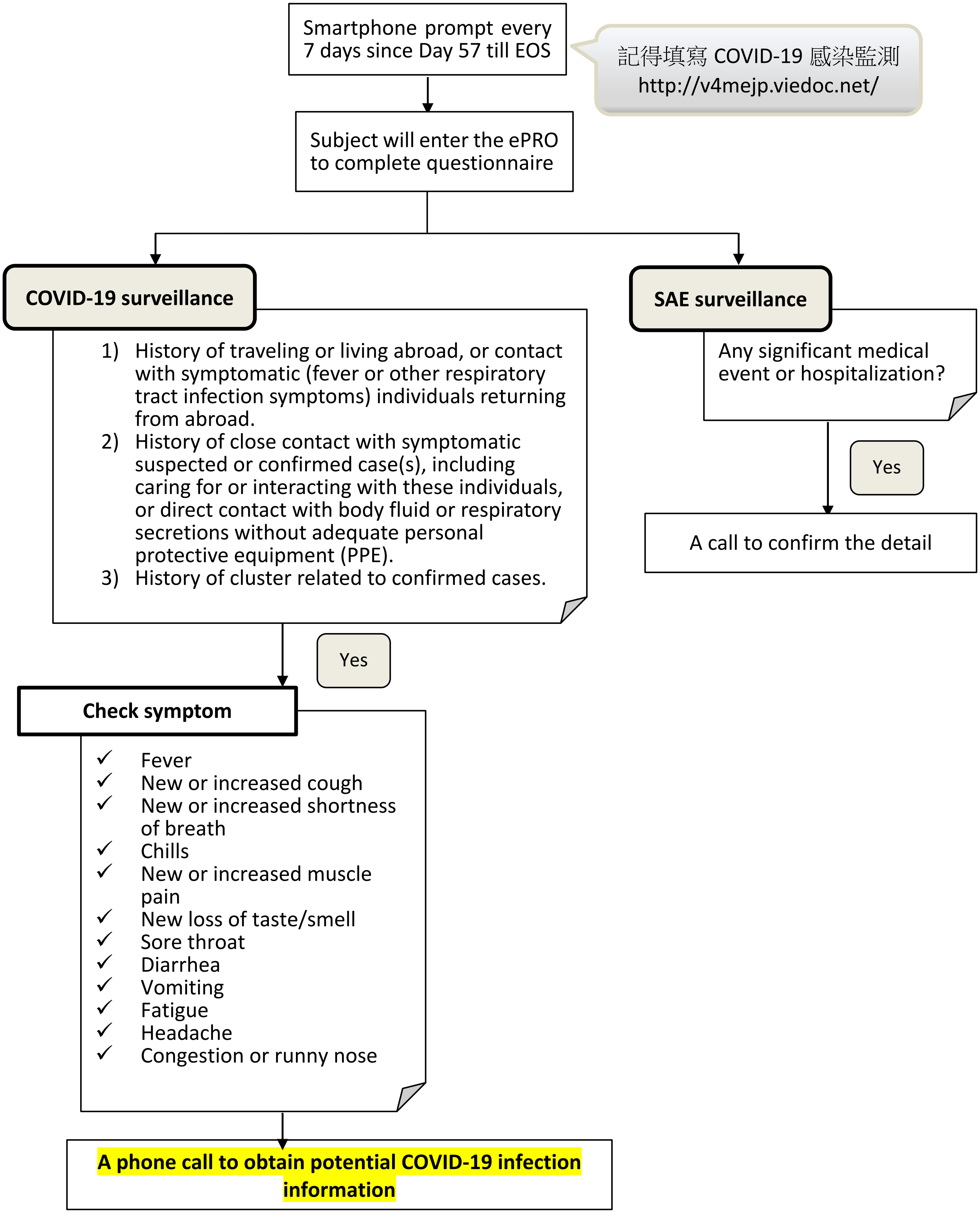
Smartphone prompt every 7 days since Day 57 till EOS
記得填寫 COVID-19 感染監測
http://v4mejp.viedoc.net/
Subject will enter the ePRO to complete questionnaire
COVID-19 surveillance
1) History of traveling or living abroad, or contact with symptomatic (fever or other respiratory tract infection symptoms) individuals returning from abroad.
2) History of close contact with symptomatic suspected or confirmed case(s), including caring for or interacting with these individuals, or direct contact with body fluid or respiratory secretions without adequate personal protective equipment (PPE).
3) History of cluster related to confirmed cases.
Yes
Check symptom
✓ Fever
✓ New or increased cough
✓ New or increased shortness of breath
✓ Chills
✓ New or increased muscle pain
✓ New loss of taste/smell
✓ Sore throat
✓ Diarrhea
✓ Vomiting
✓ Fatigue
✓ Headache
✓ Congestion or runny nose
A phone call to obtain potential COVID-19 infection information
SAE surveillance
Any significant medical event or hospitalization?
Yes
A call to confirm the detail

8.4.3 Potential COVID-19 Infection

Through ePRO system, the potential COVID-19 infection will be monitored. Once the events occur, the system alert will be sent to notice the study team members. The in-person or telemedicine contact will be conducted, optimally within 3 days after onset of illness. Several contacts may be required to obtain the following information:

- **Record potential COVID-19 illnesses, as appropriate. Note: Potential COVID-19 illnesses that are consistent with the clinical endpoint definition will be recorded on a COVID-19 CRF and will not be listed as AEs.**
- **Record the results of COVID-19 testing by an authorities approved test, if available**
- **Schedule an appointment for the subject to return for the potential COVID-19 convalescent visit once he or she has recovered.**
- **The investigator or designee completes the CRFs on COVID-19 page.**

8.4.4 COVID-19 Convalescent Visit

This visit may be conducted 28 to 35 days after the Potential COVID-19 Illness Visit.

- Record AEs, as appropriate.

  Note: Potential COVID-19 illnesses that are consistent with the clinical endpoint definition should not be recorded as AEs. These data will be captured as efficacy assessment data only on the relevant pages of the CRF, as these are expected endpoints.
- Record details of any of the prohibited medications specified in Section 7.5.4.1 received by the subject if required for his or her clinical care.
- **Collect blood samples for immunogenicity testing:**
  - **UBI SARS-CoV-2 ELISA, IgG S1-RBD ELISA, ACE2 binding inhibition ELISA and neutralization tests (~20 mL)**
- Collect/update COVID-19–related clinical and laboratory information
- Complete the source documents
- The investigator or an authorized designee completes the CRFs on COVID-19 page.

8.5 Duration of Treatment

The duration of each visit is expected to last between 2 to 3 hours, barring any unexpected adverse reactions.

It takes about 13 months for each subject to participate in the study, from recruiting to the last visit. Some subjects may withdraw the study during the course of the study.

9 IMMUNOGENICITY AND SAFETY VARIABLES

The planned schedule of assessments is in Section 7.2.

9.1 Informed Consent Form

The investigator or designee must explain to each subject the nature of the study, its purpose, the procedures involved, the expected duration, the potential risks and benefits involved and any potential adverse events. Each subject will be informed that participation in the study is voluntary and that they can be withdrawn from participation at any time.

All subjects must provide a signed and dated informed consent at Visit 1. An informed consent form must be approved by the Institutional Review Board (IRB), Ethics Committee (EC), and/or the applicable health authorities.

9.2 Demographics / Other Baseline Characteristics

The demographic and baseline characteristic data for subjects will be collected at Visit 1. The demographics include date of birth, age, sex and ethnicity. Relevant history/conditions include all those present prior to the administration of study vaccine that are listed below:

- Relevant medical history,
- All current medical conditions,
- Allergy history,

Whenever possible, diagnoses but not symptoms should be recorded.

9.3 Eligibility

Eligibility should be checked by the investigator at Visit 1 and rechecked at Visit 2 before vaccination, these 2 visits could be emerged as one. **For subjects agreed to receive 3rd vaccination, eligibility should be rechecked at Visit 6.**

9.4 Administration

The unblinded staff takes a vial of investigational vaccine/placebo, use a disposable syringe to extract vaccine/placebo and intramuscularly inject it into the middle of the lateral deltoid muscle of the subject's upper arm.

Vaccine administration will be recorded. The containers from which the vaccine is administered to the subjects will be retained for confirmation.

9.5 Safety and Immunogenicity Measurements Assessed

9.5.1 Safety Variables

9.5.1.1 Physical Examination

Complete physical examinations should be conducted by investigator/site staff at all visits. A complete physical exam will include the examination of general appearance, HEENT (head, ears, eyes, nose, throat), neck (including thyroid), lymph nodes, skin, cardiovascular, pulmonary, abdomen, neurological system and musculoskeletal/joints.

Body weight and height will be collected at Visit 1. Body weight will be measured in indoor clothing, but without shoes and blanket to the nearest 0.1 kilogram (kg). Body height will be measured in centimeters (cm).

It must be recorded when any abnormality has been found out.

9.5.1.2 Vital Sign

Systolic/diastolic blood pressure, pulse, respiratory rate, and ear temperature will be collected at all visits.

For two vaccination visits, vital sign should be measured prior to and after vaccine administration. All subjects in each group will be closely monitored by the study staff at pre-vaccination and 30 minutes after vaccination (at least) and the results will be recorded in CRF.

9.5.2 Electrocardiogram(ECG)

A 12-lead electrocardiogram (ECG) will be performed by the investigator or a suitable qualified designee after measurement of vital signs. The ECG measurement should be obtained after the subject has been seated and at rest for at least 5 minutes. The ECGs will be performed at screening visit.

Qualified physicians will interpret the ECG. Any abnormal ECG reading should be noted and recorded in the CRF.

9.5.3 Clinical Laboratory Evaluation

The clinical laboratory analyses will be performed at central laboratory designated by sponsor. Reference ranges will be supplied by the central laboratory and used by the investigator to assess the laboratory data for clinical significance and pathological changes.

Methods and timing for assessing, recording and analyzing each laboratory variable should follow local guidelines. The following laboratory safety tests will be performed:

Blood Routine

CBC, including Hgb, Hct, RBC, WBC with differential, platelet count.

Biochemistry

Creatinine, ALT, AST, total and direct bilirubin; high sensitivity (hs) CRP,

Immunology

Anti-nuclear antibody (ANA)

Urinalysis

Color, **pH,** specific gravity, appearance, nitrate, blood, glucose, ketones, protein, leukocyte esterase, WBC, RBC, casts, epithelial cells, yeast, bacteria

Proteinuria will be checked via urine routine as baseline on Visit 1. **If subject encountered ≥ Grade 3 hypertension, the existence or deterioration of proteinuria or other renal damage sign will be checked.**

9.5.4 Pregnancy Test

Screening for urine pregnancy, using pregnancy strip, will be performed on Day 1 and on Day 29 before vaccination for WoCBP only. It is not required for postmenopausal or surgically sterilized women.

A positive urine pregnancy test should be confirmed by a serum test. Pregnancy tests may be performed more frequently per request of Institutional Review Board (IRB)/Independent Ethics Committee (IEC) or if required by local regulations.

9.5.5 Self-Evaluation/Reporting (Solicited/Unsolicited Symptoms)

9.5.5.1 Solicited Symptoms

Information of solicited symptoms and body temperature will be collected by the subjects in the provided e-diary cards during a 7-day follow-up period after each vaccination (i.e. day of vaccination and 6 subsequent days), and reported by the investigator team. Meanwhile, skin allergic reaction should be monitored for 14 days via e diary. The subject should complete the assessments in the e-diary every evening.

Local reaction, skin allergic reaction, and systemic event are the one whose nature or intensity is consistent with the expected AEs described and listed below.

Table 9-1 Grading for local reaction

Local Reaction	Mild (Grade 1)	Moderate (Grade 2)	Severe (Grade 3)	Potentially Life Threatening (Grade 4)
Pain at injection site	Does not interfere with activity	Interferes with activity	Prevents daily activity	Emergency room visit or hospitalization for severe pain
Itching at injection site	Does not interfere with activity	Interferes with activity	Prevents daily activity	**Emergency room visit or hospitalization for severe symptoms**
Redness	>2.0 to 5.0 cm (5 to 10 measuring device units)	>5.0 to 10.0 cm (11 to 20 measuring device units)	>10 cm (≥21 measuring device units)	Necrosis or exfoliative dermatitis
Swelling	>2.0 to 5.0 cm (5 to 10 measuring device units)	>5.0 to 10.0 cm (11 to 20 measuring device units)	>10 cm (≥21 measuring device units)	Necrosis or exfoliative dermatitis

Table 9-2 Grading for allergic skin reaction

Local Reaction	Mild (Grade 1)	Moderate (Grade 2)	Severe (Grade 3)	Potentially Life Threatening (Grade 4)
Skin allergic reaction	Localized urticaria (wheals) with no medical intervention indicated	Localized urticaria with intervention indicated OR Mild angioedema with no medical intervention indicated	Generalized urticarial OR Angioedema with intervention indicated	Anaphylaxis

Table 9-3 Grading for systemic events

Systemic Event	Mild (Grade 1)	Moderate (Grade 2)	Severe (Grade 3)	Potentially Life Threatening (Grade 4)
Subjective fever	Does not interfere with activity	Some interference with activity	Prevents daily routine activity	**Emergency room visit or hospitalization for severe symptoms**
Chills	Does not interfere with activity	Some interference with activity	Prevents daily routine activity	Emergency room visit or hospitalization for severe chills
Nausea/anorexia	Does not interfere with activity	Some interference with activity	Prevents daily routine activity	**Emergency room visit or hospitalization for severe symptoms**
Vomiting	1 to 2 times in 24 hours	>2 times in 24 hours	Requires intravenous hydration	Emergency room visit or hospitalization for hypotensive shock
Diarrhea	2 to 3 loose stools in 24 hours	4 to 5 loose stools in 24 hours	6 or more loose stools in 24 hours	Emergency room visit or hospitalization for severe diarrhea
Headache	Does not interfere with activity	Some interference with activity	Prevents daily routine activity	Emergency room visit or hospitalization for severe headache
Fatigue/tiredness	Does not interfere with activity	Some interference with activity	Prevents daily routine activity	Emergency room visit or hospitalization for severe fatigue
Muscle pain	Does not interfere with activity	Some interference with activity	Prevents daily routine activity	Emergency room visit or hospitalization for severe new or

Systemic Event	Mild (Grade 1)	Moderate (Grade 2)	Severe (Grade 3)	Potentially Life Threatening (Grade 4)
				worsened muscle pain
Joint pain	Does not interfere with activity	Some interference with activity	Prevents daily routine activity	Emergency room visit or hospitalization for severe new or worsened joint pain

The axillary temperature will be monitored every evening on day of vaccination and 6 subsequent days. If axillary temperature has been measured for more than one time, only the highest degree level should be recorded in the e-diary card.

Table 9-4 Grading for axillary temperature

	Mild (Grade 1)	Moderate (Grade 2)	Severe (Grade 3)	Potentially Life Threatening (Grade 4)
Fever (℃)	37.3～＜38.0	38.0～＜38.5	38.5～＜39.5	≥39.5, last more than 3 days

9.5.5.2 Unsolicited Adverse Event

An unsolicited local and general AE or symptoms are the one whose nature or intensity is NOT consistent with the expected AEs described and listed above in this protocol. Information of unsolicited symptoms/AEs will be collected during study period and reported at each visit or safety calls.

9.5.5.3 Time Period and Frequency for Collecting AE and SAE Information

The time period for actively eliciting and collecting AEs and SAEs ("active collection period") for each participant begins from the time the participant receiving vaccination. All AEs, including AESI, MAAE and SAE, will be collected during the study period.

Follow-up by the investigator continues throughout and after the active collection period and until the AE or SAE or its sequelae resolves or stabilizes at a level acceptable to the investigator.

If the participant withdraws from the study and also withdraws consent for the collection of future information, the active collection period ends when consent is withdrawn.

If a participant definitively discontinues or temporarily discontinues study intervention because of an AE or SAE, the AE or SAE must be recorded on the CRF and the SAE reported using the SAE reporting form.

9.5.6 Adverse events (AEs)

9.5.6.1 Definition of Adverse Events (AE)

An AE is any untoward medical occurrence in a patient or clinical investigation subject temporally associated with the use of a medicinal product, whether or not considered related to the medicinal product. The occurrence does not necessarily have to have a causal relationship with this treatment. An AE can therefore be any unfavorable and unintended sign (including an abnormal laboratory finding), symptom, or disease (new or exacerbated) temporally associated with the use of a medicinal product, whether or not considered related to the medicinal product.

Examples of an AE include:

- Exacerbation of a chronic or intermittent pre-existing condition, including either an increase in frequency and/or intensity of the condition
- **A new condition detected or diagnosed, even though it may have been present prior to this**
- Signs, symptoms, or the clinical sequelae of a suspected interaction
- Signs, symptoms, or the clinical sequelae of a suspected overdose of either study drug or a concurrent medication.

Examples of an AE do NOT include:

- A medical or surgical procedure (*e.g.* endoscopy); the condition that leads to the procedure is an AE
- Situations where an untoward medical occurrence did not occur (social and/or convenience admission to hospital)
- Anticipated day-to-day fluctuations of pre-existing disease(s) or condition(s) present or detected before informed consent is obtained, that do not worsen.

A priori, immunogenicity endpoints as specified in the protocol will not be considered as AEs except if, because of the course or severity or any other features of such events, the investigator, according to his/her best medical judgment, considers these events as exceptional in this medical condition.

9.5.6.2 Definition of Serious Adverse Events (SAE)

A SAE is any untoward medical occurrence that at any dose:

- Results in death or,
- Is life-threatening or,

  Note: The term "life-threatening" in the definition of "serious" refers to an event in which the subject was at risk of death at the time of the event; it does not refer to an event, which hypothetically might have caused death if it were more severe.

- Requires inpatient hospitalization or prolongation of existing hospitalization or,

  Note: In general, hospitalization signifies that the subject has been detained (usually involving at least an overnight stay) at the hospital or emergency ward for observation and/or treatment that would not have been appropriate in the physician's office or out-subject setting. Complications that occur during hospitalization are AEs. If a complication prolongs

hospitalization or fulfils any other serious criteria, the event is serious. When in doubt as to whether hospitalization occurred or was necessary, the AE should be considered serious. Hospitalization for elective treatment of a pre-existing condition that did not worsen from baseline is not considered an AE.

- Results in persistent or significant disability/incapacity or,

  Note: The term disability means a substantial disruption of a person's ability to conduct normal life functions. This definition is not intended to include experiences of relatively minor medical significance such as uncomplicated headache, nausea, vomiting, diarrhoea, influenza, and accidental trauma (e.g. sprained ankle), which may interfere or prevent everyday life functions but do not constitute a substantial disruption.

- Is a congenital anomaly/birth defect or,
- Is a medically important event

  Note: Medical and scientific judgment should be exercised in deciding whether expedited reporting is appropriate in other situations, such as important medical events that may not be immediately life threatening or result in death or hospitalization but may jeopardize the subject or may require intervention to prevent one of the other outcomes listed in the definition above. These should also be considered serious. Examples of such events are invasive or malignant cancers, intensive treatment in an emergency room or at home for allergic bronchospasm, blood dyscrasias or convulsions that do not result in hospitalisation, or development of drug dependency or drug abuse.

An AE fulfilling any one or more of these criteria should be reported as a SAE, irrespective of the dose of drug given, and even if it is the result of an interaction or drug abuse.

A distinction should be drawn between serious and severe AEs. The term 'severe' is often used to describe the intensity (severity) of a specific event (as in mild, moderate, or severe myocardial infarction); the event itself, however, may be of relatively minor medical significance (such as severe headache). This is not the same as 'serious,' which is based on subject's event outcome or action criteria usually associated with events that pose a threat to a subject's life or functioning. The seriousness (not severity) serves as a guide for defining regulatory reporting obligations.

9.5.6.3 Definition of Unexpected Adverse Reaction Definition

An unexpected adverse reaction is any untoward and unintended response that is related to the administration of the study agent, at any dose that is not consistent with the applicable product information (e.g., current version of the Investigator's Brochure for an unauthorized investigational medicinal product or summary of product characteristics for an authorized product).

9.5.6.4 Definition of Medically Attended Adverse Event

A Medically Attended Adverse Event (MAAE) is an AE with a medically-attended visit that is not a routine visit for physical examination or vaccination, such as visits for hospitalization, an emergency room visit, or an otherwise unscheduled visit to or from medical personnel (medical doctor) for any reason.

9.5.6.5 Definition of Adverse Events of Special Interest (AESI)

Adverse event of special interest (AESI) is further defined in Council for International Organizations of Medical Sciences (CIOMS) VII as [15]:

An adverse event of special interest (serious or non-serious) is one of scientific and medical concern specific to the sponsor's product or specific target disease, for which ongoing monitoring and rapid communication by the investigator to the sponsor could be appropriate. Such an event might require further investigation in order to characterize and understand it. Depending on the nature of the event, rapid communication by the trial sponsor to other parties (e.g., regulators) might also be warranted. Thus AESI should include potential immune medical condition (listed in **Table 9-6**), or any newly identified potential AESI followed through 12 months after participants' final vaccination. Complications of COVID-19 (listed in **Table 9-5**) should be considered and reported as AESIs.

Subjects will be assessed for diagnosis of an AESI at all visit. AESIs include potential ADE medical conditions, AE specific to complications of COVID-19, or other potential AEs that may be determined at any time by regulatory authorities as additional information concerning COVID-19 is obtained. Given the concern for "cytokine storm", an AESI of cytokine release syndrome will be included as an AE specific to COVID-19. Thus AESI included

AESIs specific to complications of potential disease enhancement for COVID-19, also termed ADE (antibody dependent enhancement), will also be monitored (see **Table 9-5**).

Table 9-5 Adverse Events of Special Interest Relevant to COVID-19a

Body System	Diagnoses[a]
Immunologic	Enhanced disease following immunisation,[b] cytokine release syndrome related to COVID-19[c], Multisystem inflammatory syndrome in children (MIS-C)
Respiratory	Acute respiratory distress syndrome (ARDS)
Cardiac	Acute cardiac injury including:<br>• Microangiopathy<br>• Heart failure and cardiogenic shock<br>• Stress cardiomyopathy<br>• Coronary artery disease<br>• Arrhythmia<br>• Myocarditis, pericarditis
Hematologic	Coagulation disorder<br>• Deep vein thrombosis<br>• Pulmonary embolus<br>• Cerebrovascular stroke<br>• Limb ischemia<br>• Hemorrhagic disease<br>• Thrombotic complications
Renal	Acute kidney injury
Gastrointestinal	Liver injury
Neurologic	Guillain-Barré Syndrome, anosmia, ageusia, meningoencephalitis
Dermatologic	Chilblain-like lesions, single organ cutaneous vasculitis, erythema multiforme

Abbreviations: AESI = adverse event of special interest; COVID-19 = coronavirus disease 2019; DAIDS = Division of AIDS; PCR = polymerase chain reaction; SARS-CoV2 = severe acute respiratory syndrome coronavirus 2.

[a] To be recorded as AESIs relevant to COVID-19, these complications should be associated with a positive PCR test for SARS-CoV-2.

[b] COVID-19 manifestations associated with more severe presentation and decompensation with consideration of enhanced disease potential.

[c] Cytokine release syndrome related to COVID-19 infection is a disorder characterised by nausea, headache, tachycardia, hypotension, rash, and/or shortness of breath.

Because it has been hypothesized that immunizations with or without adjuvant may be associated with autoimmunity, regulatory authorities have requested that sponsor instruct investigators to be especially vigilant regarding the PIMMC listed below (**Table 9-6**). Subjects encountered the medical condition of AESI listed in **Table 9-5 and 9-6** should check HLA and SARS-CoV-2 infection,

Table 9-6 Potential Immune-Mediated Medical Conditions (PIMMC)

**Categories**	**Diagnoses (as MedDRA Preferred Terms)**
Neuro-inflammatory Disroders	Acute disseminated encephalomyelitis (including site specific variants: e.g. Non-infectious encephalitis, encephalomyelitis, myelitis, myeloradiculomyelitis), cranial nerve disorders including paralyses/paresis (e.g. Bell's palsy), generalized convulsion, Guillain-Barré syndrome (including Miller Fisher syndrome and other variants), immune-mediated peripheral neuropathies and plexopathies (including chronic inflammatory demyelination polyneuropathy, multi focal motor neuropathy and polyneurapthies associated with monoclonal gammopathy), myasthenia graves, multiple sclerosis, nacrolepsy, optic neuritis, transverse myelitis, uveitis.
Musculoskeletal and Connective Tissue Disorder	Antisynthetase syndrome, dermatomyositis, juvenile chronic arthritis (including Still's disease), mixed connective tissue disorder, poly myalgia rheumatic, polymyositis, psoriatic arthropod hypertension, relapsing polychondritis, rheumatoid arthritis, scleroderma (including diffuse systemic form and CREST syndrome), spondyloarthritis (including ankylosis spondylitis, reactive arthritis (Reiter's Syndrome) and undifferentiated spondyloarthritis), systemic lupus erythematosus, systemic sclerosis, Sjogren's syndrome.
Vasculitides	Large vessels vasculitis (including giant cell arthritis such as Takayasu's arthritis and temporal arthritis), medium sized and /or small vessels vasculitis (including poly arthritis no dose, Kawasaki's disease, microscopic polyangitis, Wegener's granule atoms is, Churg-Strauss syndrome (allergic granulomatous angiitis), Buerger's disease (thromboangiitis obliterans), necrotizing vasculitis and ANCA-positive vasculitis (type unspecified), Henoch-Schonlein purpura, Bechet's syndrome, leukocytoclastic vasculitis)
Gastrointestinal Disorders	Crohn's disease, celiac disease, ulcerative colitis, ulcerative procitis.
Hepatic Disorders	Autoimmune hepatitis, autoimmune cholangitis, primary sclerosis cholangitis, primary biliary cirrhosis
Renal Disorders	Autoimmune glomerulonephritis (including IgA nephropathy, glomerulonephritis rapidly progressive, membranous glomerulonephritis, membranoproliferative glomerulonephritis, and mesangioproliferative glomerulonephritis)
Cardiac Disorders	Autoimmune myocarditis/cardiomyopathy

Skin Disorders	Alopecia areata, psoriasis, vitiligo, Raynaud's phenomenon, erythema no do sun, autoimmune bulbous skin disease (including pemphigus, pemphigoid and dermatitis herpetiformis), cutaneous lupus erythematosus, morphed, lichen plants, Stevens-Johnson syndrome, Sweet's syndrome
Hematologic Disorders	Autoimmune hemolytic anemia, autoimmune thrombocytopenia, antiphospholipid syndrome, thrombocytopenia
Metabolic Disorders	Autoimmune thyroiditis, Grave's or Basedow's disease, new onset Hashimoto thyroiditis, diabetes mellitus type 1, Addison's disease
Other Disorders	Goodpasture syndrome, idiopathic pulmonary fibrosis, pernicious anemia, sarcoidosis.

Abbreviations: ANCA: anti-neutrophil cytoplasmic antibody, IgA: immunoglobulin A.

9.5.6.6 Assessment of Severity

All AEs, except AEs in e-diary card, will be assessed according to the US NCI Common Terminology Criteria for Adverse Events (CTCAE) 5.0 (published on November 27, 2017) associated with the AE term. The following standard with 5 grades is to be used to measure the severity of adverse events in this study.

Table 9-7 Intensity scales of AE

Grades of AE	Description
Grade 1	Mild; asymptomatic or mild symptoms; clinical or diagnostic observations only; intervention not indicated.
Grade 2	Moderate; minimal, local or noninvasive intervention indicated; limiting age-appropriate instrumental ADL*
Grade 3	Severe or medically significant but not immediately life-threatening; hospitalization or prolongation of hospitalization indicated; disabling, limiting self care ADL**
Grade 4	Life-threatening consequences; urgent intervention indicated.
Grade 5	Death related to AE.

* Instrumental activities of daily living (ADL) refer to preparing meals, shopping for groceries or clothes, using the telephone, managing money, etc.
** Self-care ADL refer to bathing, dressing and undressing, feeding self, using the toilet, taking medication, and not bedridden.

9.5.6.7 The Relationship to Study Vaccine

The investigator will make an assessment of the relationship between investigational vaccine and the occurrence of each AE/SAE, except solicited reactions to vaccination. The reasonable possibility will be determined based on the investigator's clinical judgment. The causality should be considered as one of the categories described below.

Table 9-8 The relationship between AE and study vaccine

Causality term	Assessment criteria
Certain	• Event or laboratory test abnormality, with plausible time relationship to drug intake<br>• Cannot be explained by disease or other drugs<br>• Response to withdrawal plausible (pharmacologically, pathologically)<br>• Event definitive pharmacologically or phenomenologically (i.e. an objective and specific medical disorder or a recognized pharmacological phenomenon)<br>• Rechallenge satisfactory, if necessary
Probable / Likely	• Event or laboratory test abnormality, with reasonable time relationship to drug intake<br>• Unlikely to be attributed to disease or other drugs<br>• Response to withdrawal clinically reasonable<br>• Rechallenge not required
Possible	• Event or laboratory test abnormality, with reasonable time relationship to drug intake<br>• Could also be explained by disease or other drugs<br>• Information on drug withdrawal may be lacking or unclear
Unlikely	• Event or laboratory test abnormality, with a time to drug intake that makes a relationship improbable (but not impossible)<br>• Disease or other drugs provide plausible explanations
Unrelated	• Occurred before dosing<br>• Due wholly to factors other than study treatment

Each event should be followed until resolution or the event is considered stable. Both regular return and telephone contact will be acceptable.

9.5.6.8 Adverse Events Reporting

Documentation and Reporting of Adverse Events

All investigators should follow up subjects with AEs until the event is resolved or until, in the opinion of the investigator, the event is stabilized or determined to be chronic. Details of AE resolution must be documented in the CRF.

All AEs should be reported and documented in accordance with the procedures outlined in this section. All AEs occurring during the study must be documented on the relevant CRF pages.

Reporting of Serious Adverse Events

Any SAE must be reported by the investigator if it occurs during the clinical study, whether or not the SAE is considered to be related to the investigational product. An SAE report consists of the SAE form, the AE form, and the concomitant medication form. A copy of these forms must be emailed **within 24 hours** to Contract Research Organization (CRO), StatPlus Inc., and StatPlus will inform United Biomedical, Inc., Asia on the same day.

The investigator should not wait to receive additional information to document fully the event before notification of a SAE, though additional information may be requested. Where applicable, information from relevant laboratory results, hospital case records, and autopsy reports should be obtained.

Instances of death, congenital abnormality, or an event that is of such clinical concern as to influence the overall assessment of safety, if brought to the attention of the investigator at any time after cessation of study agent administration and linked by the investigator to this study, should be reported to the study monitor.

The sponsor and/or the appointed representative(s) will promptly notify all relevant investigators and the regulatory authorities of findings that could adversely affect the safety of subjects, impact on the conduct of the study, or alter the IEC/ IRB approval/favorable opinion of the study. In addition, the sponsor and/or the appointed representative(s), will expedite the reporting to all concerned investigators, to the IEC(s)/IRB(s), where required, and to the regulatory authorities of all adverse reactions that are both serious and unexpected.

Details of the procedures to be followed if a pregnancy occurs are also provided in Section 7.4.8.4.

Documentation and Reporting of SUSARs

All suspected unexpected serious adverse reactions (SUSARs) will be the subject of expedited reporting. The sponsor and/or the appointed representative(s) shall ensure that all relevant information about a SUSAR that is fatal or life-threatening is reported to the relevant competent authorities and IEC/IRB within 7 days after knowledge by the sponsor of such a case and that relevant follow-up information is communicated within an additional 8 days. All other SUSARs will be reported to the relevant competent authorities and IEC/IRB within 15 days after knowledge by the sponsor of such a case. All investigators should follow up SUSARs until the event is resolved or until, in the opinion of the investigator, the event is stabilized or determined to be chronic. Poststudy SUSARs that occur after the subject has completed the clinical study must be reported by the investigator to the sponsor.

<u>Documentation and Reporting of SARS-CoV-2 infection</u>

All subjects will be surveilled for potential COVID-19 illness from Visit 1 onwards. Potential COVID-19 illnesses and their sequelae that are consistent with the clinical endpoint definition should not be recorded as AEs or SAEs, even though the event may meet the definition of an SAE. These data will be captured as efficacy assessment data only on the relevant pages of the CRF, as these are expected endpoints.

Potential COVID-19 illness events and their sequelae will be reviewed by IDMC. Any SAE that is determined by the IDMC not to meet endpoint criteria is reported back to the SAE reporting process. The investigator's SAE awareness date is the date on which events is confirmed not to meet endpoint criteria.

9.5.7 Immunogenicity Assessments

9.5.7.1 Surveillance and Laboratory Diagnosis of SARS-CoV-2 Infection during Clinical Trial

During the observation period of the clinical trial, the participants with fever, cough and other respiratory symptoms should immediately go to the designated hospital. The doctor or investigator may collect the nasopharyngeal/throat swabs and to perform CT and other imaging examinations to analyse whether it is caused by SARS-CoV-2 infection. In the event of SARS-CoV-2's infection during the clinical trial, it is necessary to conduct a case investigation, and the critically ill or dead cases need to continue to conduct a special investigation, mainly to analyze whether there is an ADE or VAERD phenomenon. Subjects encountered the medical condition listed in Table 9-5 should also check HLA and SARS-CoV-2 infection,

In addition to SARS-CoV-2 nucleic acid detection, multiple pathogens will be detected for differential diagnosis of swabs. The detail of detectable pathogens of test kit will be listed below:

- ✓ Adenovirus
- ✓ Coronavirus 229E
- ✓ Coronavirus HKU1
- ✓ Coronavirus NL63
- ✓ Coronavirus OC43
- ✓ Human Metapneumovirus
- ✓ Human Rhinovirus/Enterovirus
- ✓ Influenza virus A
- ✓ Influenza virus B
- ✓ Parainfluenza virus 1
- ✓ Parainfluenza virus 2
- ✓ Parainfluenza virus 3
- ✓ Parainfluenza virus 4

- ✓ Respiratory syncytial virus
- ✓ Bordetella parapertussis
- ✓ Bordetella pertussis
- ✓ Chlamydia pneumoniae
- ✓ Mycoplasma pneumoniae

The potential SARS-CoV-2 infection will be reviewed by IDMC.

9.5.7.2 Detection of ELISA Antibodies against S1-RBD of SARS-CoV-2

Anti-S1-RBD antibody titers will be measured by ELISA kit. A dilution microplate is used to prepare the serum dilutions per SOP for test serum samples. Anti-S1-RBD antibody level is expressed as linear titers of an end point dilution for a test sample. SoftMax Titer Calculation Program (Molecular Devices Co.) is used to calculate the titers. For seroconversion detected by UBI SARS-CoV2 S1-RBD ELISA, it is defined as a 4-fold increase in antibody titer from baseline.

9.5.7.3 Detection of Antibody Titers Which Inhibit S1-RBD:ACE2 binding

Antibody titers for ability to inhibit S1-RBD:ACE2 binding will be measured by ELISA kit. A dilution microplate is used to prepare the serum dilutions per SOP. Antibody levels which inhibit S1-RBD:ACE2 binding are expressed in μg/mL as titers for a test sample. SoftMax Titer Calculation Program (Molecular Devices Co.) is used to calculate the titers. Specimens that do not react in the test are considered with < 1.6 μg/mL titer in this SARS-CoV-2 qNeu Ab ELISA.

9.5.7.4 Neutralizing Antibody Titers against SARS-CoV-2

Neutralizing antibody titers will be measured by CPE-based live virus neutralization assay using Vero-E6 cells infected with SARS-CoV-2. The study will be conducted in the BSL-3 lab at Academia Sinica, Taipei. The levels of SARS-CoV-2 virus specific neutralization titers are measured based on the principle of VNT_{50} titer (≥ 50% reduction of virus-induced cytopathic effects). Virus neutralization titer of a serum specimen is defined as the reciprocal of the highest serum dilution at which 50% reduction in cytopathic effects are observed and results are calculated by the method of Reed and Muench. For seroconversion detected by live virus neutralization test, it is defined as a 4-fold increase in antibody titer from baseline.

9.5.7.5 Detection of antibody against SARS-CoV-2 antigens

Specific IgG antibodies against SARS-CoV-2 antigens derived from S2, N, and M proteins in the serum of subjects will be tested by UBI SARS-CoV-2 ELISA (antigen combinations) and Confirmatory SARS-CoV-2 ELISA (individual antigens). These ELISA tests can distinguish immune responses in infected versus vaccinated individuals.

9.5.7.6 Detection of T Cell Response

Human peripheral blood mononuclear cells will be used for evaluating vaccine-induced T cell responses. Antigen-specific interferon-gamma (IFN-γ) and IL-4 production will be measured by

ELISpot. Intracellular cytokine staining and flow cytometry will be used to evaluate $CD4^+$ and $CD8^+$ T cell responses. This assessment will be optional.

9.5.8 Communication and Use of Technology

This study may employ various methods, such as e-dairy, telephone, or other communication pathway, to contact with participants for record and maintain the study information. The communication items include but not limited to:

- Record 7-day solicited symptoms and 14-day skin allergic reaction after vaccination in reactogenicity e-diary
- Record unsolicited AEs or/and symptoms of COVID-19 on Day 8, Day 15, Day 22, Day 36, Day 43, Day 64, Day 71, Day 78, Day 85**, the day on 7 days after Visit 6 (only for subjects receiving 3rd dose),** Day 253, and Day 309 using telephone communication
- Record regular surveillance for COVID-19 illness in e-dairy. A prompt on their smartphone device to regularly surveillance for signs and symptoms of COVID-19 every week after Day 57. If any potential symptoms for COVID-19 illness is reported, a telephone call will be made between site staffs and subjects.
- Unscheduled visit set-up or visit reminders

9.5.9 Appropriateness of Measurements

The immunogenicity and safety assessments planned for this study are generally recognized as reliable, accurate, and relevant to the diagnostic modality and underlying disease/condition.

9.6 Independent Data Monitoring Committee (IDMC)

The Independent Data Monitoring Committee (IDMC) will act in an expert, independent advisory capacity to monitor participant safety and evaluate the efficacy of UB-612 vaccine against COVD-19 in this study. The IDMC will consist of at least two physicians and one statistician. An IDMC meeting will be held at:

(1) 1st time: when all adult immunogenicity data (350 evaluable young adults and 154 evaluable elderly subjects) for Day 57 are available
(2) 2nd time: when at least half of core group subjects will be completed Day 85 safety follow up. Adolescence could be enrolled after 2nd IDMC meeting.
(3) 3rd time: when all immunogenicity data for lot-to lot consistency of Day 57 are available.
(4) 4th time: when 350 evaluable adolescents will be completed Day 57 safety follow up.

The meeting schedule might be adjusted based on the progress of recruitment. The IDMC meetings may also be held at any time point if any situations after vaccination meet the study stopping criteria or when the sponsor considers it necessary. Unscheduled IDMC meetings may also be held if any potential COVID-19 illness events or obvious situations for the deterioration the subject safety or study conduct occur.

Responsibilities of the IDMC include the following:

✓ Protect the safety and confidentiality of the study participants;

- ✓ Evaluate the quality and validity of the data generated in this study;
- ✓ Consider factors external to the study when relevant information becomes available, such as scientific or therapeutic developments that may have an impact on the safety of the participants or the ethics of the trial;
- ✓ Make recommendations to the Sponsor and the Principal Investigator (PI) concerning continuation, termination or other modifications of the study based on factors including overwhelming effectiveness, futility or safety issues;
- ✓ Make recommendations and assist in the resolution of problems reported by the PI;
- ✓ Assist sponsor by commenting on any problems with study conduct and/or data collection;
- ✓ Maintain confidentiality regarding the study outside the IDMC (including, but not limited to the investigators, IRBs, regulatory agencies, or sponsor) except as authorized by the IDMC.

Detailed description of the IDMC responsibilities, data reviewed, meeting process, considerations and policies will be described in a separate IDMC charter.

9.6.1 Overall Study Stopping Rules

The following stopping rules are in place for all subjects, based on review of AE data and e-diary reactogenicity data.

1. An SAE that is assessed by the investigator as possibly related, or for which there is no alternative, plausible, attributable cause.
2. A Grade 4 local reaction, systemic adverse event or fever that is assessed as possibly related by the investigator, or for which there is no alternative, plausible, attributable cause.
3. If any 3 subjects vaccinated with UB-612 (at any dose level) report the same or similar* severe (Grade 3) AE (including laboratory abnormalities), assessed as possibly related by the investigator, or for which there is no alternative, plausible, attributable cause.

   * defined as same preferred term of current MedDRA
4. If a stopping rule is met, further dosing will be paused and the IDMC will determine whether dosing should resume.
5. If two of the same or similar *SAEs or Grade 4 local or systemic reactions occur, dosing will be suspended and both the IDMC and regulatory authorities will be consulted before resumption of dosing.

   * defined as same preferred term of current MedDRA

In the event that a stopping rule is met, the following actions will be taken:

- The stopping rule will PAUSE randomization and administration of study vaccine at all dose levels and all treatment groups.
- The IDMC will review the relevant safety data.

- For all subjects already vaccinated, all other routine study conduct activities, including ongoing data entry, reporting of AEs, electronic diary completion, blood sample collection, and subject follow-up, will continue during the pause.
- Data from placebo recipients will not contribute to the stopping rules.
- Electronic diary data confirmed by the investigator as having been entered in error will not contribute toward a stopping rule.

Should the study be terminated prematurely, the sponsor will provide written notification to all investigators and regulatory authorities specifying the reason(s) for early termination. The investigator must inform the institutional review board (IRB)/independent ethics committees (IEC) promptly and provide the reason(s) for the termination. Previously dosed subjects will be assessed through all planned study safety visits.

10 STATISTICAL METHODS

The statistical planning and analysis of the trial will be performed by the designated contract research organization.

10.1 Statistical and Analytical Plans

A statistical analysis plan will be prepared and finalized prior to database lock of the study. The statistical analysis plan will include full details of all planned statistical analyses.

10.2 Sample Size Determination

Consider a 10% drop-out rate, around 3850 adult subjects in core group will be recruited and randomized to UB-612 vaccine 100 µg or placebo with 6:1 allocation rate and around 385 adolescents will be recruited for supplementary group and randomized to UB-612 vaccine 100 µg or placebo with same allocation ratio.

10.2.1 Sample Size for Safety Evaluation

For safety outcomes, the following table shows the probability of observing at least 1 SAE for a given true event rate of a particular SAE. For example, if the true SAE rate is 0.01%, with 2400 adult subjects, 600 elderly subjects, and 300 adolescent subjects received select vaccine dose, there are 21.3%, 5.8%, and 3.0% probabilities of observing at least 1 SAE. Overall, probability of observing at least 1 SAE is 25.9% with true SAE rate of 0.01% for all 3000 subjects received study vaccine in safety group.

True SAE Rate	N=300	N=600	N=2400	N=3000
0.001%	0.003	0.006	0.024	0.030
0.010%	0.030	0.058	0.213	0.259
0.100%	0.259	0.451	0.909	0.950

10.2.2 Sample Size for Lot-to-Lot Consistency

The sample size is driven by the objective to demonstrate the consistency of GMT ratio (GMTR) with the 3 consecutive manufacturing lots of the UB-612 vaccine. The clinical lot-to-lot consistency will be tested for the three pair-wise comparisons by computing the two-sided 95% CI on the GMTR. If all confidence intervals are within the pre-defined clinical limits of [0.5, 2.0], one can conclude that the lots are consistent.

Assume the expected GMTR of 1 and a value of 0.6 for the standard deviation (SD) of the decimal logarithmic transformation (log base 10) of antibody titers (log10(GSD) is about 0.2 to 0.6; refer to following table of the V-122 interim result). In order to have at least 90% power to achieve the lot-to-lot consistency, the estimated sample size should ensure beta less than 3.3% (Bonferroni adjustment of beta for 3 comparisons between 3 lots). With the parameters above it estimates a minimum evaluable sample size per lot of 115 subjects. Consider a 10% drop-out rate, a total of 396 subjects (132 subjects per lot) will be needed for ensuring overall lot-to-lot consistency with at least 90% overall power.

Table GSD and Logarithmic Transformation of Neutralizing Antibody Titers and Anti-S1-RBD Antibody Titers at Day 56

Antibody Titers	**UB-612 10 µg**		**UB-612 30 µg**		**UB-612 100 µg**	
	GSD	log10(GSD)	GSD	log10(GSD)	GSD	log10(GSD)
Neutralizing	3.286	0.52	2.438	0.39	1.670	0.223
Anti-S1-RBD	4.185	0.62	3.112	0.49	1.436	0.16

Note: Only first six subjects in **100 µg** group had neutralizing antibody titer results at Day 56 until 2021-01-12

10.3 Analysis Populations

Enrolled Population

Enrolled Population includes subjects who have a signed ICF.

Randomized Population

Subjects who are assigned a random number be regarded as Randomized Population.

Evaluable Immunogenicity Population

Evaluable Immunogenicity Population will consist of all eligible randomized subjects who are assigned to **lot-to-lot and** immunogenicity group, receive two vaccinations within the predefined window, have a valid immunogenicity result at visit 4 (Day 57), have no major protocol deviations or protocol deviations having impact on immunogenicity data. Evaluable Immunogenicity Population will be regarded as primary population for immunogenicity evaluation on primary and secondary immunogenicity endpoints except lot-to-lot consistency.

Evaluable Lot-to-Lot Population

Evaluable Lot-to-Lot Population is a subset of Evaluable Immunogenicity but only includes subjects who are assigned to lot-to-lot consistency group and have immunogenicity determination at Day 57. Evaluable Lot-to-Lot Population will be used for evaluating lot-to-lot consistency only.

Evaluable Efficacy Population

All eligible randomized subjects who receive two vaccinations within the predefined window and have no major protocol deviations or protocol deviations **having impact on immunogenicity data** will be the Evaluable Efficacy Population. Evaluable Efficacy Population will be used to evaluate vaccine efficacy in exploratory analysis.

Available Immunogenicity Population

Available Immunogenicity Population will consist of all eligible randomized subjects who receive at least one vaccination, and have at least one post immunogenicity data determination. Available Immunogenicity Population will also be used to the immunogenicity evaluation except lot-to-lot consistency.

Evaluable Booster Population

All eligible randomized subjects who receive third dose of UB-612 within the predefined window and have no major protocol deviations or protocol deviations having impact on immunogenicity data will be the Evaluable Booster Population. Evaluable Booster

Population will be used to explore the immunogenicity evaluation after the booster vaccination.

Safety Population

Safety Population (SAF) will consist of all subjects who received at least one vaccination. The Safety Population is for safety evaluation in analysis.

10.4 Demographic and Other Baseline Characteristics

Demographic and baseline characteristic data will be summarized for each study group and overall. Descriptive statistics (N, mean, standard deviation, median, minimum, and maximum) will be presented for continuous variables. The number and percentage of subjects in each category will be presented for categorical variables. No formal testing of demographic or baseline characteristics will be performed.

10.5 Safety Evaluation

All safety assessments, including AEs, PEs, VS and clinical laboratory evaluations, where indicated, will be presented using descriptive statistics for each study group of UB-612. Data will be summarized for each study group.

Local Reactions and Systemic Events

Local reactions and systemic events recorded on e-diary will be summarized by the severity grading scales by study groups. Numbers and percentages of subjects experiencing each local reaction/systemic event will be presented for each symptom severity by study groups. Summary tables showing the occurrence of any local reactions and occurrence of any systemic event will also be presented.

Unsolicited Adverse Event

This analysis applies to all unsolicited adverse events occurring during the study, recorded in AE eCRF, with a start date on or after the date of vaccination. Unsolicited AEs occurring during the study will be coded using the most recent version of Medical Dictionary for Regulatory Activities (MedDRA). The adverse events will then be grouped by MedDRA preferred terms into frequency tables according to system organ class.

All reported unsolicited adverse events, as well as adverse events judged by the investigator as at least possibly related to study vaccine, will be summarized according to system organ class and preferred term within system organ class. When an unsolicited adverse event occurs more than once for a subject, the maximal severity and strongest relationship to the study group will be counted.

Separate summaries will be produced for the following categories, such as serious adverse events and unsolicited AEs related to vaccinations will be presented. Data listings of all unsolicited adverse events will be provided by subject.

Physical Examination

All physical examination findings will be listed and summarized by time point and study group. Shift table will be also presented, if appropriate.

Vital Signs

All vital sign findings will be listed and summarized by time point and study group.

Laboratory Evaluations

Laboratory safety data will be analysed descriptively by time point and study group. Shift table for laboratory data will be shown by visit using categorization of laboratory according to local laboratory's normal reference range.

10.5.1 Analysis of Primary Safety Endpoint(s)

- Local reactions for up to 7 days following each dose

  Local reactions for up to 7 days following each dose will be summarized with counts and percentages of participants with the indicated endpoint and the associated Clopper-Pearson 95% CIs by study group and each vaccination. The group comparison will be assessed by Fisher's exact test. Summary of descriptive statistics by age group will be also provided.

- Systemic events for up to 7 days following each dose

  Systemic events for up to 7 days following each dose will be summarized with counts and percentages of participants with the indicated endpoint and the associated Clopper-Pearson 95% CIs by study group and each vaccination. The group comparison will be assessed by Fisher's exact test. Summary of descriptive statistics by age group will be also provided.

- Unsolicited AEs from Day 1 to Day 57

  Unsolicited AEs will be presented by descriptive summary statistics (counts, percentages, and associated Clopper-Pearson 95% CIs) with system organ class and preferred term for each study group. The group comparison will be assessed by Fisher's exact test. Summary of descriptive statistics by age group will be also provided.

- MAAEs and SAEs from Day 1 to Day **365**

  MAAEs and SAEs will be summarized by counts, percentages, and associated Clopper-Pearson 95% CIs for each study group. The group comparison will be assessed by Fisher's exact test. Summary of descriptive statistics by age group will be also provided. **Additionally, the AESIs and ADEs occurred after third vaccination for subjects received three vaccinations will be also summarized separately.**

- AESIs and ADEs from Day 1 to Day 365

  AESIs and ADEs will be summarized by counts, percentages, and associated Clopper-Pearson 95% CIs for each study group. The group comparison will be assessed by Fisher's exact test. Summary of descriptive statistics by age group will be also provided. **Additionally, the AESIs and ADEs occurred after third vaccination for subjects received three vaccinations will be also summarized separately.**

10.5.2 Analysis of Secondary Safety Endpoint

- Changes of safety laboratory measures

Changes of safety laboratory measures will be summarized with descriptive statistics by study group and time point. ANCOVA model with laboratory baseline values as covariate will analyse changes of safety laboratory measures for testing the difference between study groups. Intra-group difference in safety laboratory measures will also be analysed by paired t test. Summary of descriptive statistics by age group will be also performed.

10.6 Immunogenicity Evaluation

10.6.1 Analysis of Primary Immunogenicity Endpoint(s)

- Geometric mean titer (GMT) of SARS-CoV-2 neutralizing antibody on Day 57

  For SARS-CoV-2 neutralizing antibody, GMT and 2-sided 95% CIs will be provided for each study group. Additionally, the difference between UB-612 vaccine and placebo groups will be analysed by ANCOVA model under log-transform data with baseline level as covariate and effect of study treatment, if appropriate. Summary of descriptive statistics by age group will be also performed.

- Seroconversion rate (SCR) of SARS-CoV-2 neutralizing antibody on Day 57

  The SCR will be presented as count and percentage in frequency table, and the 95% exact (Clopper-Pearson) confidence interval will be provided as well by study group. Fisher's exact test will be used for the comparison of UB-612 vaccine group and placebo group. Summary of descriptive statistics by age group will be also performed.

10.6.2 Analysis of Secondary Immunogenicity Endpoint(s)

- Seroconversion rate (SCR) of antigen-specific antibody (Anti-S1-RBD) on Day 57

  The SCR will be presented as count and percentage in frequency table, and the 95% exact (Clopper-Pearson) confidence interval will be provided as well by study group. Fisher's exact test will be used for the comparison of UB-612 vaccine group and placebo group. Summary of descriptive statistics by age group will be also performed.

- Geometric mean titer (GMT) of SARS-CoV-2 neutralizing antibody on Day 197 and 365

  GMT and 2-sided 95% CIs will be provided for each study group. Additionally, the difference between UB-612 vaccine and placebo groups will be analysed by ANCOVA model under log-transform data with baseline level as covariate and effect of study treatment, if appropriate. Summary of descriptive statistics by age group will be also performed.

  In addition, the GMT of SARS-CoV-2 neutralizing antibody on Day 365 for subjects received two vaccinations and three vaccinations should be analysed separately. Analysis of GMT of SARS-CoV-2 neutralizing antibody on Day 365 for subjects received third dose of UB-612 will be performed with the Evaluable Booster Population only.

- Geometric mean titer (GMT) of antigen-specific antibody (Anti-S1-RBD) on Day 57, 197, and 365

GMT and 2-sided 95% CIs will be provided for each study group. Additionally, the difference between UB-612 vaccine and placebo groups will be analysed by ANCOVA model under log-transform data with baseline level as covariate and effect of study treatment, if appropriate. Summary of descriptive statistics by age group will be also performed.

In addition, the GMT of Anti-S1-RBD on Day 365 for subjects received two vaccinations and three vaccinations should be analysed separately. Analysis of GMT of Anti-S1-RBD on Day 365 for subjects received three vaccinations will be performed with the Evaluable Booster Population only.

- Geometric mean fold increase in antigen-specific antibody (Anti-S1-RBD) and SARS-CoV-2 neutralizing antibody on Day 57, 197 and 365

  GMFI and 2-sided 95% CIs will be provided for each study group. Additionally, the difference between UB-612 vaccine and placebo groups will be analysed by Student's t test under log-transform data. Summary of descriptive statistics by age group will be also performed.

  In addition, the GMFI of Anti-S1-RBD on Day 365 for subjects received two vaccinations and three vaccinations should be analysed separately. Analysis of GMFI of Anti-S1-RBD on Day 365 for subjects received third dose of UB-612 will be performed with the Evaluable Booster Population only.

- Lot consistency as assessed by the comparisons of the GMT of SARS-CoV-2 neutralizing antibody on Day 57 induced by 3 independent UB-612 vaccine clinical materials. The 95% confidence intervals between groups will be within the margin of 0.5 to 2.

  For lot consistency, the 1 month after 2nd vaccination, for all pairs of lots, the two sided 95% CIs for the GMT ratios (GMTR) of neutralizing antibody will be calculated. For all pairs of lots, if the two-sided 95% CIs for the GMTR are within the [0.5,2.0] clinical limit interval, lot consistency will be concluded. The GMTR will be calculated as the mean of the difference of decimal logarithmically transformed antibody titers and exponentiating the mean with base 10. The associated 2-sided CIs will be obtained by calculating CIs using Student's t-distribution for the mean difference of the logarithmically transformed antibody titers and exponentiating the confidence limits with base 10.

10.7 Analysis of Exploratory Endpoint(s)

- T cell responses to UB-612 measured by ELISpot and flow cytometric assays on Day 57

  T cell responses to UB-612 measured by ELISpot and flow cytometric assays on Day 57 will be summarized by descriptive statistics.

- **T cell responses to UB-612 measured by ELISpot and flow cytometric assays on 14 days post 3rd dose of UB-612**

  T cell responses to UB-612 measured by ELISpot and flow cytometric assays on 14 days post 3rd dose of UB-612 will be summarized by descriptive statistics.

- **Geometric mean titer (GMT) of SARS-CoV-2 neutralizing antibody on 14 days post 3rd dose of UB-612**

  GMT of SARS-CoV-2 neutralizing antibody on 14 days post 3rd dose of UB-612 and 2-sided 95% CIs will be provided for subjects who received third dose of UB-612. Summary of descriptive statistics by age group will be also performed. Analysis of GMT of SARS-CoV-2 neutralizing antibody on 14 days post 3rd dose of UB-612 for subjects received third dose of UB-612 will be performed with the Evaluable Booster Population only. For subjects received third dose of UB-612, GMT of SARS-CoV-2 neutralizing antibody on 14 days post 3rd dose of UB-612 will compare with GMT of SARS-CoV-2 neutralizing antibody on Visit 6 (pre-3rd dose baseline) by paired t test. This analysis will be performed with the Evaluable Booster Population only.

- **Geometric mean titer (GMT) of antigen-specific antibody (Anti-S1-RBD) on 14 days post 3rd dose of UB-612**

  GMT of Anti-S1-RBD on 14 days post 3rd dose of UB-612 and 2-sided 95% CIs will be provided for subjects who received third dose of UB-612. Summary of descriptive statistics by age group will be also performed. Analysis of GMT of Anti-S1-RBD on 14 days post 3rd dose of UB-612 for subjects received third dose of UB-612 will be performed with the Evaluable Booster Population only. For subjects received third dose of UB-612, GMT of Anti-S1-RBD on 14 days post 3rd dose of UB-612 will compare with GMT of Anti-S1-RBD on Visit 6 (pre-3rd dose base line) by paired t test. This analysis will be performed with the Evaluable Booster Population only.

- **Geometric mean fold increase in SARS-CoV-2 neutralizing antibody and antigen-specific antibody (Anti-S1-RBD) on14 days post 3rd dose against pre-3rd dose baseline**

  GMFI and 2-sided 95% CIs will be provided. Summary of descriptive statistics by age group will be also performed. This analysis will be performed with the Evaluable Booster Population only.

- **Geometric mean titer (GMT) of SARS-CoV-2 neutralizing antibody and antigen-specific antibody (Anti-S1-RBD) on Day 57, Day 197 and Day 365 in adolescents**

  The analysis of GMT of SARS-CoV-2 neutralizing antibody on Day 57, Day 197, 14 days post 3rd dose and Day 365 in adolescents will follow the analysis described above in GMT.

- **Seroconversion rate (SCR) of SARS-CoV-2 neutralizing antibody on Day 57 and antigen-specific antibody (Anti-S1-RBD) in adolescents**

  The analysis of SCR of SARS-CoV-2 neutralizing antibody on Day 57 in adolescents will follow the analysis described above in SCR.

- **Geometric mean fold increase in SARS-CoV-2 neutralizing antibody and antigen-specific antibody (Anti-S1-RBD) on Day 57, Day 197, and Day 365 in adolescents**

The analysis of GMFI in SARS-CoV-2 neutralizing antibody and antigen-specific antibody (Anti-S1-RBD) on Day 57, Day 197, and Day 365 in adolescents will follow the analysis of secondary immunology endpoint in GMFI.

- Local reactions for up to 7 days following each dose in adolescents

  The analysis of local reactions for up to 7 days following each dose in adolescents will follow the analysis of local reactions in primary safety endpoints.

- Systemic events for up to 7 days following each dose in adolescents

  The analysis of systemic events for up to 7 days following each dose in adolescents will follow the analysis of systemic events in primary safety endpoints.

- Unsolicited AEs from Day 1 to Day 57 in adolescents

  The analysis of unsolicited AEs from Day 1 to Day 57 in adolescents will follow the analysis of unsolicited AEs in primary safety endpoints.

- **MAAEs and SAEs from Day 1 to Day 365 in adolescents**

  The analysis of MAAEs and SAEs from Day 1 to Day 365 in adolescents will follow the analysis of MAAEs and SAEs in primary safety endpoints.

- **AESIs and ADEs from Day 1 to Day 365 in adolescents**

  The analysis of AESIs and ADEs from Day 1 to Day 365 in adolescents will follow the analysis of AESIs and ADEs in primary safety endpoints.

- Changes of safety laboratory measures in adolescents

  The analysis of changes of safety laboratory measures in adolescents will follow the analysis of changes of safety laboratory measures in secondary safety endpoint.

- COVID-19 incidence per 1000 person-years of follow-up based on PCR test

  COVID-19 incidence per 1000 person-years of follow-up based on PCR test will be descriptively summarized by counts, percentages, and associated Clopper-Pearson 95% CIs for each study group.

- To describe the anti-S1-RBD IgG levels and SARS-CoV-2 neutralizing titers to UB-612 in confirmed and/or severe COVID-19 cases

  The anti-S1-RBD IgG levels and SARS-CoV-2 neutralizing titers to UB-612 in confirmed COVID-19 cases will be presented by descriptive summary statistics and associated 95% CIs for each study group.

- **To detect antibody against SARS-CoV-2 antigens derived from S2, N, and M protein**

The results of UBI SARS-CoV-2 ELISA will be presented by descriptive summary statistics

10.8 Subgroup Analysis

Additional study populations and subgroups will be assessed, with further information available in the SAP.

10.9 Interim Analysis

Six interim analyses and reports will be performed when (1) adult immunogenicity data (350 evaluable young adults and 154 evaluable elderly subjects) for Day 57 are available; (2) at least half of core group subjects (at least 3500 evaluable subjects) will be completed Day 85 safety follow up, as EUA application dossier; (3) all immunogenicity data for lot-to lot consistency of Day 57 are available, EUA application dossier; (4) 350 evaluable adolescents completed Day 57 safety follow up, as supplementary dossier for EUA**; (5) immunogenicity and safety data for young and elderly adult subjects receiving 3rd vaccination completed Visit 7, which is 14 days after 3rd vaccination; (6) immunogenicity and safety data for adolescent subjects receiving 3rd vaccination completed Visit 7, which is 14 days after 3rd vaccination.**

The first four analyses and reports will be analysed and drafted by the unblinded statistician, reviewed by the IDMC, and submitted to appropriate regulatory authorities for review if as EUA application dossier or supplementary dossier for EUA.

For the evaluation of safety, immunogenicity, and lot-to-lot consistency, the following endpoints will be included in the first four interim analyses.

- Local reactions, systemic events, unsolicited AEs, and SAEs
- Changes of safety laboratory measures
- Changes of vital signs and physical examinations
- Seroconversion rate of SARS-CoV-2 neutralizing antibody and antigen-specific antibody (Anti-S1-RBD) on Day 57
- Geometric mean titer of SARS-CoV-2 neutralizing antibody and antigen-specific antibody (Anti-S1-RBD) on Day 57
- Geometric mean fold increase in SARS-CoV-2 neutralizing antibody and antigen-specific antibody (Anti-S1-RBD) on Day 57
- Lot consistency by the comparisons of the GMT of SARS-CoV-2 neutralizing titers on Day 57 induced by 3 independent UB-612 vaccine clinical materials. The 95% confidence intervals between groups will be within the margin of 0.5 to 2.

For last two interim analyses (i.e., interim analysis (5) and (6)), the analyses demonstrate the immunogenicity and safety data on the adult and adolescent subjects received 3rd dose of UB-612 including (but not limited to) following endpoints.

- **Local reactions, systemic events, unsolicited AEs, and SAEs after 3rd dose of UB-612**
- **Changes of safety laboratory measures**
- **Changes of vital signs and physical examinations pre/post 3rd dose of UB-612**

- **GMT of SARS-CoV-2 neutralizing antibody on 14 days post 3rd dose of UB-612**
- **GMT of antigen-specific antibody (Anti-S1-RBD) on 14 days post 3rd dose of UB-612**
- **GMFIs in SARS-CoV-2 neutralizing antibody and antigen-specific antibody (Anti-S1-RBD) on14 days post 3rd dose against pre-3rd dose baseline**

10.10 Handling of Missing Data

All available data will be displayed and utilized in data analysis. No imputation will be considered for the missing observations.

10.11 Protocol Deviations

Protocol deviations will be categorized into important and non-important items, and definitions will be illustrated in the protocol deviation handling plan (PDHD). Events that beyond the PDHD will discuss with sponsor to determine the categorization.

11 QUALITY ASSURANCE AND QUALITY CONTROL

11.1 Audit and Inspection

Study centers and study documentation may be subject to Quality Assurance audit during the course of the study by the sponsor or its nominated representative. In addition, inspections may be conducted by regulatory authorities at their discretion.

11.2 Monitoring

Data for each subject will be recorded on an eCRF. Data collection must be completed for each subject who signs an ICF and is administered study agent.

Due to study design, monitoring activities are divided into blinded and unblinded team. Blinded team will follow the Blinded Monitoring Plan, while unblinded team should follow the Unblinded Monitoring Plan. Unblinded team will report to unblinded UBIA staff. **Members in blinded team should be blinded to immunogenicity data.**

In accordance with current good clinical practice (cGCP) and International Council for Harmonisation (ICH) guidelines, the study monitor will carry out source document verification at regular intervals to ensure that the data collected in the eCRF are accurate and reliable.

The investigator must permit the monitor, the IEC/IRB, the sponsor's internal auditors, and representatives from regulatory authorities direct access to all study-related documents and pertinent hospital or medical records for confirmation of data contained within the eCRFs.

11.3 Data Management and Coding

The sponsor and/or the appointed representative(s) will be responsible for activities associated with the data management of this study. This will include setting up a relevant database and data transfer mechanisms, along with appropriate validation of data and resolution of queries. Data generated within this clinical study will be handled according to the relevant standard operating procedures of the data management and biostatistics departments of the sponsor and/or the appointed representative(s).

Study centers will complete the eCRF. Data entered into the eCRF must be verifiable against source documents at the study center. Data to be recorded directly on the eCRF will be identified and the eCRF will be considered the source document. Any changes to the data entered into the data capture system will be compliant to FDA CFR 21 Part 11.

Medical coding will use Medical Dictionary for Regulatory Activities for AEs.

Missing or inconsistent data will be queried to the investigator for clarification. Subsequent modifications to the database will be documented.

12 RECORDS AND SUPPLIES

12.1 Drug Accountability

On receipt of the study agent (including rescue medication, if relevant), the investigator (or designee) will conduct an inventory of the supplies and verify that study agent supplies are received intact and in the correct amounts before completing a supplies receipt. The investigator will retain a copy of this receipt at the study center and return the original receipt to the study monitor. The monitor may check the study supplies at each study center at any time during the study.

It is the responsibility of the study monitor to ensure that the investigator (or designee) has correctly documented the amount of the study agent received, dispensed, and returned on the dispensing log that will be provided. A full drug accountability log will be maintained at the study center at all times. The study monitor will arrange collection of unused study agent returned by the subject. The study monitor will also perform an inventory of study agent at the close-out visit to the study center. All discrepancies must be accounted for and documented.

12.2 Financing and Insurance

Financing and insurance of this study will be outlined in a separate agreement between the contract research organization and the sponsor.

13 ETHICS

13.1 Independent Ethics Committee or Institutional Review Board

Before initiation of the study at each study center, the protocol, the ICF, other written material given to the subjects, and any other relevant study documentation will be submitted to the appropriate IEC/IRB. Written approval of the study and all relevant study information must be obtained before the study center can be initiated or the study agent is released to the investigator. Any necessary extensions or renewals of IEC/IRB approval must be obtained for changes to the study such as amendments to the protocol, the ICF or other study documentation. The written approval of the IEC/IRB together with the approved ICF must be filed in the study files.

The investigator will report promptly to the IEC/IRB any new information that may adversely affect the safety of the subjects or the conduct of the study. The investigator will submit written summaries of the study status to the IEC/IRB as required. On completion of the study, the IEC/IRB will be notified that the study has ended.

13.2 Regulatory Authorities

Relevant study documentation will be submitted to the regulatory authorities of the participating countries, according to local/national requirements, for review and approval before the beginning of the study. On completion of the study, the regulatory authorities will be notified that the study has ended.

13.3 Ethical Conduct of the Study

The investigator(s) and all parties involved in this study should conduct the study in adherence to the ethical principles based on the Declaration of Helsinki, cGCP, ICH guidelines, and the applicable national and local laws and regulatory requirements.

13.4 Informed Consent

The process of obtaining informed consent must be in accordance with applicable regulatory requirement(s) and must adhere to cGCP.

The investigator is responsible for ensuring that no subject undergoes any study related examination or activity before that subject has given written informed consent to participate in the study.

The investigator or designated personnel will inform the subject of the objectives, methods, anticipated benefits and potential risks and inconveniences of the study. The subject should be given every opportunity to ask for clarification of any points s/he does not understand and, if necessary, ask for more information. At the end of the interview, the subject will be given ample time to consider the study. Subjects will be required to sign and date the ICF. After signatures are obtained, the ICF will be kept and archived by the investigator in the investigator’s study file. A signed and dated copy of the subject ICF will be provided to the subject or their authorized representative.

It should be emphasized that the subject may refuse to enter the study or to withdraw from the study at any time, without consequences for their further care or penalty or loss of benefits to which

the subject is otherwise entitled. Subjects who refuse to give or who withdraw written informed consent should not be included or continue in the study.

If new information becomes available that may be relevant to the subject's willingness to continue participation in the study, a new ICF will be approved by the IEC(s)/IRB(s) (and regulatory authorities, if required). The study subjects will be informed about this new information and reconsent will be obtained.

13.5 Subject Confidentiality

Monitors, auditors, and other authorized agents of the sponsor and/or its designee, the IEC(s)/IRB(s) approving this research, and the United States (US) FDA, as well as that of any other applicable agency(ies), will be granted direct access to the study subjects' original medical records for verification of clinical study procedures and/or data, without violating the confidentiality of the subjects to the extent permitted by the law and regulations. In any presentations of the results of this study or in publications, the subjects' identity will remain confidential.

All personal data collected and processed for the purposes of this study should be managed by the investigator and his/her staff with adequate precautions to ensure confidentiality of those data, applicable to national and/or local laws and regulations on personal data protection.

14 REPORTING AND PUBLICATION, INCLUDING ARCHIVING

Essential documents are those documents that individually and collectively permit evaluation of the study and quality of the data produced. After completion of the study (end of study defined as the date of the last visit of the last subject), all documents and data relating to the study will be kept in an orderly manner by the investigator in a secure study file. This file will be available for inspection by the sponsor or its representatives. Essential documents should be retained for 2 years after the final marketing approval in an ICH region or for at least 2 years since the discontinuation of clinical development of the investigational product. It is the responsibility of the sponsor to inform the study center when these documents no longer need to be retained. The investigator must contact the sponsor before destroying any study related documentation. In addition, all subject medical records and other source documentation will be kept for the maximum time permitted by the hospital, institution, or medical practice.

The sponsor must review and approve any results of the study or abstracts for professional meetings prepared by the investigator(s). Published data must not compromise the objectives of the study. Data from individual study centers in multicenter studies must not be published separately.

15 REFERENCES

1. Hui DS, I Azhar E, Madani TA, et al. The continuing 2019-nCoV epidemic threat of novel coronaviruses to global health - The latest 2019 novel coronavirus outbreak in Wuhan, China. Int J Infect Dis. 2020;91:264-266.
2. Zhu N, Zhang D, Wang W, et al. A Novel Coronavirus from Patients with Pneumonia in China, 2019. N Engl J Med. 2020;382(8):727-733.
3. Guo YR, Cao QD, Hong ZS, et al. The origin, transmission and clinical therapies on coronavirus disease 2019 (COVID-19) outbreak - an update on the status. Mil Med Res. 2020;7(1):11. Published 2020 Mar 13.
4. Phan T. Novel coronavirus: From discovery to clinical diagnostics. Infect Genet Evol. 2020;79:104211.
5. Hoffmann M, Kleine-Weber H, Schroeder S, et al. SARS-CoV-2 Cell Entry Depends on ACE2 and TMPRSS2 and Is Blocked by a Clinically Proven Protease Inhibitor. Cell. 2020;181(2):271-280.e8.
6. Huang C, Wang Y, Li X, et al. Clinical features of patients infected with 2019 novel coronavirus in Wuhan, China [published correction appears in Lancet. 2020 Jan 30]. Lancet. 2020;395(10223):497-506.
7. Remy V, Largeron N, Quilici S, Carroll S. The Economic Value of Vaccination: Why Prevention Is Wealth. Value Health. 2014;17(7):A450.
8. Takashima Y, Osaki M, Ishimaru Y, Yamaguchi H, Harada A. Artificial molecular clamp: a novel device for synthetic polymerases. Angew Chem Int Ed Engl. 2011;50(33):7524-7528.
9. Liu H, Su D, Zhang J, et al. Improvement of Pharmacokinetic Profile of TRAIL via Trimer-Tag Enhances its Antitumor Activity in vivo. Sci Rep. 2017;7(1):8953.
10. Jiang S. Don't rush to deploy COVID-19 vaccines and drugs without sufficient safety guarantees. Nature. 2020;579(7799):321.
11. Peeples L. News Feature: Avoiding pitfalls in the pursuit of a COVID-19 vaccine. Proc Natl Acad Sci U S A. 2020;117(15):8218-8221.
12. Grifoni A, Weiskopf D, Ramirez SI, et al. Targets of T Cell Responses to SARS-CoV-2 Coronavirus in Humans with COVID-19 Disease and Unexposed Individuals. Cell. 2020;S0092-8674(20)30610-3.
13. Centers for Disease Control and Prevention. Cases and Deaths in the U.S. https://www.cdc.gov/coronavirus/2019-ncov/cases-updates/us-cases-deaths.html. Published 2020. Updated June 24, 2020. Accessed June 25, 2020.
14. Charlson M E, Pompei P, Ales K L, et al. A new method of classifying prognostic. comorbidity in longitudinal studies: development and validation. J Chronic Dis, 1987;40(5):373-83.
15. Barbara L., Miriam S. Priority List of Adverse Events of Special Interest: COVID-19. Safety Platform for Emergency Vaccines. V1.1, 05 March 2020
16. COVID-19 manifestations associated with more severe presentation and decompensation with

consideration of enhanced disease potential. The current listing is based on the Coalition for Epidemic Preparedness Innovations/Brighton Collaboration Consensus Meeting (12/13 March 2020) and expected to evolve as evidence accumulates (Lambert 2020)

17. Cytokine release syndrome related to COVID-19 infection is a disorder characterized by nausea, headache, tachycardia, hypotension, rash, and/or shortness of breath (DAIDS,2017)

Appendix 8

中國醫藥大學附設醫院
CHINA MEDICAL UNIVERSITY HOSPITAL
台中市北區育德路2號
2 Yude Road, Taichung, 40447, Taiwan (R.O.C.)
TEL : 886-4-22052121

中國醫藥大學暨附設醫院研究倫理委員會

Tel: 886-4-22052121 ext: 1925 Fax: 886-4-2207-1478 台中市北區育德路 2 號

計畫修正案通過證明書

計畫名稱：一個評估 UB-612 疫苗對於新型冠狀病毒於青少年、成年和老年健康受試者的免疫原性、安全性與耐受性的第二期、安慰劑控制、隨機分派、觀察者盲性臨床試驗

計畫編號/本會編號：V-205 / CMUH109-REC1-176(AR-5)

計畫主持人：兒童感染科黃高彬主治醫師

試驗機構：中國醫藥大學附設醫院

原計畫通過日期：2020 年 12 月 21 日至 2021 年 12 月 20 日

修正案通過日期：2021 年 09 月 21 日至 2021 年 12 月 20 日

計畫書：Version 3.0, Date: 06 September, 2021

中文摘要：Version 3.0, Date: 2021-09-06

英文摘要：Version 3.0, Date: 06 September, 2021

受試者同意書(成年免疫組)：Version 3.0, Date: Sep. 06, 2021

受試者同意書(青少年免疫組)：Version 3.0, Date: Sep. 06, 2021

受試者同意書(成年安全確認組)：Version 3.0, Date: Sep. 06, 2021

受試者同意書(青少年安全確認組)：Version 3.0, Date: Sep. 06, 2021

受試者同意書 (成年免疫第三劑組)：Version 3rd dose 1.0, Date: 20210906

受試者同意書 (青少年免疫第三劑組)：Version 3rd dose 1.0, Date: 20210906

受試者同意書 (成年安全確認第三劑組)：Version 3rd dose 1.0, Date: 20210906

受試者同意書 (青少年安全確認第三劑組)：Version 3rd dose 1.0, Date: 20210906

個案報告表：Version 4.0, Date: 06 September, 2021

The Committee is organized and operates in accordance with ICH6 GCP regulations and guideline.
本委員會組織與運作皆遵守 ICH6 GCP 規定

中國醫藥大學附設醫院
CHINA MEDICAL UNIVERSITY HOSPITAL
台中市北區育德路2號
2 Yude Road, Taichung, 40447, Taiwan (R.O.C.)
TEL : 886-4-22052121

中國醫藥大學暨附設醫院研究倫理委員會

Tel: 886-4-22052121 ext: 1925 Fax: 886-4-2207-1478 台中市北區育德路 2 號

上述計畫已於 2021 年 09 月 15 日經中國醫藥大學暨附設醫院研究倫理委員會第一審查委員會 2021 年第 10 次審查會議審查。本委員會的運作符合優良臨床試驗準則及國內相關法令。請在持續審查必須進行前二個月向本會檢送完整之期中報告。

此計畫任何部分若經更改，必須在執行前重新提交本會審查及核准。此外，計畫主持人必須依時通報嚴重不良事件及涉及受試者或其他人風險的非預期問題。

中國醫藥大學暨附設醫院
研究倫理委員會
台中市北區育德路2號

主任委員 傅茂祖

中 華 民 國 一 一 〇 年 九 月 二 十 八 日

The Committee is organized and operates in accordance with ICH6 GCP regulations and guideline.
本委員會組織與運作皆遵守 ICH6 GCP 規定

中國醫藥大學附設醫院
CHINA MEDICAL UNIVERSITY HOSPITAL
台中市北區育德路2號
2 Yude Road, Taichung, 40447, Taiwan (R.O.C.)
TEL : 886-4-22052121

Research Ethics Committee
China Medical University & Hospital, Taichung, Taiwan

Tel: 886-4-22052121 ext: 1925 Fax: 886-4-2207-1478

Clinical Trial/Human Research Approval

Amendment Review

Date：Sep. 28, 2021

Protocol Title：A Phase II, Placebo-controlled, Randomized, Observer-blind Study to Evaluate the Immunogenicity, Safety and Tolerability of UB-612 Vaccine against COVID-19 in Adolescent, Younger and Elderly Adult Volunteers

Protocol No. / CMUH REC No.：V-205 / CMUH109-REC1-176(AR-5)

Name of Principal Investigator：Kao-Pin Hwang (Attending Physician, Pediatric Infectious Diseases)

Name of Institution：China Medical University Hospital

Valid Date of Original Research Project: From Dec. 21, 2020 to Dec. 20, 2021

Valid Date of Amended Research Project: From Sep. 21, 2021 to Dec. 20, 2021

Protocol：Version 3.0, Date: 06 September, 2021

Chinese Synopsis：Version 3.0, Date: 2021-09-06

English Synopsis：Version 3.0, Date: 06 September, 2021

Informed Consent Form (Immunogenicity Group-Adult)：Version 3.0, Date: Sep. 06, 2021

Informed Consent Form (Immunogenicity Group-Adolescent)：Version 3.0, Date: Sep. 06, 2021

Informed Consent Form (Safety Check Group-Adult)：Version 3.0, Date: Sep. 06, 2021

Informed Consent Form (Safety Check Group-Adolescent)：Version 3.0, Date: Sep. 06, 2021

Informed Consent Form (3rd Dose Immunogenicity Group-Adult)：Version 3rd dose 1.0, Date: 20210906

Informed Consent Form (3rd Dose Immunogenicity Group-Adolescent)：Version 3rd dose 1.0, Date: 20210906

The Committee is organized and operates in accordance with ICH6 GCP regulations and guideline.

CMUH

中國醫藥大學附設醫院
CHINA MEDICAL UNIVERSITY HOSPITAL
台中市北區育德路2號
2 Yude Road, Taichung, 40447, Taiwan (R.O.C.)
TEL : 886-4-22052121

Research Ethics Committee

China Medical University & Hospital, Taichung, Taiwan

Tel: 886-4-22052121 ext: 1925 Fax: 886-4-2207-1478

Informed Consent Form (3rd Dose Safety Check Group-Adult)：Version 3rd dose 1.0, Date: 20210906

Informed Consent Form (3rd Dose Safety Check Group-Adolescent)：Version 3rd dose 1.0, Date: 20210906

Case Report Form：Version 4.0, Date: 06 September, 2021

This is to certify that the above referenced amended research project has been reviewed by the 2021 10th meeting of the Research Ethics Committee (REC) I of the China Medical University and Hospital on Sep. 15, 2021. The REC is organized under, and operates in accordance with, the Good Clinical Practices guidelines and the governmental laws and regulations. Please submit a completed progress report at least two months before the time at which continuing review must occur.

All the amendments to the research project should be re-submitted and approved by the REC BEFORE implementation. Also, the principal investigator is required to report all serious adverse events and unanticipated problems involving risks to the subjects or others on time.

CHINA MEDICAL UNIVERSITY & HOSPITAL
The Research Ethics Committee
NO. 2 Yude Road, Taichung, Taiwan, R.O.C.

Martin M-T Fuh MD, DMSci.
Chairman, Research Ethics Committee I
China Medical University & Hospital

Institutional Review Board
National Cheng Kung University Hospital
138 Sheng-Li Rd, Tainan 704, Taiwan R.O.C.
TEL:886-6-2353535 ext.3635 FAX:886-6-2388190

國立成功大學醫學院附設醫院 A119(3)
第一人體研究倫理審查委員會
台灣.台南市勝利路138號
E-mail:em73635 @mail.hosp.ncku.edu.tw

同意計畫修正證明書

研究計畫名稱：一個評估 UB-612 疫苗對於新型冠狀病毒於青少年、成年和老年健康受試者的免疫原性、安全性與耐受性的第二期、安慰劑控制、隨機分派、觀察者盲性臨床試驗

計畫編號/本會編號: V-205 / AB-CR-110-011

研究執行期間:民國 110 年 02 月 23 日至民國 111 年 12 月 31 日

本次核准期間:民國 110 年 02 月 23 日至民國 111 年 02 月 22 日

核准內容/版本：

1. 計畫書：Version 3.0, Date: 20210906
2. 中文摘要：Version 3.0, Date: 20210906
3. 英文摘要：Version 3.0, Date: 20210906
4. 受試者同意書(成年免疫組)：Version 2.0, Date:20210916
5. 受試者同意書(青少年免疫組)：Version 2.0, Date:20210916
6. 受試者同意書(成年安全確認組)：Version 2.0, Date:20210916
7. 受試者同意書(青少年安全確認組)：Version 2.0, Date:20210916
8. 個案報告表：Version 4.0, Date: 20210906
9. 新增送審文件：
   - 受試者同意書(成年免疫第三劑組)：Version 3rd dose 1.0, Date: 20210916
   - 受試者同意書(青少年免疫第三劑組)：Version 3rd dose 1.0, Date: 20210916
   - 受試者同意書(成年安全確認第三劑組)：Version 3rd dose 1.0, Date: 20210916
   - 受試者同意書(青少年安全確認第三劑組)：Version 3rd dose 1.0, Date: 20210916
10. 變更協同主持人：郭正彥醫師退出，新增王冠元醫師

試驗機構：成大醫院

研究計畫主持人：楊宜青主任(社區健康照護中心)

協同主持人：柯文謙醫師、楊登棋醫師、劉介修醫師、羅玉岱醫師、周佑聰醫師、陳泓裕醫師、蘇斐琳醫師、吳怡萱醫師、周杰穎醫師、鄭翔如醫師、廖信閔醫師、王竣令醫師、林筱茹醫師、吳晉祥醫師、謝奇璋醫師、沈靜芬醫師、蔡孟哲醫師、林亭妤醫師、王冠元醫師

協同研究員：施譪衿研究助理、郭筱薇研究助理、賴虹樺研究助理、陳彥如研究助理、王端玲研究助理、姜美如研究助理

本會經中央衛生主管機關查核通過，組織與執行皆遵照法令及主管機關規範。

本修正計畫已於**民國 110 年 10 月 27 日**經本院人體研究倫理審查委員會審核通過，本次核准執行期間至**民國 111 年 02 月 22 日**，特此證明。

已完成之研究應於研究執行期間末日後三個月內繳交結案報告，除維護受試者安全之必要作為外，於核准期間末日後應停止執行所有受試者相關之研究程序。

計畫主持人逾核准期間末日仍未繳交報告者，列入逾期名單，本會將寄發本研究案之中止/終止通知書。逾期名單將提本會審查會議報告，經會議決議後，本會將暫停受理名單上人員所主持之新案審查申請，迨繳交應繳報告並經本會會議審查通過後，始得受理其新案審查申請。

追蹤/結案報告請以書面繳交；報告書請逕送本院人體研究倫理審查委員會辦公室；報告表格最新版本請至本會網頁(http://nckuhirb.med.ncku.edu.tw/)下載。

研究計畫內容有任何變更或修正(含研究執行期間變更)，須於研究執行期間內向本會提出申請，本會不受理未在研究執行期間內提出之變更或修正案。變更或修正未獲本會核准前，須依原核准範圍執行。

已獲本會同意之研究案，因故未開始執行或不繼續執行者，應申請中止/終止。

不論研究進行中或研究完成後，受試者若發生任何不良反應，須依 GCP 規範通報。

此致

國立成功大學醫學院附設醫院
人體研究倫理審查委員會
主任委員

中 華 民 國 110 年 10 月 28 日

Institutional Review Board
National Cheng Kung University Hospital
138 Sheng-Li Rd, Tainan 704, Taiwan R.O.C.
TEL:886-6-2353535 ext.3635 FAX:886-6-2388190

國立成功大學醫學院附設醫院
第一人體研究倫理審查委員會
台灣.台南市勝利路138號
E-mail:em73635 @mail.hosp.ncku.edu.tw

Human Study Amendment Approval

Date:2021.10.28

Title: A Phase II, Placebo-controlled, Randomized, Observer-blind Study to Evaluate the Immunogenicity, Safety and Tolerability of UB-612 Vaccine against COVID-19 in Adolescent, Younger and Elderly Adult Volunteers
Protocol No/ IRB No: V-205 / AB-CR-110-011
Period of Project: From 2021.02.23 to 2022.12.31
Period of Approval: From 2021.02.23 to 2022.02.22
Content/Version:

1. Protocol: Version 3.0, Date: 20210906
2. Chinese synopsis: Version 3.0, Date: 20210906
3. English synopsis: Version 3.0, Date: 20210906
4. Informed Consent Form (Immunogenicity group -Adult): Version 2.0, Date:20210916
5. Informed Consent Form (Immunogenicity group -Adolescent): Version 2.0, Date:20210916
6. Informed Consent Form (Safety check group -Adult): Version 2.0, Date:20210916
7. Informed Consent Form (Safety check group -Adolescent): Version 2.0, Date:20210916
8. Case Report Form: Version 4.0, Date: 20210906
9. Add Submission Documents:
   - Informed Consent Form (3rd dose Immunogenicity group -Adult): Version 3rd dose 1.0, Date: 20210916
   - Informed Consent Form (3rd dose Immunogenicity group -Adolescent): Version 3rd dose 1.0, Date: 20210916
   - Informed Consent Form (3rd dose Safety check group -Adult): Version 3rd dose 1.0, Date: 20210916
   - Informed Consent Form (3rd dose Safety check group -Adolescent): Version 3rd dose 1.0, Date: 20210916
10. Amend Co-Investigator: Withdraw Dr. Cheng-Yen Kuo, Add Dr. Kuan-Yuan Wang

Institute: National Cheng Kung University Hospital
Investigator: Director Yi-Ching Yang (Community Healthcare Center)
Sub-Investigator: Dr. Wen-Chien Ko, Dr. Deng-Chi Yang, Dr. Chieh-Hsiu Liu, Dr. Yu-Tai Lo, Dr. Yu-Tsung Chou, Dr. Hung-Yu Chen, Dr. Fei-Lin Su, Dr. I-Hsuan Wu, Dr. Chieh-Ying Chou, Dr. Hsiang-Ju Cheng, Dr. Jiun-Ling Wang, Dr. Shin-Ming Liao, Dr. Hsiao-Ju Lin, Dr Jin-Shang Wu, Dr. Chi-Chang Shieh, Dr. Ching-Fen Shen, Dr.Meng-Che Tsai, Dr. Ting-Yu Lin, Dr. Kuan-Yuan Wang
Co-Researcher: Research Assistant Hui-Chin Shih, Research Assistant Hsiao-Wei Kuo, Research Assistant Hung-Hua Lai, Research Assistant Yen-Ju Chen, Research Assistant Tuan-Ling Wang, Research Assistant Mei-Ru Chiang

The Institutional Review Board of National Cheng Kung University Hospital (NCKUH) is organized and operated according to the laws and regulations of ICH-GCP and of Central Competent Authorities.

This project is reviewed and approved by NCKUH IRB in **2021.10.27.** The period of approval is granted until **2022.02.22.**

Regarding completed project, the Final Report shall be submitted within three months of its approved expiry date. Except for the health of the participants, all the procedures of the project shall be terminated on its approved stated deadline.

If PI does not submit the Interim/Final Report on time, he/she will be recorded in the overdue list and received the suspension/ termination notice from NCKUH IRB. The overdue list will be reported to the IRB. After the resolution of the board meeting, NCKUH IRB will suspend all the new projects applied by PI until the Interim/Final Report is submitted.

Please submit the Interim/Final Report in written form and send to NCKUH IRB office. The lastest application forms can be downloaded in its website (http://nckuhirb.med.ncku.edu.tw/)

Any changes or amendments to the project (including the project period), please submit an amendment application to NCKUH IRB within its approved period. Any changes or amendments in any other way will not be accepted. Before the approval of the amendment application, the project is carried out according to its previously approved plan.

For some reasons projects granted approval by NCKUH IRB couldn't be implemented, PI shall apply for suspension/termination.

During or after the project is completed, please report any unfavorable occurrence in a human study participant according to GCP.

Yours sincerely,
Ting-Tsung Chang M.D.
Chairman

Institutional Review Board
National Cheng Kung University Hospital

長庚醫療財團法人人體試驗倫理委員會

臨床試驗/研究同意證明書

地　　址：105台北市敦化北路199號
傳　　真：03-3494549
聯絡人及電話：薛育婷(03)3196200#3703
電子郵件信箱：sally6869@cgmh.org.tw

計畫名稱： 一個評估UB-612疫苗對於新型冠狀病毒於青少年、成年和老年健康受試者的免疫原性、安全性與耐受性的第二期、安慰劑控制、隨機分派、觀察者盲性臨床試驗

計畫編號： V-205

本院案號： 202100188A4C602

試驗期間： 2021年2月20日~2023年1月5日

本次核准執行期間：2021年10月12日~2022年2月19日

總主持人： 感染醫學科 黃景泰 學術組教授級主治醫師

主持人： 感染醫學科 李禎祥 學術組教授級主治醫師

協同主持人： 葉峻甫,李允吉,張鵬浩,黃文琦,黃柏諺,蘇庭儀,劉仲淇,鄭鈞文,陳怡君,謝顯森,吳彥穆,蔡青晏,郭泓頡

研究助理： 温鵬毅,王筠婷,李兆芸,洪慧雯,孫秀琪,莊惠姍,張芳瑋,莊培瑜,黃惠琳,莊淑婷,王儀鳳,郭筱音,王思怡,陳立君,陳品蓁,康瑋庭,吳珮嘉,丁秋煥,陳美真,趙潔欣,劉靜芬,林奕臻,許淑慧,劉清華,林奕君

試驗/研究機構： 林口長庚醫院,高雄長庚醫院

計畫文件版本日期：

(1)計畫書： Version 3.0, Date: 06 September, 2021

(2)中文摘要： Version 3.0, Date: 2021-09-06

(3)英文摘要： Version 3.0, Date: 06 September, 2021

(4)受試者同意書：
受試者同意書(成年安全確認第三劑組林口院區)：2021/09/16 Version 3rd dose 1.0
受試者同意書(成年安全確認第三劑組高雄院區)：2021/09/16 Version 3rd dose 1.0
受試者同意書(成年免疫第三劑組林口院區)：2021/09/16 Version 3rd dose 1.0
受試者同意書(青少年安全確認第三劑組高雄院區)：2021/09/16 Version 3rd dose 1.0
受試者同意書(青少年安全確認第三劑組林口院區)：2021/09/18 Version 3rd dose 1.0
受試者同意書(青少年免疫第三劑組林口院區)：2021/09/18 Version 3rd dose 1.0
受試者同意書(成年安全確認組林口院區)：2021/09/16 Version 2.0
受試者同意書(成年免疫組林口院區)：2021/09/16 Version 2.0
受試者同意書(青少年安全確認組林口院區)：2021/09/18 Version 2.0
受試者同意書(青少年免疫組林口院區)：2021/09/18

Version 2.0
受試者同意書(成年安全確認組高雄院區)：2021/09/16
Version 2.0
受試者同意書(青少年安全確認組高雄院區)：2021/09/16
Version 2.0

(5)其他文件： 人體試驗申請表
19A 檢體資訊
檢體外送及儲存: 新增外送及儲存機構 NEXELIS (機構地址: 525 Boul. Cartier Ouest Laval, Qulbec, Canada, H7V 3S8 (加拿大))，Vaccinology and Immunology Infection, Immunity & Inflammation Dept UCL GOS Institute of Childe Health (機構地址: UCL Great Ormond Street Institute of Child Health 30 Guilford Street London WC1N 1EH, England (英國))，VisMederi (機構地址: VisMederi Srl, Strada del Petriccio e Belriguardo, 35, 53100 Siena, Italy (義大利))
研究助理-許美秀退出

※本案須重新簽署受試者同意書

通過日期：2021年10月12日

DSMB/DSMP：已有DSMP,已有其他安全監測

期中報告繳交頻率：半年繳交一次

※本試驗計劃案業經本院人體試驗倫理委員會審核通過，同意追認其他合法審查會通過之研究計畫之核可。

※下次期中報告繳交截止日期：2022年2月19日，為免影響主持人執行研究之權益，請於截止日前六至八週繳交報告(期中報告繳交頻率為三個月者，得於試驗到期前一個月繳交報告)，以利審查作業進行。若主持人未繳交或延遲繳交期中報告，以致本會無法於核准執行期間到期前，核發下次試驗執行期間，所有的研究活動必須停止，包括：對已參與受試者之介入或各項互動，除非本會認為受試者繼續接受試驗介入或參與試驗顯有益於受試者安全或倫理上符合受試者最佳利益之情形，亦不得再納入新個案，直到期中報告核准為止。

本委員會組織與運作皆遵守GCP規定

長庚醫療財團法人

人體試驗倫理委員會謝燦堂主席

2021 年 10 月 12 日

Chang Gung Medical Foundation

Institutional Review Board

199, TUNG HWA NORTH ROAD,
TAIPEI, TAIWAN, 10507
REPUBLIC OF CHINA
Tel: (03) 3196200
Fax: (03) 3494549

Date 2021/10/12

Protocol Title: Phase II, Placebo-controlled, Randomized, Observer-blind Study to Evaluate the Immunogenicity, Safety, and Tolerability of UB-612 Vaccine against COVID-19 in Adolescent, Younger and Elderly Adult Volunteers

Protocol No.: V-205

IRB No.: 202100188A4C602

Chief Investigator(s): HUANG,CHING-TAI

Principal Investigator(s): LEE-CHEN-HSIANG

Co-Investigator(s): YEH, CHUN-FU,LI-YUN-CHI,CHANG, PENG-HAO,WEN CHI HUANG,HUANG, PO-YEN,SU, TING-YI,LAO CHONG KEI,CHENG,CHUN-WEN,YI-CHUN CHEN,SHIE,SHIAN-SEN,WU YEN-MU,TSAI, CHING-YEN,KUO, HONG-JIE

Study Coordinator(s): Wen Pengyi,WANG,YUN-TING,LI CHAO-YUN,HUI-WEN HUNG,HSIU CHIU SUN,HUI-SHAN CHUANG,CHANG FANG-WEI,Pei-Yu Chuang,HUANG HUI-LING,CHUANG, SHU-TING,Yi-Feng Wang,KUO,HSIAO-YING,WANG SZU YI,Li Chun Chen,PIN-CHEN CHEN,KANG WEI-TING,PEI-JIA WU,DING CIOU-HUAN,MEI-CHEN, CHEN,,,liu ching fen,LIN,YI-JHEN,HSU,SHU-HUI,LIU CHING HUA,Yichun Lin

Executing Institution: Linkou,Kaohsiung

Duration of Approval: From 2021/10/12 To 2022/2/19

Version/Date of documents:
(1)Protocol: Version 3.0, Date: 06 September, 2021
(2)Chinese Synopsis: Version 3.0, Date: 2021-09-06
(3)English Synopsis: Version 3.0, Date: 06 September, 2021
(4)Informed Consent Form:
2021/09/16 Version 3rd dose 1.0
2021/09/16 Version 3rd dose 1.0
2021/09/16 Version 3rd dose 1.0
2021/09/16 Version 3rd dose 1.0
2021/09/18 Version 3rd dose 1.0
2021/09/18 Version 3rd dose 1.0
2021/09/16 Version 2.0
2021/09/16 Version 2.0
2021/09/18 Version 2.0
2021/09/18 Version 2.0
2021/09/16 Version 2.0
2021/09/16 Version 2.0

(5)Others: 人體試驗申請表
19A 檢體資訊
檢體外送及儲存: 新增外送及儲存機構 NEXELIS (機構地址: 525 Boul. Cartier Ouest Laval, Qulbec, Canada, H7V 3S8 (加拿大))，Vaccinology and Immunology Infection, Immunity & Inflammation Dept UCL GOS Institute of Childe Health (機構地址: UCL Great Ormond Street Institute of Child Health 30 Guilford Street London WC1N 1EH, England (英國))，VisMederi (機構地址: VisMederi Srl, Strada del Petriccio e Belriguardo, 35, 53100 Siena, Italy (義大利))
研究助理-許美秀退出

Date of Approval: 2021/10/12
DSMB/DSMP: Already Had DSMP,Already had other safety monit
Frequency of Continuing Report: Every 6 months

※The re-consent process shall be required

※The trial research has been reviewed and approved by Chang Gung Medical Foundation Institutional Review Board, and the research has already been approved by other legal Institutional Review Board shall be recognized.

※Next Deadline of Continuing Report: 2022/02/19. To facilitate the review, please submit the report two months before the deadline (or one month before the expiration of the trial if a continuing report shall be provided every three months) in order not to influence the principal investigator’ s right to conduct the research. In the case that failure or delay to submit a continuing report makes the IRB unable to determine the next trial period by the deadline, the trial shall not be continuously conducted.If the Principal Investigator fails to submit a continuing report on time, rendering the Institutional Review Board unable to issue the next trial execution period before the previous trial execution period expires, all research activities, including the intervention or interaction with the participating trial subjects, must be suspended. Unless the Institutional Review Board considers that the continuation of trial intervention or trial participation is greatly beneficial to the trial subject's safety or in the best interest of the trial subject from a moral point of view, no new trial subject shall be included until the continuing report is formally approved.

The IRB is organized and operates in accordance with Good Clinical Practice and the applicable laws and regulations.

Tsang-Tang Hsieh,MD
Chairman
Institutional Review Board
Chang Gung Medical Foundation

【主持人須知】

一、 本人負責執行此臨床試驗，依赫爾辛基宣言精神與國內相關法令之規定，確保受試者之權益、健康、個人隱私與尊嚴。

二、 計畫執行期間與結束後，皆要求人員確實維護可辨識資料機密性或設計相關機制，並依法規存放年限妥善保存。

三、 本人不得洩露因業務知悉之秘密或與研究對象有關之資訊，會善盡保護受試者隱私的義務。隱私(privacy)是指個人私人的範圍，例如年紀、身分證統一編號、婚姻狀態、電話、 住址、病史、家族史等，不希望讓他人知道或與他人分享的部分。

四、 本人會善盡維護資料機密性的責任，並對於相關資料會有適當安全維護措施。資料機密性(confidentiality)是指個人可辨識資料的管理。

五、 實施人體研究計畫前，應擬定研究計畫，經人體試驗倫理委員會審查通過，始得為之。另醫療法所稱之人體試驗案及應用人體生物資料庫檢體進行之案件，尚需經衛生福利部核准，方可進行。人體試驗倫理委員會或主管機關命令中止/終止試驗案件時，不得繼續執行。

六、 廠商贊助試驗於執行前，應協助試驗執行醫院完成臨床試驗合約書簽署。合約書簽署流程及注意事項應依本院之「廠商贊助計畫管理作業準則」辦理。展延試驗期間者，經人體試驗倫理委員會同意後，應進行合約變更。

七、 試驗進行前，主持人應確實核對試驗計畫書、受試者同意書等之正確版本，以及人體試驗倫理委員會與衛生福利部核准之試驗進行期間；醫療法所稱之人體試驗案，應於本院人體試驗倫理委員會與衛生福利部皆已核准，方可進行；並以人體試驗倫理委員會核准之同意臨床試驗證明迄日為試驗截止日。

八、 應完全熟悉試驗藥品/醫療材料、醫療技術在試驗計畫書、最新主持人手冊及其他由試驗委託者提供的相關資訊中描述的使用方法。

九、 應明瞭並遵守「醫療法」、「人體試驗管理辦法」、「人體研究法」、「人體生物資料庫管理條例」、「藥品優良臨床試驗準則」、「醫療器材優良臨床試驗準則」等相關法規，以及本院「人體研究暨受試者保護作業管理辦法」、「人體生物資料庫管理辦法」、「研究材料外送作業準則」等之規定，善盡保護受試者之責任，並配合主管機關、人體試驗倫理委員會及執行機構的查核。

十、 應確保所有協助臨床試驗的相關人員，對試驗計畫書及試驗藥品/醫療材料、醫療技術有充分的了解，以及他們在臨床試驗中相關的責任和工作，並定期召開會議討論。

十一、 試驗主持人應負責所有臨床試驗相關的醫療決定。

十二、 在受試者參加試驗與後續追蹤期間，試驗主持人應對受試者任何與試驗相關的不良反應，提供充分的醫療照護，包括重要實驗室檢查值等。當試驗主持人察覺試驗期間受試者有疾病需要醫療照護時，必須告知受試者。

十三、 主持人須遵照「嚴重藥物不良反應通報辦法」及「藥品優良臨床試驗準則」、「人體試驗管理辦法」之規定，於時效內向相關單位完成通報。發生非預期且相關之嚴重藥品不良反應，試驗主持人應立即通知人體試驗倫理委員會，如為衛生福利部列管案件，並需確認試驗委託者已通報主管機關；如為主持人自行發起之案件，主持人需通報主管機關。但若試驗計畫書或其他文件明確排除者，不在此限。如為已上市藥品試驗案發生藥品不良反應（ADR），應依規定至院內網頁之安全通報作業系統完成通報。

十四、 試驗主持人應依本院「人體研究暨受試者保護作業管理辦法」規定繳交期中報告及結案報告，未繳者不得再申請新案，針對未於執行期間屆期前取得期中報告續核准，應立即停止研究案之所有程序，案件審議如有必要時須配合於會議列席報告。

十五、 受試者同意書使用原則須遵照「藥品優良臨床試驗準則」之規定，若具有重要性之新資訊可能影響受試者之同意時，應修訂受試者同意書及提供受試者之任何其他書面資料，並應立即告知受試者、法定代理人或有同意權之人，並重新取得書面同意。修訂後之受試者同意書及提供受試者之任何其他書面資料，應先得到人體試驗倫理委員會之核准；經主管機關核准進行之臨床試驗，並應得到主管機關之核准。

十六、 經人體試驗倫理委員會核准之計畫內容，凡於核准期間內任一變更(包含試驗計劃書、主持人手冊、受試者同意書、問卷、量表、執行院區、計畫主持人、試驗委託者等)，應於執行前送變更案審查，於接獲書面核准函後，始可為之。另計劃主持人因故自行暫停、終止或結案時，應以書面通知本會。

十七、 試驗研究團隊執行試驗時發生未遵照審查通過之計畫書、主管機關所訂立之法令規章或本會規定之情事，應遵照本院「人體研究暨受試者保護作業管理辦法」之規定，於時效內完成通報。

十八、 人體試驗倫理委員會同意之試驗期間到期，但需要展延試驗期間者，應於有效期限到期前2個月提出申請。

十九、 醫療法所稱人體試驗範圍之案件，侵入性檢查或治療，以及使用上市藥品之案件需至『HIS系統-研究計畫-研究計畫申請-臨床試驗執行管控作業』登錄受試者資料。

二十、 試驗主持人於門診(住院)時發現受試者發生與試驗相關的不良反應，需轉診時，試驗主持人應主動向受試者及家屬解釋，並協助轉診(會診)，且於病歷(會診單)上詳細記載本試驗目的、副作用、目前受試者病情及處置，並口頭告知轉診主治醫師。

二十一、 若為多國多中心之案件，計畫主持人須確認第一個Kit之正確性。

二十二、 應遵照「人體試驗管理辦法」、「醫療機構及醫事人員發布醫學新知或研究報告倫理守則」及本院「研究成果發表於報章媒體作業準則」規定，醫療法所稱人體試驗之研究成果須經衛生福利部審核通過後，試驗主持人始得發表。

二十三、 因試驗計畫主持人過失造成醫院或他人受損害時，須由計畫主持人負法律責任。

二十四、 免除受試者同意後仍會適時提供受試者試驗相關訊息。

二十五、 當受試者在納入研究後成為受刑人，主持人得知後應以非預期問題通報本會及試驗委託者。

二十六、 試驗團隊於試驗執行時需遵守事項：臨床試驗執行需於病歷記載、於病房收案需向護理人員進行教育訓練及需填寫試驗團隊工作職責分配表、每位計畫主持人執行廠商贊助計畫件數不得超過10件。

二十七、 若長庚醫療財團法人人體試驗倫理委員會審定須執行簽署受試者同意書程序，本人承諾遵循所提出之簽署受試者同意書程序，並由本人或授權之團隊成員完整詳細的解說並取得知情同意。計畫執行前，應獲得受試者自願給予之受試者同意書。執行時應確認使用長庚醫療財團法人人體試驗倫理委員會核准之最新用印版本受試者同意書。

二十八、 若試驗藥物同時在美國和歐盟進行試驗，依美國或歐盟藥品管理規定，則試驗結果將公佈於公開的臨床試驗資訊網站：Clinicaltrials.gov (美國)，clinicaltrialsregister.eu (歐盟)，所有臨床試驗需在納入第一位受試者前，登記在可供大眾取得的資料庫。

二十九、 以上內容經本人確認無誤，若需要願提供所需之相關資料予人體試驗倫理委員會，以提供受試者權益之審查。

三十、 其餘未盡事宜，請參照本院「人體研究暨受試者保護作業管理辦法」辦理。

CHANG GUNG MEDICAL FOUNDATION

199 TUNG HWA NORTH ROAD

TAIPEI, TAIWAN, 10507

REPUBLIC OF CHINA

TEL(03)3196200

FAX(03)3494549

Institutional Review Board B

2020 July - 2022 June

MEMBER	GENDER	PRIMARY SPECIALTY	AFFILIATION OF INSTITUTION
主席 : 謝燦堂<br>HSIEH, TSANG-TANG	Male<br>男性	Obstetrics/Gynecology<br>婦產科專科	CGMF (Taipei)<br>台北長庚醫院
副主席 : 賴瓊慧<br>LAI, CHYONG-HUEY	Female<br>女性	Obstetrics/Gynecology<br>婦產科專科	CGMF (Lin-Kou)<br>林口長庚醫院
醫療執秘 : 林永昌<br>LIN, YUNG-CHANG	Male<br>男性	Hematology/Oncology<br>腫瘤科專科	CGMF (Lin-Kou)<br>林口長庚醫院
鄭欽明<br>CHENG, CHIN-MING	Male<br>男性	Business Management<br>企業管理	Children's Hearing Foundation<br>財團法人雅文兒童聽語文教基金會
王悅賢<br>Wang, Yueh-Hsien	Male<br>男性	Law<br>法律	Baker & McKenzie, Taipei<br>國際通商法律事務所
黃英霓<br>Ying Ni Huang	Female<br>女性	Law<br>法律	None<br>無
蔡芸芳<br>TSAI,YUN-FANG	Female<br>女性	Nursing<br>護理	Chang Gung University<br>長庚大學
蕭靜熹<br>CHING-HSI-HSIAO	Female<br>女性	ophthalmology<br>眼科	CGMF (Taipei)<br>台北長庚醫院
邱玟惠<br>WEN-HUI CHIU	Female<br>女性	Law<br>法律	Soochow University<br>東吳大學
葉森洲<br>YEH, SAN-JOU	Male<br>男性	Cardiology<br>心臟內科專科	CGMF (Taipei)<br>台北長庚醫院
陳怡安<br>IAN CHEN	Male<br>男性	Family Medicine<br>家庭醫學專科	National Taiwan University Hospital<br>台大醫院
洪泰和<br>HUNG, TAI-HO	Male<br>男性	Obstetrics and Gynecology<br>婦產科專科	CGMF (Taipei)<br>台北長庚醫院
馮啟文<br>FENG CHI-WEN	Male<br>男性	Ethics<br>宗教	Bread of Life Christian Church in Lin-Kou<br>基督教林口靈糧堂
鄒小蕙<br>Hsiao-Hui Tsou	Female<br>女性	Statistics<br>統計	National Health Research Institutes<br>財團法人國家衛生研究院
楊政達<br>YANG, CHENG-TA	Male<br>男性	Pulmonary and Critical Care Medicine<br>胸腔內科專科	CGMF (Taoyuan)<br>桃園長庚醫院
廖繼洲<br>Chi-Chou Liao	Male<br>男性	Pharmacy<br>藥事	None<br>無

胡幼圃<br>Oliver Yoa Pu Hu	Male<br>男性	Pharmacy<br>藥事	National Defense Medical Center<br>國防醫學院
陳順勝<br>SHUN--SHENG-CHEN	Male<br>男性	Neurology<br>神經內科專科	CGMF (Kaohsiung)<br>高雄長庚醫院

Chairman: T. T. Hsieh
Tsang-Tang Hsieh,MD

高雄醫學大學附設中和紀念醫院
Kaohsiung Medical University Chung-Ho Memorial Hospital
人體試驗審查委員會
Institutional Review Board

807高雄市三民區自由一路100號
No. 100, Tzyou 1st Road, Kaohsiung 807, Taiwan
TEL： 886-7-3121101 ext.6646、886-7-3133525
FAX： 886-7-3221408

人體研究變更案同意證明書

計畫中文名稱：一個評估UB-612疫苗對於新型冠狀病毒於青少年、成年和老年健康受試者的免疫原性、安全性與耐受性的第二期、安慰劑控制、隨機分派、觀察者盲性臨床試驗

計畫主持人：盧柏樑

共同及協同主持人：蔡明儒、陳彥旭、林俊祐、林尚儀、黃崇豪、張雅婷、羅世豪、李雋元、李純瑩、張家禎、許超群、洪仁宇、鄭孟軒、陳家閔、陳惇杰、李敏生、陳昭儒、蔡毓德、李杰明

研究人員：許晏禎、江玟靜、江如萍、黃建豪、胡嘉桂、李依鴻、李茉華

機構名稱：高雄醫學大學附設中和紀念醫院

經費來源：聯亞生技開發股份有限公司

本會編號：KMUHIRB-F(II)-20210029

計畫編號：V-205

核准日期(審查通過日)：2021/10/15

計畫執行期間：2021/2/19-2022/12/31

本同意書有效期限：2022/2/18

本次修正版本：

計畫書：Version 3.0, Date: 06 September, 2021

中文摘要：Version 3.0, Date: 06 September, 2021

英文摘要：Version 3.0, Date: 06 September, 2021

個案報告表：Version 4.0, Date: 06 September, 2021

受試者同意書：

(1) 成年安全確認組：Version 2.0, Date: 20210917

(2) 青少年安全確認組：Version 2.0, Date: 20210917

本次新增版本：

第三劑受試者同意書：

(1) 成年安全確認組：Version 3rd dose 1.0, Date:20210917

(2) 青少年安全確認組：Version 3rd dose 1.0, Date:20210917

凡經衛生福利部列管之計畫案件，變更案須取得衛生福利部審核同意，方可執行變更後內容，並請確實依衛生福利部核准並符合本院人體試驗審查委員會同意之各文件版本執行。未預期事件或藥品嚴重不良反應通報後續定期追蹤之程序及應注意事項，請參閱背面。

高雄醫學大學 人體試驗 審查委員會 附設中和紀念醫院

高雄醫學大學附設中和紀念醫院
第一人體試驗審查委員會
主任委員：顏學偉

西 元 2 0 2 1 年 1 0 月 1 5 日

高雄醫學大學附設中和紀念醫院
Kaohsiung Medical University Chung-Ho Memorial Hospital
人體試驗審查委員會
Institutional Review Board

807高雄市三民區自由一路100號
No. 100, Tzyou 1st Road, Kaohsiung 807, Taiwan
TEL： 886-7-3121101 ext.6646、886-7-3133525
FAX： 886-7-3221408

Approval of Clinical Trial/Research

Protocol Title: *A Phase II, Placebo-controlled, Randomized, Observer-blind Study to Evaluate the Immunogenicity, Safety, and Tolerability of UB-612 Vaccine against COVID-19 in Adolescent, Younger and Elderly Adult Volunteers*

Principal Investigator: *Po-Liang Lu*

Co_Investigator(s): *Ming-Ju Tsai, Yen-Hsu Chen, Chun-Yu Lin, Shang-Yi Lin, Chung-Hao Huang, Ya-Ting Chang, Shih-Hao Lo, Chun-Yuan Lee, Chun-Ying Lee, Chai-Ja Chang, Chau-Chyun Sheu, Jen-Yu Hung, Meng-Hsuan Cheng, Chia-Min Chen, Tun-Chieh Chen, Min-Sheng Lee, Chao-Ju Chen, Yu-Te Tsai, Chieh-Ming Lee*

Study Coordinator: *Yan-Zhen Hsu, Wen-Ching Jiang, Ru-Ping Jiang, Chien-Hao Huang, Chia-Kuei Hu, Yi-Hong Li, Mo-Hua Li*

Institution: *Kaohsiung Medical University Chung-Ho Memorial Hospital*

Source of Funding: *United Biomedical, Inc., Asia*

IRB Number: *KMUHIRB-F(II)-20210029*

Protocol Number: *V-205*

Approval Date: *2021/10/15*

Duration of Approval: *2021/2/19- 2022/12/31*

Expiration Date of Approval: *2022/2/18*

Amendment:

Protocol: Version 3.0, Date: 06 September, 2021

Chinese Protocol Synopsis: Version 3.0, Date: 06 September, 2021

English Protocol Synopsis: Version 3.0, Date: 06 September, 2021

Informed Consent Form:

(1) Informed Consent Form (Safety check group -Adult): Version 2.0, Date: 20210917

(2) Informed Consent Form (Safety check group -Adolescent): Version 2.0, Date: 20210917

Case Report Form: Version 4.0, Date: 06 September, 2021

Add:

Informed Consent Form (3rd dose):

(1) Informed Consent Form 3rd dose (Safety check group –Adult): Version 3rd dose 1.0, Date:20210917

(2) Informed Consent Form 3rd dose (Safety check group –Adolescent: Version 3rd dose 1.0, Date:20210917

See the back of this page for the procedures for reporting unanticipated problems, or drug serious adverse reactions, or interim, and other important notes.

Hsueh-Wei Yen

Hsueh-Wei Yen, MD
Chairman
Institutional Review Board- I
Kaohsiung Medical University
Chung-Ho Memorial Hospital

Kaohsiung Medical University Institutional Review Board Chung-Ho Memorial Hospital

Tel: 886-2-87923311 ext 10552
Fax:886-2-2792-9201
E-mail: tsghirb@ndmctsgh.edu.tw

國防醫學院三軍總醫院
人體試驗審議會

台北市 11490 內湖區成功路二 325 號醫療大樓五樓 5113 室
No.325,Sec.2, Cheng-Kung Rd.
Neihu 11490, Taipei, Taiwan, R.O.C

人體試驗/研究計畫同意函

本審議會案號：C202101005
計畫編號：V-205
計畫名稱：一個評估 UB-612 疫苗對於新型冠狀病毒於青少年、成年和老年健康受試者的免疫原性、安全性與耐受性的第二期、安慰劑控制、隨機分派、觀察者盲性臨床試驗
執行機構：三軍總醫院
計畫主持人：感染及熱帶醫學科張峰義醫師
共同主持人：陳相成醫師
協同主持人：葉富強醫師；方文輝醫師；陳韋良醫師；劉偉修醫師；張芳維醫師
通過類型：變更案
通過日期：2021 年 10 月 19 日
同意核准執行期間：2021/9/5～2022/3/4
持續審查報告繳交頻率：半年一次(中度風險)
※本案須經衛生福利部核准同意後，始得進行試驗。
※本案須重新簽署受試者同意書。
※下次持續審查報告繳交截止日期：2022/3/4，應於到期日至少 6 週前提出持續審查申請，本案需經持續審查，方可繼續執行，若於到期日前完成試驗/研究，請繳交結案報告。
計畫主持人須依國內相關法令及本院規定通報嚴重不良反應事件及非預期問題。
本審議會組織與運作皆遵守 GCP 規定

Letter of Approval

TSGHIRB No.：C202101005
Protocol No.：V-205
Protocol title：A Phase II, Placebo-controlled, Randomized, Observer-blind Study to Evaluate the Immunogenicity, Safety, and Tolerability of UB-612 Vaccine against COVID-19 in Adolescent, Younger and Elderly Adult Volunteers
Research institution：Tri-Service General Hospital
Principle investigator：Dr. Feng-Yee Chang
Co-investigator：Dr. Hsiang-Cheng Chen
Sub investigator：Dr. Fu-Chiang Yeh；Wen-Hui Fang；Wei-Liang Chen；Wei-Hsiu Liu；Fung-Wei Chang
Type of Approval： Amendment
Date of Approval：2021/10/19
Duration of Approval：2021/9/5～2022/3/4
Frequency of Continuing Report：follow-up review 6 months (medium risk)
※The project may not be implemented until the approval is granted by the Ministry of Health and Welfare.
※Re-consent process is required.
※Next Deadline of Continuing Report：2022/3/4. If the study is completed prior to the approved expiration date, provide the Final Report.
The investigator is required to report any Serious Adverse Events and Unanticipated Problems in accordance with the governmental laws and regulations requirements
The organization and operation of the IRB is in accordance with Good Clinical Practice (GCP) and the applicable laws and regulations.

三軍總醫院人體試驗審議會 TSGHIRB

Institutional Review Board
余慕賢 YuMuHsien
Chairman________________________________.

Tel: 886-2-87923311 ext 10552
Fax:886-2-2792-9201
E-mail: tsghirb@ndmctsgh.edu.tw

國防醫學院三軍總醫院
人體試驗審議會

台北市 11490 內湖區成功路二 325 號醫療大樓五樓 5113 室
No.325,Sec.2, Cheng-Kung Rd.
Neihu 11490, Taipei, Taiwan, R.O.C

計畫文件版本日期 Version/Date of documents

變更項目：

1. 計畫書 Protocol：Version 3.0_06 September 2021
2. 中文摘要 Chinese Synopsis：Version 3.0_06 September 2021
3. 英文摘要 English Synopsis：Version 3.0_06 September 2021
4. 受試者同意書 Informed Consent Form：
   (1) 青少年安全確認組 Safety Check Group -Adolescent：v2.0/2021-09-17
   (2) 成年安全確認組 Safety Check Group -Adult：v2.0/2021-09-17
   (3) 青少年安全確認第三劑組 Safety Check Group 3rd dose -Adolescent：v1.0/2021-09-17
   (4) 成年安全確認第三劑組 Safety Check Group 3rd dose -Adult：v1.0/2021-09-17
5. 個案報告表 CRF：Version 4.0_06 September 2021

三軍總醫院 TSGHIRB 人體試驗審議會

Tel: 886-2-87923311 ext 10552
Fax:886-2-2792-9201
E-mail: tsghirb@ndmctsgh.edu.tw

國防醫學院三軍總醫院
人體試驗審議會

台北市 11490 內湖區成功路二 325 號醫療大樓五樓 5113 室
No.325,Sec.2, Cheng-Kung Rd.
Neihu 11490, Taipei, Taiwan, R.O.C

計畫主持人應注意事項

一、本人負責執行此人體試驗/研究，依赫爾辛基宣言精神與國內相關法令之規定，確保受試者之自主權益、個人隱私與福祉。

二、計畫執行期間與結束後，本人會善盡維護資料機密性的責任，並對於相關資料會有適當安全維護措施，皆要求研究團隊成員確實維護可辨識資料機密性或設計相關機制，並依法規與本會審查通過內容存放年限妥善保存。

三、本人不得洩露因業務知悉之秘密或與研究對象有關之資訊，會善盡保護受試者隱私的義務。隱私是指個人私人的範圍，例如年紀、身分證統一編號、婚姻狀態、電話、 住址、病史、家族史等，不希望讓他人知道或與他人分享的部分。

四、實施醫療法所稱之人體試驗案，應於本會與衛生福利部皆已核准，方可進行。

五、試驗進行前，計畫主持人應確實核對本會與衛生福利部核准之試驗計畫書、受試者同意書等文件之正確版本，以及試驗進行期限。

六、本人應完全熟悉試驗藥品/醫療材料、醫療技術在試驗計畫書、最新主持人手冊及其他由試驗委託者提供的相關資訊中描述的使用方法。應確保所有協助臨床試驗的相關人員，對試驗計畫書及試驗藥品/醫療材料、醫療技術有充分的了解，以及他們在臨床試驗中相關的責任和工作，並定期召開會議討論。

七、經核准之計畫內容，凡於核准期間內任一變更(包含計畫書、主持人手冊、受試者同意書、問卷、量表、執行地點、執行期限、團隊成員、試驗委託者等)，應於執行前送變更案審查，於審查通過後，始可為之。另計畫主持人因故自行暫停、終止或結案時，應提出報告通知本會，並確保受試者有適當之治療及追蹤。

八、在受試者參加試驗與後續追蹤期間，確保對受試者任何與試驗相關的不良反應，包括重要實驗室檢查值等，提供充分的醫療照護。當察覺試驗期間受試者有疾病需要醫療照護時，必須告知受試者。

九、計畫執行期間若發生未預期之死亡或危及生命以外之嚴重不良事件，計畫相關人員需於 15 天內通報衛生福利部及本會，若此嚴重不良事件屬未預期之死亡或危及生命之事件，須於獲知該事件之內提出初始報告，並於 15 天內提供追蹤報告，可以傳真或書面通報方式為之。通報內容需含本院「臨床試驗藥品不良反應送件說明」、衛生福利部藥物不良反應中心 ADR 通報表、計畫之不良反應通報表、其他相關報表等資料。其它不良事件可於持續審查或結案/終止/撤案審查、定期安全性報告彙整呈報。

十、遵循所提出之簽署受試者同意書之程序，並須經過完整詳細的解說並取得知情同意。試驗執行前，應獲得受試者自願給予之受試者同意書。執行時應確認使用有蓋上本審議會核准章之最新版本受試者同意書。

十一、在院外執行之人體試驗需經該機構核備同意。

十二、依相關規定繳交期中及結案/終止/撤案審查。依衛生福利部及本會規定，人體試驗審議會必須每年進行至少一次追蹤審查，重新審查該計畫是否同意繼續進行。請於同意函有效期屆滿前送交持續審查報告以利審查，若尚未通過追蹤審核，不得繼續試驗。試驗完成後，應將執行情形及結果報告送至本會核備。

十三、試驗暫停或終止時，試驗主持人及試驗委託機構應立即通知主管機關及本會，並確保受試者有適當之治療及追蹤。

副本

臺北榮民總醫院　書函

地址：11217 臺北市北投區石牌路二段201號
承辦人：李允意
電話：2875-7384轉255
電子信箱：yylee11@vghtpe.gov.tw

11051
台北市信義區信義路四段415號3樓之1
受文者：晉加股份有限公司

發文日期：中華民國110年11月11日
發文字號：北總人試字第1104905649號
速別：普通件
密等及解密條件或保密期限：
附件：

主旨：同意臺端主持之「一個評估UB-612疫苗對於新型冠狀病毒於青少年、成年和老年健康受試者的免疫原性、安全性與耐受性的第二期、安慰劑控制、隨機分派、觀察者盲性臨床試驗（計畫編號：V-205）」（本院IRB編號：2021-04-003AU#4）研究計畫變更案乙案，詳如說明段，請查照。

說明：

一、本案業經本院110年11月1日人體試驗委員會(一)第143次會議審查通過，同意修正計畫書、中文摘要、英文摘要、受試者同意書兩份(成年安全確認組、青少年安全確認組)、個案報告表；新增受試者保護指引、第三劑組受試者同意書兩份(成年安全確認第三劑組、青少年安全確認第三劑組)，版本如同意臨床試驗證明書所載。

二、計畫主持人應辦及注意事項如后：

(一)本研究計畫之核准蓋印文件請自行至臨床資訊管理系統（PTMS）（網址：https://vghtpe.cims.tw/）計畫修正案送審文件「35.其他」下載使用。

(二)《人體試驗委員會受試者同意書審定本》已加蓋騎縫

章，請複印以進行知情同意程序。

(三) 依人體試驗管理辦法第15條規定「醫療機構於人體試驗期間，不得對外發表成果或為宣傳」。已核准之廣告紙本須經本院人體試驗委員會蓋戳印方可張貼。

(四)若需展延本院人體試驗委員會核准期限，請於有效期限前3個月至6週（至少前6週）向人體試驗委員會申請持續審查，並經同意後方可繼續執行。

(五)試驗若須變更、暫停執行時，應向本院人體試驗委員會提出審查申請並經同意後，始得實施。

(六)試驗結束後於有效期限到期後3個月內，或試驗終止或撤案時，請依規定向本院人體試驗委員會提出結案、終止及撤案申請。

(七)若未於有效期限到期後3個月內提出結案、或試驗終止或撤案申請之計畫主持人，本院人體試驗委員會得不受理其爾後新案之申請，並得予以適當之處置。

正本：本院內科部感染科王復德醫師

副本：聯亞生技開發股份有限公司/ United Biomedical, Inc., Asia、晉加股份有限公司、本院臨床研究受試者保護中心、人體試驗委員會

臺北榮民總醫院

臺　北　榮　民　總　醫　院
TAIPEI VETERANS GENERAL HOSPITAL
201 SHIH-PAI ROAD. SEC.2
TAIPEI, TAIWAN 11217
REPUBLIC OF CHINA
TEL:(886)-2-2871-2121

同意臨床試驗/研究證明書

IRB 編號：2021-04-003AU#4

計畫編號：V-205

計畫名稱：一個評估 UB-612 疫苗對於新型冠狀病毒於青少年、成年和老年健康受試者的免疫原性、安全性與耐受性的第二期、安慰劑控制、隨機分派、觀察者盲性臨床試驗

部門/計畫主持人：感染科/ 王復德醫師

協同主持人：林邑璁醫師、黃信彰醫師、洪妙秋醫師、陳曾基醫師、張曉婷醫師、劉瑞瑤醫師、陳育群醫師、鄭博仁醫師、陳夙容醫師、林宜璁醫師、陳育民醫師、陳威志醫師、蘇剛正醫師、潘聖衛醫師、陽光耀醫師、余文光醫師、柯信國醫師、黃惠君醫師、黃鈴茹醫師、楊盈盈醫師

變更之計畫文件版本日期：

1. 受試者保護指引 Subject Protection Guidance: Version 1.2, Date: 20210902
2. 計畫書 Protocol：Version 3.0, Date: 20210906
3. 中文摘要 Chinese Synopsis：Version 3.0, Date: 20210906
4. 英文摘要 English Synopsis：Version 3.0, Date: 20210906
5. 受試者同意書 Informed Consent Form：
   - 受試者同意書 (成年安全確認組)：Version 2.0, Date: 20211008
   - 受試者同意書 (青少年安全確認組)：Version 2.0, Date: 20211008
6. 第三劑組受試者同意書 Informed Consent Form for 3rd dose：
   - 受試者同意書 (成年安全確認第三劑組)：Version 1.0, Date: 20211008
   - 受試者同意書 (青少年安全確認第三劑組)：Version 1.0, Date: 20211008
7. 個案報告表 Case Report Form：Version 4.0, Date: 20210906

依據本委員會標準作業程序、及政府相關法律規章，本計畫修正/變更案案經本院人體試驗委員會(一)110 年 11 月 01 日第 143 次會議，於 110 年 11 月 01 日審查通過，有效期限至 111 年 03 月 14 日止，特此證明。本委員會的運作符合藥品優良臨床試驗準則及政府相關法律規章。計畫主持人須依國內相關法令及本院規定通報嚴重不良反應事件及非預期問題。計畫主持人須於到期前 3 個月至 6 週（至少前 6 週）提出持續審查之申請，本案須經本院人體試驗委員會通過後，方可繼續執行。（凡需送衛生福利部審核之計畫案件，須取得衛生福利部審核同意函後方可開始執行）

臺北榮民總醫院人體試驗委員會 Institutional Review Board Taipei Veterans General Hospital

馬旭

臺北榮民總醫院
人體試驗委員會
主任委員
馬　旭

中　華　民　國　1　1　0　年　1　1　月　0　5　日

臺 北 榮 民 總 醫 院
TAIPEI VETERANS GENERAL HOSPITAL
201 SHIH-PAI ROAD. SEC.2
TAIPEI, TAIWAN 11217
REPUBLIC OF CHINA
TEL:(886)-2-2871-2121

Clinical Trial/Research Approval Letter

Nov 05, 2021

IRB-TPEVGH No.: 2021-04-003AU#4
Protocol No:V-205
Protocol Title: A Phase II, Placebo-controlled, Randomized, Observer-blind Study to Evaluate the Immunogenicity, Safety and Tolerability of UB-612 Vaccine against COVID-19 in Adolescent, Younger and Elderly Adult Volunteers
Department/Principal Investigator: Division of Infectious Disease/ Fu-Der Wang, M.D.
Sub-Investigator: Yi-Tsung Lin, M.D., Shinn-Jang Hwang, M.D., Miao-Chiu Hung, M.D., Tzeng-Ji Chen, M.D., Hsiao-Ting Chang, M.D., Jui-Yao Liu, M.D., Yu-Chun Chen, M.D., Bo-ren Cheng, M.D., Su-Jung Chen, M.D., Yi-Tsong Lin, M.D., Yuh-Min Chen, M.D., Wei-Chih Chen, M.D., Kang-Cheng Su, M.D., Sheng-Wei Pan, M.D., Kuang-Yao Yang, M.D., Wen-Kuang Yu, M.D., Hsin-Kuo Ko, M.D., Hui-Chun Huang, M.D., Ling-Ju Huang, M.D., Ying-Ying Yang, M.D
Version date of Amendment documents:

1. Subject Protection Guidance: Version 1.2, Date: 20210902
2. Protocol：Version 3.0, Date: 20210906
3. Chinese Synopsis：Version 3.0, Date: 20210906
4. English Synopsis：Version 3.0, Date: 20210906
5. Informed Consent Form：
   - Informed Consent Form (Safety check group -Adult)：Version 2.0, Date: 20211008
   - Informed Consent Form (Safety check group -Adolescent)：Version 2.0, Date: 20211008
6. Informed Consent Form for 3rd dose：
   - Informed Consent Form (Safety check group －Adult 3rd dose)：Version 1.0, Date: 20211008
   - Informed Consent Form (Safety check group －Adolescent 3rd dose)：Version 1.0, Date: 20211008
7. Case Report Form：Version 4.0, Date: 20210906

According to the written operating procedures, GCP, and the applicable regulatory requirements, this Amendment study project is reviewed by the143th meeting of the Institutional Review Board (1) of Taipei Veterans General Hospital on Nov 01, 2021, and approved on Nov 01, 2021. This approval is valid till Mar 14, 2022. The board is organized under, and operates according to International Conference on Harmonisation (ICH) / WHO Good Clinical Practice (GCP) and the applicable laws and regulations. The principal investigator is required to report Serious Adverse Events and Unanticipated Problems in accordance with the governmental laws and regulations and TPEVGH requirements.The principal investigator is required to submit the application for extension before the expiration date of 6 weeks to 3 months (at least 6 weeks). (If indicated by the regulations and laws, this project should be taken after the approval of Ministry of Health and Welfare, R.O.C.)

臺北榮民總醫院 人體試驗委員會 Institutional Review Board Taipei Veterans General Hospital

Hsu Ma, M.D.
Chairman
Institutional Review Board
Taipei Veterans General Hospital
Taiwan, R.O.C.

高雄榮民總醫院人體研究倫理審查委員會
KAOHSIUNG V.G.H. Institutional Review Board
TEL：07-3422121-71571
FAX：07-3468344
e-mail：irb@vghks.gov.tw

人體試驗計畫同意函(1/2)

計畫名稱：一個評估UB-612疫苗對於新型冠狀病毒於青少年、成年和老年健康受試者的免疫原性、安全性與耐受性的第二期、安慰劑控制、隨機分派、觀察者盲性臨床試驗
計畫編號：KSVGH21-CT3-05
計畫主持人：蔡宏津醫師(hctsai1011@yahoo.com.tw；0975-581737)
通過會期：第213次會議
通過日期：2021年10月6日

修正項目：

1. **計畫書：Version 3.0, Date: 20210906**
2. **中文摘要：Version 3.0, Date: 20210906**
3. **英文摘要：Version 3.0, Date: 20210906**
4. **受試者同意書：**
   (1) **受試者同意書 (成年安全確認組)：Version 2.0, Date: 20210915**
   (2) **受試者同意書 (青少年安全確認組)：Version 2.0, Date: 20210915**
5. **新增第三劑組受試者同意書：**
   (1) **受試者同意書 (成年安全確認第三劑組)：Version 3rd dose 1.0, Date: 20210915**
   (2) **受試者同意書 (青少年安全確認第三劑組)：Version 3rd dose 1.0, Date: 20210915**
6. **個案報告表：Version 4.0, Date: 20210906**

有效期限：2022年2月23日
試驗機構：高雄榮民總醫院

主任委員 陳金順

2021年10月6日

＊計畫主持人須遵守之規定請見「計畫主持人之職責」。

高雄榮民總醫院人體研究倫理審查委員會
KAOHSIUNG V.G.H. Institutional Review Board
TEL：07-3422121-71571
FAX：07-3468344
e-mail：irb@vghks.gov.tw

Certificate of Approval(2/2)

Protocol Title：A Phase II, Placebo-controlled, Randomized, Observer-blind Study to Evaluate the Immunogenicity, Safety, and Tolerability of UB-612 Vaccine against COVID-19 in Adolescent, Younger and Elderly Adult Volunteers
IRB No.：KSVGH21-CT3-05
Principal Investigator：Dr. Hung-Chin Tsai
(hctsai1011@yahoo.com.tw；0975-581737)
Board Meeting：213th
Approval Date：Oct. 6, 2021

Reason for Amendment：

1. **Protocol：Version 3.0, Date: 20210906**
2. **Chinese Synopsis：Version 3.0, Date: 20210906**
3. **English Synopsis：Version 3.0, Date: 20210906**
4. **Informed Consent Form：**
   (1) Safety check group - Adult：Version 2.0, Date: 20210915
   (2) Safety check group - Adolescent：Version 2.0, Date: 20210915
5. **Informed Consent Form for 3rd dose：**
   (1) 3rd Dose Safety check group - Adult：Version 3rd dose 1.0, Date: 20210915
   (2) 3rd Dose Safety check group - Adolescent：Version 3rd dose 1.0, Date: 20210915
6. **Case Report Form：Version 4.0, Date: 20210906**

Study Approval Expires：Feb. 23, 2022
Site: Kaohsiung Veterans General Hospital

Jin-Shuen, Chen, M.D
Chairman
Jin-Shuen Chen
Oct. 6, 2021

＊Please review and follow the responsibility of the Principal Investigator.

本會組織與執行皆符合ICH-GCP
IRB-KSVGH performs its functions according to written operating procedures and complies with GCP and with the applicable regulatory requirements

高雄榮民總醫院人體研究倫理審查委員會

計畫主持人之職責

依據衛生福利部及本院人體研究倫理審查委員會規定，計畫主持人必須確實遵守以下事項：

1. 保護受試者權益，包括人身的隱私、資料的隱密、安全及最大利益。
2. 請遵守衛生福利部公告之『人體研究法』、『臨床試驗受試者招募原則』。
3. 應讓受試者瞭解計畫內容、簽署同意書前有充分時間考慮、及在完全自主情況下做決定，並確實保護決定能力有欠缺之受試者，將受試者所簽署之受試者同意書主動且確實交由簽署人自行保存。
4. 計畫主持人需依照本會核准之計畫書、受試者同意書及相關文件之版本執行，非經本會同意不得任意變更。
5. 若台端所提之計畫案（新案或計畫修正案）已經臺北榮民總醫院/臺中榮民總醫院/中央 IRB(c-IRB/台北三軍總醫院審查通過，亦需在本會核發『人體研究/試驗計畫同意函』後方可執行。
6. 計畫主持人依按照本會所規定之期限內繳交追蹤審查報告，每 12 個月至少一次，需在 1 個月內完成繳交。
7. 如欲展延『人體研究/試驗計畫同意函』之有效期限，計畫主持人需在『人體研究/試驗計畫同意函』到期前三個月內繳交期中報告以延長同意函之有效期限。
8. 需遵守衛生福利部於 2006 年 8 月所公告之「研究用人體檢體採集與使用注意事項」之相關規定並確實執行（請至本會網站查閱）。
9. 配合並接受本委員會實地訪查與查核監督。
10. 發生於本院之「嚴重不良事件及未預期問題」(通報範圍請見 SOP16)需於得知日起 1 個工作日內立即通報本會並於 15 天內繳交相關文件，另於 7 天內通報衛生福利部並於 15 天內繳交相關文件，本會將會送審委員。
11. 人體臨床試驗之相關法規及倫理原則可至本會網站查詢，並請遵守之。
12. 依據人體試驗管理辦法第十五條「在人體試驗期間，不得對外發表成果或為宣傳」，請留意。
13. 違反相關法規或不配合本會規範者，可能使您的臨床試驗中止或終止，並於一年內不得再申請臨床試驗計畫，且需接受數小時的「優良臨床試驗」教育訓練課程。
14. 人體試驗計畫主持人需於電子病歷上註記，以便於永久保存人體試驗病歷。
15. 當研究成果可合理預期對可辨識之檢體提供者個人健康有重大影響時，經人體研究倫理審查委員會審核且受試者(檢體提供者)選擇知悉時，計畫主持人應告知並協助提供必要之相關諮詢。
16. 受試者同意書取得注意事項:

受試者同意書應按醫療法79條規定各項詳細敘述，以善盡醫療上必要之注

意，並向受試者清楚說明，取得受試者書面同意。內容包括

(1)試驗目的及方法，包括特定病人之條件、收納方式、人數、實施方式、期間與進度、追蹤及必要之復健計畫。

(2)可預期風險及副作用。

(3)預期試驗效果。

(4)其他可能之治療方式及說明。

(5)接受試驗者得隨時撤回同意之權利。

(6)試驗有關之損害補償或保險機制。

(7)受試者個人資料之保密。

(8)受試者生物檢體、個人資料或其衍生物之保存與再利用。

受試者以有意思能力之成年人為限；但顯有益於特定人口群或特殊疾病罹患者健康權益之試驗，不在此限。若受試者為限制行為能力人，應得其本人與法定代理人同意。受試者為無行為能力人，應得其法定代理人同意。若無法定代理人，應按人體研究法第12條規定，取得關係人同意。

關係人順序為: 配偶 、成年子女、父母、兄弟姊妹、祖父母。

前項關係人所為之書面同意，得以一人行之；關係人意思表示不一致時，依前項各款先後定其順序。前項同一順序之人，以親等近者為先，親等同者，以同居親屬為先，無同居親屬者，以年長者為先。

兒童受試者若為12歲以上，必須另有兒童能閱讀之同意書，清楚說明，取得其本人及法定代理人同意。研究對象為胎兒時，同意應由其母親為之。

前項告知及書面同意，主持人應給予受試者充分時間考慮，並不得以脅迫或其他不正當方式為之。同意書應簽署二份, 主持人及受試者各持一份，受試者有隨時撤回之權利。

彰化基督教醫院
CHANGHUA CHRISTIAN HOSPITAL

變更案同意臨床試驗證明書
Clinical Trials Approval Certificate(Amendments)

135 Nanxiao St., Changhua City, Changhua County 500, Taiwan (R.O.C.)
Tel :886-4-723-8595 ext.8442
E-mail:d9065@cch.org.tw
彰化基督教醫院 Changhua Christian Hospital

500 彰化市南校街 135 號
聯絡人：洪翠霞
Contact Person : Tsui-Hsia Hung
電話：(04)723-8595 轉 8442
E-mail: d9065@cch.org.tw

計畫中文名稱：一個評估 UB-612 疫苗對於新型冠狀病毒於青少年、成年和老年健康受試者的免疫原性、安全性與耐受性的第二期、安慰劑控制、隨機分派、觀察者盲性臨床試驗

計畫主持人：劉尊榮 / 協同主持人：陳昶華、陳婉真、許瑛敉、李育霖、楊順成

試驗機構名稱：彰化基督教醫療財團法人彰化基督教醫院

研究經費來源：聯亞生技開發股份有限公司

計畫編號：V-205 / 本會編號：210304

核准日(審查通過日)：西元 2021 年 10 月 02 日

核准臨床試驗期間：西元 2021 年 10 月 02 日 至 西元 2022 年 03 月 21 日止

計畫書：v3.0, 06 September, 2021

受試者同意書：
1. 受試者同意書(成年安全確認組) : Version 2.0 Date:20210917
2. 受試者同意書(青少年安全確認組) : Version 2.0 Date:20210917
3. 受試者同意書(成年安全確認第三劑組) : Version 3rd dose 1.0, Date:20210917
4. 受試者同意書(青少年安全確認第三劑組) : Version 3rd dose 1.0, Date:20210917)

個案報告表：v4.0, 06 September, 2021

協同主持人退出試驗：陳賢孟

未預期事件或藥品嚴重不良反應通報、後續定期追蹤之程序及應注意事項，請參閱背面。

彰化基督教醫院 人體試驗委員會 Institutional Review Board Changhua Christian Hospital

彰化基督教醫院
CHANGHUA CHRISTIAN HOSPITAL

Protocol Title: A Phase II, Placebo-controlled, Randomized, Observer-blind Study to Evaluate the Immunogenicity, Safety and Tolerability of UB-612 Vaccine against COVID-19 in Adolescent, Younger and Elderly Adult Volunteers
Principal Investigator(s): Chun Eng Liu / Co Investigator：chen changhua、Wan-Chin CHEN、Ing-Moi HII、Yu-Lin Lee、Yang, Shun-Cheng
Institution: CHANGHUA CHRISTIAN HOSPITAL
Sponsor: 聯亞生技開發股份有限公司
Protocol No. : V-205 / CCH IRB No. : 210304
Date of Approval: Oct 02, 2021
Duration of Approval: from Oct 02, 2021 to Mar 21, 2022

Protocol: v3.0, 06 September, 2021
Informed Consent Form:
1. 受試者同意書(成年安全確認組) : Version 2.0 Date:20210917
2. 受試者同意書(青少年安全確認組) : Version 2.0 Date:20210917
3. 受試者同意書(成年安全確認第三劑組) : Version 3rd dose 1.0, Date:20210917
4. 受試者同意書(青少年安全確認第三劑組) : Version 3rd dose 1.0, Date:20210917)

Case Report Form: v4.0, 06 September, 2021
Co-Investigator withdraw: Hsien-Meng Chen

See the back of this page for the procedures for reporting unanticipated problems, or drug serious adverse reactions, or interim, and other important notes.

彰化基督教醫院
第一人體試驗委員會
主任委員： 蘇矢立

Sincerely Yours
ShihLi Su, Ph.D.
Chairman
Institutional Review Board Committee A
Changhua Christian Hospital, Taiwan

彰化基督教醫院 人體試驗委員會 Institutional Review Board Changhua Christian Hospital

Shih-Li Su 2021.Oct.02
(signature, date)

本會組織與執行皆符合 ICH-GCP
The Institutional Review Board performs its functions according to written Operating procedures and complies with ICH-GCP and with the applicable regulations.

未預期事件通報、後續定期追蹤之程序及應注意事項：

1. 院內受試者發生死亡或危及生命案例應該在獲知日起七天以內通報本委員會，其他非預期嚴重藥品不良反應應於十五天以內向本委員會通報。
2. 可能危害受試者安全、影響試驗執行之新發現或影響人體試驗委員會同意試驗繼續進行之新發現，須向本委員會報告。
3. 期中報告：**應於　西元 2022 年 01 月 21 日 前繳交期中報告。**
   核准有效期限屆滿，若尚未通過期中報告追蹤審查，不得繼續試驗。(計畫主持人，未依規定繳交期中報告，本會針對該研究案，於應繳交日起暫停納入新受試者，且本會得拒絕計畫主持人申請新案，並直到該期中報告繳交。)。
4. 結案報告：試驗完成後，應將執行情形及結果以書面報告本會核備。
5. 暫停或終止計畫報告：計畫完成前就暫停或停止收案與追蹤，應與書面「計畫暫停或終止摘要表」，送交本會核備。
6. 嚴重或持續不配合本委員會規範，未能遵循以上事項，可能導致您的研究計畫暫停或永久終止，並影響您未來送審計畫的權益。
7. 為了受試者安全，計畫主持人必須遵循以上之規範，以確保能繼續執行試驗。

Procedures for reporting Unanticipated Problems, or interim, and other important notes:

1. If subject(s) die(s) or hospitalized, IRB should be notified within 7 days of becoming aware of this. For other unexpected serious adverse drug reactions, IRB should be notified within 15 days.
2. If any new findings affect the safety of the participants or others, or the implementation of the study, or decision of IRB as to allowed to continuing of the study, IRB should be informed promptly.
3. Interim report: **An interim report should be submitted by Jan 21, 2022.**
   If the interim report has not been submitted by the deadline, the study must be halted. (If a principal investigator fails to submit an interim report on schedule, IRB may suspend review of other protocols submitted by the investigator, and may refuse to review any further applications made by the investigator.)
4. Final report: When the study has been completed, details of the study implementation and of the results obtained should be submitted to IRB in writing for review.
5. For any reason, the study is terminated prior to the completion of a study, the summary report should be submitted to IRB.
6. Serious or repeated failure to comply with regulations and with the above requirements may result in the study being suspended or terminated, and may affect you to submit studies for review in the future.
7. Principal investigators must follow in order to continue study procedures for the safety of the subjects.

彰化基督教醫院 人體試驗委員會 Institutional Review Board Changhua Christian Hospital

臺中榮民總醫院第一/二人體研究倫理審查委員會
Institutional Review Board I&II of Taichung Veterans General Hospital
407219 臺 中市西屯區臺灣大道四段1650號
1650 Taiwan Boulevard Sect. 4 , Taichung,Taiwan 407219 , ROC
TEL：886-4-23592525#4006 FAX: 886-4-23592525#4408
E-mail: irbtc@vghtc.gov.tw

人體研究/試驗計畫修正案許可書

開立日期：西元 2021 年 10 月 12 日

計畫名稱：一個評估 UB-612 疫苗對於新型冠狀病毒於青少年、成年和老年健康受試者的免疫原性、安全性與耐受性的第二期、安慰劑控制、隨機分派、觀察者盲性臨床試驗

試驗編號：V-205

IRB 編號：SC21074A#3

計畫主持人：感染管制中心施智源醫師

協同主持人：家庭醫學部許碧珊醫師、內科部胸腔內科楊宗穎醫師、家庭醫學部社區醫學科林鉦勝醫師、內科部感染科劉伯瑜醫師、護理部黃惠美護理長、內科部感染科林詩萍醫師、內科部呼吸治療科詹明澄醫師、內科部呼吸治療科徐國軒醫師、內科部胸腔內科黃彥翔醫師、內科部內分泌新陳代謝科王俊興醫師、兒童醫學中心兒童血液腫瘤科黃芳亮醫師

研究/試驗執行機構：臺中榮民總醫院

修正內容及版本：

1.計畫書版本及日期：Version 3.0, Date：06 Sep, 2021

2.中文摘要版本及日期：Version 3.0, Date：06 Sep, 2021

3.英文摘要版本及日期：Version 3.0, Date：06 Sep, 2021

4.受試者同意書版本及日期：

- 成年安全確認組：Version 3.0, Date：17 Sep, 2021
- 成年安全確認第三劑組：Version 1.0, Date：17 Sep, 2021
- 青少年安全確認組：Version 3.0, Date：17 Sep, 2021
- 青少年安全確認第三劑組：Version 1.0, Date：17 Sep, 2021

5.個案報告表版本及日期：Version 4.0, Date：06 Sep, 2021

通過會期：第一人體研究倫理審查委員會第 110-A-11 次會議

有效期限：2022 年 03 月 11 日

（此案追蹤審查頻率為半年一次，請主持人主動繳交追蹤審查報告。）

＊ 依照赫爾辛基宣言及 ICH-GCP 規定，臨床試驗每屆滿一年，人體研究倫理審查委員會必須定期重新審查臨床試驗後方可繼續進行。請於有效期限到期二個月前繳交追蹤審查報告以利本會進行審查。
＊ 受試者於試驗期間發生嚴重不良事件及疑似未預期之嚴重藥物不良反應，主持人應依衛生福利部法規於期限內通報主管機構及審查之人體研究倫理審查委員會。
＊ 計畫展延應於許可書期限截止前二個月提出申請。
＊ 結案報告應於許可書期限截止後三個月內繳交。
＊ 本會有暫停/終止本研究計畫及撤銷本執行許可書之權責。

臺中榮民總醫院第一人體研究倫理審查委員會

主任委員 林志堅

臺中榮民總醫院第一人體研究倫理審查委員會 （印）

林志堅

本會組織與執行皆符合國際醫藥法規協合會之藥品優良臨床試驗規範
TCVGH-IRB is organized and operates according to ICH-GCP and the applicable laws and regulations.

臺中榮民總醫院第一/二人體研究倫理審查委員會
Institutional Review Board I&II of Taichung Veterans General Hospital
407219 臺 中市西屯區臺灣大道四段1650號
1650 Taiwan Boulevard Sect. 4 , Taichung,Taiwan 407219 , ROC
TEL：886-4-23592525#4006 FAX: 886-4-23592525#4408
E-mail: irbtc@vghtc.gov.tw

Certificate of Protocol Amendment

Date of Approval:12 Oct,2021

Protocol Title： A Phase II, Placebo-controlled, Randomized, Observer-blind Study to Evaluate the Immunogenicity, Safety, and Tolerability of UB-612 Vaccine against COVID-19 in Adolescent, Younger and Elderly Adult Volunteers

Protocol No.：V-205

TCVGH-IRB No.：SC21074A#3

Principal Investigator：Zhi-Yuan Shi

Sub-Investigator：Pi- Shan Hsu, Tsung-Ying Yang, Chu-Sheng Lin, Po-Yu Liu, Hui-Mei Huang, Shih-Ping Lin, Ming-Cheng Chan, Kuo-Hsuan Hsu, Yen-Hsiang Huang, Jun-Sing Wang, Fang-Liang Huang

Institute：Taichung Veterans General Hospital

Reason for Amendment & Version：

1.Protocol Version & Date：Version 3.0, Date：06 Sep, 2021

2.Protocol Synopsis Chinese Version & Date：Version 3.0, Date：06 Sep, 2021

3.Protocol Synopsis English Version & Date：Version 3.0, Date：06 Sep, 2021

4.Informed Consent Form Version & Date：

- Safety check group - Adult：Version 3.0, Date：17 Sep, 2021
- 3rd dose Safety check group - Adult：Version 1.0, Date：17 Sep, 2021
- Safety check group - Adolescent：Version 3.0, Date：17 Sep, 2021
- 3rd dose Safety check group - Adolescent：Version 1.0, Date：17 Sep, 2021

5.Case Report Form Version & Date：Version 4.0, Date：06 Sep, 2021

Board Meeting：Institutional Review Board (I) 110-A-11 Board Meeting

Approval Effective Period：11 March 2022

Frequency of Continuing Review：6 months

＊ In accordance with Declaration of Helsinki and ICH-GCP guidelines, PI is responsible for submitting a progress report to IRB two months prior to the expiry date for an annual review.
＊ Serious adverse events or SUSAR involving risk to participants must be reported to Ministry of Health and Welfare (MOHW) and IRB according to current regulation.
＊ Application for protocol extension should be submitted to IRB two months before the expiry date of the Certificate of Approval.
＊ Closing report should be submitted to IRB within three months after the expiry date of the Certificate of Approval.
＊ The IRB has authorization to suspend/terminate the protocol and to withdraw the Certificate of Approval.

Chih-Chien Lin, MD, MPH
Chair, Institutional Review Board (I), TCVGH

臺中榮民總醫院 第一人體研究倫理審查委員會 臺中榮民總醫院

Chih-Chien Lin

臺北醫學大學

臺北醫學大學暨附屬醫院聯合人體研究倫理委員會

TMU-Joint Institutional Review Board

通過證明函 – 修正案(簡易審查-實質變更)

開立日期：民國110年10月07日

本會編號：N202102039

計畫名稱：一個評估UB-612疫苗對於新型冠狀病毒於青少年、成年和老年健康受試者的免疫原性、安全性與耐受性的第二期、安慰劑控制、隨機分派、觀察者盲性臨床試驗

計畫主持人：李垣樟

共同主持人：劉明哲、邱仲峯、張君照、曾頌惠、許信德、吳建志、李欣倫、黃姚儒、周百謙、鍾啟禮、徐上富、蕭世欣、林哲玄、黃宇銳、侯甚光、陳揚卿、莊涵琄、羅詩修、陳偉傑、劉欣怡

研究人員：周鳳儀、李盈蓁、余嘉莉、黃金燕、王昌弘、丁充逢、李心平、黃珮妤、蔡家榛、蔡宛均、劉家豪、鄭錦龍、陳美雪、楊純弦、王凱立、巫怡蓮、連瑋豪、林穎、張玟惠、王楚君、廖嘉甄、陳蘊書、張家綺、蔡宛玲、謝俊宇、涂儷蓉、賴珍伶、謝清心、徐珮娟、方敏晏

試驗/研究機構：臺北醫學大學附設醫院

申請書版本/日期：Version 3.2 Date:20210930

計畫書版本/日期：Version 3.0, Date: 20210906

中文摘要：Version 3.0, Date: 20210906

受試者同意書版本/日期：

- (成年免疫組)：Version 2.0, Date:20210914
- (青少年免疫組)：Version 2.0, Date:20210914
- (成年安全確認組)：Version 2.0, Date:20210914
- (青少年安全確認組)：Version 2.0, Date:20210914
- (成年免疫第三劑組)：Version 1.0, Date:20210914
- (青少年免疫第三劑組)：Version 1.0, Date:20210914
- (成年安全確認第三劑組)：Version 1.0, Date:20210914
- (青少年安全確認第三劑組)：Version 1.0, Date:20210914

個案報告表版本/日期：Version 4.0, Date: 20210906

緊急解盲指引：Version 1.2, Date:20210722

Note to File：Date: 20210324

上述計畫之修正，將於第110-10-4次會期核備(會議日期：110年10月21日)，特此證明。有效期限自民國110年10月07日至民國111年02月16日。試驗/研究期間應接受本會之監督。

臺北醫學大學暨附屬醫院聯合人體研究倫理委員會
Taipei Medical University
Joint Institutional Review Board

依據衛生福利部與相關規定， 後續追蹤程序及要求如下：

1. 期中報告：本計畫期中繳交頻率為**每6個月**，應於有效期限到期**前二個月（民國110年12月16日）**繳交期中報告。有效期限屆滿時若尚未通過期中報告與效期展延審查者，試驗/研究不得繼續執行。
2. 結案報告：試驗/研究完成後，應將執行情形及結果依結案報告表要求送至本會審查。**核准期間到期三個月**仍未繳交者，**本會得撤銷本通過證明函，亦即撤銷本試驗/研究之核准，亦將依本會作業程序暫停主持人(含任何參與形式)申請新試驗/研究案之審查三個月**。
3. 嚴重不良事件(SAE)報告：執行人體試驗或臨床試驗之主持人應根據衛生福利部「藥品優良臨床試驗準則」和「嚴重藥物不良反應通報辦法」規定，辦理相關事宜。

主任委員：

陳中明

臺北醫學大學暨附屬醫院
聯合人體研究倫理委員會
Taipei Medical University
Joint Institutional Review Board

Taipei Medical University

Certificate of TMU-JIRB Approval

Issue Date: 2021/10/07

TMU-JIRB No.: N202102039

Protocol Title: A Phase II, Placebo-controlled, Randomized, Observer-blind Study to Evaluate the Immunogenicity, Safety and Tolerability of UB-612 Vaccine against COVID-19 in Adolescent, Younger and Elderly Adult Volunteers

Principal Investigator: Yuarn-Jang Lee

CO- Investigator: Mingche Liu, Jeng-Fong, Chiou, Chun-Chao CHANG, TSENG,SUNG-HUI, Hsu, Hsin-Te, WU CHIEN-CHIH, Lee, Hsin-Lun, Yaoru Huang, Pai-Chien Chou, CHI-LI CHUNG, Shang-Fu Hsu, SHIH-HSIN HSIAO, Lin, Che-Hsuan, Yu-Jui Huang, Hou, Sen-Kuang, Chen YangChing, Chuang HanChuan, Lo shih hsiu, Chen, WeiChieh, Hsin-Yi LIU

Study Member: Fengyi Chou, Ying-Chen Lee, CHIA-LI YU, CHIN-YEN HUANG, Chang-Hung Wang, CHUNG-FENG TING, Sin Ping Lee, peiyuhuang, tsai chia chen, Wan-Chun Tsai, Chia-Hao, Liu, Chin-Lung Cheng, Mei-Syue, Chen, Chun-Hsien Yang, WANG KAI LI, WU YI LIEN, LIEN WEI HAO, Ying Lin, WEN-HUICHANGE, NO, Liao chian chen , YUN-SHU CHEN , Chia-Chi,Chang, Wan-Ling Tsai, Chun-Yu Hsieh, Tu LI Jung, Jhen-Ling Lai, HSIEH CHING HSIN, Pei Chuan Hsu, min yan hang

Study Site:Taipei Medical University Hospital

Application Form:Version 3.2 Date:20210930

Protocol Version/Date:Version 3.0, Date: 20210906

Chinese Synopsis：Version 3.0, Date: 20210906

Informed Consent Forms:

- (Immunogenicity Group -Adult):Version 2.0, Date:20210914
- (Immunogenicity Group -Adolescent):Version 2.0, Date:20210914
- (Safety Check Group -Adult):Version 2.0, Date:20210914
- (Safety Check Group -Adolescent):Version 2.0, Date:20210914
- (3rd Dose Immunogenicity Group -Adult):Version 1.0, Date:20210914
- (3rd Dose Immunogenicity Group -Adolescent):Version 1.0, Date:20210914
- (3rd Dose Safety Check Group -Adult):Version 1.0, Date:20210914
- (3rd Dose Safety Check Group -Adolescent):Version 1.0, Date:20210914

Case Report Forms:Version 4.0, Date: 20210906

Emergency Code Breaking Instruction：Version 1.2, Date:20210722

Note to File：Date: 20210324

The amendment of above study will be approve by the TMU-Joint Institutional Review Board in meeting #110-10-4(date:2021/10/21), duration of validity is from 2021/10/07 to 2022/02/16, and must be monitored by TMU-JIRB.

臺北醫學大學暨附屬醫院聯合人體研究倫理委員會
Taipei Medical University
Joint Institutional Review Board

According to Ministry of Health and Welfare and the relevant regulations, follow-up procedures and requirements are as below:

1. Continuing Report: Frequency of the report of this trial/study is every 6 month, and should be submitted to TMU-JIRB for review 2 months prior to expiry date (2021-12-16) or the trial/study must be pending.
2. Final Report: The report should be submitted to TMU-JIRB for review once completed TMU-JIRB may withdraw the approval of the trial/study if the report didn't submitted within 3 months from the date of validity and will suspend PI from new application for 3 months per TMU-JIRB SOPs.
3. SAE: Serious Adverse Event(s) (SAE) Report: SAE report(s) should be submitted to related authorities according to "Regulations for Good Clinical Practice" as well as "Procedures for Reporting Serious Adverse Drug Reaction" by MOHW.

Chairman:

臺北醫學大學暨附屬醫院
聯合人體研究倫理委員會
Taipei Medical University
Joint Institutional Review Board

本會組織與執行皆符合適用法規
The TMU-Joint Institutional Review Board performs its functions according to written operating procedures and complies with GCP and with the applicable regulatory requirements.
b1387e754a64a19473e19b3210d96511 TMU-JIRB Form072/20200317

醫療財團法人徐元智先生醫藥基金會
亞東紀念醫院人體試驗審議委員會
Research Ethics Review Committee
Far Eastern Memorial Hospital
21, Sec. 2, Nanya S. Rd., Banciao Dist., New Taipei City 220, Taiwan (R.O.C.)
Tel：(02)7728-2152　Fax：(02)7728-1592
Email：irb@mail.femh.org.tw

試驗/研究變更許可書

案件編號：110025-I　　　　計畫編號：V-205
計畫名稱：一個評估 UB-612 疫苗對於新型冠狀病毒於青少年、成年和老年健康受試者的免疫原性、安全性與耐受性的第二期、安慰劑控制、隨機分派、觀察者盲性臨床試驗
試驗/研究機構：醫療財團法人徐元智先生醫藥基金會亞東紀念醫院
試驗/研究委託者：聯亞生技開發股份有限公司
計畫主持人：廖俊星
協同主持人：陳泓恩、楊家瑞
研究成員：徐心穗、張嫚萱
試驗/研究人數：400
試驗/研究期限：2021 年 2 月 23 日至 2022 年 12 月 31 日
追蹤審查頻率：六個月

有效期限至二○二二年二月二十二日。(請於有效期限到期二個月前繳交持續審查報告以利本會進行審查)

主任委員 **張淑雯** [signature]

Permission of continuing review of Clinical Trial/ Research

FEMH No.: 110025-I　　　　Protocol No.: V-205
Protocol Title: A Phase II, Placebo-controlled, Randomized, Observer-blind Study to Evaluate the Immunogenicity, Safety and Tolerability of UB-612 Vaccine against COVID-19 in Adolescent, Younger and Elderly Adult Volunteers
Trial/Research Institution: Far Eastern Memorial Hospital
Sponsor: United Biomedical, Inc., Asia
Principal investigator: Chun-Hsing Liao
Sub- investigator: Hong-An Chen, Chia-Jui Yang
Study Coordinator: Hsin-Sui Hsu, Man-Hsuan Chang
Number of subjects: 400
Trial period: February 23, 2021 to December 31, 2022
Continuing review frequency: Six months

The protocol is valid till February 22, 2022. (The investigator is required to apply for a continuing review

核准日期/ Approved date：2021-11-10

本委員會的運作符合優良臨床試驗準則及政府相關法律規章
The committee is organized under, and operates in accordance with, the Good Clinical Practice guidelines and governmental laws and regulations

醫療財團法人徐元智先生醫藥基金會 亞東紀念醫院 人體試驗審議委員會 TEL.(02)7728-2152

not less than two months prior to approval expiration date.)
Shu-Wen Chang M.D., Professor of Ophthalmology
Chairman
Research Ethics Review Committee

修正內容/版本 Reason for Amendment/Version:

- 計畫書版本 Protocol Version: Version 3.0, Date: 20210906
- 受試者說明及同意書版本 Informed Consent Form:
  (1.) 青少年免疫組 Immno young：Version 2.0, Date: 20211021
  (2.) 青少年安全確認組 non-Immno young：Version 2.0, Date: 20211021
  (3.) 成年免疫組 Immno adult：Version 2.0, Date: 20211021
  (4.) 成年安全確認組 non-Immno adult：Version 2.0, Date: 20211021
- 中文摘要 Chinese synopsis: Version 3.0, Date: 20210906
- 英文摘要 English synopsis: Version 3.0, Date: 20210906
- 個案報告表 Case report form: Version 4.0, Date: 20210906
- 新增受試者說明及同意書版本 Add Informed Consent Form:
  (1.) 青少年免疫第三劑組 Immno young 3rd dose：Version 3rd dose 1.0, Date: 20211013
  (2.) 青少年安全確認第三劑組 non-Immno young 3rd dose：Version 3rd dose 1.0, Date: 20211013
  (3.) 成年免疫第三劑組 Immno adult 3rd dose：Version 3rd dose 1.0, Date: 20211013
  (4.) 成年安全確認第三劑組 non-Immno adult 3rd dose：Version 3rd dose 1.0, Date: 20211013

已通過其它合法審查會之審查案件，符合得簡易程序審查範圍，不予入會
The protocol fits the criteria for Expedited Review and do not need to be discussed in this meeting.

上述計畫已於本院人體試驗審議委員會審查，同意人體試驗/研究進行
The protocol has been approved by the Research Ethics Review Committee of the Far Eastern Memorial Hospital.

核准日期/ Approved date：2021-11-10

本委員會的運作符合優良臨床試驗準則及政府相關法律規章
The committee is organized under, and operates in accordance with,
the Good Clinical Practice guidelines and governmental laws and regulations

Appendix 9

中國醫藥大學暨附設醫院
受試者同意書
（成年免疫組）

您被邀請參與此研究。此同意書主要是提供您本研究之相關資訊，以便您決定是否參加本研究。計畫主持人或其指定之研究人員會為您說明研究內容並回答您的疑問。您可以提出任何和此研究有關的問題，在您的問題尚未獲得滿意的答覆之前，請不要簽署此同意書。如果您願意參與本研究，此文件將視為您的同意紀錄。即使在您同意後，您可以隨時退出本研究不需任何理由。

計畫名稱<br>中文：一個評估 UB-612 疫苗對於新型冠狀病毒於青少年、成年和老年健康受試者的免疫原性、安全性與耐受性的第二期、安慰劑控制、隨機分派、觀察者盲性臨床試驗<br>英文：A Phase II, Placebo-controlled, Randomized, Observer-blind Study to Evaluate the Immunogenicity, Safety, and Tolerability of UB-612 Vaccine against COVID-19 in Adolescent, Younger and Elderly Adult Volunteers	
執行單位：中國醫藥大學附設醫院感染科、家庭醫學科	委託單位/藥廠：聯亞生技開發股份有限公司<br>研究經費來源：聯亞生技開發股份有限公司<br>受託研究機構：晉加股份有限公司
計畫主持人：黃高彬	職稱：主治醫師
協同主持人：林文元	職稱：主治醫師
協同主持人：林伯昌	職稱：主治醫師
緊急聯絡人：黃高彬	電話：0975-681-950
受試者姓名：<br>性別：<br>身分證字號：<br>通訊地址：	病歷號碼：<br>出生日期：<br>聯絡電話：
法定代理人或有同意權人之姓名：<br>性別：<br>身分證字號：<br>通訊地址：	與受試者關係：<br>出生日期：<br>聯絡電話：
(一)試驗簡介：<br>1. 本品/技術資料：	

中國醫藥大學暨附設醫院

受試者同意書

（成年免疫組）

新型冠狀病毒(SARS-CoV-2)於2019年12月起造成中國湖北省武漢市發現多起病毒性肺炎群聚，隨後於2020年1月底台灣出現第一起境外移入確診個案。此疾病在全球擴散，世界衛生組織宣布將此疫情為「國際關注公共衛生緊急事件」。截至2020年底，全球僅有數間疫苗公司，如美國輝瑞藥廠等，取得緊急使用授權上市。

UB-612疫苗為聯亞生技開發股份有限公司所開發新型冠狀病毒預防性疫苗，疫苗含病毒棘狀融合蛋白和胜肽片段，可產生高親和力抗體與新型冠狀病毒結合，並誘發細胞免疫反應，進而達到預防新型冠狀病毒的感染。

UB-612第一期延伸性試驗顯示，接種第三劑UB-612疫苗可以誘發極高的中和抗體，在目前變種病毒的威脅之下，施打第三劑加強免疫反應，已是許多國家的選擇。

2. 本品上市狀況：

本品仍應用於人體試驗，尚未在我國上市。

3. 本試驗使用的UB-612疫苗對新型冠狀病毒的預防效果仍未確認。

(二)試驗目的：

主要試驗目的

- 評估UB-612疫苗誘發的新型冠狀病毒中和抗體效價。
- 評估接種UB-612疫苗後的安全性和耐受性。

次要試驗目的

- 評估在試驗期間對於新型冠狀病毒的免疫反應。
- 評估三批獨立批次疫苗的批次免疫一致性。

探索性試驗目的

- 評估UB-612疫苗誘發的T細胞功能。
- 評估UB-612疫苗在年輕受試者的安全性和免疫原性。
- 評估UB-612疫苗的療效。
- 描述UB-612疫苗於確診和/或嚴重感染新型冠狀病毒案例之血液學反應。
- **評估針對SARS-CoV-2抗原的抗體反應。**

(三)試驗之主要納入與排除條件:

中國醫藥大學暨附設醫院執行本研究計畫的醫師或相關研究人員將會與您討論有關參加本研究的必要條件。請您配合必須誠實告知我們您過去的健康情形,若您有不符合參加本研究的情況,將不能參加本研究計畫。

1. 參加本研究計畫的主要條件:

☐(1) 您為納入試驗時20~85歲之間健康男性或未懷孕的女性受試者。

☐(2) 您為具生育能力的女性與男性應於首次接種疫苗至最後一次疫苗後3個月同意進行有效的避孕方式。可接受的有效避孕方式包括:

☐a. 男性或女性以手術方法絕育、植入式避孕、或子宮避孕器。

☐b. 注射避孕、避孕藥、避孕貼片、避孕環加上一種屏障避孕法*。

☐c. 合併使用兩種屏障避孕法*。

*有效的屏障避孕法為避孕隔膜、男性或女性保險套、避孕海綿或殺精劑(含可殺精化學物質的藥膏或凝膠)。

☐(3) 您能理解受試者同意書內容的說明與可能的風險,提供簽名的受試者同意書。

☐(4) 您能夠理解與遵從本試驗程序與能夠參與每次訪視。

☐(5) 您的耳溫≤ 38.0°C。

☐(6) 您依據醫療病史、身體檢查和試驗主持人的臨床判斷為健康受試者**可符合納入試驗資格。經試驗主持人判斷,即便您的病史穩定或且控制良好,但伴隨病情惡化而有提高嚴重新型冠狀病毒感染的風險。

**健康受試者有先前存在的穩定疾病者可以納入試驗,定義為該疾病在納入試驗前12週內沒有惡化至需要治療或住院的顯著變化和在納入試驗6個月內沒有惡化至需要治療或住院的顯著變化。

2. 若您有下列任一情況,您將無法參加本研究計畫:

☐(1) 您有接種疫苗後需要醫療介入的過敏性休克、蕁麻疹或其他顯著不良反應的病史。

☐(2) 您在篩選時或接種每劑疫苗前已懷孕女性或懷孕檢測為陽性的女性。

☐(3) 您為正在哺乳的女性,或計畫從接種第一劑疫苗至最後一劑疫苗後60天哺乳的女性。

☐(4) 您在接種第一劑疫苗前3天內，經試驗主持人判斷，患有任何急性疾病。

☐(5) 您在接種第一劑疫苗前1個月內有重大手術。

☐(6) 您是已知為人類免疫缺乏病毒抗體陽性。

☐(7) 您是已知為活動性B型肝炎或C型肝炎。活動性肝炎定義為肝臟轉胺酶(天門冬胺酸轉胺酶和/或丙胺酸轉胺酶)大於3倍正常值上限和/或總膽紅素大於3倍正常值上限。

☐(8) 您是已知曾暴露於新型冠狀病毒，或曾接受預防新型冠狀病毒、中東呼吸症候群冠狀病毒、嚴重急性呼吸道症候群的試驗或已上市產品。

☐(9) 您有格林-巴利症候群的病史。

☐(10) 您在簽署受試者同意書前12周內參與其他的臨床試驗。

☐(11) 您為免疫缺乏/失調疾病，無論是否由基因缺陷、免疫缺乏症或免疫抑制療法所造成。

☐(12) 您計畫或正在進行抗癌症治療。

☐(13) 您患有血小板異常或其他凝血異常可能造成注射之禁忌症。

☐(14) 您在接種第一劑疫苗前6個月長期接受(≥14天連續使用)免疫抑制劑、皮質類固醇(相當於一天使用≥ 20 mg強的松(prednisone))或細胞毒性治療。

☐(15) 您在接種第一劑疫苗前4個月接受免疫球蛋白和/或任何血液製劑的治療。

☐(16) 您在接種試驗疫苗前14天接種任何季流感疫苗或新型流感疫苗，或前28天接種其他疫苗。

☐(17) 您預期在接種試驗疫苗後14天接種任何季流感疫苗或新型流感疫苗，或後28天接種其他疫苗。

☐(18) 您使用短期(<14天使用)全身性類固醇。應於中斷使用全身性類固醇至少28天後才可使用試驗疫苗。吸入/噴霧性、關節注射、囊內或局部(皮膚或眼用)類固醇可允許使用。

☐(19) 您在篩選期前3個月失血或捐血超過500毫升，或預計在試驗期間內捐血或輸血。

☐(20) 經試驗主持人判斷，您有任何醫療疾病或狀況，可能會影響試驗結果或參與試驗可能會對受試者引發額外風險。

☐(21) 您是直接參與本試驗執行的試驗主持人所屬機構**的試驗團隊**、試驗委託者或受託

研究機構(CRO)的員工。

（四）試驗方法及相關檢驗：

這是一個第二期、觀察者盲性、多中心、隨機分派、安慰劑控制試驗，以評估青少年，成年和老年受試者使用兩劑UB-612疫苗的免疫原性，耐受度和安全性。有一部份的受試者使用UB-612疫苗，而另外一部份的受試者則使用「安慰劑」。所謂「安慰劑」是不含有效成份的疫苗。至於誰使用試驗用藥或誰使用「安慰劑」，則像丟銅板或擲骰子一樣由機率決定，不管是您或是研究醫師都不知道您使用了那一種藥，只有分發跟施打疫苗的試驗人員才知道您使用哪一種疫苗，這叫做觀察者盲性。

總計約有3850位合格成年受試者組成核心組用於申請緊急使用授權，另外大約有385位青少年受試者組成補充組申請額外適應症。所有受試者將以6:1的比例，隨機分派至兩劑100微克劑量組別和安慰劑組，包括462位大於18歲至小於65歲可評估的受試者進入批次分析組。對於免疫分析，至少包括350位可評估的成年受試者(年齡大於18歲至小於65歲)和154位可評估的老年受試者(年齡≥65歲)進行描述性分析。免疫原性的受試者將會先納入試驗。所有的受試者將會納入安全性分析，其中至少770位隨機分派的受試者為≥65歲的分層。青少年組將在核心組招募完畢後，再開始納入試驗。約有385位青少年受試者將以6:1的比例隨機分派，其中包括154位可評估的青少年受試者將收集免疫原性數據，並和成年及老年受試者數據進行比較。

您若參與這個試驗，則為有進行免疫分析檢測的免疫原性或批次一致組。

試驗總共有8個訪視。若您參與本試驗，則至少包括第一次訪視(篩選訪視)、第二次訪視（第1天，基礎值，隨機分派，第一次接種疫苗)、第三次訪視(第29天，第二次接種疫苗)、第四次訪視(第57天)、第五次訪視(第197天)。

在第五次訪視時，預計將進行個別解盲。解盲後，得知您為施打疫苗的受試者，且您有意願且符合資格接種第三劑疫苗，將請您簽署另外一份受試者同意書，以進行後續程序；包含第六次訪視(第197~242天，第三次接種疫苗)，第七次訪視(接種疫苗後第14天)，及第八次訪視(第365天)。

若個別解盲後，得知您為施打疫苗的受試者，您不願意進行第三劑疫苗接種，將只進行第八次訪視(第365天)的安全性及抗體效價的追蹤。

若個別解盲後，得知您為施打安慰劑組的受試者，將結束您的試驗。

整個試驗期間，將預期您將參與試驗最長達13個月。

注意事項

中國醫藥大學暨附設醫院
受試者同意書
（成年免疫組）

1. 如果您同意參加本試驗，研究人員會請您簽署本份受試者同意書，並確認您符合參加本試驗的條件。
2. 從您參與試驗的當天開始，每次訪視都將有合格的試驗人員執行試驗流程與聯繫。
3. 若您有任何符合新型冠狀病毒感染的定義(您曾於過去 7 天內有出國，或是接觸疑似或確認武漢肺炎之病人，而有下列症狀: 發燒、開始咳嗽或惡化、開始呼吸急促或惡化、寒顫、開始肌肉疼痛或惡化、喉嚨痛、腹瀉、嘔吐、開始味覺/嗅覺異常)，**請依照中央疫情指揮中心規定進行自主健康管理或至指定院所進行篩檢。**
4. 若您在試驗期間感染新型冠狀病毒，將依法通報主管機關。

   您是否同意? ☐是 ☐否

   簽名：____________日期：______________
5. 於試驗期間，您不論任何理由提前退出試驗，試驗研究人員都將安排您完成最後一次的訪視之所有試驗項目。您有權利拒絕此項安排，您的決定不會引起任何影響日後醫師對您的醫療照護。

試驗步驟

第一次訪視(第-28~-1 天) -篩選訪視

在試驗醫師或試驗研究人員為您提供足夠的試驗資訊，並確保您有充分的時間考慮以及詢問任何問題後，您願意讓您參與本試驗，並由您簽署本受試者同意書。在確認您已完成受試者同意書簽署並且您也保有一份副本後，試驗醫師或試驗研究人員將會進行以下試驗程序：

(1) 記錄您簽署受試者同意書的日期
(2) 為您指定一組受試者篩選編號
(3) 確認您是否符合本試驗的納入排除條件
(4) 收集您的個人基本資料（例如生日、年齡及性別）
(5) 記錄您的醫療/用藥病史
(6) 進行身體檢查，包括身高體重
(7) 確認生命徵象
(8) 進行心電圖檢測
(9) 收集尿液檢體進行尿液檢測

(10) 收集血液檢體(共 **15.5** 毫升)，進行下列檢測:

- 常規血液檢測
- 血液生化學檢測
- 免疫學檢測**(抗核抗體)**
- **備血(作為測量新型冠狀病毒血清抗體和相關研究之用)**

第二次訪視(第 1 天)-基礎值，確認符合試驗條件，第一次接種疫苗

(1) 再度確認您是否符合本試驗的納入排除條件
(2) 隨機分派，給予您一組隨機分派號碼
(3) 記錄您的醫療/用藥病史
(4) 進行身體檢查
(5) 確認生命徵象
(6) 進行尿液懷孕檢測（具有生育能力女性）
(7) 若您有產生第三級以上的高血壓，您將被收集尿液檢體以檢測是否有蛋白尿存在或惡化
(8) 收集血液檢體(若不進行 T 細胞檢測，共 20 毫升)，進行下列檢測:

- 免疫**原性**檢測，包括 Anti-S1-RBD 免疫球蛋白 G 濃度，和新型冠狀病毒中和抗體效價
- 若您願意，將進行 T 細胞檢測(需額外抽血 56 毫升)

您是否同意? ☐是 ☐否

簽名：__________日期：____________

(9) 進行第一次疫苗接種(注射部位為非慣用手，採用肌肉注射方式)。接種疫苗後，受試者應留在試驗地點至少 30 分鐘，監測生命徵象和急性過敏症狀。
(10) 將詳細地指導您如何填寫電子日誌卡(包括注射後 7 天內預期性不良事件，14 天內的皮膚過敏反應日誌卡)
(11) 收集併用藥物/治療

第一次電話安全性追蹤(第 8, 15, 22 天)

將與您電話聯繫，以追蹤未預期不良事件和新型冠狀病毒感染症狀。

第三次訪視(第 29± 3 天)-第二次接種疫苗

(1) 進行第二次接種評估(有可能會延遲接種時間)
(2) 進行身體檢查

(3) 確認生命徵象

(4) 進行尿液懷孕檢測（具生育能力的女性）

(5) 若您有產生第三級以上的高血壓，您將被收集尿液檢體以檢測是否有蛋白尿存在或惡化。

(6) 若您為批次一致/免疫原組，將收集血液檢體(共 5 毫升)，進行下列檢測:

- 特定抗原 Anti-S1-RBD 抗體

(7) 進行第二次疫苗接種。

(8) 接種疫苗後，受試者應留在試驗地點至少 30 分鐘，監測生命徵象和急性過敏症狀。

(9) 將詳細地指導您如何填寫電子日誌卡(包括注射後 7 天內預期性不良事件，14 天內的皮膚過敏反應日誌卡)

(10) 收集併用藥物/治療

(11) 記錄上一次訪視至此次訪視之間的不良事件、嚴重不良事件型或新型冠狀病毒感染症狀

第二次電話安全性追蹤(第 36, 43 天)

將與您電話聯繫，以追蹤未預期不良事件和新型冠狀病毒感染症狀。

第四次訪視(第 57±3 天) –追蹤訪視

(1) 進行身體檢查

(2) 確認生命徵象

(3) 若您有產生第三級以上的高血壓，您將被收集尿液檢體以檢測是否有蛋白尿存在或惡化。

(4) 收集血液檢體(若不進行 T 細胞檢測，共 35 毫升) ，進行下列檢測:

- 免疫**原性**檢測，包括 Anti-S1-RBD 免疫球蛋白 G 濃度、和新型冠狀病毒中和抗體效價
- 若您願意，將進行 T 細胞檢測(需額外抽血 56 毫升)
- 常規血液檢測
- 血液生化學檢測
- 免疫學檢測**(抗核抗體)**
- **備血(作為測量新型冠狀病毒血清抗體和相關研究之用)**

(5) 收集併用藥物/治療

(6) 記錄上一次訪視至此次訪視之間的不良事件、嚴重不良事件或新型冠狀病毒感染症狀

(7) 在此次訪視後，您將每周接獲訊息提醒，以定期監測新型冠狀病毒感染症狀

第三次電話安全性追蹤(第 64, 71, 78, 85 天)

將與您電話聯繫，以追蹤安全性及新型冠狀病毒感染症狀。

第五次訪視(第 197±15 天) –個別解盲

(1) 進行身體檢查
(2) 確認生命徵象
(3) 若您有產生第三級以上的高血壓，您將被收集尿液檢體以檢測是否有蛋白尿存在或惡化。
(4) 將替您個別解盲，告知您接種到疫苗或安慰劑。若您為安慰劑組，您已完成本試驗，將協助您退出試驗。但若您為疫苗組且符合資格，我們將提供另外一份受試者同意書，邀請您參與後續的第三劑接種試驗。若您不同意接種第三劑，您仍需進行後續的安全性及抗體效價的追蹤。
(5) 收集血液檢體(共 20 毫升) ，進行下列檢測:
- 免疫**原性**檢測，包括 Anti-S1-RBD 免疫球蛋白 G 濃度、和新型冠狀病毒中和抗體效價

(6) 收集併用藥物/治療
(7) 記錄上一次訪視至此次訪視之間的不良事件、嚴重不良事件或新型冠狀病毒感染症狀

第四次電話安全性追蹤(第 253, 309 天)

第 197 天後將每兩個月與您電話聯繫，以追蹤安全性及新型冠狀病毒感染症狀。

最後一次訪視(第 365±45 天) –第 12 個月追蹤

(1) 確認生命徵象
(2) 收集血液檢體(共 20 毫升) ，進行下列檢測:
- 免疫**原性**檢測，包括 Anti-S1-RBD 免疫球蛋白 G 濃度、和新型冠狀病毒中和抗體效價

(3) 記錄上一次訪視至此次訪視之間的不良事件，包括特殊不良事件、醫療不良事件、嚴重不良事件或新型冠狀病毒感染症狀

受試者之檢體(含其衍生物)之保存、使用與再利用：

1. 檢體及剩餘檢體之保存與使用

(1) 檢體(含其衍生物)之保存與使用

為研究所需，我們所蒐集您的檢體，將依本研究計畫使用，檢體將保存於**聯亞生技開發股份有限公司(試驗委託者)**，直至20年保存期限屆滿，我們將依法銷毀。為了保護您的個人隱私，我們將以一個試驗編號來代替您的名字及相關個人資料，以確認您的檢體及與相關資料受到完整保密。如果您對檢體的使用有疑慮，或您有任何想要銷毀檢體的需求，請立即與我們聯絡(聯絡人：黃高彬醫師電話：0975-681-950)，我們即會將您的檢體銷毀。您也可以聯繫中國醫藥大學暨附設醫院研究倫理委員會(電話：04-22052121 轉1925、1926)，以協助您解決檢體在研究使用上的任何爭議。

(2) 剩餘檢體(含其衍生物)之再利用

您的生物檢體將會以專屬號碼進行編碼並在聯亞生技開發股份有限公司(試驗委託者)的控管下儲存最長20年，以研究UB-612 疫苗反應者的生物標記，及改善治療方式。

所有新的研究計畫都要再經由中國醫藥大學暨附設醫院研究倫理委員會審議通過，倫理審查委員會若認定新的研究超出您同意的範圍，將要求我們重新得到您的同意。

是否同意剩餘檢體保留提供未來新型冠狀病毒感染研究之用，並授權中國醫藥大學暨附設醫院研究倫理委員會審議是否需要再取得您的同意(擇一)

☐ 不同意保存我的剩餘檢體，試驗結束後請銷毀

☐ 同意以非去連結之方式保存我的剩餘檢體，逾越原同意使用範圍時，需再次得到我的同意才可使用我的檢體進行新的研究

2. 檢體及剩餘檢體之部分類型(檢體類型可依計畫書內容自行增減)

(1) 一般生化、血液檢驗/病毒檢測檢體

在試驗期間，會將您的檢體送往聯亞生技開發股份有限公司(試驗委託者)委託的中央實驗室中國醫藥大學暨附設醫院，此機構地址為台中市北區育德路2號，和大安聯合醫事檢驗所，此機構地址為台北市大安區復興南路二段151巷33號，中央實驗室會在分析後立即將分析結果提供給試驗中心，若有剩餘的檢體，**將儲存直到至少完成臨床試驗報告為止，最長將保存20年。**

(2) 抗體/細胞免疫試驗

在試驗期間，會將您的檢體送往聯亞生技開發股份有限公司(試驗委託者)分析實驗

室。完成試驗後，若有剩餘檢體，將儲存直到至少完成臨床試驗報告為止，最長將保存20年。

(3) 中和試驗(neutralization test, NT)

在試驗期間，會將您的檢體送往聯亞生技開發股份有限公司(試驗委託者)委託的中央實驗室中央研究院進行處置、處理與進一步分析。此機構地址為台北市南港區研究院路二段128號。完成試驗後，若有剩餘檢體，將儲存直到至少完成臨床試驗報告為止，最長將保存20年。

(4) 遺傳學檢體

在試驗期間，若發生嚴重不良反應或特定不良反應，您的檢體將用於HLA分型檢驗，會將您的檢體送往聯亞生技開發股份有限公司(試驗委託者)委託的中央實驗室有勁基因股份有限公司分析，此機構地址為新北市樹林區復興路376-5號，中央實驗室不會將分析結果提供給試驗中心，若有剩餘的檢體，將會儲存直到檢驗結果複驗完畢即銷毀，不會長期儲存。

(5) 探索性試驗檢體

在試驗期間，會將您的檢體送往聯亞生技開發股份有限公司(試驗委託者)委託的實驗室(表一)進行處理或進一步分析。完成試驗後，若有剩餘檢體，將儲存直到至少完成臨床試驗報告為止，最長將保存20年。

表一、實驗室名稱與機構地址

**實驗室名稱**	**機構地址**
**聯亞生技開發(股)公司**	**新竹縣竹北市生醫路二段 6-1 號 5 樓**
**Viroclinics**	**Rotterdam Science Tower, Marconistraat 16, 3029 AK Rotterdam, The Netherlands(荷蘭)**
**DASA**	**Jonas Cruz de Araujo, Diagnostics da America S/A, Surubiju Avenue, 1890, Barueri, SP, Brazil(巴西), 06455-040**
**PHE Porton Down**	**Salisbury Wiltshire SP4 0JG, England(英國)**
**UTMB**	**University of Texas Medical Branch 301 University Boulevard Keiller Building, Room 2.150 Galveston, Texas, USA(美國)**
**Virology**	**University of São Paulo, Brazil Rua Dr EnnEn de Carvalho Aguiar 470, CEP 05403-000 (巴西)**
**VRDL**	**California Department of Public Health, 850 Marina Bay Parkway, Richmond, CA 94804, USA(美國)**

**NEXELIS**	**525 Boul. Cartier Ouest Laval, Qulbec, Canada, H7V 3S8(加拿大)**
**Vaccinology and Immunology Infection, Immunity & Inflammation Dept UCL GOS Institute of Childe Health**	**UCL Great Ormond Street Institute of Child Health 30 Guilford Street London WC1N 1EH, England(英國)**
**VisMederi**	**VisMederi Srl, Strada del Petriccio e Belriguardo, 35, 53100 Siena, Italy(義大利)**

(五)可能產生之副作用、發生率及處理方法：

1. 與試驗藥物相關的風險（本試驗疫苗的副作用）：

<u>冠狀病毒疫苗的開發</u>

過去針對與SARS-CoV-2病毒相同屬於人類冠狀病毒的SARS-CoV(嚴重急性呼吸綜合症冠狀病毒(SARS冠狀病毒))的疫苗研究發現，接種過SARS-CoV疫苗的小鼠在暴露到SARS-CoV後會發生過度免疫反應而產生病變，因此不得不停止這種疫苗的開發。所以，成功的人類冠狀病毒疫苗不只要產生可以抑制病毒的免疫反應，更要避免過度免疫產生的副作用。

<u>疫苗相關的風險: 第一期臨床試驗</u>

接種疫苗可能會出現注射部位的不良反應(例如疼痛、硬化腫脹、皮疹發紅、過敏反應、蜂窩性組織炎)，或全身性不良反應(例如發燒、腹瀉、疲倦、噁心/嘔吐、厭食、咽喉痛、頭痛、咳嗽、關節痛、非注射部位疼痛、非注射部位搔癢、皮膚和黏膜異常、急性過敏反應、昏厥、急性支氣管痙攣、呼吸困難)。

第一期臨床試驗已經有60位受試者接種兩劑疫苗(含10微克、30微克、100微克融合蛋白)，安全實驗室數值並沒有顯示有任何的臨床顯著不正常數值，也沒有發生任何第三級以上與疫苗相關的預期性不良事件。大部分的預期性不良事件都是輕微的，大約於2天之內症狀都會緩解。也沒有任何的嚴重不良事件或特殊不良事件被通報。

<u>疾病增強(disease enhancement) 的風險</u>

SARS-CoV-2候選疫苗也可能會有引發疾病增強(disease enhancement) 的風險，包括抗體依賴性增強(antibody-dependent enhancement)或疫苗相關聯的增強的呼吸道疾病(vaccine-associated enhanced respiratory disease)。在先前研發SARS疫苗時，在數個SARS-CoV動物攻毒試驗(包括鼠類、雪貂、猴類)當中，有發現疾病增強的現象。疾病增強反應的免疫病理現象包括TH2偏向及嗜酸性白血球的肺部浸潤。但是目前已發表的新型冠狀病毒肺炎

疫苗研究，仍尚未發現類似的疾病增強現象。

本試驗疫苗於數個藥理試驗呈現不一致的TH1/TH2 (輔助型T細胞1/輔助型T細胞2)免疫反應偏向，試驗結果並未一致偏向TH2，而由小鼠之SARS-CoV-2動物攻毒試驗結果顯示，本試驗疫苗誘發疾病增強之風險不高。依據文獻指出，組成複雜或容易引起非中和抗體之抗原，如不活化病毒或整片段之蛋白(包含S蛋白與N蛋白)，與易引起偏向Th2免疫反應之佐劑成分，如鋁製佐劑，皆較有可能引起疾病增強。本試驗疫苗的主要抗原為S蛋白上之RBD 區域，已有多篇文獻指出，針對S-RBD設計之SARS與MERS疫苗從未於試驗動物模型上引發疾病增強現象。本試驗疫苗雖使用易引起偏向Th2免疫反應之佐劑，但由動物實驗證實，也同時引起偏向Th1之反應，因此發生疾病增強應屬低風險。且已於多種動物模型中證實，能誘發高效價之中和抗體，於細胞培養中亦能有效抑制新冠病毒感染。

建議您在有效疫苗上市前或本試驗疫苗的產品資訊有進一步更新前，盡量避免暴露於可能感染病毒的環境。研究團隊將會在試驗中執行相關安全性監測。若有任何關於本試驗疫苗與疾病增強風險相關之任何最新資訊，將即時更新並提供給您。

疫苗佐劑相關的風險

本試驗疫苗所使用的佐劑含Adju-Phos®，是屬於一種磷酸鋁類的佐劑。磷酸鋁類佐劑已經使用超過半個世紀，具有相當的安全性。由於此類佐劑可誘導免疫反應，因此可能會造成局部發炎反應，例如在注射部位產生輕微而短暫的疼痛、發紅以及腫脹。

2. 與試驗/研究過程相關的風險：

抽血

本試驗需要抽血檢驗。抽血可能引起一些不適和瘀血。整個試驗期間13個月，共需抽血**116 毫升**。若您願意抽血檢驗T細胞檢測，則會再額外抽血共112毫升。若您罹患新型冠狀病毒感染，**可能將於每次額外訪視抽血30毫升。**

在接種疫苗過程中，可能會出現一些尚未在已完成試驗中發現的副作用。一般而言，接種某一新疫苗總是會有一定的風險，但是計畫主持人會採取一切措施預防風險的發生。**計畫主持人鼓勵您報告您遇到的任何不適。**

(六)其他替代療法及說明：

您不是非參加不可，若不參加研究，由於目前尚未有疫苗可用來預防新型冠狀病毒感染，因此預防措施與其他呼吸道感染相同，包括：勤洗手、減少觸摸眼口鼻、注意咳嗽禮節、妥善處理口鼻分泌物等，避免出入公共場所，並不要接觸野生動物。

如果您對於本試驗疫苗有任何的疑問，您可以提出來向您的試驗醫師討論。

（七）試驗預期效益：

依據臨床前試驗結果，預期本試驗疫苗對您可能可以產生抗體，預防新型冠狀病毒感染，但因每個人體質不同也有可能不會產生療效，故參加本試驗可能不會有直接的好處。

但是您參加本試驗，可協助我們獲得更多資訊，以瞭解UB-612疫苗的安全性與免疫力。

（八）試驗進行中受試者之禁忌、限制與應配合之事項：

禁止使用的藥物

以下藥物請勿在試驗期間使用：

- 直到試驗第197天禁止使用免疫抑制劑、或細胞毒性治療
- 到試驗第197天禁止使用免疫球蛋白和/或任何血液製劑
- 整個試驗期間禁止使用試驗產品(包括藥物或疫苗)
- 到試驗第197天禁止使用全身性皮質類固醇(相當於一天使用≥ 20 mg強的松(prednisone))
- 接種試驗疫苗後14天禁止接種任何季流感疫苗或新型流感疫苗，或後28天禁止接種其他非試驗疫苗。整個試驗期間禁止使用任何已上市的新型冠狀病毒疫苗產品。

允許使用的藥物

若您的藥物或治療必須常規使用，經試驗醫師判斷不會影響本試驗疫苗的免疫原性、臨床療效與安全性，則可以正常使用。您有任何關於在試驗期間可允許使用何種藥物或治療的問題，請詢問您的試驗醫師。

懷孕或母乳哺乳的風險

目前未知本試驗疫苗對於未出生胎兒的影響，因此：

- 您為具生育能力的女性受試者 (除非手術絕育或停經)，或您為男性受試者應於接種疫苗至最後一次疫苗後 3 個月同意進行有效的避孕方式，同意進行有效的避孕方式(例如子宮內節育器、荷爾蒙療法或避孕套)。
- 若您為具生育能力的女性，將請您進行懷孕檢測，結果必須為陰性，方可參與試驗。
- 若您為懷孕的女性，將被告知不可參與本試驗。

- 若您在試驗期間懷孕，請盡速通知試驗人員，並且停止施打本疫苗。
- 基於安全性考量，若您為女性受試者而在試驗期間懷孕，或您為男性受試者而您的性伴侶在試驗期間懷孕(將請您的懷孕性伴侶需簽署另外一份同意書)，您與您的胎兒將會被追蹤監測至分娩，除非另有醫學指示。

您應向您的配偶或性伴侶告知您有參與此試驗與相關風險:

簽名：__________日期：__________________

（九)機密性：

中國醫藥大學附設醫院將依法把任何可辨識您的身分之紀錄與您的個人隱私資料視為機密來處理，不會公開。研究人員將以一個研究代碼代表您的身分，此代碼不會顯示您的姓名、國民身分證統一編號、住址等可識別資料。如果發表試驗/研究結果，您的身分仍將保密。您亦瞭解若簽署同意書即同意您的原始醫療紀錄可直接受監測者、稽核者、研究倫理委員會及主管機關檢閱，以確保臨床試驗/研究過程與數據符合相關法律及法規要求，上述人員並承諾絕不違反您的身分之機密性。除了上述機構依法有權檢視外，我們會小心維護您的隱私。由於試驗藥物可能同時申請美國臨床試驗，依美國藥品管理規定，試驗結果將公佈於公開的臨床試驗資訊網站：Clinicaltrials.gov (美國)，但您的個人資料仍將保密，該網站只會有試驗之結果摘要，您可以在任何時候搜尋該網站。
在試驗/研究期間，依據計畫類型與您所授權的內容，我們將會蒐集與您有關的病歷資料、醫療紀錄、量表、問卷等資料與資訊，並以一個編號來代替您的名字及相關個人資料。前述資料若為紙本型式，將會與本同意書分開存放於研究機構之上鎖櫃中；若為電子方式儲存或建檔以供統計與分析之用，將會存放於設有密碼與適當防毒軟體之專屬電腦內。這些研究資料與資訊將會保存至藥品於我國上市後至少兩年，若試驗疫苗終止研發則保存至試驗正式停止後至少二年，至多將保存至疫苗上市後或試驗正式停止後二年。
上述資料與資訊若傳輸至國外分析與統計，您仍會獲得與本國法規相符之保障，計畫主持人與相關團隊將盡力確保您的個人資料獲得妥善保護。

（十)損害補償與保險：

1. 如依本研究所訂臨床試驗計畫，因發生不良反應造成損害，由聯亞生技開發股份有限公司負補償責任。但本受試者同意書上所記載之可預期不良反應，不予

補償。

2. 如依本研究所訂臨床試驗計畫，因而發生不良反應或損害，贊助廠商將依法負責損害賠償責任。本醫院願意提供專業醫療照顧及醫療諮詢。您不必負擔治療不良反應或損害之必要醫療費用。
3. 除前二項補償及醫療照顧外，本研究不提供其他形式之補償。若您不願意接受這樣的風險，請勿參加試驗。
4. 您不會因為簽署本同意書，而喪失在法律上的任何權利。
5. 本研究有投保責任保險。

（十一）受試者權利：

1. 試驗過程中，與您的健康或是疾病有關，可能影響您繼續接受臨床試驗意願的任何重大發現，都將即時提供給您。
2. 如果您在試驗過程中對試驗工作性質產生疑問，對身為患者之權利有意見或懷疑因參與研究而受害時，可與本院之研究倫理委員會聯絡請求諮詢，其電話號碼為：04-22052121轉1925、1926。
3. 為進行試驗工作，您必須接受黃高彬醫師的照顧。如果您現在或於試驗期間有任何問題或狀況，請不必客氣，可與在中國醫藥大學附設醫院兒童感染科的黃高彬醫師聯絡（24小時聯繫電話：0975-681-950）。
4. 參加試驗研究計畫之補助：本計畫將在每次訪視提供交通費2000元及營養費1000元給您，整個試驗預計給予您18000元。若您願意參與T細胞檢測研究，將在該次訪視另外提供營養費500元給您。
5. 本同意書一式2份，醫師已將同意書副本交給您，並已完整說明本研究之性質與目的。醫師已回答您有關藥品與研究的問題。

（十二）試驗之退出與中止：

您可自由決定是否參加本試驗；試驗過程中也可隨時撤銷同意，退出試驗，不需任何理由，且不會引起任何不愉快或影響其日後醫師對您的醫療照顧。

計畫主持人或贊助廠商亦可能於必要時中止該試驗之進行。

（十三）簽名：

1. 計畫主持人、或協同主持人已詳細解釋有關本研究計畫中上述研究方法的性質與目的，及可能產生的危險與利益。

   計畫主持人/協同主持人簽名：____________日期：________年____月____日
2. 受試者已詳細瞭解上述研究方法及其所可能產生的危險與利益，有關本試驗計畫的疑問，業經試驗主持人詳細予以解釋。本人同意接受為臨床試驗計畫的自

願受試者。

受試者簽名：____________________________日期：________年____月____日

法定代理人簽名：________________________日期：________年____月____日

* 受試者為無行為能力(未滿七歲之未成年人者或禁治產人)，由法定代理人為之；禁治產人，由監護人擔任其法定代理人。

* 受試者為限制行為人者(滿七歲以上之未成年人)，應得法定代理人之同意。

有同意權人簽名：________________________日期：________年____月____日

* 受試者雖非無行為能力或限制行為能力者，但因意識混亂或有精神與智能障礙，而無法進行有效溝通和判斷時，由有同意權之人為之。前項有同意權人為配偶及直系親屬。

3. 見證人

見證人簽名：_____________________________日期：________年____月____日

身分證字號：　　　　　　　　　　　　聯絡電話：

通訊地址：

* 受試者、法定代理人或有同意權之人皆無法閱讀時，應由見證人在場參與所有有關受試者同意之討論。並確定受試者、法定代理人或有同意權之人之同意完全出於其自由意願後，應於受試者同意書簽名並載明日期。試驗相關人員不得為見證人。

受試者同意書
（成年免疫組）

流程表:

批次分析與免疫分析組

訪視	1[m]	2 [m]		3		4		5/提早退出試驗	6[t]		7		長期性追蹤
**檢測項目**	**篩選**	**第一次接種**		**第二次接種**		**追蹤 [g]**		**個別解盲**	**第三次接種 [h]**		**追蹤 [h]**		**第 12 個月追蹤 [i]**
**天數**	**-28~-1**	**1**	**8, 15, 22**	**29 ±3 天**	**36, 43**	**57 ±3 天**	**64, 71, 78,85**	**197 ±15 天**	**197~242**	**第六次訪視後 7 天**	**第六次訪視後 14 天 ±3 天**	**253, 309**	**365 ±45 天**
獲得受試者同意書	X								**X[h]**				
納入/排除條件	X	X											
隨機分派		X											
**接種評估**				X					**X [h]**				
基本資料	X												
醫療病史	X	X											
身體檢查 [a]	X	X		X		X		X	**X [h]**		**X [h]**		
生命徵象	X	X		X		X		X	**X [h]**		**X [h]**		**X**
心電圖	X												
**實驗室檢測 (安全性)**													
血液常規檢測 [j]	X					X			**X [h]**		**X [h]**		
血液生化學檢測 [j]	X					X			**X [h]**		**X [h]**		
免疫檢測 [j]	X					X			**X [h]**		**X [h]**		
懷孕檢測 [b]		X		X					**X [h]**				

訪視	1[m]	2 [m]		3		4		5/提早退出試驗	6[t]		7		長期性追蹤
**檢測項目**	**篩選**	**第一次接種**		**第二次接種**		**追蹤 [g]**		**個別解盲**	**第三次接種 [h]**		**追蹤 [h]**		**第 12 個月追蹤 [i]**
**天數**	**-28~-1**	**1**	**8, 15, 22**	**29 ±3 天**	**36, 43**	**57 ±3 天**	**64, 71, 78,85**	**197 ±15 天**	**197~242**	**第六次訪視後 7 天**	**第六次訪視後 14 天 ±3 天**	**253, 309**	**365 ±45 天**
尿液常規檢測 [b]	X	X [q]		X [q]		X [q]		X [q]	**X [h, q]**		**X [h, q]**		
**實驗室檢測(免疫原性)**													
免疫原性 [c]		X		X [n]		X		X					X
T 細胞反應 (可選擇 [o])		X				X							
**實驗室檢測(探索性試驗)**													
**T 細胞功能性檢測(可選擇 [u])**									**X [h]**		**X[h]**		
**備血**	X					X							
**免疫原性 [c]**									**X [h]**		**X[h]**		
疫苗接種		X		X					**X [h]**				
指導使用電子日誌卡		X		X					**X [h]**				
電話安全性追蹤 [d]			X		X		X			**X[s ,h]**		X	
不良事件/特殊不良事件 [p]/醫		X [k]	X [k]	X [k]	X [k]	X [k]	X [k]	X [l]	**X [l, h]**	**X [l, h]**	**X [l, h]**	X [l]	X [l]

中國醫藥大學暨附設醫院
受試者同意書
（成年免疫組）

訪視	1[m]	2 [m]		3		4		5/提早退出試驗	6[t]		7		長期性追蹤
檢測項目	篩選	第一次接種		第二次接種		追蹤 [g]		個別解盲	第三次接種 [h]		追蹤 [h]		第 12 個月追蹤 [i]
天數	-28~-1	1	8, 15, 22	29 ±3 天	36, 43	57 ±3 天	64, 71, 78,85	197 ±15 天	197~242	第六次訪視後 7 天	第六次訪視後 14 天 ±3 天	253, 309	365 ±45 天
療需求不良事件/嚴重不良事件													
新型冠種病毒感染監測		X	X	X	X	X [e]	X [e]	X [e]	**X** [e, h]	**X** [e, h]	**X** [e, h]	X [e]	X [e]
併用藥物		X		X		X		X [f]	**X** [f, h]		**X** [f, h]		

a: 身高與體重僅在第一次訪視測量。
b:於第 1, 29 天使用尿液懷孕檢測。若尿液檢測為陽性，應以血清懷孕檢測再次確認。以血清懷孕檢測替代尿液檢測將不視為試驗偏差。蛋白尿將透過尿液常規檢測確認，做為基礎值。
c: 針對 Anti-S1-RBD 免疫球蛋白 G 濃度和新型冠狀病毒中和抗體效價，以及抑制 S1-RBD: ACE2 的抗體效價。
d: 所有受試者將進行電話安全追蹤以監測非預期性不良事件，包括特殊不良事件，和監測新型冠狀病毒感染的症狀。
e: 受試者將在每周(為每 7 天)由手機接獲一條提示以規律監測新型冠狀病毒感染症狀或病徵。記錄疑似新冠病毒感染所使用的藥物至新冠病毒感染頁面。
f: 只記錄醫療需求不良事件和嚴重不良事件之併用藥物。
g: 第二次接種疫苗後第 28 天
h: 僅針對同意並進行施打第三劑試驗疫苗的疫苗組受試者
i: 第二次接種疫苗後第十二個月(第 365 天)
j: 安全性實驗室數值包括全血液計數(血紅素、血比容、紅血球計數)、白血球計數、血小板計數、肌酸酐、丙胺酸轉胺酶、天門冬胺酸轉胺酶、總膽紅素、直接膽紅素、高靈敏度 C 反應性蛋白、抗核抗體
k: 主動收集時期
l: 被動監測時期
m: 第一次訪視與第二次訪視可為同一次訪視。
n: 僅測量 Anti-S1-RBD IgG 濃度
o: 將在選定的試驗地點納入至少 100 位>18-<65 歲的受試者。
p：包括接種最後一劑疫苗後 12 個月，可能的免疫媒介醫療狀況(PIMMC)或任何定義為可能的特殊不良事件。新冠病毒感染的併發症也將視為疾病增強事件，將被記錄與通報為特殊不良事件。
q: 若受試者有≥第三級以上的高血壓，受試者將確認是否有蛋白尿存在或惡化。
r: 備血將被冷凍存放做為 UBI 新型冠狀病毒酵素結合免疫吸附分析法，新型冠狀病毒確認酵素結合免疫吸附分析法，以及未來免疫學研究之用。
s: 若符合資格並接種第三劑疫苗的受試者將進行兩週的日誌卡追蹤紀錄，並將於接種後第 7 天進行電話安全性追蹤。

t: 第五次訪視和第六次訪視可為同一天。
u: 在選定之試驗地點中，將邀請大約 30 位年齡 >18-<65 歲的受試者和大約 30 位年齡 ≥65 歲的受試者。在適用的情況下，優先邀請曾進行過第 57 天 T 細胞功能評估的受試者。

中國醫藥大學暨附設醫院
受試者同意書
（成年安全確認組）

您被邀請參與此研究。此同意書主要是提供您本研究之相關資訊，以便您決定是否參加本研究。計畫主持人或其指定之研究人員會為您說明研究內容並回答您的疑問。您可以提出任何和此研究有關的問題，在您的問題尚未獲得滿意的答覆之前，請不要簽署此同意書。如果您願意參與本研究，此文件將視為您的同意紀錄。即使在您同意後，您可以隨時退出本研究不需任何理由。

計畫名稱<br>中文：一個評估 UB-612 疫苗對於新型冠狀病毒於青少年、成年和老年健康受試者的免疫原性、安全性與耐受性的第二期、安慰劑控制、隨機分派、觀察者盲性臨床試驗<br>英文：A Phase II, Placebo-controlled, Randomized, Observer-blind Study to Evaluate the Immunogenicity, Safety and Tolerability of UB-612 Vaccine against COVID-19 in Adolescent, Younger and Elderly Adult Volunteers	
執行單位：中國醫藥大學附設醫院感染科、家庭醫學科	委託單位/藥廠：聯亞生技開發股份有限公司<br>研究經費來源：聯亞生技開發股份有限公司<br>受託研究機構：晉加股份有限公司
計畫主持人：黃高彬	職稱：主治醫師
協同主持人：林文元	職稱：主治醫師
協同主持人：林伯昌	職稱：主治醫師
緊急聯絡人：黃高彬	電話：0975-681-950
受試者姓名：<br>性別：<br>身分證字號：<br>通訊地址：	病歷號碼：<br>出生日期：<br>聯絡電話：
法定代理人或有同意權人之姓名：<br>性別：<br>身分證字號：<br>通訊地址：	與受試者關係：<br>出生日期：<br>聯絡電話：
(一)試驗簡介：<br>1. 本品/技術資料：	

新型冠狀病毒(SARS-CoV-2)於2019年12月起造成中國湖北省武漢市發現多起病毒性肺炎群聚，隨後於2020年1月底台灣出現第一起境外移入確診個案。此疾病在全球擴散，世界衛生組織宣布將此疫情為「國際關注公共衛生緊急事件」。截至2020年底，全球僅有數間疫苗公司，如美國輝瑞藥廠等，取得緊急使用授權上市。

UB-612疫苗為聯亞生技開發股份有限公司所開發新型冠狀病毒預防性疫苗，疫苗含病毒棘狀融合蛋白和胜肽片段，可產生高親和力抗體與新型冠狀病毒結合，並誘發細胞免疫反應，進而達到預防新型冠狀病毒的感染。

UB-612第一期延伸性試驗顯示，接種第三劑UB-612疫苗可以誘發極高的中和抗體，在目前變種病毒的威脅之下，施打第三劑加強免疫反應，已是許多國家的選擇。

2. 本品上市狀況：

本品仍應用於人體試驗，尚未在我國上市。

3. 本試驗使用的UB-612疫苗對新型冠狀病毒的預防效果仍未確認。

（二）試驗目的：

主要試驗目的

- 評估UB-612疫苗誘發的新型冠狀病毒中和抗體效價。
- 評估接種UB-612疫苗後的安全性和耐受性。

次要試驗目的

- 評估在試驗期間對於新型冠狀病毒的免疫反應。
- 評估三批獨立批次疫苗的批次免疫一致性。

探索性試驗目的

- 評估UB-612疫苗誘發的T細胞功能。
- 評估UB-612疫苗在年輕受試者的安全性和免疫原性。
- 評估UB-612疫苗的療效。
- 描述UB-612疫苗於確診和/或嚴重感染新型冠狀病毒案例之血液學反應。
- **評估針對SARS-CoV-2抗原的抗體反應。**

(三)試驗之主要納入與排除條件：

中國醫藥大學暨附設醫院執行本研究計畫的醫師或相關研究人員將會與您討論有關參加本研究的必要條件。請您配合必須誠實告知我們您過去的健康情形，若您有不符合參加本研究的情況，將不能參加本研究計畫。

1. 參加本研究計畫的主要條件:

☐(1) 您為納入試驗時20~85歲之間健康男性或未懷孕的女性受試者。

☐(2) 您為具生育能力的女性與男性應於首次接種疫苗至最後一次疫苗後3個月同意進行有效的避孕方式。可接受的有效避孕方式包括:

☐a. 男性或女性以手術方法絕育、植入式避孕、或子宮避孕器。

☐b.注射避孕、避孕藥、避孕貼片、避孕環加上一種屏障避孕法*。

☐c.合併使用兩種屏障避孕法*。

*有效的屏障避孕法為避孕隔膜、男性或女性保險套、避孕海綿或殺精劑(含可殺精化學物質的藥膏或凝膠)。

☐(3) 您能理解受試者同意書內容的說明與可能的風險，提供簽名的受試者同意書。

☐(4) 您能夠理解與遵從本試驗程序與能夠參與每次訪視。

☐(5) 您的耳溫≤ 38.0°C。

☐(6) 您依據醫療病史、身體檢查和試驗主持人的臨床判斷為健康受試者**可符合納入試驗資格。經試驗主持人判斷，即便您的病史穩定或且控制良好，但伴隨病情惡化而有提高嚴重新型冠狀病毒感染的風險。

**健康受試者有先前存在的穩定疾病者可以納入試驗，定義為該疾病在納入試驗前12週內沒有惡化至需要治療或住院的顯著變化和在納入試驗6個月內沒有惡化至需要治療或住院的顯著變化。

2. 若您有下列任一情況，您將無法參加本研究計畫:

☐(1) 您有接種疫苗後需要醫療介入的過敏性休克、蕁麻疹或其他顯著不良反應的病史。

☐(2) 您在篩選時或接種每劑疫苗前已懷孕女性或懷孕檢測為陽性的女性。

☐(3) 您為正在哺乳的女性，或計畫從接種第一劑疫苗至最後一劑疫苗後60天哺乳的女性。

☐(4) 您在接種第一劑疫苗前3天內，經試驗主持人判斷，患有任何急性疾病。

☐(5) 您在接種第一劑疫苗前1個月內有重大手術。

☐(6) 您是已知為人類免疫缺乏病毒抗體陽性。

☐(7) 您是已知為活動性B型肝炎或C型肝炎。活動性肝炎定義為肝臟轉胺酶(天門冬胺酸轉胺酶和/或丙胺酸轉胺酶)大於3倍正常值上限和/或總膽紅素大於3倍正常值上限。

☐(8) 您是已知曾暴露於新型冠狀病毒，或曾接受預防新型冠狀病毒、中東呼吸症候群冠狀病毒、嚴重急性呼吸道症候群的試驗或已上市產品。

☐(9) 您有格林-巴利症候群的病史。

☐(10) 您在簽署受試者同意書前12周內參與其他的臨床試驗。

☐(11) 您為免疫缺乏/失調疾病，無論是否由基因缺陷、免疫缺乏症或免疫抑制療法所造成。

☐(12) 您計畫或正在進行抗癌症治療。

☐(13) 您患有血小板異常或其他凝血異常可能造成注射之禁忌症。

☐(14) 您在接種第一劑疫苗前6個月長期接受(≥14天連續使用)免疫抑制劑、皮質類固醇(相當於一天使用≥ 20 mg強的松(prednisone))或細胞毒性治療。

☐(15) 您在接種第一劑疫苗前4個月接受免疫球蛋白和/或任何血液製劑的治療。

☐(16) 您在接種試驗疫苗前14天接種任何季流感疫苗或新型流感疫苗，或前28天接種其他疫苗。

☐(17) 您預期在接種試驗疫苗後14天接種任何季流感疫苗或新型流感疫苗，或後28天接種其他疫苗。

☐(18) 您使用短期(<14天使用)全身性類固醇。應於中斷使用全身性類固醇至少28天後才可使用試驗疫苗。吸入/噴霧性、關節注射、囊內或局部(皮膚或眼用)類固醇可允許使用。

☐(19) 您在篩選期前3個月失血或捐血超過500毫升，或預計在試驗期間內捐血或輸血。

☐(20) 經試驗主持人判斷，您有任何醫療疾病或狀況，可能會影響試驗結果或參與試驗可能會對受試者引發額外風險。

☐(21) 您是直接參與本試驗執行的試驗主持人所屬機構**的試驗團隊、試驗委託者或受託**

研究機構(CRO)的員工。

(四)試驗方法及相關檢驗：

這是一個第二期、觀察者盲性、多中心、隨機分派、安慰劑控制試驗，以評估青少年，成年和老年受試者使用兩劑UB-612疫苗的免疫原性，耐受度和安全性。有一部份的受試者使用UB-612疫苗，而另外一部份的受試者則使用「安慰劑」。所謂「安慰劑」是不含有效成份的疫苗。至於誰使用試驗用藥或誰使用「安慰劑」，則像丟銅板或擲骰子一樣由機率決定，不管是您或是研究醫師都不知道您使用了那一種藥，只有分發跟施打疫苗的試驗人員才知道您使用哪一種疫苗，這叫做觀察者盲性。

總計約有3850位合格成年受試者組成核心組用於申請緊急使用授權，另外大約有385位青少年受試者組成補充組申請額外適應症。所有受試者將以6:1的比例，隨機分派至兩劑100微克劑量組別和安慰劑組，包括462位大於18歲至小於65歲可評估的受試者進入批次分析組。對於免疫分析，至少包括350位可評估的成年受試者(年齡大於18歲至小於65歲)和154位可評估的老年受試者(年齡≥65歲)進行描述性分析。免疫原性的受試者將會先納入試驗。所有的受試者將會納入安全性分析，其中至少770位隨機分派的受試者為≥65歲的分層。青少年組將在核心組招募完畢後，再開始納入試驗。約有385位青少年受試者將以6:1的比例隨機分派，其中包括154位可評估的青少年受試者將收集免疫原性數據，並和成年及老年受試者數據進行比較。

您若參與這個試驗，則為進行安全性確認的安全確認組。

試驗總共有8個訪視。若您參與本試驗，則至少包括第一次訪視(篩選訪視)、第二次訪視（第1天，基礎值，隨機分派，第一次接種疫苗)、第三次訪視(第29天，第二次接種疫苗)、第四次訪視(第57天)、第五次訪視(第197天)。

在第五次訪視時，預計將進行個別解盲。解盲後，得知您為施打疫苗的受試者，且您有意願且符合資格接種第三劑疫苗，將請您簽署另外一份受試者同意書，以進行後續程序；包含第六次訪視(第197~242天，第三次接種疫苗)，第七次訪視(接種疫苗後第14天)，及第八次訪視(第365天)。

若個別解盲後，得知您為施打疫苗的受試者，您不願意進行第三劑疫苗接種，將進行第八次訪視(第365天)的安全性追蹤。

若個別解盲後，得知您為施打安慰劑組的受試者，將結束您的試驗。

整個試驗期間，將預期您將參與試驗最長達13個月。

中國醫藥大學暨附設醫院
受試者同意書
（成年安全確認組）

注意事項

1. 如果您同意參加本試驗，研究人員會請您簽署本份受試者同意書，並確認您符合參加本試驗的條件。
2. 從您參與試驗的當天開始，每次訪視都將有合格的試驗人員執行試驗流程與聯繫。
3. 若您有任何符合新型冠狀病毒感染的定義(您曾於過去 7 天內有出國，或是接觸疑似或確認武漢肺炎之病人，而有下列症狀: 發燒、開始咳嗽或惡化、開始呼吸急促或惡化、寒顫、開始肌肉疼痛或惡化、喉嚨痛、腹瀉、嘔吐、開始味覺/嗅覺異常)，請**依照中央疫情指揮中心規定進行自主健康管理或至指定院所進行篩檢。**
4. 若您在試驗期間感染新型冠狀病毒，將依法通報主管機關。

   您是否同意? □是 □否

   簽名：__________日期：__________
5. 於試驗期間，您不論任何理由提前退出試驗，試驗研究人員都將安排您完成最後一次的訪視之所有試驗項目。您有權利拒絕此項安排，您的決定不會引起任何影響日後醫師對您的醫療照護。

試驗步驟

第一次訪視(第-28~-1 天) -篩選訪視

在試驗醫師或試驗研究人員為您提供足夠的試驗資訊，並確保您有充分的時間考慮以及詢問任何問題後，您願意讓您參與本試驗，並由您簽署本受試者同意書。在確認您已完成受試者同意書簽署並且您也保有一份副本後，試驗醫師或試驗研究人員將會進行以下試驗程序：

(1) 記錄您簽署受試者同意書的日期
(2) 為您指定一組受試者篩選編號
(3) 確認您是否符合本試驗的納入排除條件
(4) 收集您的個人基本資料（例如生日、年齡及性別）
(5) 記錄您的醫療/用藥病史
(6) 進行身體檢查，包括身高體重
(7) 確認生命徵象

(8) 進行心電圖檢測

(9) 收集尿液檢體進行尿液檢測

(10) 收集血液檢體(共 **15.5** 毫升)，進行下列檢測:

- 常規血液檢測
- 血液生化學檢測
- 免疫學檢測**(抗核抗體)**
- **備血(作為測量新型冠狀病毒血清抗體和相關研究之用)**

第二次訪視(第 1 天)-基礎值，確認符合試驗條件，第一次接種疫苗

(1) 再度確認您是否符合本試驗的納入排除條件

(2) 隨機分派，給予您一組隨機分派號碼

(3) 記錄您的醫療/用藥病史

(4) 進行身體檢查

(5) 確認生命徵象

(6) 進行尿液懷孕檢測（具有生育能力女性）

(7) 若您有產生第三級以上的高血壓，您將被收集尿液檢體以檢測是否有蛋白尿存在或惡化

(8) 進行第一次疫苗接種(注射部位為非慣用手，採用肌肉注射方式)。接種疫苗後，受試者應留在試驗地點至少 30 分鐘，監測生命徵象和急性過敏症狀。

(9) 將詳細地指導您如何填寫電子日誌卡(包括注射後 7 天內預期性不良事件，14 天內的皮膚過敏反應日誌卡)

(10) 收集併用藥物/治療

第一次電話安全性追蹤(第 8, 15, 22 天)

將與您電話聯繫，以追蹤未預期不良事件和新型冠狀病毒感染症狀。

第三次訪視(第 29± 3 天)-第二次接種疫苗

(1) 進行第二次接種評估(有可能會延遲接種時間)

(2) 進行身體檢查

(3) 確認生命徵象

(4) 進行尿液懷孕檢測（具生育能力的女性）

(5) 若您有產生第三級以上的高血壓，您將被收集尿液檢體以檢測是否有蛋白尿存在或惡化。

(6) 進行第二次疫苗接種。

(7) 接種疫苗後，受試者應留在試驗地點至少 30 分鐘，監測生命徵象和急性過敏症

狀。

(8) 將詳細地指導您如何填寫電子日誌卡(包括注射後 7 天內預期性不良事件，14 天內的皮膚過敏反應日誌卡)

(9) 收集併用藥物/治療

(10) 記錄上一次訪視至此次訪視之間的不良事件、嚴重不良事件型或新型冠狀病毒感染症狀

第二次電話安全性追蹤(第 36, 43 天)

將與您電話聯繫，以追蹤未預期不良事件和新型冠狀病毒感染症狀。

第四次訪視(第 57±3 天) –追蹤訪視

(1) 進行身體檢查

(2) 確認生命徵象

(3) 若您有產生第三級以上的高血壓，您將被收集尿液檢體以檢測是否有蛋白尿存在或惡化。

(4) 收集血液檢體(共 **15.5** 毫升) ，進行下列檢測:

- 常規血液檢測
- 血液生化學檢測
- 免疫學檢測**(抗核抗體)**
- **備血(作為測量新型冠狀病毒血清抗體和相關研究之用)**

(5) 收集併用藥物/治療

(6) 記錄上一次訪視至此次訪視之間的不良事件、嚴重不良事件或新型冠狀病毒感染症狀

(7) 在此次訪視後，您將每周接獲訊息提醒，以定期監測新型冠狀病毒感染症狀至第 197 天。

第三次電話安全性追蹤(第 64, 71, 78, 85 天)

將與您電話聯繫，以追蹤安全性及新型冠狀病毒感染症狀。

第五次訪視(第 197±15 天) –個別解盲

(1) 進行身體檢查

(2) 確認生命徵象

(3) 若您有產生第三級以上的高血壓，您將被收集尿液檢體以檢測是否有蛋白尿存在或惡化。

(4) 將替您個別解盲，告知您接種到疫苗或安慰劑。若您為安慰劑組，您已完成本試驗，將協助您退出試驗。但若您為疫苗組且符合資格，我們將提供另外一份受試

者同意書，邀請您參與後續的第三劑接種試驗。若您不同意接種第三劑，您仍需進行後續的安全性追蹤。

(5) 收集併用藥物/治療

(6) 記錄上一次訪視至此次訪視之間的不良事件、嚴重不良事件或新型冠狀病毒感染症狀

第四次電話安全性追蹤(第 253, 309 天)

第 197 天後將每兩個月與您電話聯繫，以追蹤安全性及新型冠狀病毒感染症狀。

最後一次訪視(第 365±45 天) –第 12 個月追蹤

(1) 確認生命徵象

(2) 記錄上一次訪視至此次訪視之間的不良事件，包括特殊不良事件、醫療不良事件、嚴重不良事件或新型冠狀病毒感染症狀

受試者之檢體(含其衍生物)之保存、使用與再利用：

1. 檢體及剩餘檢體之保存與使用

(1) 檢體(含其衍生物)之保存與使用

為研究所需，我們所蒐集您的檢體，將依本研究計畫使用，檢體將保存於**聯亞生技開發股份有限公司(試驗委託者)**，直至 20 年保存期限屆滿，我們將依法銷毀。為了保護您的個人隱私，我們將以一個試驗編號來代替您的名字及相關個人資料，以確認您的檢體及與相關資料受到完整保密。如果您對檢體的使用有疑慮，或您有任何想要銷毀檢體的需求，請立即與我們聯絡(聯絡人：黃高彬醫師電話：0975-681-950），我們即會將您的檢體銷毀。您也可以聯繫中國醫藥大學暨附設醫院研究倫理委員會(電話：04-22052121 轉 1925、1926)，以協助您解決檢體在研究使用上的任何爭議。

(2) 剩餘檢體(含其衍生物)之再利用

您的生物檢體將會以專屬號碼進行編碼並在**聯亞生技開發股份有限公司(試驗委託者)**的控管下儲存最長20年，以研究UB-612 疫苗反應者的生物標記，及改善治療方式。

所有新的研究計畫都要再經由中國醫藥大學暨附設醫院研究倫理委員會審議通過，倫理審查委員會若認定新的研究超出您同意的範圍，將要求我們重新得到您的同意。

是否同意剩餘檢體保留提供未來新型冠狀病毒感染研究之用，並授權中國醫藥大學

暨附設醫院研究倫理委員會審議是否需要再取得您的同意(擇一)

☐ 不同意保存我的剩餘檢體，試驗結束後請銷毀

☐ 同意以非去連結之方式保存我的剩餘檢體，逾越原同意使用範圍時，需再次得到我的同意才可使用我的檢體進行新的研究

2. 檢體及剩餘檢體之部分類型(檢體類型可依計畫書內容自行增減)

(1) 一般生化、血液檢驗/病毒檢測檢體

在試驗期間，會將您的檢體送往聯亞生技開發股份有限公司(試驗委託者)委託的中央實驗室中國醫藥大學暨附設醫院，此機構地址為台中市北區育德路2號，和大安聯合醫事檢驗所，此機構地址為台北市大安區復興南路二段151巷33號，中央實驗室會在分析後立即將分析結果提供給試驗中心，若有剩餘的檢體，**將儲存直到至少完成臨床試驗報告為止，最長將保存20年。**

若在研究期間安排您回診確認COVID-19感染，剩餘檢體保存與使用說明如下：

(2) 抗體/細胞免疫試驗

在試驗期間，會將您的檢體送往聯亞生技開發股份有限公司(試驗委託者)分析實驗室。完成試驗後，若有剩餘檢體，將儲存直到至少完成臨床試驗報告為止，最長將保存20年。

(3) 中和試驗(neutralization test, NT)

在試驗期間，會將您的檢體送往聯亞生技開發股份有限公司(試驗委託者)委託的中央實驗室中央研究院進行處置、處理與進一步分析。此機構地址為台北市南港區研究院路二段 128 號。完成試驗後，若有剩餘檢體，將儲存直到至少完成臨床試驗報告為止，最長將保存 20 年。

(4) 遺傳學檢體

在試驗期間，若發生嚴重不良反應或特定不良反應，您的檢體將用於 HLA 分型檢驗，會將您的檢體送往聯亞生技開發股份有限公司(試驗委託者)委託的中央實驗室有勁基因股份有限公司分析，此機構地址為新北市樹林區復興路 376-5 號，中央實驗室不會將分析結果提供給試驗中心，若有剩餘的檢體，將會儲存直到檢驗結果複驗完畢即銷毀，

不會長期儲存。

(5) 探索性試驗檢體

在試驗期間，會將您的檢體送往聯亞生技開發股份有限公司(試驗委託者)委託的實驗室(表一)進行處理或進一步分析。完成試驗後，若有剩餘檢體，將儲存直到至少完成臨床試驗報告為止，最長將保存 20 年。

表一、實驗室名稱與機構地址

實驗室名稱	機構地址
聯亞生技開發(股)公司	新竹縣竹北市生醫路二段 6-1 號 5 樓
Viroclinics	Rotterdam Science Tower, Marconistraat 16, 3029 AK Rotterdam, The Netherlands(荷蘭)
DASA	Jonas Cruz de Araujo, Diagnostics da America S/A, Surubiju Avenue, 1890, Barueri, SP, Brazil(巴西), 06455-040
PHE Porton Down	Salisbury Wiltshire SP4 0JG, England(英國)
UTMB	University of Texas Medical Branch 301 University Boulevard Keiller Building, Room 2.150 Galveston, Texas, USA(美國
Virology	University of São Paulo, Brazil Rua Dr EnnEn de Carvalho Aguiar 470, CEP 05403-000 (巴西)
VRDL	California Department of Public Health, 850 Marina Bay Parkway, Richmond, CA 94804, USA(美國)
NEXELIS	525 Boul. Cartier Ouest Laval, Qulbec, Canada, H7V 3S8(加拿大)
Vaccinology and Immunology Infection, Immunity & Inflammation Dept UCL GOS Institute of Childe Health	UCL Great Ormond Street Institute of Child Health 30 Guilford Street London WC1N 1EH, England(英國)
VisMederi	VisMederi Srl, Strada del Petriccio e Belriguardo, 35, 53100 Siena, Italy(義大利)

(五)可能產生之副作用、發生率及處理方法：

1. 與試驗藥物相關的風險（本試驗疫苗的副作用）：

<u>冠狀病毒疫苗的開發</u>

過去針對與SARS-CoV-2病毒相同屬於人類冠狀病毒的SARS-CoV(嚴重急性呼吸綜合症冠狀病毒(SARS冠狀病毒))的疫苗研究發現，接種過SARS-CoV疫苗的小鼠在暴露到SARS-CoV後會發生過度免疫反應而產生病變，因此不得不停止這種疫苗的開發。所以，

成功的人類冠狀病毒疫苗不只要產生可以抑制病毒的免疫反應，更要避免過度免疫產生的副作用。

疫苗相關的風險: 第一期臨床試驗

接種疫苗可能會出現注射部位的不良反應(例如疼痛、硬化腫脹、皮疹發紅、過敏反應、蜂窩性組織炎)，或全身性不良反應(例如發燒、腹瀉、疲倦、噁心/嘔吐、厭食、咽喉痛、頭痛、咳嗽、關節痛、非注射部位疼痛、非注射部位搔癢、皮膚和黏膜異常、急性過敏反應、昏厥、急性支氣管痙攣、呼吸困難)。

第一期臨床試驗已經有60位受試者接種兩劑疫苗(含10微克、30微克、100微克融合蛋白)，安全實驗室數值並沒有顯示有任何的臨床顯著不正常數值，也沒有發生任何第三級以上與疫苗相關的預期性不良事件。大部分的預期性不良事件都是輕微的，症狀在大約於2天之內都會緩解。試驗中也沒有任何的嚴重不良事件或特殊不良事件被通報。

疾病增強(disease enhancement) 的風險

SARS-CoV-2候選疫苗也可能會有引發疾病增強(disease enhancement) 的風險，包括抗體依賴性增強(antibody-dependent enhancement)或疫苗相關聯的增強的呼吸道疾病(vaccine-associated enhanced respiratory disease)。在先前研發SARS疫苗時，在數個SARS-CoV動物攻毒試驗(包括鼠類、雪貂、猴類)當中，有發現疾病增強的現象。疾病增強反應的免疫病理現象包括TH2偏向及嗜酸性白血球的肺部浸潤。但是目前已發表的新型冠狀病毒肺炎疫苗研究，仍尚未發現類似的疾病增強現象。

本試驗疫苗於數個藥理試驗呈現不一致的TH1/TH2 (輔助型T細胞1/輔助型T細胞2)免疫反應偏向，試驗結果並未一致偏向TH2，而由小鼠之SARS-CoV-2動物攻毒試驗結果顯示，本試驗疫苗誘發疾病增強之風險不高。依據文獻指出，組成複雜或容易引起非中和抗體之抗原，如不活化病毒或整片段之蛋白(包含S蛋白與N蛋白)，與易引起偏向Th2免疫反應之佐劑成分，如鋁製佐劑，皆較有可能引起疾病增強。本試驗疫苗的主要抗原為S蛋白上之RBD 區域，已有多篇文獻指出，針對S-RBD設計之SARS與MERS疫苗從未於試驗動物模型上引發疾病增強現象。本試驗疫苗雖使用易引起偏向Th2免疫反應之佐劑，但由動物實驗證實，也同時引起偏向Th1之反應，因此發生疾病增強應屬低風險。且已於多種動物模型中證實，能誘發高效價之中和抗體，於細胞培養中亦能有效抑制新冠病毒感染。

建議您在有效疫苗上市前或本試驗疫苗的產品資訊有進一步更新前，盡量避免暴露於可能感染病毒的環境。研究團隊將會在試驗中執行相關安全性監測。若有任何關於本試驗疫苗與疾病增強風險相關之任何最新資訊，將即時更新並提供給您。

疫苗佐劑相關的風險

本試驗疫苗所使用的佐劑含Adju-Phos®，是屬於一種磷酸鋁類的佐劑。磷酸鋁類佐劑已

經使用超過半個世紀，具有相當的安全性。由於此類佐劑可誘導免疫反應，因此可能會造成局部發炎反應，例如在注射部位產生輕微而短暫的疼痛、發紅以及腫脹。

2. 與試驗/研究過程相關的風險：

抽血

本試驗需要抽血檢驗。抽血可能引起一些不適和瘀血。整個試驗期間13個月，共需抽血**31毫升**。若您罹患新型冠狀病毒感染，可能將於每次額外訪視抽血30毫升，預計共額外抽血60毫升。

在接種疫苗過程中，可能會出現一些尚未在已完成試驗中發現的副作用。一般而言，接種某一新疫苗總是會有一定的風險，但是計畫主持人會採取一切措施預防風險的發生。**計畫主持人鼓勵您報告您遇到的任何不適。**

（六）其他替代療法及說明：

您不是非參加不可，若不參加研究，由於目前尚未有疫苗可用來預防新型冠狀病毒感染，因此預防措施與其他呼吸道感染相同，包括：勤洗手、減少觸摸眼口鼻、注意咳嗽禮節、妥善處理口鼻分泌物等，避免出入公共場所，並不要接觸野生動物。

如果您對於本試驗疫苗有任何的疑問，您可以提出來向您的試驗醫師討論。

（七）試驗預期效益：

依據臨床前試驗結果，預期本試驗疫苗對您可能可以產生抗體，預防新型冠狀病毒感染，但因每個人體質不同也有可能不會產生療效，故參加本試驗可能不會有直接的好處。

但是您參加本試驗，可協助我們獲得更多資訊，以瞭解UB-612疫苗的安全性與免疫力。

（八）試驗進行中受試者之禁忌、限制與應配合之事項：

禁止使用的藥物

以下藥物請勿在試驗期間使用：

- 直到試驗第57天禁止使用免疫抑制劑、或細胞毒性治療
- 到試驗第57天禁止使用免疫球蛋白和/或任何血液製劑
- 整個試驗期間禁止使用試驗產品(包括藥物或疫苗)
- 到試驗第57天禁止使用全身性皮質類固醇(相當於一天使用≥ 20 mg強的松

(prednisone))

- 接種試驗疫苗後14天禁止接種任何季流感疫苗或新型流感疫苗，或後28天禁止接種其他非試驗疫苗。整個試驗期間禁止使用任何已上市的新型冠狀病毒疫苗產品。

允許使用的藥物

若您的藥物或治療必須常規使用，經試驗醫師判斷不會影響本試驗疫苗的安全性，則可以正常使用。您有任何關於在試驗期間可允許使用何種藥物或治療的問題，請詢問您的試驗醫師。

懷孕或母乳哺乳的風險

目前未知本試驗疫苗對於未出生胎兒的影響，因此：

- 您為具生育能力的女性受試者 (除非手術絕育或停經)，或您為男性受試者應於接種疫苗至最後一次疫苗後 3 個月同意進行有效的避孕方式，同意進行有效的避孕方式(例如子宮內節育器、荷爾蒙療法或避孕套)。
- 若您為具生育能力的女性，將請您進行懷孕檢測，結果必須為陰性，方可參與試驗。
- 若您為懷孕的女性，將被告知不可參與本試驗。
- 若您在試驗期間懷孕，請盡速通知試驗人員，並且停止施打本疫苗。
- 基於安全性考量，若您為女性受試者而在試驗期間懷孕，或您為男性受試者而您的性伴侶在試驗期間懷孕(將請您的懷孕性伴侶需簽署另外一份同意書)，您與您的胎兒將會被追蹤監測至分娩，除非另有醫學指示。

您應向您的配偶或性伴侶告知您有參與此試驗與相關風險:

簽名：__________日期：__________________

(九)機密性：

中國醫藥大學附設醫院將依法把任何可辨識您的身分之紀錄與您的個人隱私資料視為機密來處理，不會公開。研究人員將以一個研究代碼代表您的身分，此代碼不會顯示您的姓名、國民身分證統一編號、住址等可識別資料。如果發表試驗/研究結果，您的身分仍將保密。您亦瞭解若簽署同意書即同意您的原始醫療紀錄可直接受監測者、稽核者、研究倫理委員會及主管機關檢閱，以確保臨床試驗/研究過程與數據符合相關法律及法規要

求，上述人員並承諾絕不違反您的身分之機密性。除了上述機構依法有權檢視外，我們會小心維護您的隱私。由於試驗藥物可能同時申請美國臨床試驗，依美國藥品管理規定，試驗結果將公佈於公開的臨床試驗資訊網站：Clinicaltrials.gov (美國)，但您的個人資料仍將保密，該網站只會有試驗之結果摘要，您可以在任何時候搜尋該網站。

在試驗/研究期間，依據計畫類型與您所授權的內容，我們將會蒐集與您有關的病歷資料、醫療紀錄、量表、問卷等資料與資訊，並以一個編號來代替您的名字及相關個人資料。前述資料若為紙本型式，將會與本同意書分開存放於研究機構之上鎖櫃中；若為電子方式儲存或建檔以供統計與分析之用，將會存放於設有密碼與適當防毒軟體之專屬電腦內。這些研究資料與資訊將會保存至藥品於我國上市後至少兩年，若試驗疫苗終止研發則保存至試驗正式停止後至少二年，至多將保存至疫苗上市後或試驗正式停止後二年。

上述資料與資訊若傳輸至國外分析與統計，您仍會獲得與本國法規相符之保障，計畫主持人與相關團隊將盡力確保您的個人資料獲得妥善保護。

（十）損害補償與保險：

1. 如依本研究所訂臨床試驗計畫，因發生不良反應造成損害，由聯亞生技開發股份有限公司負補償責任。但本受試者同意書上所記載之可預期不良反應，不予補償。
2. 如依本研究所訂臨床試驗計畫，因而發生不良反應或損害，贊助廠商將依法負責損害賠償責任。本醫院願意提供專業醫療照顧及醫療諮詢。您不必負擔治療不良反應或損害之必要醫療費用。
3. 除前二項補償及醫療照顧外，本研究不提供其他形式之補償。若您不願意接受這樣的風險，請勿參加試驗。
4. 您不會因為簽署本同意書，而喪失在法律上的任何權利。
5. 本研究有投保責任保險。

（十一）受試者權利：

1. 試驗過程中，與您的健康或是疾病有關，可能影響您繼續接受臨床試驗意願的任何重大發現，都將即時提供給您。
2. 如果您在試驗過程中對試驗工作性質產生疑問，對身為患者之權利有意見或懷疑因參與研究而受害時，可與本院之研究倫理委員會聯絡請求諮詢，其電話號碼為：04-22052121轉1925、1926。
3. 為進行試驗工作，您必須接受黃高彬醫師的照顧。如果您現在或於試驗期間有任何問題或狀況，請不必客氣，可與在中國醫藥大學附設醫院兒童感染科的黃高彬醫師聯絡（24小時聯繫電話：0975-681-950）。
4. 參加試驗研究計畫之補助：本計畫將在每次訪視提供交通費2000元給您，整個試

驗預計給予您12000元。

5. 本同意書一式2份，醫師已將同意書副本交給您，並已完整說明本研究之性質與目的。醫師已回答您有關藥品與研究的問題。

（十二）試驗之退出與中止：

您可自由決定是否參加本試驗；試驗過程中也可隨時撤銷同意，退出試驗，不需任何理由，且不會引起任何不愉快或影響其日後醫師對您的醫療照顧。

計畫主持人或贊助廠商亦可能於必要時中止該試驗之進行。

（十三）簽名：

1. 計畫主持人、或協同主持人已詳細解釋有關本研究計畫中上述研究方法的性質與目的，及可能產生的危險與利益。

   計畫主持人/協同主持人簽名：____________日期：________年____月____日

2. 受試者已詳細瞭解上述研究方法及其所可能產生的危險與利益，有關本試驗計畫的疑問，業經試驗主持人詳細予以解釋。本人同意接受為臨床試驗計畫的自願受試者。

   受試者簽名：____________________________日期：________年____月____日

   法定代理人簽名：________________________日期：________年____月____日

   * 受試者為無行為能力(未滿七歲之未成年人者或禁治產人)，由法定代理人為之；禁治產人，由監護人擔任其法定代理人。

   * 受試者為限制行為人者(滿七歲以上之未成年人)，應得法定代理人之同意。

   有同意權人簽名：________________________日期：________年____月____日

   * 受試者雖非無行為能力或限制行為能力者，但因意識混亂或有精神與智能障礙，而無法進行有效溝通和判斷時，由有同意權之人為之。前項有同意權人為配偶及直系親屬。

3. 見證人

   見證人簽名：_____________________________日期：________年____月____日

   身分證字號：　　　　　　　　　　聯絡電話：

   通訊地址：

   * 受試者、法定代理人或有同意權之人皆無法閱讀時，應由見證人在場參與所有有關受試者同意之討論。並確定受試者、法定代理人或有同意權之人之同意完全出於其自由意願後，應於受試者同意書簽名並載明日期。試驗相關人員不得為見證人。

中國醫藥大學暨附設醫院
受試者同意書
(成年安全確認組)

流程表:

安全確認組

**訪視**	**1**[l]	**2**[l]		**3**		**4**		**5/提早退出試驗**	**6**[f]		**7**		**長期性追蹤**
**檢測項目**	**篩選**	**第一次接種**		**第二次接種**		**追蹤**[f]		**個別解盲**	**第三次接種**[g]		**追蹤**[g]		**第 12 個月追蹤**[h]
**天數**	**-28~-1**	**1**	**8, 15, 22**	**29 ±3 天**	**36, 43**	**57 ±3 天**	**64, 71, 78, 85**	**197 ±15 天**	**197~242**	**第六次訪視後 7 天**	**第六次訪視後 14 天 ±3 天**	**253, 309**	**365 ±45 天**
獲得受試者同意書	X								**X**[g]				
納入/排除條件	X	X											
隨機分派		X											
**接種評估**				X					**X**[g]				
基本資料	X												
醫療病史	X	X											
身體檢查[a]	X	X		X		X		X	**X**[g]		**X**[g]		**X**
生命徵象	X	X		X		X		X	**X**[g]		**X**[g]		
心電圖	X												
**實驗室檢測 (安全性)**													
血液常規檢測[i]	X					X			**X**[g]		**X**[g]		
血液生化學檢測[i]	X					X			**X**[g]		**X**[g]		
免疫檢測[i]	X					X			**X**[g]		**X**[g]		
懷孕檢測[b]		X		X					**X**[g]				

中國醫藥大學暨附設醫院

受試者同意書

（成年安全確認組）

訪視	1[l]	2[l]		3		4		5/提早退出試驗	6[t]		7		長期性追蹤
檢測項目	篩選	第一次接種		第二次接種		追蹤[f]		個別解盲	第三次接種[g]		追蹤[g]		第 12 個月追蹤[h]
天數	-28~-1	1	8, 15, 22	29 ±3 天	36, 43	57 ±3 天	64, 71, 78, 85	197 ±15 天	197~242	第六次訪視後 7 天	第六次訪視後 14 天 ±3 天	253, 309	365 ±45 天
尿液常規檢測[b]	X	X[n]		X[n]		X[n]		X[n]	**X**[g, n]		**X**[g, n]		
**實驗室檢測(探索性試驗)**													
**備血**	**X**					**X**							
**免疫原性**[c]									**X**[g]		**X**[g]		**X**[g]
疫苗接種		X		X					**X**[g]				
指導使用電子日誌卡		X		X					**X**[g]				
電話安全性追蹤[c]			X		X		X			**X**[p, g]		X	
不良事件/特殊不良事件[m]/醫療需求不良事件/嚴重不良事件		X[j]	X[j]	X[j]	X[j]	X[j]	X[j]	X[k]	**X**[k, g]	**X**[k, g]	**X**[k ,g]	X[k]	X[k]
新型冠種病毒感染監測		X	X	X	X	X[d]	X[d]	X[d]	**X**[d, g]	**X**[d, g]	**X**[d, g]	X[d]	X[d]
併用藥物		X		X		X		X[e]	**X**[e, g]		**X**[e, g]		

a: 身高與體重僅在第一次訪視測量。
b: 於第 1, 29 天使用尿液懷孕檢測。若尿液檢測為陽性，應以血清懷孕檢測再次確認。以血清懷孕檢測替代尿液檢測將不視為試驗偏差。蛋白尿將透過尿液常規檢測確認，做為基礎值。

中國醫藥大學暨附設醫院

受試者同意書

（成年安全確認組）

c: 所有受試者將進行電話安全追蹤以監測非預期性不良事件，包括特殊不良事件，和監測新型冠狀病毒感染的症狀。
d: 受試者將在每周(為每 7 天)由手機接獲一條提示以規律監測新型冠狀病毒感染症狀或病徵。記錄疑似新冠病毒感染所使用的藥物至新冠病毒感染頁面。
e: 只記錄醫療需求不良事件和嚴重不良事件之併用藥物。
f: 第二次接種疫苗後第 28 天
g: **針對同意並進行施打第三劑試驗疫苗的疫苗組受試者**
h: 第二次接種疫苗後第十二個月(第 365 天)
i: 安全性實驗室數值包括全血液計數(血紅素、血比容、紅血球計數)、白血球計數、血小板計數、肌酸酐、丙胺酸轉胺酶、天門冬胺酸轉胺酶、總膽紅素、直接膽紅素、高靈敏度 C 反應性蛋白、抗核抗體
j: 主動收集時期
k: 被動監測時期
l: 第一次訪視與第二次訪視可為同一次訪視。
m: 包括接種最後一劑疫苗後 12 個月，可能的免疫媒介醫療狀況(PIMMC)或任何定義為可能的特殊不良事件。新冠病毒感染的併發症也將視為疾病增強事件，將被記錄與通報為特殊不良事件。
n: 若受試者有≥第三級以上的高血壓，受試者將或熱是否有蛋白尿存在或惡化。
o: 備血將被冷凍存放做為 UBI 新型冠狀病毒酵素結合免疫吸附分析法，新型冠狀病毒確認酵素結合免疫吸附分析法，以及未來免疫學研究之用。
p: 若符合資格並接種第三劑疫苗的受試者將進行兩週的日誌卡追蹤紀錄，並將於接種後第 7 天進行電話安全性追蹤。
q: 第五次訪視和第六次訪視可為同一天。

中國醫藥大學暨附設醫院
受試者同意書
（成年免疫第三劑組）

您被邀請參與此研究。此同意書主要是提供您本研究之相關資訊，以便您決定是否參加本研究。計畫主持人或其指定之研究人員會為您說明研究內容並回答您的疑問。您可以提出任何和此研究有關的問題，在您的問題尚未獲得滿意的答覆之前，請不要簽署此同意書。如果您願意參與本研究，此文件將視為您的同意紀錄。即使在您同意後，您可以隨時退出本研究不需任何理由。

<table>
<tr><td colspan="2">計畫名稱
中文：一個評估 UB-612 疫苗對於新型冠狀病毒於青少年、成年和老年健康受試者的免疫原性、安全性與耐受性的第二期、安慰劑控制、隨機分派、觀察者盲性臨床試驗
英文：A Phase II, Placebo-controlled, Randomized, Observer-blind Study to Evaluate the Immunogenicity, Safety, and Tolerability of UB-612 Vaccine against COVID-19 in Adolescent, Younger and Elderly Adult Volunteers</td></tr>
<tr><td>執行單位：中國醫藥大學附設醫院感染科、家庭醫學科</td><td>委託單位/藥廠：聯亞生技開發股份有限公司
研究經費來源：聯亞生技開發股份有限公司
受託研究機構：晉加股份有限公司</td></tr>
<tr><td>計畫主持人：黃高彬</td><td>職稱：主治醫師</td></tr>
<tr><td>協同主持人：林文元</td><td>職稱：主治醫師</td></tr>
<tr><td>協同主持人：林伯昌</td><td>職稱：主治醫師</td></tr>
<tr><td>緊急聯絡人：黃高彬</td><td>電話：0975-681-950</td></tr>
<tr><td>受試者姓名：
性別：
身分證字號：
通訊地址：</td><td>病歷號碼：
出生日期：
聯絡電話：</td></tr>
<tr><td>法定代理人或有同意權人之姓名：
性別：
身分證字號：
通訊地址：</td><td>與受試者關係：
出生日期：
聯絡電話：</td></tr>
<tr><td colspan="2">(一)試驗簡介：
1. 本品/技術資料：</td></tr>
</table>

新型冠狀病毒(SARS-CoV-2)於2019年12月起造成中國湖北省武漢市發現多起病毒性肺炎群聚，隨後於2020年1月底台灣出現第一起境外移入確診個案。此疾病在全球擴散，世界衛生組織宣布將此疫情為「國際關注公共衛生緊急事件」。截至2020年底，全球僅有數間疫苗公司，如美國輝瑞藥廠等，取得緊急使用授權上市。

UB-612疫苗為聯亞生技開發股份有限公司所開發新型冠狀病毒預防性疫苗，疫苗含病毒棘狀融合蛋白和胜肽片段,可產生高親和力抗體與新型冠狀病毒結合,並誘發細胞免疫反應，進而達到預防新型冠狀病毒的感染。

UB-612第一期延伸性試驗顯示，接種第三劑UB-612疫苗可以誘發極高的中和抗體，在目前變種病毒的威脅之下，施打第三劑加強免疫反應，已是許多國家的選擇。

2. 本品上市狀況：

本品仍應用於人體試驗，尚未在我國上市。

3. 本試驗使用的UB-612疫苗對新型冠狀病毒的預防效果仍未確認。

4. 您簽署這份受試者同意書是由於您願意接種第三劑UB-612疫苗。

(二)試驗目的：

主要試驗目的

- 評估UB-612疫苗誘發的新型冠狀病毒中和抗體效價。
- 評估接種UB-612疫苗後的安全性和耐受性。

次要試驗目的

- 評估在試驗期間對於新型冠狀病毒的免疫反應。
- 評估三批獨立批次疫苗的批次免疫一致性。

探索性試驗目的

- 評估UB-612疫苗誘發的T細胞功能。
- 評估UB-612疫苗在年輕受試者的安全性和免疫原性。
- 評估UB-612疫苗的療效。
- 描述UB-612疫苗於確診和/或嚴重感染新型冠狀病毒案例之血液學反應。
- **評估針對SARS-CoV-2抗原的抗體反應。**

中國醫藥大學暨附設醫院
受試者同意書
（成年免疫第三劑組）

(三)試驗之主要納入與排除條件：

中國醫藥大學暨附設醫院執行本研究計畫的醫師或相關研究人員將會與您討論有關參加本研究的必要條件。請您配合必須誠實告知我們您過去的健康情形，若您有不符合參加本研究的情況，將不能參加本研究計畫。

1. 參加本研究計畫的主要條件:

□(1) 您為納入試驗時20~85歲之間健康男性或未懷孕的女性受試者。

□(2) 您為具生育能力的女性與男性應於首次接種疫苗至最後一次疫苗後3個月同意進行有效的避孕方式。可接受的有效避孕方式包括:

□a. 男性或女性以手術方法絕育、植入式避孕、或子宮避孕器。

□b. 注射避孕、避孕藥、避孕貼片、避孕環加上一種屏障避孕法*。

□c. 合併使用兩種屏障避孕法*。

*有效的屏障避孕法為避孕隔膜、男性或女性保險套、避孕海綿或殺精劑(含可殺精化學物質的藥膏或凝膠)。

□(3) 您能理解受試者同意書內容的說明與可能的風險，提供簽名的受試者同意書。

□(4) 您能夠理解與遵從本試驗程序與能夠參與每次訪視。

□(5) 您的耳溫≤ 38.0°C。

□(6) 您依據醫療病史、身體檢查和試驗主持人的臨床判斷為健康受試者**可符合納入試驗資格。經試驗主持人判斷，即便您的病史穩定或且控制良好，但伴隨病情惡化而有提高嚴重新型冠狀病毒感染的風險。

**健康受試者有先前存在的穩定疾病者可以納入試驗，定義為該疾病在納入試驗前12週內沒有惡化至需要治療或住院的顯著變化和在納入試驗6個月內沒有惡化至需要治療或住院的顯著變化。

2. 若您有下列任一情況，您將無法參加本研究計畫:

□(1) 您有接種疫苗後需要醫療介入的過敏性休克、蕁麻疹或其他顯著不良反應的病史。

□(2) 您在篩選時或接種每劑疫苗前已懷孕女性或懷孕檢測為陽性的女性。

□(3) 您為正在哺乳的女性，或計畫從接種第一劑疫苗至最後一劑疫苗後60天哺乳的女性。

☐(4) 您在接種第一劑疫苗前3天內，經試驗主持人判斷，患有任何急性疾病。

☐(5) 您在接種第一劑疫苗前1個月內有重大手術。

☐(6) 您是已知為人類免疫缺乏病毒抗體陽性。

☐(7) 您是已知為活動性B型肝炎或C型肝炎。活動性肝炎定義為肝臟轉胺酶(天門冬胺酸轉胺酶和/或丙胺酸轉胺酶)大於3倍正常值上限和/或總膽紅素大於3倍正常值上限。

☐(8) 您是已知曾暴露於新型冠狀病毒，或曾接受預防新型冠狀病毒、中東呼吸症候群冠狀病毒、嚴重急性呼吸道症候群的試驗或已上市產品。

☐(9) 您有格林-巴利症候群的病史。

☐(10) 您在簽署受試者同意書前12周內參與其他的臨床試驗。

☐(11) 您為免疫缺乏/失調疾病，無論是否由基因缺陷、免疫缺乏症或免疫抑制療法所造成。

☐(12) 您計畫或正在進行抗癌症治療。

☐(13) 您患有血小板異常或其他凝血異常可能造成注射之禁忌症。

☐(14) 您在接種第一劑疫苗前6個月長期接受(≥14天連續使用)免疫抑制劑、皮質類固醇(相當於一天使用≥ 20 mg強的松(prednisone))或細胞毒性治療。

☐(15) 您在接種第一劑疫苗前4個月接受免疫球蛋白和/或任何血液製劑的治療。

☐(16) 您在接種試驗疫苗前14天接種任何季流感疫苗或新型流感疫苗，或前28天接種其他疫苗。

☐(17) 您預期在接種試驗疫苗後14天接種任何季流感疫苗或新型流感疫苗，或後28天接種其他疫苗。

☐(18) 您使用短期(<14天使用)全身性類固醇。應於中斷使用全身性類固醇至少28天後才可使用試驗疫苗。吸入/噴霧性、關節注射、囊內或局部(皮膚或眼用)類固醇可允許使用。

☐(19) 您在篩選期前3個月失血或捐血超過500毫升，或預計在試驗期間內捐血或輸血。

☐(20) 經試驗主持人判斷，您有任何醫療疾病或狀況，可能會影響試驗結果或參與試驗可能會對受試者引發額外風險。

☐(21) 您是直接參與本試驗執行的試驗主持人所屬機構**的試驗團隊**、試驗委託者或受託

研究機構(CRO)的員工。

（四）試驗方法及相關檢驗：

這是一個第二期、觀察者盲性、多中心、隨機分派、安慰劑控制試驗，以評估青少年，成年和老年受試者使用兩劑UB-612疫苗的免疫原性，耐受度和安全性。有一部份的受試者使用UB-612疫苗，而另外一部份的受試者則使用「安慰劑」。所謂「安慰劑」是不含有效成份的疫苗。至於誰使用試驗用藥或誰使用「安慰劑」，則像丟銅板或擲骰子一樣由機率決定，不管是您或是研究醫師都不知道您使用了那一種藥，只有分發跟施打疫苗的試驗人員才知道您使用哪一種疫苗，這叫做觀察者盲性。

總計約有3850位合格成年受試者組成核心組用於申請緊急使用授權，另外大約有385位青少年受試者組成補充組申請額外適應症。所有受試者將以6:1的比例，隨機分派至兩劑100微克劑量組別和安慰劑組，包括462位大於18歲至小於65歲可評估的受試者進入批次分析組。對於免疫分析，至少包括350位可評估的成年受試者(年齡大於18歲至小於65歲)和154位可評估的老年受試者(年齡≥65歲)進行描述性分析。免疫原性的受試者將會先納入試驗。所有的受試者將會納入安全性分析，其中至少770位隨機分派的受試者為≥65歲的分層。青少年組將在核心組招募完畢後，再開始納入試驗。約有385位青少年受試者將以6:1的比例隨機分派，其中包括154位可評估的青少年受試者將收集免疫原性數據，並和成年及老年受試者數據進行比較。

您若參與這個試驗，則為有進行免疫分析檢測的免疫原性或批次一致組。

試驗總共有8個訪視。若您參與本試驗，則至少包括第一次訪視(篩選訪視)、第二次訪視（第1天，基礎值，隨機分派，第一次接種疫苗)、第三次訪視(第29天，第二次接種疫苗)、第四次訪視(第57天)、第五次訪視(第197天)。

在第五次訪視時，預計將進行個別解盲。若個別解盲後，得知您為施打疫苗的受試者，且你有意願且符合資格接種第三劑疫苗，將進入第六次訪視(第197~242天，第三次接種疫苗)，第七次訪視(接種疫苗後第14天)，及第八次訪視(第365天)。

整個試驗期間，預期您將參與試驗最長達13個月。

注意事項

1. 如果您同意參加本試驗，研究人員會請您簽署本份受試者同意書，並確認您符合參加本試驗的條件。
2. 從您參與試驗的當天開始，每次訪視都將有合格的試驗人員執行試驗流程與聯繫。

3. 若您有任何符合新型冠狀病毒感染的定義(您曾於過去 7 天內有出國，或是接觸疑似或確認武漢肺炎之病人，而有下列症狀: 發燒、開始咳嗽或惡化、開始呼吸急促或惡化、寒顫、開始肌肉疼痛或惡化、喉嚨痛、腹瀉、嘔吐、開始味覺/嗅覺異常)，請**依照中央疫情指揮中心規定進行自主健康管理或至指定院所進行篩檢。**
4. 若您在試驗期間感染新型冠狀病毒，將依法通報主管機關。

您是否同意? □是 □否

簽名：__________日期：____________

5. 於試驗期間，您不論任何理由提前退出試驗，試驗研究人員都將安排您完成最後一次的訪視之所有試驗項目。您有權利拒絕此項安排，您的決定不會引起任何影響日後醫師對您的醫療照護。

試驗步驟

第一次訪視(第-28~-1 天) -篩選訪視

在試驗醫師或試驗研究人員為您提供足夠的試驗資訊，並確保您有充分的時間考慮以及詢問任何問題後，您願意讓您參與本試驗，並由您簽署本受試者同意書。在確認您已完成受試者同意書簽署並且您也保有一份副本後，試驗醫師或試驗研究人員將會進行以下試驗程序：

(1) 記錄您簽署受試者同意書的日期
(2) 為您指定一組受試者篩選編號
(3) 確認您是否符合本試驗的納入排除條件
(4) 收集您的個人基本資料（例如生日、年齡及性別）
(5) 記錄您的醫療/用藥病史
(6) 進行身體檢查，包括身高體重
(7) 確認生命徵象
(8) 進行心電圖檢測
(9) 收集尿液檢體進行尿液檢測
(10) 收集血液檢體(共 **15.5** 毫升)，進行下列檢測:
- 常規血液檢測
- 血液生化學檢測
- 免疫學檢測**(抗核抗體)**

- **備血(作為測量新型冠狀病毒血清抗體和相關研究之用)**

<u>第二次訪視(第 1 天)-基礎值，確認符合試驗條件，第一次接種疫苗</u>

(1) 再度確認您是否符合本試驗的納入排除條件
(2) 隨機分派，給予您一組隨機分派號碼
(3) 記錄您的醫療/用藥病史
(4) 進行身體檢查
(5) 確認生命徵象
(6) 進行尿液懷孕檢測（具有生育能力女性）
(7) 若您有產生第三級以上的高血壓，您將被收集尿液檢體以檢測是否有蛋白尿存在或惡化
(8) 收集血液檢體(若不進行 T 細胞檢測，共 20 毫升)，進行下列檢測:
   - 免疫原性檢測，包括 Anti-S1-RBD 免疫球蛋白 G 濃度，和新型冠狀病毒中和抗體效價
   - 若您願意，將進行 T 細胞檢測(需額外抽血 56 毫升)

您是否同意? ☐是 ☐否

簽名：__________日期：____________

(9) 進行第一次疫苗接種(注射部位為非慣用手，採用肌肉注射方式)。接種疫苗後，受試者應留在試驗地點至少 30 分鐘，監測生命徵象和急性過敏症狀。
(10) 將詳細地指導您如何填寫電子日誌卡(包括注射後 7 天內預期性不良事件，14 天內的皮膚過敏反應日誌卡)
(11) 收集併用藥物/治療

<u>第一次電話安全性追蹤(第 8, 15, 22 天)</u>

將與您電話聯繫，以追蹤未預期不良事件和新型冠狀病毒感染症狀。

<u>第三次訪視(第 29± 3 天)-第二次接種疫苗</u>

(1) 進行第二次接種評估(有可能會延遲接種時間)
(2) 進行身體檢查
(3) 確認生命徵象
(4) 進行尿液懷孕檢測（具生育能力的女性）
(5) 若您有產生第三級以上的高血壓，您將被收集尿液檢體以檢測是否有蛋白尿存在或惡化。

(6) 若您為批次一致/免疫原組，將收集血液檢體(共 5 毫升)，進行下列檢測:
- 特定抗原 Anti-S1-RBD 抗體

(7) 進行第二次疫苗接種。
(8) 接種疫苗後，受試者應留在試驗地點至少 30 分鐘，監測生命徵象和急性過敏症狀。
(9) 將詳細地指導您如何填寫電子日誌卡(包括注射後 7 天內預期性不良事件，14 天內的皮膚過敏反應日誌卡)
(10) 收集併用藥物/治療
(11) 記錄上一次訪視至此次訪視之間的不良事件、嚴重不良事件型或新型冠狀病毒感染症狀

第二次電話安全性追蹤(第 36, 43 天)

將與您電話聯繫，以追蹤未預期不良事件和新型冠狀病毒感染症狀。

第四次訪視(第 57±3 天) –追蹤訪視

(1) 進行身體檢查
(2) 確認生命徵象
(3) 若您有產生第三級以上的高血壓，您將被收集尿液檢體以檢測是否有蛋白尿存在或惡化。
(4) 收集血液檢體(若不進行 T 細胞檢測，共 **35.5** 毫升) ，進行下列檢測:
- 免疫**原性**檢測，包括 Anti-S1-RBD 免疫球蛋白 G 濃度、和新型冠狀病毒中和抗體效價
- 若您願意，將進行 T 細胞檢測(需額外抽血 56 毫升)
- 常規血液檢測
- 血液生化學檢測
- 免疫學檢測**(抗核抗體)**
- **備血(作為測量新型冠狀病毒血清抗體和相關研究之用)**

(5) 收集併用藥物/治療
(6) 記錄上一次訪視至此次訪視之間的不良事件、嚴重不良事件或新型冠狀病毒感染症狀
(7) 在此次訪視後，您將每周接獲訊息提醒，以定期監測新型冠狀病毒感染症狀

第三次電話安全性追蹤(第 64, 71, 78, 85 天)

將與您電話聯繫，以追蹤安全性及新型冠狀病毒感染症狀。

第五次訪視(第 197±15 天) –個別解盲

(1) 進行身體檢查

(2) 確認生命徵象

(3) 若您有產生第三級以上的高血壓，您將被收集尿液檢體以檢測是否有蛋白尿存在或惡化。

(4) 將替您個別解盲，告知您接種到疫苗或安慰劑。

(5) 收集血液檢體(共 20 毫升) ，進行下列檢測:

- 免疫**原性**檢測，包括 Anti-S1-RBD 免疫球蛋白 G 濃度、和新型冠狀病毒中和抗體效價

(6) 收集併用藥物/治療

(7) 記錄上一次訪視至此次訪視之間的不良事件、嚴重不良事件或新型冠狀病毒感染症狀

第六次訪視(第 197~242 天，第三次接種疫苗)

(1) 記錄您簽署受試者同意書的日期

(2) 確認您是否符合接種第三劑疫苗的資格，包括第三劑接種疫苗的禁忌症，或有延遲第三劑接種時間的條件

(3) 進行身體檢查

(4) 確認生命徵象

(5) 若您有產生第三級以上的高血壓，您將被收集尿液檢體以檢測是否有蛋白尿存在或惡化

(6) 收集血液檢體(共 20.5 毫升)，進行下列檢測:

- **常規血液檢測**
- **血液生化學檢測**
- **免疫學檢測(抗核抗體)**
- **探索性試驗免疫反應檢測**

(7) 進行尿液懷孕檢測（具有生育能力女性）

(8) 進行第三次疫苗接種。

(9) 接種疫苗後，受試者應留在試驗地點至少 30 分鐘，監測生命徵象和急性過敏症狀。

將詳細地指導您如何填寫電子日誌卡(包括注射後 7 天內預期性不良事件，14 天內的皮膚過敏反應日誌卡)

(10) 收集併用藥物/治療

(11) 進行新型冠狀病毒監測

(12) 記錄上一次訪視至此次訪視之間的不良事件、嚴重不良事件型或新型冠狀病毒感染症狀

第六次訪視後第 7 天電話安全性追蹤

將與您電話聯繫，以追蹤未預期不良事件和新型冠狀病毒感染症狀。此外，還將監測皮膚過敏反應，或其他非預期的過敏反應。若您有發生任何第三級以上的過敏事件，試驗人員可能將安排您額外的回診。

第七次訪視 (第六次訪視後第 14± 3 天)

(1) 進行身體檢查
(2) 確認生命徵象
(3) 若您有產生第三級以上的高血壓，您將被收集尿液檢體以檢測是否有蛋白尿存在或惡化。
(4) 收集血液檢體(共 20.5 毫升)，進行下列檢測:
  - 常規血液檢測
  - 血液生化學檢測
  - 免疫學檢測(抗核抗體)
  - 探索性試驗免疫反應檢測
(5) 收集併用藥物/治療
(6) 進行新型冠狀病毒監測
(7) 記錄上一次訪視至此次訪視之間的不良事件、嚴重不良事件型或新型冠狀病毒感染症狀

追蹤期電話安全性追蹤(第 253, 309 天)

第七次訪視後將每兩個月與您電話聯繫，以追蹤安全性及新型冠狀病毒感染症狀。

第八次訪視(第 365±45 天) –第 12 個月追蹤

(1) 確認生命徵象
(2) 收集血液檢體(共 20 毫升) ，進行下列檢測:
  - 免疫原性檢測，包括 Anti-S1-RBD 免疫球蛋白 G 濃度、和新型冠狀病毒中和抗體效價
(3) 記錄上一次訪視至此次訪視之間的不良事件，包括特殊不良事件、醫療不良事件、嚴重不良事件或新型冠狀病毒感染症狀

中國醫藥大學暨附設醫院
受試者同意書
（成年免疫第三劑組）

受試者之檢體(含其衍生物)之保存、使用與再利用：

1. 檢體及剩餘檢體之保存與使用

(1) 檢體(含其衍生物)之保存與使用

為研究所需，我們所蒐集您的檢體，將依本研究計畫使用，檢體將保存於**聯亞生技開發股份有限公司(試驗委託者)**，直至 20 年保存期限屆滿，我們將依法銷毀。為了保護您的個人隱私，我們將以一個試驗編號來代替您的名字及相關個人資料，以確認您的檢體及與相關資料受到完整保密。如果您對檢體的使用有疑慮，或您有任何想要銷毀檢體的需求，請立即與我們聯絡(聯絡人：黃高彬醫師電話：0975-681-950)，我們即會將您的檢體銷毀。您也可以聯繫中國醫藥大學暨附設醫院研究倫理委員會(電話：04-22052121 轉 1925、1926)，以協助您解決檢體在研究使用上的任何爭議。

(2) 剩餘檢體(含其衍生物)之再利用

您的生物檢體將會以專屬號碼進行編碼並在**聯亞生技開發股份有限公司(試驗委託者)**的控管下儲存最長20年，以研究UB-612 疫苗反應者的生物標記，及改善治療方式。

所有新的研究計畫都要再經由中國醫藥大學暨附設醫院研究倫理委員會審議通過，倫理審查委員會若認定新的研究超出您同意的範圍，將要求我們重新得到您的同意。

是否同意剩餘檢體保留提供未來新型冠狀病毒感染研究之用，並授權中國醫藥大學暨附設醫院研究倫理委員會審議是否需要再取得您的同意(擇一)

☐ 不同意保存我的剩餘檢體，試驗結束後請銷毀

☐ 同意以非去連結之方式保存我的剩餘檢體，逾越原同意使用範圍時，需再次得到我的同意才可使用我的檢體進行新的研究

2. 檢體及剩餘檢體之部分類型(檢體類型可依計畫書內容自行增減)

(1) 一般生化、血液檢驗/病毒檢測檢體

在試驗期間，會將您的檢體送往聯亞生技開發股份有限公司(試驗委託者)委託的中央實驗室中國醫藥大學暨附設醫院，此機構地址為台中市北區育德路2號，和大安聯合醫事檢驗所，此機構地址為台北市大安區復興南路二段151巷33號，中央實驗室會在分析後立即將分析結果提供給試驗中心，若有剩餘的檢體，**將儲存直到至少完成臨床試驗報告為止，最長將保存20年。**

(2) 抗體/細胞免疫試驗

在試驗期間，會將您的檢體送往聯亞生技開發股份有限公司(試驗委託者)分析實驗室。完成試驗後，若有剩餘檢體，將儲存直到至少完成臨床試驗報告為止，最長將保存20年。

(3) 中和試驗(neutralization test, NT)

在試驗期間，會將您的檢體送往聯亞生技開發股份有限公司(試驗委託者)委託的中央實驗室中央研究院進行處置、處理與進一步分析。此機構地址為台北市南港區研究院路二段128號。完成試驗後，若有剩餘檢體，將儲存直到至少完成臨床試驗報告為止，最長將保存20年。

(4) 遺傳學檢體

在試驗期間，若發生嚴重不良反應或特定不良反應，您的檢體將用於HLA分型檢驗，會將您的檢體送往聯亞生技開發股份有限公司(試驗委託者)委託的中央實驗室有勁基因股份有限公司分析，此機構地址為新北市樹林區復興路376-5號，中央實驗室不會將分析結果提供給試驗中心，若有剩餘的檢體，將會儲存直到檢驗結果複驗完畢即銷毀，不會長期儲存。

(5) 探索性試驗檢體

在試驗期間，會將您的檢體送往聯亞生技開發股份有限公司(試驗委託者)委託的實驗室(表一)進行處理或進一步分析。完成試驗後，若有剩餘檢體，將儲存直到至少完成臨床試驗報告為止，最長將保存20年。

表一、實驗室名稱與機構地址

實驗室名稱	機構地址
聯亞生技開發(股)公司	新竹縣竹北市生醫路二段 6-1 號 5 樓
Viroclinics	Rotterdam Science Tower, Marconistraat 16, 3029 AK Rotterdam, The Netherlands(荷蘭)
DASA	Jonas Cruz de Araujo, Diagnostics da America S/A, Surubiju Avenue, 1890, Barueri, SP, Brazil(巴西), 06455-040
PHE Porton Down	Salisbury Wiltshire SP4 0JG, England(英國)
UTMB	University of Texas Medical Branch 301 University Boulevard Keiller Building, Room 2.150 Galveston, Texas, USA(美國)

中國醫藥大學暨附設醫院

受試者同意書

（成年免疫第三劑組）

**Virology**	**University of São Paulo, Brazil Rua Dr EnnEn de Carvalho Aguiar 470, CEP 05403-000 (巴西)**
**VRDL**	**California Department of Public Health, 850 Marina Bay Parkway, Richmond, CA 94804, USA(美國)**
**NEXELIS**	**525 Boul. Cartier Ouest Laval, Qulbec, Canada, H7V 3S8(加拿大)**
**Vaccinology and Immunology Infection, Immunity & Inflammation Dept UCL GOS Institute of Childe Health**	**UCL Great Ormond Street Institute of Child Health 30 Guilford Street London WC1N 1EH, England(英國)**
**VisMederi**	**VisMederi Srl, Strada del Petriccio e Belriguardo, 35, 53100 Siena, Italy(義大利)**

(五)可能產生之副作用、發生率及處理方法：

1. 與試驗藥物相關的風險（本試驗疫苗的副作用）：

<u>冠狀病毒疫苗的開發</u>

過去針對與SARS-CoV-2病毒相同屬於人類冠狀病毒的SARS-CoV(嚴重急性呼吸綜合症冠狀病毒(SARS冠狀病毒))的疫苗研究發現，接種過SARS-CoV疫苗的小鼠在暴露到SARS-CoV後會發生過度免疫反應而產生病變，因此不得不停止這種疫苗的開發。所以，成功的人類冠狀病毒疫苗不只要產生可以抑制病毒的免疫反應，更要避免過度免疫產生的副作用。

<u>疫苗相關的風險: 第一期臨床試驗</u>

接種疫苗可能會出現注射部位的不良反應(例如疼痛、硬化腫脹、皮疹發紅、過敏反應、蜂窩性組織炎)，或全身性不良反應(例如發燒、腹瀉、疲倦、噁心/嘔吐、厭食、咽喉痛、頭痛、咳嗽、關節痛、非注射部位疼痛、非注射部位搔癢、皮膚和黏膜異常、急性過敏反應、昏厥、急性支氣管痙攣、呼吸困難)。

第一期臨床試驗已經有60位受試者接種兩劑疫苗(含10微克、30微克、100微克融合蛋白)，安全實驗室數值並沒有顯示有任何的臨床顯著不正常數值，也沒有發生任何第三級以上與疫苗相關的預期性不良事件。大部分的預期性不良事件都是輕微的，大約於2天之內症狀都會緩解。也沒有任何的嚴重不良事件或特殊不良事件被通報。

<u>疾病增強(disease enhancement) 的風險</u>

SARS-CoV-2候選疫苗也可能會有引發疾病增強(disease enhancement) 的風險，包括抗體依賴性增強(antibody-dependent enhancement)或疫苗相關聯的增強的呼吸道疾病(vaccine-associated enhanced respiratory disease)。在先前研發SARS疫苗時，在數個SARS-CoV動物

攻毒試驗(包括鼠類、雪貂、猴類)當中，有發現疾病增強的現象。疾病增強反應的免疫病理現象包括TH2偏向及嗜酸性白血球的肺部浸潤。但是目前已發表的新型冠狀病毒肺炎疫苗研究，仍尚未發現類似的疾病增強現象。

本試驗疫苗於數個藥理試驗呈現不一致的TH1/TH2 (輔助型T細胞1/輔助型T細胞2)免疫反應偏向，試驗結果並未一致偏向TH2，而由小鼠之SARS-CoV-2動物攻毒試驗結果顯示，本試驗疫苗誘發疾病增強之風險不高。依據文獻指出，組成複雜或容易引起非中和抗體之抗原，如不活化病毒或整片段之蛋白(包含S蛋白與N蛋白)，與易引起偏向Th2免疫反應之佐劑成分，如鋁製佐劑，皆較有可能引起疾病增強。本試驗疫苗的主要抗原為S蛋白上之RBD 區域，已有多篇文獻指出，針對S-RBD設計之SARS與MERS疫苗從未於試驗動物模型上引發疾病增強現象。本試驗疫苗雖使用易引起偏向Th2免疫反應之佐劑，但由動物實驗證實，也同時引起偏向Th1之反應，因此發生疾病增強應屬低風險。且已於多種動物模型中證實，能誘發高效價之中和抗體，於細胞培養中亦能有效抑制新冠病毒感染。

建議您在有效疫苗上市前或本試驗疫苗的產品資訊有進一步更新前，盡量避免暴露於可能感染病毒的環境。研究團隊將會在試驗中執行相關安全性監測。若有任何關於本試驗疫苗與疾病增強風險相關之任何最新資訊，將即時更新並提供給您。

疫苗佐劑相關的風險

本試驗疫苗所使用的佐劑含Adju-Phos®，是屬於一種磷酸鋁類的佐劑。磷酸鋁類佐劑已經使用超過半個世紀，具有相當的安全性。由於此類佐劑可誘導免疫反應，因此可能會造成局部發炎反應，例如在注射部位產生輕微而短暫的疼痛、發紅以及腫脹。

2. 與試驗/研究過程相關的風險：

抽血

本試驗需要抽血檢驗。抽血可能引起一些不適和瘀血。整個試驗期間13個月，共需抽血**157毫升**。若您願意抽血檢驗T細胞檢測，則會再額外抽血共112毫升。若您罹患新型冠狀病毒感染，**可能將於每次額外訪視抽血30毫升。**

在接種疫苗過程中，可能會出現一些尚未在已完成試驗中發現的副作用。一般而言，接種某一新疫苗總是會有一定的風險，但是計畫主持人會採取一切措施預防風險的發生。**計畫主持人鼓勵您報告您遇到的任何不適。**

(六)其他替代療法及說明：

您不是非參加不可，若不參加研究，由於目前尚未有疫苗可用來預防新型冠狀病毒感染，因此預防措施與其他呼吸道感染相同，包括：勤洗手、減少觸摸眼口鼻、注意咳嗽禮節、

妥善處理口鼻分泌物等，避免出入公共場所，並不要接觸野生動物。

如果您對於本試驗疫苗有任何的疑問，您可以提出來向您的試驗醫師討論。

（七）試驗預期效益：

依據臨床前試驗結果，預期本試驗疫苗對您可能可以產生抗體，預防新型冠狀病毒感染，但因每個人體質不同也有可能不會產生療效，故參加本試驗可能不會有直接的好處。

但是您參加本試驗，可協助我們獲得更多資訊，以瞭解UB-612疫苗的安全性與免疫力。

（八）試驗進行中受試者之禁忌、限制與應配合之事項：

禁止使用的藥物

以下藥物請勿在試驗期間使用：

- 直到試驗第197天禁止使用免疫抑制劑、或細胞毒性治療
- 到試驗第197天禁止使用免疫球蛋白和/或任何血液製劑
- 整個試驗期間禁止使用試驗產品(包括藥物或疫苗)
- 到試驗第197天禁止使用全身性皮質類固醇(相當於一天使用≥ 20 mg強的松(prednisone))
- 接種試驗疫苗後14天禁止接種任何季流感疫苗或新型流感疫苗，或後28天禁止接種其他非試驗疫苗。整個試驗期間禁止使用任何已上市的新型冠狀病毒疫苗產品。

允許使用的藥物

若您的藥物或治療必須常規使用，經試驗醫師判斷不會影響本試驗疫苗的免疫原性、臨床療效與安全性，則可以正常使用。您有任何關於在試驗期間可允許使用何種藥物或治療的問題，請詢問您的試驗醫師。

懷孕或母乳哺乳的風險

目前未知本試驗疫苗對於未出生胎兒的影響，因此：

- 您為具生育能力的女性受試者 (除非手術絕育或停經)，或您為男性受試者應於接種疫苗至最後一次疫苗後 3 個月同意進行有效的避孕方式，同意進行有效的避孕方式(例如子宮內節育器、荷爾蒙療法或避孕套)。

中國醫藥大學暨附設醫院

受試者同意書

（成年免疫第三劑組）

- 若您為具生育能力的女性，將請您進行懷孕檢測，結果必須為陰性，方可參與試驗。
- 若您為懷孕的女性，將被告知不可參與本試驗。
- 若您在試驗期間懷孕，請盡速通知試驗人員，並且停止施打本疫苗。
- 基於安全性考量，若您為女性受試者而在試驗期間懷孕，或您為男性受試者而您的性伴侶在試驗期間懷孕(將請您的懷孕性伴侶需簽署另外一份同意書)，您與您的胎兒將會被追蹤監測至分娩，除非另有醫學指示。

您應向您的配偶或性伴侶告知您有參與此試驗與相關風險:

簽名：__________日期：__________________

（九)機密性：

中國醫藥大學附設醫院將依法把任何可辨識您的身分之紀錄與您的個人隱私資料視為機密來處理，不會公開。研究人員將以一個研究代碼代表您的身分，此代碼不會顯示您的姓名、國民身分證統一編號、住址等可識別資料。如果發表試驗/研究結果，您的身分仍將保密。您亦瞭解若簽署同意書即同意您的原始醫療紀錄可直接受監測者、稽核者、研究倫理委員會及主管機關檢閱，以確保臨床試驗/研究過程與數據符合相關法律及法規要求，上述人員並承諾絕不違反您的身分之機密性。除了上述機構依法有權檢視外，我們會小心維護您的隱私。由於試驗藥物可能同時申請美國臨床試驗，依美國藥品管理規定，試驗結果將公佈於公開的臨床試驗資訊網站：Clinicaltrials.gov (美國)，但您的個人資料仍將保密，該網站只會有試驗之結果摘要，您可以在任何時候搜尋該網站。

在試驗/研究期間，依據計畫類型與您所授權的內容，我們將會蒐集與您有關的病歷資料、醫療紀錄、量表、問卷等資料與資訊，並以一個編號來代替您的名字及相關個人資料。前述資料若為紙本型式，將會與本同意書分開存放於研究機構之上鎖櫃中；若為電子方式儲存或建檔以供統計與分析之用，將會存放於設有密碼與適當防毒軟體之專屬電腦內。這些研究資料與資訊將會保存至藥品於我國上市後至少兩年，若試驗疫苗終止研發則保存至試驗正式停止後至少二年，至多將保存至疫苗上市後或試驗正式停止後二年。

上述資料與資訊若傳輸至國外分析與統計，您仍會獲得與本國法規相符之保障，計畫主持人與相關團隊將盡力確保您的個人資料獲得妥善保護。

（十)損害補償與保險：

1. 如依本研究所訂臨床試驗計畫，因發生不良反應造成損害，由聯亞生技開發股份有限公司負補償責任。但本受試者同意書上所記載之可預期不良反應，不予補償。
2. 如依本研究所訂臨床試驗計畫，因而發生不良反應或損害，贊助廠商將依法負責損害賠償責任。本醫院願意提供專業醫療照顧及醫療諮詢。您不必負擔治療不良反應或損害之必要醫療費用。
3. 除前二項補償及醫療照顧外，本研究不提供其他形式之補償。若您不願意接受這樣的風險，請勿參加試驗。
4. 您不會因為簽署本同意書，而喪失在法律上的任何權利。
5. 本研究有投保責任保險。

（十一）受試者權利：

1. 試驗過程中，與您的健康或是疾病有關，可能影響您繼續接受臨床試驗意願的任何重大發現，都將即時提供給您。
2. 如果您在試驗過程中對試驗工作性質產生疑問，對身為患者之權利有意見或懷疑因參與研究而受害時，可與本院之研究倫理委員會聯絡請求諮詢，其電話號碼為：04-22052121轉1925、1926。
3. 為進行試驗工作，您必須接受黃高彬醫師的照顧。如果您現在或於試驗期間有任何問題或狀況，請不必客氣，可與在中國醫藥大學附設醫院兒童感染科的黃高彬醫師聯絡（24小時聯繫電話：0975-681-950）。
4. 參加試驗研究計畫之補助：**本計畫將在每次訪視提供交通費及營養費給您，新增的兩個診次(第六次返診、第七次返診)將各提供費用1500元；整個試驗預計給予您21000元。若您願意參與T細胞檢測研究，將在該次訪視另外提供營養費500元給您。**
5. 本同意書一式2份，醫師已將同意書副本交給您，並已完整說明本研究之性質與目的。醫師已回答您有關藥品與研究的問題。

（十二）試驗之退出與中止：

您可自由決定是否參加本試驗；試驗過程中也可隨時撤銷同意，退出試驗，不需任何理由，且不會引起任何不愉快或影響其日後醫師對您的醫療照顧。

計畫主持人或贊助廠商亦可能於必要時中止該試驗之進行。

（十三）簽名：

1. 計畫主持人、或協同主持人已詳細解釋有關本研究計畫中上述研究方法的性質

與目的，及可能產生的危險與利益。

計畫主持人/協同主持人簽名：____________日期：________年____月____日

2. 受試者已詳細瞭解上述研究方法及其所可能產生的危險與利益，有關本試驗計畫的疑問，業經試驗主持人詳細予以解釋。本人同意接受為臨床試驗計畫的自願受試者。

受試者簽名：___________________________日期：________年____月____日

法定代理人簽名：_______________________日期：________年____月____日

* 受試者為無行為能力(未滿七歲之未成年人者或禁治產人)，由法定代理人為之；禁治產人，由監護人擔任其法定代理人。

* 受試者為限制行為人者(滿七歲以上之未成年人)，應得法定代理人之同意。

有同意權人簽名：_______________________日期：________年____月____日

* 受試者雖非無行為能力或限制行為能力者，但因意識混亂或有精神與智能障礙，而無法進行有效溝通和判斷時，由有同意權之人為之。前項有同意權人為配偶及直系親屬。

3. 見證人

見證人簽名：____________________________日期：________年____月____日

身分證字號：　　　　　　　　　　　聯絡電話：

通訊地址：

* 受試者、法定代理人或有同意權之人皆無法閱讀時，應由見證人在場參與所有有關受試者同意之討論。並確定受試者、法定代理人或有同意權之人之同意完全出於其自由意願後，應於受試者同意書簽名並載明日期。試驗相關人員不得為見證人。

中國醫藥大學暨附設醫院
受試者同意書
（成年免疫第三劑組）

流程表:

批次分析與免疫分析組

**訪視**	**1[m]**	**2[m]**		**3**		**4**		**5/提早退出試驗**	**6[t]**		**7**		**長期性追蹤**
**檢測項目**	**篩選**	**第一次接種**		**第二次接種**		**追蹤[g]**		**個別解盲**	**第三次接種[h]**		**追蹤[h]**		**第12個月追蹤[i]**
**天數**	**-28~-1**	**1**	**8, 15, 22**	**29 ±3天**	**36, 43**	**57 ±3天**	**64, 71, 78,85**	**197 ±15天**	**197~242**	**第六次訪視後7天**	**第六次訪視後14天 ±3天**	**253, 309**	**365 ±45天**
獲得受試者同意書	X								**X[h]**				
納入/排除條件	X	X											
隨機分派		X											
**接種評估**				X					**X[h]**				
基本資料	X												
醫療病史	X	X											
身體檢查[a]	X	X		X		X		X	**X[h]**		**X[h]**		
生命徵象	X	X		X		X		X	**X[h]**		**X[h]**		**X**
心電圖	X												
**實驗室檢測(安全性)**													
血液常規檢測[j]	X					X			**X[h]**		**X[h]**		
血液生化學檢測[j]	X					X			**X[h]**		**X[h]**		
免疫檢測[j]	X					X			**X[h]**		**X[h]**		
懷孕檢測[b]		X		X					**X[h]**				

訪視	1[m]	2[m]		3		4		5/提早退出試驗	6[t]		7		長期性追蹤
**檢測項目**	**篩選**	**第一次接種**		**第二次接種**		**追蹤[g]**		**個別解盲**	**第三次接種[h]**		**追蹤[h]**		**第12個月追蹤[i]**
**天數**	**-28~-1**	**1**	**8, 15, 22**	**29 ±3天**	**36, 43**	**57 ±3天**	**64, 71, 78,85**	**197 ±15天**	**197~242**	**第六次訪視後7天**	**第六次訪視後14天 ±3天**	**253, 309**	**365 ±45天**
尿液常規檢測[b]	X	X[q]		X[q]		X[q]		X[q]	**X**[h, q]		**X**[h, q]		
**實驗室檢測(免疫原性)**													
免疫原性[c]		X		X[n]		X		X					X
T細胞反應(可選擇[o])		X				X							
**實驗室檢測(探索性試驗)**													
**T細胞功能性檢測(可選擇[u])**									**X**[h]		**X**[h]		
**備血**	X					X							
**免疫原性[c]**									**X**[h]		**X**[h]		
疫苗接種		X		X					**X**[h]				
指導使用電子日誌卡		X		X					**X**[h]				
電話安全性追蹤[d]			X		X		X			**X**[s ,h]		X	
不良事件/特殊不良事件[p]/醫		X[k]	X[k]	X[k]	X[k]	X[k]	X[k]	X[l]	**X**[l, h]	**X**[l, h]	**X**[l, h]	X[l]	X[l]

中國醫藥大學暨附設醫院
受試者同意書
（成年免疫第三劑組）

訪視	1[m]	2 [m]		3		4		5/提早退出試驗	6[t]		7		長期性追蹤
檢測項目	篩選	第一次接種		第二次接種		追蹤 [g]		個別解盲	第三次接種 [h]		追蹤 [h]		第 12 個月追蹤 [i]
天數	-28~-1	1	8, 15, 22	29 ±3 天	36, 43	57 ±3 天	64, 71, 78,85	197 ±15 天	197~242	第六次訪視後 7 天	第六次訪視後 14 天 ±3 天	253, 309	365 ±45 天
療需求不良事件/嚴重不良事件													
新型冠種病毒感染監測		X	X	X	X	X [e]	X [e]	X [e]	**X** [e, h]	**X** [e, h]	**X** [e, h]	X [e]	X [e]
併用藥物		X		X		X		X [f]	**X** [f, h]		**X** [f, h]		

a: 身高與體重僅在第一次訪視測量。
b:於第 1, 29 天使用尿液懷孕檢測。若尿液檢測為陽性，應以血清懷孕檢測再次確認。以血清懷孕檢測替代尿液檢測將不視為試驗偏差。蛋白尿將透過尿液常規檢測確認，做為基礎值。
c: 針對 Anti-S1-RBD 免疫球蛋白 G 濃度和新型冠狀病毒中和抗體效價，以及抑制 S1-RBD: ACE2 的抗體效價。
d: 所有受試者將進行電話安全追蹤以監測非預期性不良事件，包括特殊不良事件，和監測新型冠狀病毒感染的症狀。
e: 受試者將在每周(為每 7 天)由手機接獲一條提示以規律監測新型冠狀病毒感染症狀或病徵。記錄疑似新冠病毒感染所使用的藥物至新冠病毒感染頁面。
f: 只記錄醫療需求不良事件和嚴重不良事件之併用藥物。
g: 第二次接種疫苗後第 28 天
h: 僅針對同意並進行施打第三劑試驗疫苗的疫苗組受試者
i: 第二次接種疫苗後第十二個月(第 365 天)
j: 安全性實驗室數值包括全血液計數(血紅素、血比容、紅血球計數)、白血球計數、血小板計數、肌酸酐、丙胺酸轉胺酶、天門冬胺酸轉胺酶、總膽紅素、直接膽紅素、高靈敏度 C 反應性蛋白、抗核抗體
k: 主動收集時期
l: 被動監測時期
m: 第一次訪視與第二次訪視可為同一次訪視。
n: 僅測量 Anti-S1-RBD IgG 濃度
o: 將在選定的試驗地點納入至少 100 位>18-<65 歲的受試者。
p : 包括接種最後一劑疫苗後 12 個月，可能的免疫媒介醫療狀況(PIMMC)或任何定義為可能的特殊不良事件。新冠病毒感染的併發症也將視為疾病增強事件，將被記錄與通報為特殊不良事件。
q: 若受試者有≥第三級以上的高血壓，受試者將確認是否有蛋白尿存在或惡化。
r: 備血將被冷凍存放做為 UBI 新型冠狀病毒酵素結合免疫吸附分析法，新型冠狀病毒確認酵素結合免疫吸附分析法，以及未來免疫學研究之用。
s: 若符合資格並接種第三劑疫苗的受試者將進行兩週的日誌卡追蹤紀錄，並將於接種後第 7 天進行電話安全性追蹤。

t: 第五次訪視和第六次訪視可為同一天。
u: 在選定之試驗地點中，將邀請大約 30 位年齡 >18-<65 歲的受試者和大約 30 位年齡 ≥65 歲的受試者。在適用的情況下，優先邀請曾進行過第 57 天 T 細胞功能評估的受試者。

中國醫藥大學暨附設醫院
受試者同意書
（成年安全確認第三劑組）

您被邀請參與此研究。此同意書主要是提供您本研究之相關資訊，以便您決定是否參加本研究。計畫主持人或其指定之研究人員會為您說明研究內容並回答您的疑問。您可以提出任何和此研究有關的問題，在您的問題尚未獲得滿意的答覆之前，請不要簽署此同意書。如果您願意參與本研究，此文件將視為您的同意紀錄。即使在您同意後，您可以隨時退出本研究不需任何理由。

<table>
<tr><td colspan="2">計畫名稱
中文：一個評估 UB-612 疫苗對於新型冠狀病毒於青少年、成年和老年健康受試者的免疫原性、安全性與耐受性的第二期、安慰劑控制、隨機分派、觀察者盲性臨床試驗
英文：A Phase II, Placebo-controlled, Randomized, Observer-blind Study to Evaluate the Immunogenicity, Safety and Tolerability of UB-612 Vaccine against COVID-19 in Adolescent, Younger and Elderly Adult Volunteers</td></tr>
<tr><td>執行單位：中國醫藥大學附設醫院感染科、家庭醫學科</td><td>委託單位/藥廠：聯亞生技開發股份有限公司
研究經費來源：聯亞生技開發股份有限公司
受託研究機構：晉加股份有限公司</td></tr>
<tr><td>計畫主持人：黃高彬</td><td>職稱：主治醫師</td></tr>
<tr><td>協同主持人：林文元</td><td>職稱：主治醫師</td></tr>
<tr><td>協同主持人：林伯昌</td><td>職稱：主治醫師</td></tr>
<tr><td>緊急聯絡人：黃高彬</td><td>電話：0975-681-950</td></tr>
<tr><td>受試者姓名：
性別：
身分證字號：
通訊地址：</td><td>病歷號碼：
出生日期：
聯絡電話：</td></tr>
<tr><td>法定代理人或有同意權人之姓名：
性別：
身分證字號：
通訊地址：</td><td>與受試者關係：
出生日期：
聯絡電話：</td></tr>
<tr><td colspan="2">(一)試驗簡介：

1. 本品/技術資料：</td></tr>
</table>

新型冠狀病毒(SARS-CoV-2)於2019年12月起造成中國湖北省武漢市發現多起病毒性肺炎群聚，隨後於2020年1月底台灣出現第一起境外移入確診個案。此疾病在全球擴散，世界衛生組織宣布將此疫情為「國際關注公共衛生緊急事件」。截至2020年底，全球僅有數間疫苗公司，如美國輝瑞藥廠等，取得緊急使用授權上市。

UB-612疫苗為聯亞生技開發股份有限公司所開發新型冠狀病毒預防性疫苗，疫苗含病毒棘狀融合蛋白和胜肽片段，可產生高親和力抗體與新型冠狀病毒結合，並誘發細胞免疫反應，進而達到預防新型冠狀病毒的感染。

UB-612第一期延伸性試驗顯示，接種第三劑UB-612疫苗可以誘發極高的中和抗體，在目前變種病毒的威脅之下，施打第三劑加強免疫反應，已是許多國家的選擇。

2. 本品上市狀況：

本品仍應用於人體試驗，尚未在我國上市。

3. 本試驗使用的UB-612疫苗對新型冠狀病毒的預防效果仍未確認。

4. **您簽署這份受試者同意書是由於您願意接種第三劑UB-612疫苗。**

（二)試驗目的：

主要試驗目的

- 評估UB-612疫苗誘發的新型冠狀病毒中和抗體效價。
- 評估接種UB-612疫苗後的安全性和耐受性。

次要試驗目的

- 評估在試驗期間對於新型冠狀病毒的免疫反應。
- 評估三批獨立批次疫苗的批次免疫一致性。

探索性試驗目的

- 評估UB-612疫苗誘發的T細胞功能。
- 評估UB-612疫苗在年輕受試者的安全性和免疫原性。
- 評估UB-612疫苗的療效。
- 描述UB-612疫苗於確診和/或嚴重感染新型冠狀病毒案例之血液學反應。
- **評估針對SARS-CoV-2抗原的抗體反應。**

中國醫藥大學暨附設醫院
受試者同意書
（成年安全確認第三劑組）

（三）試驗之主要納入與排除條件：

中國醫藥大學暨附設醫院執行本研究計畫的醫師或相關研究人員將會與您討論有關參加本研究的必要條件。請您配合必須誠實告知我們您過去的健康情形，若您有不符合參加本研究的情況，將不能參加本研究計畫。

1. 參加本研究計畫的主要條件:

☐(1) 您為納入試驗時20~85歲之間健康男性或未懷孕的女性受試者。

☐(2) 您為具生育能力的女性與男性應於首次接種疫苗至最後一次疫苗後3個月同意進行有效的避孕方式。可接受的有效避孕方式包括:

☐a. 男性或女性以手術方法絕育、植入式避孕、或子宮避孕器。

☐b.注射避孕、避孕藥、避孕貼片、避孕環加上一種屏障避孕法*。

☐c.合併使用兩種屏障避孕法*。

*有效的屏障避孕法為避孕隔膜、男性或女性保險套、避孕海綿或殺精劑(含可殺精化學物質的藥膏或凝膠)。

☐(3) 您能理解受試者同意書內容的說明與可能的風險，提供簽名的受試者同意書。

☐(4) 您能夠理解與遵從本試驗程序與能夠參與每次訪視。

☐(5) 您的耳溫≤ 38.0°C。

☐(6) 您依據醫療病史、身體檢查和試驗主持人的臨床判斷為健康受試者**可符合納入試驗資格。經試驗主持人判斷，即便您的病史穩定或且控制良好，但伴隨病情惡化而有提高嚴重新型冠狀病毒感染的風險。

**健康受試者有先前存在的穩定疾病者可以納入試驗，定義為該疾病在納入試驗前12週內沒有惡化至需要治療或住院的顯著變化和在納入試驗6個月內沒有惡化至需要治療或住院的顯著變化。

2. 若您有下列任一情況，您將無法參加本研究計畫:

☐(1) 您有接種疫苗後需要醫療介入的過敏性休克、蕁麻疹或其他顯著不良反應的病史。

☐(2) 您在篩選時或接種每劑疫苗前已懷孕女性或懷孕檢測為陽性的女性。

☐(3) 您為正在哺乳的女性，或計畫從接種第一劑疫苗至最後一劑疫苗後60天哺乳的女性。

□(4) 您在接種第一劑疫苗前3天內，經試驗主持人判斷，患有任何急性疾病。

□(5) 您在接種第一劑疫苗前1個月內有重大手術。

□(6) 您是已知為人類免疫缺乏病毒抗體陽性。

□(7) 您是已知為活動性B型肝炎或C型肝炎。活動性肝炎定義為肝臟轉胺酶(天門冬胺酸轉胺酶和/或丙胺酸轉胺酶)大於3倍正常值上限和/或總膽紅素大於3倍正常值上限。

□(8) 您是已知曾暴露於新型冠狀病毒，或曾接受預防新型冠狀病毒、中東呼吸症候群冠狀病毒、嚴重急性呼吸道症候群的試驗或已上市產品。

□(9) 您有格林-巴利症候群的病史。

□(10) 您在簽署受試者同意書前12周內參與其他的臨床試驗。

□(11) 您為免疫缺乏/失調疾病，無論是否由基因缺陷、免疫缺乏症或免疫抑制療法所造成。

□(12) 您計畫或正在進行抗癌症治療。

□(13) 您患有血小板異常或其他凝血異常可能造成注射之禁忌症。

□(14) 您在接種第一劑疫苗前6個月長期接受(≥14天連續使用)免疫抑制劑、皮質類固醇(相當於一天使用≥ 20 mg強的松(prednisone))或細胞毒性治療。

□(15) 您在接種第一劑疫苗前4個月接受免疫球蛋白和/或任何血液製劑的治療。

□(16) 您在接種試驗疫苗前14天接種任何季流感疫苗或新型流感疫苗，或前28天接種其他疫苗。

□(17) 您預期在接種試驗疫苗後14天接種任何季流感疫苗或新型流感疫苗，或後28天接種其他疫苗。

□(18) 您使用短期(<14天使用)全身性類固醇。應於中斷使用全身性類固醇至少28天後才可使用試驗疫苗。吸入/噴霧性、關節注射、囊內或局部(皮膚或眼用)類固醇可允許使用。

□(19) 您在篩選期前3個月失血或捐血超過500毫升，或預計在試驗期間內捐血或輸血。

□(20) 經試驗主持人判斷，您有任何醫療疾病或狀況，可能會影響試驗結果或參與試驗可能會對受試者引發額外風險。

□(21) 您是直接參與本試驗執行的試驗主持人所屬機構**的試驗團隊**、試驗委託者或受託

研究機構(CRO)的員工。

(四)試驗方法及相關檢驗：

這是一個第二期、觀察者盲性、多中心、隨機分派、安慰劑控制試驗，以評估青少年，成年和老年受試者使用兩劑UB-612疫苗的免疫原性，耐受度和安全性。有一部份的受試者使用UB-612疫苗，而另外一部份的受試者則使用「安慰劑」。所謂「安慰劑」是不含有效成份的疫苗。至於誰使用試驗用藥或誰使用「安慰劑」，則像丟銅板或擲骰子一樣由機率決定，不管是您或是研究醫師都不知道您使用了那一種藥，只有分發跟施打疫苗的試驗人員才知道您使用哪一種疫苗，這叫做觀察者盲性。

總計約有3850位合格成年受試者組成核心組用於申請緊急使用授權，另外大約有385位青少年受試者組成補充組申請額外適應症。所有受試者將以6:1的比例，隨機分派至兩劑100微克劑量組別和安慰劑組，包括462位大於18歲至小於65歲可評估的受試者進入批次分析組。對於免疫分析，至少包括350位可評估的成年受試者(年齡大於18歲至小於65歲)和154位可評估的老年受試者(年齡≥65歲)進行描述性分析。免疫原性的受試者將會先納入試驗。所有的受試者將會納入安全性分析，其中至少770位隨機分派的受試者為≥65歲的分層。青少年組將在核心組招募完畢後，再開始納入試驗。約有385位青少年受試者將以6:1的比例隨機分派，其中包括154位可評估的青少年受試者將收集免疫原性數據，並和成年及老年受試者數據進行比較。

您若參與這個試驗，則為進行安全性確認的安全確認組。

試驗總共有8個訪視。若您參與本試驗，則至少包括第一次訪視(篩選訪視)、第二次訪視（第1天，基礎值，隨機分派，第一次接種疫苗)、第三次訪視(第29天，第二次接種疫苗)、第四次訪視(第57天)、第五次訪視(第197天)。

在第五次訪視時，預計將進行個別解盲。若個別解盲後，得知您為施打疫苗的受試者，且你有意願且符合資格接種第三劑疫苗，將進入第六次訪視(第197~242天，第三次接種疫苗)，第七次訪視(接種疫苗後第14天)，及第八次訪視(第365天)。

整個試驗期間，預期您將參與試驗最長達13個月。

注意事項

1. 如果您同意參加本試驗，研究人員會請您簽署本份受試者同意書，並確認您符合參加本試驗的條件。
2. 從您參與試驗的當天開始，每次訪視都將有合格的試驗人員執行試驗流程與聯繫。

3. 若您有任何符合新型冠狀病毒感染的定義(您曾於過去 7 天內有出國，或是接觸疑似或確認武漢肺炎之病人，而有下列症狀: 發燒、開始咳嗽或惡化、開始呼吸急促或惡化、寒顫、開始肌肉疼痛或惡化、喉嚨痛、腹瀉、嘔吐、開始味覺/嗅覺異常)，請**依照中央疫情指揮中心規定進行自主健康管理或至指定院所進行篩檢。**

4. 若您在試驗期間感染新型冠狀病毒，將依法通報主管機關。

   您是否同意? □是 □否

   簽名：__________日期：__________

5. 於試驗期間，您不論任何理由提前退出試驗，試驗研究人員都將安排您完成最後一次的訪視之所有試驗項目。您有權利拒絕此項安排，您的決定不會引起任何影響日後醫師對您的醫療照護。

試驗步驟

第一次訪視(第-28~-1 天) -篩選訪視

在試驗醫師或試驗研究人員為您提供足夠的試驗資訊，並確保您有充分的時間考慮以及詢問任何問題後，您願意讓您參與本試驗，並由您簽署本受試者同意書。在確認您已完成受試者同意書簽署並且您也保有一份副本後，試驗醫師或試驗研究人員將會進行以下試驗程序：

(1) 記錄您簽署受試者同意書的日期
(2) 為您指定一組受試者篩選編號
(3) 確認您是否符合本試驗的納入排除條件
(4) 收集您的個人基本資料（例如生日、年齡及性別）
(5) 記錄您的醫療/用藥病史
(6) 進行身體檢查，包括身高體重
(7) 確認生命徵象
(8) 進行心電圖檢測
(9) 收集尿液檢體進行尿液檢測
(10) 收集血液檢體(共 **15.5** 毫升)，進行下列檢測:
- 常規血液檢測
- 血液生化學檢測
- 免疫學檢測**(抗核抗體)**

- **備血(作為測量新型冠狀病毒血清抗體和相關研究之用)**

第二次訪視(第 1 天)-基礎值，確認符合試驗條件，第一次接種疫苗

(1) 再度確認您是否符合本試驗的納入排除條件

(2) 隨機分派，給予您一組隨機分派號碼

(3) 記錄您的醫療/用藥病史

(4) 進行身體檢查

(5) 確認生命徵象

(6) 進行尿液懷孕檢測（具有生育能力女性）

(7) 若您有產生第三級以上的高血壓，您將被收集尿液檢體以檢測是否有蛋白尿存在或惡化

(8) 進行第一次疫苗接種(注射部位為非慣用手，採用肌肉注射方式)。接種疫苗後，受試者應留在試驗地點至少 30 分鐘，監測生命徵象和急性過敏症狀。

(9) 將詳細地指導您如何填寫電子日誌卡(包括注射後 7 天內預期性不良事件，14 天內的皮膚過敏反應日誌卡)

(10) 收集併用藥物/治療

第一次電話安全性追蹤(第 8, 15, 22 天)

將與您電話聯繫，以追蹤未預期不良事件和新型冠狀病毒感染症狀。

第三次訪視(第 29± 3 天)-第二次接種疫苗

(1) 進行第二次接種評估(有可能會延遲接種時間)

(2) 進行身體檢查

(3) 確認生命徵象

(4) 進行尿液懷孕檢測（具生育能力的女性）

(5) 若您有產生第三級以上的高血壓，您將被收集尿液檢體以檢測是否有蛋白尿存在或惡化。

(6) 進行第二次疫苗接種。

(7) 接種疫苗後，受試者應留在試驗地點至少 30 分鐘，監測生命徵象和急性過敏症狀。

(8) 將詳細地指導您如何填寫電子日誌卡(包括注射後 7 天內預期性不良事件，14 天內的皮膚過敏反應日誌卡)

(9) 收集併用藥物/治療

(10) 記錄上一次訪視至此次訪視之間的不良事件、嚴重不良事件型或新型冠狀病毒感染症狀

第二次電話安全性追蹤(第 36, 43 天)

將與您電話聯繫，以追蹤未預期不良事件和新型冠狀病毒感染症狀。

第四次訪視(第 57±3 天) –追蹤訪視

(1) 進行身體檢查
(2) 確認生命徵象
(3) 若您有產生第三級以上的高血壓，您將被收集尿液檢體以檢測是否有蛋白尿存在或惡化。
(4) 收集血液檢體(共 **15.5** 毫升) ，進行下列檢測:
- 常規血液檢測
- 血液生化學檢測
- 免疫學檢測**(抗核抗體)**
- **備血(作為測量新型冠狀病毒血清抗體和相關研究之用)**

(5) 收集併用藥物/治療
(6) 記錄上一次訪視至此次訪視之間的不良事件、嚴重不良事件或新型冠狀病毒感染症狀
(7) 在此次訪視後，您將每周接獲訊息提醒，以定期監測新型冠狀病毒感染症狀至第 197 天。

第三次電話安全性追蹤(第 64, 71, 78, 85 天)

將與您電話聯繫，以追蹤安全性及新型冠狀病毒感染症狀。

第五次訪視(第 197±15 天) –個別解盲

(1) 進行身體檢查
(2) 確認生命徵象
(3) 若您有產生第三級以上的高血壓，您將被收集尿液檢體以檢測是否有蛋白尿存在或惡化。
(4) 將替您個別解盲，告知您接種到疫苗或安慰劑。
(5) 收集併用藥物/治療
(6) 記錄上一次訪視至此次訪視之間的不良事件、嚴重不良事件或新型冠狀病毒感染症狀

第六次訪視(第 197~242 天，第三次接種疫苗)

(1) 記錄您簽署受試者同意書的日期
(2) 確認您是否符合接種第三劑疫苗的資格，包括第三劑接種疫苗的禁忌症，或有延

遲第三劑接種時間的條件

(3) 進行身體檢查

(4) 確認生命徵象

(5) 若您有產生第三級以上的高血壓，您將被收集尿液檢體以檢測是否有蛋白尿存在或惡化

(6) 收集血液檢體(共 20.5 毫升)，進行下列檢測:

- 常規血液檢測
- 血液生化學檢測
- 免疫學檢測(抗核抗體)
- 探索性試驗免疫反應檢測

(7) 進行尿液懷孕檢測（具有生育能力女性）

(8) 進行第三次疫苗接種。

(9) 接種疫苗後，受試者應留在試驗地點至少 30 分鐘，監測生命徵象和急性過敏症狀。

將詳細地指導您如何填寫電子日誌卡(包括注射後 7 天內預期性不良事件，14 天內的皮膚過敏反應日誌卡)

(10) 收集併用藥物/治療

(11) 進行新型冠狀病毒監測

(12) 記錄上一次訪視至此次訪視之間的不良事件、嚴重不良事件型或新型冠狀病毒感染症狀

<u>第六次訪視後第 7 天電話安全性追蹤</u>

將與您電話聯繫，以追蹤未預期不良事件和新型冠狀病毒感染症狀。此外，還將監測皮膚過敏反應，或其他非預期的過敏反應。若您有發生任何第三級以上的過敏事件，試驗人員可能將安排您額外的回診。

<u>第七次訪視 (第六次訪視後第 14± 3 天)</u>

(1) 進行身體檢查

(2) 確認生命徵象

(3) 若您有產生第三級以上的高血壓，您將被收集尿液檢體以檢測是否有蛋白尿存在或惡化。

(4) 收集血液檢體(共 20.5 毫升)，進行下列檢測:

- 常規血液檢測
- 血液生化學檢測

- **免疫學檢測(抗核抗體)**
- **探索性試驗免疫反應檢測**

(5) **收集併用藥物/治療**

(6) **進行新型冠狀病毒監測**

(7) **記錄上一次訪視至此次訪視之間的不良事件、嚴重不良事件型或新型冠狀病毒感染症狀**

追蹤期電話安全性追蹤(第 253, 309 天)

第七次訪視後將每兩個月與您電話聯繫，以追蹤安全性及新型冠狀病毒感染症狀。

第八次訪視(第 365±45 天) –第 12 個月追蹤

(1) 確認生命徵象

(2) **收集血液檢體(共 10 毫升)，進行下列檢測:**

- **探索性試驗免疫反應檢測**

(3) 記錄上一次訪視至此次訪視之間的不良事件，包括特殊不良事件、醫療不良事件、嚴重不良事件或新型冠狀病毒感染症狀

受試者之檢體(含其衍生物)之保存、使用與再利用：

1. 檢體及剩餘檢體之保存與使用

(1) 檢體(含其衍生物)之保存與使用

為研究所需，我們所蒐集您的檢體，將依本研究計畫使用，檢體將保存於**聯亞生技開發股份有限公司(試驗委託者)**，直至 20 年保存期限屆滿，我們將依法銷毀。為了保護您的個人隱私，我們將以一個試驗編號來代替您的名字及相關個人資料，以確認您的檢體及與相關資料受到完整保密。如果您對檢體的使用有疑慮，或您有任何想要銷毀檢體的需求，請立即與我們聯絡(聯絡人：黃高彬醫師電話：0975-681-950），我們即會將您的檢體銷毀。您也可以聯繫中國醫藥大學暨附設醫院研究倫理委員會(電話：04-22052121 轉 1925、1926)，以協助您解決檢體在研究使用上的任何爭議。

(2) 剩餘檢體(含其衍生物)之再利用

您的生物檢體將會以專屬號碼進行編碼並在**聯亞生技開發股份有限公司(試驗委託者)**的控管下儲存最長20年，以研究UB-612 疫苗反應者的生物標記，及改善治療方式。

所有新的研究計畫都要再經由中國醫藥大學暨附設醫院研究倫理委員會審議通過，

倫理審查委員會若認定新的研究超出您同意的範圍，將要求我們重新得到您的同意。

是否同意剩餘檢體保留提供未來新型冠狀病毒感染研究之用，並授權中國醫藥大學暨附設醫院研究倫理委員會審議是否需要再取得您的同意(擇一)

☐ 不同意保存我的剩餘檢體，試驗結束後請銷毀

☐ 同意以非去連結之方式保存我的剩餘檢體，逾越原同意使用範圍時，需再次得到我的同意才可使用我的檢體進行新的研究

2. 檢體及剩餘檢體之部分類型(檢體類型可依計畫書內容自行增減)

(1) 一般生化、血液檢驗/病毒檢測檢體

在試驗期間，會將您的檢體送往聯亞生技開發股份有限公司(試驗委託者)委託的中央實驗室中國醫藥大學暨附設醫院，此機構地址為台中市北區育德路2號，和大安聯合醫事檢驗所，此機構地址為台北市大安區復興南路二段151巷33號，中央實驗室會在分析後立即將分析結果提供給試驗中心，若有剩餘的檢體，**將儲存直到至少完成臨床試驗報告為止，最長將保存20年。**

(2) 抗體/細胞免疫試驗

在試驗期間，會將您的檢體送往聯亞生技開發股份有限公司(試驗委託者)分析實驗室。完成試驗後，若有剩餘檢體，將儲存直到至少完成臨床試驗報告為止，最長將保存20年。

(3) 中和試驗(neutralization test, NT)

在試驗期間，會將您的檢體送往聯亞生技開發股份有限公司(試驗委託者)委託的中央實驗室中央研究院進行處置、處理與進一步分析。此機構地址為台北市南港區研究院路二段 128 號。完成試驗後，若有剩餘檢體，將儲存直到至少完成臨床試驗報告為止，最長將保存 20 年。

(4) 遺傳學檢體

在試驗期間，若發生嚴重不良反應或特定不良反應，您的檢體將用於 HLA 分型檢驗，會將您的檢體送往聯亞生技開發股份有限公司(試驗委託者)委託的中央實驗室有勁基因股份有限公司分析，此機構地址為新北市樹林區復興路 376-5 號，中央實驗室不會將分析結果提供給試驗中心，若有剩餘的檢體，將會儲存直到檢驗結果複驗完畢即銷毀，

不會長期儲存。

(5) 探索性試驗檢體

在試驗期間，會將您的檢體送往聯亞生技開發股份有限公司(試驗委託者)委託的相關實驗室(表一)進行處理或進一步分析。完成試驗後，若有剩餘檢體，將儲存直到至少完成臨床試驗報告為止，最長將保存 20 年。

表一、實驗室名稱與機構地址

實驗室名稱	機構地址
聯亞生技開發(股)公司	新竹縣竹北市生醫路二段 6-1 號 5 樓
Viroclinics	Rotterdam Science Tower, Marconistraat 16, 3029 AK Rotterdam, The Netherlands(荷蘭)
DASA	Jonas Cruz de Araujo, Diagnostics da America S/A, Surubiju Avenue, 1890, Barueri, SP, Brazil(巴西), 06455-040
PHE Porton Down	Salisbury Wiltshire SP4 0JG, England(英國)
UTMB	University of Texas Medical Branch 301 University Boulevard Keiller Building, Room 2.150 Galveston, Texas, USA(美國
Virology	University of São Paulo, Brazil Rua Dr EnnEn de Carvalho Aguiar 470, CEP 05403-000 (巴西)
VRDL	California Department of Public Health, 850 Marina Bay Parkway, Richmond, CA 94804, USA(美國)
NEXELIS	525 Boul. Cartier Ouest Laval, Qulbec, Canada, H7V 3S8(加拿大)
Vaccinology and Immunology Infection, Immunity & Inflammation Dept UCL GOS Institute of Childe Health	UCL Great Ormond Street Institute of Child Health 30 Guilford Street London WC1N 1EH, England(英國)
VisMederi	VisMederi Srl, Strada del Petriccio e Belriguardo, 35, 53100 Siena, Italy(義大利)

(五)可能產生之副作用、發生率及處理方法：

1. 與試驗藥物相關的風險（本試驗疫苗的副作用）：

<u>冠狀病毒疫苗的開發</u>

過去針對與SARS-CoV-2病毒相同屬於人類冠狀病毒的SARS-CoV(嚴重急性呼吸綜合症冠狀病毒(SARS冠狀病毒))的疫苗研究發現，接種過SARS-CoV疫苗的小鼠在暴露到SARS-CoV後會發生過度免疫反應而產生病變，因此不得不停止這種疫苗的開發。所以，

成功的人類冠狀病毒疫苗不只要產生可以抑制病毒的免疫反應，更要避免過度免疫產生的副作用。

疫苗相關的風險: 第一期臨床試驗

接種疫苗可能會出現注射部位的不良反應(例如疼痛、硬化腫脹、皮疹發紅、過敏反應、蜂窩性組織炎)，或全身性不良反應(例如發燒、腹瀉、疲倦、噁心/嘔吐、厭食、咽喉痛、頭痛、咳嗽、關節痛、非注射部位疼痛、非注射部位搔癢、皮膚和黏膜異常、急性過敏反應、昏厥、急性支氣管痙攣、呼吸困難)。

第一期臨床試驗已經有60位受試者接種兩劑疫苗(含10微克、30微克、100微克融合蛋白)，安全實驗室數值並沒有顯示有任何的臨床顯著不正常數值，也沒有發生任何第三級以上與疫苗相關的預期性不良事件。大部分的預期性不良事件都是輕微的，症狀在大約於2天之內都會緩解。試驗中也沒有任何的嚴重不良事件或特殊不良事件被通報。

疾病增強(disease enhancement) 的風險

SARS-CoV-2候選疫苗也可能會有引發疾病增強(disease enhancement) 的風險，包括抗體依賴性增強(antibody-dependent enhancement)或疫苗相關聯的增強的呼吸道疾病(vaccine-associated enhanced respiratory disease)。在先前研發SARS疫苗時，在數個SARS-CoV動物攻毒試驗(包括鼠類、雪貂、猴類)當中，有發現疾病增強的現象。疾病增強反應的免疫病理現象包括TH2偏向及嗜酸性白血球的肺部浸潤。但是目前已發表的新型冠狀病毒肺炎疫苗研究，仍尚未發現類似的疾病增強現象。

本試驗疫苗於數個藥理試驗呈現不一致的TH1/TH2 (輔助型T細胞1/輔助型T細胞2)免疫反應偏向，試驗結果並未一致偏向TH2，而由小鼠之SARS-CoV-2動物攻毒試驗結果顯示，本試驗疫苗誘發疾病增強之風險不高。依據文獻指出，組成複雜或容易引起非中和抗體之抗原，如不活化病毒或整片段之蛋白(包含S蛋白與N蛋白)，與易引起偏向Th2免疫反應之佐劑成分，如鋁製佐劑，皆較有可能引起疾病增強。本試驗疫苗的主要抗原為S蛋白上之RBD 區域，已有多篇文獻指出，針對S-RBD設計之SARS與MERS疫苗從未於試驗動物模型上引發疾病增強現象。本試驗疫苗雖使用易引起偏向Th2免疫反應之佐劑，但由動物實驗證實，也同時引起偏向Th1之反應，因此發生疾病增強應屬低風險。且已於多種動物模型中證實，能誘發高效價之中和抗體，於細胞培養中亦能有效抑制新冠病毒感染。

建議您在有效疫苗上市前或本試驗疫苗的產品資訊有進一步更新前，盡量避免暴露於可能感染病毒的環境。研究團隊將會在試驗中執行相關安全性監測。若有任何關於本試驗疫苗與疾病增強風險相關之任何最新資訊，將即時更新並提供給您。

疫苗佐劑相關的風險

本試驗疫苗所使用的佐劑含Adju-Phos®，是屬於一種磷酸鋁類的佐劑。磷酸鋁類佐劑已

經使用超過半個世紀，具有相當的安全性。由於此類佐劑可誘導免疫反應，因此可能會造成局部發炎反應，例如在注射部位產生輕微而短暫的疼痛、發紅以及腫脹。

2. 與試驗/研究過程相關的風險：

抽血

本試驗需要抽血檢驗。抽血可能引起一些不適和瘀血。整個試驗期間13個月，共需抽血**82毫升**。若您罹患新型冠狀病毒感染，**可能將於每次額外訪視抽血30毫升。**

在接種疫苗過程中，可能會出現一些尚未在已完成試驗中發現的副作用。一般而言，接種某一新疫苗總是會有一定的風險，但是計畫主持人會採取一切措施預防風險的發生。**計畫主持人鼓勵您報告您遇到的任何不適。**

(六)其他替代療法及說明：

您不是非參加不可，若不參加研究，由於目前尚未有疫苗可用來預防新型冠狀病毒感染，因此預防措施與其他呼吸道感染相同，包括：勤洗手、減少觸摸眼口鼻、注意咳嗽禮節、妥善處理口鼻分泌物等，避免出入公共場所，並不要接觸野生動物。

如果您對於本試驗疫苗有任何的疑問，您可以提出來向您的試驗醫師討論。

(七)試驗預期效益：

依據臨床前試驗結果，預期本試驗疫苗對您可能可以產生抗體，預防新型冠狀病毒感染，但因每個人體質不同也有可能不會產生療效，故參加本試驗可能不會有直接的好處。

但是您參加本試驗，可協助我們獲得更多資訊，以瞭解UB-612疫苗的安全性與免疫力。

(八)試驗進行中受試者之禁忌、限制與應配合之事項：

禁止使用的藥物

以下藥物請勿在試驗期間使用：

- 直到試驗第57天禁止使用免疫抑制劑、或細胞毒性治療
- 到試驗第57天禁止使用免疫球蛋白和/或任何血液製劑
- 整個試驗期間禁止使用試驗產品(包括藥物或疫苗)
- 到試驗第57天禁止使用全身性皮質類固醇(相當於一天使用≥ 20 mg強的松(prednisone))

- 接種試驗疫苗後14天禁止接種任何季流感疫苗或新型流感疫苗，或後28天禁止接種其他非試驗疫苗。整個試驗期間禁止使用任何已上市的新型冠狀病毒疫苗產品。

允許使用的藥物

若您的藥物或治療必須常規使用，經試驗醫師判斷不會影響本試驗疫苗的安全性，則可以正常使用。您有任何關於在試驗期間可允許使用何種藥物或治療的問題，請詢問您的試驗醫師。

懷孕或母乳哺乳的風險

目前未知本試驗疫苗對於未出生胎兒的影響，因此：

- 您為具生育能力的女性受試者 (除非手術絕育或停經)，或您為男性受試者應於接種疫苗至最後一次疫苗後 3 個月同意進行有效的避孕方式，同意進行有效的避孕方式(例如子宮內節育器、荷爾蒙療法或避孕套)。
- 若您為具生育能力的女性，將請您進行懷孕檢測，結果必須為陰性，方可參與試驗。
- 若您為懷孕的女性，將被告知不可參與本試驗。
- 若您在試驗期間懷孕，請盡速通知試驗人員，並且停止施打本疫苗。
- 基於安全性考量，若您為女性受試者而在試驗期間懷孕，或您為男性受試者而您的性伴侶在試驗期間懷孕(將請您的懷孕性伴侶需簽署另外一份同意書)，您與您的胎兒將會被追蹤監測至分娩，除非另有醫學指示。

您應向您的配偶或性伴侶告知您有參與此試驗與相關風險:

簽名：__________日期：___________________

（九)機密性：

中國醫藥大學附設醫院將依法把任何可辨識您的身分之紀錄與您的個人隱私資料視為機密來處理，不會公開。研究人員將以一個研究代碼代表您的身分，此代碼不會顯示您的姓名、國民身分證統一編號、住址等可識別資料。如果發表試驗/研究結果，您的身分仍將保密。您亦瞭解若簽署同意書即同意您的原始醫療紀錄可直接受監測者、稽核者、研究倫理委員會及主管機關檢閱，以確保臨床試驗/研究過程與數據符合相關法律及法規要求，上述人員並承諾絕不違反您的身分之機密性。除了上述機構依法有權檢視外，我們

會小心維護您的隱私。由於試驗藥物可能同時申請美國臨床試驗，依美國藥品管理規定，試驗結果將公佈於公開的臨床試驗資訊網站：Clinicaltrials.gov (美國)，但您的個人資料仍將保密，該網站只會有試驗之結果摘要，您可以在任何時候搜尋該網站。

在試驗/研究期間，依據計畫類型與您所授權的內容，我們將會蒐集與您有關的病歷資料、醫療紀錄、量表、問卷等資料與資訊，並以一個編號來代替您的名字及相關個人資料。前述資料若為紙本型式，將會與本同意書分開存放於研究機構之上鎖櫃中；若為電子方式儲存或建檔以供統計與分析之用，將會存放於設有密碼與適當防毒軟體之專屬電腦內。這些研究資料與資訊將會保存至藥品於我國上市後至少兩年，若試驗疫苗終止研發則保存至試驗正式停止後至少二年，至多將保存至疫苗上市後或試驗正式停止後二年。

上述資料與資訊若傳輸至國外分析與統計，您仍會獲得與本國法規相符之保障，計畫主持人與相關團隊將盡力確保您的個人資料獲得妥善保護。

（十）損害補償與保險：

1. 如依本研究所訂臨床試驗計畫，因發生不良反應造成損害，由聯亞生技開發股份有限公司負補償責任。但本受試者同意書上所記載之可預期不良反應，不予補償。
2. 如依本研究所訂臨床試驗計畫，因而發生不良反應或損害，贊助廠商將依法負責損害賠償責任。本醫院願意提供專業醫療照顧及醫療諮詢。您不必負擔治療不良反應或損害之必要醫療費用。
3. 除前二項補償及醫療照顧外，本研究不提供其他形式之補償。若您不願意接受這樣的風險，請勿參加試驗。
4. 您不會因為簽署本同意書，而喪失在法律上的任何權利。
5. 本研究有投保責任保險。

（十一）受試者權利：

1. 試驗過程中，與您的健康或是疾病有關，可能影響您繼續接受臨床試驗意願的任何重大發現，都將即時提供給您。
2. 如果您在試驗過程中對試驗工作性質產生疑問，對身為患者之權利有意見或懷疑因參與研究而受害時，可與本院之研究倫理委員會聯絡請求諮詢，其電話號碼為：04-22052121轉1925、1926。
3. 為進行試驗工作，您必須接受黃高彬醫師的照顧。如果您現在或於試驗期間有任何問題或狀況，請不必客氣，可與在中國醫藥大學附設醫院兒童感染科的黃高彬醫師聯絡（24小時聯繫電話：0975-681-950）。
4. 參加試驗研究計畫之補助：**本計畫將在每次訪視提供交通費及營養費給您，新增的兩個診次(第六次返診、第七次返診)將各提供費用1000元；整個試驗預計給予**

您14000元。<br>5. 本同意書一式2份，醫師已將同意書副本交給您，並已完整說明本研究之性質與目的。醫師已回答您有關藥品與研究的問題。
（十二）試驗之退出與中止：<br>您可自由決定是否參加本試驗；試驗過程中也可隨時撤銷同意，退出試驗，不需任何理由，且不會引起任何不愉快或影響其日後醫師對您的醫療照顧。<br>計畫主持人或贊助廠商亦可能於必要時中止該試驗之進行。
（十三）簽名：<br>1. 計畫主持人、或協同主持人已詳細解釋有關本研究計畫中上述研究方法的性質與目的，及可能產生的危險與利益。<br>計畫主持人/協同主持人簽名：____________日期：________年____月____日<br>2. 受試者已詳細瞭解上述研究方法及其所可能產生的危險與利益，有關本試驗計畫的疑問，業經試驗主持人詳細予以解釋。本人同意接受為臨床試驗計畫的自願受試者。<br>受試者簽名：____________________________日期：________年____月____日<br>法定代理人簽名：________________________日期：________年____月____日<br>* 受試者為無行為能力(未滿七歲之未成年人者或禁治產人)，由法定代理人為之；禁治產人，由監護人擔任其法定代理人。<br>* 受試者為限制行為人者(滿七歲以上之未成年人)，應得法定代理人之同意。<br>有同意權人簽名：________________________日期：________年____月____日<br>* 受試者雖非無行為能力或限制行為能力者，但因意識混亂或有精神與智能障礙，而無法進行有效溝通和判斷時，由有同意權之人為之。前項有同意權人為配偶及直系親屬。<br>3. 見證人<br>見證人簽名：______________________________日期：________年____月____日<br>身分證字號：　　　　　　　　　　　　聯絡電話：<br>通訊地址：<br>* 受試者、法定代理人或有同意權之人皆無法閱讀時，應由見證人在場參與所有有關受試者同意之討論。並確定受試者、法定代理人或有同意權之人之同意完全出於其自由意願後，應於受試者同意書簽名並載明日期。試驗相關人員不得為見證人。

中國醫藥大學暨附設醫院

受試者同意書

（成年安全確認第三劑組）

流程表:

安全確認組

訪視	1[l]	2[l]		3		4		5/提早退出試驗	6[t]		7		長期性追蹤
檢測項目	篩選	第一次接種		第二次接種		追蹤 [f]		個別解盲	第三次接種[g]		追蹤 [g]		第 12 個月追蹤 [h]
天數	-28~-1	1	8, 15, 22	29 ±3 天	36, 43	57 ±3 天	64, 71, 78 ,85	197 ±15 天	197~242	第六次訪視後 7 天	第六次訪視後 14 天 ±3 天	253, 309	365 ±45 天
獲得受試者同意書	X								X [g]				
納入/排除條件	X	X											
隨機分派		X											
**接種評估**				X					X [g]				
基本資料	X												
醫療病史	X	X											
身體檢查 [a]	X	X		X		X		X	X [g]		X [g]		X
生命徵象	X	X		X		X		X	X [g]		X [g]		
心電圖	X												
**實驗室檢測 (安全性)**													
血液常規檢測 [i]	X					X			X[g]		X[g]		
血液生化學檢測 [i]	X					X			X[g]		X[g]		
免疫檢測 [i]	X					X			X[g]		X[g]		

中國醫藥大學暨附設醫院
受試者同意書
（成年安全確認第三劑組）

訪視	1[l]	2[l]		3		4		5/提早退出試驗	6[t]		7		長期性追蹤
**檢測項目**	**篩選**	**第一次接種**		**第二次接種**		**追蹤[f]**		**個別解盲**	**第三次接種[g]**		**追蹤[g]**		**第 12 個月追蹤[h]**
**天數**	**-28~-1**	**1**	**8, 15, 22**	**29 ±3 天**	**36, 43**	**57 ±3 天**	**64, 71, 78 ,85**	**197 ±15 天**	**197~242**	**第六次訪視後 7 天**	**第六次訪視後 14 天 ±3 天**	**253, 309**	**365 ±45 天**
懷孕檢測[b]		X		X					X[g]				
尿液常規檢測[b]	X	X[n]		X[n]		X[n]		X[n]	X[g, n]		X[g, n]		
**實驗室檢測(探索性試驗)**									X		X		X
**備血**	X					X							
**免疫原性[c]**									X[g]		X[g]		X[g]
疫苗接種		X		X					X[g]				
指導使用電子日誌卡		X		X					X[g]				
電話安全性追蹤[c]			X		X		X			X[p, g]		X	
不良事件/特殊不良事件[m]/醫療需求不良事件/嚴重不良事件		X[j]	X[j]	X[j]	X[j]	X[j]	X[j]	X[k]	X[k, g]	X[k, g]	X[k ,g]	X[k]	X[k]
新型冠種病毒感染監測		X	X	X	X	X[d]	X[d]	X[d]	X[d, g]	X[d, g]	X[d, g]	X[d]	X[d]
併用藥物		X		X		X		X[e]	X[e, g]		X[e, g]		

a: 身高與體重僅在第一次訪視測量。
b: 於第 1, 29 天使用尿液懷孕檢測。若尿液檢測為陽性，應以血清懷孕檢測再次確認。以血清懷孕檢測替代尿液檢測將不視為試驗偏差。蛋白尿將透過尿液常規檢測確認，做為基礎值。
c: 所有受試者將進行電話安全追蹤以監測非預期性不良事件，包括特殊不良事件，和監測新型冠狀病毒感染的症狀。
d: 受試者將在每周(為每 7 天)由手機接獲一條提示以規律監測新型冠狀病毒感染症狀或病徵。記錄疑似新冠病毒感染所使用的藥物至新冠病毒感染頁面。
e: 只記錄醫療需求不良事件和嚴重不良事件之併用藥物。
f: 第二次接種疫苗後第 28 天
g: **針對同意並進行施打第三劑試驗疫苗的疫苗組受試者**
h: 第二次接種疫苗後第十二個月(第 365 天)
i: 安全性實驗室數值包括全血液計數(血紅素、血比容、紅血球計數)、白血球計數、血小板計數、肌酸酐、丙胺酸轉胺酶、天門冬胺酸轉胺酶、總膽紅素、直接膽紅素、高靈敏度 C 反應性蛋白、抗核抗體
j: 主動收集時期
k: 被動監測時期
l: 第一次訪視與第二次訪視可為同一次訪視。
m: 包括接種最後一劑疫苗後 12 個月，可能的免疫媒介醫療狀況(PIMMC)或任何定義為可能的特殊不良事件。新冠病毒感染的併發症也將視為疾病增強事件，將被記錄與通報為特殊不良事件。
n: 若受試者有≥第三級以上的高血壓，受試者將或熱是否有蛋白尿存在或惡化。
o: 備血將被冷凍存放做為 UBI 新型冠狀病毒酵素結合免疫吸附分析法，新型冠狀病毒確認酵素結合免疫吸附分析法，以及未來免疫學研究之用。
p: 若符合資格並接種第三劑疫苗的受試者將進行兩週的日誌卡追蹤紀錄，並將於接種後第 7 天進行電話安全性追蹤。
q: 第五次訪視和第六次訪視可為同一天。

中國醫藥大學暨附設醫院
受試者同意書
（成年免疫第三劑組）

黃高彬
2021.10.25

您被邀請參與此研究。此同意書主要是提供您本研究之相關資訊，以便您決定是否參加本研究。計畫主持人或其指定之研究人員會為您說明研究內容並回答您的疑問。您可以提出任何和此研究有關的問題，在您的問題尚未獲得滿意的答覆之前，請不要簽署此同意書。如果您願意參與本研究，此文件將視為您的同意紀錄。即使在您同意後，您可以隨時退出本研究不需任何理由。

計畫名稱<br>中文：一個評估 UB-612 疫苗對於新型冠狀病毒於青少年、成年和老年健康受試者的免疫原性、安全性與耐受性的第二期、安慰劑控制、隨機分派、觀察者盲性臨床試驗<br>英文：A Phase II, Placebo-controlled, Randomized, Observer-blind Study to Evaluate the Immunogenicity, Safety, and Tolerability of UB-612 Vaccine against COVID-19 in Adolescent, Younger and Elderly Adult Volunteers	
執行單位：中國醫藥大學附設醫院感染科、家庭醫學科	委託單位/藥廠：聯亞生技開發股份有限公司<br>研究經費來源：聯亞生技開發股份有限公司<br>受託研究機構：晉加股份有限公司
計畫主持人：黃高彬	職稱：主治醫師
協同主持人：林文元	職稱：主治醫師
協同主持人：林伯昌	職稱：主治醫師
緊急聯絡人：黃高彬	電話：0975-681-950
受試者姓名：<br>性別：<br>身分證字號：<br>通訊地址：	病歷號碼：<br>出生日期：<br>聯絡電話：
法定代理人或有同意權人之姓名：<br>性別：<br>身分證字號：<br>通訊地址：	與受試者關係：<br>出生日期：<br>聯絡電話：
(一)試驗簡介：<br>1. 本品/技術資料：	

新型冠狀病毒(SARS-CoV-2)於2019年12月起造成中國湖北省武漢市發現多起病毒性肺炎群聚，隨後於2020年1月底台灣出現第一起境外移入確診個案。此疾病在全球擴散，世界衛生組織宣布將此疫情為「國際關注公共衛生緊急事件」。截至2020年底，全球僅有數間疫苗公司，如美國輝瑞藥廠等，取得緊急使用授權上市。

UB-612疫苗為聯亞生技開發股份有限公司所開發新型冠狀病毒預防性疫苗，疫苗含病毒棘狀融合蛋白和胜肽片段，可產生高親和力抗體與新型冠狀病毒結合，並誘發細胞免疫反應，進而達到預防新型冠狀病毒的感染。

UB-612第一期延伸性試驗顯示，接種第三劑UB-612疫苗可以誘發極高的中和抗體，在目前變種病毒的威脅之下，施打第三劑加強免疫反應，已是許多國家的選擇。

2. 本品上市狀況：

本品仍應用於人體試驗，尚未在我國上市。

3. 本試驗使用的UB-612疫苗對新型冠狀病毒的預防效果仍未確認。

4. 您簽署這份受試者同意書是由於您願意接種第三劑UB-612疫苗。

(二)試驗目的：

<u>主要試驗目的</u>

- 評估UB-612疫苗誘發的新型冠狀病毒中和抗體效價。
- 評估接種UB-612疫苗後的安全性和耐受性。

<u>次要試驗目的</u>

- 評估在試驗期間對於新型冠狀病毒的免疫反應。
- 評估三批獨立批次疫苗的批次免疫一致性。

<u>探索性試驗目的</u>

- 評估UB-612疫苗誘發的T細胞功能。
- 評估UB-612疫苗在年輕受試者的安全性和免疫原性。
- 評估UB-612疫苗的療效。
- 描述UB-612疫苗於確診和/或嚴重感染新型冠狀病毒案例之血液學反應。
- 評估針對SARS-CoV-2抗原的抗體反應。

中國醫藥大學暨附設醫院
受試者同意書
（成年免疫第三劑組）

(三)試驗之主要納入與排除條件：

中國醫藥大學暨附設醫院執行本研究計畫的醫師或相關研究人員將會與您討論有關參加本研究的必要條件。請您配合必須誠實告知我們您過去的健康情形，若您有不符合參加本研究的情況，將不能參加本研究計畫。

1. 參加本研究計畫的主要條件:

☐(1) 您為納入試驗時20~85歲之間健康男性或未懷孕的女性受試者。

☐(2) 您為具生育能力的女性與男性應於首次接種疫苗至最後一次疫苗後3個月同意進行有效的避孕方式。可接受的有效避孕方式包括:

☐a. 男性或女性以手術方法絕育、植入式避孕、或子宮避孕器。

☐b. 注射避孕、避孕藥、避孕貼片、避孕環加上一種屏障避孕法*。

☐c. 合併使用兩種屏障避孕法*。

*有效的屏障避孕法為避孕隔膜、男性或女性保險套、避孕海綿或殺精劑(含可殺精化學物質的藥膏或凝膠)。

☐(3) 您能理解受試者同意書內容的說明與可能的風險，提供簽名的受試者同意書。

☐(4) 您能夠理解與遵從本試驗程序與能夠參與每次訪視。

☐(5) 您的耳溫≤ 38.0°C。

☐(6) 您依據醫療病史、身體檢查和試驗主持人的臨床判斷為健康受試者**可符合納入試驗資格。經試驗主持人判斷，即便您的病史穩定或且控制良好，但伴隨病情惡化而有提高嚴重新型冠狀病毒感染的風險。

**健康受試者有先前存在的穩定疾病者可以納入試驗，定義為該疾病在納入試驗前12週內沒有惡化至需要治療或住院的顯著變化和在納入試驗6個月內沒有惡化至需要治療或住院的顯著變化。

2. 若您有下列任一情況，您將無法參加本研究計畫:

☐(1) 您有接種疫苗後需要醫療介入的過敏性休克、蕁麻疹或其他顯著不良反應的病史。

☐(2) 您在篩選時或接種每劑疫苗前已懷孕女性或懷孕檢測為陽性的女性。

☐(3) 您為正在哺乳的女性，或計畫從接種第一劑疫苗至最後一劑疫苗後60天哺乳的女性。

☐(4) 您在接種第一劑疫苗前3天內，經試驗主持人判斷，患有任何急性疾病。

☐(5) 您在接種第一劑疫苗前1個月內有重大手術。

☐(6) 您是已知為人類免疫缺乏病毒抗體陽性。

☐(7) 您是已知為活動性B型肝炎或C型肝炎。活動性肝炎定義為肝臟轉胺酶(天門冬胺酸轉胺酶和/或丙胺酸轉胺酶)大於3倍正常值上限和/或總膽紅素大於3倍正常值上限。

☐(8) 您是已知曾暴露於新型冠狀病毒，或曾接受預防新型冠狀病毒、中東呼吸症候群冠狀病毒、嚴重急性呼吸道症候群的試驗或已上市產品。

☐(9) 您有格林-巴利症候群的病史。

☐(10) 您在簽署受試者同意書前12周內參與其他的臨床試驗。

☐(11) 您為免疫缺乏/失調疾病，無論是否由基因缺陷、免疫缺乏症或免疫抑制療法所造成。

☐(12) 您計畫或正在進行抗癌症治療。

☐(13) 您患有血小板異常或其他凝血異常可能造成注射之禁忌症。

☐(14) 您在接種第一劑疫苗前6個月長期接受(≥14天連續使用)免疫抑制劑、皮質類固醇(相當於一天使用≥ 20 mg強的松(prednisone))或細胞毒性治療。

☐(15) 您在接種第一劑疫苗前4個月接受免疫球蛋白和/或任何血液製劑的治療。

☐(16) 您在接種試驗疫苗前14天接種任何季流感疫苗或新型流感疫苗，或前28天接種其他疫苗。

☐(17) 您預期在接種試驗疫苗後14天接種任何季流感疫苗或新型流感疫苗，或後28天接種其他疫苗。

☐(18) 您使用短期(<14天使用)全身性類固醇。應於中斷使用全身性類固醇至少28天後才可使用試驗疫苗。吸入/噴霧性、關節注射、囊內或局部(皮膚或眼用)類固醇可允許使用。

☐(19) 您在篩選期前3個月失血或捐血超過500毫升，或預計在試驗期間內捐血或輸血。

☐(20) 經試驗主持人判斷，您有任何醫療疾病或狀況，可能會影響試驗結果或參與試驗可能會對受試者引發額外風險。

☐(21) 您是直接參與本試驗執行的試驗主持人所屬機構的試驗團隊、試驗委託者或受託

研究機構(CRO)的員工。

（四）試驗方法及相關檢驗：

這是一個第二期、觀察者盲性、多中心、隨機分派、安慰劑控制試驗，以評估青少年，成年和老年受試者使用兩劑UB-612疫苗的免疫原性，耐受度和安全性。有一部份的受試者使用UB-612疫苗，而另外一部份的受試者則使用「安慰劑」。所謂「安慰劑」是不含有效成份的疫苗。至於誰使用試驗用藥或誰使用「安慰劑」，則像丟銅板或擲骰子一樣由機率決定，不管是您或是研究醫師都不知道您使用了那一種藥，只有分發跟施打疫苗的試驗人員才知道您使用哪一種疫苗，這叫做觀察者盲性。

總計約有3850位合格成年受試者組成核心組用於申請緊急使用授權，另外大約有385位青少年受試者組成補充組申請額外適應症。所有受試者將以6:1的比例，隨機分派至兩劑100微克劑量組別和安慰劑組，包括462位大於18歲至小於65歲可評估的受試者進入批次分析組。對於免疫分析，至少包括350位可評估的成年受試者(年齡大於18歲至小於65歲)和154位可評估的老年受試者(年齡≥65歲)進行描述性分析。免疫原性的受試者將會先納入試驗。所有的受試者將會納入安全性分析，其中至少770位隨機分派的受試者為≥65歲的分層。青少年組將在核心組招募完畢後，再開始納入試驗。約有385位青少年受試者將以6:1的比例隨機分派，其中包括154位可評估的青少年受試者將收集免疫原性數據，並和成年及老年受試者數據進行比較。

您若參與這個試驗，則為有進行免疫分析檢測的免疫原性或批次一致組。

試驗總共有8個訪視。若您參與本試驗，則至少包括第一次訪視(篩選訪視)、第二次訪視（第1天，基礎值，隨機分派，第一次接種疫苗)、第三次訪視(第29天，第二次接種疫苗)、第四次訪視(第57天)、第五次訪視(第197天)。

在第五次訪視時，預計將進行個別解盲。若個別解盲後，得知您為施打疫苗的受試者，且你有意願且符合資格接種第三劑疫苗，將進入第六次訪視(第197~242天，第三次接種疫苗)，第七次訪視(接種疫苗後第14天)，及第八次訪視(第365天)。

整個試驗期間，預期您將參與試驗最長達13個月。

注意事項

1. 如果您同意參加本試驗，研究人員會請您簽署本份受試者同意書，並確認您符合參加本試驗的條件。
2. 從您參與試驗的當天開始，每次訪視都將有合格的試驗人員執行試驗流程與聯繫。

3. 若您有任何符合新型冠狀病毒感染的定義(您曾於過去 7 天內有出國，或是接觸疑似或確認武漢肺炎之病人，而有下列症狀: 發燒、開始咳嗽或惡化、開始呼吸急促或惡化、寒顫、開始肌肉疼痛或惡化、喉嚨痛、腹瀉、嘔吐、開始味覺/嗅覺異常)，請依照中央疫情指揮中心規定進行自主健康管理或至指定院所進行篩檢。
4. 若您在試驗期間感染新型冠狀病毒，將依法通報主管機關。

您是否同意? □是 □否

簽名：__________日期：____________

5. 於試驗期間，您不論任何理由提前退出試驗，試驗研究人員都將安排您完成最後一次的訪視之所有試驗項目。您有權利拒絕此項安排，您的決定不會引起任何影響日後醫師對您的醫療照護。

試驗步驟

第一次訪視(第-28~-1 天) -篩選訪視

在試驗醫師或試驗研究人員為您提供足夠的試驗資訊，並確保您有充分的時間考慮以及詢問任何問題後，您願意讓您參與本試驗，並由您簽署本受試者同意書。在確認您已完成受試者同意書簽署並且您也保有一份副本後，試驗醫師或試驗研究人員將會進行以下試驗程序：

(1) 記錄您簽署受試者同意書的日期
(2) 為您指定一組受試者篩選編號
(3) 確認您是否符合本試驗的納入排除條件
(4) 收集您的個人基本資料（例如生日、年齡及性別）
(5) 記錄您的醫療/用藥病史
(6) 進行身體檢查，包括身高體重
(7) 確認生命徵象
(8) 進行心電圖檢測
(9) 收集尿液檢體進行尿液檢測
(10) 收集血液檢體(共 15.5 毫升)，進行下列檢測:
- 常規血液檢測
- 血液生化學檢測
- 免疫學檢測(抗核抗體)

- 備血(作為測量新型冠狀病毒血清抗體和相關研究之用)

<u>第二次訪視(第 1 天)-基礎值，確認符合試驗條件，第一次接種疫苗</u>

(1) 再度確認您是否符合本試驗的納入排除條件
(2) 隨機分派，給予您一組隨機分派號碼
(3) 記錄您的醫療/用藥病史
(4) 進行身體檢查
(5) 確認生命徵象
(6) 進行尿液懷孕檢測（具有生育能力女性）
(7) 若您有產生第三級以上的高血壓，您將被收集尿液檢體以檢測是否有蛋白尿存在或惡化
(8) 收集血液檢體(若不進行 T 細胞檢測，共 20 毫升)，進行下列檢測:
  - 免疫原性檢測，包括 Anti-S1-RBD 免疫球蛋白 G 濃度，和新型冠狀病毒中和抗體效價
  - 若您願意，將進行 T 細胞檢測(需額外抽血 56 毫升)
(9) 進行第一次疫苗接種(注射部位為非慣用手，採用肌肉注射方式)。接種疫苗後，受試者應留在試驗地點至少 30 分鐘，監測生命徵象和急性過敏症狀。
(10) 將詳細地指導您如何填寫電子日誌卡(包括注射後 7 天內預期性不良事件，14 天內的皮膚過敏反應日誌卡)
(11) 收集併用藥物/治療

<u>第一次電話安全性追蹤(第 8, 15, 22 天)</u>

將與您電話聯繫，以追蹤未預期不良事件和新型冠狀病毒感染症狀。

<u>第三次訪視(第 29± 3 天)-第二次接種疫苗</u>

(1) 進行第二次接種評估(有可能會延遲接種時間)
(2) 進行身體檢查
(3) 確認生命徵象
(4) 進行尿液懷孕檢測（具生育能力的女性）
(5) 若您有產生第三級以上的高血壓，您將被收集尿液檢體以檢測是否有蛋白尿存在或惡化。
(6) 若您為批次一致/免疫原組，將收集血液檢體(共 5 毫升)，進行下列檢測:
  - 特定抗原 Anti-S1-RBD 抗體
(7) 進行第二次疫苗接種。
(8) 接種疫苗後，受試者應留在試驗地點至少 30 分鐘，監測生命徵象和急性過敏症狀。

(9) 將詳細地指導您如何填寫電子日誌卡(包括注射後 7 天內預期性不良事件，14 天內的皮膚過敏反應日誌卡)

(10) 收集併用藥物/治療

(11) 記錄上一次訪視至此次訪視之間的不良事件、嚴重不良事件型或新型冠狀病毒感染症狀

<u>第二次電話安全性追蹤(第 36, 43 天)</u>

將與您電話聯繫，以追蹤未預期不良事件和新型冠狀病毒感染症狀。

<u>第四次訪視(第 57±3 天) –追蹤訪視</u>

(1) 進行身體檢查

(2) 確認生命徵象

(3) 若您有產生第三級以上的高血壓，您將被收集尿液檢體以檢測是否有蛋白尿存在或惡化。

(4) 收集血液檢體(若不進行 T 細胞檢測，共 35.5 毫升) ，進行下列檢測:

- 免疫原性檢測，包括 Anti-S1-RBD 免疫球蛋白 G 濃度、和新型冠狀病毒中和抗體效價
- 若您願意，將進行 T 細胞檢測(需額外抽血 56 毫升)
- 常規血液檢測
- 血液生化學檢測
- 免疫學檢測(抗核抗體)
- 備血(作為測量新型冠狀病毒血清抗體和相關研究之用)

(5) 收集併用藥物/治療

(6) 記錄上一次訪視至此次訪視之間的不良事件、嚴重不良事件或新型冠狀病毒感染症狀

(7) 在此次訪視後，您將每周接獲訊息提醒，以定期監測新型冠狀病毒感染症狀

<u>第三次電話安全性追蹤(第 64, 71, 78, 85 天)</u>

將與您電話聯繫，以追蹤安全性及新型冠狀病毒感染症狀。

<u>第五次訪視(第 197±15 天) –個別解盲</u>

(1) 進行身體檢查

(2) 確認生命徵象

(3) 若您有產生第三級以上的高血壓，您將被收集尿液檢體以檢測是否有蛋白尿存在或惡化。

(4) 將替您個別解盲，告知您接種到疫苗或安慰劑。

(5) 收集血液檢體(共 20 毫升) ，進行下列檢測:

- 免疫原性檢測，包括 Anti-S1-RBD 免疫球蛋白 G 濃度、和新型冠狀病毒中和抗體效價

(6) 收集併用藥物/治療

(7) 記錄上一次訪視至此次訪視之間的不良事件、嚴重不良事件或新型冠狀病毒感染症狀

第六次訪視(第 197~242 天，第三次接種疫苗)

(1) 記錄您簽署受試者同意書的日期

(2) 確認您是否符合接種第三劑疫苗的資格，包括第三劑接種疫苗的禁忌症，或有延遲第三劑接種時間的條件

(3) 進行身體檢查

(4) 確認生命徵象

(5) 若您有產生第三級以上的高血壓，您將被收集尿液檢體以檢測是否有蛋白尿存在或惡化

(6) 收集血液檢體(**若不進行 T 細胞檢測，**共 20.5 毫升)，進行下列檢測:

- 常規血液檢測
- 血液生化學檢測
- 免疫學檢測(抗核抗體)
- 探索性試驗免疫反應檢測
- **若您願意，將進行 T 細胞檢測(需額外抽血 56 毫升)**

您是否同意? □是 □否

簽名：___________日期：_____________

(7) 進行尿液懷孕檢測（具有生育能力女性）

(8) 進行第三次疫苗接種。

(9) 接種疫苗後，受試者應留在試驗地點至少 30 分鐘，監測生命徵象和急性過敏症狀。將詳細地指導您如何填寫電子日誌卡(包括注射後 7 天內預期性不良事件，14 天內的皮膚過敏反應日誌卡)

(10) 收集併用藥物/治療

(11) 進行新型冠狀病毒監測

(12) 記錄上一次訪視至此次訪視之間的不良事件、嚴重不良事件型或新型冠狀病毒感染症狀

第六次訪視後第 7 天電話安全性追蹤

將與您電話聯繫，以追蹤未預期不良事件和新型冠狀病毒感染症狀。此外，還將監測皮膚過敏反應，或其他非預期的過敏反應。若您有發生任何第三級以上的過敏事件，試驗人員可能將安排您額外的回診。

第七次訪視 (第六次訪視後第 14± 3 天)

(1) 進行身體檢查
(2) 確認生命徵象
(3) 若您有產生第三級以上的高血壓，您將被收集尿液檢體以檢測是否有蛋白尿存在或惡化。
(4) 收集血液檢體(**若不進行 T 細胞檢測，**共 20.5 毫升)，進行下列檢測:
- 常規血液檢測
- 血液生化學檢測
- 免疫學檢測(抗核抗體)
- 探索性試驗免疫反應檢測
- **若您願意，將進行 T 細胞檢測(需額外抽血 56 毫升)**

(5) 收集併用藥物/治療
(6) 進行新型冠狀病毒監測
(7) 記錄上一次訪視至此次訪視之間的不良事件、嚴重不良事件型或新型冠狀病毒感染症狀

追蹤期電話安全性追蹤(第 253, 309 天)

第七次訪視後將每兩個月與您電話聯繫，以追蹤安全性及新型冠狀病毒感染症狀。

第八次訪視(第 365±45 天) –第 12 個月追蹤

(1) 確認生命徵象
(2) 收集血液檢體(共 20 毫升) ，進行下列檢測:
- 免疫原性檢測，包括 Anti-S1-RBD 免疫球蛋白 G 濃度、和新型冠狀病毒中和抗體效價

(3) 記錄上一次訪視至此次訪視之間的不良事件，包括特殊不良事件、醫療不良事件、嚴重不良事件或新型冠狀病毒感染症狀

受試者之檢體(含其衍生物)之保存、使用與再利用：

1. 檢體及剩餘檢體之保存與使用

(1) 檢體(含其衍生物)之保存與使用

為研究所需，我們所蒐集您的檢體，將依本研究計畫使用，檢體將保存於聯亞生技開發股份有限公司(試驗委託者)，直至 20 年保存期限屆滿，我們將依法銷毀。為了保護您的個人隱私，我們將以一個試驗編號來代替您的名字及相關個人資料，以確認您的檢體及與相關資料受到完整保密。如果您對檢體的使用有疑慮，或您有任何想要銷毀檢體的需求，請立即與我們聯絡(聯絡人：黃高彬醫師電話：0975-681-950)，我們即會將您的檢體銷毀。您也可以聯繫中國醫藥大學暨附設醫院研究倫理委員會(電話：04-22052121 轉 1925、1926)，以協助您解決檢體在研究使用上的任何爭議。

(2) 剩餘檢體(含其衍生物)之再利用

您的生物檢體將會以專屬號碼進行編碼並在聯亞生技開發股份有限公司(試驗委託者)的控管下儲存最長20年，以研究UB-612 疫苗反應者的生物標記，及改善治療方式。

所有新的研究計畫都要再經由中國醫藥大學暨附設醫院研究倫理委員會審議通過，倫理審查委員會若認定新的研究超出您同意的範圍，將要求我們重新得到您的同意。

是否同意剩餘檢體保留提供未來新型冠狀病毒感染研究之用，並授權中國醫藥大學暨附設醫院研究倫理委員會審議是否需要再取得您的同意(擇一)

☐ 不同意保存我的剩餘檢體，試驗結束後請銷毀

☐ 同意以非去連結之方式保存我的剩餘檢體，逾越原同意使用範圍時，需再次得到我的同意才可使用我的檢體進行新的研究

2. 檢體及剩餘檢體之部分類型(檢體類型可依計畫書內容自行增減)

(1) 一般生化、血液檢驗/病毒檢測檢體

在試驗期間，會將您的檢體送往聯亞生技開發股份有限公司(試驗委託者)委託的中央實驗室中國醫藥大學暨附設醫院，此機構地址為台中市北區育德路2號，和大安聯合醫事檢驗所，此機構地址為台北市大安區復興南路二段151巷33號，中央實驗室會在分析後立即將分析結果提供給試驗中心，若有剩餘的檢體，將儲存直到至少完成臨床試驗報告為止，最長將保存20年。

(2) 抗體/細胞免疫試驗

在試驗期間，會將您的檢體送往聯亞生技開發股份有限公司(試驗委託者)分析實驗室。完成試驗後，若有剩餘檢體，將儲存直到至少完成臨床試驗報告為止，最長將保存20年。

(3) 中和試驗(neutralization test, NT)

在試驗期間，會將您的檢體送往聯亞生技開發股份有限公司(試驗委託者)委託的中央實驗室中央研究院進行處置、處理與進一步分析。此機構地址為台北市南港區研究院路二段128號。完成試驗後，若有剩餘檢體，將儲存直到至少完成臨床試驗報告為止，最長將保存20年。

(4) 遺傳學檢體

在試驗期間，若發生嚴重不良反應或特定不良反應，您的檢體將用於HLA分型檢驗，會將您的檢體送往聯亞生技開發股份有限公司(試驗委託者)委託的中央實驗室有勁基因股份有限公司分析，此機構地址為新北市樹林區復興路376-5號，中央實驗室不會將分析結果提供給試驗中心，若有剩餘的檢體，將會儲存直到檢驗結果複驗完畢即銷毀，不會長期儲存。

(5) 探索性試驗檢體

在試驗期間，會將您的檢體送往聯亞生技開發股份有限公司(試驗委託者)委託的實驗室(表一)進行處理或進一步分析。完成試驗後，若有剩餘檢體，將儲存直到至少完成臨床試驗報告為止，最長將保存20年。

表一、實驗室名稱與機構地址

實驗室名稱	機構地址
聯亞生技開發(股)公司	新竹縣竹北市生醫路二段 6-1 號 5 樓
Viroclinics	Rotterdam Science Tower, Marconistraat 16, 3029 AK Rotterdam, The Netherlands(荷蘭)
DASA	Jonas Cruz de Araujo, Diagnostics da America S/A, Surubiju Avenue, 1890, Barueri, SP, Brazil(巴西), 06455-040
PHE Porton Down	Salisbury Wiltshire SP4 0JG, England(英國)
UTMB	University of Texas Medical Branch 301 University Boulevard Keiller Building, Room 2.150 Galveston, Texas, USA(美國
Virology	University of São Paulo, Brazil Rua Dr EnnEn de Carvalho Aguiar 470, CEP 05403-000 (巴西)

VRDL	California Department of Public Health, 850 Marina Bay Parkway, Richmond, CA 94804, USA(美國)
NEXELIS	525 Boul. Cartier Ouest Laval, Qulbec, Canada, H7V 3S8(加拿大)
Vaccinology and Immunology Infection, Immunity & Inflammation Dept UCL GOS Institute of Childe Health	UCL Great Ormond Street Institute of Child Health 30 Guilford Street London WC1N 1EH, England(英國)
VisMederi	VisMederi Srl, Strada del Petriccio e Belriguardo, 35, 53100 Siena, Italy(義大利)

(五)可能產生之副作用、發生率及處理方法：

1. 與試驗藥物相關的風險（本試驗疫苗的副作用）：

<u>冠狀病毒疫苗的開發</u>

過去針對與SARS-CoV-2病毒相同屬於人類冠狀病毒的SARS-CoV(嚴重急性呼吸綜合症冠狀病毒(SARS冠狀病毒))的疫苗研究發現，接種過SARS-CoV疫苗的小鼠在暴露到SARS-CoV後會發生過度免疫反應而產生病變，因此不得不停止這種疫苗的開發。所以，成功的人類冠狀病毒疫苗不只要產生可以抑制病毒的免疫反應，更要避免過度免疫產生的副作用。

<u>疫苗相關的風險: 第一期臨床試驗</u>

接種疫苗可能會出現注射部位的不良反應(例如疼痛、硬化腫脹、皮疹發紅、過敏反應、蜂窩性組織炎)，或全身性不良反應(例如發燒、腹瀉、疲倦、噁心/嘔吐、厭食、咽喉痛、頭痛、咳嗽、關節痛、非注射部位疼痛、非注射部位搔癢、皮膚和黏膜異常、急性過敏反應、昏厥、急性支氣管痙攣、呼吸困難)。

第一期臨床試驗已經有60位受試者接種兩劑疫苗(含10微克、30微克、100微克融合蛋白)，安全實驗室數值並沒有顯示有任何的臨床顯著不正常數值，也沒有發生任何第三級以上與疫苗相關的預期性不良事件。大部分的預期性不良事件都是輕微的，大約於2天之內症狀都會緩解。也沒有任何的嚴重不良事件或特殊不良事件被通報。

<u>疾病增強(disease enhancement) 的風險</u>

SARS-CoV-2候選疫苗也可能會有引發疾病增強(disease enhancement) 的風險，包括抗體依賴性增強(antibody-dependent enhancement)或疫苗相關聯的增強的呼吸道疾病(vaccine-associated enhanced respiratory disease)。在先前研發SARS疫苗時，在數個SARS-CoV動物攻毒試驗(包括鼠類、雪貂、猴類)當中，有發現疾病增強的現象。疾病增強反應的免疫病理現象包括TH2偏向及嗜酸性白血球的肺部浸潤。但是目前已發表的新

型冠狀病毒肺炎疫苗研究，仍尚未發現類似的疾病增強現象。

本試驗疫苗於數個藥理試驗呈現不一致的TH1/TH2 (輔助型T細胞1/輔助型T細胞2)免疫反應偏向，試驗結果並未一致偏向TH2，而由小鼠之SARS-CoV-2動物攻毒試驗結果顯示，本試驗疫苗誘發疾病增強之風險不高。依據文獻指出，組成複雜或容易引起非中和抗體之抗原，如不活化病毒或整片段之蛋白(包含S蛋白與N蛋白)，與易引起偏向Th2免疫反應之佐劑成分，如鋁製佐劑，皆較有可能引起疾病增強。本試驗疫苗的主要抗原為S蛋白上之RBD 區域，已有多篇文獻指出，針對S-RBD設計之SARS與MERS疫苗從未於試驗動物模型上引發疾病增強現象。本試驗疫苗雖使用易引起偏向Th2免疫反應之佐劑，但由動物實驗證實，也同時引起偏向Th1之反應，因此發生疾病增強應屬低風險。且已於多種動物模型中證實，能誘發高效價之中和抗體，於細胞培養中亦能有效抑制新冠病毒感染。

建議您在有效疫苗上市前或本試驗疫苗的產品資訊有進一步更新前，盡量避免暴露於可能感染病毒的環境。研究團隊將會在試驗中執行相關安全性監測。若有任何關於本試驗疫苗與疾病增強風險相關之任何最新資訊，將即時更新並提供給您。

疫苗佐劑相關的風險

本試驗疫苗所使用的佐劑含Adju-Phos®，是屬於一種磷酸鋁類的佐劑。磷酸鋁類佐劑已經使用超過半個世紀，具有相當的安全性。由於此類佐劑可誘導免疫反應，因此可能會造成局部發炎反應，例如在注射部位產生輕微而短暫的疼痛、發紅以及腫脹。

2. 與試驗/研究過程相關的風險：

抽血

本試驗需要抽血檢驗。抽血可能引起一些不適和瘀血。整個試驗期間13個月，共需抽血157毫升。若您願意抽血檢驗T細胞檢測，則會**於該次訪視每次額外抽血56毫升**。若您罹患新型冠狀病毒感染，可能將於每次額外訪視抽血30毫升。

在接種疫苗過程中，可能會出現一些尚未在已完成試驗中發現的副作用。一般而言，接種某一新疫苗總是會有一定的風險，但是計畫主持人會採取一切措施預防風險的發生。**計畫主持人鼓勵您報告您遇到的任何不適。**

(六)其他替代療法及說明：

您不是非參加不可，若不參加研究，由於目前尚未有疫苗可用來預防新型冠狀病毒感染，因此預防措施與其他呼吸道感染相同，包括：勤洗手、減少觸摸眼口鼻、注意咳嗽禮節、妥善處理口鼻分泌物等，避免出入公共場所，並不要接觸野生動物。

如果您對於本試驗疫苗有任何的疑問，您可以提出來向您的試驗醫師討論。

(七)試驗預期效益：

依據臨床前試驗結果，預期本試驗疫苗對您可能可以產生抗體，預防新型冠狀病毒感染，但因每個人體質不同也有可能不會產生療效，故參加本試驗可能不會有直接的好處。

但是您參加本試驗，可協助我們獲得更多資訊，以瞭解UB-612疫苗的安全性與免疫力。

(八)試驗進行中受試者之禁忌、限制與應配合之事項：

禁止使用的藥物

以下藥物請勿在試驗期間使用：

- 直到試驗第197天禁止使用免疫抑制劑、或細胞毒性治療
- 到試驗第197天禁止使用免疫球蛋白和/或任何血液製劑
- 整個試驗期間禁止使用試驗產品(包括藥物或疫苗)
- 到試驗第197天禁止使用全身性皮質類固醇(相當於一天使用≥ 20 mg強的松(prednisone))
- 接種試驗疫苗後14天禁止接種任何季流感疫苗或新型流感疫苗，或後28天禁止接種其他非試驗疫苗。整個試驗期間禁止使用任何已上市的新型冠狀病毒疫苗產品。

允許使用的藥物

若您的藥物或治療必須常規使用，經試驗醫師判斷不會影響本試驗疫苗的免疫原性、臨床療效與安全性，則可以正常使用。您有任何關於在試驗期間可允許使用何種藥物或治療的問題，請詢問您的試驗醫師。

懷孕或母乳哺乳的風險

目前未知本試驗疫苗對於未出生胎兒的影響，因此：

- 您為具生育能力的女性受試者 (除非手術絕育或停經)，或您為男性受試者應於接種疫苗至最後一次疫苗後 3 個月同意進行有效的避孕方式，同意進行有效的避孕方式(例如子宮內節育器、荷爾蒙療法或避孕套)。
- 若您為具生育能力的女性，將請您進行懷孕檢測，結果必須為陰性，方可參與試驗。

- 若您為懷孕的女性，將被告知不可參與本試驗。
- 若您在試驗期間懷孕，請盡速通知試驗人員，並且停止施打本疫苗。
- 基於安全性考量，若您為女性受試者而在試驗期間懷孕，或您為男性受試者而您的性伴侶在試驗期間懷孕(將請您的懷孕性伴侶需簽署另外一份同意書)，您與您的胎兒將會被追蹤監測至分娩，除非另有醫學指示。

您應向您的配偶或性伴侶告知您有參與此試驗與相關風險:

簽名：__________日期：___________________

（九）機密性：

中國醫藥大學附設醫院將依法把任何可辨識您的身分之紀錄與您的個人隱私資料視為機密來處理，不會公開。研究人員將以一個研究代碼代表您的身分，此代碼不會顯示您的姓名、國民身分證統一編號、住址等可識別資料。如果發表試驗/研究結果，您的身分仍將保密。您亦瞭解若簽署同意書即同意您的原始醫療紀錄可直接受監測者、稽核者、研究倫理委員會及主管機關檢閱，以確保臨床試驗/研究過程與數據符合相關法律及法規要求，上述人員並承諾絕不違反您的身分之機密性。除了上述機構依法有權檢視外，我們會小心維護您的隱私。由於試驗藥物可能同時申請美國臨床試驗，依美國藥品管理規定，試驗結果將公佈於公開的臨床試驗資訊網站：Clinicaltrials.gov (美國)，但您的個人資料仍將保密，該網站只會有試驗之結果摘要，您可以在任何時候搜尋該網站。
在試驗/研究期間，依據計畫類型與您所授權的內容，我們將會蒐集與您有關的病歷資料、醫療紀錄、量表、問卷等資料與資訊，並以一個編號來代替您的名字及相關個人資料。前述資料若為紙本型式，將會與本同意書分開存放於研究機構之上鎖櫃中；若為電子方式儲存或建檔以供統計與分析之用，將會存放於設有密碼與適當防毒軟體之專屬電腦內。這些研究資料與資訊將會保存至藥品於我國上市後至少兩年，若試驗疫苗終止研發則保存至試驗正式停止後至少二年，至多將保存至疫苗上市後或試驗正式停止後二年。上述資料與資訊若傳輸至國外分析與統計，您仍會獲得與本國法規相符之保障，計畫主持人與相關團隊將盡力確保您的個人資料獲得妥善保護。

（十）損害補償與保險：

1. 如依本研究所訂臨床試驗計畫，因發生不良反應造成損害，由聯亞生技開發股份

有限公司負補償責任。但本受試者同意書上所記載之可預期不良反應，不予補償。

2. 如依本研究所訂臨床試驗計畫，因而發生不良反應或損害，贊助廠商將依法負責損害賠償責任。本醫院願意提供專業醫療照顧及醫療諮詢。您不必負擔治療不良反應或損害之必要醫療費用。
3. 除前二項補償及醫療照顧外，本研究不提供其他形式之補償。若您不願意接受這樣的風險，請勿參加試驗。
4. 您不會因為簽署本同意書，而喪失在法律上的任何權利。
5. 本研究有投保責任保險。

（十一）受試者權利：

1. 試驗過程中，與您的健康或是疾病有關，可能影響您繼續接受臨床試驗意願的任何重大發現，都將即時提供給您。
2. 如果您在試驗過程中對試驗工作性質產生疑問，對身為患者之權利有意見或懷疑因參與研究而受害時，可與本院之研究倫理委員會聯絡請求諮詢，其電話號碼為：04-22052121轉1925、1926。
3. 為進行試驗工作，您必須接受黃高彬醫師的照顧。如果您現在或於試驗期間有任何問題或狀況，請不必客氣，可與在中國醫藥大學附設醫院兒童感染科的黃高彬醫師聯絡（24小時聯繫電話：0975-681-950）。
4. 參加試驗研究計畫之補助：本計畫將在每次訪視提供交通費及營養費給您，新增的兩個診次(第六次返診、第七次返診)將各提供費用1500元；整個試驗預計給予您21000元。若您願意參與T細胞檢測研究，將在該次訪視另外提供營養費500元給您。
5. 本同意書一式2份，醫師已將同意書副本交給您，並已完整說明本研究之性質與目的。醫師已回答您有關藥品與研究的問題。

（十二）試驗之退出與中止：

您可自由決定是否參加本試驗；試驗過程中也可隨時撤銷同意，退出試驗，不需任何理由，且不會引起任何不愉快或影響其日後醫師對您的醫療照顧。

計畫主持人或贊助廠商亦可能於必要時中止該試驗之進行。

（十三）簽名：

1. 計畫主持人、或協同主持人已詳細解釋有關本研究計畫中上述研究方法的性質與目的，及可能產生的危險與利益。

   計畫主持人/協同主持人簽名：____________日期：________年____月____日
2. 受試者已詳細瞭解上述研究方法及其所可能產生的危險與利益，有關本試驗計畫

的疑問，業經試驗主持人詳細予以解釋。本人同意接受為臨床試驗計畫的自願受試者。

受試者簽名：______________________________日期：_________年____月____日

法定代理人簽名：__________________________日期：_________年____月____日

* 受試者為無行為能力(未滿七歲之未成年人者或禁治產人)，由法定代理人為之；禁治產人，由監護人擔任其法定代理人。

* 受試者為限制行為人者(滿七歲以上之未成年人)，應得法定代理人之同意。

有同意權人簽名：__________________________日期：_________年____月____日

* 受試者雖非無行為能力或限制行為能力者，但因意識混亂或有精神與智能障礙，而無法進行有效溝通和判斷時，由有同意權之人為之。前項有同意權人為配偶及直系親屬。

3. 見證人

見證人簽名：______________________________日期：_________年____月____日

身分證字號：　　　　　　　　　　　　聯絡電話：

通訊地址：

* 受試者、法定代理人或有同意權之人皆無法閱讀時，應由見證人在場參與所有有關受試者同意之討論。並確定受試者、法定代理人或有同意權之人之同意完全出於其自由意願後，應於受試者同意書簽名並載明日期。試驗相關人員不得為見證人。

中國醫藥大學暨附設醫院
受試者同意書
（成年免疫第三劑組）

流程表:

批次分析與免疫分析組

訪視	1[m]	2[m]		3		4		5/提早退出試驗	6[t]		7		長期性追蹤
檢測項目	篩選	第一次接種		第二次接種		追蹤[g]		個別解盲	第三次接種[h]		追蹤[h]		第 12 個月追蹤[i]
天數	-28~-1	1	8, 15, 22	29 ±3 天	36, 43	57 ±3 天	64, 71, 78,85	197 ±15 天	197~242	第六次訪視後 7 天	第六次訪視後 14 天 ±3 天	253, 309	365 ±45 天
獲得受試者同意書	X								X[h]				
納入/排除條件	X	X											
隨機分派		X											
接種評估				X					X[h]				
基本資料	X												
醫療病史	X	X											
身體檢查[a]	X	X		X		X		X	X[h]		X[h]		
生命徵象	X	X		X		X		X	X[h]		X[h]		X
心電圖	X												
**實驗室檢測 (安全性)**													
血液常規檢測[j]	X					X			X[h]		X[h]		
血液生化學檢測[j]	X					X			X[h]		X[h]		
免疫檢測[j]	X					X			X[h]		X[h]		
懷孕檢測[b]		X		X					X[h]				

訪視	1[m]	2[m]		3		4		5/提早退出試驗	6[t]		7		長期性追蹤
檢測項目	篩選	第一次接種		第二次接種		追蹤[g]		個別解盲	第三次接種[h]		追蹤[h]		第12個月追蹤[i]
天數	-28~-1	1	8, 15, 22	29 ±3天	36, 43	57 ±3天	64, 71, 78,85	197 ±15天	197~242	第六次訪視後7天	第六次訪視後14天 ±3天	253, 309	365 ±45天
尿液常規檢測[b]	X	X[q]		X[q]		X[q]		X[q]	X[h, q]		X[h, q]		
**實驗室檢測(免疫原性)**													
免疫原性[c]		X		X[n]		X		X					X
T細胞反應(可選擇[o])		X				X							
**實驗室檢測(探索性試驗)**													
T細胞功能性檢測(可選擇[u])									X[h]		X[h]		
備血	X					X							
免疫原性[c]									X[h]		X[h]		
疫苗接種		X		X					X[h]				
指導使用電子日誌卡		X		X					X[h]				
電話安全性追蹤[d]			X		X		X			X[s,h]		X	
不良事件/特殊不良事件[p]/醫		X[k]	X[k]	X[k]	X[k]	X[k]	X[k]	X[l]	X[l, h]	X[l, h]	X[l, h]	X[l]	X[l]

訪視	1[m]	2[m]		3		4		5/提早退出試驗	6[t]		7		長期性追蹤
檢測項目	篩選	第一次接種		第二次接種		追蹤[g]		個別解盲	第三次接種[h]		追蹤[h]		第12個月追蹤[i]
天數	-28~-1	1	8, 15, 22	29 ±3天	36, 43	57 ±3天	64, 71, 78,85	197 ±15天	197~242	第六次訪視後7天	第六次訪視後14天 ±3天	253, 309	365 ±45天
療需求不良事件/嚴重不良事件													
新型冠種病毒感染監測		X	X	X	X	X[e]	X[e]	X[e]	X[e, h]	X[e, h]	X[e, h]	X[e]	X[e]
併用藥物		X		X		X		X[f]	X[f, h]		X[f, h]		

a: 身高與體重僅在第一次訪視測量。
b:於第1, 29天使用尿液懷孕檢測。若尿液檢測為陽性，應以血清懷孕檢測再次確認。以血清懷孕檢測替代尿液檢測將不視為試驗偏差。蛋白尿將透過尿液常規檢測確認，做為基礎值。
c: 針對 Anti-S1-RBD 免疫球蛋白G濃度和新型冠狀病毒中和抗體效價，以及抑制S1-RBD: ACE2的抗體效價。
d: 所有受試者將進行電話安全追蹤以監測非預期性不良事件，包括特殊不良事件，和監測新型冠狀病毒感染的症狀。
e: 受試者將在每周(為每7天)由手機接獲一條提示以規律監測新型冠狀病毒感染症狀或病徵。記錄疑似新冠病毒感染所使用的藥物至新冠病毒感染頁面。
f: 只記錄醫療需求不良事件和嚴重不良事件之併用藥物。
g: 第二次接種疫苗後第28天
h: 僅針對同意並進行施打第三劑試驗疫苗的疫苗組受試者
i: 第二次接種疫苗後第十二個月(第365天)
j: 安全性實驗室數值包括全血液計數(血紅素、血比容、紅血球計數)、白血球計數、血小板計數、肌酸酐、丙胺酸轉胺酶、天門冬胺酸轉胺酶、總膽紅素、直接膽紅素、高靈敏度C反應性蛋白、抗核抗體
k: 主動收集時期
l: 被動監測時期
m: 第一次訪視與第二次訪視可為同一次訪視。
n: 僅測量 Anti-S1-RBD IgG 濃度
o: 將在選定的試驗地點納入至少100位>18-<65歲的受試者。
p: 包括接種最後一劑疫苗後12個月，可能的免疫媒介醫療狀況(PIMMC)或任何定義為可能的特殊不良事件。新冠病毒感染的併發症也將視為疾病增強事件，將被記錄與通報為特殊不良事件。
q: 若受試者有≥第三級以上的高血壓，受試者將確認是否有蛋白尿存在或惡化。
r: 備血將被冷凍存放做為UBI新型冠狀病毒酵素結合免疫吸附分析法，新型冠狀病毒確認酵素結合免疫吸附分析法，以及未來免疫學研究之用。
s: 若符合資格並接種第三劑疫苗的受試者將進行兩週的日誌卡追蹤紀錄，並將於接種後第7天進行電話安全性追蹤。
t: 第五次訪視和第六次訪視可為同一天。

中國醫藥大學暨附設醫院

受試者同意書

（成年免疫第三劑組）

u: 在選定之試驗地點中，將邀請大約 30 位年齡 >18-<65 歲的受試者和大約 30 位年齡 ≥65 歲的受試者。在適用的情況下，優先邀請曾進行過第 57 天 T 細胞功能評估的受試者。

中國醫藥大學暨附設醫院
受試者同意書
（成年安全確認第三劑組）

2021.10.25

您被邀請參與此研究。此同意書主要是提供您本研究之相關資訊，以便您決定是否參加本研究。計畫主持人或其指定之研究人員會為您說明研究內容並回答您的疑問。您可以提出任何和此研究有關的問題，在您的問題尚未獲得滿意的答覆之前，請不要簽署此同意書。如果您願意參與本研究，此文件將視為您的同意紀錄。即使在您同意後，您可以隨時退出本研究不需任何理由。

計畫名稱

中文：一個評估 UB-612 疫苗對於新型冠狀病毒於青少年、成年和老年健康受試者的免疫原性、安全性與耐受性的第二期、安慰劑控制、隨機分派、觀察者盲性臨床試驗

英文：A Phase II, Placebo-controlled, Randomized, Observer-blind Study to Evaluate the Immunogenicity, Safety and Tolerability of UB-612 Vaccine against COVID-19 in Adolescent, Younger and Elderly Adult Volunteers

執行單位：中國醫藥大學附設醫院感染科、家庭醫學科	委託單位/藥廠：聯亞生技開發股份有限公司<br>研究經費來源：聯亞生技開發股份有限公司<br>受託研究機構：晉加股份有限公司
計畫主持人：黃高彬	職稱：主治醫師
協同主持人：林文元	職稱：主治醫師
協同主持人：林伯昌	職稱：主治醫師
緊急聯絡人：黃高彬	電話：0975-681-950

受試者姓名：　病歷號碼：

性別：　出生日期：

身分證字號：　聯絡電話：

通訊地址：

法定代理人或有同意權人之姓名：　與受試者關係：

性別：　出生日期：

身分證字號：　聯絡電話：

通訊地址：

(一)試驗簡介：

1. 本品/技術資料：

中國醫藥大學暨附設醫院
受試者同意書
（成年安全確認第三劑組）

新型冠狀病毒(SARS-CoV-2)於2019年12月起造成中國湖北省武漢市發現多起病毒性肺炎群聚，隨後於2020年1月底台灣出現第一起境外移入確診個案。此疾病在全球擴散，世界衛生組織宣布將此疫情為「國際關注公共衛生緊急事件」。截至2020年底，全球僅有數間疫苗公司，如美國輝瑞藥廠等，取得緊急使用授權上市。

UB-612疫苗為聯亞生技開發股份有限公司所開發新型冠狀病毒預防性疫苗，疫苗含病毒棘狀融合蛋白和胜肽片段，可產生高親和力抗體與新型冠狀病毒結合，並誘發細胞免疫反應，進而達到預防新型冠狀病毒的感染。

UB-612第一期延伸性試驗顯示，接種第三劑UB-612疫苗可以誘發極高的中和抗體，在目前變種病毒的威脅之下，施打第三劑加強免疫反應，已是許多國家的選擇。

2. 本品上市狀況：

本品仍應用於人體試驗，尚未在我國上市。

3. 本試驗使用的UB-612疫苗對新型冠狀病毒的預防效果仍未確認。

4. 您簽署這份受試者同意書是由於您願意接種第三劑UB-612疫苗。

(二)試驗目的：

主要試驗目的

- 評估UB-612疫苗誘發的新型冠狀病毒中和抗體效價。
- 評估接種UB-612疫苗後的安全性和耐受性。

次要試驗目的

- 評估在試驗期間對於新型冠狀病毒的免疫反應。
- 評估三批獨立批次疫苗的批次免疫一致性。

探索性試驗目的

- 評估UB-612疫苗誘發的T細胞功能。
- 評估UB-612疫苗在年輕受試者的安全性和免疫原性。
- 評估UB-612疫苗的療效。
- 描述UB-612疫苗於確診和/或嚴重感染新型冠狀病毒案例之血液學反應。
- 評估針對SARS-CoV-2抗原的抗體反應。

(三)試驗之主要納入與排除條件：

中國醫藥大學暨附設醫院執行本研究計畫的醫師或相關研究人員將會與您討論有關參加本研究的必要條件。請您配合必須誠實告知我們您過去的健康情形，若您有不符合參加本研究的情況，將不能參加本研究計畫。

1. 參加本研究計畫的主要條件:

☐(1) 您為納入試驗時20~85歲之間健康男性或未懷孕的女性受試者。

☐(2) 您為具生育能力的女性與男性應於首次接種疫苗至最後一次疫苗後3個月同意進行有效的避孕方式。可接受的有效避孕方式包括:

☐a. 男性或女性以手術方法絕育、植入式避孕、或子宮避孕器。

☐b.注射避孕、避孕藥、避孕貼片、避孕環加上一種屏障避孕法*。

☐c.合併使用兩種屏障避孕法*。

*有效的屏障避孕法為避孕隔膜、男性或女性保險套、避孕海綿或殺精劑(含可殺精化學物質的藥膏或凝膠)。

☐(3) 您能理解受試者同意書內容的說明與可能的風險，提供簽名的受試者同意書。

☐(4) 您能夠理解與遵從本試驗程序與能夠參與每次訪視。

☐(5) 您的耳溫≤ 38.0°C。

☐(6) 您依據醫療病史、身體檢查和試驗主持人的臨床判斷為健康受試者**可符合納入試驗資格。經試驗主持人判斷，即便您的病史穩定或且控制良好，但伴隨病情惡化而有提高嚴重新型冠狀病毒感染的風險。

**健康受試者有先前存在的穩定疾病者可以納入試驗，定義為該疾病在納入試驗前12週內沒有惡化至需要治療或住院的顯著變化和在納入試驗6個月內沒有惡化至需要治療或住院的顯著變化。

2. 若您有下列任一情況，您將無法參加本研究計畫:

☐(1) 您有接種疫苗後需要醫療介入的過敏性休克、蕁麻疹或其他顯著不良反應的病史。

☐(2) 您在篩選時或接種每劑疫苗前已懷孕女性或懷孕檢測為陽性的女性。

☐(3) 您為正在哺乳的女性，或計畫從接種第一劑疫苗至最後一劑疫苗後60天哺乳的女性。

☐(4) 您在接種第一劑疫苗前3天內，經試驗主持人判斷，患有任何急性疾病。

☐(5) 您在接種第一劑疫苗前1個月內有重大手術。

☐(6) 您是已知為人類免疫缺乏病毒抗體陽性。

☐(7) 您是已知為活動性B型肝炎或C型肝炎。活動性肝炎定義為肝臟轉胺酶(天門冬胺酸轉胺酶和/或丙胺酸轉胺酶)大於3倍正常值上限和/或總膽紅素大於3倍正常值上限。

☐(8) 您是已知曾暴露於新型冠狀病毒，或曾接受預防新型冠狀病毒、中東呼吸症候群冠狀病毒、嚴重急性呼吸道症候群的試驗或已上市產品。

☐(9) 您有格林-巴利症候群的病史。

☐(10) 您在簽署受試者同意書前12周內參與其他的臨床試驗。

☐(11) 您為免疫缺乏/失調疾病，無論是否由基因缺陷、免疫缺乏症或免疫抑制療法所造成。

☐(12) 您計畫或正在進行抗癌症治療。

☐(13) 您患有血小板異常或其他凝血異常可能造成注射之禁忌症。

☐(14) 您在接種第一劑疫苗前6個月長期接受(≥14天連續使用)免疫抑制劑、皮質類固醇(相當於一天使用≥ 20 mg強的松(prednisone))或細胞毒性治療。

☐(15) 您在接種第一劑疫苗前4個月接受免疫球蛋白和/或任何血液製劑的治療。

☐(16) 您在接種試驗疫苗前14天接種任何季流感疫苗或新型流感疫苗，或前28天接種其他疫苗。

☐(17) 您預期在接種試驗疫苗後14天接種任何季流感疫苗或新型流感疫苗，或後28天接種其他疫苗。

☐(18) 您使用短期(<14天使用)全身性類固醇。應於中斷使用全身性類固醇至少28天後才可使用試驗疫苗。吸入/噴霧性、關節注射、囊內或局部(皮膚或眼用)類固醇可允許使用。

☐(19) 您在篩選期前3個月失血或捐血超過500毫升，或預計在試驗期間內捐血或輸血。

☐(20) 經試驗主持人判斷，您有任何醫療疾病或狀況，可能會影響試驗結果或參與試驗可能會對受試者引發額外風險。

☐(21) 您是直接參與本試驗執行的試驗主持人所屬機構的試驗團隊、試驗委託者或受託

研究機構(CRO)的員工。

(四)試驗方法及相關檢驗：

這是一個第二期、觀察者盲性、多中心、隨機分派、安慰劑控制試驗，以評估青少年，成年和老年受試者使用兩劑UB-612疫苗的免疫原性，耐受度和安全性。有一部份的受試者使用UB-612疫苗，而另外一部份的受試者則使用「安慰劑」。所謂「安慰劑」是不含有效成份的疫苗。至於誰使用試驗用藥或誰使用「安慰劑」，則像丟銅板或擲骰子一樣由機率決定，不管是您或是研究醫師都不知道您使用了那一種藥，只有分發跟施打疫苗的試驗人員才知道您使用哪一種疫苗，這叫做觀察者盲性。

總計約有3850位合格成年受試者組成核心組用於申請緊急使用授權，另外大約有385位青少年受試者組成補充組申請額外適應症。所有受試者將以6:1的比例，隨機分派至兩劑100微克劑量組別和安慰劑組，包括462位大於18歲至小於65歲可評估的受試者進入批次分析組。對於免疫分析，至少包括350位可評估的成年受試者(年齡大於18歲至小於65歲)和154位可評估的老年受試者(年齡≥65歲)進行描述性分析。免疫原性的受試者將會先納入試驗。所有的受試者將會納入安全性分析，其中至少770位隨機分派的受試者為≥65歲的分層。青少年組將在核心組招募完畢後，再開始納入試驗。約有385位青少年受試者將以6:1的比例隨機分派，其中包括154位可評估的青少年受試者將收集免疫原性數據，並和成年及老年受試者數據進行比較。

您若參與這個試驗，則為進行安全性確認的安全確認組。

試驗總共有8個訪視。若您參與本試驗，則至少包括第一次訪視(篩選訪視)、第二次訪視（第1天，基礎值，隨機分派，第一次接種疫苗)、第三次訪視(第29天，第二次接種疫苗)、第四次訪視(第57天)、第五次訪視(第197天)。

在第五次訪視時，預計將進行個別解盲。若個別解盲後，得知您為施打疫苗的受試者，且你有意願且符合資格接種第三劑疫苗，將進入第六次訪視(第197~242天，第三次接種疫苗)，第七次訪視(接種疫苗後第14天)，及第八次訪視(第365天)。

整個試驗期間，預期您將參與試驗最長達13個月。

<u>注意事項</u>

1. 如果您同意參加本試驗，研究人員會請您簽署本份受試者同意書，並確認您符合參加本試驗的條件。
2. 從您參與試驗的當天開始，每次訪視都將有合格的試驗人員執行試驗流程與聯繫。

3. 若您有任何符合新型冠狀病毒感染的定義(您曾於過去 7 天內有出國，或是接觸疑似或確認武漢肺炎之病人，而有下列症狀: 發燒、開始咳嗽或惡化、開始呼吸急促或惡化、寒顫、開始肌肉疼痛或惡化、喉嚨痛、腹瀉、嘔吐、開始味覺/嗅覺異常)，請依照中央疫情指揮中心規定進行自主健康管理或至指定院所進行篩檢。
4. 若您在試驗期間感染新型冠狀病毒，將依法通報主管機關。

您是否同意? ☐是 ☐否

簽名：__________日期：__________

5. 於試驗期間，您不論任何理由提前退出試驗，試驗研究人員都將安排您完成最後一次的訪視之所有試驗項目。您有權利拒絕此項安排，您的決定不會引起任何影響日後醫師對您的醫療照護。

試驗步驟

第一次訪視(第-28~-1 天) -篩選訪視

在試驗醫師或試驗研究人員為您提供足夠的試驗資訊，並確保您有充分的時間考慮以及詢問任何問題後，您願意讓您參與本試驗，並由您簽署本受試者同意書。在確認您已完成受試者同意書簽署並且您也保有一份副本後，試驗醫師或試驗研究人員將會進行以下試驗程序：

(1) 記錄您簽署受試者同意書的日期
(2) 為您指定一組受試者篩選編號
(3) 確認您是否符合本試驗的納入排除條件
(4) 收集您的個人基本資料（例如生日、年齡及性別）
(5) 記錄您的醫療/用藥病史
(6) 進行身體檢查，包括身高體重
(7) 確認生命徵象
(8) 進行心電圖檢測
(9) 收集尿液檢體進行尿液檢測
(10) 收集血液檢體(共 15.5 毫升)，進行下列檢測:

- 常規血液檢測
- 血液生化學檢測
- 免疫學檢測(抗核抗體)

- 備血(作為測量新型冠狀病毒血清抗體和相關研究之用)

第二次訪視(第 1 天)-基礎值，確認符合試驗條件，第一次接種疫苗

(1) 再度確認您是否符合本試驗的納入排除條件
(2) 隨機分派，給予您一組隨機分派號碼
(3) 記錄您的醫療/用藥病史
(4) 進行身體檢查
(5) 確認生命徵象
(6) 進行尿液懷孕檢測（具有生育能力女性）
(7) 若您有產生第三級以上的高血壓，您將被收集尿液檢體以檢測是否有蛋白尿存在或惡化
(8) 進行第一次疫苗接種(注射部位為非慣用手，採用肌肉注射方式)。接種疫苗後，受試者應留在試驗地點至少 30 分鐘，監測生命徵象和急性過敏症狀。
(9) 將詳細地指導您如何填寫電子日誌卡(包括注射後 7 天內預期性不良事件,14 天內的皮膚過敏反應日誌卡)
(10) 收集併用藥物/治療

第一次電話安全性追蹤(第 8, 15, 22 天)

將與您電話聯繫，以追蹤未預期不良事件和新型冠狀病毒感染症狀。

第三次訪視(第 29± 3 天)-第二次接種疫苗

(1) 進行第二次接種評估(有可能會延遲接種時間)
(2) 進行身體檢查
(3) 確認生命徵象
(4) 進行尿液懷孕檢測（具生育能力的女性）
(5) 若您有產生第三級以上的高血壓，您將被收集尿液檢體以檢測是否有蛋白尿存在或惡化。
(6) 進行第二次疫苗接種。
(7) 接種疫苗後,受試者應留在試驗地點至少 30 分鐘,監測生命徵象和急性過敏症狀。
(8) 將詳細地指導您如何填寫電子日誌卡(包括注射後 7 天內預期性不良事件，14 天內的皮膚過敏反應日誌卡)
(9) 收集併用藥物/治療
(10) 記錄上一次訪視至此次訪視之間的不良事件、嚴重不良事件型或新型冠狀病毒感染症狀

第二次電話安全性追蹤(第 36, 43 天)

將與您電話聯繫，以追蹤未預期不良事件和新型冠狀病毒感染症狀。

第四次訪視(第 57±3 天) –追蹤訪視

(1) 進行身體檢查
(2) 確認生命徵象
(3) 若您有產生第三級以上的高血壓，您將被收集尿液檢體以檢測是否有蛋白尿存在或惡化。
(4) 收集血液檢體(共 15.5 毫升) ，進行下列檢測:
- 常規血液檢測
- 血液生化學檢測
- 免疫學檢測(抗核抗體)
- 備血(作為測量新型冠狀病毒血清抗體和相關研究之用)

(5) 收集併用藥物/治療
(6) 記錄上一次訪視至此次訪視之間的不良事件、嚴重不良事件或新型冠狀病毒感染症狀
(7) 在此次訪視後，您將每周接獲訊息提醒，以定期監測新型冠狀病毒感染症狀至第 197 天。

第三次電話安全性追蹤(第 64, 71, 78, 85 天)

將與您電話聯繫，以追蹤安全性及新型冠狀病毒感染症狀。

第五次訪視(第 197±15 天) –個別解盲

(1) 進行身體檢查
(2) 確認生命徵象
(3) 若您有產生第三級以上的高血壓，您將被收集尿液檢體以檢測是否有蛋白尿存在或惡化。
(4) 將替您個別解盲，告知您接種到疫苗或安慰劑。
(5) 收集併用藥物/治療
(6) 記錄上一次訪視至此次訪視之間的不良事件、嚴重不良事件或新型冠狀病毒感染症狀

第六次訪視（第 197~242 天，第三次接種疫苗）

(1) 記錄您簽署受試者同意書的日期
(2) 確認您是否符合接種第三劑疫苗的資格，包括第三劑接種疫苗的禁忌症，或有延遲第三劑接種時間的條件
(3) 進行身體檢查

(4) 確認生命徵象
(5) 若您有產生第三級以上的高血壓，您將被收集尿液檢體以檢測是否有蛋白尿存在或惡化
(6) 收集血液檢體(**若不進行 T 細胞檢測，**共 20.5 毫升)，進行下列檢測:

- 常規血液檢測
- 血液生化學檢測
- 免疫學檢測(抗核抗體)
- 探索性試驗免疫反應檢測
- **若您願意，將進行 T 細胞檢測(需額外抽血 56 毫升)**

您是否同意? □是 □否

簽名：___________日期：_____________

(7) 進行尿液懷孕檢測（具有生育能力女性）
(8) 進行第三次疫苗接種。
(9) 接種疫苗後，受試者應留在試驗地點至少 30 分鐘，監測生命徵象和急性過敏症狀。將詳細地指導您如何填寫電子日誌卡(包括注射後 7 天內預期性不良事件，14 天內的皮膚過敏反應日誌卡)
(10) 收集併用藥物/治療
(11) 進行新型冠狀病毒監測
(12) 記錄上一次訪視至此次訪視之間的不良事件、嚴重不良事件型或新型冠狀病毒感染症狀

<u>第六次訪視後第 7 天電話安全性追蹤</u>

將與您電話聯繫，以追蹤未預期不良事件和新型冠狀病毒感染症狀。此外，還將監測皮膚過敏反應，或其他非預期的過敏反應。若您有發生任何第三級以上的過敏事件，試驗人員可能將安排您額外的回診。

<u>第七次訪視 (第六次訪視後第 14± 3 天)</u>

(1) 進行身體檢查
(2) 確認生命徵象
(3) 若您有產生第三級以上的高血壓，您將被收集尿液檢體以檢測是否有蛋白尿存在或惡化。
(4) 收集血液檢體(**若不進行 T 細胞檢測，**共 20.5 毫升)，進行下列檢測:

- 常規血液檢測

- 血液生化學檢測
- 免疫學檢測(抗核抗體)
- 探索性試驗免疫反應檢測
- **若您願意，將進行 T 細胞檢測(需額外抽血 56 毫升)**

(5) 收集併用藥物/治療

(6) 進行新型冠狀病毒監測

(7) 記錄上一次訪視至此次訪視之間的不良事件、嚴重不良事件型或新型冠狀病毒感染症狀

追蹤期電話安全性追蹤(第 253, 309 天)

第七次訪視後將每兩個月與您電話聯繫，以追蹤安全性及新型冠狀病毒感染症狀。

第八次訪視**(第 365±45 天) –第 12 個月追蹤**

(1) 確認生命徵象

(2) 收集血液檢體(共 10 毫升)，進行下列檢測:

- 探索性試驗免疫反應檢測

(3) 記錄上一次訪視至此次訪視之間的不良事件，包括特殊不良事件、醫療不良事件、嚴重不良事件或新型冠狀病毒感染症狀

受試者之檢體(含其衍生物)之保存、使用與再利用：

1. 檢體及剩餘檢體之保存與使用

(1) 檢體(含其衍生物)之保存與使用

為研究所需，我們所蒐集您的檢體，將依本研究計畫使用，檢體將保存於聯亞生技開發股份有限公司(試驗委託者)，直至 20 年保存期限屆滿，我們將依法銷毀。為了保護您的個人隱私，我們將以一個試驗編號來代替您的名字及相關個人資料，以確認您的檢體及與相關資料受到完整保密。如果您對檢體的使用有疑慮，或您有任何想要銷毀檢體的需求，請立即與我們聯絡(聯絡人：黃高彬醫師電話：0975-681-950)，我們即會將您的檢體銷毀。您也可以聯繫中國醫藥大學暨附設醫院研究倫理委員會(電話：04-22052121 轉 1925、1926)，以協助您解決檢體在研究使用上的任何爭議。

(2) 剩餘檢體(含其衍生物)之再利用

您的生物檢體將會以專屬號碼進行編碼並在聯亞生技開發股份有限公司(試驗委託

者)的控管下儲存最長20年，以研究UB-612 疫苗反應者的生物標記，及改善治療方式。

所有新的研究計畫都要再經由中國醫藥大學暨附設醫院研究倫理委員會審議通過，倫理審查委員會若認定新的研究超出您同意的範圍，將要求我們重新得到您的同意。

是否同意剩餘檢體保留提供未來新型冠狀病毒感染研究之用，並授權中國醫藥大學暨附設醫院研究倫理委員會審議是否需要再取得您的同意(擇一)

☐ 不同意保存我的剩餘檢體，試驗結束後請銷毀

☐ 同意以非去連結之方式保存我的剩餘檢體，逾越原同意使用範圍時，需再次得到我的同意才可使用我的檢體進行新的研究

2. 檢體及剩餘檢體之部分類型(檢體類型可依計畫書內容自行增減)

(1) 一般生化、血液檢驗/病毒檢測檢體

在試驗期間，會將您的檢體送往聯亞生技開發股份有限公司(試驗委託者)委託的中央實驗室中國醫藥大學暨附設醫院，此機構地址為台中市北區育德路2號，和大安聯合醫事檢驗所，此機構地址為台北市大安區復興南路二段151巷33號，中央實驗室會在分析後立即將分析結果提供給試驗中心，若有剩餘的檢體，將儲存直到至少完成臨床試驗報告為止，最長將保存20年。

(2) 抗體/細胞免疫試驗

在試驗期間，會將您的檢體送往聯亞生技開發股份有限公司(試驗委託者)分析實驗室。完成試驗後，若有剩餘檢體，將儲存直到至少完成臨床試驗報告為止，最長將保存20年。

(3) 中和試驗(neutralization test, NT)

在試驗期間，會將您的檢體送往聯亞生技開發股份有限公司(試驗委託者)委託的中央實驗室中央研究院進行處置、處理與進一步分析。此機構地址為台北市南港區研究院路二段 128 號。完成試驗後，若有剩餘檢體，將儲存直到至少完成臨床試驗報告為止，最長將保存 20 年。

(4) 遺傳學檢體

在試驗期間，若發生嚴重不良反應或特定不良反應，您的檢體將用於 HLA 分型檢驗，會將您的檢體送往聯亞生技開發股份有限公司(試驗委託者)委託的中央實驗室有勁

基因股份有限公司分析，此機構地址為新北市樹林區復興路 376-5 號，中央實驗室不會將分析結果提供給試驗中心，若有剩餘的檢體，將會儲存直到檢驗結果複驗完畢即銷毀，不會長期儲存。

(5) 探索性試驗檢體

在試驗期間，會將您的檢體送往聯亞生技開發股份有限公司(試驗委託者)委託的相關實驗室(表一)進行處理或進一步分析。完成試驗後，若有剩餘檢體，將儲存直到至少完成臨床試驗報告為止，最長將保存 20 年。

表一、實驗室名稱與機構地址

實驗室名稱	機構地址
聯亞生技開發(股)公司	新竹縣竹北市生醫路二段 6-1 號 5 樓
Viroclinics	Rotterdam Science Tower, Marconistraat 16, 3029 AK Rotterdam, The Netherlands(荷蘭)
DASA	Jonas Cruz de Araujo, Diagnostics da America S/A, Surubiju Avenue, 1890, Barueri, SP, Brazil(巴西), 06455-040
PHE Porton Down	Salisbury Wiltshire SP4 0JG, England(英國)
UTMB	University of Texas Medical Branch 301 University Boulevard Keiller Building, Room 2.150 Galveston, Texas, USA(美國
Virology	University of São Paulo, Brazil Rua Dr EnnEn de Carvalho Aguiar 470, CEP 05403-000 (巴西)
VRDL	California Department of Public Health, 850 Marina Bay Parkway, Richmond, CA 94804, USA(美國)
NEXELIS	525 Boul. Cartier Ouest Laval, Qulbec, Canada, H7V 3S8(加拿大)
Vaccinology and Immunology Infection, Immunity & Inflammation Dept UCL GOS Institute of Childe Health	UCL Great Ormond Street Institute of Child Health 30 Guilford Street London WC1N 1EH, England(英國)
VisMederi	VisMederi Srl, Strada del Petriccio e Belriguardo, 35, 53100 Siena, Italy(義大利)

(五)可能產生之副作用、發生率及處理方法：

1. 與試驗藥物相關的風險（本試驗疫苗的副作用）：

冠狀病毒疫苗的開發

過去針對與SARS-CoV-2病毒相同屬於人類冠狀病毒的SARS-CoV(嚴重急性呼吸綜合症

冠狀病毒(SARS冠狀病毒))的疫苗研究發現，接種過SARS-CoV疫苗的小鼠在暴露到SARS-CoV後會發生過度免疫反應而產生病變，因此不得不停止這種疫苗的開發。所以，成功的人類冠狀病毒疫苗不只要產生可以抑制病毒的免疫反應，更要避免過度免疫產生的副作用。

疫苗相關的風險: 第一期臨床試驗

接種疫苗可能會出現注射部位的不良反應(例如疼痛、硬化腫脹、皮疹發紅、過敏反應、蜂窩性組織炎)，或全身性不良反應(例如發燒、腹瀉、疲倦、噁心/嘔吐、厭食、咽喉痛、頭痛、咳嗽、關節痛、非注射部位疼痛、非注射部位搔癢、皮膚和黏膜異常、急性過敏反應、昏厥、急性支氣管痙攣、呼吸困難)。

第一期臨床試驗已經有60位受試者接種兩劑疫苗(含10微克、30微克、100微克融合蛋白)，安全實驗室數值並沒有顯示有任何的臨床顯著不正常數值，也沒有發生任何第三級以上與疫苗相關的預期性不良事件。大部分的預期性不良事件都是輕微的，症狀在大約於2天之內都會緩解。試驗中也沒有任何的嚴重不良事件或特殊不良事件被通報。

疾病增強(disease enhancement) 的風險

SARS-CoV-2候選疫苗也可能會有引發疾病增強(disease enhancement) 的風險，包括抗體依賴性增強(antibody-dependent enhancement)或疫苗相關聯的增強的呼吸道疾病(vaccine-associated enhanced respiratory disease)。在先前研發SARS疫苗時，在數個SARS-CoV動物攻毒試驗(包括鼠類、雪貂、猴類)當中，有發現疾病增強的現象。疾病增強反應的免疫病理現象包括TH2偏向及嗜酸性白血球的肺部浸潤。但是目前已發表的新型冠狀病毒肺炎疫苗研究，仍尚未發現類似的疾病增強現象。

本試驗疫苗於數個藥理試驗呈現不一致的TH1/TH2 (輔助型T細胞1/輔助型T細胞2)免疫反應偏向，試驗結果並未一致偏向TH2，而由小鼠之SARS-CoV-2動物攻毒試驗結果顯示，本試驗疫苗誘發疾病增強之風險不高。依據文獻指出，組成複雜或容易引起非中和抗體之抗原，如不活化病毒或整片段之蛋白(包含S蛋白與N蛋白)，與易引起偏向Th2免疫反應之佐劑成分，如鋁製佐劑，皆較有可能引起疾病增強。本試驗疫苗的主要抗原為S蛋白上之RBD 區域，已有多篇文獻指出，針對S-RBD設計之SARS與MERS疫苗從未於試驗動物模型上引發疾病增強現象。本試驗疫苗雖使用易引起偏向Th2免疫反應之佐劑，但由動物實驗證實，也同時引起偏向Th1之反應，因此發生疾病增強應屬低風險。且已於多種動物模型中證實，能誘發高效價之中和抗體，於細胞培養中亦能有效抑制新冠病毒感染。

建議您在有效疫苗上市前或本試驗疫苗的產品資訊有進一步更新前，盡量避免暴露於可能感染病毒的環境。研究團隊將會在試驗中執行相關安全性監測。若有任何關於本試驗

疫苗與疾病增強風險相關之任何最新資訊，將即時更新並提供給您。

疫苗佐劑相關的風險

本試驗疫苗所使用的佐劑含Adju-Phos®，是屬於一種磷酸鋁類的佐劑。磷酸鋁類佐劑已經使用超過半個世紀，具有相當的安全性。由於此類佐劑可誘導免疫反應，因此可能會造成局部發炎反應，例如在注射部位產生輕微而短暫的疼痛、發紅以及腫脹。

2. 與試驗/研究過程相關的風險：

抽血

本試驗需要抽血檢驗。抽血可能引起一些不適和瘀血。整個試驗期間13個月，共需抽血 82毫升。**若您願意抽血檢驗T細胞檢測，則會於特定訪視每次額外抽血56毫升。**若您罹患新型冠狀病毒感染，可能將於每次額外訪視抽血30毫升。

在接種疫苗過程中，可能會出現一些尚未在已完成試驗中發現的副作用。一般而言，接種某一新疫苗總是會有一定的風險，但是計畫主持人會採取一切措施預防風險的發生。**計畫主持人鼓勵您報告您遇到的任何不適。**

(六)其他替代療法及說明：

您不是非參加不可，若不參加研究，由於目前尚未有疫苗可用來預防新型冠狀病毒感染，因此預防措施與其他呼吸道感染相同，包括：勤洗手、減少觸摸眼口鼻、注意咳嗽禮節、妥善處理口鼻分泌物等，避免出入公共場所，並不要接觸野生動物。

如果您對於本試驗疫苗有任何的疑問，您可以提出來向您的試驗醫師討論。

(七)試驗預期效益：

依據臨床前試驗結果，預期本試驗疫苗對您可能可以產生抗體，預防新型冠狀病毒感染，但因每個人體質不同也有可能不會產生療效，故參加本試驗可能不會有直接的好處。

但是您參加本試驗，可協助我們獲得更多資訊，以瞭解UB-612疫苗的安全性與免疫力。

(八)試驗進行中受試者之禁忌、限制與應配合之事項：

禁止使用的藥物

以下藥物請勿在試驗期間使用：

- 直到試驗第57天禁止使用免疫抑制劑、或細胞毒性治療

- 到試驗第57天禁止使用免疫球蛋白和/或任何血液製劑
- 整個試驗期間禁止使用試驗產品(包括藥物或疫苗)
- 到試驗第57天禁止使用全身性皮質類固醇(相當於一天使用≥ 20 mg強的松(prednisone))
- 接種試驗疫苗後14天禁止接種任何季流感疫苗或新型流感疫苗，或後28天禁止接種其他非試驗疫苗。整個試驗期間禁止使用任何已上市的新型冠狀病毒疫苗產品。

允許使用的藥物

若您的藥物或治療必須常規使用，經試驗醫師判斷不會影響本試驗疫苗的安全性，則可以正常使用。您有任何關於在試驗期間可允許使用何種藥物或治療的問題，請詢問您的試驗醫師。

懷孕或母乳哺乳的風險

目前未知本試驗疫苗對於未出生胎兒的影響，因此：

- 您為具生育能力的女性受試者 (除非手術絕育或停經)，或您為男性受試者應於接種疫苗至最後一次疫苗後 3 個月同意進行有效的避孕方式，同意進行有效的避孕方式(例如子宮內節育器、荷爾蒙療法或避孕套)。
- 若您為具生育能力的女性，將請您進行懷孕檢測，結果必須為陰性，方可參與試驗。
- 若您為懷孕的女性，將被告知不可參與本試驗。
- 若您在試驗期間懷孕，請盡速通知試驗人員，並且停止施打本疫苗。
- 基於安全性考量，若您為女性受試者而在試驗期間懷孕，或您為男性受試者而您的性伴侶在試驗期間懷孕(將請您的懷孕性伴侶需簽署另外一份同意書)，您與您的胎兒將會被追蹤監測至分娩，除非另有醫學指示。

您應向您的配偶或性伴侶告知您有參與此試驗與相關風險:

簽名：__________日期：__________________

(九)機密性：

中國醫藥大學附設醫院將依法把任何可辨識您的身分之紀錄與您的個人隱私資料視為機密來處理，不會公開。研究人員將以一個研究代碼代表您的身分，此代碼不會顯示您的

中國醫藥大學暨附設醫院

受試者同意書

（成年安全確認第三劑組）

姓名、國民身分證統一編號、住址等可識別資料。如果發表試驗/研究結果，您的身分仍將保密。您亦瞭解若簽署同意書即同意您的原始醫療紀錄可直接受監測者、稽核者、研究倫理委員會及主管機關檢閱，以確保臨床試驗/研究過程與數據符合相關法律及法規要求，上述人員並承諾絕不違反您的身分之機密性。除了上述機構依法有權檢視外，我們會小心維護您的隱私。由於試驗藥物可能同時申請美國臨床試驗，依美國藥品管理規定，試驗結果將公佈於公開的臨床試驗資訊網站：Clinicaltrials.gov (美國)，但您的個人資料仍將保密，該網站只會有試驗之結果摘要，您可以在任何時候搜尋該網站。

在試驗/研究期間，依據計畫類型與您所授權的內容，我們將會蒐集與您有關的病歷資料、醫療紀錄、量表、問卷等資料與資訊，並以一個編號來代替您的名字及相關個人資料。前述資料若為紙本型式，將會與本同意書分開存放於研究機構之上鎖櫃中；若為電子方式儲存或建檔以供統計與分析之用，將會存放於設有密碼與適當防毒軟體之專屬電腦內。這些研究資料與資訊將會保存至藥品於我國上市後至少兩年，若試驗疫苗終止研發則保存至試驗正式停止後至少二年，至多將保存至疫苗上市後或試驗正式停止後二年。上述資料與資訊若傳輸至國外分析與統計，您仍會獲得與本國法規相符之保障，計畫主持人與相關團隊將盡力確保您的個人資料獲得妥善保護。

（十）損害補償與保險：

1. 如依本研究所訂臨床試驗計畫，因發生不良反應造成損害，由聯亞生技開發股份有限公司負補償責任。但本受試者同意書上所記載之可預期不良反應，不予補償。
2. 如依本研究所訂臨床試驗計畫，因而發生不良反應或損害，贊助廠商將依法負責損害賠償責任。本醫院願意提供專業醫療照顧及醫療諮詢。您不必負擔治療不良反應或損害之必要醫療費用。
3. 除前二項補償及醫療照顧外，本研究不提供其他形式之補償。若您不願意接受這樣的風險，請勿參加試驗。
4. 您不會因為簽署本同意書，而喪失在法律上的任何權利。
5. 本研究有投保責任保險。

（十一）受試者權利：

1. 試驗過程中，與您的健康或是疾病有關，可能影響您繼續接受臨床試驗意願的任何重大發現，都將即時提供給您。
2. 如果您在試驗過程中對試驗工作性質產生疑問，對身為患者之權利有意見或懷疑因參與研究而受害時，可與本院之研究倫理委員會聯絡請求諮詢，其電話號碼為：04-22052121轉1925、1926。
3. 為進行試驗工作，您必須接受黃高彬醫師的照顧。如果您現在或於試驗期間有任

何問題或狀況，請不必客氣，可與在中國醫藥大學附設醫院兒童感染科的黃高彬醫師聯絡（24小時聯繫電話：0975-681-950）。

4. 參加試驗研究計畫之補助：本計畫將在每次訪視提供交通費及營養費給您，新增的兩個診次(第六次返診、第七次返診)將各提供費用1000元；整個試驗預計給予您14000元。**若您願意參與T細胞檢測研究，將在該次訪視另外提供營養費500元給您。**

5. 本同意書一式2份，醫師已將同意書副本交給您，並已完整說明本研究之性質與目的。醫師已回答您有關藥品與研究的問題。

（十二）試驗之退出與中止：

您可自由決定是否參加本試驗；試驗過程中也可隨時撤銷同意，退出試驗，不需任何理由，且不會引起任何不愉快或影響其日後醫師對您的醫療照顧。

計畫主持人或贊助廠商亦可能於必要時中止該試驗之進行。

（十三）簽名：

1. 計畫主持人、或協同主持人已詳細解釋有關本研究計畫中上述研究方法的性質與目的，及可能產生的危險與利益。

   計畫主持人/協同主持人簽名：____________日期：________年____月____日

2. 受試者已詳細瞭解上述研究方法及其所可能產生的危險與利益，有關本試驗計畫的疑問，業經試驗主持人詳細予以解釋。本人同意接受為臨床試驗計畫的自願受試者。

   受試者簽名：____________________________日期：________年____月____日

   法定代理人簽名：________________________日期：________年____月____日

   * 受試者為無行為能力(未滿七歲之未成年人者或禁治產人)，由法定代理人為之；禁治產人，由監護人擔任其法定代理人。

   * 受試者為限制行為人者(滿七歲以上之未成年人)，應得法定代理人之同意。

   有同意權人簽名：________________________日期：________年____月____日

   * 受試者雖非無行為能力或限制行為能力者，但因意識混亂或有精神與智能障礙，而無法進行有效溝通和判斷時，由有同意權之人為之。前項有同意權人為配偶及直系親屬。

3. 見證人

   見證人簽名：______________________________日期：________年____月____日

   身分證字號：　　　　　　　　　　　　聯絡電話：

   通訊地址：

中國醫藥大學暨附設醫院
受試者同意書
（成年安全確認第三劑組）

* 受試者、法定代理人或有同意權之人皆無法閱讀時，應由見證人在場參與所有有關受試者同意之討論。並確定受試者、法定代理人或有同意權之人之同意完全出於其自由意願後，應於受試者同意書簽名並載明日期。試驗相關人員不得為見證人。

流程表:

安全確認組

訪視	1[l]	2[l]		3		4		5/提早退出試驗	6[t]		7		長期性追蹤
檢測項目	篩選	第一次接種		第二次接種		追蹤[f]		個別解盲	第三次接種[g]		追蹤[g]		第 12 個月追蹤[h]
天數	-28~-1	1	8, 15, 22	29 ±3 天	36, 43	57 ±3 天	64, 71, 78 ,85	197 ±15 天	197~242	第六次訪視後 7 天	第六次訪視後 14 天 ±3 天	253, 309	365 ±45 天
獲得受試者同意書	X								X[g]				
納入/排除條件	X	X											
隨機分派		X											
接種評估				X					X[g]				
基本資料	X												
醫療病史	X	X											
身體檢查[a]	X	X		X		X		X	X[g]		X[g]		X
生命徵象	X	X		X		X		X	X[g]		X[g]		
心電圖	X												
**實驗室檢測 (安全性)**													
血液常規檢測[i]	X					X			X[g]		X[g]		
血液生化學檢測[i]	X					X			X[g]		X[g]		
免疫檢測[i]	X					X			X[g]		X[g]		

訪視	1[l]	2[l]		3		4		5/提早退出試驗	6[t]		7		長期性追蹤
檢測項目	篩選	第一次接種		第二次接種		追蹤[f]		個別解盲	第三次接種[g]		追蹤[g]		第 12 個月追蹤[h]
天數	-28~-1	1	8, 15, 22	29 ±3 天	36, 43	57 ±3 天	64, 71, 78 ,85	197 ±15 天	197~242	第六次訪視後 7 天	第六次訪視後 14 天 ±3 天	253, 309	365 ±45 天
懷孕檢測[b]		X		X					X[g]				
尿液常規檢測[b]	X	X[n]		X[n]		X[n]		X[n]	X[g, n]		X[g, n]		
**實驗室檢測(探索性試驗)**									X		X		X
**T** 細胞功能性檢測(可選擇[l])									**X**[g]		**X**[g]		
備血	X					X							
免疫原性[c]									X[g]		X[g]		X[g]
疫苗接種		X		X					X[g]				
指導使用電子日誌卡		X		X					X[g]				
電話安全性追蹤[c]			X		X		X			X[p, g]		X	
不良事件/特殊不良事件[m]/醫療需求不良事件/嚴重不良事件		X[j]	X[j]	X[j]	X[j]	X[j]	X[j]	X[k]	X[k, g]	X[k, g]	X[k,g]	X[k]	X[k]

中國醫藥大學暨附設醫院

受試者同意書

（成年安全確認第三劑組）

訪視	1[l]	2[l]		3		4		5/提早退出試驗	6[t]		7		長期性追蹤
檢測項目	篩選	第一次接種		第二次接種		追蹤[f]		個別解盲	第三次接種[g]		追蹤[g]		第12個月追蹤[h]
天數	-28~-1	1	8, 15, 22	29 ±3 天	36, 43	57 ±3 天	64, 71, 78 ,85	197 ±15 天	197~242	第六次訪視後 7 天	第六次訪視後 14 天 ±3 天	253, 309	365 ±45 天
新型冠種病毒感染監測		X	X	X	X	X[d]	X[d]	X[d]	X[d, g]	X[d, g]	X[d, g]	X[d]	X[d]
併用藥物		X		X		X		X[e]	X[e, g]		X[e, g]		

a: 身高與體重僅在第一次訪視測量。
b: 於第 1, 29 天使用尿液懷孕檢測。若尿液檢測為陽性，應以血清懷孕檢測再次確認。以血清懷孕檢測替代尿液檢測將不視為試驗偏差。蛋白尿將透過尿液常規檢測確認，做為基礎值。
c: 所有受試者將進行電話安全追蹤以監測非預期性不良事件，包括特殊不良事件，和監測新型冠狀病毒感染的症狀。
d: 受試者將在每周(為每 7 天)由手機接獲一條提示以規律監測新型冠狀病毒感染症狀或病徵。記錄疑似新冠病毒感染所使用的藥物至新冠病毒感染頁面。
e: 只記錄醫療需求不良事件和嚴重不良事件之併用藥物。
f: 第二次接種疫苗後第 28 天
g: 針對同意並進行施打第三劑試驗疫苗的疫苗組受試者
h: 第二次接種疫苗後第十二個月(第 365 天)
i: 安全性實驗室數值包括全血液計數(血紅素、血比容、紅血球計數)、白血球計數、血小板計數、肌酸酐、丙胺酸轉胺酶、天門冬胺酸轉胺酶、總膽紅素、直接膽紅素、高靈敏度 C 反應性蛋白、抗核抗體
j: 主動收集時期
k: 被動監測時期
l: 第一次訪視與第二次訪視可為同一次訪視。
m: 包括接種最後一劑疫苗後 12 個月，可能的免疫媒介醫療狀況(PIMMC)或任何定義為可能的特殊不良事件。新冠病毒感染的併發症也將視為疾病增強事件，將被記錄與通報為特殊不良事件。
n: 若受試者有≥第三級以上的高血壓，受試者將或熱是否有蛋白尿存在或惡化。
o: 備血將被冷凍存放做為 UBI 新型冠狀病毒酵素結合免疫吸附分析法，新型冠狀病毒確認酵素結合免疫吸附分析法，以及未來免疫學研究之用。
p: 若符合資格並接種第三劑疫苗的受試者將進行兩週的日誌卡追蹤紀錄，並將於接種後第 7 天進行電話安全性追蹤。
q: 第五次訪視和第六次訪視可為同一天。
r: 在選定之試驗地點中，將邀請大約 30 位年齡 >18-<65 歲的受試者和大約 30 位年齡 ≥65 歲的受試者。在適用的情況下，優先邀請曾進行過第 57 天 T 細胞功能評估的受試者。
